# Metagenomic insights into the microbial diversity in manganese-contaminated mine tailings and their role in biogeochemical cycling of manganese

**DOI:** 10.1038/s41598-018-26311-w

**Published:** 2018-05-29

**Authors:** Shreya Ghosh, Alok Prasad Das

**Affiliations:** 1 Bioengineering and Bio Mineral processing Laboratory, Centre for Biotechnology, Siksha ‘O’ Anusandhan University, Bhubaneswar, Odisha India; 2 Department of Chemical & Polymer Engineering Tripura University, (A Central University) Suryamaninagar, Agartala, India

## Abstract

To extend the knowledge on the microbial diversity of manganese rich environments, we performed a clone library based study using metagenomic approach. Pyrosequencing based analysis of 16S rRNA genes were carried out on an Illumina platform to gain insights into the bacterial community inhabiting in a manganese mining site and the taxonomic profiles were correlated with the inherent capacities of these strains to solubilise manganese. The application of shot gun sequencing in this study yielded results which revealed the highest prevalence of *Proteobacteria* (42.47%), followed by *Actinobacteria* (23.99%) in the area of study. Cluster of orthologous group (COG) functional category has 85,066 predicted functions. Out of which 11% are involved in metabolism of amino acid, 9% are involved in production and conversion of energy while Keto Encyclopedia of Gene and Genomes (KEGG) functional category has 107,388 predicted functions, out of which 55% are involved in cellular metabolism, 15% are environmental and information processing and 12% are genetic information processing in nature. The isolated microbial consortia demonstrated visible growth in presence of high concentrations of Mn. Solubilisation studies resulted in 86% of manganese recovery after 20 days. The result presented in this study has important implications in understanding the microbial diversity in manganese contaminated mine tailings and their role in natural geochemical cycling of Mn.

## Introduction

Microorganisms are the fundamental form of life on earth and play significant role in soil composition maintenance and metal recycling. Understanding novel metal resistant microorganisms in the mining-environments are valuable for biomining of solid metallic residues and soil remediation^1,2^. Manganese is one of the most abundant elements in the earth and is mostly present in the nature in the form of ores, ocean nodules and crustal rocks which is retrieved by deep site mining. Mn is not only an industrially important metal but also has essential roles to play in the environment^3,4^. It is also one of the most important micronutrients that are required by almost all microorganisms. Ecosystems across the globe are receiving elevated levels of manganese due to the inflated rates of industrial activities. However, bacterial strains play a crucial role in Mn biogeochemical cycling due to the presence of inherent cellular mechanisms aiding in maintaining homeostasis across the ecosystem^5^. Microbial strains are known to possess enzymes like multi copper oxidase and manganese reductase^6^ which can take up excess Mn from the environment and solubilise it in the cell before releasing it to the environment^7–9^. Some bacterial species such as *Bacillus* and *Aerobacter*, release strong chelating agents called “siderophores” into the surroundings which generally act as metal-capturing devise. However their role in the solubilisation process is yet to be established.

The importance of native microbes is well known but very few studies report their vulnerability to environmental disturbances or their capability of resilience^10^. The huge bio diversity and uncultivable nature of certain microorganisms make it immensely difficult for accurate representation of microbial communities in a particular ecological niche. Metagenomics is a revolutionary concept in the aspect of studying microbial bio diversity, their adaptation to the ecological niches and their evolution^11–14^. Metagenomic data sets are obtained by high-throughput sequencing of environmental samples and provides an aggregation of all the conceptually provides all the genetic materials of the studied environment^15^. This strategy easily overcomes the bottle necks associated with conventional molecular methods of retrieving genetic information for a particular environment. High throughput bioinformatic analysis enables the accurate exploration of a gene of interest^16,17^. Comprehensive analysis of varied ecosystems and their resilience to environmental changes can be well documented by the help of metagenomic analysis. In context to the geological and geobiochemical environment, metagenomic approaches have enabled straight forward investigation of the microbiome in deep mining deposits^18,19^. Several studies on the diversity analysis of microbial communities in varied environments like acid-mine drainage, marine water and sediments and soils have been reported^20–22^. These studies have provided novel insights not only on the community structure of a region but also on novel genes, metabolic processes, the evolutionary history of the dwelling microorganisms, the mechanism of their metal tolerance and solubilation abilities^23^. However, our understanding on the response of microbial communities to environmental stresses and contaminants and their role in metal solubilisation continues to be very scanty due to the lack of comprehensive geochemical datasets combined with their metagenomic sequence data.

Widespread mining of manganese due to its industrial applications have led to the depreciation in the ores natural reserves and has also lead to extensive environmental pollution due to metal rich mining waste and residues^24,25^. Moreover a huge amount of this metal is also lost in these wastes which could be easily recycled and reused in industries. Hence, researchers are now resorting to bioleaching of manganese by solubilising activity of native microbial strains^26^. Although cultivation-dependent studies enable the isolation of novel microorganisms, yet cultivation independent strategy leads to a better and accurate assessment of microbial biodiversity thereby providing a clearer picture of the roles they play in metal solubilisation^27^.

The present study was designed to investigate metagenomic insights into the microbial diversity in manganese-contaminated mine tailings and their role in its biogeochemical cycling. We used shotgun-metagenomic based strategy to (a) analyse the microbial communities in dwelling in the low-permeability, nutrient-poor environment, (b) to ascertain the role of the isolated microbial consortia in Mn solubilisation and (c) gain insights into the underlying factors aiding to the adaptation of microbial communities in such heavy metal contaminated environment. In the present investigation, we investigated the diversity of culturable and non-culturable microbial diversity of manganese contaminated mining sites and screened the isolated microbial consortia for their manganese tolerance and bioleaching potential through bioleaching experiments. The results of this study will provide insight into the community structure of the study site and also shed lights on their inherent mechanisms that make them potent for tolerating high metal concentrations in their surroundings. Diversity analysis of such extreme environments has got ample attention due to their diverse ecology that can aid in unravelling the underlying mechanisms of their metal resistance and role in biogeochemical cycling of manganese.

## Results

### Sample characterisation

The mining residues sample was reddish brown in colour and had a dry and rough texture. The collected sample was analysed for the percentage of Mn content using acid digestion by aqua regia and the sample was found to have manganese percentage of 20. EDX analysis indicated the presence of manganese along with Fe and Si.

### Overview of metagenomic sequencing

**Figure 1:**
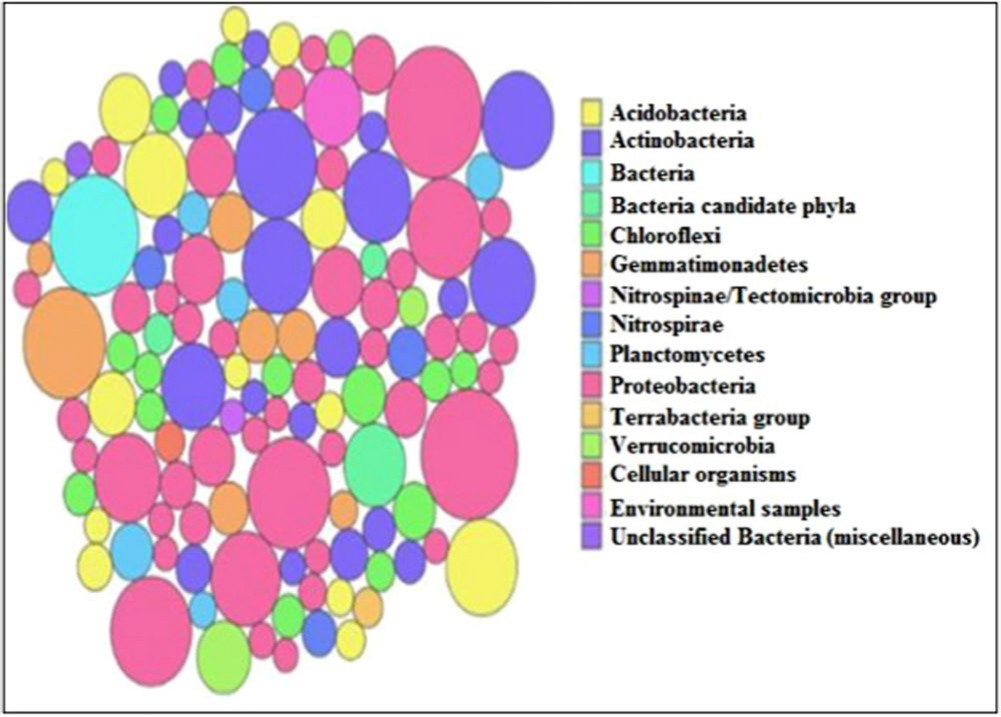
Bubble plot showing the relative taxonomic abundance of the sample. Bubble size indicates taxon abundance relative to its maximum abundance (largest bubble size). The size of the circle is scaled logarithmically to represent the number of ORFs assigned directly to the taxon.

**Figure 2:**
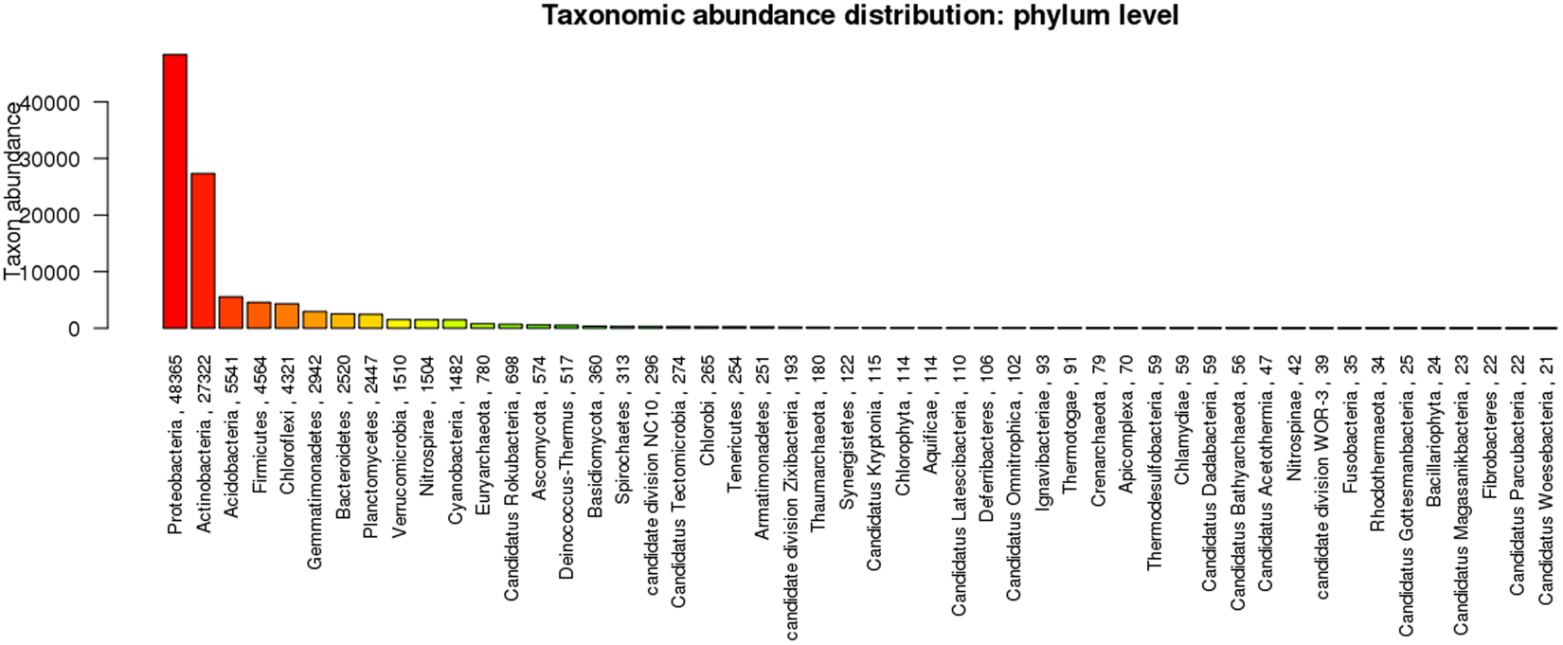
Bar chart showing the taxonomic abundance at phylum level. From the figure it can be inferred that Proteobacteria was found to be most abundant followed by Actinobacteria. X-axis represents the annotated taxa and the number ORFs assigned at the phylum level. Y-axis is the taxon abundance.

**Figure 3:**
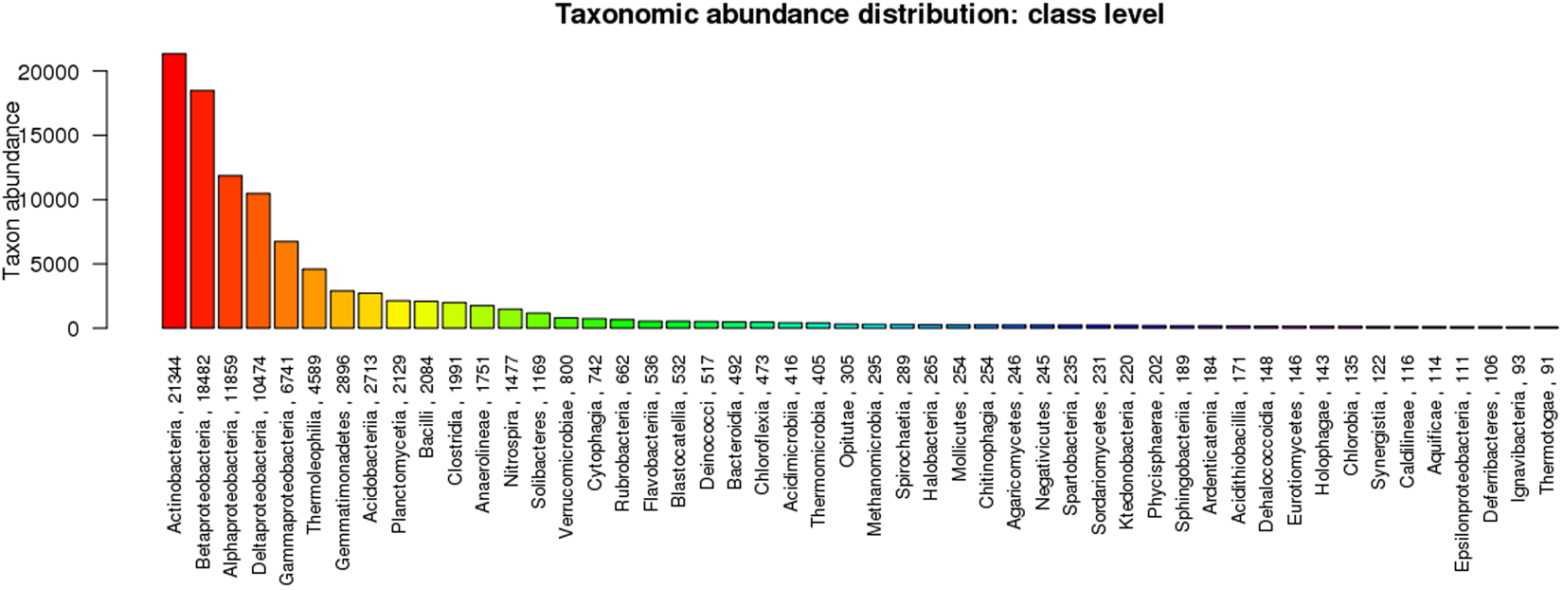
Bar chart showing the taxonomic abundance at class level. From the figure it can be inferred that Actinobacteria was found to be most abundant followed by Betaproteobacteria, Alphaproteobaceria and Deltaproteobacteria. x-axis represents the annotated taxa and the number ORFs assigned at the class level. Y-axis is the taxon abundance.

**Figure 4:**
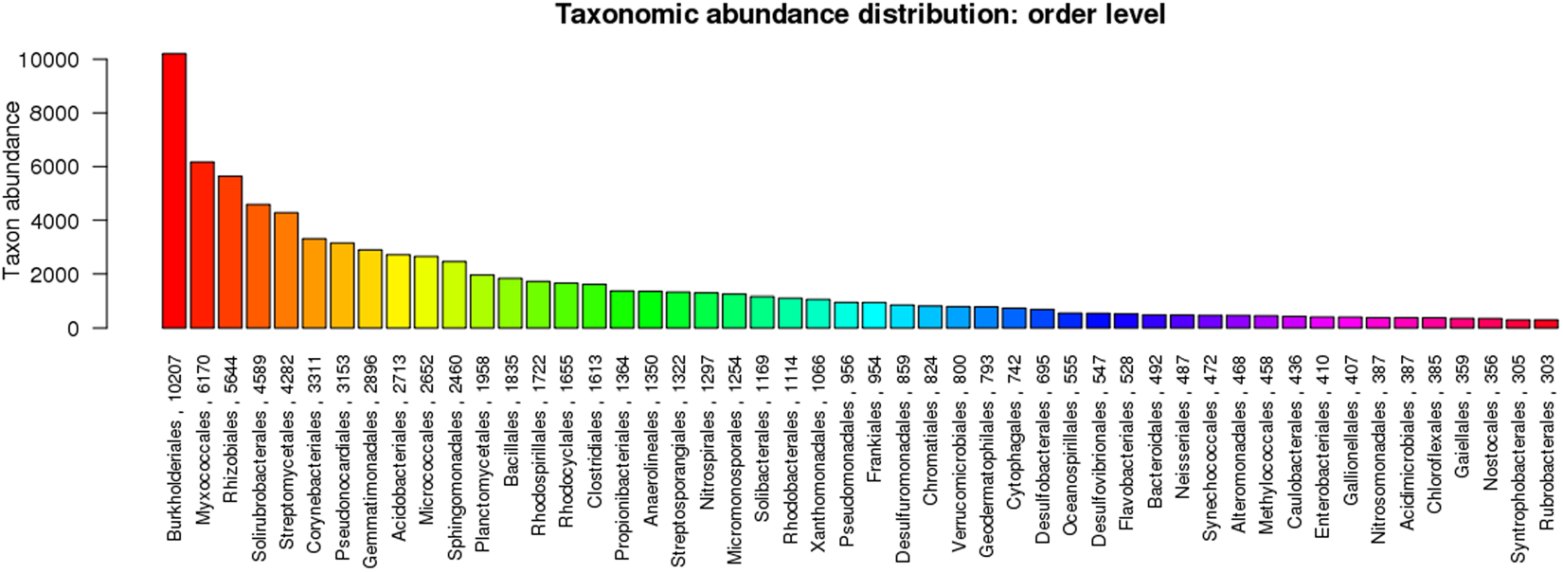
Bar chart showing the taxonomic abundance at order level. From the figure it can be inferred that Burkholderiales was found to be most abundant followed by Bacillales and Acidobacteriale*s*. X-axis represents the annotated taxa and the number ORFs assigned at the order level. Y-axis is the taxon abundance.

**Figure 5:**
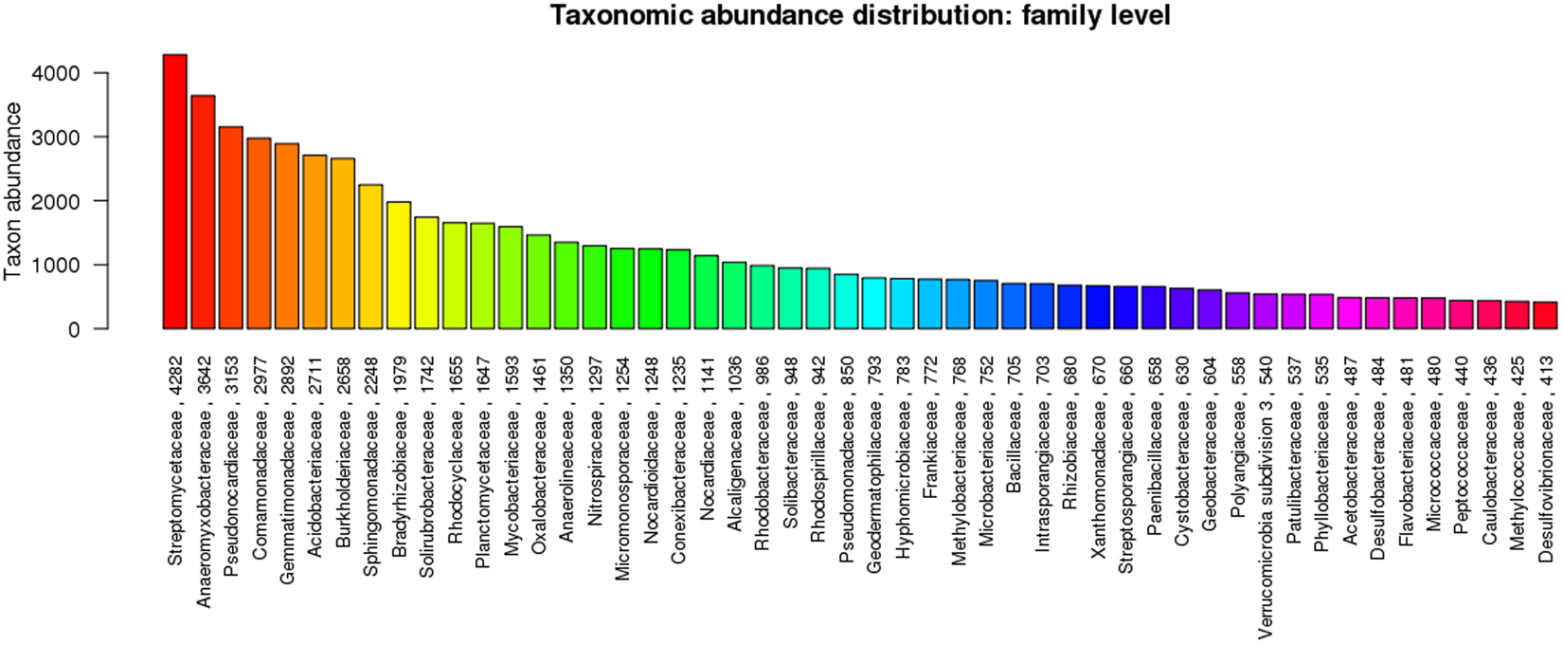
Bar chart showing the taxonomic abundance at family level. From the figure it can be inferred that Streptomycetaceae was found to be most abundant followed Anaeromyxobacteriaceae and Pseudonocardiaceae. x-axis represents the annotated taxa and the number ORFs assigned at the family level. Y-axis is the taxon abundance.

**Figure 6:**
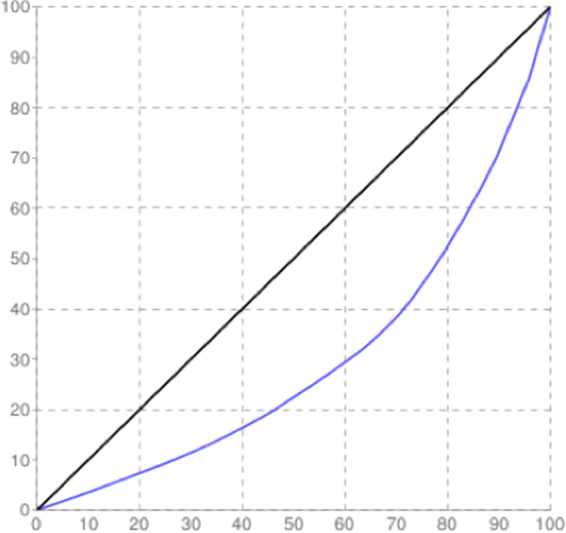
Lorenze graph, a graphical tool depicting the characterization of the concentration of microbial population.

**Figure 7:**
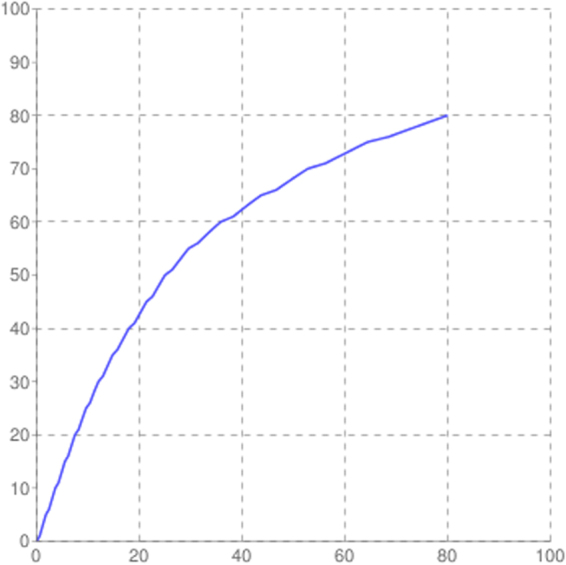
Rarefaction curve depicting species richness of the sample.

**Figure 8:**
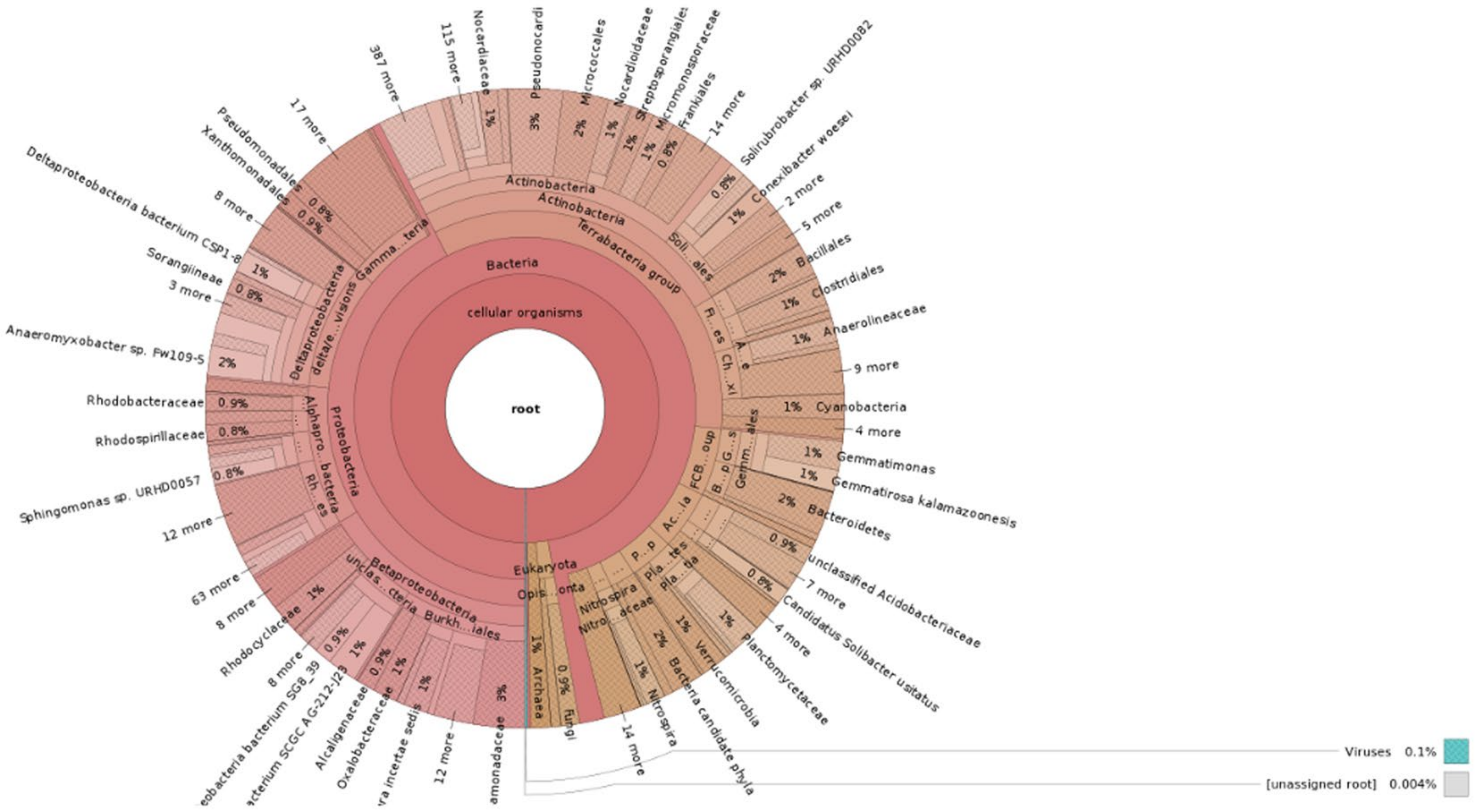
The Krona graph showing the relative abundance of annotated taxa in the sample. Proteobacteria, Terrabacteria group and Actinobacteria were found to be highly enriched.

The mean size of the library was 558 bp and the library was sequenced (2 × 150 bp chemistry). 8,681,960 paired end reads were generated which corresponded to ~2.5–3.0 GB data, deposited in NCBI with Sequence Read Archive accession number PRJNA360904. Abundance and functional groups estimations are critical in metagenomic studies. Their hierarchical contexts and their prediction confidence are very significant considerations due to the uncertainty associated with these group assignments. Bubble plot has been generated which shows the relative taxonomic abundance of mining residue sample (Fig. 1). The size of the bubble corresponds to taxon abundance relative to its maximum abundance (largest bubble size). From the figure it can be inferred that Proteobacteria, Actinobacteria and Acidobacteria were found to be highly enriched. Phylum level taxonomic hits distribution of top 50 shows that mining residue sample has 48,365 Proteobacteria followed by 27,322 Actinobacteria (Fig. 2). Class level taxonomic hits distribution of top 50 shows that mining residue sample has 21,344 Actinobacteria followed by 18,482 Betaproteobacteria, 11,859 Alphaproteobacteria 10,474 Deltaproteobacteria (Fig. 3). Actinobacteria was found to be most abundant followed by Betaproteobacteria, Alphaproteobaceria and Deltaproteobacteria. Order level taxonomic hits distribution reports the presence of 10,207 Burkholderiales followed by 6,170 Myxococcales, 5,644 Rhizobiales (Fig. 4). Burkholderiales was found to be most abundant followed by Bacillales and Acidobacteriales. Members from the Streptomycetaceae family (4,282) was predominant in the sample, followed by Anaeromyxobacteriacea (3,642) and Pseudonocardiaceae (3,153) (Fig. 5). Streptomycetaceae was found to be most abundant followed by Anaeromyxobacteriaceae and Pseudonocardiaceae. The majority if the genus level taxonomic hits was occupied by Streptomyces (3,759) followed by Anaeromyxobacter (3,642). Species level taxonomic hits distribution of top 50 showed that mining residue sample has 1,718 Anaeromyxobacter sp. Fw109-5, followed by 1,702 Betaproteobacteria bacterium SCGC AG-212-J23 and 1,250 Deltaproteobacteria bacterium CSP1-8. The Shannon diversity index was found to 5.2 which implies the presence of a high level of microbial diversity in the analyzed sample. The rarefaction curve and lorenze graph are presented in Figs 6 and 7. Krona aids in the estimation relative abundances even within complex metagenomic classifications. The Krona graph showing the relative abundance of annotated taxa in mining residue sample is presented in Fig. 8. Proteobacteria, Terrabacteria group and Actinobacteria were found to be highly enriched. To perform a taxonomic analysis, the MEGAN program places the ORFs onto the NCBI taxonomy. A total of 170,647 ORFs along with 8 rRNAs of mining residue samples were subjected to taxonomical analysis. Megan taxonomic analysis was performed at phylum and genus level and Proteobacteria was found to be most abundant followed by Actinobacteria.

### Functional category hits distribution of mining residue sample

**Figure 9:**
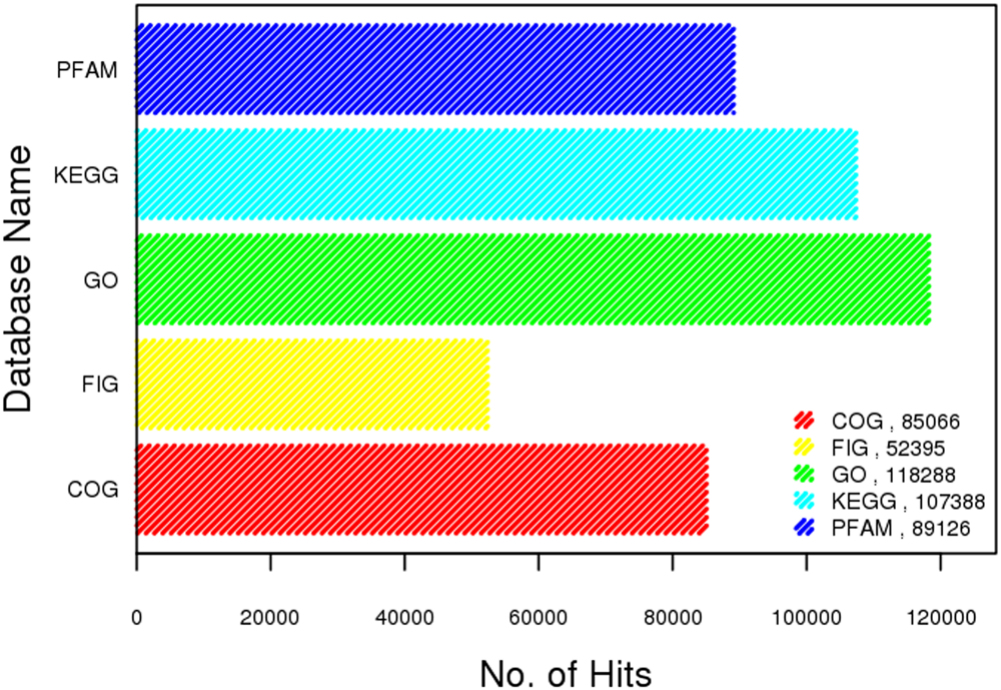
Source hits distribution of the sample. Each number indicated represents the number of ORFs annotated against a particular database. Maximum number of hits was observed against GO database, followed by KEGG and PFAM.

**Figure 10:**
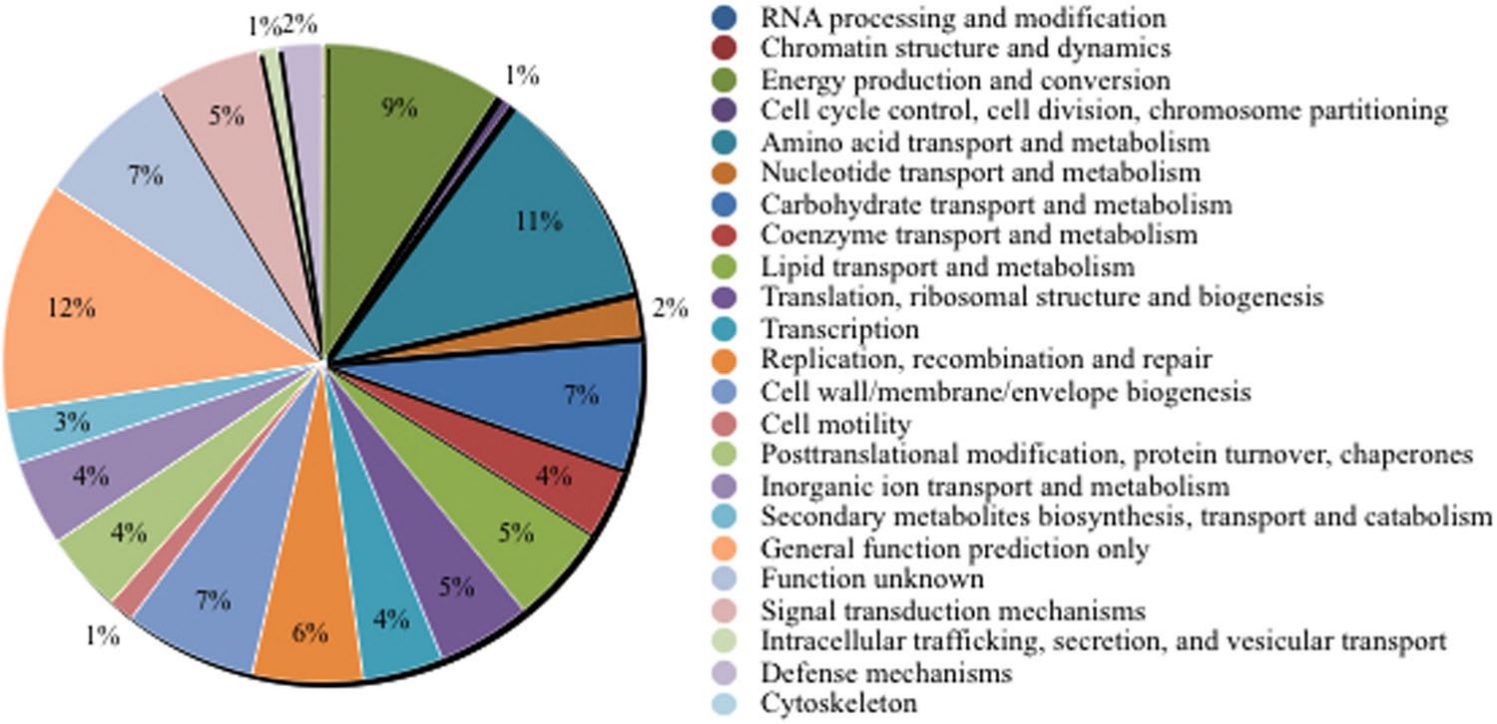
COG functional category hits distribution of the sample.

**Figure 11:**
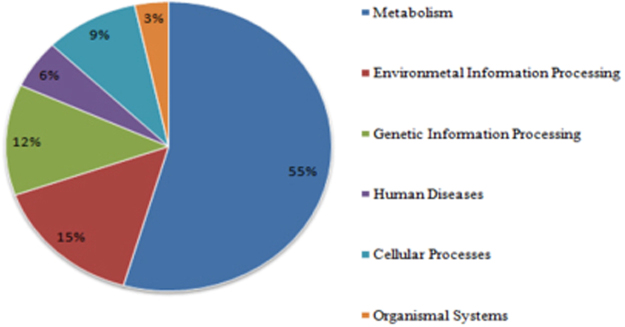
KEGG functional category hits distribution of the sample.

The maximum number of hits was observed against GO database, followed by KEGG and PFAM database (Fig. 9). COG Functional Category has 85,066 predicted functions. Out of which 11% are Amino acid transport and metabolism, 9% are energy production and conversion as shown in below (Fig. 10). While KEGG Functional Category has 107,388 predicted functions, out of which 55% are metabolism, 15% are Environmental and Information Processing and 12% are Genetic Information Processing (Fig. 11) (supplementary information). Pfam functional annotation is used to assess protein families and domain. PFAM functional analysis has 89,126 predicted functions against a manually curated Pfam database. FIGfams functional analysis has 52,395 predicted functions against manually annotated FIGfams database. FIGfams functional annotation is used to find the families of proteins that have the same functions. GO functional analysis has 118,288 predicted functions against GO database.

### Manganese tolerance studies

Experimental results revealed that the microbial strains in the bacterial consortia could sustain visible growth upto Mn concentration of 500 mM. Beyond this concentration, the number of colonies observed were substantially reduced. No growth was observed beyond 1000 mM Mn concentration. The bacterial strains of the community dwelling in the sample selection site showed high level of tolerance to manganese. This may be due to the inherent mechanisms they have evolved while growing in such extreme metal rich environments.

### Manganese solubilisation

**Figure 12:**
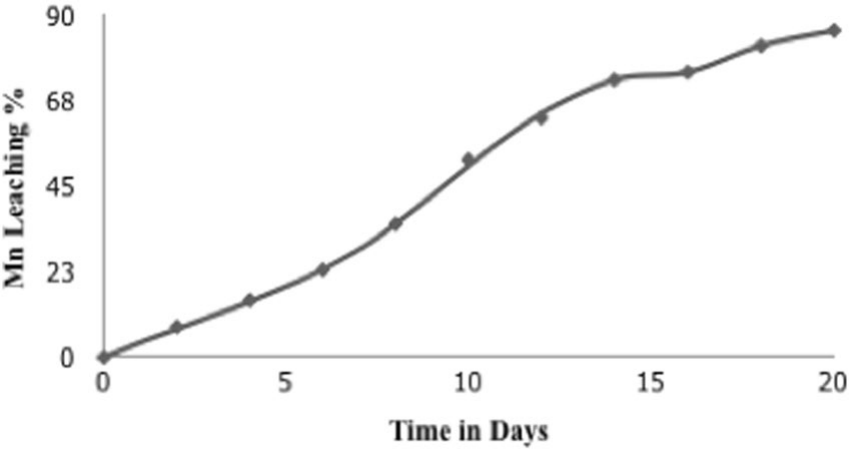
Manganese solubilisation using microbial consortia. 86% of Mn was recovered after 20 days.

**Figure 13:**
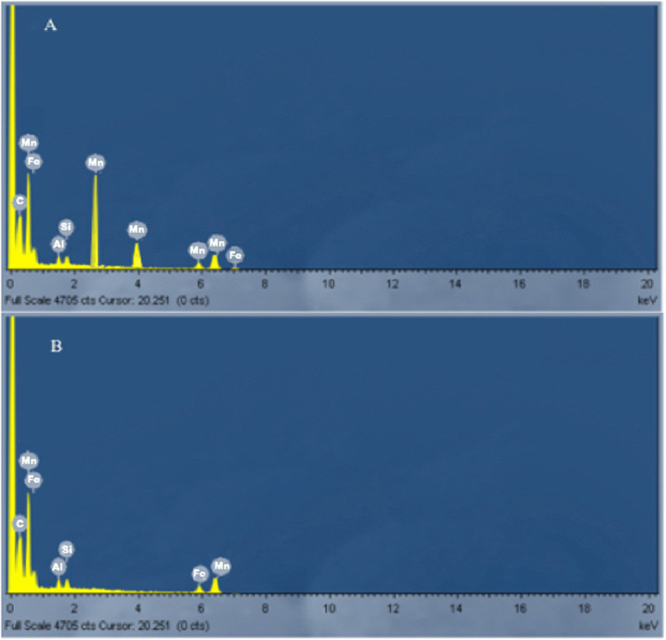
Visualization of metallic constituents by energy dispersive X-ray spectrometry of the ore sample. (**A**) Before bio solubilisation studies; (**B**) After bio solubilisation studies.

Solubilisation experiments using native microbial consortia resulted in 86% of recovered Mn was after 20 days (Fig. 12). The bioleaching rate in the first 7 days was slower in comparison to the rate acquired after the 9^th^ day. The isolation of native bacterial consortia contributes to the high percentage of recovery due to the metal tolerance and solubilisation mechanisms that these species have generally evolved with^28,29^. No noteworthy results were observed in the control experimental set up. The EDX analysis of the original ore sample showed the presence of several peaks of Mn. However, the EDX analysis after bioleaching studies depict diminished Mn peaks validating the bio recovery of Mn during the course of study (Fig. 13). The ability of microbial strains to tolerate and thus solubilize heavy metals is supported by five basic mechanisms including enzymatic conversion, metal effluxing and reduction in sensitivity of cellular targets, intra or extracellular sequestration and permeability barrier exclusion. These microorganisms possess a variety of active and intrinsic metal resistance systems that enables them to grow in very high metal concentrations^28^. The metallic constituents of the ore are solubilised by bacterial cells by direct or indirect mechanism. In case of indirect bioleaching, there is no physical contact between microorganism and mineral particles and there is the formation of reductive metabolic compounds, organic carbon and energy sources. In some cases, bacteria require direct contact with the ore constituents to solubilise the metals. Some strains also use enzymatic machinery to solubilise metals conjugated in the ore samples^8,30^.

## Discussion

In the present study, the microbial biodiversity in manganese mining deposits of Odisha in the Indian subcontinent was investigated by using a metagenomic approach. The sample was analysed to ascertain the dominant phyla of microorganisms inhabiting in extreme metal rich conditions prevalent in the sampling region. The dominant phylum in sample was *Proteobacteria* (42.47%), followed by *Actinobacteria* (23.99%). These two groups of microorganisms are frequently found in most of the soil samples. A high value of Shannon index of 5.2 was obtained which implies the richness of the sample in terms of biodiversity. Our previous studies on the isolation of cultivable manganese solubilising bacteria reported the presence of *Acinetobacter sp*. and *Enterobacter sp*. belonging to phyla Proteobacteria and *Lysinibacillus sp., Bacillus sp., Bacillus cereus, Bacillus nealsonnii* and *Staphylococcus hominis* belonging to Firmicutes^28,31^. The reports of this study are somewhat consistent with the previous results. However this study sheds light on the presence of uncultivable phyla of strains namely *Cidobacteria*, *Verrucomicrobia*, *Bacteroidetes*, *Chloroflexi*, *Planctomycetes* and *Gemmatimonadetes* that were not known previously. Janssen *et al*.^32^, carried out a metagenomic study on 16S rRNA gene sequences obtained from mining residue samples and observed that *Proteobacteria* (39%) and *Acidobacteria* (19%) were the most abundant members followed by *Verrucomicrobia*, *Bacteroidetes*, *Chloroflexi*, *Planctomycetes*, *Gemmatimonadetes*, and *Firmicutes*. All of these phyla are represented in the sample used in the present study, although in different proportions. A high level of variability in the abundance of different phyla and class members in different samples has been reported. It is however not very clear whether the variations are systematic in response to the prevailing environmental conditions. Integration of the biotic and abiotic influences on biodiversity of a community is essential for correct evaluation of a microbial ecosystem. While soil moisture influences Verrucomicrobia abundance^33^, the pH of oil controls the abundance of acidobacteria. Several factors such as Ca^2+^/Mg^2+^ ratio, altitude, Aluminium and phosphorus content also influences the microbial biodiversity of site^2^. Thus it is very significant to understand the influence of abiotic properties for a better understanding of microbial biodiversity and answering the hidden reasons behind community changes.

**Figure 14:**
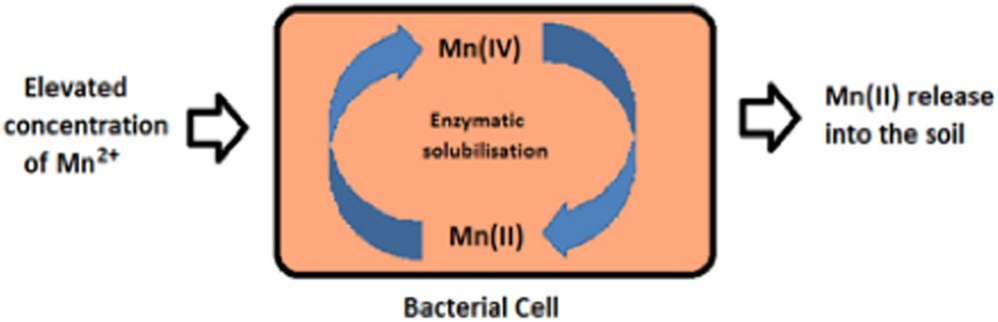
Schematic representation of bacterial solubilisation of manganese.

Manganese occupies a central position in various biological processes owing to its redox properties. It is often a part of the biogeochemical cycling process, mediated by abiotic and biotic components of the nature. Varied group of bacterial strains are capable of solubilising it effectively and can compete with chemical oxidation. Mn oxides are strong oxidants in nature and therefore control the fate of several elements^9^. Enzymatic mechanisms in specific bacterial strains help them to scavenge this metal ion and associated elements, thereby playing a crucial role in biogeochemical cycles. Bacterial strains mostly carry out solubilisation through direct and enzymatic mechanism which involves direct contact of the ore surface with the bacteria and the enzymatic machinery within the bacterial strains^8,24^. Mn solubilising microbial strains are known to possess various metal solubilising enzymes like multi copper oxidase and manganese reductase (Fig. 14)^6,9^ which can take up excess metal ions from the environment and solubilise it in the cell before releasing it to the environment^7,8,34,35^. Genes like CumA, mnxC, mnxD coding for multicopper oxidases have been reported to be involved in bacterial manganese solubilisation. Indirect pathway involving the secretion of organic acids is also responsible to some extent in the bacterial biotransformation of Mn in the environment^36^. It is also an essential nutritional requirement in many living organisms, aiding in their growth and survival. It plays central role in redox reactions and oxygenic photosynthesis taking place in cyanobacteria^37^, protects it from metal toxicity and UV light radiations, scavenging micronutrients, catabolism of organic matter, production of oxygen, and protection against reactive oxygen species in bacteria^38^.

Proteobacteria, Actinobacteria and Firmicutes groups of bacteria have been known to possess manganese solubisling activity^31^. However, the ecology of this group of microorganisms is still enigma^39^. The COG analysis reports enzyme like multi copper oxidase, peroxidase, ferric reductase and several other classes of reductase which have been known to play a significant role in manganese solubilisation. The FIG and Pfam analysis reveals the presence of heat shock proteins from the family HSP 20, 60, 70 and 90 which are essential proteins which enable microbial strains to cope up with stress conditions^40,41^. Considering that the bacterial strains were isolated from metal rich environments, hence the presence of HSPs is very likely. GO analysis reveals several gene functions like manganese ion binding, metal ion transport, high-affinity iron ion transmembrane transport, regulation of pH, copper oxidase activity and response to stress^42^. These functions are essential for metal solubilisation and are especially true in the case of ferro manganese ores which is found in bounty in the area of study. Mn tolerance studies demonstrated that the bacterial consortia could support visible growth upto concentration of 500 mM, beyond which the growth decreases drastically and finally ceases at concentration of 1000 mM. The bioleaching studies carried out using bacterial consortia also agrees to the above findings. Mn recovery of 87% was observed in 20 days which well highlights the solubilisation potential of the bacterial biodiversity of the mining deposits. Although there are reports on different mechanisms in different strains, bacterial strains are known to solubilise Mn by a direct pathway which involves intracellular enzymatic processes. The present study therefore correlates well with the fact that the native bacterial strains of this region possess inherent capacity to solubilise manganese through their cellular mechanisms and play a noteworthy role in the biogeochemical cycling of manganese.

Excess concentrations of heavy metals in the environment leads to the evolving of cellular mechanisms to resist and tolerate metal load. The microorganisms aims to avoid metal toxicity symptoms by using various mechanisms that are usually specific for a particular species. Some mechanisms of resistance are encoded in the plasmid and are somewhat specific for a particular metal while other mechanisms might lead to multi metal resistance. Exopolymers or extracellular polymeric substances (EPSs) are produced by several microbial strains that can strongly bind to metal ions and can mobilize or immobilize the toxic metals^43^. These exopolymers are more efficient in binding to heavy metals, such as lead, cadmium and uranium. Siderophores another types of extracellular molecule capable of binding to metals. They are iron-complexing organic compounds that can concentrate iron in low iron environments and can lead to its transport into the cell. Biosurfactants are also produced by microbial strains and they have now been recognised for their ability of complexing metals such as cadmium, lead, and zinc. Biosurfactant-producing microorganisms are mostly found in metal-contaminated environments than from uncontaminated ones^44^. Some organic or inorganic acids produced by certain microorganisms like *Thiobacillus, Penicillium, Bacillus* and *Aspergillus* are capable of extracting metals from solid substrates. Toxic metals can be mobilized under anaerobic conditions as co precipitates with iron oxides and this co precipitation of toxic metals with ferric iron can aid in bioremediation of high metal content waste streams^45^. Although mining is one of the major causes for heavy metal pollution, the mining sites serve as a rich bed of microbial diversity that has evolved to elicit several metal tolerant mechanisms and have metabolic processes that can aid in the remediation of metal polluted sites and can also double as a solubilisation technique for metals from mining wastes and effluents.

## Conclusion

Environmental conditions occupy a key role in structuring of microbial communities in mining environment and also aids in their functional adaptations. Metagenomic analysis of the bacterial diversity of manganese mining deposits was investigated to assess their role in biogeochemical cycling of Mn. Taxonomic analysis by Kaiju revealed that Phylum Proteobacteria was found to be most enriched followed by Actinobacteria in the sample while at species level, Anaeromyxobacter sp. Fw109-5 was found to be most enriched followed by Betaproteobacteria bacterium SCGC AG-212-J23 and Deltaproteobacteria bacterium CSP1-8. Taxonomic analysis was also validated using MEGAN and the results obtained, correlated with the Kaiju report. COG functional analysis revealed genes that are involved in transport and metabolism amino acid and production and conversion of energy in the cell. KEGG functional analysis showed that mining residue sample was enriched in genes that are involved in Metabolism, Environmental Information Processing and Genetic Information Processing. The bacterial consortia demonstrated high level of Mn tolerance and solubilisation thereby indicating their adaptation to Mn rich environment. Marked signatures at the community level and the speculated gene functions of the microbial community suggest that the microbial biodiversity in mining sites endow them with a selective advantage in metal rich conditions and aid in the biogeochemical cycling. Because microbial biodiversity analysis in mining environment and their possible role in mineral geochemical cycling has been lacking scientific interest, this study provides useful information for investigating microbial biogeochemical relationships in these ecosystems, unearthing of rare genes and industrially important mechanism of microbial strains and metagenomic insights into their community structure. The presented metagenomic analysis provides a first insight into the uncultured microbial diversity living in extreme manganese rich environments. Culture independent molecular approaches would enable a more comprehensive assessment of microbial biodiversity and their biogeochemical role in mining ecosystems in the near future. We hope this study can pave the way for the dissemination of improved isolation strategies for metal solubilising microbial strains, crucial in obtaining evidence for their proposed biogeochemical role.

## Methods

### Sample collection

Manganese mining deposits in Odisha State of India are situated in the Barbil district (latitude and longitude: 21°9′North and 85°29′East). The manganese ores found in this region are mostly low to medium grade type having low phosphorous content^46^. The 35 manganese mines located in Odisha, spread over an area of 21745.52 hectares, has a combined annual Mn production of around 660 Th. tonnes. The mining method prevalent in this region is mostly traditional and labour intensive. The degree of mechanisation in mining in Odisha has not reached a saturation or near-saturation level as observed in developed countries. Extensive deposits and ongoing mining operations in this region makes it a suitable study site for analysis of metal solubilising microbial diversity. Mining residue samples from mining deposits of Odisha were collected in May 2016 for carrying out experiments on microbial diversity analysis and bio solubilisation of Mn. Samples were collected along the mining sites of Odisha in the Barbil district which transverses 20 km of an area. For sample collection, the site was cleaned superficially to remove organic matter and the mining residues in a circle of approximately 50 cm in diameter, from 0 to 20 cm in depth, was thoroughly mixed. Two replicated samples (approximately 500 g) from the deposits were collected and stored in sterile containers. The samples were stored at 4 °C for 72 hours, protected from contamination sources until they were used for experimental studies. The representative samples were subjected to acid digestion using aqua regia for determination of its metal contents. Energy dispersive X-ray analysis (EDX) was carried out to evaluate the elemental composition of the collected sample.

### DNA isolation and sequencing

Genomic DNA isolation of the mining residue samples was carried out using Xcelgen gDNA kit and its quality was checked on 1% Agarose gel. A 260/280 ratio was determined using Nanodrop 8000, and the DNA concentration was determined using Qubit® 2.0 Fluorometer. Illumina Library Preparation Kit (TruSeq Nano DNA HT) was used for the library preparation. Fragmentation of 200 ng gDNA was carried out by Covaris and then they were subjected to end-repair. Removal of the 3′ overhangs is carried out by the exonuclease activity of the repair mix. The 5′ overhangs are however filled by its polymerase activity. Indexing adapters ligated to DNA fragments were prepared for hybridization onto a flow cell. The products were purified and amplified using PCR. The library formed was then analyzed in Bioanalyzer 2100 (Agilent Technologies).

Qubit concentration for the library was obtained from Bioanalyser profile. Cluster generation and sequencing of the library was carried out on an Illumina platform. Bi directional sequencing was achieved by paired-End sequencing of the template fragments. Complementary adapter oligos get bound by library molecules on paired-end flow cell. Selective cleavage of the forward strands is carried out by adapters after the accomplishment of reverse strand re-synthesis. The copied reverse strand then acts as a template to generate a sequence from the other end of the fragment.

### Metagenome assembly and ORF prediction

The mining residue sample data was assembled in CLC Genomics Workbench 6.0. A minimum scaffold length of 200 was used and the mismatch cost, insertion cost and deletion cost were set at 2, 3 and 3 respectively. A length fraction of 0.5 was used while a similarity fraction of 0.8 was set while carrying out metagenomic assembly. The statistical elements of the assemblies were calculated by using perl scripts are given below.

Number of Scaffolds: 176,978

Genome length (Mb): 82.48

Average Scaffolds size: 466.05

Scaffold N50: 416

Max Scaffold size: 20,960

Total rRNAs were predicted for mining residue sample by aligning the assembled scaffolds against in-house RNA database using BlastN. The Open Reading Frames were predicted for the mining residue sample using Prodigal (v2.6.1). The predicted ORFs along with their rRNAs containing scaffolds were then taken for Taxonomic and Functional Annotation.

### Taxonomic and functional metagenomic annotation

Taxonomic and functional metagenomic studies provide significant perception of the community structure and function of a particular region. This in turn aids in the exploitation of the dwelling microbial life for a wide array of applications based on their inherent metabolic capacities. Kaiju is a sensitive metagenome classifier which enables the deduction of protein level matches and works on Burrows–Wheeler transform algorithm^47^. A total of 170,647 ORFs along with 8 rRNAs of mining residue samples were uploaded in the Kaiju Web-server and certain parameters were selected. The run mode was selected as greedy, having a minimum match length of 15, minimum match score of 65 and 5 allowed mismatches. Bacterial diversity analysis was accomplished by calculating by the Shannon diversity index (*H*), by the Al Young studios program. The rarefaction and Lorenz curve was also plotted. To assess the functional capacities of microbial communities, COGNIZER was used. Cognizer is a comprehensive stand-alone framework which is enabled to simultaneously provide COG, KEGG, Pfam, GO and FIGfams annotations to individual sequences constituting metagenomic datasets. A total of 170,647 ORFs along with 8 rRNAs of sample were subjected to functional annotation.

### Mn solubilisation

In nature Mn occurs in three different oxidation states. Biotic and abiotic components both play important roles in the oxidation and reduction of Mn. Therefore, a major challenge in understanding Mn biotransformation is the differentiation and subsequent quantification of these components of the processes. The collected mining samples were acclimatised in manganese supplemented liquid growth media. This was followed by a 7 day harvesting of the inherent bacterial strains. Mn tolerance studies were carried using agar plates supplemented with varying concentration of Mn ranging from 100 mM to 1000 mM. The plates were analysed for the number of bacterial colonies after 48 hours of incubation. Manganese solubilisation studies were carried out to ascertain the role of native microbial consortia in the natural biogeochemical cycling of manganese. Solubilisation experiments were carried out in 500 ml erlenmeyer flasks containing 225 ml modified K medium having a composition of (g/l): (MnSO_4_ 0.3, yeast extract 0.7 and peptone 2.2) and 25 ml of the bacterial consortium that was harvested from the mining sample. The media was supplemented with low grade ores collected from mining deposits at a pulp density of 2% (w/v). The experiments were carried out for 21 days under continuous shaking conditions. A flask containing growth media but devoid of low grade ore served as control. Aliquots of 5 ml were collected regularly from the experimental flasks at the interval of 3 days. They were centrifuged at 10000 RMP for 15 minutes and the supernatant obtained was used to check the amount of Mn solubilised by using titration method. Finally, the leached liquor was analysed for the Mn solubilisation percentage at the end of 21 days using ICP AES (6550 I-Funnel QTOF LC-MS/MS coupled with Agilent 1260 Infinity Nanoflow pump). Aliquot of 10 ml was collected from the final experimental flask which was subjected to ICP AES analysis. On the other hand, the residue ore particles obtained after filtering the leached liquor was dried and analysed for their metal constituents using EDX analysis and the results obtained were correlated to the EDX analysis of the low grade ore sample prior to validate the potential of the isolated bacterial consortia in Mn solubilisation^8,30^.

## Electronic supplementary material

Supplementary information
