# Supplementary material for: Metagenomic insights into the microbial diversity in manganese-contaminated mine tailings and their role in biogeochemical cycling of manganese
Source: Sci Rep. 2018 May 29;8:8257. doi: 10.1038/s41598-018-26311-w (PMC5974364; doi:10.1038/s41598-018-26311-w)
Supplement: Supplementary file 1 — Supplementary information [file 41598_2018_26311_MOESM1_ESM.pdf]

**Metagenomic insights into the microbial diversity in Manganese-contaminated mine tailings and their role in biogeochemical cycling of Manganese**

**Shreya Ghosh[1] and Alok Prasad Das[2*]**

1. Bioengineering and Bio Mineral processing Laboratory, Centre for Biotechnology,

Siksha 'O' Anusandhan University, Bhubaneswar, Odisha

2. Department of Chemical & Polymer Engineering

Tripura University, (A Central University) Suryamaninagar, Agartala, India

Email: alokprasaddas@tripurauniv.in, Phone no. 9178581814

| COG ID | Sequence count | COG domain |
|---|---|---|
| COG0001 | 58 | Glutamate-1-semialdehyde aminotransferase |
| COG0002 | 44 | Acetylglutamate semialdehyde dehydrogenase |
| COG0003 | 43 | Oxyanion-translocating ATPase |
| COG0004 | 59 | Ammonia permease |
| COG0005 | 48 | Purine nucleoside phosphorylase |
| COG0006 | 114 | Xaa-Pro aminopeptidase |
| COG0007 | 23 | Uroporphyrinogen-III methylase |
| COG0008 | 99 | Glutamyl- and glutaminyl-tRNA synthetases |
| COG0009 | 33 | Putative translation factor (SUA5) |
| COG0010 | 51 | Arginase/agmatinase/formimionoglutamate hydrolase, arginase family |
| COG0011 | 7 | Uncharacterized conserved protein |
| COG0012 | 29 | Predicted GTPase, probable translation factor |
| COG0013 | 69 | Alanyl-tRNA synthetase |
| COG0014 | 25 | Gamma-glutamyl phosphate reductase |
| COG0015 | 56 | Adenylosuccinate lyase |
| COG0016 | 40 | Phenylalanyl-tRNA synthetase alpha subunit |
| COG0017 | 26 | Aspartyl/asparaginyl-tRNA synthetases |
| COG0018 | 64 | Arginyl-tRNA synthetase |
| COG0019 | 71 | Diaminopimelate decarboxylase |
| COG0020 | 25 | Undecaprenyl pyrophosphate synthase |
| COG0021 | 77 | Transketolase |
| COG0022 | 82 | Pyruvate/2-oxoglutarate dehydrogenase complex, dehydrogenase (E1) component, eukaryotic type, beta subunit |
| COG0023 | 3 | Translation initiation factor 1 (eIF-1/SUI1) and related proteins |
| COG0024 | 30 | Methionine aminopeptidase |
| COG0025 | 23 | NhaP-type Na+/H+ and K+/H+ antiporters |
| COG0026 | 16 | Phosphoribosylaminoimidazole carboxylase (NCAIR synthetase) |
| COG0027 | 2 | Formate-dependent phosphoribosylglycinamide formyltransferase (GAR transformylase) |
| COG0028 | 232 | Thiamine pyrophosphate-requiring enzymes [acetolactate synthase, pyruvate dehydrogenase (cytochrome), glyoxylate carboligase, phospl |
| COG0029 | 29 | Aspartate oxidase |
| COG0030 | 34 | Dimethyladenosine transferase (rRNA methylation) |
| COG0031 | 88 | Cysteine synthase |
| COG0033 | 21 | Phosphoglucomutase |
| COG0034 | 52 | Glutamine phosphoribosylpyrophosphate amidotransferase |
| COG0035 | 13 | Uracil phosphoribosyltransferase |
| COG0036 | 19 | Pentose-5-phosphate-3-epimerase |
| COG0037 | 46 | Predicted ATPase of the PP-loop superfamily implicated in cell cycle control |
| COG0038 | 26 | Chloride channel protein EriC |
| COG0039 | 27 | Malate/lactate dehydrogenases |
| COG0040 | 26 | ATP phosphoribosyltransferase |
| COG0041 | 19 | Phosphoribosylcarboxyaminoimidazole (NCAIR) mutase |
| COG0042 | 37 | tRNA-dihydrouridine synthase |
| COG0043 | 89 | 3-polyprenyl-4-hydroxybenzoate decarboxylase and related decarboxylases |
| COG0044 | 98 | Dihydroorotase and related cyclic amidohydrolases |
| COG0045 | 30 | Succinyl-CoA synthetase, beta subunit |
| COG0046 | 57 | Phosphoribosylformylglycinamidine (FGAM) synthase, synthetase domain |
| COG0047 | 18 | Phosphoribosylformylglycinamidine (FGAM) synthase, glutamine amidotransferase domain |
| COG0048 | 5 | Ribosomal protein S12 |
| COG0049 | 11 | Ribosomal protein S7 |
| COG0050 | 10 | GTPases - translation elongation factors |
| COG0051 | 5 | Ribosomal protein S10 |
| COG0052 | 18 | Ribosomal protein S2 |
| COG0053 | 75 | Predicted Co/Zn/Cd cation transporters |
| COG0054 | 15 | Riboflavin synthase beta-chain |
| COG0055 | 19 | F0F1-type ATP synthase, beta subunit |
| COG0056 | 32 | F0F1-type ATP synthase, alpha subunit |
| COG0057 | 29 | Glyceraldehyde-3-phosphate dehydrogenase/erythrose-4-phosphate dehydrogenase |
| COG0058 | 90 | Glucan phosphorylase |
| COG0059 | 16 | Ketol-acid reductoisomerase |
| COG0060 | 83 | Isoleucyl-tRNA synthetase |
| COG0061 | 30 | Predicted sugar kinase |
| COG0062 | 16 | Uncharacterized conserved protein |
| COG0063 | 23 | Predicted sugar kinase |
| COG0064 | 50 | Asp-tRNAAsn/Glu-tRNAGln amidotransferase B subunit (PET112 homolog) |
| COG0065 | 48 | 3-isopropylmalate dehydratase large subunit |
| COG0066 | 20 | 3-isopropylmalate dehydratase small subunit |
| COG0067 | 20 | Glutamate synthase domain 1 |
| COG0068 | 44 | Hydrogenase maturation factor |
| COG0069 | 56 | Glutamate synthase domain 2 |
| COG0070 | 20 | Glutamate synthase domain 3 |
| COG0071 | 60 | Molecular chaperone (small heat shock protein) |
| COG0072 | 65 | Phenylalanyl-tRNA synthetase beta subunit |
| COG0073 | 22 | EMAP domain |
| COG0074 | 25 | Succinyl-CoA synthetase, alpha subunit |
| COG0075 | 64 | Serine-pyruvate aminotransferase/archaeal aspartate aminotransferase |
| COG0076 | 83 | Glutamate decarboxylase and related PLP-dependent proteins |
| COG0077 | 25 | Prephenate dehydratase |
| COG0078 | 43 | Ornithine carbamoyltransferase |
| COG0079 | 56 | Histidinol-phosphate/aromatic aminotransferase and cobyric acid decarboxylase |
| COG0080 | 10 | Ribosomal protein L11 |
| COG0081 | 16 | Ribosomal protein L1 |
| COG0082 | 34 | Chorismate synthase |
| COG0083 | 7 | Homoserine kinase |
| COG0084 | 27 | Mg-dependent DNase |

| | | | | |
|---|---|---|---|---|
| COG0085 | 74 | DNA-directed RNA polymerase, beta subunit/140 kD subunit | | |
| COG0086 | 82 | DNA-directed RNA polymerase, beta' subunit/160 kD subunit | | |
| COG0087 | 32 | Ribosomal protein L3 | | |
| COG0088 | 27 | Ribosomal protein L4 | | |
| COG0089 | 17 | Ribosomal protein L23 | | |
| COG0090 | 28 | Ribosomal protein L2 | | |
| COG0091 | 19 | Ribosomal protein L22 | | |
| COG0092 | 19 | Ribosomal protein S3 | | |
| COG0093 | 11 | Ribosomal protein L14 | | |
| COG0094 | 15 | Ribosomal protein L5 | | |
| COG0095 | 12 | Lipoate-protein ligase A | | |
| COG0096 | 21 | Ribosomal protein S8 | | |
| COG0097 | 26 | Ribosomal protein L6P/L9E | | |
| COG0098 | 13 | Ribosomal protein S5 | | |
| COG0099 | 14 | Ribosomal protein S13 | | |
| COG0100 | 7 | Ribosomal protein S11 | | |
| COG0101 | 23 | Pseudouridylate synthase | | |
| COG0102 | 15 | Ribosomal protein L13 | | |
| COG0103 | 15 | Ribosomal protein S9 | | |
| COG0104 | 43 | Adenylosuccinate synthase | | |
| COG0105 | 15 | Nucleoside diphosphate kinase | | |
| COG0106 | 18 | Phosphoribosylformimino-5-aminoimidazole carboxamide ribonucleotide (ProFAR) isomerase | | |
| COG0107 | 23 | Imidazoleglycerol-phosphate synthase | | |
| COG0108 | 17 | 3,4-dihydroxy-2-butanone 4-phosphate synthase | | |
| COG0109 | 30 | Polyprenyltransferase (cytochrome oxidase assembly factor) | | |
| COG0110 | 53 | Acetyltransferase (isoleucine patch superfamily) | | |
| COG0111 | 147 | Phosphoglycerate dehydrogenase and related dehydrogenases | | |
| COG0112 | 47 | Glycine/serine hydroxymethyltransferase | | |
| COG0113 | 18 | Delta-aminolevulinic acid dehydratase | | |
| COG0114 | 24 | Fumarase | | |
| COG0115 | 78 | Branched-chain amino acid aminotransferase/4-amino-4-deoxychorismate lyase | | |
| COG0116 | 17 | Predicted N6-adenine-specific DNA methylase | | |
| COG0117 | 6 | Pyrimidine deaminase | | |
| COG0118 | 27 | Glutamine amidotransferase | | |
| COG0119 | 109 | Isopropylmalate/homocitrate/citramalate synthases | | |
| COG0120 | 7 | Ribose 5-phosphate isomerase | | |
| COG0121 | 22 | Predicted glutamine amidotransferase | | |
| COG0122 | 26 | 3-methyladenine DNA glycosylase/8-oxoguanine DNA glycosylase | | |
| COG0123 | 46 | Deacetylases, including yeast histone deacetylase and acetoin utilization protein | | |
| COG0124 | 46 | Histidyl-tRNA synthetase | | |
| COG0125 | 32 | Thymidylate kinase | | |
| COG0126 | 35 | 3-phosphoglycerate kinase | | |
| COG0127 | 20 | Xanthosine triphosphate pyrophosphatase | | |
| COG0128 | 58 | 5-enolpyruvylshikimate-3-phosphate synthase | | |
| COG0129 | 75 | Dihydroxyacid dehydratase/phosphogluconate dehydratase | | |
| COG0130 | 37 | Pseudouridine synthase | | |
| COG0131 | 14 | Imidazoleglycerol-phosphate dehydratase | | |
| COG0132 | 5 | Dethiobiotin synthetase | | |
| COG0133 | 21 | Tryptophan synthase beta chain | | |
| COG0134 | 19 | Indole-3-glycerol phosphate synthase | | |
| COG0135 | 21 | Phosphoribosylanthranilate isomerase | | |
| COG0136 | 34 | Aspartate-semialdehyde dehydrogenase | | |
| COG0137 | 30 | Argininosuccinate synthase | | |
| COG0138 | 27 | AICAR transformylase/IMP cyclohydrolase PurH (only IMP cyclohydrolase domain in Aful) | | |
| COG0139 | 8 | Phosphoribosyl-AMP cyclohydrolase | | |
| COG0140 | 5 | Phosphoribosyl-ATP pyrophosphohydrolase | | |
| COG0141 | 43 | Histidinol dehydrogenase | | |
| COG0142 | 48 | Geranylgeranyl pyrophosphate synthase | | |
| COG0143 | 54 | Methionyl-tRNA synthetase | | |
| COG0144 | 55 | tRNA and rRNA cytosine-C5-methylases | | |
| COG0145 | 76 | N-methylhydantoinase A/acetone carboxylase, beta subunit | | |
| COG0146 | 86 | N-methylhydantoinase B/acetone carboxylase, alpha subunit | | |
| COG0147 | 73 | Anthranilate/para-aminobenzoate synthases component I | | |
| COG0148 | 31 | Enolase | | |
| COG0149 | 17 | Triosephosphate isomerase | | |
| COG0150 | 31 | Phosphoribosylaminoimidazole (AIR) synthetase | | |
| COG0151 | 30 | Phosphoribosylamine-glycine ligase | | |
| COG0152 | 27 | Phosphoribosylaminoimidazolesuccinocarboxamide (SAICAR) synthase | | |
| COG0153 | 11 | Galactokinase | | |
| COG0154 | 178 | Asp-tRNAAsn/Glu-tRNAGln amidotransferase A subunit and related amidases | | |
| COG0155 | 37 | Sulfite reductase, beta subunit (hemoprotein) | | |
| COG0156 | 67 | 7-keto-8-aminopelargonate synthetase and related enzymes | | |
| COG0157 | 30 | Nicotinate-nucleotide pyrophosphorylase | | |
| COG0158 | 11 | Fructose-1,6-bisphosphatase | | |
| COG0159 | 29 | Tryptophan synthase alpha chain | | |
| COG0160 | 73 | 4-aminobutyrate aminotransferase and related aminotransferases | | |
| COG0161 | 77 | Adenosylmethionine-8-amino-7-oxononanoate aminotransferase | | |
| COG0162 | 50 | Tyrosyl-tRNA synthetase | | |
| COG0163 | 18 | 3-polyprenyl-4-hydroxybenzoate decarboxylase | | |
| COG0164 | 26 | Ribonuclease HII | | |
| COG0165 | 42 | Argininosuccinate lyase | | |
| COG0166 | 51 | Glucose-6-phosphate isomerase | | |
| COG0167 | 59 | Dihydroorotate dehydrogenase | | |
| COG0168 | 9 | Trk-type K+ transport systems, membrane components | | |

| | | | | |
|---|---|---|---|---|
| COG0169 | 37 | Shikimate 5-dehydrogenase | | |
| COG0170 | 2 | Dolichol kinase | | |
| COG0171 | 37 | NAD synthase | | |
| COG0172 | 56 | Seryl-tRNA synthetase | | |
| COG0173 | 53 | Aspartyl-tRNA synthetase | | |
| COG0174 | 104 | Glutamine synthetase | | |
| COG0175 | 26 | 3'-phosphoadenosine 5'-phosphosulfate sulfotransferase (PAPS reductase)/FAD synthetase and related enzymes | | |
| COG0176 | 40 | Transaldolase | | |
| COG0177 | 24 | Predicted EndoIII-related endonuclease | | |
| COG0178 | 109 | Excinuclease ATPase subunit | | |
| COG0179 | 80 | 2-keto-4-pentenoate hydratase/2-oxohepta-3-ene-1,7-dioic acid hydratase (catechol pathway) | | |
| COG0180 | 46 | Tryptophanyl-tRNA synthetase | | |
| COG0181 | 27 | Porphobilinogen deaminase | | |
| COG0182 | 23 | Predicted translation initiation factor 2B subunit, eIF-2B alpha/beta/delta family | | |
| COG0183 | 228 | Acetyl-CoA acetyltransferase | | |
| COG0184 | 7 | Ribosomal protein S15P/S13E | | |
| COG0185 | 4 | Ribosomal protein S19 | | |
| COG0186 | 11 | Ribosomal protein S17 | | |
| COG0187 | 52 | Type IIA topoisomerase (DNA gyrase/topo II, topoisomerase IV), B subunit | | |
| COG0188 | 84 | Type IIA topoisomerase (DNA gyrase/topo II, topoisomerase IV), A subunit | | |
| COG0189 | 30 | Glutathione synthase/Ribosomal protein S6 modification enzyme (glutaminyl transferase) | | |
| COG0190 | 31 | 5,10-methylene-tetrahydrofolate dehydrogenase/Methenyl tetrahydrofolate cyclohydrolase | | |
| COG0191 | 8 | Fructose/tagatose bisphosphate aldolase | | |
| COG0192 | 19 | S-adenosylmethionine synthetase | | |
| COG0193 | 24 | Peptidyl-tRNA hydrolase | | |
| COG0194 | 19 | Guanylate kinase | | |
| COG0195 | 28 | Transcription elongation factor | | |
| COG0196 | 28 | FAD synthase | | |
| COG0197 | 9 | Ribosomal protein L16/L10E | | |
| COG0198 | 16 | Ribosomal protein L24 | | |
| COG0199 | 3 | Ribosomal protein S14 | | |
| COG0200 | 13 | Ribosomal protein L15 | | |
| COG0201 | 30 | Preprotein translocase subunit SecY | | |
| COG0202 | 30 | DNA-directed RNA polymerase, alpha subunit/40 kD subunit | | |
| COG0203 | 19 | Ribosomal protein L17 | | |
| COG0204 | 126 | 1-acyl-sn-glycerol-3-phosphate acyltransferase | | |
| COG0205 | 53 | 6-phosphofructokinase | | |
| COG0206 | 37 | Cell division GTPase | | |
| COG0207 | 9 | Thymidylate synthase | | |
| COG0208 | 14 | Ribonucleotide reductase, beta subunit | | |
| COG0209 | 81 | Ribonucleotide reductase, alpha subunit | | |
| COG0210 | 123 | Superfamily I DNA and RNA helicases | | |
| COG0211 | 12 | Ribosomal protein L27 | | |
| COG0212 | 11 | 5-formyltetrahydrofolate cyclo-ligase | | |
| COG0213 | 22 | Thymidine phosphorylase | | |
| COG0214 | 8 | Pyridoxine biosynthesis enzyme | | |
| COG0215 | 43 | Cysteinyl-tRNA synthetase | | |
| COG0216 | 21 | Protein chain release factor A | | |
| COG0217 | 21 | Uncharacterized conserved protein | | |
| COG0218 | 18 | Predicted GTPase | | |
| COG0219 | 14 | Predicted rRNA methylase (SpoU class) | | |
| COG0220 | 7 | Predicted S-adenosylmethionine-dependent methyltransferase | | |
| COG0221 | 20 | Inorganic pyrophosphatase | | |
| COG0222 | 5 | Ribosomal protein L7/L12 | | |
| COG0223 | 50 | Methionyl-tRNA formyltransferase | | |
| COG0224 | 35 | F0F1-type ATP synthase, gamma subunit | | |
| COG0225 | 33 | Peptide methionine sulfoxide reductase | | |
| COG0226 | 59 | ABC-type phosphate transport system, periplasmic component | | |
| COG0227 | 6 | Ribosomal protein L28 | | |
| COG0228 | 12 | Ribosomal protein S16 | | |
| COG0229 | 15 | Conserved domain frequently associated with peptide methionine sulfoxide reductase | | |
| COG0230 | 1 | Ribosomal protein L34 | | |
| COG0231 | 30 | Translation elongation factor P (EF-P)/translation initiation factor 5A (eIF-5A) | | |
| COG0232 | 23 | dGTP triphosphohydrolase | | |
| COG0233 | 25 | Ribosome recycling factor | | |
| COG0234 | 9 | Co-chaperonin GroES (HSP10) | | |
| COG0235 | 58 | Ribulose-5-phosphate 4-epimerase and related epimerases and aldolases | | |
| COG0236 | 19 | Acyl carrier protein | | |
| COG0237 | 19 | Dephospho-CoA kinase | | |
| COG0238 | 10 | Ribosomal protein S18 | | |
| COG0239 | 16 | Integral membrane protein possibly involved in chromosome condensation | | |
| COG0240 | 37 | Glycerol-3-phosphate dehydrogenase | | |
| COG0241 | 12 | Histidinol phosphatase and related phosphatases | | |
| COG0242 | 35 | N-formylmethionyl-tRNA deformylase | | |
| COG0243 | 306 | Anaerobic dehydrogenases, typically selenocysteine-containing | | |
| COG0244 | 19 | Ribosomal protein L10 | | |
| COG0245 | 18 | 2C-methyl-D-erythritol 2,4-cyclodiphosphate synthase | | |
| COG0246 | 9 | Mannitol-1-phosphate/altronate dehydrogenases | | |
| COG0247 | 153 | Fe-S oxidoreductase | | |
| COG0248 | 56 | Exopolyphosphatase | | |
| COG0249 | 78 | Mismatch repair ATPase (MutS family) | | |
| COG0250 | 16 | Transcription antiterminator | | |
| COG0251 | 94 | Putative translation initiation inhibitor, yjgF family | | |
| COG0252 | 12 | L-asparaginase/archaeal Glu-tRNAGln amidotransferase subunit D | | |

| | | | | |
|---|---|---|---|---|
| COG0253 | 26 | Diaminopimelate epimerase | | |
| COG0254 | 6 | Ribosomal protein L31 | | |
| COG0255 | 7 | Ribosomal protein L29 | | |
| COG0256 | 11 | Ribosomal protein L18 | | |
| COG0257 | 1 | Ribosomal protein L36 | | |
| COG0258 | 43 | 5'-3' exonuclease (including N-terminal domain of PolI) | | |
| COG0259 | 18 | Pyridoxamine-phosphate oxidase | | |
| COG0260 | 52 | Leucyl aminopeptidase | | |
| COG0261 | 15 | Ribosomal protein L21 | | |
| COG0262 | 77 | Dihydrofolate reductase | | |
| COG0263 | 22 | Glutamate 5-kinase | | |
| COG0264 | 31 | Translation elongation factor Ts | | |
| COG0265 | 171 | Trypsin-like serine proteases, typically periplasmic, contain C-terminal PDZ domain | | |
| COG0266 | 64 | Formamidopyrimidine-DNA glycosylase | | |
| COG0267 | 5 | Ribosomal protein L33 | | |
| COG0268 | 10 | Ribosomal protein S20 | | |
| COG0269 | 1 | 3-hexulose-6-phosphate synthase and related proteins | | |
| COG0270 | 14 | Site-specific DNA methylase | | |
| COG0271 | 4 | Stress-induced morphogen (activity unknown) | | |
| COG0272 | 63 | NAD-dependent DNA ligase (contains BRCT domain type II) | | |
| COG0274 | 13 | Deoxyribose-phosphate aldolase | | |
| COG0275 | 31 | Predicted S-adenosylmethionine-dependent methyltransferase involved in cell envelope biogenesis | | |
| COG0276 | 32 | Protoheme ferro-lyase (ferrochelatase) | | |
| COG0277 | 253 | FAD/FMN-containing dehydrogenases | | |
| COG0278 | 3 | Glutaredoxin-related protein | | |
| COG0279 | 11 | Phosphoheptose isomerase | | |
| COG0280 | 44 | Phosphotransacetylase | | |
| COG0281 | 47 | Malic enzyme | | |
| COG0282 | 26 | Acetate kinase | | |
| COG0283 | 18 | Cytidylate kinase | | |
| COG0284 | 27 | Orotidine-5'-phosphate decarboxylase | | |
| COG0285 | 47 | Folylpolyglutamate synthase | | |
| COG0286 | 44 | Type I restriction-modification system methyltransferase subunit | | |
| COG0287 | 21 | Prephenate dehydrogenase | | |
| COG0288 | 24 | Carbonic anhydrase | | |
| COG0289 | 21 | Dihydrodipicolinate reductase | | |
| COG0290 | 24 | Translation initiation factor 3 (IF-3) | | |
| COG0291 | 3 | Ribosomal protein L35 | | |
| COG0292 | 5 | Ribosomal protein L20 | | |
| COG0293 | 13 | 23S rRNA methylase | | |
| COG0294 | 34 | Dihydropteroate synthase and related enzymes | | |
| COG0295 | 8 | Cytidine deaminase | | |
| COG0296 | 59 | 1,4-alpha-glucan branching enzyme | | |
| COG0297 | 22 | Glycogen synthase | | |
| COG0298 | 11 | Hydrogenase maturation factor | | |
| COG0299 | 27 | Folate-dependent phosphoribosylglycinamide formyltransferase PurN | | |
| COG0300 | 25 | Short-chain dehydrogenases of various substrate specificities | | |
| COG0301 | 4 | Thiamine biosynthesis ATP pyrophosphatase | | |
| COG0302 | 25 | GTP cyclohydrolase I | | |
| COG0303 | 61 | Molybdopterin biosynthesis enzyme | | |
| COG0304 | 88 | 3-oxoacyl-(acyl-carrier-protein) synthase | | |
| COG0305 | 59 | Replicative DNA helicase | | |
| COG0306 | 31 | Phosphate/sulphate permeases | | |
| COG0307 | 29 | Riboflavin synthase alpha chain | | |
| COG0308 | 100 | Aminopeptidase N | | |
| COG0309 | 20 | Hydrogenase maturation factor | | |
| COG0310 | 6 | ABC-type Co2+ transport system, permease component | | |
| COG0311 | 4 | Predicted glutamine amidotransferase involved in pyridoxine biosynthesis | | |
| COG0312 | 93 | Predicted Zn-dependent proteases and their inactivated homologs | | |
| COG0313 | 34 | Predicted methyltransferases | | |
| COG0314 | 14 | Molybdopterin converting factor, large subunit | | |
| COG0315 | 14 | Molybdenum cofactor biosynthesis enzyme | | |
| COG0316 | 24 | Uncharacterized conserved protein | | |
| COG0317 | 92 | Guanosine polyphosphate pyrophosphohydrolases/synthetases | | |
| COG0318 | 370 | Acyl-CoA synthetases (AMP-forming)/AMP-acid ligases II | | |
| COG0319 | 14 | Predicted metal-dependent hydrolase | | |
| COG0320 | 32 | Lipoate synthase | | |
| COG0321 | 22 | Lipoate-protein ligase B | | |
| COG0322 | 58 | Nuclease subunit of the excinuclease complex | | |
| COG0323 | 43 | DNA mismatch repair enzyme (predicted ATPase) | | |
| COG0324 | 28 | tRNA delta(2)-isopentenylpyrophosphate transferase | | |
| COG0325 | 22 | Predicted enzyme with a TIM-barrel fold | | |
| COG0326 | 11 | Molecular chaperone, HSP90 family | | |
| COG0327 | 8 | Uncharacterized conserved protein | | |
| COG0328 | 18 | Ribonuclease HI | | |
| COG0329 | 68 | Dihydrodipicolinate synthase/N-acetylneuraminate lyase | | |
| COG0330 | 81 | Membrane protease subunits, stomatin/prohibitin homologs | | |
| COG0331 | 37 | (acyl-carrier-protein) S-malonyltransferase | | |
| COG0332 | 71 | 3-oxoacyl-[acyl-carrier-protein] synthase III | | |
| COG0333 | 2 | Ribosomal protein L32 | | |
| COG0334 | 66 | Glutamate dehydrogenase/leucine dehydrogenase | | |
| COG0335 | 11 | Ribosomal protein L19 | | |
| COG0336 | 26 | tRNA-(guanine-N1)-methyltransferase | | |
| COG0337 | 29 | 3-dehydroquinate synthetase | | |

| | | | | |
|---|---|---|---|---|
| COG0338 | 1 | Site-specific DNA methylase | | |
| COG0339 | 48 | Zn-dependent oligopeptidases | | |
| COG0340 | 28 | Biotin-(acetyl-CoA carboxylase) ligase | | |
| COG0341 | 31 | Preprotein translocase subunit SecF | | |
| COG0342 | 42 | Preprotein translocase subunit SecD | | |
| COG0343 | 25 | Queuine/archaeosine tRNA-ribosyltransferase | | |
| COG0344 | 21 | Predicted membrane protein | | |
| COG0345 | 27 | Pyrroline-5-carboxylate reductase | | |
| COG0346 | 152 | Lactoylglutathione lyase and related lyases | | |
| COG0347 | 14 | Nitrogen regulatory protein PII | | |
| COG0348 | 27 | Polyferredoxin | | |
| COG0349 | 17 | Ribonuclease D | | |
| COG0350 | 29 | Methylated DNA-protein cysteine methyltransferase | | |
| COG0351 | 25 | Hydroxymethylpyrimidine/phosphomethylpyrimidine kinase | | |
| COG0352 | 20 | Thiamine monophosphate synthase | | |
| COG0353 | 17 | Recombinational DNA repair protein (RecF pathway) | | |
| COG0354 | 27 | Predicted aminomethyltransferase related to GcvT | | |
| COG0355 | 7 | F0F1-type ATP synthase, epsilon subunit (mitochondrial delta subunit) | | |
| COG0356 | 46 | F0F1-type ATP synthase, subunit a | | |
| COG0357 | 23 | Predicted S-adenosylmethionine-dependent methyltransferase involved in bacterial cell division | | |
| COG0358 | 48 | DNA primase (bacterial type) | | |
| COG0359 | 22 | Ribosomal protein L9 | | |
| COG0360 | 18 | Ribosomal protein S6 | | |
| COG0361 | 12 | Translation initiation factor 1 (IF-1) | | |
| COG0362 | 12 | 6-phosphogluconate dehydrogenase | | |
| COG0363 | 25 | 6-phosphogluconolactonase/Glucosamine-6-phosphate isomerase/deaminase | | |
| COG0364 | 39 | Glucose-6-phosphate 1-dehydrogenase | | |
| COG0365 | 191 | Acyl-coenzyme A synthetases/AMP-(fatty) acid ligases | | |
| COG0366 | 99 | Glycosidases | | |
| COG0367 | 152 | Asparagine synthase (glutamine-hydrolyzing) | | |
| COG0369 | 8 | Sulfite reductase, alpha subunit (flavoprotein) | | |
| COG0370 | 35 | Fe2+ transport system protein B | | |
| COG0371 | 3 | Glycerol dehydrogenase and related enzymes | | |
| COG0372 | 54 | Citrate synthase | | |
| COG0373 | 42 | Glutamyl-tRNA reductase | | |
| COG0374 | 37 | Ni,Fe-hydrogenase I large subunit | | |
| COG0375 | 3 | Zn finger protein HypA/HybF (possibly regulating hydrogenase expression) | | |
| COG0376 | 17 | Catalase (peroxidase I) | | |
| COG0377 | 12 | NADH:ubiquinone oxidoreductase 20 kD subunit and related Fe-S oxidoreductases | | |
| COG0378 | 12 | Ni2+-binding GTPase involved in regulation of expression and maturation of urease and hydrogenase | | |
| COG0379 | 23 | Quinolinate synthase | | |
| COG0380 | 44 | Trehalose-6-phosphate synthase | | |
| COG0381 | 42 | UDP-N-acetylglucosamine 2-epimerase | | |
| COG0382 | 49 | 4-hydroxybenzoate polyprenyltransferase and related prenyltransferases | | |
| COG0383 | 19 | Alpha-mannosidase | | |
| COG0384 | 42 | Predicted epimerase, PhzC/PhzF homolog | | |
| COG0385 | 2 | Predicted Na+-dependent transporter | | |
| COG0386 | 7 | Glutathione peroxidase | | |
| COG0387 | 22 | Ca2+/H+ antiporter | | |
| COG0388 | 121 | Predicted amidohydrolase | | |
| COG0389 | 63 | Nucleotidyltransferase/DNA polymerase involved in DNA repair | | |
| COG0390 | 1 | ABC-type uncharacterized transport system, permease component | | |
| COG0391 | 41 | Uncharacterized conserved protein | | |
| COG0392 | 25 | Predicted integral membrane protein | | |
| COG0393 | 10 | Uncharacterized conserved protein | | |
| COG0394 | 34 | Protein-tyrosine-phosphatase | | |
| COG0395 | 139 | ABC-type sugar transport system, permease component | | |
| COG0396 | 14 | ABC-type transport system involved in Fe-S cluster assembly, ATPase component | | |
| COG0397 | 11 | Uncharacterized conserved protein | | |
| COG0398 | 13 | Uncharacterized conserved protein | | |
| COG0399 | 131 | Predicted pyridoxal phosphate-dependent enzyme apparently involved in regulation of cell wall biogenesis | | |
| COG0400 | 14 | Predicted esterase | | |
| COG0402 | 95 | Cytosine deaminase and related metal-dependent hydrolases | | |
| COG0403 | 27 | Glycine cleavage system protein P (pyridoxal-binding), N-terminal domain | | |
| COG0404 | 96 | Glycine cleavage system T protein (aminomethyltransferase) | | |
| COG0405 | 136 | Gamma-glutamyltransferase | | |
| COG0406 | 50 | Fructose-2,6-bisphosphatase | | |
| COG0407 | 38 | Uroporphyrinogen-III decarboxylase | | |
| COG0408 | 7 | Coproporphyrinogen III oxidase | | |
| COG0409 | 11 | Hydrogenase maturation factor | | |
| COG0410 | 110 | ABC-type branched-chain amino acid transport systems, ATPase component | | |
| COG0411 | 124 | ABC-type branched-chain amino acid transport systems, ATPase component | | |
| COG0412 | 71 | Dienelactone hydrolase and related enzymes | | |
| COG0413 | 26 | Ketopantoate hydroxymethyltransferase | | |
| COG0414 | 21 | Panthothenate synthetase | | |
| COG0415 | 28 | Deoxyribodipyrimidine photolyase | | |
| COG0416 | 30 | Fatty acid/phospholipid biosynthesis enzyme | | |
| COG0417 | 16 | DNA polymerase elongation subunit (family B) | | |
| COG0418 | 9 | Dihydroorotase | | |
| COG0419 | 14 | ATPase involved in DNA repair | | |
| COG0420 | 5 | DNA repair exonuclease | | |
| COG0421 | 30 | Spermidine synthase | | |
| COG0422 | 10 | Thiamine biosynthesis protein ThiC | | |
| COG0423 | 14 | Glycyl-tRNA synthetase (class II) | | |

| | | | | |
|---|---|---|---|---|
| COG0424 | 26 | Nucleotide-binding protein implicated in inhibition of septum formation | | |
| COG0425 | 12 | Predicted redox protein, regulator of disulfide bond formation | | |
| COG0426 | 2 | Uncharacterized flavoproteins | | |
| COG0427 | 15 | Acetyl-CoA hydrolase | | |
| COG0428 | 8 | Predicted divalent heavy-metal cations transporter | | |
| COG0429 | 16 | Predicted hydrolase of the alpha/beta-hydrolase fold | | |
| COG0430 | 6 | RNA 3'-terminal phosphate cyclase | | |
| COG0431 | 31 | Predicted flavoprotein | | |
| COG0432 | 26 | Uncharacterized conserved protein | | |
| COG0433 | 45 | Predicted ATPase | | |
| COG0434 | 6 | Predicted TIM-barrel enzyme | | |
| COG0435 | 11 | Predicted glutathione S-transferase | | |
| COG0436 | 188 | Aspartate/tyrosine/aromatic aminotransferase | | |
| COG0437 | 73 | Fe-S-cluster-containing hydrogenase components 1 | | |
| COG0438 | 517 | Glycosyltransferase | | |
| COG0439 | 49 | Biotin carboxylase | | |
| COG0440 | 12 | Acetolactate synthase, small (regulatory) subunit | | |
| COG0441 | 66 | Threonyl-tRNA synthetase | | |
| COG0442 | 44 | Prolyl-tRNA synthetase | | |
| COG0443 | 81 | Molecular chaperone | | |
| COG0444 | 36 | ABC-type dipeptide/oligopeptide/nickel transport system, ATPase component | | |
| COG0445 | 38 | NAD/FAD-utilizing enzyme apparently involved in cell division | | |
| COG0446 | 106 | Uncharacterized NAD(FAD)-dependent dehydrogenases | | |
| COG0447 | 12 | Dihydroxynaphthoic acid synthase | | |
| COG0448 | 42 | ADP-glucose pyrophosphorylase | | |
| COG0449 | 54 | Glucosamine 6-phosphate synthetase, contains amidotransferase and phosphosugar isomerase domains | | |
| COG0450 | 15 | Peroxiredoxin | | |
| COG0451 | 352 | Nucleoside-diphosphate-sugar epimerases | | |
| COG0452 | 38 | Phosphopantothenoylcysteine synthetase/decarboxylase | | |
| COG0454 | 165 | Histone acetyltransferase HPA2 and related acetyltransferases | | |
| COG0455 | 9 | ATPases involved in chromosome partitioning | | |
| COG0456 | 37 | Acetyltransferases | | |
| COG0457 | 393 | FOG: TPR repeat | | |
| COG0458 | 60 | Carbamoylphosphate synthase large subunit (split gene in MJ) | | |
| COG0459 | 39 | Chaperonin GroEL (HSP60 family) | | |
| COG0460 | 37 | Homoserine dehydrogenase | | |
| COG0461 | 21 | Orotate phosphoribosyltransferase | | |
| COG0462 | 50 | Phosphoribosylpyrophosphate synthetase | | |
| COG0463 | 336 | Glycosyltransferases involved in cell wall biogenesis | | |
| COG0464 | 59 | ATPases of the AAA+ class | | |
| COG0465 | 71 | ATP-dependent Zn proteases | | |
| COG0466 | 92 | ATP-dependent Lon protease, bacterial type | | |
| COG0467 | 37 | RecA-superfamily ATPases implicated in signal transduction | | |
| COG0468 | 31 | RecA/RadA recombinase | | |
| COG0469 | 41 | Pyruvate kinase | | |
| COG0470 | 20 | ATPase involved in DNA replication | | |
| COG0471 | 24 | Di- and tricarboxylate transporters | | |
| COG0472 | 57 | UDP-N-acetylmuramyl pentapeptide phosphotransferase/UDP-N-acetylglucosamine-1-phosphate transferase | | |
| COG0473 | 58 | Isocitrate/isopropylmalate dehydrogenase | | |
| COG0474 | 159 | Cation transport ATPase | | |
| COG0475 | 59 | Kef-type K+ transport systems, membrane components | | |
| COG0476 | 39 | Dinucleotide-utilizing enzymes involved in molybdopterin and thiamine biosynthesis family 2 | | |
| COG0477 | 941 | Permeases of the major facilitator superfamily | | |
| COG0479 | 35 | Succinate dehydrogenase/fumarate reductase, Fe-S protein subunit | | |
| COG0480 | 130 | Translation elongation factors (GTPases) | | |
| COG0481 | 45 | Membrane GTPase LepA | | |
| COG0482 | 46 | Predicted tRNA(5-methylaminomethyl-2-thiouridylate) methyltransferase, contains the PP-loop ATPase domain | | |
| COG0483 | 52 | Archaeal fructose-1,6-bisphosphatase and related enzymes of inositol monophosphatase family | | |
| COG0484 | 50 | DnaJ-class molecular chaperone with C-terminal Zn finger domain | | |
| COG0486 | 35 | Predicted GTPase | | |
| COG0488 | 66 | ATPase components of ABC transporters with duplicated ATPase domains | | |
| COG0489 | 76 | ATPases involved in chromosome partitioning | | |
| COG0490 | 2 | Putative regulatory, ligand-binding protein related to C-terminal domains of K+ channels | | |
| COG0491 | 230 | Zn-dependent hydrolases, including glyoxylases | | |
| COG0492 | 96 | Thioredoxin reductase | | |
| COG0493 | 110 | NADPH-dependent glutamate synthase beta chain and related oxidoreductases | | |
| COG0494 | 80 | NTP pyrophosphohydrolases including oxidative damage repair enzymes | | |
| COG0495 | 73 | Leucyl-tRNA synthetase | | |
| COG0496 | 25 | Predicted acid phosphatase | | |
| COG0497 | 48 | ATPase involved in DNA repair | | |
| COG0498 | 65 | Threonine synthase | | |
| COG0499 | 28 | S-adenosylhomocysteine hydrolase | | |
| COG0500 | 502 | SAM-dependent methyltransferases | | |
| COG0501 | 88 | Zn-dependent protease with chaperone function | | |
| COG0502 | 20 | Biotin synthase and related enzymes | | |
| COG0503 | 10 | Adenine/guanine phosphoribosyltransferases and related PRPP-binding proteins | | |
| COG0504 | 32 | CTP synthase (UTP-ammonia lyase) | | |
| COG0505 | 39 | Carbamoylphosphate synthase small subunit | | |
| COG0506 | 28 | Proline dehydrogenase | | |
| COG0507 | 44 | ATP-dependent exoDNAse (exonuclease V), alpha subunit - helicase superfamily I member | | |
| COG0508 | 99 | Pyruvate/2-oxoglutarate dehydrogenase complex, dihydrolipoamide acyltransferase (E2) component, and related enzymes | | |
| COG0509 | 28 | Glycine cleavage system H protein (lipoate-binding) | | |
| COG0510 | 6 | Predicted choline kinase involved in LPS biosynthesis | | |
| COG0511 | 11 | Biotin carboxyl carrier protein | | |

| | | | | |
|---|---|---|---|---|
| COG0512 | 16 | Anthranilate/para-aminobenzoate synthases component II | | |
| COG0513 | 60 | Superfamily II DNA and RNA helicases | | |
| COG0514 | 40 | Superfamily II DNA helicase | | |
| COG0515 | 365 | Serine/threonine protein kinase | | |
| COG0516 | 52 | IMP dehydrogenase/GMP reductase | | |
| COG0517 | 103 | FOG: CBS domain | | |
| COG0518 | 25 | GMP synthase - Glutamine amidotransferase domain | | |
| COG0519 | 21 | GMP synthase, PP-ATPase domain/subunit | | |
| COG0520 | 111 | Selenocysteine lyase | | |
| COG0521 | 18 | Molybdopterin biosynthesis enzymes | | |
| COG0522 | 18 | Ribosomal protein S4 and related proteins | | |
| COG0523 | 19 | Putative GTPases (G3E family) | | |
| COG0524 | 73 | Sugar kinases, ribokinase family | | |
| COG0525 | 84 | Valyl-tRNA synthetase | | |
| COG0526 | 147 | Thiol-disulfide isomerase and thioredoxins | | |
| COG0527 | 35 | Aspartokinases | | |
| COG0528 | 18 | Uridylate kinase | | |
| COG0529 | 15 | Adenylylsulfate kinase and related kinases | | |
| COG0530 | 18 | Ca2+/Na+ antiporter | | |
| COG0531 | 252 | Amino acid transporters | | |
| COG0532 | 40 | Translation initiation factor 2 (IF-2; GTPase) | | |
| COG0533 | 28 | Metal-dependent proteases with possible chaperone activity | | |
| COG0534 | 48 | Na+-driven multidrug efflux pump | | |
| COG0535 | 97 | Predicted Fe-S oxidoreductases | | |
| COG0536 | 33 | Predicted GTPase | | |
| COG0537 | 49 | Diadenosine tetraphosphate (Ap4A) hydrolase and other HIT family hydrolases | | |
| COG0538 | 19 | Isocitrate dehydrogenases | | |
| COG0539 | 43 | Ribosomal protein S1 | | |
| COG0540 | 35 | Aspartate carbamoyltransferase, catalytic chain | | |
| COG0541 | 40 | Signal recognition particle GTPase | | |
| COG0542 | 110 | ATPases with chaperone activity, ATP-binding subunit | | |
| COG0543 | 57 | 2-polyprenylphenol hydroxylase and related flavodoxin oxidoreductases | | |
| COG0544 | 51 | FKBP-type peptidyl-prolyl cis-trans isomerase (trigger factor) | | |
| COG0545 | 11 | FKBP-type peptidyl-prolyl cis-trans isomerases 1 | | |
| COG0546 | 41 | Predicted phosphatases | | |
| COG0547 | 29 | Anthranilate phosphoribosyltransferase | | |
| COG0548 | 28 | Acetylglutamate kinase | | |
| COG0549 | 16 | Carbamate kinase | | |
| COG0550 | 61 | Topoisomerase IA | | |
| COG0551 | 15 | Zn-finger domain associated with topoisomerase type I | | |
| COG0552 | 18 | Signal recognition particle GTPase | | |
| COG0553 | 56 | Superfamily II DNA/RNA helicases, SNF2 family | | |
| COG0554 | 40 | Glycerol kinase | | |
| COG0555 | 17 | ABC-type sulfate transport system, permease component | | |
| COG0556 | 41 | Helicase subunit of the DNA excision repair complex | | |
| COG0557 | 64 | Exoribonuclease R | | |
| COG0558 | 54 | Phosphatidylglycerophosphate synthase | | |
| COG0559 | 153 | Branched-chain amino acid ABC-type transport system, permease components | | |
| COG0560 | 28 | Phosphoserine phosphatase | | |
| COG0561 | 33 | Predicted hydrolases of the HAD superfamily | | |
| COG0562 | 11 | UDP-galactopyranose mutase | | |
| COG0563 | 35 | Adenylate kinase and related kinases | | |
| COG0564 | 55 | Pseudouridylate synthases, 23S RNA-specific | | |
| COG0565 | 19 | rRNA methylase | | |
| COG0566 | 55 | rRNA methylases | | |
| COG0567 | 38 | 2-oxoglutarate dehydrogenase complex, dehydrogenase (E1) component, and related enzymes | | |
| COG0568 | 82 | DNA-directed RNA polymerase, sigma subunit (sigma70/sigma32) | | |
| COG0569 | 23 | K+ transport systems, NAD-binding component | | |
| COG0571 | 26 | dsRNA-specific ribonuclease | | |
| COG0572 | 18 | Uridine kinase | | |
| COG0573 | 38 | ABC-type phosphate transport system, permease component | | |
| COG0574 | 126 | Phosphoenolpyruvate synthase/pyruvate phosphate dikinase | | |
| COG0575 | 22 | CDP-diglyceride synthetase | | |
| COG0576 | 21 | Molecular chaperone GrpE (heat shock protein) | | |
| COG0577 | 187 | ABC-type antimicrobial peptide transport system, permease component | | |
| COG0578 | 47 | Glycerol-3-phosphate dehydrogenase | | |
| COG0579 | 30 | Predicted dehydrogenase | | |
| COG0580 | 30 | Glycerol uptake facilitator and related permeases (Major Intrinsic Protein Family) | | |
| COG0581 | 38 | ABC-type phosphate transport system, permease component | | |
| COG0582 | 118 | Integrase | | |
| COG0583 | 248 | Transcriptional regulator | | |
| COG0584 | 36 | Glycerophosphoryl diester phosphodiesterase | | |
| COG0585 | 3 | Uncharacterized conserved protein | | |
| COG0586 | 59 | Uncharacterized membrane-associated protein | | |
| COG0587 | 186 | DNA polymerase III, alpha subunit | | |
| COG0588 | 7 | Phosphoglycerate mutase 1 | | |
| COG0589 | 79 | Universal stress protein UspA and related nucleotide-binding proteins | | |
| COG0590 | 25 | Cytosine/adenosine deaminases | | |
| COG0591 | 61 | Na+/proline symporter | | |
| COG0592 | 48 | DNA polymerase sliding clamp subunit (PCNA homolog) | | |
| COG0593 | 48 | ATPase involved in DNA replication initiation | | |
| COG0594 | 18 | RNase P protein component | | |
| COG0595 | 45 | Predicted hydrolase of the metallo-beta-lactamase superfamily | | |
| COG0596 | 521 | Predicted hydrolases or acyltransferases (alpha/beta hydrolase superfamily) | | |

| | | | | |
|---|---|---|---|---|
| COG0597 | 15 | Lipoprotein signal peptidase | | |
| COG0598 | 50 | Mg2+ and Co2+ transporters | | |
| COG0599 | 27 | Uncharacterized homolog of gamma-carboxymuconolactone decarboxylase subunit | | |
| COG0600 | 80 | ABC-type nitrate/sulfonate/bicarbonate transport system, permease component | | |
| COG0601 | 184 | ABC-type dipeptide/oligopeptide/nickel transport systems, permease components | | |
| COG0602 | 15 | Organic radical activating enzymes | | |
| COG0603 | 21 | Predicted PP-loop superfamily ATPase | | |
| COG0604 | 194 | NADPH:quinone reductase and related Zn-dependent oxidoreductases | | |
| COG0605 | 21 | Superoxide dismutase | | |
| COG0606 | 60 | Predicted ATPase with chaperone activity | | |
| COG0607 | 78 | Rhodanese-related sulfurtransferase | | |
| COG0608 | 41 | Single-stranded DNA-specific exonuclease | | |
| COG0609 | 13 | ABC-type Fe3+-siderophore transport system, permease component | | |
| COG0610 | 35 | Type I site-specific restriction-modification system, R (restriction) subunit and related helicases | | |
| COG0611 | 25 | Thiamine monophosphate kinase | | |
| COG0612 | 209 | Predicted Zn-dependent peptidases | | |
| COG0613 | 28 | Predicted metal-dependent phosphoesterases (PHP family) | | |
| COG0614 | 35 | ABC-type Fe3+-hydroxamate transport system, periplasmic component | | |
| COG0616 | 42 | Periplasmic serine proteases (ClpP class) | | |
| COG0617 | 51 | tRNA nucleotidyltransferase/poly(A) polymerase | | |
| COG0618 | 31 | Exopolyphosphatase-related proteins | | |
| COG0619 | 13 | ABC-type cobalt transport system, permease component CbiQ and related transporters | | |
| COG0620 | 72 | Methionine synthase II (cobalamin-independent) | | |
| COG0621 | 77 | 2-methylthioadenine synthetase | | |
| COG0622 | 18 | Predicted phosphoesterase | | |
| COG0623 | 35 | Enoyl-[acyl-carrier-protein] reductase (NADH) | | |
| COG0624 | 226 | Acetylornithine deacetylase/Succinyl-diaminopimelate desuccinylase and related deacylases | | |
| COG0625 | 133 | Glutathione S-transferase | | |
| COG0626 | 84 | Cystathionine beta-lyases/cystathionine gamma-synthases | | |
| COG0627 | 17 | Predicted esterase | | |
| COG0628 | 65 | Predicted permease | | |
| COG0629 | 24 | Single-stranded DNA-binding protein | | |
| COG0630 | 2 | Type IV secretory pathway, VirB11 components, and related ATPases involved in archaeal flagella biosynthesis | | |
| COG0631 | 43 | Serine/threonine protein phosphatase | | |
| COG0632 | 21 | Holliday junction resolvasome, DNA-binding subunit | | |
| COG0633 | 13 | Ferredoxin | | |
| COG0634 | 26 | Hypoxanthine-guanine phosphoribosyltransferase | | |
| COG0635 | 52 | Coproporphyrinogen III oxidase and related Fe-S oxidoreductases | | |
| COG0636 | 8 | F0F1-type ATP synthase, subunit c/Archaeal/vacuolar-type H+-ATPase, subunit K | | |
| COG0637 | 26 | Predicted phosphatase/phosphohexomutase | | |
| COG0638 | 11 | 20S proteasome, alpha and beta subunits | | |
| COG0639 | 59 | Diadenosine tetraphosphatase and related serine/threonine protein phosphatases | | |
| COG0640 | 86 | Predicted transcriptional regulators | | |
| COG0641 | 10 | Arylsulfatase regulator (Fe-S oxidoreductase) | | |
| COG0642 | 750 | Signal transduction histidine kinase | | |
| COG0643 | 80 | Chemotaxis protein histidine kinase and related kinases | | |
| COG0644 | 62 | Dehydrogenases (flavoproteins) | | |
| COG0645 | 3 | Predicted kinase | | |
| COG0646 | 41 | Methionine synthase I (cobalamin-dependent), methyltransferase domain | | |
| COG0647 | 19 | Predicted sugar phosphatases of the HAD superfamily | | |
| COG0648 | 34 | Endonuclease IV | | |
| COG0649 | 70 | NADH:ubiquinone oxidoreductase 49 kD subunit 7 | | |
| COG0650 | 13 | Formate hydrogenlyase subunit 4 | | |
| COG0651 | 54 | Formate hydrogenlyase subunit 3/Multisubunit Na+/H+ antiporter, MnhD subunit | | |
| COG0652 | 34 | Peptidyl-prolyl cis-trans isomerase (rotamase) - cyclophilin family | | |
| COG0653 | 73 | Preprotein translocase subunit SecA (ATPase, RNA helicase) | | |
| COG0654 | 127 | 2-polyprenyl-6-methoxyphenol hydroxylase and related FAD-dependent oxidoreductases | | |
| COG0655 | 28 | Multimeric flavodoxin WrbA | | |
| COG0656 | 39 | Aldo/keto reductases, related to diketogulonate reductase | | |
| COG0657 | 49 | Esterase/lipase | | |
| COG0658 | 23 | Predicted membrane metal-binding protein | | |
| COG0659 | 53 | Sulfate permease and related transporters (MFS superfamily) | | |
| COG0661 | 57 | Predicted unusual protein kinase | | |
| COG0662 | 20 | Mannose-6-phosphate isomerase | | |
| COG0663 | 31 | Carbonic anhydrases/acetyltransferases, isoleucine patch superfamily | | |
| COG0664 | 150 | cAMP-binding proteins - catabolite gene activator and regulatory subunit of cAMP-dependent protein kinases | | |
| COG0665 | 190 | Glycine/D-amino acid oxidases (deaminating) | | |
| COG0666 | 38 | FOG: Ankyrin repeat | | |
| COG0667 | 157 | Predicted oxidoreductases (related to aryl-alcohol dehydrogenases) | | |
| COG0668 | 45 | Small-conductance mechanosensitive channel | | |
| COG0669 | 22 | Phosphopantetheine adenylyltransferase | | |
| COG0670 | 10 | Integral membrane protein, interacts with FtsH | | |
| COG0671 | 14 | Membrane-associated phospholipid phosphatase | | |
| COG0672 | 13 | High-affinity Fe2+/Pb2+ permease | | |
| COG0673 | 224 | Predicted dehydrogenases and related proteins | | |
| COG0674 | 108 | Pyruvate:ferredoxin oxidoreductase and related 2-oxoacid:ferredoxin oxidoreductases, alpha subunit | | |
| COG0675 | 10 | Transposase and inactivated derivatives | | |
| COG0676 | 1 | Uncharacterized enzymes related to aldose 1-epimerase | | |
| COG0677 | 49 | UDP-N-acetyl-D-mannosaminuronate dehydrogenase | | |
| COG0678 | 3 | Peroxiredoxin | | |
| COG0679 | 11 | Predicted permeases | | |
| COG0680 | 15 | Ni,Fe-hydrogenase maturation factor | | |
| COG0681 | 47 | Signal peptidase I | | |
| COG0682 | 26 | Prolipoprotein diacylglyceryltransferase | | |

| | | | | |
|---|---|---|---|---|
| COG0683 | 253 | ABC-type branched-chain amino acid transport systems, periplasmic component | | |
| COG0684 | 27 | Demethylmenaquinone methyltransferase | | |
| COG0685 | 56 | 5,10-methylenetetrahydrofolate reductase | | |
| COG0686 | 27 | Alanine dehydrogenase | | |
| COG0687 | 35 | Spermidine/putrescine-binding periplasmic protein | | |
| COG0688 | 14 | Phosphatidylserine decarboxylase | | |
| COG0689 | 19 | RNase PH | | |
| COG0690 | 2 | Preprotein translocase subunit SecE | | |
| COG0691 | 26 | tmRNA-binding protein | | |
| COG0692 | 6 | Uracil DNA glycosylase | | |
| COG0693 | 39 | Putative intracellular protease/amidase | | |
| COG0694 | 6 | Thioredoxin-like proteins and domains | | |
| COG0695 | 13 | Glutaredoxin and related proteins | | |
| COG0696 | 22 | Phosphoglyceromutase | | |
| COG0697 | 200 | Permeases of the drug/metabolite transporter (DMT) superfamily | | |
| COG0698 | 25 | Ribose 5-phosphate isomerase RpiB | | |
| COG0699 | 12 | Predicted GTPases (dynamin-related) | | |
| COG0700 | 4 | Uncharacterized membrane protein | | |
| COG0701 | 18 | Predicted permeases | | |
| COG0702 | 72 | Predicted nucleoside-diphosphate-sugar epimerases | | |
| COG0703 | 22 | Shikimate kinase | | |
| COG0704 | 22 | Phosphate uptake regulator | | |
| COG0705 | 46 | Uncharacterized membrane protein (homolog of Drosophila rhomboid) | | |
| COG0706 | 54 | Preprotein translocase subunit YidC | | |
| COG0707 | 37 | UDP-N-acetylglucosamine:LPS N-acetylglucosamine transferase | | |
| COG0708 | 29 | Exonuclease III | | |
| COG0709 | 27 | Selenophosphate synthase | | |
| COG0710 | 3 | 3-dehydroquinate dehydratase | | |
| COG0711 | 9 | F0F1-type ATP synthase, subunit b | | |
| COG0712 | 19 | F0F1-type ATP synthase, delta subunit (mitochondrial oligomycin sensitivity protein) | | |
| COG0713 | 10 | NADH:ubiquinone oxidoreductase subunit 11 or 4L (chain K) | | |
| COG0714 | 92 | MoxR-like ATPases | | |
| COG0715 | 105 | ABC-type nitrate/sulfonate/bicarbonate transport systems, periplasmic components | | |
| COG0716 | 3 | Flavodoxins | | |
| COG0717 | 20 | Deoxycytidine deaminase | | |
| COG0718 | 10 | Uncharacterized protein conserved in bacteria | | |
| COG0719 | 48 | ABC-type transport system involved in Fe-S cluster assembly, permease component | | |
| COG0720 | 21 | 6-pyruvoyl-tetrahydropterin synthase | | |
| COG0721 | 7 | Asp-tRNAAsn/Glu-tRNAGln amidotransferase C subunit | | |
| COG0722 | 11 | 3-deoxy-D-arabino-heptulosonate 7-phosphate (DAHP) synthase | | |
| COG0723 | 31 | Rieske Fe-S protein | | |
| COG0724 | 10 | RNA-binding proteins (RRM domain) | | |
| COG0725 | 58 | ABC-type molybdate transport system, periplasmic component | | |
| COG0726 | 106 | Predicted xylanase/chitin deacetylase | | |
| COG0727 | 5 | Predicted Fe-S-cluster oxidoreductase | | |
| COG0728 | 46 | Uncharacterized membrane protein, putative virulence factor | | |
| COG0729 | 23 | Outer membrane protein | | |
| COG0730 | 86 | Predicted permeases | | |
| COG0731 | 2 | Fe-S oxidoreductases | | |
| COG0732 | 13 | Restriction endonuclease S subunits | | |
| COG0733 | 5 | Na+-dependent transporters of the SNF family | | |
| COG0735 | 32 | Fe2+/Zn2+ uptake regulation proteins | | |
| COG0736 | 14 | Phosphopantetheinyl transferase (holo-ACP synthase) | | |
| COG0737 | 42 | 5'-nucleotidase/2',3'-cyclic phosphodiesterase and related esterases | | |
| COG0738 | 2 | Fucose permease | | |
| COG0739 | 116 | Membrane proteins related to metalloendopeptidases | | |
| COG0740 | 26 | Protease subunit of ATP-dependent Clp proteases | | |
| COG0741 | 103 | Soluble lytic murein transglycosylase and related regulatory proteins (some contain LysM/invasin domains) | | |
| COG0742 | 23 | N6-adenine-specific methylase | | |
| COG0743 | 33 | 1-deoxy-D-xylulose 5-phosphate reductoisomerase | | |
| COG0744 | 106 | Membrane carboxypeptidase (penicillin-binding protein) | | |
| COG0745 | 284 | Response regulators consisting of a CheY-like receiver domain and a winged-helix DNA-binding domain | | |
| COG0746 | 9 | Molybdopterin-guanine dinucleotide biosynthesis protein A | | |
| COG0747 | 349 | ABC-type dipeptide transport system, periplasmic component | | |
| COG0748 | 4 | Putative heme iron utilization protein | | |
| COG0749 | 48 | DNA polymerase I - 3'-5' exonuclease and polymerase domains | | |
| COG0750 | 48 | Predicted membrane-associated Zn-dependent proteases 1 | | |
| COG0751 | 33 | Glycyl-tRNA synthetase, beta subunit | | |
| COG0752 | 12 | Glycyl-tRNA synthetase, alpha subunit | | |
| COG0753 | 10 | Catalase | | |
| COG0754 | 9 | Glutathionylspermidine synthase | | |
| COG0755 | 47 | ABC-type transport system involved in cytochrome c biogenesis, permease component | | |
| COG0756 | 13 | dUTPase | | |
| COG0757 | 17 | 3-dehydroquinate dehydratase II | | |
| COG0758 | 32 | Predicted Rossmann fold nucleotide-binding protein involved in DNA uptake | | |
| COG0759 | 13 | Uncharacterized conserved protein | | |
| COG0760 | 86 | Parvulin-like peptidyl-prolyl isomerase | | |
| COG0761 | 29 | Penicillin tolerance protein | | |
| COG0762 | 10 | Predicted integral membrane protein | | |
| COG0763 | 27 | Lipid A disaccharide synthetase | | |
| COG0764 | 23 | 3-hydroxymyristoyl/3-hydroxydecanoyl-(acyl carrier protein) dehydratases | | |
| COG0765 | 49 | ABC-type amino acid transport system, permease component | | |
| COG0766 | 49 | UDP-N-acetylglucosamine enolpyruvyl transferase | | |
| COG0767 | 50 | ABC-type transport system involved in resistance to organic solvents, permease component | | |

| | | | | |
|---|---|---|---|---|
| COG0768 | 159 | Cell division protein FtsI/penicillin-binding protein 2 | | |
| COG0769 | 56 | UDP-N-acetylmuramyl tripeptide synthase | | |
| COG0770 | 34 | UDP-N-acetylmuramyl pentapeptide synthase | | |
| COG0771 | 31 | UDP-N-acetylmuramoylalanine-D-glutamate ligase | | |
| COG0772 | 84 | Bacterial cell division membrane protein | | |
| COG0773 | 50 | UDP-N-acetylmuramate-alanine ligase | | |
| COG0774 | 25 | UDP-3-O-acyl-N-acetylglucosamine deacetylase | | |
| COG0775 | 6 | Nucleoside phosphorylase | | |
| COG0776 | 32 | Bacterial nucleoid DNA-binding protein | | |
| COG0777 | 22 | Acetyl-CoA carboxylase beta subunit | | |
| COG0778 | 57 | Nitroreductase | | |
| COG0779 | 22 | Uncharacterized protein conserved in bacteria | | |
| COG0780 | 11 | Enzyme related to GTP cyclohydrolase I | | |
| COG0781 | 19 | Transcription termination factor | | |
| COG0782 | 35 | Transcription elongation factor | | |
| COG0783 | 10 | DNA-binding ferritin-like protein (oxidative damage protectant) | | |
| COG0784 | 286 | FOG: CheY-like receiver | | |
| COG0785 | 18 | Cytochrome c biogenesis protein | | |
| COG0786 | 1 | Na+/glutamate symporter | | |
| COG0787 | 36 | Alanine racemase | | |
| COG0788 | 6 | Formyltetrahydrofolate hydrolase | | |
| COG0789 | 54 | Predicted transcriptional regulators | | |
| COG0790 | 25 | FOG: TPR repeat, SEL1 subfamily | | |
| COG0791 | 33 | Cell wall-associated hydrolases (invasion-associated proteins) | | |
| COG0792 | 13 | Predicted endonuclease distantly related to archaeal Holliday junction resolvase | | |
| COG0793 | 84 | Periplasmic protease | | |
| COG0794 | 12 | Predicted sugar phosphate isomerase involved in capsule formation | | |
| COG0795 | 41 | Predicted permeases | | |
| COG0796 | 15 | Glutamate racemase | | |
| COG0797 | 13 | Lipoproteins | | |
| COG0798 | 22 | Arsenite efflux pump ACR3 and related permeases | | |
| COG0799 | 15 | Uncharacterized homolog of plant Iojap protein | | |
| COG0800 | 8 | 2-keto-3-deoxy-6-phosphogluconate aldolase | | |
| COG0801 | 17 | 7,8-dihydro-6-hydroxymethylpterin-pyrophosphokinase | | |
| COG0802 | 17 | Predicted ATPase or kinase | | |
| COG0803 | 33 | ABC-type metal ion transport system, periplasmic component/surface adhesin | | |
| COG0804 | 11 | Urea amidohydrolase (urease) alpha subunit | | |
| COG0805 | 33 | Sec-independent protein secretion pathway component TatC | | |
| COG0806 | 16 | RimM protein, required for 16S rRNA processing | | |
| COG0807 | 25 | GTP cyclohydrolase II | | |
| COG0809 | 34 | S-adenosylmethionine:tRNA-ribosyltransferase-isomerase (queuine synthetase) | | |
| COG0810 | 28 | Periplasmic protein TonB, links inner and outer membranes | | |
| COG0811 | 29 | Biopolymer transport proteins | | |
| COG0812 | 30 | UDP-N-acetylmuramate dehydrogenase | | |
| COG0813 | 4 | Purine-nucleoside phosphorylase | | |
| COG0815 | 31 | Apolipoprotein N-acyltransferase | | |
| COG0816 | 18 | Predicted endonuclease involved in recombination (possible Holliday junction resolvase in Mycoplasmas and B. subtilis) | | |
| COG0817 | 18 | Holliday junction resolvasome, endonuclease subunit | | |
| COG0818 | 5 | Diacylglycerol kinase | | |
| COG0819 | 4 | Putative transcription activator | | |
| COG0820 | 39 | Predicted Fe-S-cluster redox enzyme | | |
| COG0821 | 24 | Enzyme involved in the deoxyxylulose pathway of isoprenoid biosynthesis | | |
| COG0822 | 15 | NifU homolog involved in Fe-S cluster formation | | |
| COG0823 | 109 | Periplasmic component of the Tol biopolymer transport system | | |
| COG0824 | 48 | Predicted thioesterase | | |
| COG0825 | 20 | Acetyl-CoA carboxylase alpha subunit | | |
| COG0826 | 29 | Collagenase and related proteases | | |
| COG0827 | 5 | Adenine-specific DNA methylase | | |
| COG0828 | 4 | Ribosomal protein S21 | | |
| COG0829 | 2 | Urease accessory protein UreH | | |
| COG0830 | 1 | Urease accessory protein UreF | | |
| COG0831 | 4 | Urea amidohydrolase (urease) gamma subunit | | |
| COG0832 | 3 | Urea amidohydrolase (urease) beta subunit | | |
| COG0834 | 78 | ABC-type amino acid transport/signal transduction systems, periplasmic component/domain | | |
| COG0835 | 24 | Chemotaxis signal transduction protein | | |
| COG0836 | 36 | Mannose-1-phosphate guanylyltransferase | | |
| COG0837 | 9 | Glucokinase | | |
| COG0838 | 18 | NADH:ubiquinone oxidoreductase subunit 3 (chain A) | | |
| COG0839 | 20 | NADH:ubiquinone oxidoreductase subunit 6 (chain J) | | |
| COG0840 | 90 | Methyl-accepting chemotaxis protein | | |
| COG0841 | 410 | Cation/multidrug efflux pump | | |
| COG0842 | 141 | ABC-type multidrug transport system, permease component | | |
| COG0843 | 81 | Heme/copper-type cytochrome/quinol oxidases, subunit 1 | | |
| COG0845 | 326 | Membrane-fusion protein | | |
| COG0846 | 28 | NAD-dependent protein deacetylases, SIR2 family | | |
| COG0847 | 26 | DNA polymerase III, epsilon subunit and related 3'-5' exonucleases | | |
| COG0848 | 18 | Biopolymer transport protein | | |
| COG0849 | 32 | Actin-like ATPase involved in cell division | | |
| COG0850 | 2 | Septum formation inhibitor | | |
| COG0851 | 2 | Septum formation topological specificity factor | | |
| COG0852 | 21 | NADH:ubiquinone oxidoreductase 27 kD subunit | | |
| COG0853 | 6 | Aspartate 1-decarboxylase | | |
| COG0854 | 19 | Pyridoxal phosphate biosynthesis protein | | |
| COG0855 | 47 | Polyphosphate kinase | | |

| | | | | |
|---|---|---|---|---|
| COG0857 | 4 | BioD-like N-terminal domain of phosphotransacetylase | | |
| COG0858 | 16 | Ribosome-binding factor A | | |
| COG0859 | 52 | ADP-heptose:LPS heptosyltransferase | | |
| COG0860 | 42 | N-acetylmuramoyl-L-alanine amidase | | |
| COG0861 | 43 | Membrane protein TerC, possibly involved in tellurium resistance | | |
| COG0863 | 35 | DNA modification methylase | | |
| COG1001 | 14 | Adenine deaminase | | |
| COG1002 | 28 | Type II restriction enzyme, methylase subunits | | |
| COG1003 | 36 | Glycine cleavage system protein P (pyridoxal-binding), C-terminal domain | | |
| COG1004 | 50 | Predicted UDP-glucose 6-dehydrogenase | | |
| COG1005 | 55 | NADH:ubiquinone oxidoreductase subunit 1 (chain H) | | |
| COG1006 | 3 | Multisubunit Na+/H+ antiporter, MnhC subunit | | |
| COG1007 | 59 | NADH:ubiquinone oxidoreductase subunit 2 (chain N) | | |
| COG1008 | 63 | NADH:ubiquinone oxidoreductase subunit 4 (chain M) | | |
| COG1009 | 64 | NADH:ubiquinone oxidoreductase subunit 5 (chain L)/Multisubunit Na+/H+ antiporter, MnhA subunit | | |
| COG1010 | 6 | Precorrin-3B methylase | | |
| COG1011 | 76 | Predicted hydrolase (HAD superfamily) | | |
| COG1012 | 358 | NAD-dependent aldehyde dehydrogenases | | |
| COG1013 | 65 | Pyruvate:ferredoxin oxidoreductase and related 2-oxoacid:ferredoxin oxidoreductases, beta subunit | | |
| COG1014 | 74 | Pyruvate:ferredoxin oxidoreductase and related 2-oxoacid:ferredoxin oxidoreductases, gamma subunit | | |
| COG1015 | 18 | Phosphopentomutase | | |
| COG1017 | 1 | Hemoglobin-like flavoprotein | | |
| COG1018 | 37 | Flavodoxin reductases (ferredoxin-NADPH reductases) family 1 | | |
| COG1019 | 1 | Predicted nucleotidyltransferase | | |
| COG1020 | 110 | Non-ribosomal peptide synthetase modules and related proteins | | |
| COG1021 | 10 | Peptide arylation enzymes | | |
| COG1022 | 95 | Long-chain acyl-CoA synthetases (AMP-forming) | | |
| COG1023 | 19 | Predicted 6-phosphogluconate dehydrogenase | | |
| COG1024 | 292 | Enoyl-CoA hydratase/carnithine racemase | | |
| COG1026 | 1 | Predicted Zn-dependent peptidases, insulinase-like | | |
| COG1027 | 19 | Aspartate ammonia-lyase | | |
| COG1028 | 682 | Dehydrogenases with different specificities (related to short-chain alcohol dehydrogenases) | | |
| COG1029 | 1 | Formylmethanofuran dehydrogenase subunit B | | |
| COG1030 | 31 | Membrane-bound serine protease (ClpP class) | | |
| COG1032 | 154 | Fe-S oxidoreductase | | |
| COG1033 | 18 | Predicted exporters of the RND superfamily | | |
| COG1034 | 38 | NADH dehydrogenase/NADH:ubiquinone oxidoreductase 75 kD subunit (chain G) | | |
| COG1035 | 4 | Coenzyme F420-reducing hydrogenase, beta subunit | | |
| COG1036 | 1 | Archaeal flavoproteins | | |
| COG1038 | 22 | Pyruvate carboxylase | | |
| COG1039 | 3 | Ribonuclease HIII | | |
| COG1040 | 22 | Predicted amidophosphoribosyltransferases | | |
| COG1042 | 111 | Acyl-CoA synthetase (NDP forming) | | |
| COG1043 | 31 | Acyl-[acyl carrier protein]--UDP-N-acetylglucosamine O-acyltransferase | | |
| COG1044 | 31 | UDP-3-O-[3-hydroxymyristoyl] glucosamine N-acyltransferase | | |
| COG1045 | 18 | Serine acetyltransferase | | |
| COG1047 | 9 | FKBP-type peptidyl-prolyl cis-trans isomerases 2 | | |
| COG1048 | 69 | Aconitase A | | |
| COG1049 | 7 | Aconitase B | | |
| COG1051 | 39 | ADP-ribose pyrophosphatase | | |
| COG1052 | 38 | Lactate dehydrogenase and related dehydrogenases | | |
| COG1053 | 117 | Succinate dehydrogenase/fumarate reductase, flavoprotein subunit | | |
| COG1054 | 1 | Predicted sulfurtransferase | | |
| COG1055 | 29 | Na+/H+ antiporter NhaD and related arsenite permeases | | |
| COG1057 | 22 | Nicotinic acid mononucleotide adenylyltransferase | | |
| COG1058 | 34 | Predicted nucleotide-utilizing enzyme related to molybdopterin-biosynthesis enzyme MoeA | | |
| COG1060 | 51 | Thiamine biosynthesis enzyme ThiH and related uncharacterized enzymes | | |
| COG1061 | 38 | DNA or RNA helicases of superfamily II | | |
| COG1062 | 49 | Zn-dependent alcohol dehydrogenases, class III | | |
| COG1063 | 157 | Threonine dehydrogenase and related Zn-dependent dehydrogenases | | |
| COG1064 | 51 | Zn-dependent alcohol dehydrogenases | | |
| COG1066 | 37 | Predicted ATP-dependent serine protease | | |
| COG1067 | 32 | Predicted ATP-dependent protease | | |
| COG1069 | 7 | Ribulose kinase | | |
| COG1070 | 44 | Sugar (pentulose and hexulose) kinases | | |
| COG1071 | 92 | Pyruvate/2-oxoglutarate dehydrogenase complex, dehydrogenase (E1) component, eukaryotic type, alpha subunit | | |
| COG1072 | 4 | Panthothenate kinase | | |
| COG1073 | 69 | Hydrolases of the alpha/beta superfamily | | |
| COG1074 | 24 | ATP-dependent exoDNAse (exonuclease V) beta subunit (contains helicase and exonuclease domains) | | |
| COG1075 | 20 | Predicted acetyltransferases and hydrolases with the alpha/beta hydrolase fold | | |
| COG1076 | 10 | DnaJ-domain-containing proteins 1 | | |
| COG1077 | 34 | Actin-like ATPase involved in cell morphogenesis | | |
| COG1078 | 7 | HD superfamily phosphohydrolases | | |
| COG1079 | 28 | Uncharacterized ABC-type transport system, permease component | | |
| COG1080 | 33 | Phosphoenolpyruvate-protein kinase (PTS system EI component in bacteria) | | |
| COG1082 | 64 | Sugar phosphate isomerases/epimerases | | |
| COG1083 | 2 | CMP-N-acetylneuraminic acid synthetase | | |
| COG1085 | 25 | Galactose-1-phosphate uridylyltransferase | | |
| COG1086 | 39 | Predicted nucleoside-diphosphate sugar epimerases | | |
| COG1087 | 28 | UDP-glucose 4-epimerase | | |
| COG1088 | 25 | dTDP-D-glucose 4,6-dehydratase | | |
| COG1089 | 29 | GDP-D-mannose dehydratase | | |
| COG1090 | 17 | Predicted nucleoside-diphosphate sugar epimerase | | |
| COG1091 | 36 | dTDP-4-dehydrorhamnose reductase | | |

| | | | | |
|---|---|---|---|---|
| COG1092 | 37 | Predicted SAM-dependent methyltransferases | | |
| COG1099 | 5 | Predicted metal-dependent hydrolases with the TIM-barrel fold | | |
| COG1100 | 13 | GTPase SAR1 and related small G proteins | | |
| COG1102 | 1 | Cytidylate kinase | | |
| COG1104 | 54 | Cysteine sulfinate desulfinase/cysteine desulfurase and related enzymes | | |
| COG1105 | 14 | Fructose-1-phosphate kinase and related fructose-6-phosphate kinase (PfkB) | | |
| COG1108 | 22 | ABC-type Mn2+/Zn2+ transport systems, permease components | | |
| COG1109 | 100 | Phosphomannomutase | | |
| COG1112 | 22 | Superfamily I DNA and RNA helicases and helicase subunits | | |
| COG1113 | 2 | Gamma-aminobutyrate permease and related permeases | | |
| COG1116 | 33 | ABC-type nitrate/sulfonate/bicarbonate transport system, ATPase component | | |
| COG1117 | 14 | ABC-type phosphate transport system, ATPase component | | |
| COG1118 | 11 | ABC-type sulfate/molybdate transport systems, ATPase component | | |
| COG1119 | 6 | ABC-type molybdenum transport system, ATPase component/photorepair protein PhrA | | |
| COG1120 | 12 | ABC-type cobalamin/Fe3+-siderophores transport systems, ATPase components | | |
| COG1121 | 11 | ABC-type Mn/Zn transport systems, ATPase component | | |
| COG1122 | 25 | ABC-type cobalt transport system, ATPase component | | |
| COG1123 | 25 | ATPase components of various ABC-type transport systems, contain duplicated ATPase | | |
| COG1125 | 6 | ABC-type proline/glycine betaine transport systems, ATPase components | | |
| COG1126 | 11 | ABC-type polar amino acid transport system, ATPase component | | |
| COG1127 | 37 | ABC-type transport system involved in resistance to organic solvents, ATPase component | | |
| COG1129 | 41 | ABC-type sugar transport system, ATPase component | | |
| COG1131 | 163 | ABC-type multidrug transport system, ATPase component | | |
| COG1132 | 290 | ABC-type multidrug transport system, ATPase and permease components | | |
| COG1134 | 29 | ABC-type polysaccharide/polyol phosphate transport system, ATPase component | | |
| COG1135 | 3 | ABC-type metal ion transport system, ATPase component | | |
| COG1136 | 86 | ABC-type antimicrobial peptide transport system, ATPase component | | |
| COG1137 | 13 | ABC-type (unclassified) transport system, ATPase component | | |
| COG1138 | 47 | Cytochrome c biogenesis factor | | |
| COG1139 | 21 | Uncharacterized conserved protein containing a ferredoxin-like domain | | |
| COG1140 | 12 | Nitrate reductase beta subunit | | |
| COG1141 | 2 | Ferredoxin | | |
| COG1142 | 3 | Fe-S-cluster-containing hydrogenase components 2 | | |
| COG1143 | 30 | Formate hydrogenlyase subunit 6/NADH:ubiquinone oxidoreductase 23 kD subunit (chain I) | | |
| COG1144 | 13 | Pyruvate:ferredoxin oxidoreductase and related 2-oxoacid:ferredoxin oxidoreductases, delta subunit | | |
| COG1145 | 48 | Ferredoxin | | |
| COG1146 | 19 | Ferredoxin | | |
| COG1148 | 53 | Heterodisulfide reductase, subunit A and related polyferredoxins | | |
| COG1150 | 12 | Heterodisulfide reductase, subunit C | | |
| COG1151 | 6 | 6Fe-6S prismane cluster-containing protein | | |
| COG1152 | 2 | CO dehydrogenase/acetyl-CoA synthase alpha subunit | | |
| COG1154 | 40 | Deoxyxylulose-5-phosphate synthase | | |
| COG1155 | 14 | Archaeal/vacuolar-type H+-ATPase subunit A | | |
| COG1156 | 9 | Archaeal/vacuolar-type H+-ATPase subunit B | | |
| COG1157 | 11 | Flagellar biosynthesis/type III secretory pathway ATPase | | |
| COG1158 | 30 | Transcription termination factor | | |
| COG1159 | 30 | GTPase | | |
| COG1160 | 53 | Predicted GTPases | | |
| COG1162 | 36 | Predicted GTPases | | |
| COG1163 | 9 | Predicted GTPase | | |
| COG1164 | 43 | Oligoendopeptidase F | | |
| COG1165 | 23 | 2-succinyl-6-hydroxy-2,4-cyclohexadiene-1-carboxylate synthase | | |
| COG1166 | 21 | Arginine decarboxylase (spermidine biosynthesis) | | |
| COG1167 | 74 | Transcriptional regulators containing a DNA-binding HTH domain and an aminotransferase domain (MocR family) and their eukaryotic c | | |
| COG1168 | 11 | Bifunctional PLP-dependent enzyme with beta-cystathionase and maltose regulon repressor activities | | |
| COG1169 | 16 | Isochorismate synthase | | |
| COG1171 | 83 | Threonine dehydratase | | |
| COG1172 | 54 | Ribose/xylose/arabinose/galactoside ABC-type transport systems, permease components | | |
| COG1173 | 156 | ABC-type dipeptide/oligopeptide/nickel transport systems, permease components | | |
| COG1174 | 11 | ABC-type proline/glycine betaine transport systems, permease component | | |
| COG1175 | 133 | ABC-type sugar transport systems, permease components | | |
| COG1176 | 29 | ABC-type spermidine/putrescine transport system, permease component I | | |
| COG1177 | 41 | ABC-type spermidine/putrescine transport system, permease component II | | |
| COG1178 | 72 | ABC-type Fe3+ transport system, permease component | | |
| COG1179 | 3 | Dinucleotide-utilizing enzymes involved in molybdopterin and thiamine biosynthesis family 1 | | |
| COG1180 | 30 | Pyruvate-formate lyase-activating enzyme | | |
| COG1181 | 75 | D-alanine-D-alanine ligase and related ATP-grasp enzymes | | |
| COG1182 | 1 | Acyl carrier protein phosphodiesterase | | |
| COG1183 | 19 | Phosphatidylserine synthase | | |
| COG1185 | 39 | Polyribonucleotide nucleotidyltransferase (polynucleotide phosphorylase) | | |
| COG1186 | 27 | Protein chain release factor B | | |
| COG1187 | 46 | 16S rRNA uridine-516 pseudouridylate synthase and related pseudouridylate synthases | | |
| COG1188 | 7 | Ribosome-associated heat shock protein implicated in the recycling of the 50S subunit (S4 paralog) | | |
| COG1189 | 16 | Predicted rRNA methylase | | |
| COG1190 | 35 | Lysyl-tRNA synthetase (class II) | | |
| COG1191 | 43 | DNA-directed RNA polymerase specialized sigma subunit | | |
| COG1192 | 59 | ATPases involved in chromosome partitioning | | |
| COG1193 | 30 | Mismatch repair ATPase (MutS family) | | |
| COG1194 | 33 | A/G-specific DNA glycosylase | | |
| COG1195 | 17 | Recombinational DNA repair ATPase (RecF pathway) | | |
| COG1196 | 51 | Chromosome segregation ATPases | | |
| COG1197 | 72 | Transcription-repair coupling factor (superfamily II helicase) | | |
| COG1198 | 56 | Primosomal protein N' (replication factor Y) - superfamily II helicase | | |
| COG1199 | 57 | Rad3-related DNA helicases | | |

| | | | | |
|---|---|---|---|---|
| COG1200 | 50 | RecG-like helicase | | |
| COG1201 | 91 | Lhr-like helicases | | |
| COG1202 | 1 | Superfamily II helicase, archaea-specific | | |
| COG1203 | 6 | Predicted helicases | | |
| COG1204 | 2 | Superfamily II helicase | | |
| COG1205 | 42 | Distinct helicase family with a unique C-terminal domain including a metal-binding cysteine cluster | | |
| COG1206 | 26 | NAD(FAD)-utilizing enzyme possibly involved in translation | | |
| COG1207 | 49 | N-acetylglucosamine-1-phosphate uridyltransferase (contains nucleotidyltransferase and I-patch acetyltransferase domains) | | |
| COG1208 | 77 | Nucleoside-diphosphate-sugar pyrophosphorylase involved in lipopolysaccharide biosynthesis/translation initiation factor 2B, gamma/eps | | |
| COG1209 | 33 | dTDP-glucose pyrophosphorylase | | |
| COG1210 | 17 | UDP-glucose pyrophosphorylase | | |
| COG1211 | 19 | 4-diphosphocytidyl-2-methyl-D-erithritol synthase | | |
| COG1212 | 20 | CMP-2-keto-3-deoxyoctulosonic acid synthetase | | |
| COG1213 | 1 | Predicted sugar nucleotidyltransferases | | |
| COG1214 | 19 | Inactive homolog of metal-dependent proteases, putative molecular chaperone | | |
| COG1215 | 82 | Glycosyltransferases, probably involved in cell wall biogenesis | | |
| COG1216 | 63 | Predicted glycosyltransferases | | |
| COG1217 | 32 | Predicted membrane GTPase involved in stress response | | |
| COG1218 | 8 | 3'-Phosphoadenosine 5'-phosphosulfate (PAPS) 3'-phosphatase | | |
| COG1219 | 19 | ATP-dependent protease Clp, ATPase subunit | | |
| COG1220 | 20 | ATP-dependent protease HslVU (ClpYQ), ATPase subunit | | |
| COG1221 | 7 | Transcriptional regulators containing an AAA-type ATPase domain and a DNA-binding domain | | |
| COG1222 | 4 | ATP-dependent 26S proteasome regulatory subunit | | |
| COG1223 | 1 | Predicted ATPase (AAA+ superfamily) | | |
| COG1225 | 49 | Peroxiredoxin | | |
| COG1226 | 56 | Kef-type K+ transport systems, predicted NAD-binding component | | |
| COG1227 | 4 | Inorganic pyrophosphatase/exopolyphosphatase | | |
| COG1228 | 155 | Imidazolonepropionase and related amidohydrolases | | |
| COG1229 | 8 | Formylmethanofuran dehydrogenase subunit A | | |
| COG1230 | 33 | Co/Zn/Cd efflux system component | | |
| COG1231 | 20 | Monoamine oxidase | | |
| COG1232 | 52 | Protoporphyrinogen oxidase | | |
| COG1233 | 93 | Phytoene dehydrogenase and related proteins | | |
| COG1234 | 43 | Metal-dependent hydrolases of the beta-lactamase superfamily III | | |
| COG1235 | 67 | Metal-dependent hydrolases of the beta-lactamase superfamily I | | |
| COG1236 | 59 | Predicted exonuclease of the beta-lactamase fold involved in RNA processing | | |
| COG1237 | 12 | Metal-dependent hydrolases of the beta-lactamase superfamily II | | |
| COG1238 | 4 | Predicted membrane protein | | |
| COG1239 | 31 | Mg-chelatase subunit ChlI | | |
| COG1240 | 4 | Mg-chelatase subunit ChlD | | |
| COG1242 | 2 | Predicted Fe-S oxidoreductase | | |
| COG1243 | 4 | Histone acetyltransferase | | |
| COG1244 | 2 | Predicted Fe-S oxidoreductase | | |
| COG1246 | 14 | N-acetylglutamate synthase and related acetyltransferases | | |
| COG1247 | 21 | Sortase and related acyltransferases | | |
| COG1249 | 142 | Pyruvate/2-oxoglutarate dehydrogenase complex, dihydrolipoamide dehydrogenase (E3) component, and related enzymes | | |
| COG1250 | 96 | 3-hydroxyacyl-CoA dehydrogenase | | |
| COG1251 | 23 | NAD(P)H-nitrite reductase | | |
| COG1252 | 52 | NADH dehydrogenase, FAD-containing subunit | | |
| COG1253 | 66 | Hemolysins and related proteins containing CBS domains | | |
| COG1254 | 16 | Acylphosphatases | | |
| COG1256 | 6 | Flagellar hook-associated protein | | |
| COG1257 | 17 | Hydroxymethylglutaryl-CoA reductase | | |
| COG1259 | 18 | Uncharacterized conserved protein | | |
| COG1260 | 12 | Myo-inositol-1-phosphate synthase | | |
| COG1261 | 1 | Flagellar basal body P-ring biosynthesis protein | | |
| COG1262 | 78 | Uncharacterized conserved protein | | |
| COG1263 | 3 | Phosphotransferase system IIC components, glucose/maltose/N-acetylglucosamine-specific | | |
| COG1264 | 1 | Phosphotransferase system IIB components | | |
| COG1266 | 30 | Predicted metal-dependent membrane protease | | |
| COG1267 | 8 | Phosphatidylglycerophosphatase A and related proteins | | |
| COG1268 | 5 | Uncharacterized conserved protein | | |
| COG1269 | 4 | Archaeal/vacuolar-type H+-ATPase subunit I | | |
| COG1270 | 8 | Cobalamin biosynthesis protein CobD/CbiB | | |
| COG1271 | 33 | Cytochrome bd-type quinol oxidase, subunit 1 | | |
| COG1272 | 15 | Predicted membrane protein, hemolysin III homolog | | |
| COG1273 | 36 | Uncharacterized conserved protein | | |
| COG1274 | 30 | Phosphoenolpyruvate carboxykinase (GTP) | | |
| COG1275 | 5 | Tellurite resistance protein and related permeases | | |
| COG1277 | 29 | ABC-type transport system involved in multi-copper enzyme maturation, permease component | | |
| COG1278 | 28 | Cold shock proteins | | |
| COG1280 | 22 | Putative threonine efflux protein | | |
| COG1281 | 5 | Disulfide bond chaperones of the HSP33 family | | |
| COG1282 | 18 | NAD/NADP transhydrogenase beta subunit | | |
| COG1283 | 10 | Na+/phosphate symporter | | |
| COG1284 | 2 | Uncharacterized conserved protein | | |
| COG1285 | 11 | Uncharacterized membrane protein | | |
| COG1286 | 2 | Uncharacterized membrane protein, required for colicin V production | | |
| COG1288 | 3 | Predicted membrane protein | | |
| COG1289 | 1 | Predicted membrane protein | | |
| COG1290 | 55 | Cytochrome b subunit of the bc complex | | |
| COG1291 | 5 | Flagellar motor component | | |
| COG1292 | 4 | Choline-glycine betaine transporter | | |
| COG1293 | 6 | Predicted RNA-binding protein homologous to eukaryotic snRNP | | |

| | | | | |
|---|---|---|---|---|
| COG1294 | 19 | Cytochrome bd-type quinol oxidase, subunit 2 | | |
| COG1295 | 45 | Predicted membrane protein | | |
| COG1296 | 10 | Predicted branched-chain amino acid permease (azaleucine resistance) | | |
| COG1297 | 43 | Predicted membrane protein | | |
| COG1298 | 16 | Flagellar biosynthesis pathway, component FlhA | | |
| COG1299 | 1 | Phosphotransferase system, fructose-specific IIC component | | |
| COG1300 | 14 | Uncharacterized membrane protein | | |
| COG1301 | 31 | Na+/H+-dicarboxylate symporters | | |
| COG1302 | 2 | Uncharacterized protein conserved in bacteria | | |
| COG1304 | 58 | L-lactate dehydrogenase (FMN-dependent) and related alpha-hydroxy acid dehydrogenases | | |
| COG1305 | 63 | Transglutaminase-like enzymes, putative cysteine proteases | | |
| COG1306 | 11 | Uncharacterized conserved protein | | |
| COG1307 | 21 | Uncharacterized protein conserved in bacteria | | |
| COG1308 | 1 | Transcription factor homologous to NACalpha-BTF3 | | |
| COG1309 | 219 | Transcriptional regulator | | |
| COG1310 | 4 | Predicted metal-dependent protease of the PAD1/JAB1 superfamily | | |
| COG1312 | 7 | D-mannonate dehydratase | | |
| COG1313 | 12 | Uncharacterized Fe-S protein PflX, homolog of pyruvate formate lyase activating proteins | | |
| COG1314 | 2 | Preprotein translocase subunit SecG | | |
| COG1316 | 31 | Transcriptional regulator | | |
| COG1317 | 3 | Flagellar biosynthesis/type III secretory pathway protein | | |
| COG1318 | 1 | Predicted transcriptional regulators | | |
| COG1319 | 72 | Aerobic-type carbon monoxide dehydrogenase, middle subunit CoxM/CutM homologs | | |
| COG1320 | 1 | Multisubunit Na+/H+ antiporter, MnhG subunit | | |
| COG1321 | 25 | Mn-dependent transcriptional regulator | | |
| COG1322 | 28 | Uncharacterized protein conserved in bacteria | | |
| COG1324 | 9 | Uncharacterized protein involved in tolerance to divalent cations | | |
| COG1327 | 6 | Predicted transcriptional regulator, consists of a Zn-ribbon and ATP-cone domains | | |
| COG1328 | 5 | Oxygen-sensitive ribonucleoside-triphosphate reductase | | |
| COG1329 | 12 | Transcriptional regulators, similar to M. xanthus CarD | | |
| COG1330 | 10 | Exonuclease V gamma subunit | | |
| COG1331 | 42 | Highly conserved protein containing a thioredoxin domain | | |
| COG1333 | 12 | ResB protein required for cytochrome c biosynthesis | | |
| COG1334 | 1 | Uncharacterized flagellar protein FlaG | | |
| COG1335 | 60 | Amidases related to nicotinamidase | | |
| COG1338 | 6 | Flagellar biosynthesis pathway, component FliP | | |
| COG1341 | 1 | Predicted GTPase or GTP-binding protein | | |
| COG1343 | 1 | Uncharacterized protein predicted to be involved in DNA repair | | |
| COG1344 | 18 | Flagellin and related hook-associated proteins | | |
| COG1345 | 9 | Flagellar capping protein | | |
| COG1346 | 1 | Putative effector of murein hydrolase | | |
| COG1347 | 2 | Na+-transporting NADH:ubiquinone oxidoreductase, subunit NqrD | | |
| COG1348 | 1 | Nitrogenase subunit NifH (ATPase) | | |
| COG1349 | 22 | Transcriptional regulators of sugar metabolism | | |
| COG1350 | 18 | Predicted alternative tryptophan synthase beta-subunit (paralog of TrpB) | | |
| COG1351 | 21 | Predicted alternative thymidylate synthase | | |
| COG1352 | 58 | Methylase of chemotaxis methyl-accepting proteins | | |
| COG1354 | 19 | Uncharacterized conserved protein | | |
| COG1355 | 16 | Predicted dioxygenase | | |
| COG1357 | 13 | Uncharacterized low-complexity proteins | | |
| COG1358 | 4 | Ribosomal protein HS6-type (S12/L30/L7a) | | |
| COG1359 | 5 | Uncharacterized conserved protein | | |
| COG1360 | 17 | Flagellar motor protein | | |
| COG1362 | 2 | Aspartyl aminopeptidase | | |
| COG1363 | 27 | Cellulase M and related proteins | | |
| COG1364 | 31 | N-acetylglutamate synthase (N-acetylornithine aminotransferase) | | |
| COG1366 | 40 | Anti-anti-sigma regulatory factor (antagonist of anti-sigma factor) | | |
| COG1368 | 13 | Phosphoglycerol transferase and related proteins, alkaline phosphatase superfamily | | |
| COG1371 | 5 | Uncharacterized conserved protein | | |
| COG1372 | 13 | Intein/homing endonuclease | | |
| COG1373 | 11 | Predicted ATPase (AAA+ superfamily) | | |
| COG1376 | 47 | Uncharacterized protein conserved in bacteria | | |
| COG1377 | 6 | Flagellar biosynthesis pathway, component FlhB | | |
| COG1378 | 1 | Predicted transcriptional regulators | | |
| COG1379 | 2 | Uncharacterized conserved protein | | |
| COG1380 | 2 | Putative effector of murein hydrolase LrgA | | |
| COG1381 | 23 | Recombinational DNA repair protein (RecF pathway) | | |
| COG1384 | 4 | Lysyl-tRNA synthetase (class I) | | |
| COG1385 | 18 | Uncharacterized protein conserved in bacteria | | |
| COG1386 | 16 | Predicted transcriptional regulator containing the HTH domain | | |
| COG1387 | 25 | Histidinol phosphatase and related hydrolases of the PHP family | | |
| COG1388 | 8 | FOG: LysM repeat | | |
| COG1389 | 4 | DNA topoisomerase VI, subunit B | | |
| COG1390 | 1 | Archaeal/vacuolar-type H+-ATPase subunit E | | |
| COG1391 | 54 | Glutamine synthetase adenylyltransferase | | |
| COG1392 | 19 | Phosphate transport regulator (distant homolog of PhoU) | | |
| COG1393 | 7 | Arsenate reductase and related proteins, glutaredoxin family | | |
| COG1394 | 3 | Archaeal/vacuolar-type H+-ATPase subunit D | | |
| COG1396 | 49 | Predicted transcriptional regulators | | |
| COG1397 | 7 | ADP-ribosylglycohydrolase | | |
| COG1398 | 40 | Fatty-acid desaturase | | |
| COG1399 | 22 | Predicted metal-binding, possibly nucleic acid-binding protein | | |
| COG1401 | 2 | GTPase subunit of restriction endonuclease | | |
| COG1402 | 24 | Uncharacterized protein, putative amidase | | |

| | | | | |
|---|---|---|---|---|
| COG1403 | 52 | Restriction endonuclease | | |
| COG1404 | 158 | Subtilisin-like serine proteases | | |
| COG1407 | 2 | Predicted ICC-like phosphoesterases | | |
| COG1408 | 25 | Predicted phosphohydrolases | | |
| COG1409 | 59 | Predicted phosphohydrolases | | |
| COG1410 | 63 | Methionine synthase I, cobalamin-binding domain | | |
| COG1411 | 1 | Uncharacterized protein related to proFAR isomerase (HisA) | | |
| COG1413 | 31 | FOG: HEAT repeat | | |
| COG1414 | 60 | Transcriptional regulator | | |
| COG1415 | 8 | Uncharacterized conserved protein | | |
| COG1416 | 3 | Uncharacterized conserved protein | | |
| COG1418 | 25 | Predicted HD superfamily hydrolase | | |
| COG1419 | 6 | Flagellar GTP-binding protein | | |
| COG1420 | 36 | Transcriptional regulator of heat shock gene | | |
| COG1426 | 15 | Uncharacterized protein conserved in bacteria | | |
| COG1427 | 7 | Predicted periplasmic solute-binding protein | | |
| COG1428 | 23 | Deoxynucleoside kinases | | |
| COG1429 | 17 | Cobalamin biosynthesis protein CobN and related Mg-chelatases | | |
| COG1430 | 14 | Uncharacterized conserved protein | | |
| COG1432 | 10 | Uncharacterized conserved protein | | |
| COG1434 | 13 | Uncharacterized conserved protein | | |
| COG1435 | 7 | Thymidine kinase | | |
| COG1437 | 1 | Adenylate cyclase, class 2 (thermophilic) | | |
| COG1438 | 7 | Arginine repressor | | |
| COG1443 | 3 | Isopentenyldiphosphate isomerase | | |
| COG1446 | 19 | Asparaginase | | |
| COG1448 | 8 | Aspartate/tyrosine/aromatic aminotransferase | | |
| COG1449 | 34 | Alpha-amylase/alpha-mannosidase | | |
| COG1450 | 53 | Type II secretory pathway, component PulD | | |
| COG1451 | 12 | Predicted metal-dependent hydrolase | | |
| COG1452 | 27 | Organic solvent tolerance protein OstA | | |
| COG1453 | 4 | Predicted oxidoreductases of the aldo/keto reductase family | | |
| COG1454 | 36 | Alcohol dehydrogenase, class IV | | |
| COG1456 | 5 | CO dehydrogenase/acetyl-CoA synthase gamma subunit (corrinoid Fe-S protein) | | |
| COG1457 | 13 | Purine-cytosine permease and related proteins | | |
| COG1459 | 106 | Type II secretory pathway, component PulF | | |
| COG1461 | 18 | Predicted kinase related to dihydroxyacetone kinase | | |
| COG1462 | 9 | Uncharacterized protein involved in formation of curli polymers | | |
| COG1463 | 58 | ABC-type transport system involved in resistance to organic solvents, periplasmic component | | |
| COG1464 | 2 | ABC-type metal ion transport system, periplasmic component/surface antigen | | |
| COG1465 | 2 | Predicted alternative 3-dehydroquinate synthase | | |
| COG1466 | 11 | DNA polymerase III, delta subunit | | |
| COG1467 | 1 | Eukaryotic-type DNA primase, catalytic (small) subunit | | |
| COG1468 | 3 | RecB family exonuclease | | |
| COG1469 | 8 | Uncharacterized conserved protein | | |
| COG1470 | 4 | Predicted membrane protein | | |
| COG1471 | 1 | Ribosomal protein S4E | | |
| COG1472 | 105 | Beta-glucosidase-related glycosidases | | |
| COG1473 | 80 | Metal-dependent amidase/aminoacylase/carboxypeptidase | | |
| COG1474 | 2 | Cdc6-related protein, AAA superfamily ATPase | | |
| COG1475 | 44 | Predicted transcriptional regulators | | |
| COG1476 | 1 | Predicted transcriptional regulators | | |
| COG1477 | 31 | Membrane-associated lipoprotein involved in thiamine biosynthesis | | |
| COG1478 | 8 | Uncharacterized conserved protein | | |
| COG1479 | 9 | Uncharacterized conserved protein | | |
| COG1480 | 11 | Predicted membrane-associated HD superfamily hydrolase | | |
| COG1481 | 4 | Uncharacterized protein conserved in bacteria | | |
| COG1482 | 11 | Phosphomannose isomerase | | |
| COG1483 | 4 | Predicted ATPase (AAA+ superfamily) | | |
| COG1484 | 48 | DNA replication protein | | |
| COG1485 | 7 | Predicted ATPase | | |
| COG1486 | 16 | Alpha-galactosidases/6-phospho-beta-glucosidases, family 4 of glycosyl hydrolases | | |
| COG1487 | 8 | Predicted nucleic acid-binding protein, contains PIN domain | | |
| COG1488 | 46 | Nicotinic acid phosphoribosyltransferase | | |
| COG1489 | 2 | DNA-binding protein, stimulates sugar fermentation | | |
| COG1490 | 17 | D-Tyr-tRNAtyr deacylase | | |
| COG1492 | 9 | Cobyric acid synthase | | |
| COG1493 | 16 | Serine kinase of the HPr protein, regulates carbohydrate metabolism | | |
| COG1494 | 19 | Fructose-1,6-bisphosphatase/sedoheptulose 1,7-bisphosphatase and related proteins | | |
| COG1495 | 8 | Disulfide bond formation protein DsbB | | |
| COG1496 | 25 | Uncharacterized conserved protein | | |
| COG1501 | 24 | Alpha-glucosidases, family 31 of glycosyl hydrolases | | |
| COG1502 | 72 | Phosphatidylserine/phosphatidylglycerophosphate/cardiolipin synthases and related enzymes | | |
| COG1503 | 1 | Peptide chain release factor 1 (eRF1) | | |
| COG1504 | 1 | Uncharacterized conserved protein | | |
| COG1505 | 59 | Serine proteases of the peptidase family S9A | | |
| COG1506 | 217 | Dipeptidyl aminopeptidases/acylaminoacyl-peptidases | | |
| COG1507 | 2 | Uncharacterized conserved protein | | |
| COG1508 | 40 | DNA-directed RNA polymerase specialized sigma subunit, sigma54 homolog | | |
| COG1509 | 25 | Lysine 2,3-aminomutase | | |
| COG1510 | 5 | Predicted transcriptional regulators | | |
| COG1511 | 2 | Predicted membrane protein | | |
| COG1512 | 14 | Beta-propeller domains of methanol dehydrogenase type | | |
| COG1513 | 1 | Cyanate lyase | | |

| | | | | |
|---|---|---|---|---|
| COG1514 | 8 | 2'-5' RNA ligase | | |
| COG1515 | 3 | Deoxyinosine 3'endonuclease (endonuclease V) | | |
| COG1516 | 3 | Flagellin-specific chaperone FliS | | |
| COG1518 | 9 | Uncharacterized protein predicted to be involved in DNA repair | | |
| COG1519 | 16 | 3-deoxy-D-manno-octulosonic-acid transferase | | |
| COG1520 | 51 | FOG: WD40-like repeat | | |
| COG1521 | 30 | Putative transcriptional regulator, homolog of Bvg accessory factor | | |
| COG1522 | 54 | Transcriptional regulators | | |
| COG1523 | 36 | Type II secretory pathway, pullulanase PulA and related glycosidases | | |
| COG1524 | 34 | Uncharacterized proteins of the AP superfamily | | |
| COG1525 | 9 | Micrococcal nuclease (thermonuclease) homologs | | |
| COG1526 | 16 | Uncharacterized protein required for formate dehydrogenase activity | | |
| COG1528 | 6 | Ferritin-like protein | | |
| COG1529 | 482 | Aerobic-type carbon monoxide dehydrogenase, large subunit CoxL/CutL homologs | | |
| COG1530 | 43 | Ribonucleases G and E | | |
| COG1533 | 27 | DNA repair photolyase | | |
| COG1534 | 3 | Predicted RNA-binding protein containing KH domain, possibly ribosomal protein | | |
| COG1536 | 14 | Flagellar motor switch protein | | |
| COG1538 | 123 | Outer membrane protein | | |
| COG1539 | 13 | Dihydroneopterin aldolase | | |
| COG1540 | 7 | Uncharacterized proteins, homologs of lactam utilization protein B | | |
| COG1541 | 72 | Coenzyme F390 synthetase | | |
| COG1543 | 18 | Uncharacterized conserved protein | | |
| COG1544 | 17 | Ribosome-associated protein Y (PSrp-1) | | |
| COG1545 | 20 | Predicted nucleic-acid-binding protein containing a Zn-ribbon | | |
| COG1546 | 24 | Uncharacterized protein (competence- and mitomycin-induced) | | |
| COG1547 | 3 | Uncharacterized conserved protein | | |
| COG1548 | 2 | Predicted transcriptional regulator/sugar kinase | | |
| COG1550 | 6 | Uncharacterized protein conserved in bacteria | | |
| COG1551 | 5 | Carbon storage regulator (could also regulate swarming and quorum sensing) | | |
| COG1553 | 2 | Uncharacterized conserved protein involved in intracellular sulfur reduction | | |
| COG1554 | 12 | Trehalose and maltose hydrolases (possible phosphorylases) | | |
| COG1555 | 12 | DNA uptake protein and related DNA-binding proteins | | |
| COG1556 | 10 | Uncharacterized conserved protein | | |
| COG1558 | 6 | Flagellar basal body rod protein | | |
| COG1559 | 28 | Predicted periplasmic solute-binding protein | | |
| COG1560 | 27 | Lauroyl/myristoyl acyltransferase | | |
| COG1561 | 19 | Uncharacterized stress-induced protein | | |
| COG1562 | 43 | Phytoene/squalene synthetase | | |
| COG1565 | 13 | Uncharacterized conserved protein | | |
| COG1566 | 22 | Multidrug resistance efflux pump | | |
| COG1568 | 1 | Predicted methyltransferases | | |
| COG1569 | 2 | Predicted nucleic acid-binding protein, contains PIN domain | | |
| COG1570 | 44 | Exonuclease VII, large subunit | | |
| COG1573 | 55 | Uracil-DNA glycosylase | | |
| COG1574 | 88 | Predicted metal-dependent hydrolase with the TIM-barrel fold | | |
| COG1575 | 10 | 1,4-dihydroxy-2-naphthoate octaprenyltransferase | | |
| COG1576 | 10 | Uncharacterized conserved protein | | |
| COG1577 | 1 | Mevalonate kinase | | |
| COG1578 | 3 | Uncharacterized conserved protein | | |
| COG1579 | 10 | Zn-ribbon protein, possibly nucleic acid-binding | | |
| COG1584 | 6 | Predicted membrane protein | | |
| COG1585 | 2 | Membrane protein implicated in regulation of membrane protease activity | | |
| COG1586 | 3 | S-adenosylmethionine decarboxylase | | |
| COG1587 | 22 | Uroporphyrinogen-III synthase | | |
| COG1589 | 14 | Cell division septal protein | | |
| COG1592 | 10 | Rubrerythrin | | |
| COG1593 | 129 | TRAP-type C4-dicarboxylate transport system, large permease component | | |
| COG1595 | 311 | DNA-directed RNA polymerase specialized sigma subunit, sigma24 homolog | | |
| COG1596 | 49 | Periplasmic protein involved in polysaccharide export | | |
| COG1597 | 38 | Sphingosine kinase and enzymes related to eukaryotic diacylglycerol kinase | | |
| COG1598 | 6 | Uncharacterized conserved protein | | |
| COG1600 | 28 | Uncharacterized Fe-S protein | | |
| COG1601 | 1 | Translation initiation factor 2, beta subunit (eIF-2beta)/eIF-5 N-terminal domain | | |
| COG1605 | 3 | Chorismate mutase | | |
| COG1606 | 13 | ATP-utilizing enzymes of the PP-loop superfamily | | |
| COG1607 | 18 | Acyl-CoA hydrolase | | |
| COG1608 | 1 | Predicted archaeal kinase | | |
| COG1609 | 73 | Transcriptional regulators | | |
| COG1610 | 10 | Uncharacterized conserved protein | | |
| COG1611 | 34 | Predicted Rossmann fold nucleotide-binding protein | | |
| COG1612 | 27 | Uncharacterized protein required for cytochrome oxidase assembly | | |
| COG1613 | 16 | ABC-type sulfate transport system, periplasmic component | | |
| COG1614 | 6 | CO dehydrogenase/acetyl-CoA synthase beta subunit | | |
| COG1615 | 29 | Uncharacterized conserved protein | | |
| COG1619 | 10 | Uncharacterized proteins, homologs of microcin C7 resistance protein MccF | | |
| COG1620 | 13 | L-lactate permease | | |
| COG1621 | 5 | Beta-fructosidases (levanase/invertase) | | |
| COG1622 | 56 | Heme/copper-type cytochrome/quinol oxidases, subunit 2 | | |
| COG1623 | 10 | Predicted nucleic-acid-binding protein (contains the HHH domain) | | |
| COG1624 | 21 | Uncharacterized conserved protein | | |
| COG1625 | 8 | Fe-S oxidoreductase, related to NifB/MoaA family | | |
| COG1626 | 3 | Neutral trehalase | | |
| COG1628 | 2 | Uncharacterized conserved protein | | |

| | | | | |
|---|---|---|---|---|
| COG1629 | 191 | Outer membrane receptor proteins, mostly Fe transport | | |
| COG1632 | 1 | Ribosomal protein L15E | | |
| COG1633 | 10 | Uncharacterized conserved protein | | |
| COG1635 | 1 | Flavoprotein involved in thiazole biosynthesis | | |
| COG1636 | 1 | Uncharacterized protein conserved in bacteria | | |
| COG1637 | 3 | Predicted nuclease of the RecB family | | |
| COG1638 | 107 | TRAP-type C4-dicarboxylate transport system, periplasmic component | | |
| COG1639 | 7 | Predicted signal transduction protein | | |
| COG1640 | 51 | 4-alpha-glucanotransferase | | |
| COG1641 | 25 | Uncharacterized conserved protein | | |
| COG1643 | 53 | HrpA-like helicases | | |
| COG1646 | 1 | Predicted phosphate-binding enzymes, TIM-barrel fold | | |
| COG1647 | 7 | Esterase/lipase | | |
| COG1648 | 9 | Siroheme synthase (precorrin-2 oxidase/ferrochelatase domain) | | |
| COG1649 | 12 | Uncharacterized protein conserved in bacteria | | |
| COG1651 | 46 | Protein-disulfide isomerase | | |
| COG1652 | 15 | Uncharacterized protein containing LysM domain | | |
| COG1653 | 166 | ABC-type sugar transport system, periplasmic component | | |
| COG1654 | 2 | Biotin operon repressor | | |
| COG1656 | 9 | Uncharacterized conserved protein | | |
| COG1657 | 23 | Squalene cyclase | | |
| COG1659 | 3 | Uncharacterized protein, linocin/CFP29 homolog | | |
| COG1660 | 19 | Predicted P-loop-containing kinase | | |
| COG1661 | 3 | Predicted DNA-binding protein with PD1-like DNA-binding motif | | |
| COG1662 | 1 | Transposase and inactivated derivatives, IS1 family | | |
| COG1663 | 10 | Tetraacyldisaccharide-1-P 4'-kinase | | |
| COG1664 | 24 | Integral membrane protein CcmA involved in cell shape determination | | |
| COG1666 | 13 | Uncharacterized protein conserved in bacteria | | |
| COG1668 | 20 | ABC-type Na+ efflux pump, permease component | | |
| COG1669 | 6 | Predicted nucleotidyltransferases | | |
| COG1670 | 69 | Acetyltransferases, including N-acetylases of ribosomal proteins | | |
| COG1671 | 5 | Uncharacterized protein conserved in bacteria | | |
| COG1672 | 5 | Predicted ATPase (AAA+ superfamily) | | |
| COG1674 | 58 | DNA segregation ATPase FtsK/SpoIIIE and related proteins | | |
| COG1677 | 1 | Flagellar hook-basal body protein | | |
| COG1678 | 24 | Putative transcriptional regulator | | |
| COG1679 | 10 | Uncharacterized conserved protein | | |
| COG1680 | 170 | Beta-lactamase class C and other penicillin binding proteins | | |
| COG1682 | 49 | ABC-type polysaccharide/polyol phosphate export systems, permease component | | |
| COG1683 | 8 | Uncharacterized conserved protein | | |
| COG1684 | 2 | Flagellar biosynthesis pathway, component FliR | | |
| COG1686 | 36 | D-alanyl-D-alanine carboxypeptidase | | |
| COG1690 | 47 | Uncharacterized conserved protein | | |
| COG1691 | 15 | NCAIR mutase (PurE)-related proteins | | |
| COG1692 | 19 | Uncharacterized protein conserved in bacteria | | |
| COG1694 | 25 | Predicted pyrophosphatase | | |
| COG1695 | 32 | Predicted transcriptional regulators | | |
| COG1696 | 19 | Predicted membrane protein involved in D-alanine export | | |
| COG1697 | 6 | DNA topoisomerase VI, subunit A | | |
| COG1702 | 19 | Phosphate starvation-inducible protein PhoH, predicted ATPase | | |
| COG1703 | 27 | Putative periplasmic protein kinase ArgK and related GTPases of G3E family | | |
| COG1704 | 31 | Uncharacterized conserved protein | | |
| COG1705 | 2 | Muramidase (flagellum-specific) | | |
| COG1706 | 4 | Flagellar basal-body P-ring protein | | |
| COG1707 | 1 | ACT domain-containing protein | | |
| COG1708 | 3 | Predicted nucleotidyltransferases | | |
| COG1712 | 7 | Predicted dinucleotide-utilizing enzyme | | |
| COG1714 | 18 | Predicted membrane protein/domain | | |
| COG1715 | 1 | Restriction endonuclease | | |
| COG1716 | 58 | FOG: FHA domain | | |
| COG1718 | 2 | Serine/threonine protein kinase involved in cell cycle control | | |
| COG1720 | 10 | Uncharacterized conserved protein | | |
| COG1721 | 74 | Uncharacterized conserved protein (some members contain a von Willebrand factor type A (vWA) domain) | | |
| COG1722 | 5 | Exonuclease VII small subunit | | |
| COG1724 | 2 | Predicted periplasmic or secreted lipoprotein | | |
| COG1725 | 8 | Predicted transcriptional regulators | | |
| COG1726 | 2 | Na+-transporting NADH:ubiquinone oxidoreductase, subunit NqrA | | |
| COG1727 | 1 | Ribosomal protein L18E | | |
| COG1729 | 12 | Uncharacterized protein conserved in bacteria | | |
| COG1732 | 25 | Periplasmic glycine betaine/choline-binding (lipo)protein of an ABC-type transport system (osmoprotectant binding protein) | | |
| COG1733 | 38 | Predicted transcriptional regulators | | |
| COG1734 | 34 | DnaK suppressor protein | | |
| COG1735 | 10 | Predicted metal-dependent hydrolase with the TIM-barrel fold | | |
| COG1737 | 8 | Transcriptional regulators | | |
| COG1738 | 5 | Uncharacterized conserved protein | | |
| COG1740 | 22 | Ni,Fe-hydrogenase I small subunit | | |
| COG1741 | 40 | Pirin-related protein | | |
| COG1743 | 12 | Adenine-specific DNA methylase containing a Zn-ribbon | | |
| COG1744 | 28 | Uncharacterized ABC-type transport system, periplasmic component/surface lipoprotein | | |
| COG1746 | 1 | tRNA nucleotidyltransferase (CCA-adding enzyme) | | |
| COG1747 | 1 | Uncharacterized N-terminal domain of the transcription elongation factor GreA | | |
| COG1748 | 17 | Saccharopine dehydrogenase and related proteins | | |
| COG1749 | 7 | Flagellar hook protein FlgE | | |
| COG1750 | 1 | Archaeal serine proteases | | |

| | | | | |
|---|---|---|---|---|
| COG1752 | 81 | Predicted esterase of the alpha-beta hydrolase superfamily | | |
| COG1754 | 11 | Uncharacterized C-terminal domain of topoisomerase IA | | |
| COG1755 | 2 | Uncharacterized protein conserved in bacteria | | |
| COG1758 | 3 | DNA-directed RNA polymerase, subunit K/omega | | |
| COG1760 | 8 | L-serine deaminase | | |
| COG1762 | 17 | Phosphotransferase system mannitol/fructose-specific IIA domain (Ntr-type) | | |
| COG1763 | 6 | Molybdopterin-guanine dinucleotide biosynthesis protein | | |
| COG1764 | 28 | Predicted redox protein, regulator of disulfide bond formation | | |
| COG1765 | 21 | Predicted redox protein, regulator of disulfide bond formation | | |
| COG1766 | 14 | Flagellar biosynthesis/type III secretory pathway lipoprotein | | |
| COG1770 | 24 | Protease II | | |
| COG1773 | 3 | Rubredoxin | | |
| COG1774 | 12 | Uncharacterized homolog of PSP1 | | |
| COG1775 | 19 | Benzoyl-CoA reductase/2-hydroxyglutaryl-CoA dehydratase subunit, BcrC/BadD/HgdB | | |
| COG1776 | 4 | Chemotaxis protein CheC, inhibitor of MCP methylation | | |
| COG1778 | 14 | Low specificity phosphatase (HAD superfamily) | | |
| COG1781 | 1 | Aspartate carbamoyltransferase, regulatory subunit | | |
| COG1782 | 1 | Predicted metal-dependent RNase, consists of a metallo-beta-lactamase domain and an RNA-binding KH domain | | |
| COG1783 | 2 | Phage terminase large subunit | | |
| COG1785 | 10 | Alkaline phosphatase | | |
| COG1786 | 5 | Uncharacterized conserved protein | | |
| COG1788 | 35 | Acyl CoA:acetate/3-ketoacid CoA transferase, alpha subunit | | |
| COG1791 | 1 | Uncharacterized conserved protein, contains double-stranded beta-helix domain | | |
| COG1792 | 23 | Cell shape-determining protein | | |
| COG1793 | 111 | ATP-dependent DNA ligase | | |
| COG1794 | 15 | Aspartate racemase | | |
| COG1795 | 1 | Uncharacterized conserved protein | | |
| COG1796 | 25 | DNA polymerase IV (family X) | | |
| COG1797 | 16 | Cobyrinic acid a,c-diamide synthase | | |
| COG1799 | 3 | Uncharacterized protein conserved in bacteria | | |
| COG1801 | 61 | Uncharacterized conserved protein | | |
| COG1802 | 40 | Transcriptional regulators | | |
| COG1803 | 5 | Methylglyoxal synthase | | |
| COG1804 | 195 | Predicted acyl-CoA transferases/carnitine dehydratase | | |
| COG1805 | 6 | Na+-transporting NADH:ubiquinone oxidoreductase, subunit NqrB | | |
| COG1806 | 9 | Uncharacterized protein conserved in bacteria | | |
| COG1807 | 54 | 4-amino-4-deoxy-L-arabinose transferase and related glycosyltransferases of PMT family | | |
| COG1809 | 6 | Uncharacterized conserved protein | | |
| COG1810 | 2 | Uncharacterized protein conserved in archaea | | |
| COG1811 | 2 | Uncharacterized membrane protein, possible Na+ channel or pump | | |
| COG1812 | 9 | Archaeal S-adenosylmethionine synthetase | | |
| COG1814 | 22 | Uncharacterized membrane protein | | |
| COG1815 | 5 | Flagellar basal body protein | | |
| COG1816 | 34 | Adenosine deaminase | | |
| COG1817 | 3 | Uncharacterized protein conserved in archaea | | |
| COG1819 | 23 | Glycosyl transferases, related to UDP-glucuronosyltransferase | | |
| COG1820 | 12 | N-acetylglucosamine-6-phosphate deacetylase | | |
| COG1824 | 3 | Permease, similar to cation transporters | | |
| COG1825 | 28 | Ribosomal protein L25 (general stress protein Ctc) | | |
| COG1826 | 9 | Sec-independent protein secretion pathway components | | |
| COG1828 | 4 | Phosphoribosylformylglycinamidine (FGAM) synthase, PurS component | | |
| COG1830 | 13 | DhnA-type fructose-1,6-bisphosphate aldolase and related enzymes | | |
| COG1832 | 31 | Predicted CoA-binding protein | | |
| COG1833 | 1 | Uncharacterized conserved protein | | |
| COG1834 | 18 | N-Dimethylarginine dimethylaminohydrolase | | |
| COG1835 | 31 | Predicted acyltransferases | | |
| COG1836 | 1 | Predicted membrane protein | | |
| COG1837 | 8 | Predicted RNA-binding protein (contains KH domain) | | |
| COG1838 | 10 | Tartrate dehydratase beta subunit/Fumarate hydratase class I, C-terminal domain | | |
| COG1839 | 5 | Uncharacterized conserved protein | | |
| COG1840 | 57 | ABC-type Fe3+ transport system, periplasmic component | | |
| COG1841 | 5 | Ribosomal protein L30/L7E | | |
| COG1842 | 16 | Phage shock protein A (IM30), suppresses sigma54-dependent transcription | | |
| COG1843 | 6 | Flagellar hook capping protein | | |
| COG1845 | 36 | Heme/copper-type cytochrome/quinol oxidase, subunit 3 | | |
| COG1846 | 95 | Transcriptional regulators | | |
| COG1847 | 7 | Predicted RNA-binding protein | | |
| COG1848 | 11 | Predicted nucleic acid-binding protein, contains PIN domain | | |
| COG1850 | 7 | Ribulose 1,5-bisphosphate carboxylase, large subunit | | |
| COG1852 | 2 | Uncharacterized conserved protein | | |
| COG1853 | 41 | Conserved protein/domain typically associated with flavoprotein oxygenases, DIM6/NTAB family | | |
| COG1856 | 3 | Uncharacterized homolog of biotin synthetase | | |
| COG1858 | 50 | Cytochrome c peroxidase | | |
| COG1859 | 1 | RNA:NAD 2'-phosphotransferase | | |
| COG1861 | 5 | Spore coat polysaccharide biosynthesis protein F, CMP-KDO synthetase homolog | | |
| COG1862 | 8 | Preprotein translocase subunit YajC | | |
| COG1864 | 5 | DNA/RNA endonuclease G, NUC1 | | |
| COG1866 | 20 | Phosphoenolpyruvate carboxykinase (ATP) | | |
| COG1868 | 11 | Flagellar motor switch protein | | |
| COG1869 | 1 | ABC-type ribose transport system, auxiliary component | | |
| COG1871 | 6 | Chemotaxis protein; stimulates methylation of MCP proteins | | |
| COG1872 | 12 | Uncharacterized conserved protein | | |
| COG1874 | 14 | Beta-galactosidase | | |
| COG1875 | 18 | Predicted ATPase related to phosphate starvation-inducible protein PhoH | | |

| | | | | |
|---|---|---|---|---|
| COG1876 | 5 | D-alanyl-D-alanine carboxypeptidase | | |
| COG1877 | 13 | Trehalose-6-phosphatase | | |
| COG1878 | 24 | Predicted metal-dependent hydrolase | | |
| COG1879 | 53 | ABC-type sugar transport system, periplasmic component | | |
| COG1881 | 25 | Phospholipid-binding protein | | |
| COG1882 | 20 | Pyruvate-formate lyase | | |
| COG1883 | 5 | Na+-transporting methylmalonyl-CoA/oxaloacetate decarboxylase, beta subunit | | |
| COG1884 | 72 | Methylmalonyl-CoA mutase, N-terminal domain/subunit | | |
| COG1886 | 4 | Flagellar motor switch/type III secretory pathway protein | | |
| COG1887 | 12 | Putative glycosyl/glycerophosphate transferases involved in teichoic acid biosynthesis TagF/TagB/EpsJ/RodC | | |
| COG1890 | 1 | Ribosomal protein S3AE | | |
| COG1893 | 42 | Ketopantoate reductase | | |
| COG1894 | 82 | NADH:ubiquinone oxidoreductase, NADH-binding (51 kD) subunit | | |
| COG1895 | 1 | Uncharacterized conserved protein related to C-terminal domain of eukaryotic chaperone, SACSIN | | |
| COG1896 | 1 | Predicted hydrolases of HD superfamily | | |
| COG1897 | 1 | Homoserine trans-succinylase | | |
| COG1898 | 34 | dTDP-4-dehydrorhamnose 3,5-epimerase and related enzymes | | |
| COG1899 | 18 | Deoxyhypusine synthase | | |
| COG1902 | 70 | NADH:flavin oxidoreductases, Old Yellow Enzyme family | | |
| COG1903 | 4 | Cobalamin biosynthesis protein CbiD | | |
| COG1904 | 6 | Glucuronate isomerase | | |
| COG1905 | 29 | NADH:ubiquinone oxidoreductase 24 kD subunit | | |
| COG1908 | 12 | Coenzyme F420-reducing hydrogenase, delta subunit | | |
| COG1910 | 6 | Periplasmic molybdate-binding protein/domain | | |
| COG1911 | 1 | Ribosomal protein L30E | | |
| COG1912 | 29 | Uncharacterized conserved protein | | |
| COG1913 | 7 | Predicted Zn-dependent proteases | | |
| COG1914 | 49 | Mn2+ and Fe2+ transporters of the NRAMP family | | |
| COG1915 | 10 | Uncharacterized conserved protein | | |
| COG1916 | 2 | Uncharacterized homolog of PrgY (pheromone shutdown protein) | | |
| COG1917 | 29 | Uncharacterized conserved protein, contains double-stranded beta-helix domain | | |
| COG1918 | 2 | Fe2+ transport system protein A | | |
| COG1920 | 5 | Uncharacterized conserved protein | | |
| COG1921 | 35 | Selenocysteine synthase [seryl-tRNASer selenium transferase] | | |
| COG1922 | 15 | Teichoic acid biosynthesis proteins | | |
| COG1923 | 6 | Uncharacterized host factor I protein | | |
| COG1924 | 34 | Activator of 2-hydroxyglutaryl-CoA dehydratase (HSP70-class ATPase domain) | | |
| COG1925 | 8 | Phosphotransferase system, HPr-related proteins | | |
| COG1926 | 31 | Predicted phosphoribosyltransferases | | |
| COG1928 | 6 | Dolichyl-phosphate-mannose--protein O-mannosyl transferase | | |
| COG1929 | 12 | Glycerate kinase | | |
| COG1932 | 17 | Phosphoserine aminotransferase | | |
| COG1933 | 1 | Archaeal DNA polymerase II, large subunit | | |
| COG1934 | 8 | Uncharacterized protein conserved in bacteria | | |
| COG1937 | 8 | Uncharacterized protein conserved in bacteria | | |
| COG1940 | 76 | Transcriptional regulator/sugar kinase | | |
| COG1941 | 17 | Coenzyme F420-reducing hydrogenase, gamma subunit | | |
| COG1942 | 5 | Uncharacterized protein, 4-oxalocrotonate tautomerase homolog | | |
| COG1943 | 39 | Transposase and inactivated derivatives | | |
| COG1945 | 5 | Uncharacterized conserved protein | | |
| COG1946 | 12 | Acyl-CoA thioesterase | | |
| COG1947 | 22 | 4-diphosphocytidyl-2C-methyl-D-erythritol 2-phosphate synthase | | |
| COG1949 | 10 | Oligoribonuclease (3'->5' exoribonuclease) | | |
| COG1950 | 5 | Predicted membrane protein | | |
| COG1951 | 17 | Tartrate dehydratase alpha subunit/Fumarate hydratase class I, N-terminal domain | | |
| COG1952 | 7 | Preprotein translocase subunit SecB | | |
| COG1953 | 10 | Cytosine/uracil/thiamine/allantoin permeases | | |
| COG1956 | 7 | GAF domain-containing protein | | |
| COG1957 | 11 | Inosine-uridine nucleoside N-ribohydrolase | | |
| COG1959 | 37 | Predicted transcriptional regulator | | |
| COG1960 | 474 | Acyl-CoA dehydrogenases | | |
| COG1961 | 26 | Site-specific recombinases, DNA invertase Pin homologs | | |
| COG1962 | 1 | Tetrahydromethanopterin S-methyltransferase, subunit H | | |
| COG1963 | 4 | Uncharacterized protein conserved in bacteria | | |
| COG1964 | 18 | Predicted Fe-S oxidoreductases | | |
| COG1965 | 1 | Protein implicated in iron transport, frataxin homolog | | |
| COG1966 | 25 | Carbon starvation protein, predicted membrane protein | | |
| COG1968 | 36 | Uncharacterized bacitracin resistance protein | | |
| COG1969 | 8 | Ni,Fe-hydrogenase I cytochrome b subunit | | |
| COG1970 | 15 | Large-conductance mechanosensitive channel | | |
| COG1971 | 2 | Predicted membrane protein | | |
| COG1972 | 17 | Nucleoside permease | | |
| COG1974 | 28 | SOS-response transcriptional repressors (RecA-mediated autopeptidases) | | |
| COG1975 | 44 | Xanthine and CO dehydrogenases maturation factor, XdhC/CoxF family | | |
| COG1977 | 10 | Molybdopterin converting factor, small subunit | | |
| COG1979 | 1 | Uncharacterized oxidoreductases, Fe-dependent alcohol dehydrogenase family | | |
| COG1980 | 8 | Archaeal fructose 1,6-bisphosphatase | | |
| COG1981 | 5 | Predicted membrane protein | | |
| COG1982 | 36 | Arginine/lysine/ornithine decarboxylases | | |
| COG1983 | 9 | Putative stress-responsive transcriptional regulator | | |
| COG1984 | 10 | Allophanate hydrolase subunit 2 | | |
| COG1985 | 27 | Pyrimidine reductase, riboflavin biosynthesis | | |
| COG1988 | 1 | Predicted membrane-bound metal-dependent hydrolases | | |
| COG1989 | 24 | Type II secretory pathway, prepilin signal peptidase PulO and related peptidases | | |

| | | | | |
|---|---|---|---|---|
| COG1990 | 3 | Uncharacterized conserved protein | | |
| COG1993 | 20 | Uncharacterized conserved protein | | |
| COG1994 | 29 | Zn-dependent proteases | | |
| COG1995 | 31 | Pyridoxal phosphate biosynthesis protein | | |
| COG1997 | 1 | Ribosomal protein L37AE/L43A | | |
| COG1999 | 47 | Uncharacterized protein SCO1/SenC/PrrC, involved in biogenesis of respiratory and photosynthetic systems | | |
| COG2000 | 1 | Predicted Fe-S protein | | |
| COG2001 | 24 | Uncharacterized protein conserved in bacteria | | |
| COG2002 | 3 | Regulators of stationary/sporulation gene expression | | |
| COG2003 | 28 | DNA repair proteins | | |
| COG2004 | 1 | Ribosomal protein S24E | | |
| COG2005 | 8 | N-terminal domain of molybdenum-binding protein | | |
| COG2006 | 12 | Uncharacterized conserved protein | | |
| COG2007 | 3 | Ribosomal protein S8E | | |
| COG2008 | 30 | Threonine aldolase | | |
| COG2009 | 12 | Succinate dehydrogenase/fumarate reductase, cytochrome b subunit | | |
| COG2010 | 100 | Cytochrome c, mono- and diheme variants | | |
| COG2013 | 16 | Uncharacterized conserved protein | | |
| COG2015 | 3 | Alkyl sulfatase and related hydrolases | | |
| COG2017 | 23 | Galactose mutarotase and related enzymes | | |
| COG2018 | 5 | Uncharacterized distant relative of homeotic protein bithoraxoid | | |
| COG2020 | 46 | Putative protein-S-isoprenylcysteine methyltransferase | | |
| COG2021 | 30 | Homoserine acetyltransferase | | |
| COG2022 | 21 | Uncharacterized enzyme of thiazole biosynthesis | | |
| COG2025 | 29 | Electron transfer flavoprotein, alpha subunit | | |
| COG2026 | 6 | Cytotoxic translational repressor of toxin-antitoxin stability system | | |
| COG2027 | 25 | D-alanyl-D-alanine carboxypeptidase (penicillin-binding protein 4) | | |
| COG2030 | 76 | Acyl dehydratase | | |
| COG2031 | 4 | Short chain fatty acids transporter | | |
| COG2032 | 3 | Cu/Zn superoxide dismutase | | |
| COG2036 | 1 | Histones H3 and H4 | | |
| COG2037 | 6 | Formylmethanofuran:tetrahydromethanopterin formyltransferase | | |
| COG2038 | 5 | NaMN:DMB phosphoribosyltransferase | | |
| COG2039 | 3 | Pyrrolidone-carboxylate peptidase (N-terminal pyroglutamyl peptidase) | | |
| COG2040 | 12 | Homocysteine/selenocysteine methylase (S-methylmethionine-dependent) | | |
| COG2041 | 73 | Sulfite oxidase and related enzymes | | |
| COG2043 | 3 | Uncharacterized protein conserved in archaea | | |
| COG2044 | 2 | Predicted peroxiredoxins | | |
| COG2045 | 11 | Phosphosulfolactate phosphohydrolase and related enzymes | | |
| COG2046 | 16 | ATP sulfurylase (sulfate adenylyltransferase) | | |
| COG2048 | 22 | Heterodisulfide reductase, subunit B | | |
| COG2049 | 6 | Allophanate hydrolase subunit 1 | | |
| COG2050 | 52 | Uncharacterized protein, possibly involved in aromatic compounds catabolism | | |
| COG2054 | 1 | Uncharacterized archaeal kinase related to aspartokinases, uridylate kinases | | |
| COG2055 | 37 | Malate/L-lactate dehydrogenases | | |
| COG2057 | 28 | Acyl CoA:acetate/3-ketoacid CoA transferase, beta subunit | | |
| COG2059 | 47 | Chromate transport protein ChrA | | |
| COG2060 | 18 | K+-transporting ATPase, A chain | | |
| COG2062 | 12 | Phosphohistidine phosphatase SixA | | |
| COG2063 | 2 | Flagellar basal body L-ring protein | | |
| COG2064 | 16 | Flp pilus assembly protein TadC | | |
| COG2065 | 13 | Pyrimidine operon attenuation protein/uracil phosphoribosyltransferase | | |
| COG2066 | 6 | Glutaminase | | |
| COG2067 | 22 | Long-chain fatty acid transport protein | | |
| COG2068 | 12 | Uncharacterized MobA-related protein | | |
| COG2069 | 1 | CO dehydrogenase/acetyl-CoA synthase delta subunit (corrinoid Fe-S protein) | | |
| COG2070 | 67 | Dioxygenases related to 2-nitropropane dioxygenase | | |
| COG2071 | 19 | Predicted glutamine amidotransferases | | |
| COG2072 | 90 | Predicted flavoprotein involved in K+ transport | | |
| COG2073 | 1 | Cobalamin biosynthesis protein CbiG | | |
| COG2074 | 6 | 2-phosphoglycerate kinase | | |
| COG2075 | 1 | Ribosomal protein L24E | | |
| COG2076 | 4 | Membrane transporters of cations and cationic drugs | | |
| COG2077 | 6 | Peroxiredoxin | | |
| COG2078 | 9 | Uncharacterized conserved protein | | |
| COG2079 | 82 | Uncharacterized protein involved in propionate catabolism | | |
| COG2080 | 43 | Aerobic-type carbon monoxide dehydrogenase, small subunit CoxS/CutS homologs | | |
| COG2081 | 9 | Predicted flavoproteins | | |
| COG2082 | 4 | Precorrin isomerase | | |
| COG2084 | 106 | 3-hydroxyisobutyrate dehydrogenase and related beta-hydroxyacid dehydrogenases | | |
| COG2085 | 27 | Predicted dinucleotide-binding enzymes | | |
| COG2086 | 32 | Electron transfer flavoprotein, beta subunit | | |
| COG2087 | 7 | Adenosyl cobinamide kinase/adenosyl cobinamide phosphate guanylyltransferase | | |
| COG2088 | 4 | Uncharacterized protein, involved in the regulation of septum location | | |
| COG2089 | 14 | Sialic acid synthase | | |
| COG2091 | 3 | Phosphopantetheinyl transferase | | |
| COG2094 | 14 | 3-methyladenine DNA glycosylase | | |
| COG2095 | 18 | Multiple antibiotic transporter | | |
| COG2096 | 16 | Uncharacterized conserved protein | | |
| COG2097 | 1 | Ribosomal protein L31E | | |
| COG2099 | 2 | Precorrin-6x reductase | | |
| COG2102 | 3 | Predicted ATPases of PP-loop superfamily | | |
| COG2103 | 17 | Predicted sugar phosphate isomerase | | |
| COG2104 | 5 | Sulfur transfer protein involved in thiamine biosynthesis | | |

| | | | | |
|---|---|---|---|---|
| COG2107 | 18 | Predicted periplasmic solute-binding protein | | |
| COG2109 | 8 | ATP:corrinoid adenosyltransferase | | |
| COG2110 | 22 | Predicted phosphatase homologous to the C-terminal domain of histone macroH2A1 | | |
| COG2113 | 1 | ABC-type proline/glycine betaine transport systems, periplasmic components | | |
| COG2114 | 173 | Adenylate cyclase, family 3 (some proteins contain HAMP domain) | | |
| COG2115 | 9 | Xylose isomerase | | |
| COG2116 | 16 | Formate/nitrite family of transporters | | |
| COG2119 | 9 | Predicted membrane protein | | |
| COG2120 | 59 | Uncharacterized proteins, LmbE homologs | | |
| COG2121 | 5 | Uncharacterized protein conserved in bacteria | | |
| COG2122 | 1 | Uncharacterized conserved protein | | |
| COG2124 | 150 | Cytochrome P450 | | |
| COG2125 | 1 | Ribosomal protein S6E (S10) | | |
| COG2127 | 6 | Uncharacterized conserved protein | | |
| COG2128 | 38 | Uncharacterized conserved protein | | |
| COG2129 | 25 | Predicted phosphoesterases, related to the Icc protein | | |
| COG2130 | 19 | Putative NADP-dependent oxidoreductases | | |
| COG2131 | 11 | Deoxycytidylate deaminase | | |
| COG2132 | 103 | Putative multicopper oxidases | | |
| COG2133 | 66 | Glucose/sorbosone dehydrogenases | | |
| COG2135 | 14 | Uncharacterized conserved protein | | |
| COG2137 | 9 | Uncharacterized protein conserved in bacteria | | |
| COG2138 | 6 | Uncharacterized conserved protein | | |
| COG2139 | 2 | Ribosomal protein L21E | | |
| COG2140 | 3 | Thermophilic glucose-6-phosphate isomerase and related metalloenzymes | | |
| COG2141 | 256 | Coenzyme F420-dependent N5,N10-methylene tetrahydromethanopterin reductase and related flavin-dependent oxidoreductases | | |
| COG2142 | 5 | Succinate dehydrogenase, hydrophobic anchor subunit | | |
| COG2143 | 1 | Thioredoxin-related protein | | |
| COG2145 | 2 | Hydroxyethylthiazole kinase, sugar kinase family | | |
| COG2146 | 35 | Ferredoxin subunits of nitrite reductase and ring-hydroxylating dioxygenases | | |
| COG2147 | 1 | Ribosomal protein L19E | | |
| COG2148 | 42 | Sugar transferases involved in lipopolysaccharide synthesis | | |
| COG2149 | 3 | Predicted membrane protein | | |
| COG2151 | 28 | Predicted metal-sulfur cluster biosynthetic enzyme | | |
| COG2152 | 9 | Predicted glycosylase | | |
| COG2153 | 2 | Predicted acyltransferase | | |
| COG2154 | 19 | Pterin-4a-carbinolamine dehydratase | | |
| COG2155 | 1 | Uncharacterized conserved protein | | |
| COG2156 | 10 | K+-transporting ATPase, c chain | | |
| COG2159 | 131 | Predicted metal-dependent hydrolase of the TIM-barrel fold | | |
| COG2160 | 7 | L-arabinose isomerase | | |
| COG2161 | 1 | Antitoxin of toxin-antitoxin stability system | | |
| COG2162 | 10 | Arylamine N-acetyltransferase | | |
| COG2163 | 2 | Ribosomal protein L14E/L6E/L27E | | |
| COG2164 | 3 | Uncharacterized conserved protein | | |
| COG2165 | 25 | Type II secretory pathway, pseudopilin PulG | | |
| COG2166 | 3 | SufE protein probably involved in Fe-S center assembly | | |
| COG2168 | 2 | Uncharacterized conserved protein involved in oxidation of intracellular sulfur | | |
| COG2169 | 17 | Adenosine deaminase | | |
| COG2170 | 23 | Uncharacterized conserved protein | | |
| COG2171 | 18 | Tetrahydrodipicolinate N-succinyltransferase | | |
| COG2172 | 28 | Anti-sigma regulatory factor (Ser/Thr protein kinase) | | |
| COG2173 | 4 | D-alanyl-D-alanine dipeptidase | | |
| COG2175 | 45 | Probable taurine catabolism dioxygenase | | |
| COG2176 | 10 | DNA polymerase III, alpha subunit (gram-positive type) | | |
| COG2177 | 15 | Cell division protein | | |
| COG2178 | 1 | Predicted RNA-binding protein of the translin family | | |
| COG2179 | 1 | Predicted hydrolase of the HAD superfamily | | |
| COG2180 | 4 | Nitrate reductase delta subunit | | |
| COG2181 | 4 | Nitrate reductase gamma subunit | | |
| COG2182 | 6 | Maltose-binding periplasmic proteins/domains | | |
| COG2183 | 10 | Transcriptional accessory protein | | |
| COG2184 | 1 | Protein involved in cell division | | |
| COG2185 | 14 | Methylmalonyl-CoA mutase, C-terminal domain/subunit (cobalamin-binding) | | |
| COG2186 | 22 | Transcriptional regulators | | |
| COG2187 | 14 | Uncharacterized protein conserved in bacteria | | |
| COG2188 | 31 | Transcriptional regulators | | |
| COG2189 | 16 | Adenine specific DNA methylase Mod | | |
| COG2190 | 3 | Phosphotransferase system IIA components | | |
| COG2191 | 3 | Formylmethanofuran dehydrogenase subunit E | | |
| COG2192 | 37 | Predicted carbamoyl transferase, NodU family | | |
| COG2193 | 19 | Bacterioferritin (cytochrome b1) | | |
| COG2194 | 1 | Predicted membrane-associated, metal-dependent hydrolase | | |
| COG2195 | 34 | Di- and tripeptidases | | |
| COG2197 | 245 | Response regulator containing a CheY-like receiver domain and an HTH DNA-binding domain | | |
| COG2198 | 4 | FOG: HPt domain | | |
| COG2199 | 189 | FOG: GGDEF domain | | |
| COG2200 | 127 | FOG: EAL domain | | |
| COG2201 | 63 | Chemotaxis response regulator containing a CheY-like receiver domain and a methylesterase domain | | |
| COG2202 | 390 | FOG: PAS/PAC domain | | |
| COG2203 | 147 | FOG: GAF domain | | |
| COG2204 | 282 | Response regulator containing CheY-like receiver, AAA-type ATPase, and DNA-binding domains | | |
| COG2205 | 54 | Osmosensitive K+ channel histidine kinase | | |
| COG2206 | 101 | HD-GYP domain | | |

| | | | | |
|---|---|---|---|---|
| COG2207 | 57 | AraC-type DNA-binding domain-containing proteins | | |
| COG2208 | 77 | Serine phosphatase RsbU, regulator of sigma subunit | | |
| COG2209 | 1 | Na+-transporting NADH:ubiquinone oxidoreductase, subunit NqrE | | |
| COG2210 | 12 | Uncharacterized conserved protein | | |
| COG2211 | 12 | Na+/melibiose symporter and related transporters | | |
| COG2212 | 1 | Multisubunit Na+/H+ antiporter, MnhF subunit | | |
| COG2213 | 3 | Phosphotransferase system, mannitol-specific IIBC component | | |
| COG2214 | 23 | DnaJ-class molecular chaperone | | |
| COG2215 | 5 | ABC-type uncharacterized transport system, permease component | | |
| COG2216 | 19 | High-affinity K+ transport system, ATPase chain B | | |
| COG2217 | 145 | Cation transport ATPase | | |
| COG2220 | 63 | Predicted Zn-dependent hydrolases of the beta-lactamase fold | | |
| COG2221 | 7 | Dissimilatory sulfite reductase (desulfoviridin), alpha and beta subunits | | |
| COG2222 | 15 | Predicted phosphosugar isomerases | | |
| COG2223 | 27 | Nitrate/nitrite transporter | | |
| COG2224 | 12 | Isocitrate lyase | | |
| COG2225 | 22 | Malate synthase | | |
| COG2226 | 49 | Methylase involved in ubiquinone/menaquinone biosynthesis | | |
| COG2227 | 20 | 2-polyprenyl-3-methyl-5-hydroxy-6-metoxy-1,4-benzoquinol methylase | | |
| COG2230 | 41 | Cyclopropane fatty acid synthase and related methyltransferases | | |
| COG2231 | 3 | Uncharacterized protein related to Endonuclease III | | |
| COG2232 | 3 | Predicted ATP-dependent carboligase related to biotin carboxylase | | |
| COG2233 | 17 | Xanthine/uracil permeases | | |
| COG2234 | 110 | Predicted aminopeptidases | | |
| COG2235 | 10 | Arginine deiminase | | |
| COG2236 | 4 | Predicted phosphoribosyltransferases | | |
| COG2239 | 42 | Mg/Co/Ni transporter MgtE (contains CBS domain) | | |
| COG2240 | 1 | Pyridoxal/pyridoxine/pyridoxamine kinase | | |
| COG2241 | 4 | Precorrin-6B methylase 1 | | |
| COG2242 | 2 | Precorrin-6B methylase 2 | | |
| COG2243 | 1 | Precorrin-2 methylase | | |
| COG2244 | 30 | Membrane protein involved in the export of O-antigen and teichoic acid | | |
| COG2246 | 11 | Predicted membrane protein | | |
| COG2249 | 7 | Putative NADPH-quinone reductase (modulator of drug activity B) | | |
| COG2251 | 8 | Predicted nuclease (RecB family) | | |
| COG2252 | 24 | Permeases | | |
| COG2253 | 1 | Uncharacterized conserved protein | | |
| COG2255 | 31 | Holliday junction resolvasome, helicase subunit | | |
| COG2256 | 29 | ATPase related to the helicase subunit of the Holliday junction resolvase | | |
| COG2257 | 1 | Uncharacterized homolog of the cytoplasmic domain of flagellar protein FhlB | | |
| COG2258 | 11 | Uncharacterized protein conserved in bacteria | | |
| COG2259 | 35 | Predicted membrane protein | | |
| COG2261 | 8 | Predicted membrane protein | | |
| COG2262 | 24 | GTPases | | |
| COG2263 | 1 | Predicted RNA methylase | | |
| COG2264 | 21 | Ribosomal protein L11 methylase | | |
| COG2265 | 48 | SAM-dependent methyltransferases related to tRNA (uracil-5-)-methyltransferase | | |
| COG2267 | 17 | Lysophospholipase | | |
| COG2268 | 17 | Uncharacterized protein conserved in bacteria | | |
| COG2269 | 4 | Truncated, possibly inactive, lysyl-tRNA synthetase (class II) | | |
| COG2270 | 19 | Permeases of the major facilitator superfamily | | |
| COG2271 | 3 | Sugar phosphate permease | | |
| COG2272 | 16 | Carboxylesterase type B | | |
| COG2273 | 13 | Beta-glucanase/Beta-glucan synthetase | | |
| COG2274 | 26 | ABC-type bacteriocin/lantibiotic exporters, contain an N-terminal double-glycine peptidase domain | | |
| COG2301 | 34 | Citrate lyase beta subunit | | |
| COG2303 | 190 | Choline dehydrogenase and related flavoproteins | | |
| COG2304 | 61 | Uncharacterized protein containing a von Willebrand factor type A (vWA) domain | | |
| COG2306 | 2 | Uncharacterized conserved protein | | |
| COG2307 | 13 | Uncharacterized protein conserved in bacteria | | |
| COG2308 | 19 | Uncharacterized conserved protein | | |
| COG2309 | 39 | Leucyl aminopeptidase (aminopeptidase T) | | |
| COG2311 | 3 | Predicted membrane protein | | |
| COG2312 | 11 | Erythromycin esterase homolog | | |
| COG2313 | 13 | Uncharacterized enzyme involved in pigment biosynthesis | | |
| COG2314 | 3 | Predicted membrane protein | | |
| COG2315 | 2 | Uncharacterized protein conserved in bacteria | | |
| COG2316 | 7 | Predicted hydrolase (HD superfamily) | | |
| COG2317 | 20 | Zn-dependent carboxypeptidase | | |
| COG2318 | 13 | Uncharacterized protein conserved in bacteria | | |
| COG2319 | 81 | FOG: WD40 repeat | | |
| COG2320 | 4 | Uncharacterized conserved protein | | |
| COG2321 | 12 | Predicted metalloprotease | | |
| COG2323 | 13 | Predicted membrane protein | | |
| COG2324 | 3 | Predicted membrane protein | | |
| COG2326 | 31 | Uncharacterized conserved protein | | |
| COG2327 | 2 | Uncharacterized conserved protein | | |
| COG2329 | 9 | Uncharacterized enzyme involved in biosynthesis of extracellular polysaccharides | | |
| COG2331 | 8 | Uncharacterized protein conserved in bacteria | | |
| COG2332 | 10 | Cytochrome c-type biogenesis protein CcmE | | |
| COG2333 | 29 | Predicted hydrolase (metallo-beta-lactamase superfamily) | | |
| COG2334 | 15 | Putative homoserine kinase type II (protein kinase fold) | | |
| COG2335 | 10 | Secreted and surface protein containing fasciclin-like repeats | | |
| COG2336 | 1 | Growth regulator | | |

| | | | | |
|---|---|---|---|---|
| COG2337 | 5 | Growth inhibitor | | |
| COG2339 | 4 | Predicted membrane protein | | |
| COG2340 | 14 | Uncharacterized protein with SCP/PR1 domains | | |
| COG2343 | 15 | Uncharacterized protein conserved in bacteria | | |
| COG2344 | 7 | AT-rich DNA-binding protein | | |
| COG2345 | 16 | Predicted transcriptional regulator | | |
| COG2346 | 12 | Truncated hemoglobins | | |
| COG2348 | 5 | Uncharacterized protein involved in methicillin resistance | | |
| COG2350 | 1 | Uncharacterized protein conserved in bacteria | | |
| COG2351 | 5 | Transthyretin-like protein | | |
| COG2352 | 39 | Phosphoenolpyruvate carboxylase | | |
| COG2353 | 47 | Uncharacterized conserved protein | | |
| COG2354 | 2 | Uncharacterized protein conserved in bacteria | | |
| COG2355 | 44 | Zn-dependent dipeptidase, microsomal dipeptidase homolog | | |
| COG2356 | 2 | Endonuclease I | | |
| COG2358 | 41 | TRAP-type uncharacterized transport system, periplasmic component | | |
| COG2360 | 12 | Leu/Phe-tRNA-protein transferase | | |
| COG2361 | 2 | Uncharacterized conserved protein | | |
| COG2362 | 13 | D-aminopeptidase | | |
| COG2363 | 5 | Uncharacterized small membrane protein | | |
| COG2364 | 4 | Predicted membrane protein | | |
| COG2365 | 4 | Protein tyrosine/serine phosphatase | | |
| COG2366 | 78 | Protein related to penicillin acylase | | |
| COG2367 | 14 | Beta-lactamase class A | | |
| COG2368 | 31 | Aromatic ring hydroxylase | | |
| COG2369 | 1 | Uncharacterized protein, homolog of phage Mu protein gp30 | | |
| COG2370 | 1 | Hydrogenase/urease accessory protein | | |
| COG2371 | 2 | Urease accessory protein UreE | | |
| COG2372 | 4 | Uncharacterized protein, homolog of Cu resistance protein CopC | | |
| COG2373 | 31 | Large extracellular alpha-helical protein | | |
| COG2374 | 15 | Predicted extracellular nuclease | | |
| COG2375 | 2 | Siderophore-interacting protein | | |
| COG2376 | 13 | Dihydroxyacetone kinase | | |
| COG2377 | 23 | Predicted molecular chaperone distantly related to HSP70-fold metalloproteases | | |
| COG2378 | 24 | Predicted transcriptional regulator | | |
| COG2379 | 29 | Putative glycerate kinase | | |
| COG2382 | 7 | Enterochelin esterase and related enzymes | | |
| COG2385 | 22 | Sporulation protein and related proteins | | |
| COG2386 | 15 | ABC-type transport system involved in cytochrome c biogenesis, permease component | | |
| COG2388 | 3 | Predicted acetyltransferase | | |
| COG2389 | 1 | Uncharacterized metal-binding protein | | |
| COG2390 | 1 | Transcriptional regulator, contains sigma factor-related N-terminal domain | | |
| COG2391 | 17 | Predicted transporter component | | |
| COG2402 | 3 | Predicted nucleic acid-binding protein, contains PIN domain | | |
| COG2403 | 14 | Predicted GTPase | | |
| COG2407 | 10 | L-fucose isomerase and related proteins | | |
| COG2409 | 80 | Predicted drug exporters of the RND superfamily | | |
| COG2411 | 1 | Uncharacterized conserved protein | | |
| COG2414 | 69 | Aldehyde:ferredoxin oxidoreductase | | |
| COG2421 | 36 | Predicted acetamidase/formamidase | | |
| COG2423 | 39 | Predicted ornithine cyclodeaminase, mu-crystallin homolog | | |
| COG2425 | 3 | Uncharacterized protein containing a von Willebrand factor type A (vWA) domain | | |
| COG2442 | 12 | Uncharacterized conserved protein | | |
| COG2445 | 1 | Uncharacterized conserved protein | | |
| COG2452 | 3 | Predicted site-specific integrase-resolvase | | |
| COG2453 | 3 | Predicted protein-tyrosine phosphatase | | |
| COG2461 | 3 | Uncharacterized conserved protein | | |
| COG2502 | 6 | Asparagine synthetase A | | |
| COG2503 | 2 | Predicted secreted acid phosphatase | | |
| COG2508 | 16 | Regulator of polyketide synthase expression | | |
| COG2509 | 10 | Uncharacterized FAD-dependent dehydrogenases | | |
| COG2513 | 56 | PEP phosphonomutase and related enzymes | | |
| COG2514 | 17 | Predicted ring-cleavage extradiol dioxygenase | | |
| COG2515 | 12 | 1-aminocyclopropane-1-carboxylate deaminase | | |
| COG2516 | 5 | Biotin synthase-related enzyme | | |
| COG2518 | 39 | Protein-L-isoaspartate carboxylmethyltransferase | | |
| COG2519 | 6 | tRNA(1-methyladenosine) methyltransferase and related methyltransferases | | |
| COG2521 | 1 | Predicted archaeal methyltransferase | | |
| COG2602 | 1 | Beta-lactamase class D | | |
| COG2603 | 3 | Predicted ATPase | | |
| COG2605 | 8 | Predicted kinase related to galactokinase and mevalonate kinase | | |
| COG2606 | 27 | Uncharacterized conserved protein | | |
| COG2607 | 9 | Predicted ATPase (AAA+ superfamily) | | |
| COG2608 | 8 | Copper chaperone | | |
| COG2609 | 43 | Pyruvate dehydrogenase complex, dehydrogenase (E1) component | | |
| COG2610 | 7 | H+/gluconate symporter and related permeases | | |
| COG2703 | 11 | Hemerythrin | | |
| COG2704 | 4 | Anaerobic C4-dicarboxylate transporter | | |
| COG2706 | 9 | 3-carboxymuconate cyclase | | |
| COG2710 | 6 | Nitrogenase molybdenum-iron protein, alpha and beta chains | | |
| COG2715 | 6 | Uncharacterized membrane protein, required for spore maturation in B.subtilis. | | |
| COG2716 | 1 | Glycine cleavage system regulatory protein | | |
| COG2717 | 7 | Predicted membrane protein | | |
| COG2718 | 7 | Uncharacterized conserved protein | | |

| | | | | |
|---|---|---|---|---|
| COG2719 | 10 | Uncharacterized conserved protein | | |
| COG2720 | 11 | Uncharacterized vancomycin resistance protein | | |
| COG2721 | 27 | Altronate dehydratase | | |
| COG2723 | 34 | Beta-glucosidase/6-phospho-beta-glucosidase/beta-galactosidase | | |
| COG2730 | 8 | Endoglucanase | | |
| COG2733 | 9 | Predicted membrane protein | | |
| COG2738 | 3 | Predicted Zn-dependent protease | | |
| COG2739 | 1 | Uncharacterized protein conserved in bacteria | | |
| COG2740 | 5 | Predicted nucleic-acid-binding protein implicated in transcription termination | | |
| COG2746 | 3 | Aminoglycoside N3'-acetyltransferase | | |
| COG2755 | 28 | Lysophospholipase L1 and related esterases | | |
| COG2759 | 22 | Formyltetrahydrofolate synthetase | | |
| COG2761 | 14 | Predicted dithiol-disulfide isomerase involved in polyketide biosynthesis | | |
| COG2764 | 25 | Uncharacterized protein conserved in bacteria | | |
| COG2766 | 31 | Putative Ser protein kinase | | |
| COG2768 | 3 | Uncharacterized Fe-S center protein | | |
| COG2770 | 23 | FOG: HAMP domain | | |
| COG2771 | 54 | DNA-binding HTH domain-containing proteins | | |
| COG2801 | 230 | Transposase and inactivated derivatives | | |
| COG2802 | 11 | Uncharacterized protein, similar to the N-terminal domain of Lon protease | | |
| COG2804 | 84 | Type II secretory pathway, ATPase PulE/Tfp pilus assembly pathway, ATPase PilB | | |
| COG2805 | 62 | Tfp pilus assembly protein, pilus retraction ATPase PilT | | |
| COG2807 | 6 | Cyanate permease | | |
| COG2808 | 8 | Transcriptional regulator | | |
| COG2812 | 40 | DNA polymerase III, gamma/tau subunits | | |
| COG2813 | 2 | 16S RNA G1207 methylase RsmC | | |
| COG2814 | 6 | Arabinose efflux permease | | |
| COG2815 | 14 | Uncharacterized protein conserved in bacteria | | |
| COG2816 | 13 | NTP pyrophosphohydrolases containing a Zn-finger, probably nucleic-acid-binding | | |
| COG2818 | 10 | 3-methyladenine DNA glycosylase | | |
| COG2819 | 5 | Predicted hydrolase of the alpha/beta superfamily | | |
| COG2820 | 3 | Uridine phosphorylase | | |
| COG2821 | 13 | Membrane-bound lytic murein transglycosylase | | |
| COG2822 | 2 | Predicted periplasmic lipoprotein involved in iron transport | | |
| COG2823 | 18 | Predicted periplasmic or secreted lipoprotein | | |
| COG2825 | 4 | Outer membrane protein | | |
| COG2826 | 17 | Transposase and inactivated derivatives, IS30 family | | |
| COG2827 | 8 | Predicted endonuclease containing a URI domain | | |
| COG2828 | 9 | Uncharacterized protein conserved in bacteria | | |
| COG2829 | 8 | Outer membrane phospholipase A | | |
| COG2831 | 20 | Hemolysin activation/secretion protein | | |
| COG2832 | 1 | Uncharacterized protein conserved in bacteria | | |
| COG2833 | 1 | Uncharacterized protein conserved in bacteria | | |
| COG2834 | 11 | Outer membrane lipoprotein-sorting protein | | |
| COG2835 | 2 | Uncharacterized conserved protein | | |
| COG2836 | 7 | Uncharacterized conserved protein | | |
| COG2837 | 17 | Predicted iron-dependent peroxidase | | |
| COG2838 | 10 | Monomeric isocitrate dehydrogenase | | |
| COG2840 | 4 | Uncharacterized protein conserved in bacteria | | |
| COG2843 | 26 | Putative enzyme of poly-gamma-glutamate biosynthesis (capsule formation) | | |
| COG2844 | 45 | UTP:GlnB (protein PII) uridylyltransferase | | |
| COG2845 | 1 | Uncharacterized protein conserved in bacteria | | |
| COG2846 | 4 | Regulator of cell morphogenesis and NO signaling | | |
| COG2847 | 6 | Uncharacterized protein conserved in bacteria | | |
| COG2848 | 3 | Uncharacterized conserved protein | | |
| COG2849 | 4 | Uncharacterized protein conserved in bacteria | | |
| COG2850 | 10 | Uncharacterized conserved protein | | |
| COG2852 | 3 | Uncharacterized protein conserved in bacteria | | |
| COG2853 | 6 | Surface lipoprotein | | |
| COG2854 | 15 | ABC-type transport system involved in resistance to organic solvents, auxiliary component | | |
| COG2855 | 8 | Predicted membrane protein | | |
| COG2856 | 3 | Predicted Zn peptidase | | |
| COG2857 | 13 | Cytochrome c1 | | |
| COG2860 | 12 | Predicted membrane protein | | |
| COG2861 | 2 | Uncharacterized protein conserved in bacteria | | |
| COG2862 | 3 | Predicted membrane protein | | |
| COG2863 | 61 | Cytochrome c553 | | |
| COG2864 | 16 | Cytochrome b subunit of formate dehydrogenase | | |
| COG2865 | 4 | Predicted transcriptional regulator containing an HTH domain and an uncharacterized domain shared with the mammalian protein Schlafe | | |
| COG2866 | 20 | Predicted carboxypeptidase | | |
| COG2867 | 3 | Oligoketide cyclase/lipid transport protein | | |
| COG2869 | 2 | Na+-transporting NADH:ubiquinone oxidoreductase, subunit NqrC | | |
| COG2870 | 43 | ADP-heptose synthase, bifunctional sugar kinase/adenylyltransferase | | |
| COG2871 | 3 | Na+-transporting NADH:ubiquinone oxidoreductase, subunit NqrF | | |
| COG2872 | 14 | Predicted metal-dependent hydrolases related to alanyl-tRNA synthetase HxxxH domain | | |
| COG2873 | 29 | O-acetylhomoserine sulfhydrylase | | |
| COG2875 | 4 | Precorrin-4 methylase | | |
| COG2876 | 30 | 3-deoxy-D-arabino-heptulosonate 7-phosphate (DAHP) synthase | | |
| COG2877 | 19 | 3-deoxy-D-manno-octulosonic acid (KDO) 8-phosphate synthase | | |
| COG2878 | 6 | Predicted NADH:ubiquinone oxidoreductase, subunit RnfB | | |
| COG2884 | 8 | Predicted ATPase involved in cell division | | |
| COG2885 | 71 | Outer membrane protein and related peptidoglycan-associated (lipo)proteins | | |
| COG2887 | 16 | RecB family exonuclease | | |
| COG2890 | 36 | Methylase of polypeptide chain release factors | | |

| | | | | |
|---|---|---|---|---|
| COG2891 | 3 | Cell shape-determining protein | | |
| COG2893 | 10 | Phosphotransferase system, mannose/fructose-specific component IIA | | |
| COG2894 | 5 | Septum formation inhibitor-activating ATPase | | |
| COG2895 | 28 | GTPases - Sulfate adenylate transferase subunit 1 | | |
| COG2896 | 28 | Molybdenum cofactor biosynthesis enzyme | | |
| COG2897 | 38 | Rhodanese-related sulfurtransferase | | |
| COG2898 | 28 | Uncharacterized conserved protein | | |
| COG2899 | 2 | Uncharacterized protein conserved in bacteria | | |
| COG2901 | 1 | Factor for inversion stimulation Fis, transcriptional activator | | |
| COG2902 | 36 | NAD-specific glutamate dehydrogenase | | |
| COG2905 | 26 | Predicted signal-transduction protein containing cAMP-binding and CBS domains | | |
| COG2907 | 16 | Predicted NAD/FAD-binding protein | | |
| COG2908 | 9 | Uncharacterized protein conserved in bacteria | | |
| COG2909 | 49 | ATP-dependent transcriptional regulator | | |
| COG2910 | 1 | Putative NADH-flavin reductase | | |
| COG2911 | 9 | Uncharacterized protein conserved in bacteria | | |
| COG2912 | 18 | Uncharacterized conserved protein | | |
| COG2913 | 6 | Small protein A (tmRNA-binding) | | |
| COG2914 | 2 | Uncharacterized protein conserved in bacteria | | |
| COG2917 | 8 | Intracellular septation protein A | | |
| COG2918 | 6 | Gamma-glutamylcysteine synthetase | | |
| COG2919 | 3 | Septum formation initiator | | |
| COG2920 | 14 | Dissimilatory sulfite reductase (desulfoviridin), gamma subunit | | |
| COG2921 | 2 | Uncharacterized conserved protein | | |
| COG2922 | 1 | Uncharacterized protein conserved in bacteria | | |
| COG2923 | 1 | Uncharacterized protein involved in the oxidation of intracellular sulfur | | |
| COG2924 | 3 | Fe-S cluster protector protein | | |
| COG2927 | 6 | DNA polymerase III, chi subunit | | |
| COG2928 | 8 | Uncharacterized conserved protein | | |
| COG2929 | 7 | Uncharacterized protein conserved in bacteria | | |
| COG2930 | 11 | Uncharacterized conserved protein | | |
| COG2931 | 80 | RTX toxins and related Ca2+-binding proteins | | |
| COG2933 | 3 | Predicted SAM-dependent methyltransferase | | |
| COG2935 | 7 | Putative arginyl-tRNA:protein arginylyltransferase | | |
| COG2936 | 97 | Predicted acyl esterases | | |
| COG2937 | 12 | Glycerol-3-phosphate O-acyltransferase | | |
| COG2938 | 2 | Uncharacterized conserved protein | | |
| COG2939 | 19 | Carboxypeptidase C (cathepsin A) | | |
| COG2940 | 6 | Proteins containing SET domain | | |
| COG2941 | 3 | Ubiquinone biosynthesis protein COQ7 | | |
| COG2942 | 3 | N-acyl-D-glucosamine 2-epimerase | | |
| COG2943 | 4 | Membrane glycosyltransferase | | |
| COG2944 | 2 | Predicted transcriptional regulator | | |
| COG2945 | 12 | Predicted hydrolase of the alpha/beta superfamily | | |
| COG2946 | 1 | Putative phage replication protein RstA | | |
| COG2947 | 12 | Uncharacterized conserved protein | | |
| COG2949 | 1 | Uncharacterized membrane protein | | |
| COG2951 | 21 | Membrane-bound lytic murein transglycosylase B | | |
| COG2954 | 2 | Uncharacterized protein conserved in bacteria | | |
| COG2956 | 9 | Predicted N-acetylglucosaminyl transferase | | |
| COG2957 | 12 | Peptidylarginine deiminase and related enzymes | | |
| COG2959 | 3 | Uncharacterized enzyme of heme biosynthesis | | |
| COG2960 | 1 | Uncharacterized protein conserved in bacteria | | |
| COG2961 | 2 | Protein involved in catabolism of external DNA | | |
| COG2962 | 10 | Predicted permeases | | |
| COG2963 | 18 | Transposase and inactivated derivatives | | |
| COG2965 | 4 | Primosomal replication protein N | | |
| COG2966 | 5 | Uncharacterized conserved protein | | |
| COG2967 | 4 | Uncharacterized protein affecting Mg2+/Co2+ transport | | |
| COG2968 | 9 | Uncharacterized conserved protein | | |
| COG2969 | 4 | Stringent starvation protein B | | |
| COG2971 | 7 | Predicted N-acetylglucosamine kinase | | |
| COG2972 | 13 | Predicted signal transduction protein with a C-terminal ATPase domain | | |
| COG2975 | 1 | Uncharacterized protein conserved in bacteria | | |
| COG2976 | 4 | Uncharacterized protein conserved in bacteria | | |
| COG2977 | 2 | Phosphopantetheinyl transferase component of siderophore synthetase | | |
| COG2978 | 2 | Putative p-aminobenzoyl-glutamate transporter | | |
| COG2980 | 5 | Rare lipoprotein B | | |
| COG2982 | 17 | Uncharacterized protein involved in outer membrane biogenesis | | |
| COG2983 | 3 | Uncharacterized conserved protein | | |
| COG2984 | 46 | ABC-type uncharacterized transport system, periplasmic component | | |
| COG2985 | 4 | Predicted permease | | |
| COG2986 | 31 | Histidine ammonia-lyase | | |
| COG2987 | 17 | Urocanate hydratase | | |
| COG2989 | 20 | Uncharacterized protein conserved in bacteria | | |
| COG2993 | 10 | Cbb3-type cytochrome oxidase, cytochrome c subunit | | |
| COG2995 | 5 | Uncharacterized paraquat-inducible protein A | | |
| COG2998 | 8 | ABC-type tungstate transport system, permease component | | |
| COG3000 | 25 | Sterol desaturase | | |
| COG3001 | 4 | Fructosamine-3-kinase | | |
| COG3002 | 6 | Uncharacterized protein conserved in bacteria | | |
| COG3004 | 15 | Na+/H+ antiporter | | |
| COG3005 | 9 | Nitrate/TMAO reductases, membrane-bound tetraheme cytochrome c subunit | | |
| COG3008 | 8 | Paraquat-inducible protein B | | |

| | | | | |
|---|---|---|---|---|
| COG3011 | 1 | Uncharacterized protein conserved in bacteria | | |
| COG3012 | 1 | Uncharacterized protein conserved in bacteria | | |
| COG3014 | 1 | Uncharacterized protein conserved in bacteria | | |
| COG3016 | 6 | Uncharacterized iron-regulated protein | | |
| COG3017 | 3 | Outer membrane lipoprotein involved in outer membrane biogenesis | | |
| COG3019 | 7 | Predicted metal-binding protein | | |
| COG3021 | 1 | Uncharacterized protein conserved in bacteria | | |
| COG3023 | 9 | Negative regulator of beta-lactamase expression | | |
| COG3024 | 2 | Uncharacterized protein conserved in bacteria | | |
| COG3025 | 5 | Uncharacterized conserved protein | | |
| COG3026 | 9 | Negative regulator of sigma E activity | | |
| COG3027 | 7 | Uncharacterized protein conserved in bacteria | | |
| COG3028 | 2 | Uncharacterized protein conserved in bacteria | | |
| COG3029 | 5 | Fumarate reductase subunit C | | |
| COG3030 | 5 | Protein affecting phage T7 exclusion by the F plasmid | | |
| COG3031 | 1 | Type II secretory pathway, component PulC | | |
| COG3033 | 15 | Tryptophanase | | |
| COG3034 | 10 | Uncharacterized protein conserved in bacteria | | |
| COG3037 | 1 | Uncharacterized protein conserved in bacteria | | |
| COG3038 | 4 | Cytochrome B561 | | |
| COG3039 | 19 | Transposase and inactivated derivatives, IS5 family | | |
| COG3040 | 2 | Bacterial lipocalin | | |
| COG3041 | 1 | Uncharacterized protein conserved in bacteria | | |
| COG3043 | 6 | Nitrate reductase cytochrome c-type subunit | | |
| COG3046 | 2 | Uncharacterized protein related to deoxyribodipyrimidine photolyase | | |
| COG3051 | 1 | Citrate lyase, alpha subunit | | |
| COG3058 | 5 | Uncharacterized protein involved in formate dehydrogenase formation | | |
| COG3063 | 9 | Tfp pilus assembly protein PilF | | |
| COG3065 | 2 | Starvation-inducible outer membrane lipoprotein | | |
| COG3069 | 1 | C4-dicarboxylate transporter | | |
| COG3070 | 3 | Regulator of competence-specific genes | | |
| COG3071 | 3 | Uncharacterized enzyme of heme biosynthesis | | |
| COG3073 | 1 | Negative regulator of sigma E activity | | |
| COG3075 | 1 | Anaerobic glycerol-3-phosphate dehydrogenase | | |
| COG3080 | 5 | Fumarate reductase subunit D | | |
| COG3081 | 1 | Nucleoid-associated protein | | |
| COG3083 | 4 | Predicted hydrolase of alkaline phosphatase superfamily | | |
| COG3087 | 1 | Cell division protein | | |
| COG3088 | 11 | Uncharacterized protein involved in biosynthesis of c-type cytochromes | | |
| COG3090 | 28 | TRAP-type C4-dicarboxylate transport system, small permease component | | |
| COG3093 | 11 | Plasmid maintenance system antidote protein | | |
| COG3094 | 2 | Uncharacterized protein conserved in bacteria | | |
| COG3103 | 1 | SH3 domain protein | | |
| COG3104 | 27 | Dipeptide/tripeptide permease | | |
| COG3107 | 4 | Putative lipoprotein | | |
| COG3108 | 4 | Uncharacterized protein conserved in bacteria | | |
| COG3113 | 1 | Predicted NTP binding protein (contains STAS domain) | | |
| COG3116 | 1 | Cell division protein | | |
| COG3117 | 2 | Uncharacterized protein conserved in bacteria | | |
| COG3118 | 10 | Thioredoxin domain-containing protein | | |
| COG3119 | 117 | Arylsulfatase A and related enzymes | | |
| COG3121 | 4 | P pilus assembly protein, chaperone PapD | | |
| COG3123 | 1 | Uncharacterized protein conserved in bacteria | | |
| COG3124 | 1 | Uncharacterized protein conserved in bacteria | | |
| COG3126 | 2 | Uncharacterized protein conserved in bacteria | | |
| COG3127 | 19 | Predicted ABC-type transport system involved in lysophospholipase L1 biosynthesis, permease component | | |
| COG3128 | 1 | Uncharacterized iron-regulated protein | | |
| COG3131 | 3 | Periplasmic glucans biosynthesis protein | | |
| COG3132 | 12 | Uncharacterized protein conserved in bacteria | | |
| COG3134 | 1 | Predicted outer membrane lipoprotein | | |
| COG3135 | 6 | Uncharacterized protein involved in benzoate metabolism | | |
| COG3137 | 1 | Putative salt-induced outer membrane protein | | |
| COG3138 | 3 | Arginine/ornithine N-succinyltransferase beta subunit | | |
| COG3142 | 6 | Uncharacterized protein involved in copper resistance | | |
| COG3143 | 1 | Chemotaxis protein | | |
| COG3144 | 1 | Flagellar hook-length control protein | | |
| COG3145 | 10 | Alkylated DNA repair protein | | |
| COG3146 | 9 | Uncharacterized protein conserved in bacteria | | |
| COG3147 | 2 | Uncharacterized protein conserved in bacteria | | |
| COG3148 | 1 | Uncharacterized conserved protein | | |
| COG3150 | 5 | Predicted esterase | | |
| COG3152 | 1 | Predicted membrane protein | | |
| COG3153 | 5 | Predicted acetyltransferase | | |
| COG3154 | 2 | Putative lipid carrier protein | | |
| COG3155 | 2 | Uncharacterized protein involved in an early stage of isoprenoid biosynthesis | | |
| COG3156 | 10 | Type II secretory pathway, component PulK | | |
| COG3157 | 1 | Hemolysin-coregulated protein (uncharacterized) | | |
| COG3158 | 37 | K+ transporter | | |
| COG3159 | 5 | Uncharacterized protein conserved in bacteria | | |
| COG3162 | 2 | Predicted membrane protein | | |
| COG3164 | 22 | Predicted membrane protein | | |
| COG3165 | 5 | Uncharacterized protein conserved in bacteria | | |
| COG3166 | 6 | Tfp pilus assembly protein PilN | | |
| COG3167 | 9 | Tfp pilus assembly protein PilO | | |

| | | | | |
|---|---|---|---|---|
| COG3168 | 4 | Tfp pilus assembly protein PilP | | |
| COG3169 | 3 | Uncharacterized protein conserved in bacteria | | |
| COG3170 | 17 | Tfp pilus assembly protein FimV | | |
| COG3172 | 1 | Predicted ATPase/kinase involved in NAD metabolism | | |
| COG3173 | 37 | Predicted aminoglycoside phosphotransferase | | |
| COG3174 | 8 | Predicted membrane protein | | |
| COG3175 | 6 | Cytochrome oxidase assembly factor | | |
| COG3176 | 7 | Putative hemolysin | | |
| COG3177 | 12 | Uncharacterized conserved protein | | |
| COG3178 | 16 | Predicted phosphotransferase related to Ser/Thr protein kinases | | |
| COG3179 | 1 | Predicted chitinase | | |
| COG3180 | 8 | Putative ammonia monooxygenase | | |
| COG3181 | 791 | Uncharacterized protein conserved in bacteria | | |
| COG3182 | 3 | Uncharacterized iron-regulated membrane protein | | |
| COG3184 | 2 | Uncharacterized protein conserved in bacteria | | |
| COG3185 | 14 | 4-hydroxyphenylpyruvate dioxygenase and related hemolysins | | |
| COG3186 | 17 | Phenylalanine-4-hydroxylase | | |
| COG3187 | 2 | Heat shock protein | | |
| COG3188 | 10 | P pilus assembly protein, porin PapC | | |
| COG3189 | 9 | Uncharacterized conserved protein | | |
| COG3190 | 2 | Flagellar biogenesis protein | | |
| COG3191 | 23 | L-aminopeptidase/D-esterase | | |
| COG3193 | 20 | Uncharacterized protein, possibly involved in utilization of glycolate and propanediol | | |
| COG3194 | 1 | Ureidoglycolate hydrolase | | |
| COG3195 | 4 | Uncharacterized protein conserved in bacteria | | |
| COG3198 | 2 | Uncharacterized protein conserved in bacteria | | |
| COG3199 | 5 | Uncharacterized conserved protein | | |
| COG3200 | 13 | 3-deoxy-D-arabino-heptulosonate 7-phosphate (DAHP) synthase | | |
| COG3201 | 2 | Nicotinamide mononucleotide transporter | | |
| COG3202 | 15 | ATP/ADP translocase | | |
| COG3203 | 11 | Outer membrane protein (porin) | | |
| COG3204 | 4 | Uncharacterized protein conserved in bacteria | | |
| COG3206 | 21 | Uncharacterized protein involved in exopolysaccharide biosynthesis | | |
| COG3208 | 2 | Predicted thioesterase involved in non-ribosomal peptide biosynthesis | | |
| COG3209 | 82 | Rhs family protein | | |
| COG3210 | 45 | Large exoproteins involved in heme utilization or adhesion | | |
| COG3211 | 7 | Predicted phosphatase | | |
| COG3213 | 7 | Uncharacterized protein involved in response to NO | | |
| COG3214 | 30 | Uncharacterized protein conserved in bacteria | | |
| COG3215 | 2 | Tfp pilus assembly protein PilZ | | |
| COG3216 | 3 | Uncharacterized protein conserved in bacteria | | |
| COG3217 | 12 | Uncharacterized Fe-S protein | | |
| COG3218 | 1 | ABC-type uncharacterized transport system, auxiliary component | | |
| COG3220 | 9 | Uncharacterized protein conserved in bacteria | | |
| COG3221 | 23 | ABC-type phosphate/phosphonate transport system, periplasmic component | | |
| COG3222 | 9 | Uncharacterized protein conserved in bacteria | | |
| COG3223 | 3 | Predicted membrane protein | | |
| COG3225 | 13 | ABC-type uncharacterized transport system involved in gliding motility, auxiliary component | | |
| COG3227 | 8 | Zinc metalloprotease (elastase) | | |
| COG3228 | 8 | Uncharacterized protein conserved in bacteria | | |
| COG3231 | 2 | Aminoglycoside phosphotransferase | | |
| COG3233 | 1 | Predicted deacetylase | | |
| COG3235 | 1 | Predicted membrane protein | | |
| COG3236 | 1 | Uncharacterized protein conserved in bacteria | | |
| COG3237 | 7 | Uncharacterized protein conserved in bacteria | | |
| COG3238 | 3 | Uncharacterized protein conserved in bacteria | | |
| COG3239 | 19 | Fatty acid desaturase | | |
| COG3240 | 2 | Phospholipase/lecithinase/hemolysin | | |
| COG3243 | 53 | Poly(3-hydroxyalkanoate) synthetase | | |
| COG3245 | 11 | Cytochrome c5 | | |
| COG3246 | 24 | Uncharacterized conserved protein | | |
| COG3247 | 4 | Uncharacterized conserved protein | | |
| COG3250 | 34 | Beta-galactosidase/beta-glucuronidase | | |
| COG3251 | 1 | Uncharacterized protein conserved in bacteria | | |
| COG3252 | 3 | Methenyltetrahydromethanopterin cyclohydrolase | | |
| COG3253 | 15 | Uncharacterized conserved protein | | |
| COG3254 | 1 | Uncharacterized conserved protein | | |
| COG3255 | 5 | Putative sterol carrier protein | | |
| COG3256 | 21 | Nitric oxide reductase large subunit | | |
| COG3258 | 6 | Cytochrome c | | |
| COG3259 | 39 | Coenzyme F420-reducing hydrogenase, alpha subunit | | |
| COG3260 | 11 | Ni,Fe-hydrogenase III small subunit | | |
| COG3261 | 18 | Ni,Fe-hydrogenase III large subunit | | |
| COG3262 | 3 | Ni,Fe-hydrogenase III component G | | |
| COG3263 | 16 | NhaP-type Na+/H+ and K+/H+ antiporters with a unique C-terminal domain | | |
| COG3264 | 4 | Small-conductance mechanosensitive channel | | |
| COG3265 | 4 | Gluconate kinase | | |
| COG3267 | 23 | Type II secretory pathway, component ExeA (predicted ATPase) | | |
| COG3268 | 18 | Uncharacterized conserved protein | | |
| COG3271 | 1 | Predicted double-glycine peptidase | | |
| COG3272 | 1 | Uncharacterized conserved protein | | |
| COG3273 | 1 | Uncharacterized conserved protein | | |
| COG3275 | 6 | Putative regulator of cell autolysis | | |
| COG3276 | 28 | Selenocysteine-specific translation elongation factor | | |

| | | | | |
|---|---|---|---|---|
| COG3278 | 16 | Cbb3-type cytochrome oxidase, subunit 1 | | |
| COG3279 | 42 | Response regulator of the LytR/AlgR family | | |
| COG3280 | 33 | Maltooligosyl trehalose synthase | | |
| COG3281 | 12 | Uncharacterized protein, probably involved in trehalose biosynthesis | | |
| COG3283 | 3 | Transcriptional regulator of aromatic amino acids metabolism | | |
| COG3284 | 20 | Transcriptional activator of acetoin/glycerol metabolism | | |
| COG3285 | 63 | Predicted eukaryotic-type DNA primase | | |
| COG3287 | 2 | Uncharacterized conserved protein | | |
| COG3288 | 34 | NAD/NADP transhydrogenase alpha subunit | | |
| COG3291 | 55 | FOG: PKD repeat | | |
| COG3292 | 22 | Predicted periplasmic ligand-binding sensor domain | | |
| COG3293 | 46 | Transposase and inactivated derivatives | | |
| COG3294 | 2 | Uncharacterized conserved protein | | |
| COG3298 | 8 | Predicted 3'-5' exonuclease related to the exonuclease domain of PolB | | |
| COG3300 | 1 | MHYT domain (predicted integral membrane sensor domain) | | |
| COG3301 | 1 | Formate-dependent nitrite reductase, membrane component | | |
| COG3302 | 1 | DMSO reductase anchor subunit | | |
| COG3303 | 9 | Formate-dependent nitrite reductase, periplasmic cytochrome c552 subunit | | |
| COG3304 | 2 | Predicted membrane protein | | |
| COG3305 | 5 | Predicted membrane protein | | |
| COG3307 | 8 | Lipid A core - O-antigen ligase and related enzymes | | |
| COG3310 | 1 | Uncharacterized protein conserved in bacteria | | |
| COG3311 | 1 | Predicted transcriptional regulator | | |
| COG3313 | 1 | Predicted Fe-S protein | | |
| COG3315 | 21 | O-Methyltransferase involved in polyketide biosynthesis | | |
| COG3316 | 2 | Transposase and inactivated derivatives | | |
| COG3317 | 7 | Uncharacterized lipoprotein | | |
| COG3318 | 1 | Predicted metal-binding protein related to the C-terminal domain of SecA | | |
| COG3319 | 5 | Thioesterase domains of type I polyketide synthases or non-ribosomal peptide synthetases | | |
| COG3320 | 11 | Putative dehydrogenase domain of multifunctional non-ribosomal peptide synthetases and related enzymes | | |
| COG3321 | 55 | Polyketide synthase modules and related proteins | | |
| COG3322 | 3 | Predicted periplasmic ligand-binding sensor domain | | |
| COG3324 | 29 | Predicted enzyme related to lactoylglutathione lyase | | |
| COG3325 | 3 | Chitinase | | |
| COG3327 | 4 | Phenylacetic acid-responsive transcriptional repressor | | |
| COG3328 | 61 | Transposase and inactivated derivatives | | |
| COG3329 | 2 | Predicted permease | | |
| COG3330 | 1 | Uncharacterized protein conserved in bacteria | | |
| COG3332 | 5 | Uncharacterized conserved protein | | |
| COG3333 | 90 | Uncharacterized protein conserved in bacteria | | |
| COG3334 | 1 | Uncharacterized conserved protein | | |
| COG3335 | 80 | Transposase and inactivated derivatives | | |
| COG3336 | 11 | Predicted membrane protein | | |
| COG3339 | 6 | Uncharacterized conserved protein | | |
| COG3340 | 9 | Peptidase E | | |
| COG3342 | 18 | Uncharacterized conserved protein | | |
| COG3344 | 56 | Retron-type reverse transcriptase | | |
| COG3345 | 10 | Alpha-galactosidase | | |
| COG3346 | 13 | Uncharacterized conserved protein | | |
| COG3347 | 18 | Uncharacterized conserved protein | | |
| COG3349 | 10 | Uncharacterized conserved protein | | |
| COG3350 | 1 | Uncharacterized conserved protein | | |
| COG3357 | 2 | Predicted transcriptional regulator containing an HTH domain fused to a Zn-ribbon | | |
| COG3358 | 7 | Uncharacterized conserved protein | | |
| COG3359 | 7 | Predicted exonuclease | | |
| COG3360 | 6 | Uncharacterized conserved protein | | |
| COG3361 | 8 | Uncharacterized conserved protein | | |
| COG3363 | 1 | Archaeal IMP cyclohydrolase | | |
| COG3367 | 9 | Uncharacterized conserved protein | | |
| COG3369 | 10 | Uncharacterized conserved protein | | |
| COG3375 | 6 | Uncharacterized conserved protein | | |
| COG3376 | 10 | High-affinity nickel permease | | |
| COG3378 | 3 | Predicted ATPase | | |
| COG3379 | 13 | Uncharacterized conserved protein | | |
| COG3380 | 2 | Predicted NAD/FAD-dependent oxidoreductase | | |
| COG3381 | 5 | Uncharacterized component of anaerobic dehydrogenases | | |
| COG3382 | 5 | Uncharacterized conserved protein | | |
| COG3383 | 46 | Uncharacterized anaerobic dehydrogenase | | |
| COG3384 | 13 | Uncharacterized conserved protein | | |
| COG3385 | 33 | FOG: Transposase and inactivated derivatives | | |
| COG3386 | 70 | Gluconolactonase | | |
| COG3387 | 78 | Glucoamylase and related glycosyl hydrolases | | |
| COG3391 | 254 | Uncharacterized conserved protein | | |
| COG3392 | 3 | Adenine-specific DNA methylase | | |
| COG3393 | 4 | Predicted acetyltransferase | | |
| COG3394 | 5 | Uncharacterized protein conserved in bacteria | | |
| COG3395 | 8 | Uncharacterized protein conserved in bacteria | | |
| COG3396 | 23 | Uncharacterized conserved protein | | |
| COG3397 | 1 | Uncharacterized protein conserved in bacteria | | |
| COG3401 | 3 | Fibronectin type 3 domain-containing protein | | |
| COG3402 | 1 | Uncharacterized conserved protein | | |
| COG3404 | 4 | Methenyl tetrahydrofolate cyclohydrolase | | |
| COG3405 | 3 | Endoglucanase Y | | |
| COG3408 | 43 | Glycogen debranching enzyme | | |

| | | | | |
|---|---|---|---|---|
| COG3409 | 16 | Putative peptidoglycan-binding domain-containing protein | | |
| COG3410 | 1 | Uncharacterized conserved protein | | |
| COG3411 | 8 | Ferredoxin | | |
| COG3412 | 1 | Uncharacterized protein conserved in bacteria | | |
| COG3415 | 13 | Transposase and inactivated derivatives | | |
| COG3417 | 3 | Collagen-binding surface adhesin SpaP (antigen I/II family) | | |
| COG3419 | 38 | Tfp pilus assembly protein, tip-associated adhesin PilY1 | | |
| COG3420 | 9 | Nitrous oxidase accessory protein | | |
| COG3424 | 12 | Predicted naringenin-chalcone synthase | | |
| COG3425 | 4 | 3-hydroxy-3-methylglutaryl CoA synthase | | |
| COG3426 | 3 | Butyrate kinase | | |
| COG3427 | 21 | Uncharacterized conserved protein | | |
| COG3428 | 1 | Predicted membrane protein | | |
| COG3429 | 3 | Glucose-6-P dehydrogenase subunit | | |
| COG3431 | 2 | Predicted membrane protein | | |
| COG3434 | 7 | Predicted signal transduction protein containing EAL and modified HD-GYP domains | | |
| COG3435 | 23 | Gentisate 1,2-dioxygenase | | |
| COG3436 | 48 | Transposase and inactivated derivatives | | |
| COG3437 | 64 | Response regulator containing a CheY-like receiver domain and an HD-GYP domain | | |
| COG3439 | 7 | Uncharacterized conserved protein | | |
| COG3440 | 6 | Predicted restriction endonuclease | | |
| COG3442 | 8 | Predicted glutamine amidotransferase | | |
| COG3444 | 3 | Phosphotransferase system, mannose/fructose/N-acetylgalactosamine-specific component IIB | | |
| COG3447 | 4 | Predicted integral membrane sensor domain | | |
| COG3448 | 2 | CBS-domain-containing membrane protein | | |
| COG3451 | 9 | Type IV secretory pathway, VirB4 components | | |
| COG3452 | 1 | Predicted periplasmic ligand-binding sensor domain | | |
| COG3453 | 1 | Uncharacterized protein conserved in bacteria | | |
| COG3454 | 1 | Metal-dependent hydrolase involved in phosphonate metabolism | | |
| COG3455 | 6 | Uncharacterized protein conserved in bacteria | | |
| COG3456 | 3 | Uncharacterized conserved protein, contains FHA domain | | |
| COG3457 | 1 | Predicted amino acid racemase | | |
| COG3458 | 1 | Acetyl esterase (deacetylase) | | |
| COG3459 | 49 | Cellobiose phosphorylase | | |
| COG3460 | 4 | Uncharacterized enzyme of phenylacetate metabolism | | |
| COG3461 | 2 | Uncharacterized conserved protein | | |
| COG3463 | 2 | Predicted membrane protein | | |
| COG3464 | 26 | Transposase and inactivated derivatives | | |
| COG3467 | 7 | Predicted flavin-nucleotide-binding protein | | |
| COG3468 | 5 | Type V secretory pathway, adhesin AidA | | |
| COG3469 | 2 | Chitinase | | |
| COG3471 | 2 | Predicted periplasmic/secreted protein | | |
| COG3472 | 1 | Uncharacterized conserved protein | | |
| COG3473 | 14 | Maleate cis-trans isomerase | | |
| COG3474 | 5 | Cytochrome c2 | | |
| COG3476 | 10 | Tryptophan-rich sensory protein (mitochondrial benzodiazepine receptor homolog) | | |
| COG3480 | 5 | Predicted secreted protein containing a PDZ domain | | |
| COG3481 | 6 | Predicted HD-superfamily hydrolase | | |
| COG3482 | 1 | Uncharacterized conserved protein | | |
| COG3483 | 11 | Tryptophan 2,3-dioxygenase (vermilion) | | |
| COG3484 | 4 | Predicted proteasome-type protease | | |
| COG3485 | 28 | Protocatechuate 3,4-dioxygenase beta subunit | | |
| COG3486 | 1 | Lysine/ornithine N-monooxygenase | | |
| COG3488 | 6 | Predicted thiol oxidoreductase | | |
| COG3491 | 7 | Isopenicillin N synthase and related dioxygenases | | |
| COG3494 | 10 | Uncharacterized protein conserved in bacteria | | |
| COG3495 | 1 | Uncharacterized protein conserved in bacteria | | |
| COG3496 | 10 | Uncharacterized conserved protein | | |
| COG3497 | 21 | Phage tail sheath protein FI | | |
| COG3500 | 6 | Phage protein D | | |
| COG3501 | 13 | Uncharacterized protein conserved in bacteria | | |
| COG3503 | 12 | Predicted membrane protein | | |
| COG3505 | 5 | Type IV secretory pathway, VirD4 components | | |
| COG3507 | 8 | Beta-xylosidase | | |
| COG3508 | 27 | Homogentisate 1,2-dioxygenase | | |
| COG3509 | 13 | Poly(3-hydroxybutyrate) depolymerase | | |
| COG3510 | 3 | Cephalosporin hydroxylase | | |
| COG3511 | 33 | Phospholipase C | | |
| COG3513 | 4 | Uncharacterized protein conserved in bacteria | | |
| COG3514 | 1 | Uncharacterized protein conserved in bacteria | | |
| COG3515 | 7 | Uncharacterized protein conserved in bacteria | | |
| COG3516 | 8 | Uncharacterized protein conserved in bacteria | | |
| COG3517 | 9 | Uncharacterized protein conserved in bacteria | | |
| COG3518 | 3 | Uncharacterized protein conserved in bacteria | | |
| COG3519 | 7 | Uncharacterized protein conserved in bacteria | | |
| COG3520 | 4 | Uncharacterized protein conserved in bacteria | | |
| COG3521 | 1 | Uncharacterized protein conserved in bacteria | | |
| COG3522 | 8 | Uncharacterized protein conserved in bacteria | | |
| COG3523 | 8 | Uncharacterized protein conserved in bacteria | | |
| COG3525 | 20 | N-acetyl-beta-hexosaminidase | | |
| COG3526 | 1 | Uncharacterized protein conserved in bacteria | | |
| COG3528 | 2 | Uncharacterized protein conserved in bacteria | | |
| COG3531 | 1 | Predicted protein-disulfide isomerase | | |
| COG3533 | 22 | Uncharacterized protein conserved in bacteria | | |

| | | | | |
|---|---|---|---|---|
| COG3534 | 19 | Alpha-L-arabinofuranosidase | | |
| COG3535 | 1 | Uncharacterized conserved protein | | |
| COG3536 | 3 | Uncharacterized protein conserved in bacteria | | |
| COG3537 | 15 | Putative alpha-1,2-mannosidase | | |
| COG3540 | 38 | Phosphodiesterase/alkaline phosphatase D | | |
| COG3541 | 1 | Predicted nucleotidyltransferase | | |
| COG3544 | 3 | Uncharacterized protein conserved in bacteria | | |
| COG3545 | 5 | Predicted esterase of the alpha/beta hydrolase fold | | |
| COG3546 | 1 | Mn-containing catalase | | |
| COG3547 | 167 | Transposase and inactivated derivatives | | |
| COG3548 | 14 | Predicted integral membrane protein | | |
| COG3549 | 3 | Plasmid maintenance system killer protein | | |
| COG3550 | 1 | Uncharacterized protein related to capsule biosynthesis enzymes | | |
| COG3551 | 1 | Uncharacterized protein conserved in bacteria | | |
| COG3552 | 33 | Protein containing von Willebrand factor type A (vWA) domain | | |
| COG3555 | 4 | Aspartyl/asparaginyl beta-hydroxylase and related dioxygenases | | |
| COG3556 | 1 | Predicted membrane protein | | |
| COG3557 | 1 | Uncharacterized domain/protein associated with RNAses G and E | | |
| COG3558 | 7 | Uncharacterized protein conserved in bacteria | | |
| COG3559 | 2 | Putative exporter of polyketide antibiotics | | |
| COG3562 | 3 | Capsule polysaccharide export protein | | |
| COG3563 | 2 | Capsule polysaccharide export protein | | |
| COG3565 | 3 | Predicted dioxygenase of extradiol dioxygenase family | | |
| COG3568 | 21 | Metal-dependent hydrolase | | |
| COG3569 | 18 | Topoisomerase IB | | |
| COG3571 | 7 | Predicted hydrolase of the alpha/beta-hydrolase fold | | |
| COG3572 | 10 | Gamma-glutamylcysteine synthetase | | |
| COG3573 | 9 | Predicted oxidoreductase | | |
| COG3575 | 1 | Uncharacterized protein conserved in bacteria | | |
| COG3576 | 13 | Predicted flavin-nucleotide-binding protein structurally related to pyridoxine 5'-phosphate oxidase | | |
| COG3577 | 5 | Predicted aspartyl protease | | |
| COG3579 | 2 | Aminopeptidase C | | |
| COG3580 | 14 | Uncharacterized protein conserved in bacteria | | |
| COG3581 | 7 | Uncharacterized protein conserved in bacteria | | |
| COG3583 | 3 | Uncharacterized protein conserved in bacteria | | |
| COG3584 | 1 | Uncharacterized protein conserved in bacteria | | |
| COG3585 | 2 | Molybdopterin-binding protein | | |
| COG3586 | 1 | Uncharacterized conserved protein | | |
| COG3587 | 2 | Restriction endonuclease | | |
| COG3588 | 16 | Fructose-1,6-bisphosphate aldolase | | |
| COG3590 | 53 | Predicted metalloendopeptidase | | |
| COG3591 | 1 | V8-like Glu-specific endopeptidase | | |
| COG3592 | 2 | Uncharacterized conserved protein | | |
| COG3593 | 2 | Predicted ATP-dependent endonuclease of the OLD family | | |
| COG3595 | 5 | Uncharacterized conserved protein | | |
| COG3597 | 1 | Uncharacterized protein/domain associated with GTPases | | |
| COG3598 | 1 | RecA-family ATPase | | |
| COG3599 | 12 | Cell division initiation protein | | |
| COG3603 | 3 | Uncharacterized conserved protein | | |
| COG3604 | 43 | Transcriptional regulator containing GAF, AAA-type ATPase, and DNA binding domains | | |
| COG3605 | 7 | Signal transduction protein containing GAF and PtsI domains | | |
| COG3607 | 4 | Predicted lactoylglutathione lyase | | |
| COG3608 | 5 | Predicted deacylase | | |
| COG3609 | 2 | Predicted transcriptional regulators containing the CopG/Arc/MetJ DNA-binding domain | | |
| COG3610 | 3 | Uncharacterized conserved protein | | |
| COG3614 | 2 | Predicted periplasmic ligand-binding sensor domain | | |
| COG3616 | 23 | Predicted amino acid aldolase or racemase | | |
| COG3617 | 1 | Prophage antirepressor | | |
| COG3618 | 32 | Predicted metal-dependent hydrolase of the TIM-barrel fold | | |
| COG3620 | 3 | Predicted transcriptional regulator with C-terminal CBS domains | | |
| COG3621 | 6 | Patatin | | |
| COG3622 | 10 | Hydroxypyruvate isomerase | | |
| COG3626 | 3 | Uncharacterized enzyme of phosphonate metabolism | | |
| COG3627 | 1 | Uncharacterized enzyme of phosphonate metabolism | | |
| COG3628 | 4 | Phage baseplate assembly protein W | | |
| COG3629 | 33 | DNA-binding transcriptional activator of the SARP family | | |
| COG3631 | 3 | Ketosteroid isomerase-related protein | | |
| COG3634 | 1 | Alkyl hydroperoxide reductase, large subunit | | |
| COG3635 | 12 | Predicted phosphoglycerate mutase, AP superfamily | | |
| COG3636 | 1 | Predicted transcriptional regulator | | |
| COG3637 | 4 | Opacity protein and related surface antigens | | |
| COG3638 | 6 | ABC-type phosphate/phosphonate transport system, ATPase component | | |
| COG3639 | 4 | ABC-type phosphate/phosphonate transport system, permease component | | |
| COG3640 | 9 | CO dehydrogenase maturation factor | | |
| COG3643 | 20 | Glutamate formiminotransferase | | |
| COG3647 | 7 | Predicted membrane protein | | |
| COG3648 | 5 | Uricase (urate oxidase) | | |
| COG3649 | 2 | Uncharacterized protein predicted to be involved in DNA repair | | |
| COG3651 | 6 | Uncharacterized protein conserved in bacteria | | |
| COG3652 | 6 | Predicted outer membrane protein | | |
| COG3653 | 83 | N-acyl-D-aspartate/D-glutamate deacylase | | |
| COG3654 | 6 | Prophage maintenance system killer protein | | |
| COG3655 | 1 | Predicted transcriptional regulator | | |
| COG3657 | 3 | Uncharacterized protein conserved in bacteria | | |

| | | | | |
|---|---|---|---|---|
| COG3658 | 4 | Cytochrome b | | |
| COG3659 | 1 | Carbohydrate-selective porin | | |
| COG3660 | 2 | Predicted nucleoside-diphosphate-sugar epimerase | | |
| COG3661 | 3 | Alpha-glucuronidase | | |
| COG3662 | 8 | Uncharacterized protein conserved in bacteria | | |
| COG3663 | 5 | G:T/U mismatch-specific DNA glycosylase | | |
| COG3664 | 12 | Beta-xylosidase | | |
| COG3665 | 6 | Uncharacterized conserved protein | | |
| COG3666 | 45 | Transposase and inactivated derivatives | | |
| COG3667 | 1 | Uncharacterized protein involved in copper resistance | | |
| COG3668 | 6 | Plasmid stabilization system protein | | |
| COG3669 | 18 | Alpha-L-fucosidase | | |
| COG3670 | 4 | Lignostilbene-alpha,beta-dioxygenase and related enzymes | | |
| COG3671 | 2 | Predicted membrane protein | | |
| COG3672 | 1 | Predicted periplasmic protein | | |
| COG3673 | 6 | Uncharacterized conserved protein | | |
| COG3676 | 4 | Transposase and inactivated derivatives | | |
| COG3677 | 2 | Transposase and inactivated derivatives | | |
| COG3682 | 5 | Predicted transcriptional regulator | | |
| COG3684 | 10 | Tagatose-1,6-bisphosphate aldolase | | |
| COG3685 | 10 | Uncharacterized protein conserved in bacteria | | |
| COG3686 | 1 | Predicted membrane protein | | |
| COG3687 | 5 | Predicted metal-dependent hydrolase | | |
| COG3688 | 1 | Predicted RNA-binding protein containing a PIN domain | | |
| COG3689 | 1 | Predicted membrane protein | | |
| COG3693 | 3 | Beta-1,4-xylanase | | |
| COG3694 | 14 | ABC-type uncharacterized transport system, permease component | | |
| COG3695 | 7 | Predicted methylated DNA-protein cysteine methyltransferase | | |
| COG3696 | 179 | Putative silver efflux pump | | |
| COG3701 | 1 | Type IV secretory pathway, TrbF components | | |
| COG3702 | 1 | Type IV secretory pathway, VirB3 components | | |
| COG3703 | 6 | Uncharacterized protein involved in cation transport | | |
| COG3704 | 2 | Type IV secretory pathway, VirB6 components | | |
| COG3705 | 15 | ATP phosphoribosyltransferase involved in histidine biosynthesis | | |
| COG3706 | 40 | Response regulator containing a CheY-like receiver domain and a GGDEF domain | | |
| COG3707 | 14 | Response regulator with putative antiterminator output domain | | |
| COG3710 | 11 | DNA-binding winged-HTH domains | | |
| COG3712 | 1 | Fe2+-dicitrate sensor, membrane component | | |
| COG3713 | 6 | Outer membrane protein V | | |
| COG3714 | 2 | Predicted membrane protein | | |
| COG3715 | 2 | Phosphotransferase system, mannose/fructose/N-acetylgalactosamine-specific component IIC | | |
| COG3716 | 6 | Phosphotransferase system, mannose/fructose/N-acetylgalactosamine-specific component IID | | |
| COG3718 | 2 | Uncharacterized enzyme involved in inositol metabolism | | |
| COG3719 | 1 | Ribonuclease I | | |
| COG3723 | 1 | Recombinational DNA repair protein (RecE pathway) | | |
| COG3724 | 1 | Succinylarginine dihydrolase | | |
| COG3726 | 1 | Uncharacterized membrane protein affecting hemolysin expression | | |
| COG3727 | 4 | DNA G:T-mismatch repair endonuclease | | |
| COG3729 | 1 | General stress protein | | |
| COG3733 | 10 | Cu2+-containing amine oxidase | | |
| COG3734 | 1 | 2-keto-3-deoxy-galactonokinase | | |
| COG3735 | 6 | Uncharacterized protein conserved in bacteria | | |
| COG3737 | 4 | Uncharacterized conserved protein | | |
| COG3738 | 1 | Uncharacterized protein conserved in bacteria | | |
| COG3740 | 4 | Phage head maturation protease | | |
| COG3741 | 7 | N-formylglutamate amidohydrolase | | |
| COG3742 | 2 | Uncharacterized protein conserved in bacteria | | |
| COG3743 | 1 | Uncharacterized conserved protein | | |
| COG3744 | 3 | Uncharacterized protein conserved in bacteria | | |
| COG3745 | 5 | Flp pilus assembly protein CpaB | | |
| COG3746 | 1 | Phosphate-selective porin | | |
| COG3748 | 20 | Predicted membrane protein | | |
| COG3749 | 4 | Uncharacterized protein conserved in bacteria | | |
| COG3752 | 4 | Predicted membrane protein | | |
| COG3753 | 4 | Uncharacterized protein conserved in bacteria | | |
| COG3754 | 4 | Lipopolysaccharide biosynthesis protein | | |
| COG3755 | 1 | Uncharacterized protein conserved in bacteria | | |
| COG3757 | 4 | Lyzozyme M1 (1,4-beta-N-acetylmuramidase) | | |
| COG3759 | 2 | Predicted membrane protein | | |
| COG3761 | 3 | NADH:ubiquinone oxidoreductase 17.2 kD subunit | | |
| COG3762 | 7 | Predicted membrane protein | | |
| COG3764 | 9 | Sortase (surface protein transpeptidase) | | |
| COG3766 | 3 | Predicted membrane protein | | |
| COG3768 | 1 | Predicted membrane protein | | |
| COG3772 | 1 | Phage-related lysozyme (muraminidase) | | |
| COG3773 | 4 | Cell wall hydrolyses involved in spore germination | | |
| COG3774 | 1 | Mannosyltransferase OCH1 and related enzymes | | |
| COG3775 | 2 | Phosphotransferase system, galactitol-specific IIC component | | |
| COG3777 | 8 | Uncharacterized conserved protein | | |
| COG3779 | 1 | Uncharacterized protein conserved in bacteria | | |
| COG3780 | 1 | DNA endonuclease related to intein-encoded endonucleases | | |
| COG3784 | 1 | Uncharacterized protein conserved in bacteria | | |
| COG3785 | 2 | Uncharacterized conserved protein | | |
| COG3786 | 5 | Uncharacterized protein conserved in bacteria | | |

| | | | | |
|---|---|---|---|---|
| COG3791 | 35 | Uncharacterized conserved protein | | |
| COG3794 | 17 | Plastocyanin | | |
| COG3795 | 27 | Uncharacterized protein conserved in bacteria | | |
| COG3797 | 6 | Uncharacterized protein conserved in bacteria | | |
| COG3800 | 3 | Predicted transcriptional regulator | | |
| COG3801 | 7 | Uncharacterized protein conserved in bacteria | | |
| COG3803 | 3 | Uncharacterized protein conserved in bacteria | | |
| COG3804 | 7 | Uncharacterized conserved protein related to dihydrodipicolinate reductase | | |
| COG3805 | 2 | Aromatic ring-cleaving dioxygenase | | |
| COG3806 | 1 | Anti-sigma factor | | |
| COG3807 | 1 | Uncharacterized protein conserved in bacteria | | |
| COG3808 | 28 | Inorganic pyrophosphatase | | |
| COG3809 | 4 | Uncharacterized protein conserved in bacteria | | |
| COG3812 | 6 | Uncharacterized protein conserved in bacteria | | |
| COG3813 | 4 | Uncharacterized protein conserved in bacteria | | |
| COG3815 | 2 | Predicted membrane protein | | |
| COG3816 | 2 | Uncharacterized protein conserved in bacteria | | |
| COG3818 | 1 | Predicted acetyltransferase, GNAT superfamily | | |
| COG3819 | 2 | Predicted membrane protein | | |
| COG3821 | 2 | Predicted membrane protein | | |
| COG3822 | 2 | ABC-type sugar transport system, auxiliary component | | |
| COG3823 | 3 | Glutamine cyclotransferase | | |
| COG3824 | 7 | Uncharacterized protein conserved in bacteria | | |
| COG3825 | 5 | Uncharacterized protein conserved in bacteria | | |
| COG3826 | 7 | Uncharacterized protein conserved in bacteria | | |
| COG3828 | 4 | Uncharacterized protein conserved in bacteria | | |
| COG3829 | 26 | Transcriptional regulator containing PAS, AAA-type ATPase, and DNA-binding domains | | |
| COG3830 | 1 | ACT domain-containing protein | | |
| COG3832 | 49 | Uncharacterized conserved protein | | |
| COG3833 | 4 | ABC-type maltose transport systems, permease component | | |
| COG3835 | 2 | Sugar diacid utilization regulator | | |
| COG3836 | 38 | 2,4-dihydroxyhept-2-ene-1,7-dioic acid aldolase | | |
| COG3837 | 15 | Uncharacterized conserved protein, contains double-stranded beta-helix domain | | |
| COG3838 | 2 | Type IV secretory pathway, VirB2 components (pilins) | | |
| COG3839 | 35 | ABC-type sugar transport systems, ATPase components | | |
| COG3842 | 39 | ABC-type spermidine/putrescine transport systems, ATPase components | | |
| COG3843 | 3 | Type IV secretory pathway, VirD2 components (relaxase) | | |
| COG3844 | 16 | Kynureninase | | |
| COG3845 | 35 | ABC-type uncharacterized transport systems, ATPase components | | |
| COG3850 | 13 | Signal transduction histidine kinase, nitrate/nitrite-specific | | |
| COG3851 | 3 | Signal transduction histidine kinase, glucose-6-phosphate specific | | |
| COG3852 | 15 | Signal transduction histidine kinase, nitrogen specific | | |
| COG3853 | 5 | Uncharacterized protein involved in tellurite resistance | | |
| COG3854 | 4 | Uncharacterized protein conserved in bacteria | | |
| COG3856 | 2 | Uncharacterized conserved protein (small basic protein) | | |
| COG3857 | 2 | ATP-dependent nuclease, subunit B | | |
| COG3858 | 14 | Predicted glycosyl hydrolase | | |
| COG3860 | 2 | Uncharacterized protein conserved in bacteria | | |
| COG3861 | 1 | Uncharacterized protein conserved in bacteria | | |
| COG3864 | 1 | Uncharacterized protein conserved in bacteria | | |
| COG3865 | 21 | Uncharacterized protein conserved in bacteria | | |
| COG3866 | 1 | Pectate lyase | | |
| COG3867 | 2 | Arabinogalactan endo-1,4-beta-galactosidase | | |
| COG3868 | 3 | Uncharacterized conserved protein | | |
| COG3869 | 4 | Arginine kinase | | |
| COG3870 | 5 | Uncharacterized protein conserved in bacteria | | |
| COG3871 | 2 | Uncharacterized stress protein (general stress protein 26) | | |
| COG3872 | 15 | Predicted metal-dependent enzyme | | |
| COG3875 | 21 | Uncharacterized conserved protein | | |
| COG3876 | 26 | Uncharacterized protein conserved in bacteria | | |
| COG3877 | 1 | Uncharacterized protein conserved in bacteria | | |
| COG3878 | 1 | Uncharacterized protein conserved in bacteria | | |
| COG3879 | 5 | Uncharacterized protein conserved in bacteria | | |
| COG3880 | 2 | Uncharacterized protein with conserved CXXC pairs | | |
| COG3882 | 4 | Predicted enzyme involved in methoxymalonyl-ACP biosynthesis | | |
| COG3883 | 3 | Uncharacterized protein conserved in bacteria | | |
| COG3885 | 1 | Uncharacterized conserved protein | | |
| COG3886 | 3 | Predicted HKD family nuclease | | |
| COG3887 | 2 | Predicted signaling protein consisting of a modified GGDEF domain and a DHH domain | | |
| COG3892 | 1 | Uncharacterized protein conserved in bacteria | | |
| COG3893 | 4 | Inactivated superfamily I helicase | | |
| COG3894 | 25 | Uncharacterized metal-binding protein | | |
| COG3897 | 3 | Predicted methyltransferase | | |
| COG3899 | 98 | Predicted ATPase | | |
| COG3901 | 1 | Regulator of nitric oxide reductase transcription | | |
| COG3903 | 80 | Predicted ATPase | | |
| COG3907 | 1 | PAP2 (acid phosphatase) superfamily protein | | |
| COG3910 | 5 | Predicted ATPase | | |
| COG3911 | 1 | Predicted ATPase | | |
| COG3913 | 5 | Uncharacterized protein conserved in bacteria | | |
| COG3914 | 17 | Predicted O-linked N-acetylglucosamine transferase, SPINDLY family | | |
| COG3917 | 14 | 2-hydroxychromene-2-carboxylate isomerase | | |
| COG3919 | 5 | Predicted ATP-grasp enzyme | | |
| COG3920 | 26 | Signal transduction histidine kinase | | |

| | | | | |
|---|---|---|---|---|
| COG3921 | 1 | Uncharacterized protein conserved in bacteria | | |
| COG3926 | 2 | Putative secretion activating protein | | |
| COG3930 | 2 | Uncharacterized protein conserved in bacteria | | |
| COG3932 | 2 | Uncharacterized ABC-type transport system, permease components | | |
| COG3934 | 3 | Endo-beta-mannanase | | |
| COG3937 | 1 | Uncharacterized conserved protein | | |
| COG3938 | 12 | Proline racemase | | |
| COG3943 | 1 | Virulence protein | | |
| COG3944 | 1 | Capsular polysaccharide biosynthesis protein | | |
| COG3945 | 8 | Uncharacterized conserved protein | | |
| COG3946 | 5 | Type IV secretory pathway, VirJ component | | |
| COG3947 | 3 | Response regulator containing CheY-like receiver and SARP domains | | |
| COG3951 | 2 | Rod binding protein | | |
| COG3952 | 1 | Predicted membrane protein | | |
| COG3953 | 2 | SLT domain proteins | | |
| COG3954 | 7 | Phosphoribulokinase | | |
| COG3956 | 8 | Protein containing tetrapyrrole methyltransferase domain and MazG-like (predicted pyrophosphatase) domain | | |
| COG3957 | 21 | Phosphoketolase | | |
| COG3958 | 11 | Transketolase, C-terminal subunit | | |
| COG3959 | 14 | Transketolase, N-terminal subunit | | |
| COG3960 | 7 | Glyoxylate carboligase | | |
| COG3961 | 3 | Pyruvate decarboxylase and related thiamine pyrophosphate-requiring enzymes | | |
| COG3962 | 12 | Acetolactate synthase | | |
| COG3963 | 3 | Phospholipid N-methyltransferase | | |
| COG3964 | 8 | Predicted amidohydrolase | | |
| COG3967 | 1 | Short-chain dehydrogenase involved in D-alanine esterification of lipoteichoic acid and wall teichoic acid (D-alanine transfer protein) | | |
| COG3968 | 11 | Uncharacterized protein related to glutamine synthetase | | |
| COG3969 | 3 | Predicted phosphoadenosine phosphosulfate sulfotransferase | | |
| COG3970 | 10 | Fumarylacetoacetate (FAA) hydrolase family protein | | |
| COG3971 | 11 | 2-keto-4-pentenoate hydratase | | |
| COG3972 | 4 | Superfamily I DNA and RNA helicases | | |
| COG3973 | 13 | Superfamily I DNA and RNA helicases | | |
| COG3975 | 35 | Predicted protease with the C-terminal PDZ domain | | |
| COG3979 | 35 | Uncharacterized protein contain chitin-binding domain type 3 | | |
| COG3980 | 1 | Spore coat polysaccharide biosynthesis protein, predicted glycosyltransferase | | |
| COG3981 | 1 | Predicted acetyltransferase | | |
| COG4012 | 3 | Uncharacterized protein conserved in archaea | | |
| COG4032 | 5 | Predicted thiamine-pyrophosphate-binding protein | | |
| COG4034 | 1 | Uncharacterized protein conserved in archaea | | |
| COG4060 | 1 | Tetrahydromethanopterin S-methyltransferase, subunit D | | |
| COG4089 | 1 | Predicted membrane protein | | |
| COG4091 | 20 | Predicted homoserine dehydrogenase | | |
| COG4094 | 10 | Predicted membrane protein | | |
| COG4095 | 3 | Uncharacterized conserved protein | | |
| COG4096 | 27 | Type I site-specific restriction-modification system, R (restriction) subunit and related helicases | | |
| COG4097 | 5 | Predicted ferric reductase | | |
| COG4099 | 4 | Predicted peptidase | | |
| COG4101 | 4 | Predicted mannose-6-phosphate isomerase | | |
| COG4102 | 19 | Uncharacterized protein conserved in bacteria | | |
| COG4103 | 1 | Uncharacterized protein conserved in bacteria | | |
| COG4105 | 17 | DNA uptake lipoprotein | | |
| COG4106 | 2 | Trans-aconitate methyltransferase | | |
| COG4107 | 1 | ABC-type phosphonate transport system, ATPase component | | |
| COG4108 | 18 | Peptide chain release factor RF-3 | | |
| COG4109 | 1 | Predicted transcriptional regulator containing CBS domains | | |
| COG4111 | 2 | Uncharacterized conserved protein | | |
| COG4112 | 1 | Predicted phosphoesterase (MutT family) | | |
| COG4113 | 3 | Predicted nucleic acid-binding protein, contains PIN domain | | |
| COG4115 | 1 | Uncharacterized protein conserved in bacteria | | |
| COG4117 | 9 | Thiosulfate reductase cytochrome B subunit (membrane anchoring protein) | | |
| COG4118 | 2 | Antitoxin of toxin-antitoxin stability system | | |
| COG4119 | 7 | Predicted NTP pyrophosphohydrolase | | |
| COG4120 | 1 | ABC-type uncharacterized transport system, permease component | | |
| COG4121 | 3 | Uncharacterized conserved protein | | |
| COG4122 | 19 | Predicted O-methyltransferase | | |
| COG4123 | 1 | Predicted O-methyltransferase | | |
| COG4124 | 7 | Beta-mannanase | | |
| COG4126 | 6 | Hydantoin racemase | | |
| COG4132 | 6 | ABC-type uncharacterized transport system, permease component | | |
| COG4133 | 4 | ABC-type transport system involved in cytochrome c biogenesis, ATPase component | | |
| COG4134 | 5 | ABC-type uncharacterized transport system, periplasmic component | | |
| COG4137 | 3 | ABC-type uncharacterized transport system, permease component | | |
| COG4143 | 3 | ABC-type thiamine transport system, periplasmic component | | |
| COG4145 | 1 | Na+/panthothenate symporter | | |
| COG4146 | 11 | Predicted symporter | | |
| COG4147 | 25 | Predicted symporter | | |
| COG4148 | 5 | ABC-type molybdate transport system, ATPase component | | |
| COG4149 | 9 | ABC-type molybdate transport system, permease component | | |
| COG4152 | 11 | ABC-type uncharacterized transport system, ATPase component | | |
| COG4154 | 1 | Fucose dissimilation pathway protein FucU | | |
| COG4158 | 2 | Predicted ABC-type sugar transport system, permease component | | |
| COG4166 | 44 | ABC-type oligopeptide transport system, periplasmic component | | |
| COG4172 | 2 | ABC-type uncharacterized transport system, duplicated ATPase component | | |
| COG4174 | 2 | ABC-type uncharacterized transport system, permease component | | |

| | | | | |
|---|---|---|---|---|
| COG4175 | 1 | ABC-type proline/glycine betaine transport system, ATPase component | | |
| COG4176 | 1 | ABC-type proline/glycine betaine transport system, permease component | | |
| COG4177 | 209 | ABC-type branched-chain amino acid transport system, permease component | | |
| COG4178 | 16 | ABC-type uncharacterized transport system, permease and ATPase components | | |
| COG4181 | 8 | Predicted ABC-type transport system involved in lysophospholipase L1 biosynthesis, ATPase component | | |
| COG4185 | 2 | Uncharacterized protein conserved in bacteria | | |
| COG4188 | 6 | Predicted dienelactone hydrolase | | |
| COG4190 | 2 | Predicted transcriptional regulator | | |
| COG4191 | 13 | Signal transduction histidine kinase regulating C4-dicarboxylate transport system | | |
| COG4195 | 1 | Phage-related replication protein | | |
| COG4196 | 10 | Uncharacterized protein conserved in bacteria | | |
| COG4198 | 11 | Uncharacterized conserved protein | | |
| COG4200 | 1 | Uncharacterized protein conserved in bacteria | | |
| COG4206 | 19 | Outer membrane cobalamin receptor protein | | |
| COG4208 | 8 | ABC-type sulfate transport system, permease component | | |
| COG4211 | 1 | ABC-type glucose/galactose transport system, permease component | | |
| COG4213 | 5 | ABC-type xylose transport system, periplasmic component | | |
| COG4214 | 14 | ABC-type xylose transport system, permease component | | |
| COG4219 | 3 | Antirepressor regulating drug resistance, predicted signal transduction N-terminal membrane component | | |
| COG4220 | 2 | Phage DNA packaging protein, Nu1 subunit of terminase | | |
| COG4221 | 21 | Short-chain alcohol dehydrogenase of unknown specificity | | |
| COG4222 | 2 | Uncharacterized protein conserved in bacteria | | |
| COG4225 | 1 | Predicted unsaturated glucuronyl hydrolase involved in regulation of bacterial surface properties, and related proteins | | |
| COG4226 | 1 | Uncharacterized protein encoded in hypervariable junctions of pilus gene clusters | | |
| COG4227 | 3 | Antirestriction protein | | |
| COG4230 | 10 | Delta 1-pyrroline-5-carboxylate dehydrogenase | | |
| COG4231 | 66 | Indolepyruvate ferredoxin oxidoreductase, alpha and beta subunits | | |
| COG4232 | 22 | Thiol:disulfide interchange protein | | |
| COG4233 | 5 | Uncharacterized protein predicted to be involved in C-type cytochrome biogenesis | | |
| COG4235 | 8 | Cytochrome c biogenesis factor | | |
| COG4237 | 6 | Hydrogenase 4 membrane component (E) | | |
| COG4239 | 4 | ABC-type uncharacterized transport system, permease component | | |
| COG4242 | 2 | Cyanophycinase and related exopeptidases | | |
| COG4243 | 1 | Predicted membrane protein | | |
| COG4244 | 6 | Predicted membrane protein | | |
| COG4248 | 2 | Uncharacterized protein with protein kinase and helix-hairpin-helix DNA-binding domains | | |
| COG4249 | 17 | Uncharacterized protein containing caspase domain | | |
| COG4250 | 1 | Predicted sensor protein/domain | | |
| COG4251 | 10 | Bacteriophytochrome (light-regulated signal transduction histidine kinase) | | |
| COG4252 | 25 | Predicted transmembrane sensor domain | | |
| COG4254 | 3 | Uncharacterized protein conserved in bacteria | | |
| COG4255 | 2 | Uncharacterized protein conserved in bacteria | | |
| COG4256 | 1 | Hemin uptake protein | | |
| COG4257 | 30 | Streptogramin lyase | | |
| COG4258 | 10 | Predicted exporter | | |
| COG4260 | 5 | Putative virion core protein (lumpy skin disease virus) | | |
| COG4261 | 2 | Predicted acyltransferase | | |
| COG4262 | 3 | Predicted spermidine synthase with an N-terminal membrane domain | | |
| COG4263 | 14 | Nitrous oxide reductase | | |
| COG4266 | 8 | Allantoicase | | |
| COG4267 | 2 | Predicted membrane protein | | |
| COG4269 | 7 | Predicted membrane protein | | |
| COG4271 | 3 | Predicted nucleotide-binding protein containing TIR -like domain | | |
| COG4274 | 2 | Uncharacterized conserved protein | | |
| COG4275 | 30 | Uncharacterized conserved protein | | |
| COG4276 | 3 | Uncharacterized conserved protein | | |
| COG4277 | 6 | Predicted DNA-binding protein with the Helix-hairpin-helix motif | | |
| COG4279 | 1 | Uncharacterized conserved protein | | |
| COG4280 | 6 | Predicted membrane protein | | |
| COG4281 | 2 | Acyl-CoA-binding protein | | |
| COG4283 | 1 | Uncharacterized conserved protein | | |
| COG4284 | 5 | UDP-glucose pyrophosphorylase | | |
| COG4286 | 1 | Uncharacterized conserved protein related to MYG1 family | | |
| COG4287 | 5 | PhoPQ-activated pathogenicity-related protein | | |
| COG4288 | 1 | Uncharacterized protein conserved in bacteria | | |
| COG4289 | 1 | Uncharacterized protein conserved in bacteria | | |
| COG4290 | 9 | Guanyl-specific ribonuclease Sa | | |
| COG4291 | 3 | Predicted membrane protein | | |
| COG4292 | 11 | Predicted membrane protein | | |
| COG4293 | 3 | Uncharacterized protein conserved in bacteria | | |
| COG4299 | 6 | Uncharacterized conserved protein | | |
| COG4301 | 21 | Uncharacterized conserved protein | | |
| COG4302 | 1 | Ethanolamine ammonia-lyase, small subunit | | |
| COG4303 | 4 | Ethanolamine ammonia-lyase, large subunit | | |
| COG4306 | 1 | Uncharacterized protein conserved in bacteria | | |
| COG4308 | 1 | Limonene-1,2-epoxide hydrolase | | |
| COG4309 | 1 | Uncharacterized conserved protein | | |
| COG4310 | 4 | Uncharacterized protein conserved in bacteria with an aminopeptidase-like domain | | |
| COG4311 | 2 | Sarcosine oxidase delta subunit | | |
| COG4312 | 25 | Uncharacterized protein conserved in bacteria | | |
| COG4313 | 4 | Protein involved in meta-pathway of phenol degradation | | |
| COG4315 | 10 | Uncharacterized protein conserved in bacteria | | |
| COG4319 | 3 | Ketosteroid isomerase homolog | | |
| COG4320 | 10 | Uncharacterized protein conserved in bacteria | | |

| | | | | |
|---|---|---|---|---|
| COG4323 | 3 | Predicted membrane protein | | |
| COG4324 | 11 | Predicted aminopeptidase | | |
| COG4325 | 4 | Predicted membrane protein | | |
| COG4327 | 6 | Predicted membrane protein | | |
| COG4328 | 1 | Uncharacterized protein conserved in bacteria | | |
| COG4330 | 2 | Predicted membrane protein | | |
| COG4333 | 2 | Uncharacterized protein conserved in bacteria | | |
| COG4336 | 6 | Uncharacterized conserved protein | | |
| COG4338 | 1 | Uncharacterized protein conserved in bacteria | | |
| COG4339 | 1 | Uncharacterized protein conserved in bacteria | | |
| COG4340 | 1 | Uncharacterized protein conserved in bacteria | | |
| COG4346 | 1 | Predicted membrane-bound dolichyl-phosphate-mannose-protein mannosyltransferase | | |
| COG4352 | 1 | Ribosomal protein L13E | | |
| COG4354 | 1 | Predicted bile acid beta-glucosidase | | |
| COG4359 | 1 | Uncharacterized conserved protein, possibly involved in methylthioadenosine recycling | | |
| COG4362 | 2 | Nitric oxide synthase, oxygenase domain | | |
| COG4365 | 6 | Uncharacterized protein conserved in bacteria | | |
| COG4373 | 1 | Mu-like prophage FluMu protein gp28 | | |
| COG4374 | 1 | Uncharacterized protein conserved in bacteria | | |
| COG4380 | 1 | Uncharacterized protein conserved in bacteria | | |
| COG4389 | 1 | Site-specific recombinase | | |
| COG4391 | 2 | Uncharacterized protein conserved in bacteria | | |
| COG4392 | 5 | Predicted membrane protein | | |
| COG4394 | 2 | Uncharacterized protein conserved in bacteria | | |
| COG4395 | 6 | Uncharacterized protein conserved in bacteria | | |
| COG4398 | 19 | Uncharacterized protein conserved in bacteria | | |
| COG4399 | 1 | Uncharacterized protein conserved in bacteria | | |
| COG4401 | 12 | Chorismate mutase | | |
| COG4402 | 5 | Uncharacterized protein conserved in bacteria | | |
| COG4409 | 1 | Neuraminidase (sialidase) | | |
| COG4412 | 5 | Uncharacterized protein conserved in bacteria | | |
| COG4420 | 12 | Predicted membrane protein | | |
| COG4422 | 11 | Bacteriophage protein gp37 | | |
| COG4424 | 1 | Uncharacterized protein conserved in bacteria | | |
| COG4425 | 3 | Predicted membrane protein | | |
| COG4427 | 5 | Uncharacterized protein conserved in bacteria | | |
| COG4430 | 7 | Uncharacterized protein conserved in bacteria | | |
| COG4443 | 1 | Uncharacterized protein conserved in bacteria | | |
| COG4446 | 1 | Uncharacterized protein conserved in bacteria | | |
| COG4447 | 11 | Uncharacterized protein related to plant photosystem II stability/assembly factor | | |
| COG4448 | 15 | L-asparaginase II | | |
| COG4451 | 3 | Ribulose bisphosphate carboxylase small subunit | | |
| COG4452 | 10 | Inner membrane protein involved in colicin E2 resistance | | |
| COG4453 | 1 | Uncharacterized protein conserved in bacteria | | |
| COG4454 | 7 | Uncharacterized copper-binding protein | | |
| COG4455 | 1 | Protein of avirulence locus involved in temperature-dependent protein secretion | | |
| COG4459 | 1 | Periplasmic nitrate reductase system, NapE component | | |
| COG4464 | 12 | Capsular polysaccharide biosynthesis protein | | |
| COG4478 | 1 | Predicted membrane protein | | |
| COG4481 | 1 | Uncharacterized protein conserved in bacteria | | |
| COG4487 | 4 | Uncharacterized protein conserved in bacteria | | |
| COG4493 | 1 | Uncharacterized protein conserved in bacteria | | |
| COG4508 | 1 | Uncharacterized protein conserved in bacteria | | |
| COG4517 | 1 | Uncharacterized protein conserved in bacteria | | |
| COG4520 | 4 | Surface antigen | | |
| COG4521 | 3 | ABC-type taurine transport system, periplasmic component | | |
| COG4529 | 3 | Uncharacterized protein conserved in bacteria | | |
| COG4533 | 1 | ABC-type uncharacterized transport system, periplasmic component | | |
| COG4535 | 5 | Putative Mg2+ and Co2+ transporter CorC | | |
| COG4536 | 9 | Putative Mg2+ and Co2+ transporter CorB | | |
| COG4539 | 5 | Predicted membrane protein | | |
| COG4544 | 1 | Uncharacterized conserved protein | | |
| COG4547 | 6 | Cobalamin biosynthesis protein CobT (nicotinate-mononucleotide:5, 6-dimethylbenzimidazole phosphoribosyltransferase) | | |
| COG4548 | 3 | Nitric oxide reductase activation protein | | |
| COG4552 | 3 | Predicted acetyltransferase involved in intracellular survival and related acetyltransferases | | |
| COG4553 | 10 | Poly-beta-hydroxyalkanoate depolymerase | | |
| COG4555 | 4 | ABC-type Na+ transport system, ATPase component | | |
| COG4558 | 2 | ABC-type hemin transport system, periplasmic component | | |
| COG4559 | 1 | ABC-type hemin transport system, ATPase component | | |
| COG4564 | 15 | Signal transduction histidine kinase | | |
| COG4565 | 2 | Response regulator of citrate/malate metabolism | | |
| COG4566 | 20 | Response regulator | | |
| COG4567 | 7 | Response regulator consisting of a CheY-like receiver domain and a Fis-type HTH domain | | |
| COG4569 | 3 | Acetaldehyde dehydrogenase (acetylating) | | |
| COG4570 | 1 | Holliday junction resolvase | | |
| COG4572 | 1 | Putative cation transport regulator | | |
| COG4573 | 3 | Predicted tagatose 6-phosphate kinase | | |
| COG4575 | 4 | Uncharacterized conserved protein | | |
| COG4576 | 4 | Carbon dioxide concentrating mechanism/carboxysome shell protein | | |
| COG4577 | 9 | Carbon dioxide concentrating mechanism/carboxysome shell protein | | |
| COG4579 | 11 | Isocitrate dehydrogenase kinase/phosphatase | | |
| COG4580 | 1 | Maltoporin (phage lambda and maltose receptor) | | |
| COG4581 | 30 | Superfamily II RNA helicase | | |
| COG4582 | 2 | Uncharacterized protein conserved in bacteria | | |

| | | | | |
|---|---|---|---|---|
| COG4583 | 1 | Sarcosine oxidase gamma subunit | | |
| COG4584 | 53 | Transposase and inactivated derivatives | | |
| COG4585 | 96 | Signal transduction histidine kinase | | |
| COG4586 | 7 | ABC-type uncharacterized transport system, ATPase component | | |
| COG4587 | 7 | ABC-type uncharacterized transport system, permease component | | |
| COG4589 | 4 | Predicted CDP-diglyceride synthetase/phosphatidate cytidylyltransferase | | |
| COG4590 | 6 | ABC-type uncharacterized transport system, permease component | | |
| COG4591 | 39 | ABC-type transport system, involved in lipoprotein release, permease component | | |
| COG4597 | 2 | ABC-type amino acid transport system, permease component | | |
| COG4603 | 29 | ABC-type uncharacterized transport system, permease component | | |
| COG4608 | 39 | ABC-type oligopeptide transport system, ATPase component | | |
| COG4618 | 8 | ABC-type protease/lipase transport system, ATPase and permease components | | |
| COG4623 | 8 | Predicted soluble lytic transglycosylase fused to an ABC-type amino acid-binding protein | | |
| COG4624 | 2 | Iron only hydrogenase large subunit, C-terminal domain | | |
| COG4625 | 4 | Uncharacterized protein with a C-terminal OMP (outer membrane protein) domain | | |
| COG4626 | 4 | Phage terminase-like protein, large subunit | | |
| COG4627 | 4 | Uncharacterized protein conserved in bacteria | | |
| COG4630 | 2 | Xanthine dehydrogenase, iron-sulfur cluster and FAD-binding subunit A | | |
| COG4631 | 10 | Xanthine dehydrogenase, molybdopterin-binding subunit B | | |
| COG4632 | 1 | Exopolysaccharide biosynthesis protein related to N-acetylglucosamine-1-phosphodiester alpha-N-acetylglucosaminidase | | |
| COG4633 | 4 | Uncharacterized protein conserved in bacteria | | |
| COG4634 | 1 | Uncharacterized protein conserved in bacteria | | |
| COG4635 | 8 | Flavodoxin | | |
| COG4636 | 19 | Uncharacterized protein conserved in cyanobacteria | | |
| COG4637 | 5 | Predicted ATPase | | |
| COG4638 | 84 | Phenylpropionate dioxygenase and related ring-hydroxylating dioxygenases, large terminal subunit | | |
| COG4639 | 4 | Predicted kinase | | |
| COG4641 | 18 | Uncharacterized protein conserved in bacteria | | |
| COG4642 | 4 | Uncharacterized protein conserved in bacteria | | |
| COG4643 | 1 | Uncharacterized protein conserved in bacteria | | |
| COG4644 | 7 | Transposase and inactivated derivatives, TnpA family | | |
| COG4646 | 4 | DNA methylase | | |
| COG4648 | 2 | Predicted membrane protein | | |
| COG4650 | 1 | Sigma54-dependent transcription regulator containing an AAA-type ATPase domain and a DNA-binding domain | | |
| COG4651 | 5 | Kef-type K+ transport system, predicted NAD-binding component | | |
| COG4653 | 1 | Predicted phage phi-C31 gp36 major capsid-like protein | | |
| COG4654 | 7 | Cytochrome c551/c552 | | |
| COG4656 | 8 | Predicted NADH:ubiquinone oxidoreductase, subunit RnfC | | |
| COG4657 | 2 | Predicted NADH:ubiquinone oxidoreductase, subunit RnfA | | |
| COG4658 | 3 | Predicted NADH:ubiquinone oxidoreductase, subunit RnfD | | |
| COG4659 | 2 | Predicted NADH:ubiquinone oxidoreductase, subunit RnfG | | |
| COG4660 | 2 | Predicted NADH:ubiquinone oxidoreductase, subunit RnfE | | |
| COG4662 | 8 | ABC-type tungstate transport system, periplasmic component | | |
| COG4663 | 14 | TRAP-type mannitol/chloroaromatic compound transport system, periplasmic component | | |
| COG4664 | 36 | TRAP-type mannitol/chloroaromatic compound transport system, large permease component | | |
| COG4665 | 20 | TRAP-type mannitol/chloroaromatic compound transport system, small permease component | | |
| COG4666 | 32 | TRAP-type uncharacterized transport system, fused permease components | | |
| COG4669 | 3 | Type III secretory pathway, lipoprotein EscJ | | |
| COG4670 | 7 | Acyl CoA:acetate/3-ketoacid CoA transferase | | |
| COG4671 | 16 | Predicted glycosyl transferase | | |
| COG4674 | 3 | Uncharacterized ABC-type transport system, ATPase component | | |
| COG4675 | 5 | Microcystin-dependent protein | | |
| COG4676 | 3 | Uncharacterized protein conserved in bacteria | | |
| COG4677 | 2 | Pectin methylesterase | | |
| COG4678 | 1 | Muramidase (phage lambda lysozyme) | | |
| COG4679 | 8 | Phage-related protein | | |
| COG4680 | 1 | Uncharacterized protein conserved in bacteria | | |
| COG4681 | 4 | Uncharacterized protein conserved in bacteria | | |
| COG4682 | 1 | Predicted membrane protein | | |
| COG4683 | 2 | Uncharacterized protein conserved in bacteria | | |
| COG4684 | 1 | Predicted membrane protein | | |
| COG4690 | 6 | Dipeptidase | | |
| COG4692 | 6 | Predicted neuraminidase (sialidase) | | |
| COG4695 | 5 | Phage-related protein | | |
| COG4704 | 1 | Uncharacterized protein conserved in bacteria | | |
| COG4705 | 6 | Uncharacterized membrane-anchored protein conserved in bacteria | | |
| COG4706 | 1 | Predicted 3-hydroxylacyl-(acyl carrier protein) dehydratase | | |
| COG4712 | 1 | Uncharacterized protein conserved in bacteria | | |
| COG4714 | 1 | Uncharacterized membrane-anchored protein conserved in bacteria | | |
| COG4715 | 2 | Uncharacterized conserved protein | | |
| COG4716 | 1 | Myosin-crossreactive antigen | | |
| COG4717 | 1 | Uncharacterized conserved protein | | |
| COG4719 | 3 | Uncharacterized protein conserved in bacteria | | |
| COG4725 | 1 | Transcriptional activator, adenine-specific DNA methyltransferase | | |
| COG4726 | 3 | Tfp pilus assembly protein PilX | | |
| COG4727 | 1 | Uncharacterized protein conserved in bacteria | | |
| COG4728 | 1 | Uncharacterized protein conserved in bacteria | | |
| COG4729 | 1 | Uncharacterized conserved protein | | |
| COG4731 | 3 | Uncharacterized protein conserved in bacteria | | |
| COG4736 | 1 | Cbb3-type cytochrome oxidase, subunit 3 | | |
| COG4741 | 1 | Predicted secreted endonuclease distantly related to archaeal Holliday junction resolvase | | |
| COG4745 | 5 | Predicted membrane-bound mannosyltransferase | | |
| COG4747 | 7 | ACT domain-containing protein | | |
| COG4749 | 1 | Uncharacterized protein conserved in archaea | | |

| | | | | |
|---|---|---|---|---|
| COG4752 | 1 | Uncharacterized protein conserved in bacteria | | |
| COG4753 | 5 | Response regulator containing CheY-like receiver domain and AraC-type DNA-binding domain | | |
| COG4754 | 1 | Uncharacterized conserved protein | | |
| COG4756 | 1 | Predicted cation transporter | | |
| COG4757 | 2 | Predicted alpha/beta hydrolase | | |
| COG4759 | 1 | Uncharacterized protein conserved in bacteria containing thioredoxin-like domain | | |
| COG4760 | 1 | Predicted membrane protein | | |
| COG4762 | 1 | Uncharacterized protein conserved in bacteria | | |
| COG4769 | 2 | Predicted membrane protein | | |
| COG4770 | 38 | Acetyl/propionyl-CoA carboxylase, alpha subunit | | |
| COG4771 | 5 | Outer membrane receptor for ferrienterochelin and colicins | | |
| COG4772 | 4 | Outer membrane receptor for Fe3+-dicitrate | | |
| COG4775 | 59 | Outer membrane protein/protective antigen OMA87 | | |
| COG4778 | 2 | ABC-type phosphonate transport system, ATPase component | | |
| COG4779 | 2 | ABC-type enterobactin transport system, permease component | | |
| COG4782 | 3 | Uncharacterized protein conserved in bacteria | | |
| COG4783 | 26 | Putative Zn-dependent protease, contains TPR repeats | | |
| COG4784 | 18 | Putative Zn-dependent protease | | |
| COG4785 | 2 | Lipoprotein NlpI, contains TPR repeats | | |
| COG4786 | 16 | Flagellar basal body rod protein | | |
| COG4787 | 1 | Flagellar basal body rod protein | | |
| COG4789 | 2 | Type III secretory pathway, component EscV | | |
| COG4792 | 1 | Type III secretory pathway, component EscU | | |
| COG4795 | 2 | Type II secretory pathway, component PulJ | | |
| COG4796 | 49 | Type II secretory pathway, component HofQ | | |
| COG4798 | 5 | Predicted methyltransferase | | |
| COG4799 | 90 | Acetyl-CoA carboxylase, carboxyltransferase component (subunits alpha and beta) | | |
| COG4802 | 2 | Ferredoxin-thioredoxin reductase, catalytic subunit | | |
| COG4803 | 1 | Predicted membrane protein | | |
| COG4804 | 4 | Uncharacterized conserved protein | | |
| COG4805 | 37 | Uncharacterized protein conserved in bacteria | | |
| COG4806 | 7 | L-rhamnose isomerase | | |
| COG4813 | 1 | Trehalose utilization protein | | |
| COG4819 | 11 | Ethanolamine utilization protein, possible chaperonin protecting lyase from inhibition | | |
| COG4821 | 5 | Uncharacterized protein containing SIS (Sugar ISomerase) phosphosugar binding domain | | |
| COG4825 | 7 | Uncharacterized membrane-anchored protein conserved in bacteria | | |
| COG4826 | 11 | Serine protease inhibitor | | |
| COG4829 | 4 | Muconolactone delta-isomerase | | |
| COG4830 | 1 | Ribosomal protein S26 | | |
| COG4832 | 3 | Uncharacterized conserved protein | | |
| COG4833 | 1 | Predicted glycosyl hydrolase | | |
| COG4834 | 1 | Uncharacterized protein conserved in bacteria | | |
| COG4842 | 3 | Uncharacterized protein conserved in bacteria | | |
| COG4843 | 1 | Uncharacterized protein conserved in bacteria | | |
| COG4845 | 3 | Chloramphenicol O-acetyltransferase | | |
| COG4850 | 3 | Uncharacterized conserved protein | | |
| COG4856 | 1 | Uncharacterized protein conserved in bacteria | | |
| COG4861 | 2 | Uncharacterized protein conserved in bacteria | | |
| COG4864 | 1 | Uncharacterized protein conserved in bacteria | | |
| COG4865 | 4 | Glutamate mutase epsilon subunit | | |
| COG4867 | 19 | Uncharacterized protein with a von Willebrand factor type A (vWA) domain | | |
| COG4868 | 2 | Uncharacterized protein conserved in bacteria | | |
| COG4869 | 1 | Propanediol utilization protein | | |
| COG4870 | 10 | Cysteine protease | | |
| COG4874 | 1 | Uncharacterized protein conserved in bacteria containing a pentein-type domain | | |
| COG4877 | 2 | Uncharacterized protein conserved in bacteria | | |
| COG4881 | 4 | Predicted membrane protein | | |
| COG4883 | 1 | Uncharacterized protein conserved in archaea | | |
| COG4886 | 1 | Leucine-rich repeat (LRR) protein | | |
| COG4887 | 4 | Uncharacterized metal-binding protein conserved in archaea | | |
| COG4889 | 1 | Predicted helicase | | |
| COG4894 | 3 | Uncharacterized conserved protein | | |
| COG4898 | 1 | Uncharacterized protein conserved in bacteria | | |
| COG4901 | 1 | Ribosomal protein S25 | | |
| COG4907 | 1 | Predicted membrane protein | | |
| COG4908 | 1 | Uncharacterized protein containing a NRPS condensation (elongation) domain | | |
| COG4909 | 4 | Propanediol dehydratase, large subunit | | |
| COG4911 | 10 | Uncharacterized conserved protein | | |
| COG4912 | 4 | Predicted DNA alkylation repair enzyme | | |
| COG4913 | 1 | Uncharacterized protein conserved in bacteria | | |
| COG4922 | 2 | Uncharacterized protein conserved in bacteria | | |
| COG4924 | 2 | Uncharacterized protein conserved in bacteria | | |
| COG4928 | 2 | Predicted P-loop ATPase | | |
| COG4929 | 1 | Uncharacterized membrane-anchored protein | | |
| COG4930 | 3 | Predicted ATP-dependent Lon-type protease | | |
| COG4934 | 19 | Predicted protease | | |
| COG4935 | 2 | Regulatory P domain of the subtilisin-like proprotein convertases and other proteases | | |
| COG4938 | 1 | Uncharacterized conserved protein | | |
| COG4941 | 52 | Predicted RNA polymerase sigma factor containing a TPR repeat domain | | |
| COG4942 | 6 | Membrane-bound metallopeptidase | | |
| COG4945 | 2 | Membrane-anchored protein predicted to be involved in regulation of amylopullulanase | | |
| COG4946 | 22 | Uncharacterized protein related to the periplasmic component of the Tol biopolymer transport system | | |
| COG4947 | 10 | Uncharacterized protein conserved in bacteria | | |
| COG4948 | 165 | L-alanine-DL-glutamate epimerase and related enzymes of enolase superfamily | | |

| | | | | |
|---|---|---|---|---|
| COG4951 | 1 | Uncharacterized protein conserved in bacteria | | |
| COG4952 | 3 | Predicted sugar isomerase | | |
| COG4953 | 18 | Membrane carboxypeptidase/penicillin-binding protein PbpC | | |
| COG4956 | 5 | Integral membrane protein (PIN domain superfamily) | | |
| COG4957 | 6 | Predicted transcriptional regulator | | |
| COG4959 | 1 | Type IV secretory pathway, protease TraF | | |
| COG4960 | 1 | Flp pilus assembly protein, protease CpaA | | |
| COG4961 | 1 | Flp pilus assembly protein TadG | | |
| COG4962 | 41 | Flp pilus assembly protein, ATPase CpaF | | |
| COG4963 | 6 | Flp pilus assembly protein, ATPase CpaE | | |
| COG4964 | 15 | Flp pilus assembly protein, secretin CpaC | | |
| COG4965 | 16 | Flp pilus assembly protein TadB | | |
| COG4966 | 4 | Tfp pilus assembly protein PilW | | |
| COG4967 | 2 | Tfp pilus assembly protein PilV | | |
| COG4968 | 1 | Tfp pilus assembly protein PilE | | |
| COG4969 | 6 | Tfp pilus assembly protein, major pilin PilA | | |
| COG4970 | 6 | Tfp pilus assembly protein FimT | | |
| COG4972 | 35 | Tfp pilus assembly protein, ATPase PilM | | |
| COG4973 | 20 | Site-specific recombinase XerC | | |
| COG4974 | 53 | Site-specific recombinase XerD | | |
| COG4976 | 5 | Predicted methyltransferase (contains TPR repeat) | | |
| COG4977 | 20 | Transcriptional regulator containing an amidase domain and an AraC-type DNA-binding HTH domain | | |
| COG4978 | 2 | Transcriptional regulator, effector-binding domain/component | | |
| COG4984 | 1 | Predicted membrane protein | | |
| COG4985 | 1 | ABC-type phosphate transport system, auxiliary component | | |
| COG4986 | 6 | ABC-type anion transport system, duplicated permease component | | |
| COG4987 | 2 | ABC-type transport system involved in cytochrome bd biosynthesis, fused ATPase and permease components | | |
| COG4989 | 2 | Predicted oxidoreductase | | |
| COG4992 | 57 | Ornithine/acetylornithine aminotransferase | | |
| COG4993 | 98 | Glucose dehydrogenase | | |
| COG4994 | 1 | Uncharacterized protein conserved in bacteria | | |
| COG4995 | 17 | Uncharacterized protein conserved in bacteria | | |
| COG5000 | 35 | Signal transduction histidine kinase involved in nitrogen fixation and metabolism regulation | | |
| COG5001 | 15 | Predicted signal transduction protein containing a membrane domain, an EAL and a GGDEF domain | | |
| COG5002 | 10 | Signal transduction histidine kinase | | |
| COG5006 | 6 | Predicted permease, DMT superfamily | | |
| COG5007 | 4 | Predicted transcriptional regulator, BolA superfamily | | |
| COG5008 | 13 | Tfp pilus assembly protein, ATPase PilU | | |
| COG5009 | 65 | Membrane carboxypeptidase/penicillin-binding protein | | |
| COG5011 | 6 | Uncharacterized protein conserved in bacteria | | |
| COG5012 | 10 | Predicted cobalamin binding protein | | |
| COG5013 | 23 | Nitrate reductase alpha subunit | | |
| COG5016 | 13 | Pyruvate/oxaloacetate carboxyltransferase | | |
| COG5021 | 1 | Ubiquitin-protein ligase | | |
| COG5022 | 1 | Myosin heavy chain | | |
| COG5023 | 1 | Tubulin | | |
| COG5045 | 1 | Ribosomal protein S10E | | |
| COG5048 | 8 | FOG: Zn-finger | | |
| COG5108 | 1 | Mitochondrial DNA-directed RNA polymerase | | |
| COG5126 | 1 | Ca2+-binding protein (EF-Hand superfamily) | | |
| COG5159 | 1 | 26S proteasome regulatory complex component | | |
| COG5163 | 1 | Protein required for biogenesis of the 60S ribosomal subunit | | |
| COG5171 | 2 | Ran GTPase-activating protein (Ran-binding protein) | | |
| COG5181 | 1 | U2 snRNP spliceosome subunit | | |
| COG5184 | 24 | Alpha-tubulin suppressor and related RCC1 domain-containing proteins | | |
| COG5192 | 1 | GTP-binding protein required for 40S ribosome biogenesis | | |
| COG5212 | 2 | Low-affinity cAMP phosphodiesterase | | |
| COG5239 | 1 | mRNA deadenylase, exonuclease subunit and related nucleases | | |
| COG5256 | 2 | Translation elongation factor EF-1alpha (GTPase) | | |
| COG5262 | 2 | Histone H2A | | |
| COG5263 | 1 | FOG: Glucan-binding domain (YG repeat) | | |
| COG5265 | 17 | ABC-type transport system involved in Fe-S cluster assembly, permease and ATPase components | | |
| COG5267 | 24 | Uncharacterized protein conserved in bacteria | | |
| COG5272 | 1 | Ubiquitin | | |
| COG5276 | 33 | Uncharacterized conserved protein | | |
| COG5277 | 2 | Actin and related proteins | | |
| COG5278 | 11 | Predicted periplasmic ligand-binding sensor domain | | |
| COG5280 | 1 | Phage-related minor tail protein | | |
| COG5282 | 14 | Uncharacterized conserved protein | | |
| COG5285 | 4 | Protein involved in biosynthesis of mitomycin antibiotics/polyketide fumonisin | | |
| COG5297 | 4 | Cellobiohydrolase A (1,4-beta-cellobiosidase A) | | |
| COG5298 | 3 | Uncharacterized protein conserved in bacteria | | |
| COG5304 | 1 | Uncharacterized protein conserved in bacteria | | |
| COG5305 | 16 | Predicted membrane protein | | |
| COG5309 | 4 | Exo-beta-1,3-glucanase | | |
| COG5310 | 4 | Homospermidine synthase | | |
| COG5316 | 20 | Uncharacterized conserved protein | | |
| COG5317 | 1 | Uncharacterized protein conserved in bacteria | | |
| COG5319 | 3 | Uncharacterized protein conserved in bacteria | | |
| COG5322 | 5 | Predicted dehydrogenase | | |
| COG5323 | 4 | Uncharacterized conserved protein | | |
| COG5324 | 1 | Uncharacterized conserved protein | | |
| COG5328 | 1 | Uncharacterized protein conserved in bacteria | | |
| COG5331 | 1 | Uncharacterized protein conserved in bacteria | | |

| | | | | |
|---|---|---|---|---|
| COG5336 | 2 | Uncharacterized protein conserved in bacteria | | |
| COG5337 | 2 | Spore coat assembly protein | | |
| COG5340 | 3 | Predicted transcriptional regulator | | |
| COG5342 | 1 | Invasion protein B, involved in pathogenesis | | |
| COG5346 | 1 | Predicted membrane protein | | |
| COG5347 | 1 | GTPase-activating protein that regulates ARFs (ADP-ribosylation factors), involved in ARF-mediated vesicular transport | | |
| COG5350 | 1 | Predicted protein tyrosine phosphatase | | |
| COG5351 | 3 | Uncharacterized protein conserved in bacteria | | |
| COG5352 | 3 | Uncharacterized protein conserved in bacteria | | |
| COG5360 | 2 | Uncharacterized protein conserved in bacteria | | |
| COG5361 | 8 | Uncharacterized conserved protein | | |
| COG5362 | 3 | Phage-related terminase | | |
| COG5368 | 10 | Uncharacterized protein conserved in bacteria | | |
| COG5373 | 10 | Predicted membrane protein | | |
| COG5377 | 1 | Phage-related protein, predicted endonuclease | | |
| COG5383 | 1 | Uncharacterized protein conserved in bacteria | | |
| COG5385 | 1 | Uncharacterized protein conserved in bacteria | | |
| COG5389 | 1 | Uncharacterized protein conserved in bacteria | | |
| COG5394 | 9 | Uncharacterized protein conserved in bacteria | | |
| COG5395 | 4 | Predicted membrane protein | | |
| COG5403 | 2 | Uncharacterized conserved protein | | |
| COG5405 | 10 | ATP-dependent protease HslVU (ClpYQ), peptidase subunit | | |
| COG5410 | 1 | Uncharacterized protein conserved in bacteria | | |
| COG5412 | 1 | Phage-related protein | | |
| COG5421 | 7 | Transposase | | |
| COG5424 | 6 | Pyrroloquinoline quinone (Coenzyme PQQ) biosynthesis protein C | | |
| COG5426 | 2 | Uncharacterized membrane protein | | |
| COG5427 | 6 | Uncharacterized membrane protein | | |
| COG5429 | 3 | Uncharacterized secreted protein | | |
| COG5430 | 9 | Uncharacterized secreted protein | | |
| COG5433 | 21 | Transposase | | |
| COG5434 | 7 | Endopolygalacturonase | | |
| COG5438 | 4 | Predicted multitransmembrane protein | | |
| COG5441 | 4 | Uncharacterized conserved protein | | |
| COG5442 | 1 | Flagellar biosynthesis regulator FlaF | | |
| COG5443 | 1 | Flagellar biosynthesis regulator FlbT | | |
| COG5448 | 1 | Uncharacterized conserved protein | | |
| COG5449 | 1 | Uncharacterized conserved protein | | |
| COG5450 | 1 | Transcription regulator of the Arc/MetJ class | | |
| COG5452 | 1 | Uncharacterized conserved protein | | |
| COG5459 | 1 | Predicted rRNA methylase | | |
| COG5462 | 1 | Predicted secreted (periplasmic) protein | | |
| COG5465 | 2 | Uncharacterized conserved protein | | |
| COG5466 | 1 | Predicted small metal-binding protein | | |
| COG5468 | 1 | Predicted secreted (periplasmic) protein | | |
| COG5470 | 12 | Uncharacterized conserved protein | | |
| COG5472 | 2 | Predicted small integral membrane protein | | |
| COG5473 | 5 | Predicted integral membrane protein | | |
| COG5476 | 20 | Uncharacterized conserved protein | | |
| COG5478 | 4 | Predicted small integral membrane protein | | |
| COG5479 | 14 | Uncharacterized protein potentially involved in peptidoglycan biosynthesis | | |
| COG5483 | 4 | Uncharacterized conserved protein | | |
| COG5485 | 16 | Predicted ester cyclase | | |
| COG5486 | 11 | Predicted metal-binding integral membrane protein | | |
| COG5488 | 6 | Integral membrane protein | | |
| COG5490 | 1 | Uncharacterized conserved protein | | |
| COG5492 | 6 | Bacterial surface proteins containing Ig-like domains | | |
| COG5495 | 8 | Uncharacterized conserved protein | | |
| COG5496 | 9 | Predicted thioesterase | | |
| COG5499 | 5 | Predicted transcription regulator containing HTH domain | | |
| COG5501 | 3 | Predicted secreted protein | | |
| COG5507 | 5 | Uncharacterized conserved protein | | |
| COG5511 | 6 | Bacteriophage capsid protein | | |
| COG5512 | 3 | Zn-ribbon-containing, possibly RNA-binding protein and truncated derivatives | | |
| COG5513 | 1 | Predicted secreted protein | | |
| COG5514 | 3 | Uncharacterized conserved protein | | |
| COG5516 | 4 | Conserved protein containing a Zn-ribbon-like motif, possibly RNA-binding | | |
| COG5517 | 9 | Small subunit of phenylpropionate dioxygenase | | |
| COG5518 | 1 | Bacteriophage capsid portal protein | | |
| COG5520 | 3 | O-Glycosyl hydrolase | | |
| COG5522 | 4 | Predicted integral membrane protein | | |
| COG5525 | 12 | Bacteriophage tail assembly protein | | |
| COG5526 | 2 | Uncharacterized conserved protein | | |
| COG5528 | 2 | Predicted integral membrane protein | | |
| COG5531 | 1 | SWIB-domain-containing proteins implicated in chromatin remodeling | | |
| COG5542 | 1 | Predicted integral membrane protein | | |
| COG5545 | 1 | Predicted P-loop ATPase and inactivated derivatives | | |
| COG5549 | 1 | Predicted Zn-dependent protease | | |
| COG5552 | 4 | Uncharacterized conserved protein | | |
| COG5553 | 11 | Predicted metal-dependent enzyme of the double-stranded beta helix superfamily | | |
| COG5557 | 44 | Polysulphide reductase | | |
| COG5563 | 12 | Predicted integral membrane proteins containing uncharacterized repeats | | |
| COG5564 | 2 | Predicted TIM-barrel enzyme, possibly a dioxygenase | | |
| COG5569 | 4 | Uncharacterized conserved protein | | |

| | | | | |
|---|---|---|---|---|
| COG5572 | 1 | Predicted integral membrane protein | | |
| COG5573 | 3 | Predicted nucleic-acid-binding protein, contains PIN domain | | |
| COG5576 | 1 | Homeodomain-containing transcription factor | | |
| COG5579 | 3 | Uncharacterized conserved protein | | |
| COG5581 | 2 | Predicted glycosyltransferase | | |
| COG5583 | 1 | Uncharacterized small protein | | |
| COG5585 | 1 | NAD+--asparagine ADP-ribosyltransferase | | |
| COG5587 | 6 | Uncharacterized conserved protein | | |
| COG5588 | 13 | Uncharacterized conserved protein | | |
| COG5590 | 1 | Uncharacterized conserved protein | | |
| COG5591 | 3 | Uncharacterized conserved protein | | |
| COG5592 | 3 | Uncharacterized conserved protein | | |
| COG5598 | 22 | Trimethylamine:corrinoid methyltransferase | | |
| COG5605 | 2 | Predicted small integral membrane protein | | |
| COG5606 | 2 | Uncharacterized conserved small protein | | |
| COG5607 | 4 | Uncharacterized conserved protein | | |
| COG5609 | 2 | Uncharacterized conserved protein | | |
| COG5611 | 1 | Predicted nucleic-acid-binding protein, contains PIN domain | | |
| COG5615 | 1 | Predicted integral membrane protein | | |
| COG5616 | 71 | Predicted integral membrane protein | | |
| COG5619 | 1 | Uncharacterized conserved protein | | |
| COG5621 | 12 | Predicted secreted hydrolase | | |
| COG5632 | 1 | N-acetylmuramoyl-L-alanine amidase | | |
| COG5635 | 13 | Predicted NTPase (NACHT family) | | |
| COG5637 | 9 | Predicted integral membrane protein | | |
| COG5640 | 9 | Secreted trypsin-like serine protease | | |
| COG5642 | 2 | Uncharacterized conserved protein | | |
| COG5646 | 5 | Uncharacterized conserved protein | | |
| COG5649 | 6 | Uncharacterized conserved protein | | |
| COG5650 | 3 | Predicted integral membrane protein | | |
| COG5651 | 1 | PPE-repeat proteins | | |
| COG5652 | 5 | Predicted integral membrane protein | | |
| COG5653 | 2 | Protein involved in cellulose biosynthesis (CelD) | | |
| COG5654 | 4 | Uncharacterized conserved protein | | |
| COG5655 | 1 | Plasmid rolling circle replication initiator protein and truncated derivatives | | |
| COG5658 | 1 | Predicted integral membrane protein | | |
| COG5659 | 30 | FOG: Transposase | | |
| COG5660 | 3 | Predicted integral membrane protein | | |
| COG5662 | 12 | Predicted transmembrane transcriptional regulator (anti-sigma factor) | | |

| Functional categories | Sequence count | COG categories |
|---|---|---|
| A | RNA processing and modification | 18 |
| B | Chromatin structure and dynamics | 50 |
| C | Energy production and conversion | 7775 |
| D | Cell cycle control, cell division, chromosome partitioning | 692 |
| E | Amino acid transport and metabolism | 9706 |
| F | Nucleotide transport and metabolism | 1913 |
| G | Carbohydrate transport and metabolism | 5688 |
| H | Coenzyme transport and metabolism | 3083 |
| I | Lipid transport and metabolism | 4336 |
| J | Translation, ribosomal structure and biogenesis | 4051 |
| K | Transcription | 3471 |
| L | Replication, recombination and repair | 4751 |
| M | Cell wall/membrane/envelope biogenesis | 5688 |
| N | Cell motility | 1097 |
| O | Posttranslational modification, protein turnover, chaperones | 3454 |
| P | Inorganic ion transport and metabolism | 3709 |
| Q | Secondary metabolites biosynthesis, transport and catabolism | 2264 |
| R | General function prediction only | 9923 |

| | | |
|---|---|---|
| S | Function unknown | 6105 |
| T | Signal transduction mechanisms | 4559 |
| U | Intracellular trafficking, secretion, and vesicular transport | 841 |
| V | Defense mechanisms | 1888 |
| Z | Cytoskeleton | 4 |

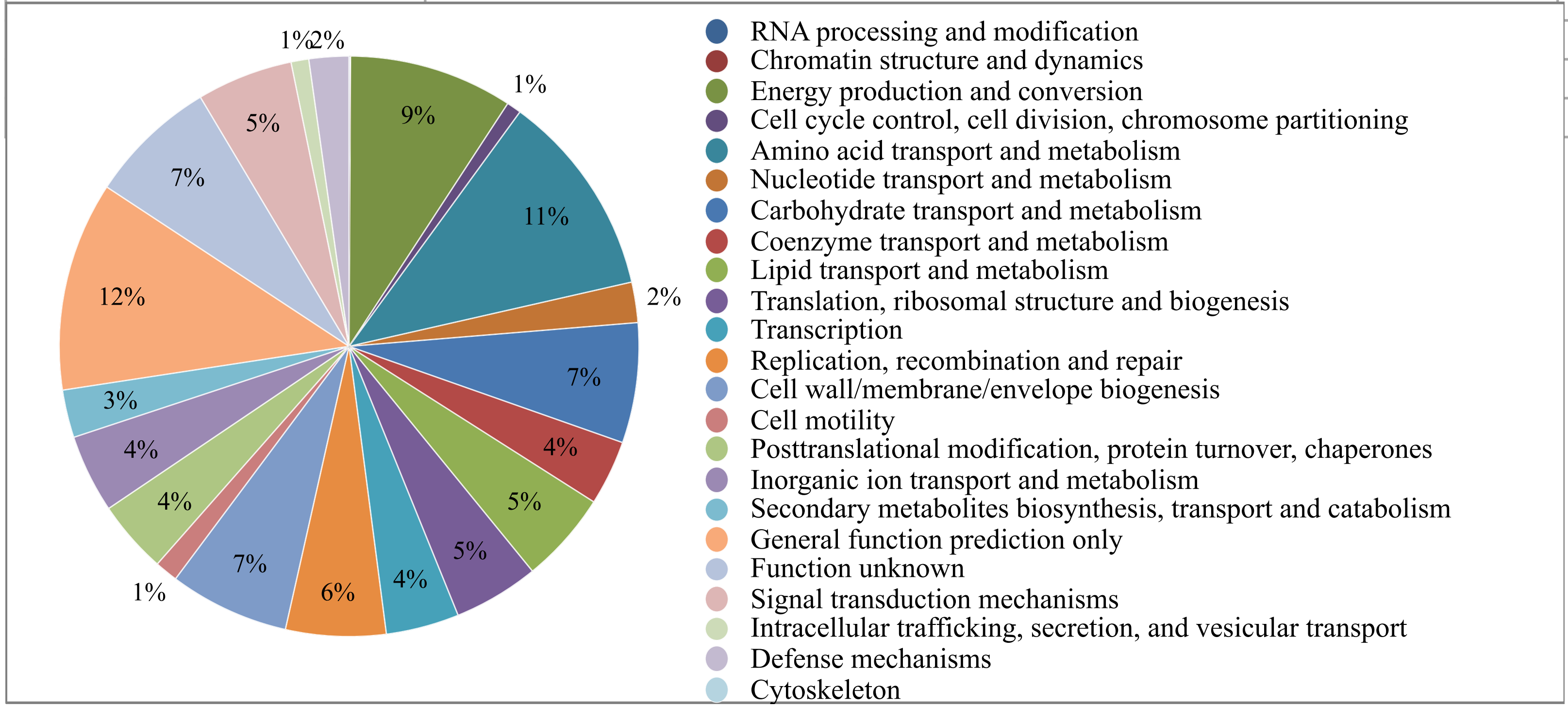

| | | | | | | | | | | | | |
|---|---|---|---|---|---|---|---|---|---|---|---|---|
| | | | | | | | | | | | | |
| | | | | | | | | | | | | |
| | | | | | | | | | | | | |
| | | | | | | | | | | | | |
| | | | | | | | | | | | | |
| | | | | | | | | | | | | |
| | | | | | | | | | | | | |
| | | | | | | | | | | | | |
| | | | | | | | | | | | | |
| | | | | | | | | | | | | |
| | | | | | | | | | | | | |
| | | | | | | | | | | | | |
| | | | | | | | | | | | | |

| |
|---|
| |
| |
| |
| |
| |
| |
| |
| |
| |
| |
| |
| |
| |

| | | | | |
|---|---|---|---|---|
| K00001 | 181 | alcohol dehydrogenase [EC:1.1.1.1] | | |
| K00002 | 3 | alcohol dehydrogenase (NADP+) [EC:1.1.1.2] | | |
| K00003 | 74 | homoserine dehydrogenase [EC:1.1.1.3] | | |
| K00004 | 3 | (R,R)-butanediol dehydrogenase / diacetyl reductase [EC:1.1.1.4 | | |
| K00005 | 10 | glycerol dehydrogenase [EC:1.1.1.6] | | |
| K00008 | 37 | L-iditol 2-dehydrogenase [EC:1.1.1.14] | | |
| K00009 | 4 | mannitol-1-phosphate 5-dehydrogenase [EC:1.1.1.17] | | |
| K00010 | 96 | myo-inositol 2-dehydrogenase [EC:1.1.1.18] | | |
| K00011 | 4 | aldehyde reductase [EC:1.1.1.21] | | |
| K00012 | 45 | UDPglucose 6-dehydrogenase [EC:1.1.1.22] | | |
| K00013 | 43 | histidinol dehydrogenase [EC:1.1.1.23] | | |
| K00014 | 37 | shikimate dehydrogenase [EC:1.1.1.25] | | |
| K00015 | 39 | glyoxylate reductase [EC:1.1.1.26] | | |
| K00016 | 26 | L-lactate dehydrogenase [EC:1.1.1.27] | | |
| K00018 | 11 | glycerate dehydrogenase [EC:1.1.1.29] | | |
| K00019 | 33 | 3-hydroxybutyrate dehydrogenase [EC:1.1.1.30] | | |
| K00020 | 92 | 3-hydroxyisobutyrate dehydrogenase [EC:1.1.1.31] | | |
| K00021 | 14 | 3-hydroxy-3-methylglutaryl-CoA reductase [EC:1.1.1.34] | | |
| K00023 | 24 | acetoacetyl-CoA reductase [EC:1.1.1.36] | | |
| K00024 | 35 | malate dehydrogenase [EC:1.1.1.37] | | |
| K00027 | 40 | malate dehydrogenase (oxaloacetate-decarboxylating) [EC:1.1.1.38] | | |
| K00028 | 4 | malate dehydrogenase (decarboxylating) [EC:1.1.1.39] | | |
| K00029 | 39 | malate dehydrogenase (oxaloacetate-decarboxylating)(NADP+) | | |
| K00030 | 19 | isocitrate dehydrogenase (NAD+) [EC:1.1.1.41] | | |
| K00031 | 37 | isocitrate dehydrogenase [EC:1.1.1.42] | | |
| K00032 | 1 | phosphogluconate 2-dehydrogenase [EC:1.1.1.43] | | |
| K00033 | 37 | 6-phosphogluconate dehydrogenase [EC:1.1.1.44] | | |
| K00034 | 25 | glucose 1-dehydrogenase [EC:1.1.1.47] | | |
| K00035 | 1 | D-galactose 1-dehydrogenase [EC:1.1.1.48] | | |
| K00036 | 118 | glucose-6-phosphate 1-dehydrogenase [EC:1.1.1.49] | | |
| K00037 | 2 | 3-alpha-hydroxysteroid dehydrogenase [EC:1.1.1.50] | | |
| K00038 | 6 | 3alpha(or 20beta)-hydroxysteroid dehydrogenase [EC:1.1.1.53] | | |
| K00040 | 4 | fructuronate reductase [EC:1.1.1.57] | | |
| K00042 | 60 | 2-hydroxy-3-oxopropionate reductase [EC:1.1.1.60] | | |
| K00043 | 6 | 4-hydroxybutyrate dehydrogenase [EC:1.1.1.61] | | |
| K00044 | 10 | estradiol 17beta-dehydrogenase [EC:1.1.1.62] | | |
| K00045 | 4 | mannitol 2-dehydrogenase [EC:1.1.1.67] | | |
| K00046 | 33 | gluconate 5-dehydrogenase [EC:1.1.1.69] | | |
| K00048 | 20 | lactaldehyde reductase [EC:1.1.1.77] | | |
| K00050 | 46 | hydroxypyruvate reductase [EC:1.1.1.81] | | |
| K00051 | 10 | malate dehydrogenase (NADP+) [EC:1.1.1.82] | | |
| K00052 | 66 | 3-isopropylmalate dehydrogenase [EC:1.1.1.85] | | |
| K00053 | 17 | ketol-acid reductoisomerase [EC:1.1.1.86] | | |
| K00054 | 7 | hydroxymethylglutaryl-CoA reductase [EC:1.1.1.88] | | |
| K00055 | 2 | aryl-alcohol dehydrogenase [EC:1.1.1.90] | | |
| K00057 | 38 | glycerol-3-phosphate dehydrogenase (NAD(P)+) [EC:1.1.1.94] | | |
| K00058 | 145 | D-3-phosphoglycerate dehydrogenase [EC:1.1.1.95] | | |
| K00059 | 312 | 3-oxoacyl-[acyl-carrier protein] reductase [EC:1.1.1.100] | | |
| K00060 | 50 | threonine 3-dehydrogenase [EC:1.1.1.103] | | |
| K00064 | 7 | D-threo-aldose 1-dehydrogenase [EC:1.1.1.122] | | |

| | | | | |
|---|---|---|---|---|
| K00065 | 29 | 2-deoxy-D-gluconate 3-dehydrogenase [EC:1.1.1.125] | | |
| K00066 | 25 | GDP-mannose 6-dehydrogenase [EC:1.1.1.132] | | |
| K00067 | 36 | dTDP-4-dehydrorhamnose reductase [EC:1.1.1.133] | | |
| K00068 | 7 | sorbitol-6-phosphate 2-dehydrogenase [EC:1.1.1.140] | | |
| K00071 | 3 | 11beta-hydroxysteroid dehydrogenase [EC:1.1.1.146] | | |
| K00073 | 35 | ureidoglycolate dehydrogenase [EC:1.1.1.154] | | |
| K00074 | 63 | 3-hydroxybutyryl-CoA dehydrogenase [EC:1.1.1.157] | | |
| K00075 | 37 | UDP-N-acetylmuramate dehydrogenase [EC:1.1.1.158] | | |
| K00076 | 5 | 7-alpha-hydroxysteroid dehydrogenase [EC:1.1.1.159] | | |
| K00077 | 44 | 2-dehydropantoate 2-reductase [EC:1.1.1.169] | | |
| K00079 | 12 | carbonyl reductase (NADPH) [EC:1.1.1.184] | | |
| K00082 | 9 | 5-amino-6-(5-phosphoribosylamino)uracil reductase [EC:1.1.1.193] | | |
| K00083 | 2 | cinnamyl-alcohol dehydrogenase [EC:1.1.1.195] | | |
| K00086 | 5 | 1,3-propanediol dehydrogenase [EC:1.1.1.202] | | |
| K00087 | 105 | xanthine dehydrogenase molybdenum-binding subunit [EC:1.17.1.4] | | |
| K00088 | 84 | IMP dehydrogenase [EC:1.1.1.205] | | |
| K00090 | 13 | gluconate 2-dehydrogenase [EC:1.1.1.215] | | |
| K00091 | 22 | dihydroflavonol-4-reductase [EC:1.1.1.219] | | |
| K00094 | 1 | galactitol-1-phosphate 5-dehydrogenase [EC:1.1.1.251] | | |
| K00096 | 3 | glycerol-1-phosphate dehydrogenase [NAD(P)] [EC:1.1.1.261] | | |
| K00097 | 42 | 4-hydroxythreonine-4-phosphate dehydrogenase [EC:1.1.1.262] | | |
| K00098 | 4 | L-idonate 5-dehydrogenase [EC:1.1.1.264] | | |
| K00099 | 51 | 1-deoxy-D-xylulose-5-phosphate reductoisomerase [EC:1.1.1.267] | | |
| K00100 | 439 | Unclassified; E1.1.1.- | | |
| K00101 | 50 | L-lactate dehydrogenase (cytochrome) [EC:1.1.2.3] | | |
| K00102 | 85 | D-lactate dehydrogenase (cytochrome) [EC:1.1.2.4] | | |
| K00103 | 10 | L-gulonolactone oxidase [EC:1.1.3.8] | | |
| K00104 | 91 | glycolate oxidase [EC:1.1.3.15] | | |
| K00105 | 3 | alpha-glycerophosphate oxidase [EC:1.1.3.21] | | |
| K00108 | 51 | choline dehydrogenase [EC:1.1.99.1] | | |
| K00109 | 14 | 2-hydroxyglutarate dehydrogenase [EC:1.1.99.2] | | |
| K00111 | 53 | glycerol-3-phosphate dehydrogenase [EC:1.1.5.3] | | |
| K00112 | 2 | glycerol-3-phosphate dehydrogenase subunit B [EC:1.1.5.3] | | |
| K00113 | 10 | glycerol-3-phosphate dehydrogenase subunit C [EC:1.1.5.3] | | |
| K00114 | 80 | alcohol dehydrogenase (acceptor) [EC:1.1.99.8] | | |
| K00116 | 24 | malate dehydrogenase (quinone) [EC:1.1.5.4] | | |
| K00117 | 55 | quinoprotein glucose dehydrogenase [EC:1.1.5.2] | | |
| K00118 | 12 | glucose-fructose oxidoreductase [EC:1.1.99.28] | | |
| K00119 | 103 | Unclassified; E1.1.99.- | | |
| K00120 | 82 | Unclassified; E1.1.-.- | | |
| K00121 | 49 | S-(hydroxymethyl)glutathione dehydrogenase / alcohol dehydrogenase | | |
| K00122 | 100 | formate dehydrogenase [EC:1.2.1.2] | | |
| K00123 | 164 | formate dehydrogenase, alpha subunit [EC:1.2.1.2] | | |
| K00124 | 24 | formate dehydrogenase, beta subunit [EC:1.2.1.2] | | |
| K00127 | 21 | formate dehydrogenase, gamma subunit [EC:1.2.1.2] | | |
| K00128 | 152 | aldehyde dehydrogenase (NAD+) [EC:1.2.1.3] | | |
| K00129 | 2 | aldehyde dehydrogenase (NAD(P)+) [EC:1.2.1.5] | | |
| K00130 | 60 | betaine-aldehyde dehydrogenase [EC:1.2.1.8] | | |
| K00131 | 21 | glyceraldehyde-3-phosphate dehydrogenase (NADP) [EC:1.2.1.9] | | |
| K00132 | 6 | acetaldehyde dehydrogenase (acetylating) [EC:1.2.1.10] | | |

| | | | | |
|---|---|---|---|---|
| K00133 | 35 | aspartate-semialdehyde dehydrogenase [EC:1.2.1.11] | | |
| K00134 | 29 | glyceraldehyde 3-phosphate dehydrogenase [EC:1.2.1.12] | | |
| K00135 | 94 | succinate-semialdehyde dehydrogenase (NADP+) [EC:1.2.1.16] | | |
| K00137 | 5 | aminobutyraldehyde dehydrogenase [EC:1.2.1.19] | | |
| K00138 | 6 | aldehyde dehydrogenase [EC:1.2.1.-] | | |
| K00140 | 32 | methylmalonate-semialdehyde dehydrogenase [EC:1.2.1.27] | | |
| K00141 | 6 | benzaldehyde dehydrogenase (NAD) [EC:1.2.1.28] | | |
| K00145 | 50 | N-acetyl-gamma-glutamyl-phosphate reductase [EC:1.2.1.38] | | |
| K00146 | 17 | phenylacetaldehyde dehydrogenase [EC:1.2.1.39] | | |
| K00147 | 25 | glutamate-5-semialdehyde dehydrogenase [EC:1.2.1.41] | | |
| K00148 | 5 | glutathione-independent formaldehyde dehydrogenase [EC:1.2.1.46] | | |
| K00150 | 2 | glyceraldehyde-3-phosphate dehydrogenase (NAD(P)) [EC:1.2.1.59] | | |
| K00151 | 15 | 5-carboxymethyl-2-hydroxymuconic-semialdehyde dehydrogenase | | |
| K00152 | 5 | salicylaldehyde dehydrogenase [EC:1.2.1.65] | | |
| K00153 | 7 | NAD/factor-dependent formaldehyde dehydrogenase [EC:1.2.1.66] | | |
| K00154 | 3 | coniferyl-aldehyde dehydrogenase [EC:1.2.1.68] | | |
| K00155 | 81 | Unclassified; E1.2.1.- | | |
| K00156 | 27 | pyruvate dehydrogenase (cytochrome) [EC:1.2.2.2] | | |
| K00157 | 22 | aldehyde oxidase [EC:1.2.3.1] | | |
| K00158 | 6 | pyruvate oxidase [EC:1.2.3.3] | | |
| K00161 | 65 | pyruvate dehydrogenase E1 component subunit alpha [EC:1.2.4.1] | | |
| K00162 | 66 | pyruvate dehydrogenase E1 component subunit beta [EC:1.2.4.1] | | |
| K00163 | 45 | pyruvate dehydrogenase E1 component [EC:1.2.4.1] | | |
| K00164 | 41 | 2-oxoglutarate dehydrogenase E1 component [EC:1.2.4.2] | | |
| K00166 | 32 | 2-oxoisovalerate dehydrogenase E1 component, alpha subunit | | |
| K00167 | 31 | 2-oxoisovalerate dehydrogenase E1 component, beta subunit | | |
| K00169 | 37 | pyruvate ferredoxin oxidoreductase, alpha subunit [EC:1.2.7.1] | | |
| K00170 | 17 | pyruvate ferredoxin oxidoreductase, beta subunit [EC:1.2.7.1] | | |
| K00171 | 15 | pyruvate ferredoxin oxidoreductase, delta subunit [EC:1.2.7.1] | | |
| K00172 | 16 | pyruvate ferredoxin oxidoreductase, gamma subunit [EC:1.2.7.1] | | |
| K00174 | 70 | 2-oxoglutarate ferredoxin oxidoreductase subunit alpha [EC:1.2.7.3] | | |
| K00175 | 41 | 2-oxoglutarate ferredoxin oxidoreductase subunit beta [EC:1.2.7.3] | | |
| K00176 | 10 | 2-oxoglutarate ferredoxin oxidoreductase subunit delta [EC:1.2.7.3] | | |
| K00177 | 12 | 2-oxoglutarate ferredoxin oxidoreductase subunit gamma [EC:1.2.7.3] | | |
| K00178 | 23 | oxidoreductase containing iron-sulfur protein [EC:1.2.7.-] | | |
| K00179 | 43 | indolepyruvate ferredoxin oxidoreductase, alpha subunit [EC:1.2.7.8] | | |
| K00180 | 23 | indolepyruvate ferredoxin oxidoreductase, beta subunit [EC:1.2.7.8] | | |
| K00183 | 52 | molybdopterin oxidoreductase, molybdopterin binding subunit | | |
| K00184 | 53 | molybdopterin oxidoreductase, iron-sulfur binding subunit | | |
| K00185 | 44 | molybdopterin oxidoreductase, membrane subunit [EC:1.2.7.-] | | |
| K00186 | 10 | 2-oxoisovalerate ferredoxin oxidoreductase, alpha subunit | | |
| K00187 | 7 | 2-oxoisovalerate ferredoxin oxidoreductase, beta subunit | | |
| K00191 | 7 | CO-methylating acetyl-CoA synthase [EC:2.3.1.169] | | |
| K00194 | 1 | carbon-monoxide dehydrogenase delta subunit [EC:1.2.99.2] | | |
| K00197 | 5 | carbon-monoxide dehydrogenase gamma subunit [EC:1.2.99.2] | | |
| K00198 | 3 | carbon-monoxide dehydrogenase catalytic subunit [EC:1.2.99.2] | | |
| K00200 | 8 | formylmethanofuran dehydrogenase subunit A [EC:1.2.99.5] | | |
| K00201 | 1 | formylmethanofuran dehydrogenase subunit B [EC:1.2.99.5] | | |
| K00202 | 2 | formylmethanofuran dehydrogenase subunit C [EC:1.2.99.5] | | |
| K00206 | 1 | Unclassified; E1.2.-.- | | |

| | | | | |
|---|---|---|---|---|
| K00207 | 10 | dihydropyrimidine dehydrogenase (NADP+) [EC:1.3.1.2] | | |
| K00208 | 71 | enoyl-[acyl-carrier protein] reductase I [EC:1.3.1.9] | | |
| K00209 | 3 | enoyl-[acyl-carrier-protein] reductase (NADPH2, B-specific) | | |
| K00210 | 23 | prephenate dehydrogenase [EC:1.3.1.12] | | |
| K00214 | 1 | biliverdin reductase [EC:1.3.1.24] | | |
| K00215 | 29 | dihydrodipicolinate reductase [EC:1.3.1.26] | | |
| K00216 | 2 | 2,3-dihydro-2,3-dihydroxybenzoate dehydrogenase [EC:1.3.1.28] | | |
| K00217 | 4 | maleylacetate reductase [EC:1.3.1.32] | | |
| K00218 | 9 | protochlorophyllide reductase [EC:1.3.1.33] | | |
| K00219 | 58 | 2,4-dienoyl-CoA reductase (NADPH2) [EC:1.3.1.34] | | |
| K00220 | 4 | cyclohexadienyl dehydrogenase [EC:1.3.1.43] | | |
| K00224 | 11 | Unclassified; E1.3.1.- | | |
| K00226 | 72 | dihydroorotate oxidase [EC:1.3.3.1] | | |
| K00227 | 3 | lathosterol oxidase [EC:1.14.21.6] | | |
| K00228 | 17 | coproporphyrinogen III oxidase [EC:1.3.3.3] | | |
| K00230 | 8 | protoporphyrinogen oxidase [EC:1.3.3.4] | | |
| K00231 | 27 | protoporphyrinogen oxidase [EC:1.3.3.4] | | |
| K00232 | 1 | acyl-CoA oxidase [EC:1.3.3.6] | | |
| K00239 | 70 | succinate dehydrogenase flavoprotein subunit [EC:1.3.99.1] | | |
| K00240 | 31 | succinate dehydrogenase iron-sulfur protein [EC:1.3.99.1] | | |
| K00241 | 21 | succinate dehydrogenase cytochrome b-556 subunit [EC:1.3.99.1] | | |
| K00242 | 5 | succinate dehydrogenase hydrophobic membrane anchor protein | | |
| K00244 | 39 | fumarate reductase flavoprotein subunit [EC:1.3.99.1] | | |
| K00245 | 7 | fumarate reductase iron-sulfur protein [EC:1.3.99.1] | | |
| K00246 | 13 | fumarate reductase subunit C [EC:1.3.99.1] | | |
| K00247 | 5 | fumarate reductase subunit D [EC:1.3.99.1] | | |
| K00248 | 107 | butyryl-CoA dehydrogenase [EC:1.3.99.2] | | |
| K00249 | 223 | acyl-CoA dehydrogenase [EC:1.3.99.3] | | |
| K00252 | 32 | glutaryl-CoA dehydrogenase [EC:1.3.99.7] | | |
| K00253 | 67 | isovaleryl-CoA dehydrogenase [EC:1.3.99.10] | | |
| K00255 | 14 | long-chain-acyl-CoA dehydrogenase [EC:1.3.99.13] | | |
| K00256 | 109 | isoquinoline 1-oxidoreductase [EC:1.3.99.16] | | |
| K00257 | 333 | Unclassified; E1.3.99.- | | |
| K00258 | 29 | Unclassified; E1.3.-.- | | |
| K00259 | 29 | alanine dehydrogenase [EC:1.4.1.1] | | |
| K00260 | 63 | glutamate dehydrogenase [EC:1.4.1.2] | | |
| K00261 | 38 | glutamate dehydrogenase (NAD(P)+) [EC:1.4.1.3] | | |
| K00262 | 26 | glutamate dehydrogenase (NADP+) [EC:1.4.1.4] | | |
| K00263 | 17 | leucine dehydrogenase [EC:1.4.1.9] | | |
| K00265 | 89 | glutamate synthase (NADPH/NADH) large chain [EC:1.4.1.13 1.4.1.14] | | |
| K00266 | 101 | glutamate synthase (NADPH/NADH) small chain [EC:1.4.1.13 1.4.1.14] | | |
| K00271 | 14 | valine dehydrogenase [EC:1.4.1.-] | | |
| K00273 | 15 | D-amino-acid oxidase [EC:1.4.3.3] | | |
| K00274 | 21 | monoamine oxidase [EC:1.4.3.4] | | |
| K00275 | 18 | pyridoxamine 5'-phosphate oxidase [EC:1.4.3.5] | | |
| K00276 | 60 | primary-amine oxidase [EC:1.4.3.21] | | |
| K00278 | 44 | L-aspartate oxidase [EC:1.4.3.16] | | |
| K00279 | 15 | cytokinin dehydrogenase [EC:1.5.99.12] | | |
| K00280 | 8 | Unclassified; E1.4.3.- | | |
| K00281 | 24 | glycine dehydrogenase [EC:1.4.4.2] | | |

| | | | | |
|---|---|---|---|---|
| K00282 | 30 | glycine dehydrogenase subunit 1 [EC:1.4.4.2] | | |
| K00283 | 25 | glycine dehydrogenase subunit 2 [EC:1.4.4.2] | | |
| K00284 | 28 | glutamate synthase (ferredoxin) [EC:1.4.7.1] | | |
| K00285 | 34 | D-amino-acid dehydrogenase [EC:1.4.99.1] | | |
| K00286 | 27 | pyrroline-5-carboxylate reductase [EC:1.5.1.2] | | |
| K00287 | 56 | dihydrofolate reductase [EC:1.5.1.3] | | |
| K00290 | 30 | saccharopine dehydrogenase (NAD+, L-lysine forming) [EC:1.5.1.7] | | |
| K00292 | 2 | saccharopine dehydrogenase (NAD+, L-glutamate forming) [EC:1.5.1.9] | | |
| K00294 | 69 | 1-pyrroline-5-carboxylate dehydrogenase [EC:1.5.1.12] | | |
| K00297 | 67 | methylenetetrahydrofolate reductase (NADPH) [EC:1.5.1.20] | | |
| K00299 | 34 | FMN reductase [EC:1.5.1.29] | | |
| K00301 | 35 | sarcosine oxidase [EC:1.5.3.1] | | |
| K00302 | 11 | sarcosine oxidase, subunit alpha [EC:1.5.3.1] | | |
| K00303 | 37 | sarcosine oxidase, subunit beta [EC:1.5.3.1] | | |
| K00304 | 2 | sarcosine oxidase, subunit delta [EC:1.5.3.1] | | |
| K00305 | 1 | sarcosine oxidase, subunit gamma [EC:1.5.3.1] | | |
| K00309 | 57 | Unclassified; E1.5.3.- | | |
| K00311 | 35 | electron-transferring-flavoprotein dehydrogenase [EC:1.5.5.1] | | |
| K00313 | 5 | electron transfer flavoprotein-quinone oxidoreductase [EC:1.5.5.-] | | |
| K00314 | 44 | sarcosine dehydrogenase [EC:1.5.99.1] | | |
| K00315 | 21 | dimethylglycine dehydrogenase [EC:1.5.99.2] | | |
| K00317 | 11 | trimethylamine dehydrogenase [EC:1.5.8.2] | | |
| K00318 | 21 | proline dehydrogenase [EC:1.5.99.8] | | |
| K00320 | 25 | coenzyme F420-dependent N5,N10-methenyltetrahydromethanopterin | | |
| K00321 | 1 | Unclassified; E1.5.99.- | | |
| K00322 | 12 | NAD(P) transhydrogenase [EC:1.6.1.1] | | |
| K00324 | 34 | NAD(P) transhydrogenase subunit alpha [EC:1.6.1.2] | | |
| K00325 | 18 | NAD(P) transhydrogenase subunit beta [EC:1.6.1.2] | | |
| K00326 | 1 | cytochrome-b5 reductase [EC:1.6.2.2] | | |
| K00327 | 1 | NADPH-ferrihemoprotein reductase [EC:1.6.2.4] | | |
| K00329 | 36 | NADH dehydrogenase [EC:1.6.5.3] | | |
| K00330 | 25 | NADH dehydrogenase I subunit A [EC:1.6.5.3] | | |
| K00331 | 27 | NADH dehydrogenase I subunit B [EC:1.6.5.3] | | |
| K00332 | 21 | NADH dehydrogenase I subunit C [EC:1.6.5.3] | | |
| K00333 | 64 | NADH dehydrogenase I subunit D [EC:1.6.5.3] | | |
| K00334 | 43 | NADH dehydrogenase I subunit E [EC:1.6.5.3] | | |
| K00335 | 80 | NADH dehydrogenase I subunit F [EC:1.6.5.3] | | |
| K00336 | 63 | NADH dehydrogenase I subunit G [EC:1.6.5.3] | | |
| K00337 | 55 | NADH dehydrogenase I subunit H [EC:1.6.5.3] | | |
| K00338 | 26 | NADH dehydrogenase I subunit I [EC:1.6.5.3] | | |
| K00339 | 20 | NADH dehydrogenase I subunit J [EC:1.6.5.3] | | |
| K00340 | 15 | NADH dehydrogenase I subunit K [EC:1.6.5.3] | | |
| K00341 | 57 | NADH dehydrogenase I subunit L [EC:1.6.5.3] | | |
| K00342 | 63 | NADH dehydrogenase I subunit M [EC:1.6.5.3] | | |
| K00343 | 62 | NADH dehydrogenase I subunit N [EC:1.6.5.3] | | |
| K00344 | 170 | NADPH2:quinone reductase [EC:1.6.5.5] | | |
| K00346 | 2 | Na+-transporting NADH:ubiquinone oxidoreductase subunit A | | |
| K00347 | 10 | Na+-transporting NADH:ubiquinone oxidoreductase subunit B | | |
| K00348 | 7 | Na+-transporting NADH:ubiquinone oxidoreductase subunit C | | |
| K00349 | 2 | Na+-transporting NADH:ubiquinone oxidoreductase subunit D | | |

| | | | | |
|---|---|---|---|---|
| K00350 | 9 | Na+-transporting NADH:ubiquinone oxidoreductase subunit E | | |
| K00351 | 4 | Na+-transporting NADH:ubiquinone oxidoreductase subunit F | | |
| K00353 | 1 | Unclassified; E1.6.6.- | | |
| K00354 | 18 | NADPH2 dehydrogenase [EC:1.6.99.1] | | |
| K00355 | 4 | NAD(P)H dehydrogenase (quinone) [EC:1.6.5.2] | | |
| K00356 | 59 | NADH dehydrogenase [EC:1.6.99.3] | | |
| K00358 | 10 | Unclassified; E1.6.99.- | | |
| K00359 | 38 | NADH oxidase [EC:1.6.-.-] | | |
| K00360 | 39 | nitrate reductase (NADH) [EC:1.7.1.1] | | |
| K00362 | 26 | nitrite reductase (NAD(P)H) large subunit [EC:1.7.1.4] | | |
| K00363 | 13 | nitrite reductase (NAD(P)H) small subunit [EC:1.7.1.4] | | |
| K00364 | 4 | GMP reductase [EC:1.7.1.7] | | |
| K00365 | 9 | urate oxidase [EC:1.7.3.3] | | |
| K00366 | 23 | ferredoxin-nitrite reductase [EC:1.7.7.1] | | |
| K00367 | 5 | ferredoxin-nitrate reductase [EC:1.7.7.2] | | |
| K00368 | 53 | nitrite reductase (NO-forming) [EC:1.7.2.1] | | |
| K00369 | 45 | nitrate reductase [EC:1.7.99.4] | | |
| K00370 | 34 | nitrate reductase 1, alpha subunit [EC:1.7.99.4] | | |
| K00371 | 12 | nitrate reductase 1, beta subunit [EC:1.7.99.4] | | |
| K00372 | 36 | nitrate reductase catalytic subunit [EC:1.7.99.4] | | |
| K00373 | 4 | nitrate reductase 1, delta subunit [EC:1.7.99.4] | | |
| K00374 | 4 | nitrate reductase 1, gamma subunit [EC:1.7.99.4] | | |
| K00375 | 33 | GntR family transcriptional regulator / MocR family aminotransferase | | |
| K00376 | 14 | nitrous-oxide reductase [EC:1.7.99.6] | | |
| K00378 | 3 | hydroxylamine reductase [EC:1.7.-.-] | | |
| K00380 | 19 | sulfite reductase (NADPH) flavoprotein alpha-component [EC:1.8.1.2] | | |
| K00381 | 24 | sulfite reductase (NADPH) hemoprotein beta-component [EC:1.8.1.2] | | |
| K00382 | 97 | dihydrolipoamide dehydrogenase [EC:1.8.1.4] | | |
| K00383 | 19 | glutathione reductase (NADPH) [EC:1.8.1.7] | | |
| K00384 | 111 | thioredoxin reductase (NADPH) [EC:1.8.1.9] | | |
| K00385 | 1 | anaerobic sulfite reductase subunit C [EC:1.8.1.-] | | |
| K00386 | 20 | Unclassified; E1.8.2.- | | |
| K00387 | 22 | sulfite oxidase [EC:1.8.3.1] | | |
| K00389 | 9 | putative membrane protein | | |
| K00390 | 17 | phosphoadenosine phosphosulfate reductase [EC:1.8.4.8] | | |
| K00391 | 5 | Unclassified; E1.8.4.- | | |
| K00392 | 14 | sulfite reductase (ferredoxin) [EC:1.8.7.1] | | |
| K00394 | 11 | adenylylsulfate reductase, subunit A [EC:1.8.99.2] | | |
| K00395 | 3 | adenylylsulfate reductase, subunit B [EC:1.8.99.2] | | |
| K00397 | 12 | anaerobic dimethyl sulfoxide reductase [EC:1.8.99.-] | | |
| K00404 | 16 | cb-type cytochrome c oxidase subunit I [EC:1.9.3.1] | | |
| K00405 | 17 | cb-type cytochrome c oxidase subunit II [EC:1.9.3.1] | | |
| K00406 | 34 | cb-type cytochrome c oxidase subunit III [EC:1.9.3.1] | | |
| K00407 | 6 | cb-type cytochrome c oxidase subunit IV [EC:1.9.3.1] | | |
| K00411 | 10 | ubiquinol-cytochrome c reductase iron-sulfur subunit [EC:1.10.2.2] | | |
| K00412 | 35 | ubiquinol-cytochrome c reductase cytochrome b subunit [EC:1.10.2.2] | | |
| K00413 | 8 | ubiquinol-cytochrome c reductase cytochrome c1 subunit [EC:1.10.2.2] | | |
| K00421 | 4 | Unclassified; E1.10.2.- | | |
| K00423 | 22 | L-ascorbate oxidase [EC:1.10.3.3] | | |
| K00425 | 39 | cytochrome bd-I oxidase subunit I [EC:1.10.3.-] | | |

| | | | | |
|---|---|---|---|---|
| K00426 | 23 | cytochrome bd-I oxidase subunit II [EC:1.10.3.-] | | |
| K00427 | 2 | L-lactate permease | | |
| K00428 | 51 | cytochrome c peroxidase [EC:1.11.1.5] | | |
| K00429 | 1 | catalase [EC:1.11.1.6] | | |
| K00432 | 7 | glutathione peroxidase [EC:1.11.1.9] | | |
| K00433 | 36 | chloride peroxidase [EC:1.11.1.10] | | |
| K00435 | 19 | peroxiredoxin [EC:1.11.1.-] | | |
| K00436 | 91 | hydrogen dehydrogenase [EC:1.12.1.2] | | |
| K00437 | 8 | cytochrome-c3 hydrogenase [EC:1.12.2.1] | | |
| K00441 | 4 | coenzyme F420 hydrogenase beta subunit [EC:1.12.98.1] | | |
| K00442 | 3 | coenzyme F420 hydrogenase delta subunit | | |
| K00446 | 7 | catechol 2,3-dioxygenase [EC:1.13.11.2] | | |
| K00448 | 10 | protocatechuate 3,4-dioxygenase, alpha subunit [EC:1.13.11.3] | | |
| K00449 | 6 | protocatechuate 3,4-dioxygenase, beta subunit [EC:1.13.11.3] | | |
| K00450 | 24 | gentisate 1,2-dioxygenase [EC:1.13.11.4] | | |
| K00451 | 27 | homogentisate 1,2-dioxygenase [EC:1.13.11.5] | | |
| K00453 | 11 | tryptophan 2,3-dioxygenase [EC:1.13.11.11] | | |
| K00455 | 1 | 3,4-dihydroxyphenylacetate 2,3-dioxygenase [EC:1.13.11.15] | | |
| K00457 | 19 | 4-hydroxyphenylpyruvate dioxygenase [EC:1.13.11.27] | | |
| K00459 | 54 | nitronate monooxygenase [EC:1.13.12.16] | | |
| K00462 | 4 | biphenyl-2,3-diol 1,2-dioxygenase [EC:1.13.11.39] | | |
| K00464 | 2 | lignostilbene-alpha,beta-dioxygenase [EC:1.13.11.43] | | |
| K00465 | 24 | Unclassified; E1.13.11.- | | |
| K00466 | 5 | tryptophan 2-monooxygenase [EC:1.13.12.3] | | |
| K00467 | 12 | lactate 2-monooxygenase [EC:1.13.12.4] | | |
| K00470 | 8 | Unclassified; E1.14.1.- | | |
| K00476 | 1 | aspartate beta-hydroxylase [EC:1.14.11.16] | | |
| K00477 | 2 | phytanoyl-CoA hydroxylase [EC:1.14.11.18] | | |
| K00478 | 6 | Unclassified; E1.14.11.- | | |
| K00479 | 6 | Rieske 2Fe-2S family protein | | |
| K00480 | 28 | salicylate hydroxylase [EC:1.14.13.1] | | |
| K00481 | 6 | p-hydroxybenzoate 3-monooxygenase [EC:1.14.13.2] | | |
| K00483 | 31 | 4-hydroxyphenylacetate-3-hydroxylase large chain [EC:1.14.13.3] | | |
| K00484 | 2 | 4-hydroxyphenylacetate-3-hydroxylase small chain [EC:1.14.13.3] | | |
| K00485 | 11 | dimethylaniline monooxygenase (N-oxide forming) [EC:1.14.13.8] | | |
| K00491 | 2 | nitric-oxide synthase, bacterial [EC:1.14.13.39] | | |
| K00492 | 81 | Unclassified; E1.14.13.- | | |
| K00493 | 46 | unspecific monooxygenase [EC:1.14.14.1] | | |
| K00494 | 70 | alkanal monooxygenase (FMN-linked) [EC:1.14.14.3] | | |
| K00495 | 10 | Unclassified; E1.14.14.- | | |
| K00499 | 10 | choline monooxygenase [EC:1.14.15.7] | | |
| K00500 | 17 | phenylalanine-4-hydroxylase [EC:1.14.16.1] | | |
| K00504 | 76 | peptidylglycine monooxygenase [EC:1.14.17.3] | | |
| K00507 | 40 | stearoyl-CoA desaturase (delta-9 desaturase) [EC:1.14.19.1] | | |
| K00508 | 4 | linoleoyl-CoA desaturase [EC:1.14.19.3] | | |
| K00514 | 1 | zeta-carotene desaturase [EC:1.14.99.30] | | |
| K00517 | 179 | Unclassified; E1.14.-.- | | |
| K00520 | 60 | mercuric reductase [EC:1.16.1.1] | | |
| K00523 | 25 | CDP-4-dehydro-6-deoxyglucose reductase [EC:1.17.1.1] | | |
| K00525 | 87 | ribonucleoside-diphosphate reductase alpha chain [EC:1.17.4.1] | | |

| | | | | |
|---|---|---|---|---|
| K00526 | 14 | ribonucleoside-diphosphate reductase beta chain [EC:1.17.4.1] | | |
| K00527 | 5 | ribonucleoside-triphosphate reductase [EC:1.17.4.2] | | |
| K00528 | 42 | ferredoxin--NADP+ reductase [EC:1.18.1.2] | | |
| K00529 | 61 | ferredoxin--NAD+ reductase [EC:1.18.1.3] | | |
| K00530 | 11 | Unclassified; E1.18.1.- | | |
| K00532 | 22 | ferredoxin hydrogenase [EC:1.12.7.2] | | |
| K00533 | 4 | ferredoxin hydrogenase large subunit [EC:1.12.7.2] | | |
| K00534 | 11 | ferredoxin hydrogenase small subunit [EC:1.12.7.2] | | |
| K00535 | 2 | Unclassified; E1.18.-.- | | |
| K00537 | 7 | arsenate reductase [EC:1.20.4.1] | | |
| K00538 | 5 | formate acetyltransferase activating enzyme [EC:1.97.1.4] | | |
| K00539 | 1 | Unclassified; E1.97.1.- | | |
| K00540 | 1132 | Unclassified; E1.-.-.- | | |
| K00544 | 5 | betaine-homocysteine S-methyltransferase [EC:2.1.1.5] | | |
| K00547 | 33 | homocysteine S-methyltransferase [EC:2.1.1.10] | | |
| K00548 | 117 | 5-methyltetrahydrofolate--homocysteine methyltransferase | | |
| K00549 | 72 | 5-methyltetrahydropteroyltriglutamate--homocysteine | | |
| K00551 | 21 | phosphatidylethanolamine N-methyltransferase [EC:2.1.1.17] | | |
| K00554 | 30 | tRNA (guanine-N1-)-methyltransferase [EC:2.1.1.31] | | |
| K00556 | 3 | tRNA (guanosine-2'-O-)-methyltransferase [EC:2.1.1.34] | | |
| K00557 | 12 | tRNA (uracil-5-)-methyltransferase [EC:2.1.1.35] | | |
| K00558 | 15 | DNA (cytosine-5-)-methyltransferase [EC:2.1.1.37] | | |
| K00559 | 1 | sterol 24-C-methyltransferase [EC:2.1.1.41] | | |
| K00560 | 9 | thymidylate synthase [EC:2.1.1.45] | | |
| K00561 | 14 | rRNA (adenine-N6-)-methyltransferase [EC:2.1.1.48] | | |
| K00563 | 4 | rRNA (guanine-N1-)-methyltransferase [EC:2.1.1.51] | | |
| K00564 | 1 | ribosomal RNA small subunit methyltransferase C [EC:2.1.1.52] | | |
| K00566 | 48 | tRNA (5-methylaminomethyl-2-thiouridylate)-methyltransferase | | |
| K00567 | 30 | methylated-DNA-[protein]-cysteine S-methyltransferase [EC:2.1.1.63] | | |
| K00568 | 65 | 3-demethylubiquinone-9 3-methyltransferase [EC:2.1.1.- 2.1.1.64] | | |
| K00569 | 4 | thiopurine S-methyltransferase [EC:2.1.1.67] | | |
| K00570 | 5 | phosphatidyl-N-methylethanolamine N-methyltransferase [EC:2.1.1.71] | | |
| K00571 | 26 | site-specific DNA-methyltransferase (adenine-specific) [EC:2.1.1.72] | | |
| K00573 | 52 | protein-L-isoaspartate(D-aspartate) O-methyltransferase | | |
| K00574 | 63 | cyclopropane-fatty-acyl-phospholipid synthase [EC:2.1.1.79] | | |
| K00575 | 79 | chemotaxis protein methyltransferase CheR [EC:2.1.1.80] | | |
| K00584 | 1 | tetrahydromethanopterin S-methyltransferase subunit H [EC:2.1.1.86] | | |
| K00587 | 7 | protein-S-isoprenylcysteine O-methyltransferase [EC:2.1.1.100] | | |
| K00588 | 15 | caffeoyl-CoA O-methyltransferase [EC:2.1.1.104] | | |
| K00590 | 11 | site-specific DNA-methyltransferase (cytosine-N4-specific) | | |
| K00594 | 3 | xylitol oxidase [EC:1.1.3.41] | | |
| K00595 | 6 | precorrin-6Y C5,15-methyltransferase / precorrin-8W decarboxylase | | |
| K00596 | 3 | 2,2-dialkylglycine decarboxylase (pyruvate) [EC:4.1.1.64] | | |
| K00598 | 16 | trans-aconitate 2-methyltransferase [EC:2.1.1.144] | | |
| K00599 | 435 | Unclassified; E2.1.1.- | | |
| K00600 | 49 | glycine hydroxymethyltransferase [EC:2.1.2.1] | | |
| K00602 | 28 | phosphoribosylaminoimidazolecarboxamide formyltransferase / IMP | | |
| K00603 | 20 | glutamate formiminotransferase [EC:2.1.2.5] | | |
| K00604 | 50 | methionyl-tRNA formyltransferase [EC:2.1.2.9] | | |
| K00605 | 69 | aminomethyltransferase [EC:2.1.2.10] | | |

| | | | | |
|---|---|---|---|---|
| K00606 | 26 | 3-methyl-2-oxobutanoate hydroxymethyltransferase [EC:2.1.2.11] | | |
| K00607 | 3 | Unclassified; E2.1.2.- | | |
| K00609 | 35 | aspartate carbamoyltransferase catalytic subunit [EC:2.1.3.2] | | |
| K00610 | 12 | aspartate carbamoyltransferase regulatory subunit | | |
| K00611 | 50 | ornithine carbamoyltransferase [EC:2.1.3.3] | | |
| K00612 | 37 | carbamoyltransferase [EC:2.1.3.-] | | |
| K00613 | 1 | glycine amidinotransferase [EC:2.1.4.1] | | |
| K00614 | 13 | Unclassified; E2.1.-.- | | |
| K00615 | 102 | transketolase [EC:2.2.1.1] | | |
| K00616 | 40 | transaldolase [EC:2.2.1.2] | | |
| K00617 | 1 | Unclassified; E2.2.1.- | | |
| K00619 | 22 | amino-acid N-acetyltransferase [EC:2.3.1.1] | | |
| K00620 | 31 | glutamate N-acetyltransferase / amino-acid N-acetyltransferase | | |
| K00622 | 5 | arylamine N-acetyltransferase [EC:2.3.1.5] | | |
| K00625 | 21 | phosphate acetyltransferase [EC:2.3.1.8] | | |
| K00626 | 211 | acetyl-CoA C-acetyltransferase [EC:2.3.1.9] | | |
| K00627 | 68 | pyruvate dehydrogenase E2 component (dihydrolipoamide | | |
| K00631 | 12 | glycerol-3-phosphate O-acyltransferase [EC:2.3.1.15] | | |
| K00632 | 93 | acetyl-CoA acyltransferase [EC:2.3.1.16] | | |
| K00633 | 18 | galactoside O-acetyltransferase [EC:2.3.1.18] | | |
| K00634 | 11 | phosphate butyryltransferase [EC:2.3.1.19] | | |
| K00638 | 6 | chloramphenicol O-acetyltransferase [EC:2.3.1.28] | | |
| K00639 | 42 | glycine C-acetyltransferase [EC:2.3.1.29] | | |
| K00640 | 27 | serine O-acetyltransferase [EC:2.3.1.30] | | |
| K00641 | 37 | homoserine O-acetyltransferase [EC:2.3.1.31] | | |
| K00643 | 12 | 5-aminolevulinate synthase [EC:2.3.1.37] | | |
| K00645 | 48 | [acyl-carrier-protein] S-malonyltransferase [EC:2.3.1.39] | | |
| K00647 | 39 | 3-oxoacyl-[acyl-carrier-protein] synthase I [EC:2.3.1.41] | | |
| K00648 | 79 | 3-oxoacyl-[acyl-carrier-protein] synthase III [EC:2.3.1.180] | | |
| K00651 | 3 | homoserine O-succinyltransferase [EC:2.3.1.46] | | |
| K00652 | 51 | 8-amino-7-oxononanoate synthase [EC:2.3.1.47] | | |
| K00654 | 7 | serine palmitoyltransferase [EC:2.3.1.50] | | |
| K00655 | 121 | 1-acyl-sn-glycerol-3-phosphate acyltransferase [EC:2.3.1.51] | | |
| K00656 | 38 | formate C-acetyltransferase [EC:2.3.1.54] | | |
| K00657 | 13 | diamine N-acetyltransferase [EC:2.3.1.57] | | |
| K00658 | 41 | 2-oxoglutarate dehydrogenase E2 component (dihydrolipoamide | | |
| K00660 | 12 | chalcone synthase [EC:2.3.1.74] | | |
| K00661 | 8 | maltose O-acetyltransferase [EC:2.3.1.79] | | |
| K00662 | 3 | aminoglycoside N3'-acetyltransferase [EC:2.3.1.81] | | |
| K00663 | 9 | aminoglycoside N6'-acetyltransferase [EC:2.3.1.82] | | |
| K00666 | 187 | fatty-acyl-CoA synthase [EC:6.2.1.-] | | |
| K00672 | 6 | formylmethanofuran--tetrahydromethanopterin N-formyltransferase | | |
| K00673 | 3 | arginine N-succinyltransferase [EC:2.3.1.109] | | |
| K00674 | 18 | 2,3,4,5-tetrahydropyridine-2-carboxylate N-succinyltransferase | | |
| K00675 | 16 | N-hydroxyarylamine O-acetyltransferase [EC:2.3.1.118] | | |
| K00676 | 20 | ribosomal-protein-alanine N-acetyltransferase [EC:2.3.1.128] | | |
| K00677 | 32 | UDP-N-acetylglucosamine acyltransferase [EC:2.3.1.129] | | |
| K00680 | 275 | Unclassified; E2.3.1.- | | |
| K00681 | 137 | gamma-glutamyltranspeptidase [EC:2.3.2.2] | | |
| K00683 | 3 | glutaminyl-peptide cyclotransferase [EC:2.3.2.5] | | |

| | | | | |
|---|---|---|---|---|
| K00684 | 12 | leucyl/phenylalanyl-tRNA--protein transferase [EC:2.3.2.6] | | |
| K00685 | 7 | arginine-tRNA-protein transferase [EC:2.3.2.8] | | |
| K00688 | 90 | starch phosphorylase [EC:2.4.1.1] | | |
| K00690 | 3 | sucrose phosphorylase [EC:2.4.1.7] | | |
| K00691 | 17 | maltose phosphorylase [EC:2.4.1.8] | | |
| K00693 | 14 | glycogen(starch) synthase [EC:2.4.1.11] | | |
| K00694 | 12 | cellulose synthase (UDP-forming) [EC:2.4.1.12] | | |
| K00695 | 2 | sucrose synthase [EC:2.4.1.13] | | |
| K00696 | 1 | sucrose-phosphate synthase [EC:2.4.1.14] | | |
| K00697 | 44 | alpha,alpha-trehalose-phosphate synthase (UDP-forming) [EC:2.4.1.15] | | |
| K00700 | 62 | 1,4-alpha-glucan branching enzyme [EC:2.4.1.18] | | |
| K00701 | 2 | cyclomaltodextrin glucanotransferase [EC:2.4.1.19] | | |
| K00702 | 10 | cellobiose phosphorylase [EC:2.4.1.20] | | |
| K00703 | 39 | starch synthase [EC:2.4.1.21] | | |
| K00705 | 74 | 4-alpha-glucanotransferase [EC:2.4.1.25] | | |
| K00712 | 3 | poly(glycerol-phosphate) alpha-glucosyltransferase [EC:2.4.1.52] | | |
| K00720 | 5 | ceramide glucosyltransferase [EC:2.4.1.80] | | |
| K00721 | 123 | dolichol-phosphate mannosyltransferase [EC:2.4.1.83] | | |
| K00743 | 2 | N-acetyllactosaminide 3-alpha-galactosyltransferase [EC:2.4.1.87] | | |
| K00748 | 28 | lipid-A-disaccharide synthase [EC:2.4.1.182] | | |
| K00752 | 7 | hyaluronan synthase [EC:2.4.1.212] | | |
| K00754 | 464 | Unclassified; E2.4.1.- | | |
| K00756 | 8 | pyrimidine-nucleoside phosphorylase [EC:2.4.2.2] | | |
| K00757 | 2 | uridine phosphorylase [EC:2.4.2.3] | | |
| K00758 | 21 | thymidine phosphorylase [EC:2.4.2.4] | | |
| K00759 | 26 | adenine phosphoribosyltransferase [EC:2.4.2.7] | | |
| K00760 | 33 | hypoxanthine phosphoribosyltransferase [EC:2.4.2.8] | | |
| K00761 | 13 | uracil phosphoribosyltransferase [EC:2.4.2.9] | | |
| K00762 | 22 | orotate phosphoribosyltransferase [EC:2.4.2.10] | | |
| K00763 | 180 | nicotinate phosphoribosyltransferase [EC:2.4.2.11] | | |
| K00764 | 58 | amidophosphoribosyltransferase [EC:2.4.2.14] | | |
| K00765 | 31 | ATP phosphoribosyltransferase [EC:2.4.2.17] | | |
| K00766 | 31 | anthranilate phosphoribosyltransferase [EC:2.4.2.18] | | |
| K00767 | 54 | nicotinate-nucleotide pyrophosphorylase (carboxylating) | | |
| K00768 | 8 | nicotinate-nucleotide--dimethylbenzimidazole | | |
| K00772 | 30 | 5'-methylthioadenosine phosphorylase [EC:2.4.2.28] | | |
| K00773 | 26 | queuine tRNA-ribosyltransferase [EC:2.4.2.29] | | |
| K00777 | 15 | Unclassified; E2.4.2.- | | |
| K00782 | 10 | hypothetical protein | | |
| K00783 | 10 | hypothetical protein | | |
| K00784 | 75 | ribonuclease Z [EC:3.1.26.11] | | |
| K00785 | 2 | beta-galactosamide-alpha-2,3-sialyltransferase [EC:2.4.99.-] | | |
| K00786 | 201 | Unclassified; E2.4.-.- | | |
| K00788 | 35 | thiamine-phosphate pyrophosphorylase [EC:2.5.1.3] | | |
| K00789 | 31 | S-adenosylmethionine synthetase [EC:2.5.1.6] | | |
| K00790 | 50 | UDP-N-acetylglucosamine 1-carboxyvinyltransferase [EC:2.5.1.7] | | |
| K00791 | 29 | tRNA dimethylallyltransferase [EC:2.5.1.75] | | |
| K00793 | 31 | riboflavin synthase alpha chain [EC:2.5.1.9] | | |
| K00794 | 15 | riboflavin synthase beta chain [EC:2.5.1.-] | | |
| K00795 | 8 | farnesyl diphosphate synthase [EC:2.5.1.1 2.5.1.10] | | |

| | | | | |
|---|---|---|---|---|
| K00796 | 35 | dihydropteroate synthase [EC:2.5.1.15] | | |
| K00797 | 37 | spermidine synthase [EC:2.5.1.16] | | |
| K00798 | 27 | cob(I)alamin adenosyltransferase [EC:2.5.1.17] | | |
| K00799 | 108 | glutathione S-transferase [EC:2.5.1.18] | | |
| K00800 | 62 | 3-phosphoshikimate 1-carboxyvinyltransferase [EC:2.5.1.19] | | |
| K00801 | 13 | farnesyl-diphosphate farnesyltransferase [EC:2.5.1.21] | | |
| K00802 | 3 | spermine synthase [EC:2.5.1.22] | | |
| K00803 | 23 | alkyldihydroxyacetonephosphate synthase [EC:2.5.1.26] | | |
| K00805 | 9 | heptaprenyl diphosphate synthase [EC:2.5.1.30] | | |
| K00806 | 20 | undecaprenyl diphosphate synthase [EC:2.5.1.31] | | |
| K00808 | 4 | homospermidine synthase [EC:2.5.1.44] | | |
| K00809 | 18 | deoxyhypusine synthase [EC:2.5.1.46] | | |
| K00810 | 24 | Unclassified; E2.5.1.- | | |
| K00811 | 2 | aspartate aminotransferase [EC:2.6.1.1] | | |
| K00812 | 46 | aspartate aminotransferase [EC:2.6.1.1] | | |
| K00813 | 7 | aspartate aminotransferase [EC:2.6.1.1] | | |
| K00814 | 3 | alanine transaminase [EC:2.6.1.2] | | |
| K00817 | 59 | histidinol-phosphate aminotransferase [EC:2.6.1.9] | | |
| K00818 | 49 | acetylornithine aminotransferase [EC:2.6.1.11] | | |
| K00819 | 14 | ornithine--oxo-acid transaminase [EC:2.6.1.13] | | |
| K00820 | 67 | glucosamine--fructose-6-phosphate aminotransferase (isomerizing) | | |
| K00821 | 47 | acetylornithine/N-succinyldiaminopimelate aminotransferase | | |
| K00822 | 14 | beta-alanine--pyruvate transaminase [EC:2.6.1.18] | | |
| K00823 | 47 | 4-aminobutyrate aminotransferase [EC:2.6.1.19] | | |
| K00824 | 14 | D-alanine transaminase [EC:2.6.1.21] | | |
| K00825 | 17 | 2-aminoadipate transaminase [EC:2.6.1.39] | | |
| K00826 | 73 | branched-chain amino acid aminotransferase [EC:2.6.1.42] | | |
| K00828 | 29 | serine--glyoxylate transaminase [EC:2.6.1.45] | | |
| K00831 | 23 | phosphoserine aminotransferase [EC:2.6.1.52] | | |
| K00832 | 8 | aromatic-amino-acid transaminase [EC:2.6.1.57] | | |
| K00833 | 19 | adenosylmethionine-8-amino-7-oxononanoate aminotransferase | | |
| K00835 | 12 | valine--pyruvate aminotransferase [EC:2.6.1.66] | | |
| K00836 | 5 | diaminobutyrate-2-oxoglutarate transaminase [EC:2.6.1.76] | | |
| K00837 | 274 | Unclassified; E2.6.1.- | | |
| K00839 | 38 | aminotransferase [EC:2.6.1.-] | | |
| K00840 | 1 | succinylornithine aminotransferase [EC:2.6.1.81] | | |
| K00841 | 1 | aminotransferase [EC:2.6.1.-] | | |
| K00842 | 11 | aminotransferase [EC:2.6.1.-] | | |
| K00843 | 6 | Unclassified; E2.6.-.- | | |
| K00845 | 73 | glucokinase [EC:2.7.1.2] | | |
| K00846 | 2 | ketohexokinase [EC:2.7.1.3] | | |
| K00847 | 33 | fructokinase [EC:2.7.1.4] | | |
| K00848 | 14 | rhamnulokinase [EC:2.7.1.5] | | |
| K00849 | 14 | galactokinase [EC:2.7.1.6] | | |
| K00850 | 56 | 6-phosphofructokinase [EC:2.7.1.11] | | |
| K00851 | 14 | gluconokinase [EC:2.7.1.12] | | |
| K00852 | 25 | ribokinase [EC:2.7.1.15] | | |
| K00853 | 8 | L-ribulokinase [EC:2.7.1.16] | | |
| K00854 | 34 | xylulokinase [EC:2.7.1.17] | | |
| K00855 | 13 | phosphoribulokinase [EC:2.7.1.19] | | |

| | | | | |
|---|---|---|---|---|
| K00856 | 21 | adenosine kinase [EC:2.7.1.20] | | |
| K00857 | 8 | thymidine kinase [EC:2.7.1.21] | | |
| K00858 | 35 | NAD+ kinase [EC:2.7.1.23] | | |
| K00859 | 35 | dephospho-CoA kinase [EC:2.7.1.24] | | |
| K00860 | 18 | adenylylsulfate kinase [EC:2.7.1.25] | | |
| K00861 | 3 | riboflavin kinase [EC:2.7.1.26] | | |
| K00862 | 1 | erythritol kinase [EC:2.7.1.27] | | |
| K00863 | 8 | dihydroxyacetone kinase [EC:2.7.1.29] | | |
| K00864 | 41 | glycerol kinase [EC:2.7.1.30] | | |
| K00865 | 12 | glycerate kinase [EC:2.7.1.31] | | |
| K00866 | 1 | choline kinase [EC:2.7.1.32] | | |
| K00867 | 2 | type I pantothenate kinase [EC:2.7.1.33] | | |
| K00868 | 445 | pyridoxine kinase [EC:2.7.1.35] | | |
| K00869 | 1 | mevalonate kinase [EC:2.7.1.36] | | |
| K00870 | 68 | protein kinase [EC:2.7.1.37] | | |
| K00872 | 9 | homoserine kinase [EC:2.7.1.39] | | |
| K00873 | 44 | pyruvate kinase [EC:2.7.1.40] | | |
| K00874 | 20 | 2-dehydro-3-deoxygluconokinase [EC:2.7.1.45] | | |
| K00875 | 2 | D-ribulokinase [EC:2.7.1.47] | | |
| K00876 | 12 | uridine kinase [EC:2.7.1.48] | | |
| K00877 | 3 | hydroxymethylpyrimidine kinase [EC:2.7.1.49] | | |
| K00878 | 3 | hydroxyethylthiazole kinase [EC:2.7.1.50] | | |
| K00879 | 4 | L-fuculokinase [EC:2.7.1.51] | | |
| K00880 | 1 | L-xylulokinase [EC:2.7.1.53] | | |
| K00882 | 14 | 1-phosphofructokinase [EC:2.7.1.56] | | |
| K00883 | 1 | 2-dehydro-3-deoxygalactonokinase [EC:2.7.1.58] | | |
| K00884 | 7 | N-acetylglucosamine kinase [EC:2.7.1.59] | | |
| K00885 | 1 | N-acylmannosamine kinase [EC:2.7.1.60] | | |
| K00886 | 9 | polyphosphate glucokinase [EC:2.7.1.63] | | |
| K00887 | 10 | undecaprenol kinase [EC:2.7.1.66] | | |
| K00891 | 31 | shikimate kinase [EC:2.7.1.71] | | |
| K00894 | 3 | ethanolamine kinase [EC:2.7.1.82] | | |
| K00895 | 22 | pyrophosphate--fructose-6-phosphate 1-phosphotransferase | | |
| K00897 | 10 | aminoglycoside 3'-phosphotransferase [EC:2.7.1.95] | | |
| K00901 | 6 | diacylglycerol kinase [EC:2.7.1.107] | | |
| K00903 | 70 | protein-tyrosine kinase [EC:2.7.10.-] | | |
| K00904 | 18 | deoxyguanosine kinase [EC:2.7.1.113] | | |
| K00906 | 11 | isocitrate dehydrogenase kinase/phosphatase [EC:2.7.11.5 3.1.3.-] | | |
| K00908 | 216 | Ca2+/calmodulin-dependent protein kinase [EC:2.7.11.17] | | |
| K00912 | 13 | tetraacyldisaccharide 4'-kinase [EC:2.7.1.130] | | |
| K00917 | 9 | tagatose 6-phosphate kinase [EC:2.7.1.144] | | |
| K00919 | 25 | 4-diphosphocytidyl-2-C-methyl-D-erythritol kinase [EC:2.7.1.148] | | |
| K00924 | 351 | Unclassified; E2.7.1.- | | |
| K00925 | 31 | acetate kinase [EC:2.7.2.1] | | |
| K00926 | 18 | carbamate kinase [EC:2.7.2.2] | | |
| K00927 | 35 | phosphoglycerate kinase [EC:2.7.2.3] | | |
| K00928 | 33 | aspartate kinase [EC:2.7.2.4] | | |
| K00929 | 3 | butyrate kinase [EC:2.7.2.7] | | |
| K00930 | 23 | acetylglutamate kinase [EC:2.7.2.8] | | |
| K00931 | 54 | glutamate 5-kinase [EC:2.7.2.11] | | |

| | | | | |
|---|---|---|---|---|
| K00932 | 1 | propionate kinase [EC:2.7.2.15] | | |
| K00933 | 1 | creatine kinase [EC:2.7.3.2] | | |
| K00934 | 4 | arginine kinase [EC:2.7.3.3] | | |
| K00935 | 4 | Unclassified; E2.7.3.9 | | |
| K00936 | 1280 | Unclassified; E2.7.3.- | | |
| K00937 | 60 | polyphosphate kinase [EC:2.7.4.1] | | |
| K00938 | 5 | phosphomevalonate kinase [EC:2.7.4.2] | | |
| K00939 | 34 | adenylate kinase [EC:2.7.4.3] | | |
| K00940 | 20 | nucleoside-diphosphate kinase [EC:2.7.4.6] | | |
| K00941 | 29 | phosphomethylpyrimidine kinase [EC:2.7.4.7] | | |
| K00942 | 26 | guanylate kinase [EC:2.7.4.8] | | |
| K00943 | 33 | dTMP kinase [EC:2.7.4.9] | | |
| K00945 | 33 | cytidylate kinase [EC:2.7.4.14] | | |
| K00946 | 33 | thiamine-monophosphate kinase [EC:2.7.4.16] | | |
| K00947 | 27 | Unclassified; E2.7.4.- | | |
| K00948 | 177 | ribose-phosphate pyrophosphokinase [EC:2.7.6.1] | | |
| K00950 | 17 | 2-amino-4-hydroxy-6-hydroxymethyldihydropteridine diphosphokinase | | |
| K00951 | 90 | GTP pyrophosphokinase [EC:2.7.6.5] | | |
| K00954 | 23 | pantetheine-phosphate adenylyltransferase [EC:2.7.7.3] | | |
| K00955 | 28 | bifunctional enzyme CysN/CysC [EC:2.7.7.4 2.7.1.25] | | |
| K00956 | 29 | sulfate adenylyltransferase subunit 1 [EC:2.7.7.4] | | |
| K00957 | 10 | sulfate adenylyltransferase subunit 2 [EC:2.7.7.4] | | |
| K00958 | 25 | sulfate adenylyltransferase [EC:2.7.7.4] | | |
| K00960 | 7 | DNA-directed RNA polymerase [EC:2.7.7.6] | | |
| K00961 | 7 | DNA polymerase [EC:2.7.7.7] | | |
| K00962 | 39 | polyribonucleotide nucleotidyltransferase [EC:2.7.7.8] | | |
| K00963 | 23 | UTP--glucose-1-phosphate uridylyltransferase [EC:2.7.7.9] | | |
| K00964 | 23 | galactose-1-phosphate uridylyltransferase [EC:2.7.7.10] | | |
| K00965 | 24 | UDPglucose--hexose-1-phosphate uridylyltransferase [EC:2.7.7.12] | | |
| K00966 | 49 | mannose-1-phosphate guanylyltransferase [EC:2.7.7.13] | | |
| K00969 | 56 | nicotinate-nucleotide adenylyltransferase [EC:2.7.7.18] | | |
| K00970 | 57 | poly(A) polymerase [EC:2.7.7.19] | | |
| K00971 | 42 | mannose-1-phosphate guanylyltransferase [EC:2.7.7.22] | | |
| K00972 | 3 | UDP-N-acetylglucosamine pyrophosphorylase [EC:2.7.7.23] | | |
| K00973 | 45 | glucose-1-phosphate thymidylyltransferase [EC:2.7.7.24] | | |
| K00974 | 27 | tRNA nucleotidyltransferase (CCA-adding enzyme) [EC:2.7.7.25 | | |
| K00975 | 42 | glucose-1-phosphate adenylyltransferase [EC:2.7.7.27] | | |
| K00978 | 15 | glucose-1-phosphate cytidylyltransferase [EC:2.7.7.33] | | |
| K00979 | 26 | 3-deoxy-manno-octulosonate cytidylyltransferase (CMP-KDO synthetase) | | |
| K00980 | 8 | glycerol-3-phosphate cytidylyltransferase [EC:2.7.7.39] | | |
| K00981 | 27 | phosphatidate cytidylyltransferase [EC:2.7.7.41] | | |
| K00982 | 55 | glutamate-ammonia-ligase adenylyltransferase [EC:2.7.7.42] | | |
| K00983 | 2 | N-acylneuraminate cytidylyltransferase [EC:2.7.7.43] | | |
| K00985 | 6 | RNA-directed RNA polymerase [EC:2.7.7.48] | | |
| K00986 | 61 | RNA-directed DNA polymerase [EC:2.7.7.49] | | |
| K00989 | 19 | ribonuclease PH [EC:2.7.7.56] | | |
| K00990 | 45 | [protein-PII] uridylyltransferase [EC:2.7.7.59] | | |
| K00991 | 20 | 2-C-methyl-D-erythritol 4-phosphate cytidylyltransferase | | |
| K00992 | 28 | Unclassified; E2.7.7.- | | |
| K00995 | 115 | CDP-diacylglycerol--glycerol-3-phosphate 3-phosphatidyltransferase | | |

| | | | | |
|---|---|---|---|---|
| K00996 | 3 | undecaprenyl-phosphate galactose phosphotransferase [EC:2.7.8.6] | | |
| K00997 | 15 | holo-[acyl-carrier protein] synthase [EC:2.7.8.7] | | |
| K00998 | 20 | phosphatidylserine synthase [EC:2.7.8.8] | | |
| K00999 | 4 | CDP-diacylglycerol--inositol 3-phosphatidyltransferase [EC:2.7.8.11] | | |
| K01000 | 52 | phospho-N-acetylmuramoyl-pentapeptide-transferase [EC:2.7.8.13] | | |
| K01002 | 9 | phosphoglycerol transferase [EC:2.7.8.20] | | |
| K01003 | 30 | carboxyvinyl-carboxyphosphonate phosphorylmutase [EC:2.7.8.23] | | |
| K01005 | 35 | Unclassified; E2.7.8.- | | |
| K01006 | 55 | pyruvate,orthophosphate dikinase [EC:2.7.9.1] | | |
| K01007 | 80 | pyruvate, water dikinase [EC:2.7.9.2] | | |
| K01008 | 29 | selenide, water dikinase [EC:2.7.9.3] | | |
| K01010 | 60 | thiosulfate sulfurtransferase [EC:2.8.1.1] | | |
| K01011 | 21 | 3-mercaptopyruvate sulfurtransferase [EC:2.8.1.2] | | |
| K01012 | 22 | biotin synthetase [EC:2.8.1.6] | | |
| K01013 | 25 | Unclassified; E2.8.1.- | | |
| K01026 | 7 | propionate CoA-transferase [EC:2.8.3.1] | | |
| K01027 | 3 | 3-oxoacid CoA-transferase [EC:2.8.3.5] | | |
| K01028 | 6 | 3-oxoacid CoA-transferase subunit A [EC:2.8.3.5] | | |
| K01029 | 3 | 3-oxoacid CoA-transferase subunit B [EC:2.8.3.5] | | |
| K01031 | 3 | 3-oxoadipate CoA-transferase, alpha subunit [EC:2.8.3.6] | | |
| K01032 | 3 | 3-oxoadipate CoA-transferase, beta subunit [EC:2.8.3.6] | | |
| K01035 | 2 | acetate CoA-transferase beta subunit [EC:2.8.3.8] | | |
| K01036 | 1 | butyrate-acetoacetate CoA-transferase [EC:2.8.3.9] | | |
| K01039 | 25 | glutaconate CoA-transferase, subunit A [EC:2.8.3.12] | | |
| K01040 | 18 | glutaconate CoA-transferase, subunit B [EC:2.8.3.12] | | |
| K01041 | 111 | Unclassified; E2.8.3.- | | |
| K01042 | 37 | L-seryl-tRNA(Ser) seleniumtransferase [EC:2.9.1.1] | | |
| K01043 | 161 | Unclassified; E2.-.-.- | | |
| K01044 | 56 | carboxylesterase [EC:3.1.1.1] | | |
| K01046 | 68 | triacylglycerol lipase [EC:3.1.1.3] | | |
| K01048 | 6 | lysophospholipase [EC:3.1.1.5] | | |
| K01051 | 2 | pectinesterase [EC:3.1.1.11] | | |
| K01053 | 63 | gluconolactonase [EC:3.1.1.17] | | |
| K01054 | 3 | acylglycerol lipase [EC:3.1.1.23] | | |
| K01055 | 56 | 3-oxoadipate enol-lactonase [EC:3.1.1.24] | | |
| K01056 | 29 | peptidyl-tRNA hydrolase, PTH1 family [EC:3.1.1.29] | | |
| K01057 | 24 | 6-phosphogluconolactonase [EC:3.1.1.31] | | |
| K01058 | 8 | phospholipase A1 [EC:3.1.1.32] | | |
| K01060 | 3 | cephalosporin-C deacetylase [EC:3.1.1.41] | | |
| K01061 | 68 | carboxymethylenebutenolidase [EC:3.1.1.45] | | |
| K01062 | 1 | 1-alkyl-2-acetylglycerophosphocholine esterase [EC:3.1.1.47] | | |
| K01066 | 94 | esterase / lipase [EC:3.1.1.-] | | |
| K01067 | 12 | acetyl-CoA hydrolase [EC:3.1.2.1] | | |
| K01068 | 5 | palmitoyl-CoA hydrolase [EC:3.1.2.2] | | |
| K01069 | 110 | hydroxyacylglutathione hydrolase [EC:3.1.2.6] | | |
| K01070 | 5 | S-formylglutathione hydrolase [EC:3.1.2.12] | | |
| K01071 | 2 | oleoyl-[acyl-carrier-protein] hydrolase [EC:3.1.2.14] | | |
| K01073 | 7 | acyl-CoA hydrolase [EC:3.1.2.20] | | |
| K01075 | 12 | 4-hydroxybenzoyl-CoA thioesterase [EC:3.1.2.23] | | |
| K01076 | 26 | Unclassified; E3.1.2.- | | |

| | | | | |
|---|---|---|---|---|
| K01077 | 90 | alkaline phosphatase [EC:3.1.3.1] | | |
| K01078 | 17 | acid phosphatase [EC:3.1.3.2] | | |
| K01079 | 34 | phosphoserine phosphatase [EC:3.1.3.3] | | |
| K01081 | 32 | 5'-nucleotidase [EC:3.1.3.5] | | |
| K01083 | 1 | 3-phytase [EC:3.1.3.8] | | |
| K01087 | 73 | trehalose-phosphatase [EC:3.1.3.12] | | |
| K01090 | 68 | protein phosphatase [EC:3.1.3.16] | | |
| K01091 | 72 | phosphoglycolate phosphatase [EC:3.1.3.18] | | |
| K01092 | 58 | myo-inositol-1(or 4)-monophosphatase [EC:3.1.3.25] | | |
| K01093 | 4 | 4-phytase / acid phosphatase [EC:3.1.3.26 3.1.3.2] | | |
| K01095 | 8 | phosphatidylglycerophosphatase A [EC:3.1.3.27] | | |
| K01096 | 4 | phosphatidylglycerophosphatase B [EC:3.1.3.27] | | |
| K01101 | 7 | 4-nitrophenyl phosphatase [EC:3.1.3.41] | | |
| K01103 | 1 | fructose-2,6-bisphosphatase [EC:3.1.3.46] | | |
| K01104 | 39 | protein-tyrosine phosphatase [EC:3.1.3.48] | | |
| K01112 | 32 | Unclassified; E3.1.3.- | | |
| K01113 | 42 | phosphodiesterase/alkaline phosphatase D [EC:3.1.4.1] | | |
| K01114 | 33 | phospholipase C [EC:3.1.4.3] | | |
| K01115 | 8 | phospholipase D [EC:3.1.4.4] | | |
| K01118 | 3 | FMN-dependent NADH-azoreductase [EC:1.7.-.-] | | |
| K01119 | 13 | 2',3'-cyclic-nucleotide 2'-phosphodiesterase [EC:3.1.4.16] | | |
| K01120 | 22 | 3',5'-cyclic-nucleotide phosphodiesterase [EC:3.1.4.17] | | |
| K01121 | 1 | 2',3'-cyclic-nucleotide 3'-phosphodiesterase [EC:3.1.4.37] | | |
| K01126 | 47 | glycerophosphoryl diester phosphodiesterase [EC:3.1.4.46] | | |
| K01128 | 8 | Unclassified; E3.1.4.- | | |
| K01129 | 24 | dGTPase [EC:3.1.5.1] | | |
| K01130 | 86 | arylsulfatase [EC:3.1.6.1] | | |
| K01131 | 7 | steryl-sulfatase [EC:3.1.6.2] | | |
| K01132 | 17 | N-acetylgalactosamine-6-sulfatase [EC:3.1.6.4] | | |
| K01133 | 31 | choline-sulfatase [EC:3.1.6.6] | | |
| K01134 | 23 | arylsulfatase A [EC:3.1.6.8] | | |
| K01135 | 17 | arylsulfatase B [EC:3.1.6.12] | | |
| K01136 | 8 | iduronate 2-sulfatase [EC:3.1.6.13] | | |
| K01137 | 10 | N-acetylglucosamine-6-sulfatase [EC:3.1.6.14] | | |
| K01138 | 72 | Unclassified; E3.1.6.- | | |
| K01139 | 45 | guanosine-3',5'-bis(diphosphate) 3'-pyrophosphohydrolase | | |
| K01142 | 29 | exodeoxyribonuclease III [EC:3.1.11.2] | | |
| K01144 | 43 | exodeoxyribonuclease V [EC:3.1.11.5] | | |
| K01146 | 7 | Unclassified; E3.1.11.- | | |
| K01147 | 24 | exoribonuclease II [EC:3.1.13.1] | | |
| K01151 | 37 | deoxyribonuclease IV [EC:3.1.21.2] | | |
| K01152 | 2 | type I restriction enzyme [EC:3.1.21.3] | | |
| K01153 | 63 | type I restriction enzyme, R subunit [EC:3.1.21.3] | | |
| K01154 | 15 | type I restriction enzyme, S subunit [EC:3.1.21.3] | | |
| K01155 | 2 | type II restriction enzyme [EC:3.1.21.4] | | |
| K01156 | 7 | type III restriction enzyme [EC:3.1.21.5] | | |
| K01157 | 37 | Unclassified; E3.1.21.- | | |
| K01159 | 34 | crossover junction endodeoxyribonuclease RuvC [EC:3.1.22.4] | | |
| K01162 | 2 | ribonuclease III [EC:3.1.26.3] | | |
| K01166 | 1 | ribonuclease T2 [EC:3.1.27.1] | | |

| | | | | |
|---|---|---|---|---|
| K01167 | 9 | ribonuclease T1 [EC:3.1.27.3] | | |
| K01172 | 1 | Unclassified; E3.1.27.- | | |
| K01173 | 1 | endonuclease [EC:3.1.30.-] | | |
| K01174 | 9 | micrococcal nuclease [EC:3.1.31.1] | | |
| K01175 | 109 | Unclassified; E3.1.-.- | | |
| K01176 | 36 | alpha-amylase [EC:3.2.1.1] | | |
| K01178 | 57 | glucoamylase [EC:3.2.1.3] | | |
| K01179 | 39 | endoglucanase [EC:3.2.1.4] | | |
| K01181 | 11 | endo-1,4-beta-xylanase [EC:3.2.1.8] | | |
| K01182 | 13 | oligo-1,6-glucosidase [EC:3.2.1.10] | | |
| K01183 | 28 | chitinase [EC:3.2.1.14] | | |
| K01184 | 1 | polygalacturonase [EC:3.2.1.15] | | |
| K01185 | 14 | lysozyme [EC:3.2.1.17] | | |
| K01186 | 2 | sialidase-1 [EC:3.2.1.18] | | |
| K01187 | 42 | alpha-glucosidase [EC:3.2.1.20] | | |
| K01188 | 77 | beta-glucosidase [EC:3.2.1.21] | | |
| K01190 | 51 | beta-galactosidase [EC:3.2.1.23] | | |
| K01191 | 17 | alpha-mannosidase [EC:3.2.1.24] | | |
| K01192 | 9 | beta-mannosidase [EC:3.2.1.25] | | |
| K01193 | 2 | beta-fructofuranosidase [EC:3.2.1.26] | | |
| K01194 | 6 | alpha,alpha-trehalase [EC:3.2.1.28] | | |
| K01195 | 8 | beta-glucuronidase [EC:3.2.1.31] | | |
| K01197 | 1 | hyaluronoglucosaminidase [EC:3.2.1.35] | | |
| K01198 | 9 | xylan 1,4-beta-xylosidase [EC:3.2.1.37] | | |
| K01199 | 9 | glucan endo-1,3-beta-D-glucosidase [EC:3.2.1.39] | | |
| K01200 | 4 | pullulanase [EC:3.2.1.41] | | |
| K01201 | 13 | glucosylceramidase [EC:3.2.1.45] | | |
| K01206 | 21 | alpha-L-fucosidase [EC:3.2.1.51] | | |
| K01207 | 53 | beta-N-acetylhexosaminidase [EC:3.2.1.52] | | |
| K01208 | 1 | cyclomaltodextrinase [EC:3.2.1.54] | | |
| K01209 | 22 | alpha-N-arabinofuranosidase [EC:3.2.1.55] | | |
| K01210 | 10 | glucan 1,3-beta-glucosidase [EC:3.2.1.58] | | |
| K01212 | 11 | levanase [EC:3.2.1.65] | | |
| K01213 | 6 | galacturan 1,4-alpha-galacturonidase [EC:3.2.1.67] | | |
| K01215 | 2 | glucan 1,6-alpha-glucosidase [EC:3.2.1.70] | | |
| K01216 | 10 | licheninase [EC:3.2.1.73] | | |
| K01218 | 6 | mannan endo-1,4-beta-mannosidase [EC:3.2.1.78] | | |
| K01220 | 15 | 6-phospho-beta-galactosidase [EC:3.2.1.85] | | |
| K01222 | 6 | 6-phospho-beta-glucosidase [EC:3.2.1.86] | | |
| K01223 | 1 | 6-phospho-beta-glucosidase [EC:3.2.1.86] | | |
| K01224 | 2 | arabinogalactan endo-1,4-beta-galactosidase [EC:3.2.1.89] | | |
| K01225 | 25 | cellulose 1,4-beta-cellobiosidase [EC:3.2.1.91] | | |
| K01226 | 3 | trehalose-6-phosphate hydrolase [EC:3.2.1.93] | | |
| K01234 | 2 | neopullulanase [EC:3.2.1.135] | | |
| K01235 | 3 | alpha-glucuronidase [EC:3.2.1.139] | | |
| K01236 | 28 | maltooligosyltrehalose trehalohydrolase [EC:3.2.1.141] | | |
| K01238 | 177 | Unclassified; E3.2.1.- | | |
| K01239 | 17 | purine nucleosidase [EC:3.2.2.1] | | |
| K01241 | 4 | AMP nucleosidase [EC:3.2.2.4] | | |
| K01243 | 6 | S-adenosylhomocysteine/5'-methylthioadenosine nucleosidase | | |

| | | | | |
|---|---|---|---|---|
| K01246 | 12 | DNA-3-methyladenine glycosylase I [EC:3.2.2.20] | | |
| K01247 | 18 | DNA-3-methyladenine glycosylase II [EC:3.2.2.21] | | |
| K01249 | 33 | DNA glycosylase [EC:3.2.2.-] | | |
| K01250 | 8 | pyrimidine-specific ribonucleoside hydrolase [EC:3.2.-.-] | | |
| K01251 | 29 | adenosylhomocysteinase [EC:3.3.1.1] | | |
| K01253 | 35 | microsomal epoxide hydrolase [EC:3.3.2.9] | | |
| K01255 | 52 | leucyl aminopeptidase [EC:3.4.11.1] | | |
| K01256 | 102 | aminopeptidase N [EC:3.4.11.2] | | |
| K01258 | 13 | tripeptide aminopeptidase [EC:3.4.11.4] | | |
| K01259 | 49 | proline iminopeptidase [EC:3.4.11.5] | | |
| K01262 | 80 | X-Pro aminopeptidase [EC:3.4.11.9] | | |
| K01263 | 9 | cytosol alanyl aminopeptidase [EC:3.4.11.14] | | |
| K01264 | 9 | aminopeptidase Y [EC:3.4.11.15] | | |
| K01265 | 30 | methionyl aminopeptidase [EC:3.4.11.18] | | |
| K01266 | 19 | D-aminopeptidase [EC:3.4.11.19] | | |
| K01267 | 2 | aspartyl aminopeptidase [EC:3.4.11.21] | | |
| K01268 | 1 | aminopeptidase I [EC:3.4.11.22] | | |
| K01269 | 174 | aminopeptidase [EC:3.4.11.-] | | |
| K01270 | 3 | aminoacylhistidine dipeptidase [EC:3.4.13.3] | | |
| K01271 | 52 | X-Pro dipeptidase [EC:3.4.13.9] | | |
| K01273 | 44 | membrane dipeptidase [EC:3.4.13.19] | | |
| K01274 | 10 | D-alanyl-D-alanine dipeptidase [EC:3.4.13.-] | | |
| K01277 | 4 | dipeptidyl-peptidase III [EC:3.4.14.4] | | |
| K01278 | 26 | dipeptidyl-peptidase 4 [EC:3.4.14.5] | | |
| K01281 | 8 | X-Pro dipeptidyl-peptidase [EC:3.4.14.11] | | |
| K01283 | 4 | peptidyl-dipeptidase A [EC:3.4.15.1] | | |
| K01284 | 35 | peptidyl-dipeptidase Dcp [EC:3.4.15.5] | | |
| K01286 | 36 | D-alanyl-D-alanine carboxypeptidase [EC:3.4.16.4] | | |
| K01289 | 13 | carboxypeptidase [EC:3.4.16.-] | | |
| K01295 | 41 | glutamate carboxypeptidase [EC:3.4.17.11] | | |
| K01297 | 11 | muramoyltetrapeptide carboxypeptidase [EC:3.4.17.13] | | |
| K01299 | 20 | carboxypeptidase Taq [EC:3.4.17.19] | | |
| K01301 | 10 | glutamate carboxypeptidase II [EC:3.4.17.21] | | |
| K01302 | 1 | Unclassified; E3.4.17.- | | |
| K01303 | 93 | acylaminoacyl-peptidase [EC:3.4.19.1] | | |
| K01304 | 3 | pyroglutamyl-peptidase [EC:3.4.19.3] | | |
| K01312 | 9 | trypsin [EC:3.4.21.4] | | |
| K01318 | 1 | glutamyl endopeptidase [EC:3.4.21.19] | | |
| K01322 | 103 | prolyl oligopeptidase [EC:3.4.21.26] | | |
| K01325 | 2 | tissue kallikrein [EC:3.4.21.35] | | |
| K01338 | 158 | ATP-dependent Lon protease [EC:3.4.21.53] | | |
| K01341 | 5 | kexin [EC:3.4.21.61] | | |
| K01342 | 54 | subtilisin [EC:3.4.21.62] | | |
| K01344 | 1 | protein C (activated) [EC:3.4.21.69] | | |
| K01347 | 2 | IgA-specific serine endopeptidase [EC:3.4.21.72] | | |
| K01354 | 61 | oligopeptidase B [EC:3.4.21.83] | | |
| K01356 | 28 | repressor LexA [EC:3.4.21.88] | | |
| K01358 | 30 | ATP-dependent Clp protease, protease subunit [EC:3.4.21.92] | | |
| K01361 | 6 | lactocepin [EC:3.4.21.96] | | |
| K01362 | 487 | Unclassified; E3.4.21.- | | |

| | | | | |
|---|---|---|---|---|
| K01372 | 2 | bleomycin hydrolase [EC:3.4.22.40] | | |
| K01376 | 12 | Unclassified; E3.4.22.- | | |
| K01387 | 13 | microbial collagenase [EC:3.4.24.3] | | |
| K01389 | 2 | neprilysin [EC:3.4.24.11] | | |
| K01392 | 9 | thimet oligopeptidase [EC:3.4.24.15] | | |
| K01400 | 2 | bacillolysin [EC:3.4.24.28] | | |
| K01401 | 1 | aureolysin [EC:3.4.24.29] | | |
| K01406 | 35 | serralysin [EC:3.4.24.40] | | |
| K01407 | 1 | protease III [EC:3.4.24.55] | | |
| K01409 | 44 | O-sialoglycoprotein endopeptidase [EC:3.4.24.57] | | |
| K01412 | 104 | mitochondrial processing peptidase [EC:3.4.24.64] | | |
| K01414 | 25 | oligopeptidase A [EC:3.4.24.70] | | |
| K01415 | 46 | endothelin-converting enzyme [EC:3.4.24.71] | | |
| K01416 | 2 | snapalysin [EC:3.4.24.77] | | |
| K01417 | 359 | Unclassified; E3.4.24.- | | |
| K01419 | 10 | ATP-dependent HslUV protease, peptidase subunit HslV [EC:3.4.25.-] | | |
| K01420 | 81 | CRP/FNR family transcriptional regulator, anaerobic regulatory | | |
| K01421 | 2 | putative membrane protein | | |
| K01422 | 101 | Unclassified; E3.4.99.- | | |
| K01423 | 395 | Unclassified; E3.4.-.- | | |
| K01424 | 54 | L-asparaginase [EC:3.5.1.1] | | |
| K01425 | 10 | glutaminase [EC:3.5.1.2] | | |
| K01426 | 156 | amidase [EC:3.5.1.4] | | |
| K01428 | 11 | urease alpha subunit [EC:3.5.1.5] | | |
| K01429 | 3 | urease beta subunit [EC:3.5.1.5] | | |
| K01430 | 6 | urease gamma subunit [EC:3.5.1.5] | | |
| K01431 | 27 | beta-ureidopropionase [EC:3.5.1.6] | | |
| K01433 | 7 | formyltetrahydrofolate deformylase [EC:3.5.1.10] | | |
| K01434 | 69 | penicillin amidase [EC:3.5.1.11] | | |
| K01436 | 74 | aminoacylase [EC:3.5.1.14] | | |
| K01438 | 28 | acetylornithine deacetylase [EC:3.5.1.16] | | |
| K01439 | 82 | succinyl-diaminopimelate desuccinylase [EC:3.5.1.18] | | |
| K01443 | 68 | N-acetylglucosamine-6-phosphate deacetylase [EC:3.5.1.25] | | |
| K01444 | 9 | N4-(beta-N-acetylglucosaminyl)-L-asparaginase [EC:3.5.1.26] | | |
| K01446 | 2 | N-acetylmuramoyl-L-alanine amidase [EC:3.5.1.28] | | |
| K01447 | 31 | N-acetylmuramoyl-L-alanine amidase [EC:3.5.1.28] | | |
| K01448 | 611 | N-acetylmuramoyl-L-alanine amidase [EC:3.5.1.28] | | |
| K01449 | 4 | N-acetylmuramoyl-L-alanine amidase [EC:3.5.1.28] | | |
| K01451 | 45 | hippurate hydrolase [EC:3.5.1.32] | | |
| K01452 | 38 | chitin deacetylase [EC:3.5.1.41] | | |
| K01453 | 19 | Unclassified; E3.5.1.46 | | |
| K01454 | 11 | Unclassified; E3.5.1.48 | | |
| K01455 | 26 | formamidase [EC:3.5.1.49] | | |
| K01457 | 9 | allophanate hydrolase [EC:3.5.1.54] | | |
| K01458 | 6 | N-formylglutamate deformylase [EC:3.5.1.68] | | |
| K01459 | 6 | Unclassified; E3.5.1.77 | | |
| K01461 | 67 | Unclassified; E3.5.1.82 | | |
| K01462 | 44 | Unclassified; E3.5.1.88 | | |
| K01463 | 176 | Unclassified; E3.5.1.- | | |
| K01464 | 42 | dihydropyrimidinase [EC:3.5.2.2] | | |

| | | | | |
|---|---|---|---|---|
| K01465 | 147 | dihydroorotase [EC:3.5.2.3] | | |
| K01466 | 28 | allantoinase [EC:3.5.2.5] | | |
| K01467 | 153 | beta-lactamase [EC:3.5.2.6] | | |
| K01468 | 45 | imidazolonepropionase [EC:3.5.2.7] | | |
| K01469 | 35 | 5-oxoprolinase (ATP-hydrolysing) [EC:3.5.2.9] | | |
| K01470 | 24 | creatinine amidohydrolase [EC:3.5.2.10] | | |
| K01471 | 12 | Unclassified; E3.5.2.12 | | |
| K01473 | 68 | N-methylhydantoinase A [EC:3.5.2.14] | | |
| K01474 | 76 | N-methylhydantoinase B [EC:3.5.2.14] | | |
| K01476 | 13 | arginase [EC:3.5.3.1] | | |
| K01477 | 8 | allantoicase [EC:3.5.3.4] | | |
| K01478 | 10 | arginine deiminase [EC:3.5.3.6] | | |
| K01479 | 4 | formiminoglutamase [EC:3.5.3.8] | | |
| K01480 | 40 | agmatinase [EC:3.5.3.11] | | |
| K01482 | 9 | dimethylargininase [EC:3.5.3.18] | | |
| K01483 | 1 | ureidoglycolate hydrolase [EC:3.5.3.19] | | |
| K01484 | 1 | succinylarginine dihydrolase [EC:3.5.3.23] | | |
| K01485 | 25 | cytosine deaminase [EC:3.5.4.1] | | |
| K01486 | 15 | adenine deaminase [EC:3.5.4.2] | | |
| K01487 | 12 | guanine deaminase [EC:3.5.4.3] | | |
| K01488 | 35 | adenosine deaminase [EC:3.5.4.4] | | |
| K01489 | 21 | cytidine deaminase [EC:3.5.4.5] | | |
| K01491 | 34 | methylenetetrahydrofolate dehydrogenase (NADP+) / | | |
| K01492 | 2 | phosphoribosylaminoimidazolecarboxamide formyltransferase | | |
| K01493 | 13 | dCMP deaminase [EC:3.5.4.12] | | |
| K01494 | 20 | dCTP deaminase [EC:3.5.4.13] | | |
| K01495 | 28 | GTP cyclohydrolase I [EC:3.5.4.16] | | |
| K01496 | 6 | phosphoribosyl-AMP cyclohydrolase [EC:3.5.4.19] | | |
| K01497 | 24 | GTP cyclohydrolase II [EC:3.5.4.25] | | |
| K01498 | 1 | diaminohydroxyphosphoribosylaminopyrimidine deaminase [EC:3.5.4.26] | | |
| K01499 | 3 | methenyltetrahydromethanopterin cyclohydrolase [EC:3.5.4.27] | | |
| K01500 | 18 | Unclassified; E3.5.4.- | | |
| K01501 | 28 | nitrilase [EC:3.5.5.1] | | |
| K01502 | 12 | aliphatic nitrilase [EC:3.5.5.7] | | |
| K01503 | 10 | Unclassified; E3.5.5.- | | |
| K01504 | 6 | glucosamine-6-phosphate isomerase [EC:3.5.99.6] | | |
| K01505 | 4 | 1-aminocyclopropane-1-carboxylate deaminase [EC:3.5.99.7] | | |
| K01506 | 64 | Unclassified; E3.5.-.- | | |
| K01507 | 54 | inorganic pyrophosphatase [EC:3.6.1.1] | | |
| K01512 | 16 | acylphosphatase [EC:3.6.1.7] | | |
| K01514 | 57 | exopolyphosphatase [EC:3.6.1.11] | | |
| K01515 | 41 | ADP-ribose pyrophosphatase [EC:3.6.1.13] | | |
| K01516 | 30 | nucleoside-triphosphatase [EC:3.6.1.15] | | |
| K01518 | 13 | bis(5'-nucleosidyl)-tetraphosphatase [EC:3.6.1.17] | | |
| K01520 | 14 | dUTP pyrophosphatase [EC:3.6.1.23] | | |
| K01523 | 4 | phosphoribosyl-ATP pyrophosphohydrolase [EC:3.6.1.31] | | |
| K01524 | 18 | guanosine-5'-triphosphate,3'-diphosphate pyrophosphatase | | |
| K01525 | 13 | bis(5'-nucleosyl)-tetraphosphatase (symmetrical) [EC:3.6.1.41] | | |
| K01529 | 223 | Unclassified; E3.6.1.- | | |
| K01531 | 33 | Mg2+-importing ATPase [EC:3.6.3.2] | | |

| | | | | |
|---|---|---|---|---|
| K01533 | 135 | Cu2+-exporting ATPase [EC:3.6.3.4] | | |
| K01534 | 28 | Cd2+/Zn2+-exporting ATPase [EC:3.6.3.3 3.6.3.5] | | |
| K01535 | 2 | H+-transporting ATPase [EC:3.6.3.6] | | |
| K01537 | 113 | Ca2+-transporting ATPase [EC:3.6.3.8] | | |
| K01539 | 6 | sodium/potassium-transporting ATPase subunit alpha [EC:3.6.3.9] | | |
| K01546 | 18 | K+-transporting ATPase ATPase A chain [EC:3.6.3.12] | | |
| K01547 | 19 | K+-transporting ATPase ATPase B chain [EC:3.6.3.12] | | |
| K01548 | 14 | K+-transporting ATPase ATPase C chain [EC:3.6.3.12] | | |
| K01551 | 26 | arsenite-transporting ATPase [EC:3.6.3.16] | | |
| K01552 | 141 | Unclassified; E3.6.3.- | | |
| K01554 | 4 | Unclassified; E3.6.-.- | | |
| K01555 | 12 | fumarylacetoacetase [EC:3.7.1.2] | | |
| K01556 | 30 | kynureninase [EC:3.7.1.3] | | |
| K01557 | 5 | acylpyruvate hydrolase [EC:3.7.1.5] | | |
| K01559 | 14 | Unclassified; E3.7.1.- | | |
| K01560 | 34 | 2-haloacid dehalogenase [EC:3.8.1.2] | | |
| K01561 | 20 | haloacetate dehalogenase [EC:3.8.1.3] | | |
| K01563 | 44 | haloalkane dehalogenase [EC:3.8.1.5] | | |
| K01564 | 47 | Unclassified; E3.8.1.- | | |
| K01567 | 169 | Unclassified; E3.-.-.- | | |
| K01568 | 1 | pyruvate decarboxylase [EC:4.1.1.1] | | |
| K01571 | 11 | oxaloacetate decarboxylase, alpha subunit [EC:4.1.1.3] | | |
| K01572 | 9 | oxaloacetate decarboxylase, beta subunit [EC:4.1.1.3] | | |
| K01576 | 25 | benzoylformate decarboxylase [EC:4.1.1.7] | | |
| K01577 | 9 | oxalyl-CoA decarboxylase [EC:4.1.1.8] | | |
| K01578 | 19 | malonyl-CoA decarboxylase [EC:4.1.1.9] | | |
| K01579 | 12 | aspartate 1-decarboxylase [EC:4.1.1.11] | | |
| K01580 | 8 | glutamate decarboxylase [EC:4.1.1.15] | | |
| K01581 | 17 | ornithine decarboxylase [EC:4.1.1.17] | | |
| K01582 | 22 | lysine decarboxylase [EC:4.1.1.18] | | |
| K01583 | 17 | arginine decarboxylase [EC:4.1.1.19] | | |
| K01584 | 17 | arginine decarboxylase [EC:4.1.1.19] | | |
| K01585 | 30 | arginine decarboxylase [EC:4.1.1.19] | | |
| K01586 | 61 | diaminopimelate decarboxylase [EC:4.1.1.20] | | |
| K01588 | 20 | 5-(carboxyamino)imidazole ribonucleotide mutase [EC:5.4.99.18] | | |
| K01589 | 16 | 5-(carboxyamino)imidazole ribonucleotide synthase [EC:6.3.4.18] | | |
| K01591 | 30 | orotidine-5'-phosphate decarboxylase [EC:4.1.1.23] | | |
| K01592 | 14 | tyrosine decarboxylase [EC:4.1.1.25] | | |
| K01593 | 61 | aromatic-L-amino-acid decarboxylase [EC:4.1.1.28] | | |
| K01594 | 14 | sulfinoalanine decarboxylase [EC:4.1.1.29] | | |
| K01595 | 40 | phosphoenolpyruvate carboxylase [EC:4.1.1.31] | | |
| K01596 | 30 | phosphoenolpyruvate carboxykinase (GTP) [EC:4.1.1.32] | | |
| K01598 | 9 | phosphopantothenoylcysteine decarboxylase [EC:4.1.1.36] | | |
| K01599 | 38 | uroporphyrinogen decarboxylase [EC:4.1.1.37] | | |
| K01601 | 7 | ribulose-bisphosphate carboxylase large chain [EC:4.1.1.39] | | |
| K01602 | 3 | ribulose-bisphosphate carboxylase small chain [EC:4.1.1.39] | | |
| K01605 | 1 | methylmalonyl-CoA decarboxylase beta chain [EC:4.1.1.41] | | |
| K01606 | 11 | methylmalonyl-CoA decarboxylase gamma chain [EC:4.1.1.41] | | |
| K01607 | 55 | 4-carboxymuconolactone decarboxylase [EC:4.1.1.44] | | |
| K01608 | 8 | tartronate-semialdehyde synthase [EC:4.1.1.47] | | |

| | | | | |
|---|---|---|---|---|
| K01609 | 19 | indole-3-glycerol phosphate synthase [EC:4.1.1.48] | | |
| K01610 | 22 | phosphoenolpyruvate carboxykinase (ATP) [EC:4.1.1.49] | | |
| K01611 | 4 | S-adenosylmethionine decarboxylase [EC:4.1.1.50] | | |
| K01612 | 5 | 4-hydroxybenzoate decarboxylase [EC:4.1.1.61] | | |
| K01613 | 14 | phosphatidylserine decarboxylase [EC:4.1.1.65] | | |
| K01615 | 12 | glutaconyl-CoA decarboxylase [EC:4.1.1.70] | | |
| K01616 | 9 | 2-oxoglutarate decarboxylase [EC:4.1.1.71] | | |
| K01617 | 5 | 4-oxalocrotonate decarboxylase [EC:4.1.1.77] | | |
| K01618 | 62 | Unclassified; E4.1.1.- | | |
| K01619 | 13 | deoxyribose-phosphate aldolase [EC:4.1.2.4] | | |
| K01620 | 32 | threonine aldolase [EC:4.1.2.5] | | |
| K01621 | 20 | phosphoketolase [EC:4.1.2.9] | | |
| K01623 | 28 | fructose-bisphosphate aldolase, class I [EC:4.1.2.13] | | |
| K01624 | 8 | fructose-bisphosphate aldolase, class II [EC:4.1.2.13] | | |
| K01625 | 11 | 2-dehydro-3-deoxyphosphogluconate aldolase / | | |
| K01626 | 26 | 3-deoxy-7-phosphoheptulonate synthase [EC:2.5.1.54] | | |
| K01627 | 29 | 2-dehydro-3-deoxyphosphooctonate aldolase (KDO 8-P synthase) | | |
| K01628 | 51 | L-fuculose-phosphate aldolase [EC:4.1.2.17] | | |
| K01629 | 6 | rhamnulose-1-phosphate aldolase [EC:4.1.2.19] | | |
| K01630 | 13 | 2-dehydro-3-deoxyglucarate aldolase [EC:4.1.2.20] | | |
| K01631 | 11 | 2-dehydro-3-deoxyphosphogalactonate aldolase [EC:4.1.2.21] | | |
| K01632 | 12 | fructose-6-phosphate phosphoketolase [EC:4.1.2.22] | | |
| K01633 | 13 | dihydroneopterin aldolase [EC:4.1.2.25] | | |
| K01634 | 7 | sphinganine-1-phosphate aldolase [EC:4.1.2.27] | | |
| K01635 | 10 | tagatose 1,6-diphosphate aldolase [EC:4.1.2.40] | | |
| K01636 | 37 | Unclassified; E4.1.2.- | | |
| K01637 | 13 | isocitrate lyase [EC:4.1.3.1] | | |
| K01638 | 23 | malate synthase [EC:2.3.3.9] | | |
| K01639 | 16 | N-acetylneuraminate lyase [EC:4.1.3.3] | | |
| K01640 | 23 | hydroxymethylglutaryl-CoA lyase [EC:4.1.3.4] | | |
| K01641 | 3 | hydroxymethylglutaryl-CoA synthase [EC:2.3.3.10] | | |
| K01643 | 11 | citrate lyase subunit alpha / citrate CoA-transferase [EC:4.1.3.6 | | |
| K01644 | 42 | citrate lyase subunit beta / citryl-CoA lyase [EC:4.1.3.6 4.1.3.34] | | |
| K01647 | 50 | citrate synthase [EC:2.3.3.1] | | |
| K01648 | 1 | ATP citrate (pro-S)-lyase [EC:2.3.3.8] | | |
| K01649 | 82 | 2-isopropylmalate synthase [EC:2.3.3.13] | | |
| K01652 | 178 | acetolactate synthase I/II/III large subunit [EC:2.2.1.6] | | |
| K01653 | 22 | acetolactate synthase I/III small subunit [EC:2.2.1.6] | | |
| K01654 | 14 | N-acetylneuraminate synthase [EC:2.5.1.56] | | |
| K01655 | 8 | homocitrate synthase [EC:2.3.3.14] | | |
| K01657 | 59 | anthranilate synthase component I [EC:4.1.3.27] | | |
| K01658 | 28 | anthranilate synthase component II [EC:4.1.3.27] | | |
| K01659 | 6 | 2-methylcitrate synthase [EC:2.3.3.5] | | |
| K01661 | 17 | naphthoate synthase [EC:4.1.3.36] | | |
| K01662 | 55 | 1-deoxy-D-xylulose-5-phosphate synthase [EC:2.2.1.7] | | |
| K01664 | 14 | para-aminobenzoate synthetase component II [EC:2.6.1.85] | | |
| K01665 | 22 | para-aminobenzoate synthetase component I [EC:2.6.1.85] | | |
| K01666 | 4 | 4-hydroxy 2-oxovalerate aldolase [EC:4.1.3.39] | | |
| K01667 | 15 | tryptophanase [EC:4.1.99.1] | | |
| K01668 | 12 | tyrosine phenol-lyase [EC:4.1.99.2] | | |

| | | | | |
|---|---|---|---|---|
| K01669 | 28 | deoxyribodipyrimidine photo-lyase [EC:4.1.99.3] | | |
| K01673 | 27 | carbonic anhydrase [EC:4.2.1.1] | | |
| K01676 | 23 | fumarate hydratase, class I [EC:4.2.1.2] | | |
| K01677 | 4 | fumarate hydratase subunit alpha [EC:4.2.1.2] | | |
| K01679 | 37 | fumarate hydratase, class II [EC:4.2.1.2] | | |
| K01681 | 81 | aconitate hydratase 1 [EC:4.2.1.3] | | |
| K01682 | 7 | aconitate hydratase 2 [EC:4.2.1.3] | | |
| K01684 | 33 | galactonate dehydratase [EC:4.2.1.6] | | |
| K01685 | 24 | altronate hydrolase [EC:4.2.1.7] | | |
| K01686 | 9 | mannonate dehydratase [EC:4.2.1.8] | | |
| K01687 | 73 | dihydroxy-acid dehydratase [EC:4.2.1.9] | | |
| K01689 | 42 | enolase [EC:4.2.1.11] | | |
| K01690 | 2 | phosphogluconate dehydratase [EC:4.2.1.12] | | |
| K01692 | 266 | enoyl-CoA hydratase [EC:4.2.1.17] | | |
| K01693 | 14 | imidazoleglycerol-phosphate dehydratase [EC:4.2.1.19] | | |
| K01695 | 29 | tryptophan synthase alpha chain [EC:4.2.1.20] | | |
| K01696 | 29 | tryptophan synthase beta chain [EC:4.2.1.20] | | |
| K01697 | 25 | cystathionine beta-synthase [EC:4.2.1.22] | | |
| K01698 | 18 | porphobilinogen synthase [EC:4.2.1.24] | | |
| K01699 | 4 | propanediol dehydratase large subunit [EC:4.2.1.28] | | |
| K01703 | 59 | 3-isopropylmalate/(R)-2-methylmalate dehydratase large subunit | | |
| K01704 | 24 | 3-isopropylmalate/(R)-2-methylmalate dehydratase small subunit | | |
| K01705 | 1 | homoaconitate hydratase [EC:4.2.1.36] | | |
| K01706 | 7 | glucarate dehydratase [EC:4.2.1.40] | | |
| K01708 | 22 | galactarate dehydratase [EC:4.2.1.42] | | |
| K01709 | 15 | CDP-glucose 4,6-dehydratase [EC:4.2.1.45] | | |
| K01710 | 121 | dTDP-glucose 4,6-dehydratase [EC:4.2.1.46] | | |
| K01711 | 34 | GDPmannose 4,6-dehydratase [EC:4.2.1.47] | | |
| K01712 | 17 | urocanate hydratase [EC:4.2.1.49] | | |
| K01713 | 19 | prephenate dehydratase [EC:4.2.1.51] | | |
| K01714 | 67 | dihydrodipicolinate synthase [EC:4.2.1.52] | | |
| K01715 | 61 | 3-hydroxybutyryl-CoA dehydratase [EC:4.2.1.55] | | |
| K01716 | 1 | 3-hydroxydecanoyl-[acyl-carrier-protein] dehydratase [EC:4.2.1.60] | | |
| K01718 | 4 | pseudouridylate synthase [EC:4.2.1.70] | | |
| K01719 | 22 | uroporphyrinogen-III synthase [EC:4.2.1.75] | | |
| K01720 | 63 | 2-methylcitrate dehydratase [EC:4.2.1.79] | | |
| K01722 | 3 | Unclassified; E4.2.1.89 | | |
| K01724 | 20 | 4a-hydroxytetrahydrobiopterin dehydratase [EC:4.2.1.96] | | |
| K01725 | 1 | cyanate lyase [EC:4.2.1.104] | | |
| K01726 | 99 | Unclassified; E4.2.1.- | | |
| K01728 | 1 | pectate lyase [EC:4.2.2.2] | | |
| K01733 | 66 | threonine synthase [EC:4.2.3.1] | | |
| K01734 | 6 | methylglyoxal synthase [EC:4.2.3.3] | | |
| K01735 | 30 | 3-dehydroquinate synthase [EC:4.2.3.4] | | |
| K01736 | 40 | chorismate synthase [EC:4.2.3.5] | | |
| K01737 | 25 | 6-pyruvoyl tetrahydrobiopterin synthase [EC:4.2.3.12] | | |
| K01738 | 73 | cysteine synthase A [EC:2.5.1.47] | | |
| K01739 | 49 | cystathionine gamma-synthase [EC:2.5.1.48] | | |
| K01740 | 36 | O-acetylhomoserine (thiol)-lyase [EC:2.5.1.49] | | |
| K01741 | 3 | DNA-(apurinic or apyrimidinic site) lyase [EC:4.2.99.18] | | |

| | | | | |
|---|---|---|---|---|
| K01743 | 3 | Unclassified; E4.2.-.- | | |
| K01744 | 18 | aspartate ammonia-lyase [EC:4.3.1.1] | | |
| K01745 | 32 | histidine ammonia-lyase [EC:4.3.1.3] | | |
| K01746 | 14 | formiminotetrahydrofolate cyclodeaminase [EC:4.3.1.4] | | |
| K01749 | 28 | hydroxymethylbilane synthase [EC:2.5.1.61] | | |
| K01750 | 39 | ornithine cyclodeaminase [EC:4.3.1.12] | | |
| K01751 | 4 | diaminopropionate ammonia-lyase [EC:4.3.1.15] | | |
| K01752 | 8 | L-serine dehydratase [EC:4.3.1.17] | | |
| K01754 | 80 | threonine dehydratase [EC:4.3.1.19] | | |
| K01755 | 59 | argininosuccinate lyase [EC:4.3.2.1] | | |
| K01756 | 53 | adenylosuccinate lyase [EC:4.3.2.2] | | |
| K01757 | 6 | strictosidine synthase [EC:4.3.3.2] | | |
| K01758 | 28 | cystathionine gamma-lyase [EC:4.4.1.1] | | |
| K01759 | 62 | lactoylglutathione lyase [EC:4.4.1.5] | | |
| K01760 | 59 | cystathionine beta-lyase [EC:4.4.1.8] | | |
| K01761 | 21 | methionine-gamma-lyase [EC:4.4.1.11] | | |
| K01768 | 403 | adenylate cyclase [EC:4.6.1.1] | | |
| K01769 | 7 | guanylate cyclase, other [EC:4.6.1.2] | | |
| K01770 | 23 | 2-C-methyl-D-erythritol 2,4-cyclodiphosphate synthase [EC:4.6.1.12] | | |
| K01772 | 32 | ferrochelatase [EC:4.99.1.1] | | |
| K01774 | 3 | Unclassified; E4.-.-.- | | |
| K01775 | 45 | alanine racemase [EC:5.1.1.1] | | |
| K01776 | 47 | glutamate racemase [EC:5.1.1.3] | | |
| K01777 | 12 | proline racemase [EC:5.1.1.4] | | |
| K01778 | 33 | diaminopimelate epimerase [EC:5.1.1.7] | | |
| K01779 | 30 | aspartate racemase [EC:5.1.1.13] | | |
| K01780 | 26 | Unclassified; E5.1.1.- | | |
| K01781 | 68 | mandelate racemase [EC:5.1.2.2] | | |
| K01782 | 36 | 3-hydroxyacyl-CoA dehydrogenase / enoyl-CoA hydratase / | | |
| K01783 | 21 | ribulose-phosphate 3-epimerase [EC:5.1.3.1] | | |
| K01784 | 233 | UDP-glucose 4-epimerase [EC:5.1.3.2] | | |
| K01785 | 23 | aldose 1-epimerase [EC:5.1.3.3] | | |
| K01786 | 5 | L-ribulose-5-phosphate 4-epimerase [EC:5.1.3.4] | | |
| K01787 | 7 | N-acylglucosamine 2-epimerase [EC:5.1.3.8] | | |
| K01789 | 11 | UDP-glucuronate 5'-epimerase [EC:5.1.3.12] | | |
| K01790 | 33 | dTDP-4-dehydrorhamnose 3,5-epimerase [EC:5.1.3.13] | | |
| K01791 | 43 | UDP-N-acetylglucosamine 2-epimerase [EC:5.1.3.14] | | |
| K01792 | 3 | glucose-6-phosphate 1-epimerase [EC:5.1.3.15] | | |
| K01795 | 128 | Unclassified; E5.1.3.- | | |
| K01796 | 35 | alpha-methylacyl-CoA racemase [EC:5.1.99.4] | | |
| K01797 | 89 | Unclassified; E5.1.99.- | | |
| K01798 | 3 | Unclassified; E5.1.-.- | | |
| K01799 | 14 | maleate isomerase [EC:5.2.1.1] | | |
| K01800 | 11 | maleylacetoacetate isomerase [EC:5.2.1.2] | | |
| K01801 | 8 | maleylpyruvate isomerase [EC:5.2.1.4] | | |
| K01802 | 87 | peptidylprolyl isomerase [EC:5.2.1.8] | | |
| K01803 | 29 | triosephosphate isomerase (TIM) [EC:5.3.1.1] | | |
| K01804 | 10 | L-arabinose isomerase [EC:5.3.1.4] | | |
| K01805 | 13 | xylose isomerase [EC:5.3.1.5] | | |
| K01807 | 12 | ribose 5-phosphate isomerase A [EC:5.3.1.6] | | |

| | | | | |
|---|---|---|---|---|
| K01808 | 26 | ribose 5-phosphate isomerase B [EC:5.3.1.6] | | |
| K01809 | 44 | mannose-6-phosphate isomerase [EC:5.3.1.8] | | |
| K01810 | 53 | glucose-6-phosphate isomerase [EC:5.3.1.9] | | |
| K01811 | 10 | putative family 31 glucosidase | | |
| K01812 | 7 | glucuronate isomerase [EC:5.3.1.12] | | |
| K01813 | 9 | L-rhamnose isomerase [EC:5.3.1.14] | | |
| K01814 | 30 | phosphoribosylformimino-5-aminoimidazole carboxamide ribotide | | |
| K01816 | 12 | hydroxypyruvate isomerase [EC:5.3.1.22] | | |
| K01817 | 27 | phosphoribosylanthranilate isomerase [EC:5.3.1.24] | | |
| K01818 | 6 | L-fucose isomerase [EC:5.3.1.25] | | |
| K01820 | 9 | Unclassified; E5.3.1.- | | |
| K01821 | 5 | 4-oxalocrotonate tautomerase [EC:5.3.2.-] | | |
| K01822 | 1 | steroid delta-isomerase [EC:5.3.3.1] | | |
| K01823 | 12 | isopentenyl-diphosphate delta-isomerase [EC:5.3.3.2] | | |
| K01825 | 4 | 3-hydroxyacyl-CoA dehydrogenase / enoyl-CoA hydratase / | | |
| K01826 | 20 | 5-carboxymethyl-2-hydroxymuconate isomerase [EC:5.3.3.10] | | |
| K01828 | 20 | Unclassified; E5.3.3.- | | |
| K01829 | 20 | protein disulfide-isomerase [EC:5.3.4.1] | | |
| K01834 | 100 | phosphoglycerate mutase [EC:5.4.2.1] | | |
| K01835 | 41 | phosphoglucomutase [EC:5.4.2.2] | | |
| K01838 | 4 | beta-phosphoglucomutase [EC:5.4.2.6] | | |
| K01839 | 18 | phosphopentomutase [EC:5.4.2.7] | | |
| K01840 | 87 | phosphomannomutase [EC:5.4.2.8] | | |
| K01841 | 3 | phosphoenolpyruvate phosphomutase [EC:5.4.2.9] | | |
| K01842 | 5 | Unclassified; E5.4.2.- | | |
| K01843 | 25 | lysine 2,3-aminomutase [EC:5.4.3.2] | | |
| K01844 | 3 | beta-lysine 5,6-aminomutase [EC:5.4.3.3] | | |
| K01845 | 65 | glutamate-1-semialdehyde 2,1-aminomutase [EC:5.4.3.8] | | |
| K01846 | 4 | methylaspartate mutase [EC:5.4.99.1] | | |
| K01847 | 14 | methylmalonyl-CoA mutase [EC:5.4.99.2] | | |
| K01848 | 52 | methylmalonyl-CoA mutase, N-terminal domain [EC:5.4.99.2] | | |
| K01849 | 12 | methylmalonyl-CoA mutase, C-terminal domain [EC:5.4.99.2] | | |
| K01851 | 1 | salicylate biosynthesis isochorismate synthase [EC:5.4.4.2] | | |
| K01854 | 46 | UDP-galactopyranose mutase [EC:5.4.99.9] | | |
| K01856 | 36 | muconate cycloisomerase [EC:5.5.1.1] | | |
| K01857 | 15 | 3-carboxy-cis,cis-muconate cycloisomerase [EC:5.5.1.2] | | |
| K01858 | 12 | myo-inositol-1-phosphate synthase [EC:5.5.1.4] | | |
| K01860 | 15 | chloromuconate cycloisomerase [EC:5.5.1.7] | | |
| K01865 | 17 | Unclassified; E5.-.-.- | | |
| K01866 | 51 | tyrosyl-tRNA synthetase [EC:6.1.1.1] | | |
| K01867 | 46 | tryptophanyl-tRNA synthetase [EC:6.1.1.2] | | |
| K01868 | 67 | threonyl-tRNA synthetase [EC:6.1.1.3] | | |
| K01869 | 73 | leucyl-tRNA synthetase [EC:6.1.1.4] | | |
| K01870 | 85 | isoleucyl-tRNA synthetase [EC:6.1.1.5] | | |
| K01872 | 71 | alanyl-tRNA synthetase [EC:6.1.1.7] | | |
| K01873 | 99 | valyl-tRNA synthetase [EC:6.1.1.9] | | |
| K01874 | 56 | methionyl-tRNA synthetase [EC:6.1.1.10] | | |
| K01875 | 59 | seryl-tRNA synthetase [EC:6.1.1.11] | | |
| K01876 | 57 | aspartyl-tRNA synthetase [EC:6.1.1.12] | | |
| K01878 | 12 | glycyl-tRNA synthetase alpha chain [EC:6.1.1.14] | | |

| | | | | |
|---|---|---|---|---|
| K01879 | 36 | glycyl-tRNA synthetase beta chain [EC:6.1.1.14] | | |
| K01880 | 17 | glycyl-tRNA synthetase [EC:6.1.1.14] | | |
| K01881 | 50 | prolyl-tRNA synthetase [EC:6.1.1.15] | | |
| K01883 | 69 | cysteinyl-tRNA synthetase [EC:6.1.1.16] | | |
| K01885 | 74 | glutamyl-tRNA synthetase [EC:6.1.1.17] | | |
| K01886 | 28 | glutaminyl-tRNA synthetase [EC:6.1.1.18] | | |
| K01887 | 498 | arginyl-tRNA synthetase [EC:6.1.1.19] | | |
| K01889 | 40 | phenylalanyl-tRNA synthetase alpha chain [EC:6.1.1.20] | | |
| K01890 | 515 | phenylalanyl-tRNA synthetase beta chain [EC:6.1.1.20] | | |
| K01892 | 46 | histidyl-tRNA synthetase [EC:6.1.1.21] | | |
| K01893 | 25 | asparaginyl-tRNA synthetase [EC:6.1.1.22] | | |
| K01894 | 20 | glutamyl-Q tRNA(Asp) synthetase [EC:6.1.1.-] | | |
| K01895 | 175 | acetyl-CoA synthetase [EC:6.2.1.1] | | |
| K01897 | 294 | long-chain acyl-CoA synthetase [EC:6.2.1.3] | | |
| K01902 | 19 | succinyl-CoA synthetase alpha subunit [EC:6.2.1.5] | | |
| K01903 | 30 | succinyl-CoA synthetase beta subunit [EC:6.2.1.5] | | |
| K01904 | 37 | 4-coumarate--CoA ligase [EC:6.2.1.12] | | |
| K01905 | 37 | acetyl-CoA synthetase (ADP-forming) [EC:6.2.1.13] | | |
| K01906 | 43 | 6-carboxyhexanoate--CoA ligase [EC:6.2.1.14] | | |
| K01907 | 38 | acetoacetyl-CoA synthetase [EC:6.2.1.16] | | |
| K01908 | 12 | propionyl-CoA synthetase [EC:6.2.1.17] | | |
| K01911 | 22 | O-succinylbenzoic acid--CoA ligase [EC:6.2.1.26] | | |
| K01912 | 88 | phenylacetate-CoA ligase [EC:6.2.1.30] | | |
| K01913 | 125 | Unclassified; E6.2.1.- | | |
| K01914 | 7 | aspartate--ammonia ligase [EC:6.3.1.1] | | |
| K01915 | 141 | glutamine synthetase [EC:6.3.1.2] | | |
| K01916 | 33 | NAD+ synthase [EC:6.3.1.5] | | |
| K01917 | 15 | glutathionylspermidine synthase [EC:6.3.1.8] | | |
| K01918 | 21 | pantoate--beta-alanine ligase [EC:6.3.2.1] | | |
| K01919 | 16 | glutamate--cysteine ligase [EC:6.3.2.2] | | |
| K01920 | 20 | glutathione synthase [EC:6.3.2.3] | | |
| K01921 | 88 | D-alanine-D-alanine ligase [EC:6.3.2.4] | | |
| K01922 | 6 | phosphopantothenate-cysteine ligase [EC:6.3.2.5] | | |
| K01923 | 35 | phosphoribosylaminoimidazole-succinocarboxamide synthase | | |
| K01924 | 35 | UDP-N-acetylmuramate--alanine ligase [EC:6.3.2.8] | | |
| K01925 | 33 | UDP-N-acetylmuramoylalanine--D-glutamate ligase [EC:6.3.2.9] | | |
| K01926 | 7 | AT-rich DNA-binding protein | | |
| K01928 | 42 | UDP-N-acetylmuramoylalanyl-D-glutamate--2,6-diaminopimelate ligase | | |
| K01929 | 58 | UDP-N-acetylmuramoylalanyl-D-glutamyl-2,6-diaminopimelate--D-alanyl- | | |
| K01932 | 7 | Unclassified; E6.3.2.- | | |
| K01933 | 32 | phosphoribosylformylglycinamidine cyclo-ligase [EC:6.3.3.1] | | |
| K01934 | 15 | 5-formyltetrahydrofolate cyclo-ligase [EC:6.3.3.2] | | |
| K01935 | 11 | dethiobiotin synthetase [EC:6.3.3.3] | | |
| K01937 | 42 | CTP synthase [EC:6.3.4.2] | | |
| K01938 | 23 | formate--tetrahydrofolate ligase [EC:6.3.4.3] | | |
| K01939 | 47 | adenylosuccinate synthase [EC:6.3.4.4] | | |
| K01940 | 34 | argininosuccinate synthase [EC:6.3.4.5] | | |
| K01941 | 8 | urea carboxylase [EC:6.3.4.6] | | |
| K01945 | 31 | phosphoribosylamine--glycine ligase [EC:6.3.4.13] | | |
| K01947 | 4 | biotin-[acetyl-CoA-carboxylase] ligase / type III pantothenate | | |

| | | | | |
|---|---|---|---|---|
| K01950 | 63 | NAD+ synthase (glutamine-hydrolysing) [EC:6.3.5.1] | | |
| K01951 | 50 | GMP synthase (glutamine-hydrolysing) [EC:6.3.5.2] | | |
| K01952 | 89 | phosphoribosylformylglycinamidine synthase [EC:6.3.5.3] | | |
| K01953 | 160 | asparagine synthase (glutamine-hydrolysing) [EC:6.3.5.4] | | |
| K01955 | 66 | carbamoyl-phosphate synthase large subunit [EC:6.3.5.5] | | |
| K01956 | 39 | carbamoyl-phosphate synthase small subunit [EC:6.3.5.5] | | |
| K01957 | 10 | glutamyl-tRNA (Gln) amidotransferase [EC:6.3.5.-] | | |
| K01958 | 22 | pyruvate carboxylase [EC:6.4.1.1] | | |
| K01959 | 2 | pyruvate carboxylase subunit A [EC:6.4.1.1] | | |
| K01960 | 23 | pyruvate carboxylase subunit B [EC:6.4.1.1] | | |
| K01961 | 38 | acetyl-CoA carboxylase, biotin carboxylase subunit [EC:6.4.1.2 | | |
| K01962 | 21 | acetyl-CoA carboxylase carboxyl transferase subunit alpha | | |
| K01963 | 31 | acetyl-CoA carboxylase carboxyl transferase subunit beta | | |
| K01965 | 36 | propionyl-CoA carboxylase alpha chain [EC:6.4.1.3] | | |
| K01966 | 69 | propionyl-CoA carboxylase beta chain [EC:6.4.1.3] | | |
| K01968 | 21 | 3-methylcrotonyl-CoA carboxylase alpha subunit [EC:6.4.1.4] | | |
| K01969 | 19 | 3-methylcrotonyl-CoA carboxylase beta subunit [EC:6.4.1.4] | | |
| K01970 | 3 | Unclassified; E6.4.-.- | | |
| K01971 | 172 | DNA ligase (ATP) [EC:6.5.1.1] | | |
| K01972 | 63 | DNA ligase (NAD+) [EC:6.5.1.2] | | |
| K01974 | 6 | RNA 3'-terminal phosphate cyclase [EC:6.5.1.4] | | |
| K01975 | 8 | 2'-5' RNA ligase [EC:6.5.1.-] | | |
| K01976 | 26 | Unclassified; E6.-.-.- | | |
| K01989 | 47 | putative ABC transport system substrate-binding protein | | |
| K01990 | 199 | ABC-2 type transport system ATP-binding protein | | |
| K01991 | 50 | polysaccharide export outer membrane protein | | |
| K01992 | 215 | ABC-2 type transport system permease protein | | |
| K01993 | 36 | HlyD family secretion protein | | |
| K01994 | 1 | LuxR family transcriptional regulator, transcriptional regulator of | | |
| K01995 | 126 | branched-chain amino acid transport system ATP-binding protein | | |
| K01996 | 125 | branched-chain amino acid transport system ATP-binding protein | | |
| K01997 | 153 | branched-chain amino acid transport system permease protein | | |
| K01998 | 326 | branched-chain amino acid transport system permease protein | | |
| K01999 | 254 | branched-chain amino acid transport system substrate-binding protein | | |
| K02000 | 5 | glycine betaine/proline transport system ATP-binding protein | | |
| K02001 | 2 | glycine betaine/proline transport system permease protein | | |
| K02002 | 2 | glycine betaine/proline transport system substrate-binding protein | | |
| K02003 | 126 | Unclassified; ABC.CD.A | | |
| K02004 | 274 | Unclassified; ABC.CD.P | | |
| K02005 | 128 | HlyD family secretion protein | | |
| K02006 | 28 | cobalt/nickel transport system ATP-binding protein | | |
| K02007 | 5 | cobalt/nickel transport system permease protein | | |
| K02008 | 17 | cobalt/nickel transport system permease protein | | |
| K02010 | 19 | iron(III) transport system ATP-binding protein [EC:3.6.3.30] | | |
| K02011 | 71 | iron(III) transport system permease protein | | |
| K02012 | 46 | iron(III) transport system substrate-binding protein | | |
| K02013 | 25 | iron complex transport system ATP-binding protein [EC:3.6.3.34] | | |
| K02014 | 205 | iron complex outermembrane recepter protein | | |
| K02015 | 23 | iron complex transport system permease protein | | |
| K02016 | 43 | iron complex transport system substrate-binding protein | | |

| | | | | |
|---|---|---|---|---|
| K02017 | 11 | molybdate transport system ATP-binding protein [EC:3.6.3.29] | | |
| K02018 | 15 | molybdate transport system permease protein | | |
| K02019 | 7 | molybdate transport system regulatory protein | | |
| K02020 | 55 | molybdate transport system substrate-binding protein | | |
| K02021 | 41 | putative ABC transport system ATP-binding protein | | |
| K02022 | 18 | Unclassified; ABC.MR.TX | | |
| K02023 | 24 | multiple sugar transport system ATP-binding protein | | |
| K02024 | 1 | maltoporin | | |
| K02025 | 139 | multiple sugar transport system permease protein | | |
| K02026 | 137 | multiple sugar transport system permease protein | | |
| K02027 | 138 | multiple sugar transport system substrate-binding protein | | |
| K02028 | 18 | polar amino acid transport system ATP-binding protein [EC:3.6.3.21] | | |
| K02029 | 26 | polar amino acid transport system permease protein | | |
| K02030 | 96 | polar amino acid transport system substrate-binding protein | | |
| K02031 | 66 | peptide/nickel transport system ATP-binding protein | | |
| K02032 | 185 | peptide/nickel transport system ATP-binding protein | | |
| K02033 | 207 | peptide/nickel transport system permease protein | | |
| K02034 | 165 | peptide/nickel transport system permease protein | | |
| K02035 | 407 | peptide/nickel transport system substrate-binding protein | | |
| K02036 | 24 | phosphate transport system ATP-binding protein [EC:3.6.3.27] | | |
| K02037 | 43 | phosphate transport system permease protein | | |
| K02038 | 42 | phosphate transport system permease protein | | |
| K02039 | 31 | phosphate transport system protein | | |
| K02040 | 62 | phosphate transport system substrate-binding protein | | |
| K02041 | 11 | phosphonate transport system ATP-binding protein | | |
| K02042 | 4 | phosphonate transport system permease protein | | |
| K02044 | 24 | phosphonate transport system substrate-binding protein | | |
| K02045 | 11 | sulfate transport system ATP-binding protein [EC:3.6.3.25] | | |
| K02046 | 14 | sulfate transport system permease protein | | |
| K02047 | 9 | sulfate transport system permease protein | | |
| K02048 | 16 | sulfate transport system substrate-binding protein | | |
| K02049 | 46 | sulfonate/nitrate/taurine transport system ATP-binding protein | | |
| K02050 | 86 | sulfonate/nitrate/taurine transport system permease protein | | |
| K02051 | 109 | sulfonate/nitrate/taurine transport system substrate-binding protein | | |
| K02052 | 32 | putative spermidine/putrescine transport system ATP-binding protein | | |
| K02053 | 39 | putative spermidine/putrescine transport system permease protein | | |
| K02054 | 31 | putative spermidine/putrescine transport system permease protein | | |
| K02055 | 46 | putative spermidine/putrescine transport system substrate-binding | | |
| K02056 | 66 | simple sugar transport system ATP-binding protein [EC:3.6.3.17] | | |
| K02057 | 106 | simple sugar transport system permease protein | | |
| K02058 | 54 | simple sugar transport system substrate-binding protein | | |
| K02059 | 4 | putative sulfate transport system ATP-binding protein | | |
| K02060 | 7 | putative sulfate transport system permease protein | | |
| K02061 | 3 | putative sulfate transport system substrate-binding protein | | |
| K02063 | 7 | thiamine transport system permease protein | | |
| K02064 | 3 | thiamine transport system substrate-binding protein | | |
| K02065 | 37 | putative ABC transport system ATP-binding protein | | |
| K02066 | 51 | putative ABC transport system permease protein | | |
| K02067 | 57 | putative ABC transport system substrate-binding protein | | |
| K02068 | 1 | putative ABC transport system ATP-binding protein | | |

| | | | | |
|---|---|---|---|---|
| K02069 | 5 | putative ABC transport system permease protein | | |
| K02071 | 4 | D-methionine transport system ATP-binding protein | | |
| K02073 | 2 | D-methionine transport system substrate-binding protein | | |
| K02074 | 9 | zinc/manganese transport system ATP-binding protein | | |
| K02075 | 17 | zinc/manganese transport system permease protein | | |
| K02076 | 2 | Fur family transcriptional regulator, zinc uptake regulator | | |
| K02077 | 30 | zinc/manganese transport system substrate-binding protein | | |
| K02078 | 15 | acyl carrier protein | | |
| K02079 | 2 | N-acetylgalactosamine-6-phosphate deacetylase [EC:3.5.1.25] | | |
| K02081 | 13 | DeoR family transcriptional regulator, aga operon transcriptional | | |
| K02082 | 2 | tagatose-6-phosphate ketose/aldose isomerase [EC:5.-.-.-] | | |
| K02083 | 5 | allantoate deiminase [EC:3.5.3.9] | | |
| K02099 | 3 | AraC family transcriptional regulator, arabinose operon regulatory | | |
| K02103 | 1 | GntR family transcriptional regulator, arabinose operon | | |
| K02106 | 4 | short-chain fatty acids transporter | | |
| K02108 | 50 | F-type H+-transporting ATPase subunit a [EC:3.6.3.14] | | |
| K02109 | 12 | F-type H+-transporting ATPase subunit b [EC:3.6.3.14] | | |
| K02110 | 10 | F-type H+-transporting ATPase subunit c [EC:3.6.3.14] | | |
| K02111 | 32 | F-type H+-transporting ATPase subunit alpha [EC:3.6.3.14] | | |
| K02112 | 21 | F-type H+-transporting ATPase subunit beta [EC:3.6.3.14] | | |
| K02113 | 26 | F-type H+-transporting ATPase subunit delta [EC:3.6.3.14] | | |
| K02114 | 7 | F-type H+-transporting ATPase subunit epsilon [EC:3.6.3.14] | | |
| K02115 | 35 | F-type H+-transporting ATPase subunit gamma [EC:3.6.3.14] | | |
| K02116 | 2 | ATP synthase protein I | | |
| K02117 | 20 | V-type H+-transporting ATPase subunit A [EC:3.6.3.14] | | |
| K02118 | 13 | V-type H+-transporting ATPase subunit B [EC:3.6.3.14] | | |
| K02120 | 3 | V-type H+-transporting ATPase subunit D [EC:3.6.3.14] | | |
| K02121 | 2 | V-type H+-transporting ATPase subunit E [EC:3.6.3.14] | | |
| K02123 | 15 | V-type H+-transporting ATPase subunit I [EC:3.6.3.14] | | |
| K02124 | 4 | V-type H+-transporting ATPase subunit K [EC:3.6.3.14] | | |
| K02160 | 11 | acetyl-CoA carboxylase biotin carboxyl carrier protein | | |
| K02164 | 4 | nitric-oxide reductase NorE protein [EC:1.7.99.7] | | |
| K02169 | 17 | biotin synthesis protein BioC | | |
| K02170 | 4 | biotin biosynthesis protein BioH | | |
| K02182 | 16 | crotonobetaine/carnitine-CoA ligase [EC:6.2.1.-] | | |
| K02188 | 4 | cobalamin biosynthesis protein CbiD | | |
| K02189 | 1 | cobalamin biosynthesis protein CbiG | | |
| K02193 | 29 | heme exporter protein A [EC:3.6.3.41] | | |
| K02194 | 15 | heme exporter protein B | | |
| K02195 | 42 | heme exporter protein C | | |
| K02197 | 10 | cytochrome c-type biogenesis protein CcmE | | |
| K02198 | 81 | cytochrome c-type biogenesis protein CcmF | | |
| K02199 | 52 | cytochrome c biogenesis protein CcmG, thiol:disulfide interchange | | |
| K02200 | 19 | cytochrome c-type biogenesis protein CcmH | | |
| K02203 | 6 | phosphoserine / homoserine phosphotransferase [EC:3.1.3.3 2.7.1.39] | | |
| K02204 | 5 | homoserine kinase type II [EC:2.7.1.39] | | |
| K02217 | 12 | ferritin [EC:1.16.3.1] | | |
| K02221 | 26 | YggT family protein | | |
| K02224 | 18 | cobyrinic acid a,c-diamide synthase [EC:6.3.5.9 6.3.1.-] | | |
| K02226 | 2 | alpha-ribazole phosphatase [EC:3.1.3.73] | | |

| | | | | |
|---|---|---|---|---|
| K02227 | 11 | adenosylcobinamide-phosphate synthase CobD [EC:6.3.1.10] | | |
| K02229 | 1 | precorrin-3B synthase [EC:1.14.13.83] | | |
| K02230 | 13 | cobaltochelatase CobN [EC:6.6.1.2] | | |
| K02231 | 13 | adenosylcobinamide kinase / adenosylcobinamide-phosphate | | |
| K02232 | 9 | adenosylcobyric acid synthase [EC:6.3.5.10] | | |
| K02233 | 2 | adenosylcobinamide-GDP ribazoletransferase [EC:2.7.8.26] | | |
| K02234 | 13 | cobalamin biosynthesis protein CobW | | |
| K02236 | 9 | leader peptidase (prepilin peptidase) / N-methyltransferase | | |
| K02237 | 16 | competence protein ComEA | | |
| K02238 | 57 | competence protein ComEC | | |
| K02240 | 1 | competence protein ComFA | | |
| K02242 | 4 | competence protein ComFC | | |
| K02244 | 3 | competence protein ComGB | | |
| K02248 | 3 | competence protein ComGF | | |
| K02258 | 6 | cytochrome c oxidase subunit XI assembly protein | | |
| K02259 | 27 | cytochrome c oxidase subunit XV assembly protein | | |
| K02274 | 88 | cytochrome c oxidase subunit I [EC:1.9.3.1] | | |
| K02275 | 66 | cytochrome c oxidase subunit II [EC:1.9.3.1] | | |
| K02276 | 37 | cytochrome c oxidase subunit III [EC:1.9.3.1] | | |
| K02278 | 1 | prepilin peptidase CpaA [EC:3.4.23.43] | | |
| K02279 | 5 | pilus assembly protein CpaB | | |
| K02280 | 15 | pilus assembly protein CpaC | | |
| K02282 | 37 | pilus assembly protein CpaE | | |
| K02283 | 46 | pilus assembly protein CpaF | | |
| K02287 | 2 | phycocyanin-associated, rod | | |
| K02288 | 2 | phycocyanobilin lyase alpha subunit [EC:4.-.-.-] | | |
| K02291 | 30 | phytoene synthase [EC:2.5.1.32] | | |
| K02292 | 50 | beta-carotene ketolase (CrtO type) | | |
| K02293 | 3 | phytoene dehydrogenase, phytoene desaturase [EC:1.14.99.-] | | |
| K02297 | 3 | cytochrome o ubiquinol oxidase subunit II [EC:1.10.3.-] | | |
| K02298 | 6 | cytochrome o ubiquinol oxidase subunit I [EC:1.10.3.-] | | |
| K02299 | 4 | cytochrome o ubiquinol oxidase subunit III [EC:1.10.3.-] | | |
| K02301 | 31 | protoheme IX farnesyltransferase [EC:2.5.1.-] | | |
| K02302 | 27 | uroporphyrin-III C-methyltransferase / precorrin-2 dehydrogenase / | | |
| K02303 | 22 | uroporphyrin-III C-methyltransferase [EC:2.1.1.107] | | |
| K02304 | 6 | precorrin-2 dehydrogenase / sirohydrochlorin ferrochelatase | | |
| K02305 | 3 | nitric-oxide reductase, cytochrome c-containing subunit II | | |
| K02312 | 5 | 2,3-dihydroxybenzoate-AMP ligase [EC:2.7.7.58] | | |
| K02313 | 51 | chromosomal replication initiator protein | | |
| K02314 | 78 | replicative DNA helicase [EC:3.6.1.-] | | |
| K02315 | 8 | DNA replication protein DnaC | | |
| K02316 | 65 | DNA primase [EC:2.7.7.-] | | |
| K02319 | 11 | DNA polymerase I [EC:2.7.7.7] | | |
| K02330 | 6 | DNA polymerase beta subunit [EC:2.7.7.7 4.2.99.-] | | |
| K02334 | 43 | DNA polymerase bacteriophage-type [EC:2.7.7.7] | | |
| K02335 | 90 | DNA polymerase I [EC:2.7.7.7] | | |
| K02336 | 4 | DNA polymerase II [EC:2.7.7.7] | | |
| K02337 | 211 | DNA polymerase III subunit alpha [EC:2.7.7.7] | | |
| K02338 | 59 | DNA polymerase III subunit beta [EC:2.7.7.7] | | |
| K02339 | 6 | DNA polymerase III subunit chi [EC:2.7.7.7] | | |

| | | | | |
|---|---|---|---|---|
| K02340 | 11 | DNA polymerase III subunit delta [EC:2.7.7.7] | | |
| K02341 | 35 | DNA polymerase III subunit delta' [EC:2.7.7.7] | | |
| K02342 | 60 | DNA polymerase III subunit epsilon [EC:2.7.7.7] | | |
| K02343 | 65 | DNA polymerase III subunit gamma/tau [EC:2.7.7.7] | | |
| K02345 | 1 | DNA polymerase III subunit theta [EC:2.7.7.7] | | |
| K02346 | 46 | DNA polymerase IV [EC:2.7.7.7] | | |
| K02347 | 45 | DNA polymerase (family X) | | |
| K02348 | 2 | ElaA protein | | |
| K02351 | 7 | putative membrane protein | | |
| K02352 | 1 | drp35 | | |
| K02355 | 154 | elongation factor EF-G [EC:3.6.5.3] | | |
| K02356 | 32 | elongation factor EF-P | | |
| K02357 | 44 | elongation factor EF-Ts | | |
| K02358 | 138 | elongation factor EF-Tu [EC:3.6.5.3] | | |
| K02361 | 19 | isochorismate synthase [EC:5.4.4.2] | | |
| K02363 | 1 | enterobactin 2,3-dihydroxybenzoate-AMP ligase / | | |
| K02364 | 7 | enterobactin synthetase component F [EC:2.7.7.-] | | |
| K02371 | 8 | enoyl-[acyl carrier protein] reductase II [EC:1.3.1.-] | | |
| K02372 | 22 | 3R-hydroxymyristoyl ACP dehydrase [EC:4.2.1.-] | | |
| K02377 | 24 | GDP-L-fucose synthase [EC:1.1.1.271] | | |
| K02379 | 19 | FdhD protein | | |
| K02380 | 5 | FdhE protein | | |
| K02381 | 6 | FdrA protein | | |
| K02382 | 3 | flagellar protein FlbA | | |
| K02384 | 3 | flagellar protein FlbC | | |
| K02386 | 1 | flagella basal body P-ring formation protein FlgA | | |
| K02387 | 12 | flagellar basal-body rod protein FlgB | | |
| K02388 | 133 | flagellar basal-body rod protein FlgC | | |
| K02389 | 14 | flagellar basal-body rod modification protein FlgD | | |
| K02390 | 14 | flagellar hook protein FlgE | | |
| K02391 | 4 | flagellar basal-body rod protein FlgF | | |
| K02392 | 15 | flagellar basal-body rod protein FlgG | | |
| K02393 | 4 | flagellar L-ring protein precursor FlgH | | |
| K02394 | 6 | flagellar P-ring protein precursor FlgI | | |
| K02395 | 11 | flagellar protein FlgJ | | |
| K02396 | 12 | flagellar hook-associated protein 1 FlgK | | |
| K02397 | 8 | flagellar hook-associated protein 3 FlgL | | |
| K02400 | 16 | flagellar biosynthesis protein FlhA | | |
| K02401 | 8 | flagellar biosynthetic protein FlhB | | |
| K02402 | 1 | flagellar transcriptional activator FlhC | | |
| K02404 | 6 | flagellar biosynthesis protein FlhF | | |
| K02405 | 21 | RNA polymerase sigma factor for flagellar operon FliA | | |
| K02406 | 10 | flagellin | | |
| K02407 | 17 | flagellar hook-associated protein 2 | | |
| K02408 | 2 | flagellar hook-basal body complex protein FliE | | |
| K02409 | 14 | flagellar M-ring protein FliF | | |
| K02410 | 16 | flagellar motor switch protein FliG | | |
| K02411 | 3 | flagellar assembly protein FliH | | |
| K02412 | 12 | flagellum-specific ATP synthase [EC:3.6.3.14] | | |
| K02413 | 1 | flagellar FliJ protein | | |

| | | | | |
|---|---|---|---|---|
| K02414 | 1 | flagellar hook-length control protein FliK | | |
| K02415 | 3 | flagellar FliL protein | | |
| K02416 | 15 | flagellar motor switch protein FliM | | |
| K02417 | 6 | flagellar motor switch protein FliN/FliY | | |
| K02418 | 2 | flagellar protein FliO/FliZ | | |
| K02419 | 7 | flagellar biosynthetic protein FliP | | |
| K02420 | 2 | flagellar biosynthetic protein FliQ | | |
| K02421 | 2 | flagellar biosynthetic protein FliR | | |
| K02422 | 3 | flagellar protein FliS | | |
| K02424 | 2 | cystine transport system substrate-binding protein | | |
| K02426 | 10 | cysteine desulfuration protein SufE | | |
| K02427 | 13 | ribosomal RNA large subunit methyltransferase E [EC:2.1.1.-] | | |
| K02428 | 30 | nucleoside-triphosphate pyrophosphatase [EC:3.6.1.19] | | |
| K02429 | 11 | MFS transporter, FHS family, L-fucose permease | | |
| K02431 | 1 | L-fucose mutarotase [EC:5.1.3.-] | | |
| K02433 | 141 | aspartyl-tRNA(Asn)/glutamyl-tRNA (Gln) amidotransferase subunit A | | |
| K02434 | 54 | aspartyl-tRNA(Asn)/glutamyl-tRNA (Gln) amidotransferase subunit B | | |
| K02435 | 18 | aspartyl-tRNA(Asn)/glutamyl-tRNA (Gln) amidotransferase subunit C | | |
| K02436 | 2 | DeoR family transcriptional regulator, galactitol utilization operon | | |
| K02437 | 29 | glycine cleavage system H protein | | |
| K02438 | 33 | glycogen operon protein GlgX [EC:3.2.1.-] | | |
| K02439 | 2 | thiosulfate sulfurtransferase [EC:2.8.1.1] | | |
| K02440 | 14 | glycerol uptake facilitator protein | | |
| K02441 | 1 | GlpG protein | | |
| K02444 | 5 | DeoR family transcriptional regulator, glycerol-3-phosphate regulon | | |
| K02445 | 1 | MFS transporter, OPA family, glycerol-3-phosphate transporter | | |
| K02446 | 28 | fructose-1,6-bisphosphatase II [EC:3.1.3.11] | | |
| K02448 | 3 | nitric-oxide reductase NorD protein [EC:1.7.99.7] | | |
| K02450 | 12 | general secretion pathway protein A | | |
| K02452 | 1 | general secretion pathway protein C | | |
| K02453 | 90 | general secretion pathway protein D | | |
| K02454 | 48 | general secretion pathway protein E | | |
| K02455 | 102 | general secretion pathway protein F | | |
| K02456 | 16 | general secretion pathway protein G | | |
| K02457 | 2 | general secretion pathway protein H | | |
| K02458 | 2 | general secretion pathway protein I | | |
| K02459 | 2 | general secretion pathway protein J | | |
| K02460 | 10 | general secretion pathway protein K | | |
| K02461 | 7 | general secretion pathway protein L | | |
| K02464 | 6 | general secretion pathway protein O [EC:3.4.23.43 2.1.1.-] | | |
| K02469 | 80 | DNA gyrase subunit A [EC:5.99.1.3] | | |
| K02470 | 50 | DNA gyrase subunit B [EC:5.99.1.3] | | |
| K02471 | 16 | putative ATP-binding cassette transporter | | |
| K02472 | 10 | UDP-N-acetyl-D-mannosaminuronic acid dehydrogenase [EC:1.1.1.-] | | |
| K02473 | 35 | UDP-N-acetylglucosamine 4-epimerase [EC:5.1.3.7] | | |
| K02474 | 11 | UDP-N-acetyl-D-galactosamine dehydrogenase [EC:1.1.1.-] | | |
| K02475 | 2 | two-component system, CitB family, response regulator | | |
| K02477 | 67 | two-component system, LytT family, response regulator | | |
| K02478 | 4 | two-component system, LytT family, sensor kinase [EC:2.7.13.3] | | |
| K02479 | 79 | two-component system, NarL family, response regulator | | |

| | | | | |
|---|---|---|---|---|
| K02480 | 32 | two-component system, NarL family, sensor kinase [EC:2.7.13.3] | | |
| K02481 | 134 | two-component system, NtrC family, response regulator | | |
| K02482 | 143 | two-component system, NtrC family, sensor kinase [EC:2.7.13.3] | | |
| K02483 | 128 | two-component system, OmpR family, response regulator | | |
| K02484 | 139 | two-component system, OmpR family, sensor kinase [EC:2.7.13.3] | | |
| K02485 | 11 | two-component system, unclassified family, response regulator | | |
| K02486 | 15 | two-component system, unclassified family, sensor kinase | | |
| K02487 | 1 | type IV pili sensor histidine kinase and response regulator | | |
| K02488 | 217 | two-component system, cell cycle response regulator | | |
| K02489 | 49 | two-component system, cell cycle sensor kinase and response | | |
| K02490 | 30 | two-component system, response regulator, stage 0 sporulation | | |
| K02491 | 8 | two-component system, sporulation sensor kinase A [EC:2.7.13.3] | | |
| K02492 | 42 | glutamyl-tRNA reductase [EC:1.2.1.70] | | |
| K02493 | 32 | methyltransferase [EC:2.1.1.-] | | |
| K02494 | 3 | outer membrane lipoprotein LolB | | |
| K02495 | 53 | oxygen-independent coproporphyrinogen III oxidase [EC:1.3.99.22] | | |
| K02496 | 3 | uroporphyrin-III C-methyltransferase [EC:2.1.1.107] | | |
| K02497 | 2 | HemX protein | | |
| K02498 | 3 | HemY protein | | |
| K02499 | 13 | tetrapyrrole methylase family protein / MazG family protein | | |
| K02500 | 27 | cyclase HisF [EC:4.1.3.-] | | |
| K02501 | 31 | glutamine amidotransferase [EC:2.4.2.-] | | |
| K02502 | 22 | ATP phosphoribosyltransferase regulatory subunit | | |
| K02503 | 30 | Hit-like protein involved in cell-cycle regulation | | |
| K02504 | 1 | protein transport protein HofB | | |
| K02507 | 4 | protein transport protein HofQ | | |
| K02508 | 2 | AraC family transcriptional regulator, 4-hydroxyphenylacetate | | |
| K02509 | 7 | 2-oxo-hept-3-ene-1,7-dioate hydratase [EC:4.2.1.-] | | |
| K02510 | 35 | 2,4-dihydroxyhept-2-ene-1,7-dioic acid aldolase [EC:4.1.2.-] | | |
| K02511 | 1 | MFS transporter, ACS family, 4-hydroxyphenylacetate permease | | |
| K02517 | 28 | lipid A biosynthesis lauroyl acyltransferase [EC:2.3.1.-] | | |
| K02518 | 13 | translation initiation factor IF-1 | | |
| K02519 | 44 | translation initiation factor IF-2 | | |
| K02520 | 31 | translation initiation factor IF-3 | | |
| K02523 | 22 | octaprenyl diphosphate synthase [EC:2.5.1.-] | | |
| K02525 | 1 | LacI family transcriptional regulator, kdg operon repressor | | |
| K02526 | 3 | 2-keto-3-deoxygluconate permease | | |
| K02527 | 16 | 3-deoxy-D-manno-octulosonic-acid transferase [EC:2.-.-.-] | | |
| K02528 | 35 | dimethyladenosine transferase [EC:2.1.1.-] | | |
| K02529 | 80 | LacI family transcriptional regulator | | |
| K02532 | 1 | MFS transporter, OHS family, lactose permease | | |
| K02533 | 19 | tRNA/rRNA methyltransferase [EC:2.1.1.-] | | |
| K02535 | 25 | UDP-3-O-[3-hydroxymyristoyl] N-acetylglucosamine deacetylase | | |
| K02536 | 33 | UDP-3-O-[3-hydroxymyristoyl] glucosamine N-acyltransferase | | |
| K02545 | 2 | penicillin-binding protein 2 prime | | |
| K02547 | 1 | methicillin resistance protein | | |
| K02548 | 10 | 1,4-dihydroxy-2-naphthoate octaprenyltransferase [EC:2.5.1.-] | | |
| K02549 | 20 | O-succinylbenzoate synthase [EC:4.2.1.113] | | |
| K02550 | 1 | glycolate permease | | |
| K02551 | 32 | 2-succinyl-5-enolpyruvyl-6-hydroxy-3-cyclohexene-1-carboxylate | | |

| | | | | |
|---|---|---|---|---|
| K02552 | 12 | menaquinone-specific isochorismate synthase [EC:5.4.4.2] | | |
| K02553 | 1 | regulator of ribonuclease activity A | | |
| K02554 | 5 | 2-keto-4-pentenoate hydratase [EC:4.2.1.80] | | |
| K02556 | 5 | chemotaxis protein MotA | | |
| K02557 | 23 | chemotaxis protein MotB | | |
| K02558 | 24 | UDP-N-acetylmuramate: L-alanyl-gamma-D-glutamyl-meso-diaminopimelate | | |
| K02562 | 2 | mannitol operon repressor | | |
| K02563 | 30 | UDP-N-acetylglucosamine--N-acetylmuramyl-(pentapeptide) | | |
| K02564 | 23 | glucosamine-6-phosphate deaminase [EC:3.5.99.6] | | |
| K02565 | 5 | N-acetylglucosamine repressor | | |
| K02566 | 16 | NagD protein | | |
| K02567 | 28 | periplasmic nitrate reductase NapA [EC:1.7.99.4] | | |
| K02568 | 8 | cytochrome c-type protein NapB | | |
| K02569 | 3 | cytochrome c-type protein NapC | | |
| K02571 | 1 | periplasmic nitrate reductase NapE | | |
| K02572 | 2 | ferredoxin-type protein NapF | | |
| K02573 | 7 | ferredoxin-type protein NapG | | |
| K02574 | 7 | ferredoxin-type protein NapH | | |
| K02575 | 27 | MFS transporter, NNP family, nitrate/nitrite transporter | | |
| K02584 | 38 | Nif-specific regulatory protein | | |
| K02585 | 2 | nitrogen fixation protein NifB | | |
| K02586 | 4 | nitrogenase molybdenum-iron protein alpha chain [EC:1.18.6.1] | | |
| K02587 | 1 | nitrogenase molybdenum-cofactor synthesis protein NifE | | |
| K02588 | 1 | nitrogenase iron protein NifH [EC:1.18.6.1] | | |
| K02591 | 3 | nitrogenase molybdenum-iron protein beta chain [EC:1.18.6.1] | | |
| K02592 | 1 | nitrogenase molybdenum-iron protein NifN | | |
| K02594 | 3 | homocitrate synthase NifV | | |
| K02597 | 5 | nitrogen fixation protein NifZ | | |
| K02598 | 1 | nitrite transporter NirC | | |
| K02600 | 28 | N utilization substance protein A | | |
| K02601 | 16 | transcriptional antiterminator NusG | | |
| K02609 | 16 | phenylacetic acid degradation protein | | |
| K02610 | 4 | phenylacetic acid degradation protein | | |
| K02611 | 10 | phenylacetic acid degradation protein | | |
| K02612 | 14 | phenylacetic acid degradation protein | | |
| K02613 | 11 | phenylacetic acid degradation NADH oxidoreductase | | |
| K02614 | 21 | phenylacetic acid degradation protein | | |
| K02615 | 3 | acetyl-CoA acetyltransferase [EC:2.3.1.-] | | |
| K02616 | 6 | phenylacetic acid degradation operon negative regulatory protein | | |
| K02617 | 6 | phenylacetic acid degradation protein | | |
| K02618 | 19 | phenylacetic acid degradation protein | | |
| K02619 | 44 | 4-amino-4-deoxychorismate lyase [EC:4.1.3.38] | | |
| K02621 | 13 | topoisomerase IV subunit A [EC:5.99.1.-] | | |
| K02622 | 18 | topoisomerase IV subunit B [EC:5.99.1.-] | | |
| K02623 | 10 | LysR family transcriptional regulator, pca operon transcriptional | | |
| K02624 | 13 | IclR family transcriptional regulator, pca regulon regulatory | | |
| K02626 | 5 | arginine decarboxylase [EC:4.1.1.19] | | |
| K02635 | 19 | cytochrome b6 | | |
| K02636 | 14 | cytochrome b6-f complex iron-sulfur subunit [EC:1.10.99.1] | | |
| K02637 | 1 | cytochrome b6-f complex subunit 4 | | |

| | | | | |
|---|---|---|---|---|
| K02638 | 2 | plastocyanin | | |
| K02641 | 2 | ferredoxin--NADP+ reductase [EC:1.18.1.2] | | |
| K02647 | 2 | carbohydrate diacid regulator | | |
| K02650 | 7 | type IV pilus assembly protein PilA | | |
| K02651 | 5 | pilus assembly protein Flp/PilA | | |
| K02652 | 69 | type IV pilus assembly protein PilB | | |
| K02653 | 77 | type IV pilus assembly protein PilC | | |
| K02654 | 24 | leader peptidase (prepilin peptidase) / N-methyltransferase | | |
| K02655 | 6 | type IV pilus assembly protein PilE | | |
| K02656 | 15 | type IV pilus assembly protein PilF | | |
| K02657 | 13 | twitching motility two-component system response regulator PilG | | |
| K02658 | 37 | twitching motility two-component system response regulator PilH | | |
| K02659 | 5 | twitching motility protein PilI | | |
| K02660 | 18 | twitching motility protein PilJ | | |
| K02661 | 2 | type IV pilus assembly protein PilK | | |
| K02662 | 35 | type IV pilus assembly protein PilM | | |
| K02663 | 6 | type IV pilus assembly protein PilN | | |
| K02664 | 9 | type IV pilus assembly protein PilO | | |
| K02665 | 4 | type IV pilus assembly protein PilP | | |
| K02666 | 66 | type IV pilus assembly protein PilQ | | |
| K02667 | 64 | two-component system, NtrC family, response regulator PilR | | |
| K02668 | 66 | two-component system, NtrC family, sensor histidine kinase PilS | | |
| K02669 | 68 | twitching motility protein PilT | | |
| K02670 | 13 | twitching motility protein PilU | | |
| K02671 | 2 | type IV pilus assembly protein PilV | | |
| K02672 | 4 | type IV pilus assembly protein PilW | | |
| K02673 | 7 | type IV pilus assembly protein PilX | | |
| K02674 | 38 | type IV pilus assembly protein PilY1 | | |
| K02676 | 2 | type IV pilus assembly protein PilZ | | |
| K02686 | 4 | primosomal replication protein N | | |
| K02687 | 27 | ribosomal protein L11 methyltransferase [EC:2.1.1.-] | | |
| K02688 | 2 | transcriptional regulator, propionate catabolism operon regulatory | | |
| K02690 | 1 | photosystem I core protein Ib | | |
| K02745 | 1 | PTS system, N-acetylgalactosamine-specific IIB component | | |
| K02755 | 1 | PTS system, beta-glucosides-specific IIA component [EC:2.7.1.69] | | |
| K02759 | 4 | PTS system, cellobiose-specific IIA component [EC:2.7.1.69] | | |
| K02761 | 9 | PTS system, cellobiose-specific IIC component | | |
| K02765 | 3 | PTS system, D-glucosamine-specific IIC component | | |
| K02768 | 6 | PTS system, fructose-specific IIA component [EC:2.7.1.69] | | |
| K02769 | 1 | PTS system, fructose-specific IIB component [EC:2.7.1.69] | | |
| K02770 | 12 | PTS system, fructose-specific IIC component | | |
| K02774 | 5 | PTS system, galactitol-specific IIB component [EC:2.7.1.69] | | |
| K02775 | 2 | PTS system, galactitol-specific IIC component | | |
| K02777 | 109 | PTS system, glucose-specific IIA component [EC:2.7.1.69] | | |
| K02779 | 2 | PTS system, glucose-specific IIC component | | |
| K02784 | 4 | phosphocarrier protein HPr | | |
| K02793 | 6 | PTS system, mannose-specific IIA component [EC:2.7.1.69] | | |
| K02794 | 3 | PTS system, mannose-specific IIB component [EC:2.7.1.69] | | |
| K02795 | 3 | PTS system, mannose-specific IIC component | | |
| K02796 | 6 | PTS system, mannose-specific IID component | | |

| | | | | |
|---|---|---|---|---|
| K02799 | 2 | PTS system, mannitol-specific IIB component [EC:2.7.1.69] | | |
| K02800 | 3 | PTS system, mannitol-specific IIC component | | |
| K02804 | 1 | PTS system, N-acetylglucosamine-specific IIC component | | |
| K02805 | 4 | lipopolysaccharide biosynthesis protein | | |
| K02806 | 15 | PTS system, nitrogen regulatory IIA component [EC:2.7.1.69] | | |
| K02821 | 9 | PTS system, ascorbate-specific IIA component [EC:2.7.1.69] | | |
| K02823 | 18 | dihydroorotate dehydrogenase electron transfer subunit | | |
| K02824 | 6 | uracil permease | | |
| K02825 | 13 | pyrimidine operon attenuation protein / uracil | | |
| K02826 | 1 | quinol oxidase polypeptide II [EC:1.9.3.-] | | |
| K02827 | 1 | quinol oxidase polypeptide I [EC:1.9.3.-] | | |
| K02828 | 1 | quinol oxidase polypeptide III [EC:1.9.3.-] | | |
| K02834 | 16 | ribosome-binding factor A | | |
| K02835 | 26 | peptide chain release factor RF-1 | | |
| K02836 | 23 | peptide chain release factor RF-2 | | |
| K02837 | 18 | peptide chain release factor RF-3 | | |
| K02838 | 30 | ribosome recycling factor | | |
| K02839 | 2 | peptide chain release factor RF-H | | |
| K02841 | 22 | heptosyltransferase I [EC:2.4.-.-] | | |
| K02843 | 38 | heptosyltransferase II [EC:2.4.-.-] | | |
| K02844 | 36 | UDP-glucose:(heptosyl)LPS alpha-1,3-glucosyltransferase [EC:2.4.1.-] | | |
| K02846 | 9 | N-methyl-L-tryptophan oxidase [EC:1.5.3.-] | | |
| K02849 | 21 | heptosyltransferase III [EC:2.4.-.-] | | |
| K02852 | 8 | UDP-N-acetyl-D-mannosaminuronic acid transferase [EC:2.4.1.-] | | |
| K02854 | 1 | AraC family transcriptional regulator, L-rhamnose operon | | |
| K02855 | 3 | AraC family transcriptional regulator, L-rhamnose operon regulatory | | |
| K02856 | 3 | L-rhamnose-H+ transport protein | | |
| K02858 | 40 | 3,4-dihydroxy 2-butanone 4-phosphate synthase [EC:4.1.99.12] | | |
| K02860 | 41 | 16S rRNA processing protein RimM | | |
| K02862 | 6 | putative membrane protein | | |
| K02863 | 21 | large subunit ribosomal protein L1 | | |
| K02864 | 19 | large subunit ribosomal protein L10 | | |
| K02867 | 17 | large subunit ribosomal protein L11 | | |
| K02871 | 16 | large subunit ribosomal protein L13 | | |
| K02874 | 22 | large subunit ribosomal protein L14 | | |
| K02876 | 12 | large subunit ribosomal protein L15 | | |
| K02878 | 19 | large subunit ribosomal protein L16 | | |
| K02879 | 21 | large subunit ribosomal protein L17 | | |
| K02881 | 11 | large subunit ribosomal protein L18 | | |
| K02884 | 16 | large subunit ribosomal protein L19 | | |
| K02886 | 47 | large subunit ribosomal protein L2 | | |
| K02887 | 6 | large subunit ribosomal protein L20 | | |
| K02888 | 23 | large subunit ribosomal protein L21 | | |
| K02890 | 18 | large subunit ribosomal protein L22 | | |
| K02892 | 34 | large subunit ribosomal protein L23 | | |
| K02895 | 14 | large subunit ribosomal protein L24 | | |
| K02897 | 28 | large subunit ribosomal protein L25 | | |
| K02899 | 20 | large subunit ribosomal protein L27 | | |
| K02902 | 9 | large subunit ribosomal protein L28 | | |
| K02904 | 6 | large subunit ribosomal protein L29 | | |

| | | | | |
|---|---|---|---|---|
| K02906 | 36 | large subunit ribosomal protein L3 | | |
| K02907 | 9 | large subunit ribosomal protein L30 | | |
| K02909 | 22 | large subunit ribosomal protein L31 | | |
| K02911 | 73 | large subunit ribosomal protein L32 | | |
| K02913 | 9 | large subunit ribosomal protein L33 | | |
| K02914 | 15 | large subunit ribosomal protein L34 | | |
| K02916 | 7 | large subunit ribosomal protein L35 | | |
| K02919 | 2 | large subunit ribosomal protein L36 | | |
| K02926 | 30 | large subunit ribosomal protein L4 | | |
| K02931 | 15 | large subunit ribosomal protein L5 | | |
| K02933 | 28 | large subunit ribosomal protein L6 | | |
| K02935 | 6 | large subunit ribosomal protein L7/L12 | | |
| K02939 | 23 | large subunit ribosomal protein L9 | | |
| K02945 | 47 | small subunit ribosomal protein S1 | | |
| K02946 | 441 | small subunit ribosomal protein S10 | | |
| K02948 | 11 | small subunit ribosomal protein S11 | | |
| K02950 | 7 | small subunit ribosomal protein S12 | | |
| K02952 | 15 | small subunit ribosomal protein S13 | | |
| K02954 | 7 | small subunit ribosomal protein S14 | | |
| K02956 | 8 | small subunit ribosomal protein S15 | | |
| K02959 | 23 | small subunit ribosomal protein S16 | | |
| K02961 | 10 | small subunit ribosomal protein S17 | | |
| K02963 | 15 | small subunit ribosomal protein S18 | | |
| K02965 | 4 | small subunit ribosomal protein S19 | | |
| K02967 | 68 | small subunit ribosomal protein S2 | | |
| K02968 | 28 | small subunit ribosomal protein S20 | | |
| K02970 | 4 | small subunit ribosomal protein S21 | | |
| K02982 | 20 | small subunit ribosomal protein S3 | | |
| K02986 | 30 | small subunit ribosomal protein S4 | | |
| K02988 | 13 | small subunit ribosomal protein S5 | | |
| K02990 | 23 | small subunit ribosomal protein S6 | | |
| K02992 | 15 | small subunit ribosomal protein S7 | | |
| K02994 | 23 | small subunit ribosomal protein S8 | | |
| K02996 | 16 | small subunit ribosomal protein S9 | | |
| K03040 | 34 | DNA-directed RNA polymerase subunit alpha [EC:2.7.7.6] | | |
| K03043 | 77 | DNA-directed RNA polymerase subunit beta [EC:2.7.7.6] | | |
| K03046 | 516 | DNA-directed RNA polymerase subunit beta' [EC:2.7.7.6] | | |
| K03060 | 10 | DNA-directed RNA polymerase subunit omega [EC:2.7.7.6] | | |
| K03070 | 74 | preprotein translocase subunit SecA | | |
| K03071 | 7 | preprotein translocase subunit SecB | | |
| K03072 | 476 | preprotein translocase subunit SecD | | |
| K03073 | 2 | preprotein translocase subunit SecE | | |
| K03074 | 41 | preprotein translocase subunit SecF | | |
| K03075 | 3 | preprotein translocase subunit SecG | | |
| K03076 | 36 | preprotein translocase subunit SecY | | |
| K03077 | 3 | L-ribulose-5-phosphate 4-epimerase [EC:5.1.3.4] | | |
| K03080 | 2 | L-ribulose-5-phosphate 4-epimerase [EC:5.1.3.4] | | |
| K03086 | 56 | RNA polymerase primary sigma factor | | |
| K03087 | 22 | RNA polymerase nonessential primary-like sigma factor | | |
| K03088 | 405 | RNA polymerase sigma-70 factor, ECF subfamily | | |

| | | | | |
|---|---|---|---|---|
| K03089 | 23 | RNA polymerase sigma-32 factor | | |
| K03090 | 20 | RNA polymerase sigma-B factor | | |
| K03091 | 8 | RNA polymerase sporulation-specific sigma factor | | |
| K03092 | 47 | RNA polymerase sigma-54 factor | | |
| K03093 | 27 | RNA polymerase sigma factor | | |
| K03098 | 2 | outer membrane lipoprotein Blc | | |
| K03100 | 92 | signal peptidase I [EC:3.4.21.89] | | |
| K03101 | 20 | signal peptidase II [EC:3.4.23.36] | | |
| K03106 | 47 | signal recognition particle subunit SRP54 | | |
| K03110 | 23 | fused signal recognition particle receptor | | |
| K03111 | 34 | single-strand DNA-binding protein | | |
| K03113 | 2 | translation initiation factor SUI1 | | |
| K03116 | 10 | sec-independent protein translocase protein TatA | | |
| K03117 | 4 | sec-independent protein translocase protein TatB | | |
| K03118 | 57 | sec-independent protein translocase protein TatC | | |
| K03119 | 43 | taurine dioxygenase [EC:1.14.11.17] | | |
| K03146 | 1 | thiamine biosynthetic enzyme | | |
| K03147 | 11 | thiamine biosynthesis protein ThiC | | |
| K03148 | 16 | adenylyltransferase ThiF [EC:2.7.7.-] | | |
| K03149 | 27 | thiamine biosynthesis ThiG | | |
| K03150 | 1 | thiamine biosynthesis ThiH | | |
| K03151 | 4 | thiamine biosynthesis protein ThiI | | |
| K03152 | 3 | 4-methyl-5(b-hydroxyethyl)-thiazole monophosphate biosynthesis | | |
| K03153 | 10 | glycine oxidase [EC:1.4.3.19] | | |
| K03154 | 5 | thiamine biosynthesis ThiS | | |
| K03166 | 6 | DNA topoisomerase VI subunit A [EC:5.99.1.3] | | |
| K03167 | 4 | DNA topoisomerase VI subunit B [EC:5.99.1.3] | | |
| K03168 | 100 | DNA topoisomerase I [EC:5.99.1.2] | | |
| K03169 | 32 | DNA topoisomerase III [EC:5.99.1.2] | | |
| K03177 | 42 | tRNA pseudouridine synthase B [EC:5.4.99.12] | | |
| K03179 | 26 | 4-hydroxybenzoate octaprenyltransferase [EC:2.5.1.-] | | |
| K03182 | 88 | 3-octaprenyl-4-hydroxybenzoate carboxy-lyase UbiD [EC:4.1.1.-] | | |
| K03183 | 77 | ubiquinone/menaquinone biosynthesis methyltransferase [EC:2.1.1.-] | | |
| K03184 | 1 | 2-octaprenyl-3-methyl-6-methoxy-1,4-benzoquinol hydroxylase | | |
| K03185 | 16 | 2-octaprenyl-6-methoxyphenol hydroxylase [EC:1.14.13.-] | | |
| K03186 | 20 | 3-octaprenyl-4-hydroxybenzoate carboxy-lyase UbiX [EC:4.1.1.-] | | |
| K03187 | 2 | urease accessory protein | | |
| K03188 | 1 | urease accessory protein | | |
| K03189 | 3 | urease accessory protein | | |
| K03190 | 2 | urease accessory protein | | |
| K03192 | 1 | urease accessory protein | | |
| K03196 | 1 | type IV secretion system protein VirB11 | | |
| K03197 | 2 | type IV secretion system protein VirB2 | | |
| K03198 | 1 | type IV secretion system protein VirB3 | | |
| K03199 | 8 | type IV secretion system protein VirB4 | | |
| K03200 | 1 | type IV secretion system protein VirB5 | | |
| K03201 | 2 | type IV secretion system protein VirB6 | | |
| K03205 | 7 | type IV secretion system protein VirD4 | | |
| K03208 | 1 | colanic acid biosynthesis glycosyl transferase WcaI | | |
| K03210 | 16 | preprotein translocase subunit YajC | | |

| | | | | |
|---|---|---|---|---|
| K03215 | 31 | RNA methyltransferase, TrmA family [EC:2.1.1.-] | | |
| K03216 | 14 | RNA methyltransferase, TrmH family, group 2 [EC:2.1.1.-] | | |
| K03217 | 71 | preprotein translocase subunit YidC | | |
| K03218 | 28 | RNA methyltransferase, TrmH family [EC:2.1.1.-] | | |
| K03219 | 1 | type III secretion protein SctC | | |
| K03221 | 1 | type III secretion protein SctF | | |
| K03222 | 3 | type III secretion protein SctJ | | |
| K03223 | 1 | type III secretion protein SctL | | |
| K03224 | 1 | ATP synthase in type III secretion protein SctN [EC:3.6.3.14] | | |
| K03225 | 2 | type III secretion protein SctQ | | |
| K03227 | 2 | type III secretion protein SctS | | |
| K03228 | 2 | type III secretion protein SctT | | |
| K03229 | 1 | type III secretion protein SctU | | |
| K03230 | 2 | type III secretion protein SctV | | |
| K03265 | 1 | peptide chain release factor eRF subunit 1 | | |
| K03269 | 5 | UDP-2,3-diacylglucosamine hydrolase [EC:3.6.1.-] | | |
| K03270 | 14 | 3-deoxy-D-manno-octulosonate 8-phosphate phosphatase (KDO 8-P | | |
| K03271 | 12 | phosphoheptose isomerase [EC:5.-.-.-] | | |
| K03272 | 33 | D-beta-D-heptose 7-phosphate kinase / D-beta-D-heptose 1-phosphate | | |
| K03273 | 14 | D-glycero-D-manno-heptose 1,7-bisphosphate phosphatase [EC:3.1.3.-] | | |
| K03274 | 9 | ADP-L-glycero-D-manno-heptose 6-epimerase [EC:5.1.3.20] | | |
| K03280 | 1 | UDP-N-acetylglucosamine:(glucosyl)LPS | | |
| K03281 | 27 | chloride channel protein, CIC family | | |
| K03282 | 26 | large conductance mechanosensitive channel | | |
| K03284 | 57 | metal ion transporter, MIT family | | |
| K03285 | 10 | general bacterial porin, GBP family | | |
| K03286 | 45 | OmpA-OmpF porin, OOP family | | |
| K03287 | 84 | outer membrane factor, OMF family | | |
| K03288 | 4 | MFS transporter, MHS family, citrate/tricarballylate:H+ symporter | | |
| K03289 | 2 | MFS transporter, NHS family, nucleoside permease | | |
| K03290 | 3 | MFS transporter, SHS family, sialic acid transporter | | |
| K03292 | 11 | glycoside/pentoside/hexuronide:cation symporter, GPH family | | |
| K03293 | 16 | amino acid transporter, AAT family | | |
| K03294 | 140 | basic amino acid/polyamine antiporter, APA family | | |
| K03295 | 35 | cation efflux system protein, CDF family | | |
| K03296 | 415 | hydrophobic/amphiphilic exporter-1 (mainly G- bacteria), HAE1 family | | |
| K03297 | 2 | small multidrug resistance protein, SMR family | | |
| K03298 | 13 | drug/metabolite transporter, DME family | | |
| K03299 | 35 | gluconate:H+ symporter, GntP family | | |
| K03302 | 3 | anaerobic C4-dicarboxylate transporter, Dcu family | | |
| K03303 | 17 | lactate transporter, LctP family | | |
| K03304 | 4 | tellurite resistance/dicarboxylate transporter, TDT family | | |
| K03305 | 27 | proton-dependent oligopeptide transporter, POT family | | |
| K03306 | 31 | inorganic phosphate transporter, PiT family | | |
| K03307 | 78 | solute:Na+ symporter, SSS family | | |
| K03308 | 31 | neurotransmitter:Na+ symporter, NSS family | | |
| K03309 | 14 | dicarboxylate/amino acid:cation (Na+ or H+) symporter, DAACS family | | |
| K03312 | 1 | glutamate:Na+ symporter, ESS family | | |
| K03313 | 15 | Na+:H+ antiporter, NhaA family | | |
| K03315 | 2 | Na+:H+ antiporter, NhaC family | | |

| | | | | |
|---|---|---|---|---|
| K03316 | 31 | monovalent cation:H+ antiporter, CPA1 family | | |
| K03317 | 19 | concentrative nucleoside transporter, CNT family | | |
| K03319 | 2 | divalent anion:Na+ symporter, DASS family | | |
| K03320 | 62 | ammonium transporter, Amt family | | |
| K03321 | 52 | sulfate permease, SulP family | | |
| K03322 | 54 | manganese transport protein | | |
| K03324 | 10 | phosphate:Na+ symporter, PNaS family | | |
| K03325 | 22 | arsenite transporter, ACR3 family | | |
| K03326 | 1 | C4-dicarboxylate transporter, DcuC family | | |
| K03327 | 27 | multidrug resistance protein, MATE family | | |
| K03328 | 25 | polysaccharide transporter, PST family | | |
| K03331 | 1 | L-xylulose reductase [EC:1.1.1.10] | | |
| K03332 | 2 | fructan beta-fructosidase [EC:3.2.1.80] | | |
| K03333 | 41 | cholesterol oxidase [EC:1.1.3.6] | | |
| K03335 | 20 | inosose dehydratase [EC:4.2.1.44] | | |
| K03336 | 12 | 3D-(3,5/4)-trihydroxycyclohexane-1,2-dione hydrolase [EC:3.7.1.-] | | |
| K03337 | 2 | 5-deoxy-glucuronate isomerase [EC:5.3.1.-] | | |
| K03338 | 5 | 5-dehydro-2-deoxygluconokinase [EC:2.7.1.92] | | |
| K03339 | 1 | 6-phospho-5-dehydro-2-deoxy-D-gluconate aldolase [EC:4.1.2.29] | | |
| K03342 | 7 | para-aminobenzoate synthetase / 4-amino-4-deoxychorismate lyase | | |
| K03343 | 8 | putrescine oxidase [EC:1.4.3.10] | | |
| K03366 | 4 | (R,R)-butanediol dehydrogenase / diacetyl reductase [EC:1.1.1.4 | | |
| K03367 | 32 | D-alanine-poly(phosphoribitol) ligase [EC:6.1.1.13] | | |
| K03379 | 37 | cyclohexanone monooxygenase [EC:1.14.13.22] | | |
| K03380 | 35 | phenol 2-monooxygenase [EC:1.14.13.7] | | |
| K03381 | 13 | catechol 1,2-dioxygenase [EC:1.13.11.1] | | |
| K03382 | 5 | hydroxyatrazine ethylaminohydrolase [EC:3.5.99.3] | | |
| K03384 | 10 | Unclassified; E1.14.12.- | | |
| K03385 | 9 | formate-dependent nitrite reductase, periplasmic cytochrome c552 | | |
| K03386 | 31 | peroxiredoxin (alkyl hydroperoxide reductase subunit C) | | |
| K03387 | 2 | alkyl hydroperoxide reductase subunit F [EC:1.6.4.-] | | |
| K03388 | 65 | heterodisulfide reductase subunit A [EC:1.8.98.1] | | |
| K03389 | 22 | heterodisulfide reductase subunit B [EC:1.8.98.1] | | |
| K03390 | 14 | heterodisulfide reductase subunit C [EC:1.8.98.1] | | |
| K03391 | 42 | pentachlorophenol monooxygenase [EC:1.14.13.50] | | |
| K03392 | 61 | aminocarboxymuconate-semialdehyde decarboxylase [EC:4.1.1.45] | | |
| K03394 | 1 | precorrin-2 C20-methyltransferase / cobalt-factor-2 | | |
| K03396 | 1 | S-(hydroxymethyl)glutathione synthase [EC:4.4.1.22] | | |
| K03399 | 2 | precorrin-6Y C5,15-methyltransferase [EC:2.1.1.132] | | |
| K03402 | 7 | transcriptional regulator of arginine metabolism | | |
| K03403 | 4 | magnesium chelatase subunit H [EC:6.6.1.1] | | |
| K03404 | 10 | magnesium chelatase subunit D [EC:6.6.1.1] | | |
| K03405 | 29 | magnesium chelatase subunit I [EC:6.6.1.1] | | |
| K03406 | 186 | methyl-accepting chemotaxis protein | | |
| K03407 | 82 | two-component system, chemotaxis family, sensor kinase CheA | | |
| K03408 | 21 | purine-binding chemotaxis protein CheW | | |
| K03410 | 4 | chemotaxis protein CheC | | |
| K03411 | 16 | chemotaxis protein CheD [EC:3.5.1.44] | | |
| K03412 | 64 | two-component system, chemotaxis family, response regulator CheB | | |
| K03413 | 117 | two-component system, chemotaxis family, response regulator CheY | | |

| | | | | |
|---|---|---|---|---|
| K03414 | 3 | chemotaxis protein CheZ | | |
| K03416 | 11 | methylmalonyl-CoA carboxyltransferase [EC:2.1.3.1] | | |
| K03417 | 36 | methylisocitrate lyase [EC:4.1.3.30] | | |
| K03423 | 57 | Unclassified; E1.8.-.- | | |
| K03424 | 47 | TatD DNase family protein [EC:3.1.21.-] | | |
| K03426 | 21 | NAD+ diphosphatase [EC:3.6.1.22] | | |
| K03427 | 48 | type I restriction enzyme M protein [EC:2.1.1.72] | | |
| K03429 | 22 | 1,2-diacylglycerol 3-glucosyltransferase [EC:2.4.1.157] | | |
| K03430 | 5 | 2-aminoethylphosphonate-pyruvate transaminase [EC:2.6.1.37] | | |
| K03431 | 33 | phosphoglucosamine mutase [EC:5.4.2.10] | | |
| K03432 | 2 | proteasome alpha subunit [EC:3.4.25.1] | | |
| K03433 | 9 | proteasome beta subunit [EC:3.4.25.1] | | |
| K03434 | 14 | N-acetylglucosaminylphosphatidylinositol deacetylase [EC:3.5.1.89] | | |
| K03435 | 5 | LacI family transcriptional regulator, fructose operon | | |
| K03436 | 5 | DeoR family transcriptional regulator, fructose operon | | |
| K03437 | 25 | RNA methyltransferase, TrmH family | | |
| K03438 | 31 | S-adenosyl-methyltransferase [EC:2.1.1.-] | | |
| K03439 | 7 | tRNA (guanine-N7-)-methyltransferase [EC:2.1.1.33] | | |
| K03442 | 38 | small conductance mechanosensitive channel | | |
| K03445 | 2 | MFS transporter, DHA1 family, purine ribonucleoside efflux pump | | |
| K03446 | 127 | MFS transporter, DHA2 family, multidrug resistance protein B | | |
| K03449 | 8 | MFS transporter, CP family, cyanate transporter | | |
| K03453 | 3 | bile acid:Na+ symporter, BASS family | | |
| K03455 | 84 | monovalent cation:H+ antiporter-2, CPA2 family | | |
| K03457 | 23 | nucleobase:cation symporter-1, NCS1 family | | |
| K03458 | 12 | nucleobase:cation symporter-2, NCS2 family | | |
| K03463 | 5 | Unclassified; E5.5.1.- | | |
| K03464 | 2 | muconolactone D-isomerase [EC:5.3.3.4] | | |
| K03465 | 12 | thymidylate synthase (FAD) [EC:2.1.1.148] | | |
| K03466 | 493 | DNA segregation ATPase FtsK/SpoIIIE, S-DNA-T family | | |
| K03469 | 41 | ribonuclease HI [EC:3.1.26.4] | | |
| K03470 | 44 | ribonuclease HII [EC:3.1.26.4] | | |
| K03471 | 3 | ribonuclease HIII [EC:3.1.26.4] | | |
| K03473 | 1 | erythronate-4-phosphate dehydrogenase [EC:1.1.1.290] | | |
| K03474 | 23 | pyridoxine 5-phosphate synthase [EC:2.6.99.2] | | |
| K03475 | 1 | PTS system, ascorbate-specific IIC component | | |
| K03476 | 18 | L-ascorbate 6-phosphate lactonase [EC:3.1.1.-] | | |
| K03478 | 5 | hypothetical protein | | |
| K03484 | 4 | LacI family transcriptional regulator, sucrose operon repressor | | |
| K03495 | 39 | glucose inhibited division protein A | | |
| K03496 | 82 | chromosome partitioning protein | | |
| K03497 | 69 | chromosome partitioning protein, ParB family | | |
| K03498 | 19 | trk system potassium uptake protein TrkH | | |
| K03499 | 59 | trk system potassium uptake protein TrkA | | |
| K03500 | 54 | ribosomal RNA small subunit methyltransferase B [EC:2.1.1.-] | | |
| K03501 | 26 | glucose inhibited division protein B [EC:2.1.-.-] | | |
| K03502 | 3 | DNA polymerase V | | |
| K03517 | 23 | quinolinate synthase [EC:2.5.1.72] | | |
| K03518 | 27 | carbon-monoxide dehydrogenase small subunit [EC:1.2.99.2] | | |
| K03519 | 52 | carbon-monoxide dehydrogenase medium subunit [EC:1.2.99.2] | | |

| | | | | |
|---|---|---|---|---|
| K03520 | 196 | carbon-monoxide dehydrogenase large subunit [EC:1.2.99.2] | | |
| K03521 | 32 | electron transfer flavoprotein beta subunit | | |
| K03522 | 30 | electron transfer flavoprotein alpha subunit | | |
| K03523 | 5 | putative biotin biosynthesis protein BioY | | |
| K03524 | 31 | BirA family transcriptional regulator, biotin operon repressor / | | |
| K03525 | 31 | type III pantothenate kinase [EC:2.7.1.33] | | |
| K03526 | 24 | (E)-4-hydroxy-3-methylbut-2-enyl-diphosphate synthase [EC:1.17.7.1] | | |
| K03527 | 34 | 4-hydroxy-3-methylbut-2-enyl diphosphate reductase [EC:1.17.1.2] | | |
| K03529 | 61 | chromosome segregation protein | | |
| K03530 | 59 | DNA-binding protein HU-beta | | |
| K03531 | 44 | cell division protein FtsZ | | |
| K03532 | 3 | trimethylamine-N-oxide reductase (cytochrome c) 1, cytochrome c-type | | |
| K03534 | 1 | L-rhamnose mutarotase [EC:5.1.3.-] | | |
| K03535 | 12 | MFS transporter, ACS family, glucarate transporter | | |
| K03536 | 21 | ribonuclease P protein component [EC:3.1.26.5] | | |
| K03540 | 15 | ribonuclease P subunit RPR2 [EC:3.1.26.5] | | |
| K03543 | 22 | multidrug resistance protein A | | |
| K03544 | 21 | ATP-dependent Clp protease ATP-binding subunit ClpX | | |
| K03545 | 53 | trigger factor | | |
| K03546 | 16 | exonuclease SbcC | | |
| K03547 | 5 | exonuclease SbcD | | |
| K03548 | 14 | putative permease | | |
| K03549 | 37 | KUP system potassium uptake protein | | |
| K03550 | 21 | holliday junction DNA helicase RuvA | | |
| K03551 | 35 | holliday junction DNA helicase RuvB | | |
| K03553 | 41 | recombination protein RecA | | |
| K03555 | 79 | DNA mismatch repair protein MutS | | |
| K03556 | 63 | LuxR family transcriptional regulator, maltose regulon positive | | |
| K03557 | 4 | Fis family transcriptional regulator, factor for inversion | | |
| K03558 | 4 | membrane protein required for colicin V production | | |
| K03559 | 16 | biopolymer transport protein ExbD | | |
| K03560 | 7 | biopolymer transport protein TolR | | |
| K03561 | 29 | biopolymer transport protein ExbB | | |
| K03562 | 14 | biopolymer transport protein TolQ | | |
| K03563 | 5 | carbon storage regulator | | |
| K03564 | 47 | peroxiredoxin Q/BCP [EC:1.11.1.15] | | |
| K03565 | 29 | regulatory protein | | |
| K03566 | 22 | LysR family transcriptional regulator, glycine cleavage system | | |
| K03567 | 1 | glycine cleavage system transcriptional repressor | | |
| K03568 | 39 | TldD protein | | |
| K03569 | 45 | rod shape-determining protein MreB and related proteins | | |
| K03570 | 25 | rod shape-determining protein MreC | | |
| K03571 | 3 | rod shape-determining protein MreD | | |
| K03572 | 44 | DNA mismatch repair protein MutL | | |
| K03574 | 44 | 7,8-dihydro-8-oxoguanine triphosphatase [EC:3.6.1.-] | | |
| K03575 | 41 | A/G-specific adenine glycosylase [EC:3.2.2.-] | | |
| K03576 | 19 | LysR family transcriptional regulator, regulator for metE and metH | | |
| K03577 | 16 | TetR/AcrR family transcriptional regulator, acrAB operon repressor | | |
| K03578 | 38 | ATP-dependent helicase HrpA [EC:3.6.1.-] | | |
| K03579 | 15 | ATP-dependent helicase HrpB [EC:3.6.1.-] | | |

| | | | | |
|---|---|---|---|---|
| K03580 | 7 | ATP-dependent helicase HepA [EC:3.6.1.-] | | |
| K03581 | 29 | exodeoxyribonuclease V alpha subunit [EC:3.1.11.5] | | |
| K03582 | 8 | exodeoxyribonuclease V beta subunit [EC:3.1.11.5] | | |
| K03583 | 15 | exodeoxyribonuclease V gamma subunit [EC:3.1.11.5] | | |
| K03584 | 34 | DNA repair protein RecO (recombination protein O) | | |
| K03585 | 50 | membrane fusion protein | | |
| K03586 | 1 | cell division protein FtsL | | |
| K03587 | 74 | cell division protein FtsI (penicillin-binding protein 3) | | |
| K03588 | 46 | cell division protein FtsW | | |
| K03589 | 17 | cell division protein FtsQ | | |
| K03590 | 36 | cell division protein FtsA | | |
| K03592 | 45 | PmbA protein | | |
| K03593 | 48 | ATP-binding protein involved in chromosome partitioning | | |
| K03594 | 20 | bacterioferritin | | |
| K03595 | 464 | GTP-binding protein Era | | |
| K03596 | 50 | GTP-binding protein LepA | | |
| K03597 | 1 | sigma-E factor negative regulatory protein RseA | | |
| K03598 | 9 | sigma-E factor negative regulatory protein RseB | | |
| K03599 | 4 | stringent starvation protein A | | |
| K03600 | 4 | stringent starvation protein B | | |
| K03601 | 45 | exodeoxyribonuclease VII large subunit [EC:3.1.11.6] | | |
| K03602 | 5 | exodeoxyribonuclease VII small subunit [EC:3.1.11.6] | | |
| K03604 | 5 | LacI family transcriptional regulator, purine nucleotide synthesis | | |
| K03605 | 10 | hydrogenase 1 maturation protease [EC:3.4.24.-] | | |
| K03606 | 8 | putative colanic acid biosysnthesis UDP-glucose lipid carrier | | |
| K03608 | 2 | cell division topological specificity factor | | |
| K03609 | 12 | septum site-determining protein MinD | | |
| K03610 | 2 | septum site-determining protein MinC | | |
| K03611 | 2 | disulfide bond formation protein DsbB | | |
| K03612 | 2 | electron transport complex protein RnfG | | |
| K03613 | 26 | electron transport complex protein RnfE | | |
| K03614 | 3 | electron transport complex protein RnfD | | |
| K03615 | 13 | electron transport complex protein RnfC | | |
| K03616 | 8 | electron transport complex protein RnfB | | |
| K03617 | 4 | electron transport complex protein RnfA | | |
| K03618 | 1 | hydrogenase-1 operon protein HyaF | | |
| K03620 | 12 | Ni/Fe-hydrogenase 1 B-type cytochrome subunit | | |
| K03621 | 30 | glycerol-3-phosphate acyltransferase PlsX [EC:2.3.1.15] | | |
| K03624 | 24 | transcription elongation factor GreA | | |
| K03625 | 21 | N utilization substance protein B | | |
| K03628 | 36 | transcription termination factor Rho | | |
| K03629 | 18 | DNA replication and repair protein RecF | | |
| K03630 | 39 | DNA repair protein RadC | | |
| K03631 | 48 | DNA repair protein RecN (Recombination protein N) | | |
| K03634 | 10 | outer membrane lipoprotein carrier protein | | |
| K03635 | 22 | molybdenum cofactor biosynthesis protein E | | |
| K03636 | 10 | molybdenum cofactor biosynthesis protein D | | |
| K03637 | 16 | molybdenum cofactor biosynthesis protein C | | |
| K03638 | 9 | molybdenum cofactor biosynthesis protein B | | |
| K03639 | 47 | molybdenum cofactor biosynthesis protein A | | |

| | | | | |
|---|---|---|---|---|
| K03640 | 41 | peptidoglycan-associated lipoprotein | | |
| K03641 | 109 | TolB protein | | |
| K03642 | 13 | rare lipoprotein A | | |
| K03643 | 6 | LPS-assembly lipoprotein | | |
| K03644 | 32 | lipoic acid synthetase [EC:2.8.1.8] | | |
| K03648 | 29 | uracil-DNA glycosylase [EC:3.2.2.-] | | |
| K03649 | 18 | TDG/mug DNA glycosylase family protein [EC:3.2.2.-] | | |
| K03650 | 36 | tRNA modification GTPase | | |
| K03651 | 6 | Icc protein | | |
| K03652 | 17 | DNA-3-methyladenine glycosylase [EC:3.2.2.21] | | |
| K03654 | 47 | ATP-dependent DNA helicase RecQ [EC:3.6.1.-] | | |
| K03655 | 58 | ATP-dependent DNA helicase RecG [EC:3.6.1.-] | | |
| K03656 | 16 | ATP-dependent DNA helicase Rep [EC:3.6.1.-] | | |
| K03657 | 133 | DNA helicase II / ATP-dependent DNA helicase PcrA [EC:3.6.1.-] | | |
| K03664 | 27 | SsrA-binding protein | | |
| K03665 | 26 | GTP-binding protein HflX | | |
| K03666 | 10 | host factor-I protein | | |
| K03667 | 21 | ATP-dependent HslUV protease ATP-binding subunit HslU | | |
| K03668 | 3 | heat shock protein HslJ | | |
| K03669 | 4 | membrane glycosyltransferase [EC:2.4.1.-] | | |
| K03670 | 4 | periplasmic glucans biosynthesis protein | | |
| K03671 | 33 | thioredoxin 1 | | |
| K03672 | 8 | thioredoxin 2 [EC:1.8.1.8] | | |
| K03673 | 13 | thiol:disulfide interchange protein DsbA | | |
| K03676 | 7 | glutaredoxin 3 | | |
| K03677 | 11 | CysQ protein | | |
| K03684 | 17 | ribonuclease D [EC:3.1.13.5] | | |
| K03685 | 34 | ribonuclease III [EC:3.1.26.3] | | |
| K03686 | 68 | molecular chaperone DnaJ | | |
| K03687 | 21 | molecular chaperone GrpE | | |
| K03688 | 57 | ubiquinone biosynthesis protein | | |
| K03690 | 5 | hypothetical protein | | |
| K03693 | 1 | penicillin-binding protein | | |
| K03694 | 10 | ATP-dependent Clp protease ATP-binding subunit ClpA | | |
| K03695 | 75 | ATP-dependent Clp protease ATP-binding subunit ClpB | | |
| K03696 | 39 | ATP-dependent Clp protease ATP-binding subunit ClpC | | |
| K03697 | 1 | ATP-dependent Clp protease ATP-binding subunit ClpE | | |
| K03698 | 6 | CMP-binding protein | | |
| K03699 | 57 | putative hemolysin | | |
| K03701 | 117 | excinuclease ABC subunit A | | |
| K03702 | 42 | excinuclease ABC subunit B | | |
| K03703 | 61 | excinuclease ABC subunit C | | |
| K03704 | 67 | cold shock protein (beta-ribbon, CspA family) | | |
| K03705 | 36 | heat-inducible transcriptional repressor | | |
| K03707 | 6 | transcriptional activator TenA [EC:3.5.99.2] | | |
| K03709 | 23 | DtxR family transcriptional regulator, Mn-dependent transcriptional | | |
| K03710 | 43 | GntR family transcriptional regulator | | |
| K03711 | 30 | Fur family transcriptional regulator, ferric uptake regulator | | |
| K03712 | 51 | MarR family transcriptional regulator | | |
| K03713 | 4 | MerR family transcriptional regulator, glutamine synthetase | | |

| | | | | |
|---|---|---|---|---|
| K03715 | 6 | 1,2-diacylglycerol 3-beta-galactosyltransferase [EC:2.4.1.46] | | |
| K03717 | 10 | LysR family transcriptional regulator, transcriptional activator of | | |
| K03718 | 11 | Lrp/AsnC family transcriptional regulator, regulator for asnA, asnC | | |
| K03719 | 24 | Lrp/AsnC family transcriptional regulator, leucine-responsive | | |
| K03721 | 11 | transcriptional regulator of aroF, aroG, tyrA and aromatic amino | | |
| K03722 | 55 | ATP-dependent DNA helicase DinG [EC:3.6.1.-] | | |
| K03723 | 74 | transcription-repair coupling factor (superfamily II helicase) | | |
| K03724 | 91 | ATP-dependent helicase Lhr and Lhr-like helicase [EC:3.6.1.-] | | |
| K03726 | 1 | helicase [EC:3.6.1.-] | | |
| K03727 | 17 | ATP-dependent RNA helicase HelY [EC:3.6.1.-] | | |
| K03731 | 2 | trehalose 6-phosphate phosphorylase [EC:2.4.1.216] | | |
| K03732 | 1 | ATP-dependent RNA helicase RhlB [EC:3.6.4.13] | | |
| K03733 | 65 | integrase/recombinase XerC | | |
| K03734 | 31 | thiamine biosynthesis lipoprotein | | |
| K03735 | 4 | ethanolamine ammonia-lyase large subunit [EC:4.3.1.7] | | |
| K03736 | 1 | ethanolamine ammonia-lyase small subunit [EC:4.3.1.7] | | |
| K03737 | 30 | putative pyruvate-flavodoxin oxidoreductase [EC:1.2.7.-] | | |
| K03738 | 68 | aldehyde:ferredoxin oxidoreductase [EC:1.2.7.5] | | |
| K03740 | 1 | D-alanine transfer protein | | |
| K03741 | 27 | arsenate reductase [EC:1.20.4.1] | | |
| K03742 | 58 | competence/damage-inducible protein CinA | | |
| K03743 | 19 | Unclassified; K03743 | | |
| K03744 | 31 | LemA protein | | |
| K03746 | 2 | DNA-binding protein H-NS | | |
| K03747 | 1 | Smg protein | | |
| K03748 | 3 | SanA protein | | |
| K03749 | 2 | DedD protein | | |
| K03750 | 62 | molybdopterin biosynthesis protein MoeA | | |
| K03751 | 39 | molybdopterin biosynthesis protein MoeB | | |
| K03752 | 10 | molybdopterin-guanine dinucleotide biosynthesis protein A | | |
| K03753 | 6 | molybdopterin-guanine dinucleotide biosynthesis protein B | | |
| K03756 | 3 | putrescine:ornithine antiporter | | |
| K03758 | 2 | arginine:ornithine antiporter | | |
| K03759 | 5 | arginine:agmatine antiporter | | |
| K03760 | 1 | phosphoethanolamine transferase | | |
| K03761 | 2 | MFS transporter, MHS family, alpha-ketoglutarate permease | | |
| K03762 | 32 | MFS transporter, MHS family, proline/betaine transporter | | |
| K03763 | 10 | DNA polymerase III subunit alpha, Gram-positive type [EC:2.7.7.7] | | |
| K03765 | 5 | transcriptional activator of cad operon | | |
| K03767 | 15 | peptidyl-prolyl cis-trans isomerase A (cyclophilin A) [EC:5.2.1.8] | | |
| K03768 | 25 | peptidyl-prolyl cis-trans isomerase B (cyclophilin B) [EC:5.2.1.8] | | |
| K03769 | 56 | peptidyl-prolyl cis-trans isomerase C [EC:5.2.1.8] | | |
| K03770 | 44 | peptidyl-prolyl cis-trans isomerase D [EC:5.2.1.8] | | |
| K03771 | 59 | peptidyl-prolyl cis-trans isomerase SurA [EC:5.2.1.8] | | |
| K03772 | 10 | FKBP-type peptidyl-prolyl cis-trans isomerase FkpA [EC:5.2.1.8] | | |
| K03773 | 4 | FKBP-type peptidyl-prolyl cis-trans isomerase FklB [EC:5.2.1.8] | | |
| K03774 | 1 | FKBP-type peptidyl-prolyl cis-trans isomerase SlpA [EC:5.2.1.8] | | |
| K03775 | 9 | FKBP-type peptidyl-prolyl cis-trans isomerase SlyD [EC:5.2.1.8] | | |
| K03776 | 16 | aerotaxis receptor | | |
| K03777 | 7 | D-lactate dehydrogenase [EC:1.1.1.28] | | |

| | | | | |
|---|---|---|---|---|
| K03778 | 7 | D-lactate dehydrogenase [EC:1.1.1.28] | | |
| K03779 | 2 | L(+)-tartrate dehydratase alpha subunit [EC:4.2.1.32] | | |
| K03780 | 3 | L(+)-tartrate dehydratase beta subunit [EC:4.2.1.32] | | |
| K03781 | 12 | catalase [EC:1.11.1.6] | | |
| K03782 | 24 | catalase/peroxidase [EC:1.11.1.6 1.11.1.7] | | |
| K03783 | 27 | purine-nucleoside phosphorylase [EC:2.4.2.1] | | |
| K03784 | 5 | purine-nucleoside phosphorylase [EC:2.4.2.1] | | |
| K03785 | 2 | 3-dehydroquinate dehydratase I [EC:4.2.1.10] | | |
| K03786 | 17 | 3-dehydroquinate dehydratase II [EC:4.2.1.10] | | |
| K03787 | 26 | 5'-nucleotidase [EC:3.1.3.5] | | |
| K03789 | 28 | ribosomal-protein-alanine N-acetyltransferase [EC:2.3.1.128] | | |
| K03790 | 10 | ribosomal-protein-alanine N-acetyltransferase [EC:2.3.1.128] | | |
| K03791 | 7 | putative chitinase | | |
| K03793 | 3 | pteridine reductase [EC:1.5.1.33] | | |
| K03795 | 10 | sirohydrochlorin cobaltochelatase [EC:4.99.1.3] | | |
| K03797 | 79 | carboxyl-terminal processing protease [EC:3.4.21.102] | | |
| K03798 | 73 | cell division protease FtsH [EC:3.4.24.-] | | |
| K03799 | 40 | heat shock protein HtpX [EC:3.4.24.-] | | |
| K03800 | 12 | lipoate-protein ligase A [EC:2.7.7.63] | | |
| K03801 | 27 | lipoyl(octanoyl) transferase [EC:2.3.1.181] | | |
| K03802 | 15 | cyanophycin synthetase [EC:6.-.-.-] | | |
| K03806 | 4 | AmpD protein | | |
| K03808 | 5 | paraquat-inducible protein A | | |
| K03809 | 6 | Trp repressor binding protein | | |
| K03810 | 12 | virulence factor | | |
| K03811 | 3 | nicotinamide mononucleotide transporter | | |
| K03814 | 19 | monofunctional biosynthetic peptidoglycan transglycosylase | | |
| K03815 | 1 | xanthosine phosphorylase [EC:2.4.2.-] | | |
| K03818 | 8 | putative colanic acid biosynthesis acetyltransferase WcaF | | |
| K03819 | 1 | putative colanic acid biosynthesis acetyltransferase WcaB | | |
| K03820 | 42 | apolipoprotein N-acyltransferase [EC:2.3.1.-] | | |
| K03821 | 52 | polyhydroxyalkanoate synthase [EC:2.3.1.-] | | |
| K03822 | 3 | putative long chain acyl-CoA synthase [EC:6.2.1.-] | | |
| K03823 | 23 | phosphinothricin acetyltransferase [EC:2.3.1.183] | | |
| K03824 | 4 | putative acetyltransferase [EC:2.3.1.-] | | |
| K03825 | 4 | putative acetyltransferase [EC:2.3.1.-] | | |
| K03826 | 2 | putative acetyltransferase [EC:2.3.1.-] | | |
| K03827 | 2 | putative acetyltransferase [EC:2.3.1.-] | | |
| K03828 | 9 | putative acetyltransferase [EC:2.3.1.-] | | |
| K03829 | 8 | putative acetyltransferase [EC:2.3.1.-] | | |
| K03831 | 8 | molybdopterin biosynthesis protein Mog | | |
| K03832 | 28 | periplasmic protein TonB | | |
| K03833 | 28 | selenocysteine-specific elongation factor | | |
| K03837 | 1 | serine transporter | | |
| K03839 | 5 | flavodoxin I | | |
| K03841 | 11 | fructose-1,6-bisphosphatase I [EC:3.1.3.11] | | |
| K03851 | 2 | taurine-pyruvate aminotransferase [EC:2.6.1.77] | | |
| K03852 | 6 | sulfoacetaldehyde acetyltransferase [EC:2.3.3.15] | | |
| K03856 | 30 | 3-deoxy-7-phosphoheptulonate synthase [EC:2.5.1.54] | | |
| K03862 | 13 | vanillate monooxygenase [EC:1.14.13.82] | | |

| | | | | |
|---|---|---|---|---|
| K03863 | 2 | vanillate monooxygenase [EC:1.14.13.82] | | |
| K03885 | 77 | NADH dehydrogenase [EC:1.6.99.3] | | |
| K03886 | 3 | menaquinol-cytochrome c reductase iron-sulfur subunit [EC:1.10.2.-] | | |
| K03887 | 9 | menaquinol-cytochrome c reductase cytochrome b subunit [EC:1.10.2.-] | | |
| K03888 | 2 | menaquinol-cytochrome c reductase cytochrome b/c subunit | | |
| K03889 | 6 | ubiquinol-cytochrome c reductase cytochrome c subunit | | |
| K03890 | 3 | ubiquinol-cytochrome c reductase iron-sulfur subunit | | |
| K03891 | 15 | ubiquinol-cytochrome c reductase cytochrome b subunit | | |
| K03892 | 63 | ArsR family transcriptional regulator | | |
| K03893 | 12 | arsenical pump membrane protein | | |
| K03897 | 1 | lysine N6-hydroxylase [EC:1.14.13.59] | | |
| K03918 | 4 | L-lysine 6-transaminase [EC:2.6.1.36] | | |
| K03924 | 61 | MoxR-like ATPase [EC:3.6.3.-] | | |
| K03925 | 28 | MraZ protein | | |
| K03926 | 9 | periplasmic divalent cation tolerance protein | | |
| K03927 | 17 | carboxylesterase type B [EC:3.1.1.1] | | |
| K03928 | 9 | carboxylesterase [EC:3.1.1.1] | | |
| K03930 | 1 | putative tributyrin esterase [EC:3.1.1.-] | | |
| K03931 | 6 | putative isomerase | | |
| K03932 | 12 | polyhydroxybutyrate depolymerase | | |
| K03933 | 3 | chitin-binding protein | | |
| K03943 | 6 | NADH dehydrogenase (ubiquinone) flavoprotein 2 [EC:1.6.5.3 1.6.99.3] | | |
| K03969 | 18 | phage shock protein A | | |
| K03972 | 3 | phage shock protein E | | |
| K03973 | 8 | phage shock protein C | | |
| K03974 | 2 | psp operon transcriptional activator | | |
| K03975 | 32 | membrane-associated protein | | |
| K03977 | 56 | GTP-binding protein | | |
| K03978 | 19 | GTP-binding protein | | |
| K03979 | 43 | GTP-binding protein | | |
| K03980 | 50 | virulence factor | | |
| K03981 | 8 | thiol:disulfide interchange protein DsbC [EC:5.3.4.1] | | |
| K04014 | 24 | formate-dependent nitrite reductase, Fe-S protein | | |
| K04015 | 1 | formate-dependent nitrate reductase complex, transmembrane protein | | |
| K04016 | 4 | formate-dependent nitrite reductase, possible assembly protein | | |
| K04019 | 11 | ethanolamine utilization protein EutA | | |
| K04020 | 4 | phosphotransacetylase | | |
| K04021 | 1 | aldehyde dehydrogenase | | |
| K04027 | 5 | ethanolamine utilization protein EutM | | |
| K04028 | 4 | ethanolamine utilization protein EutN | | |
| K04033 | 1 | AraC family transcriptional regulator, ethanolamine operon | | |
| K04034 | 54 | anaerobic magnesium-protoporphyrin IX monomethyl ester cyclase | | |
| K04035 | 62 | magnesium-protoporphyrin IX monomethyl ester (oxidative) cyclase | | |
| K04041 | 6 | fructose-1,6-bisphosphatase III [EC:3.1.3.11] | | |
| K04042 | 55 | bifunctional protein GlmU [EC:2.7.7.23 2.3.1.157] | | |
| K04043 | 64 | molecular chaperone DnaK | | |
| K04044 | 18 | molecular chaperone HscA | | |
| K04045 | 1 | molecular chaperone HscC | | |
| K04046 | 1 | hypothetical chaperone protein | | |
| K04047 | 11 | starvation-inducible DNA-binding protein | | |

| | | | | |
|---|---|---|---|---|
| K04061 | 2 | flagellar biosynthesis protein | | |
| K04063 | 19 | osmotically inducible protein OsmC | | |
| K04065 | 12 | hyperosmotically inducible periplasmic protein | | |
| K04066 | 67 | primosomal protein N' (replication factor Y) (superfamily II | | |
| K04067 | 9 | primosomal replication protein N'' | | |
| K04069 | 36 | pyruvate formate lyase activating enzyme [EC:1.97.1.4] | | |
| K04070 | 12 | putative pyruvate formate lyase activating enzyme [EC:1.97.1.4] | | |
| K04072 | 15 | acetaldehyde dehydrogenase / alcohol dehydrogenase [EC:1.2.1.10 | | |
| K04073 | 3 | acetaldehyde dehydrogenase [EC:1.2.1.10] | | |
| K04074 | 6 | cell division initiation protein | | |
| K04075 | 46 | tRNA(Ile)-lysidine synthase [EC:6.3.4.-] | | |
| K04076 | 10 | Lon-like ATP-dependent protease [EC:3.4.21.-] | | |
| K04077 | 43 | chaperonin GroEL | | |
| K04078 | 11 | chaperonin GroES | | |
| K04079 | 17 | molecular chaperone HtpG | | |
| K04080 | 2 | molecular chaperone IbpA | | |
| K04082 | 9 | molecular chaperone HscB | | |
| K04083 | 6 | molecular chaperone Hsp33 | | |
| K04084 | 26 | thiol:disulfide interchange protein DsbD [EC:1.8.1.8] | | |
| K04085 | 8 | tRNA 2-thiouridine synthesizing protein A [EC:2.8.1.-] | | |
| K04087 | 11 | membrane protease subunit HflC [EC:3.4.-.-] | | |
| K04088 | 14 | membrane protease subunit HflK [EC:3.4.-.-] | | |
| K04090 | 44 | indolepyruvate ferredoxin oxidoreductase [EC:1.2.7.8] | | |
| K04091 | 50 | alkanesulfonate monooxygenase [EC:1.14.14.5] | | |
| K04094 | 26 | glucose inhibited division protein Gid | | |
| K04095 | 1 | cell filamentation protein | | |
| K04096 | 36 | DNA processing protein | | |
| K04097 | 7 | glutathione S-transferase [EC:2.5.1.18] | | |
| K04098 | 2 | hydroxyquinol 1,2-dioxygenase [EC:1.13.11.37] | | |
| K04103 | 3 | indolepyruvate decarboxylase [EC:4.1.1.74] | | |
| K04107 | 6 | 4-hydroxybenzoyl-CoA reductase subunit 1 [EC:1.3.99.20] | | |
| K04108 | 42 | 4-hydroxybenzoyl-CoA reductase subunit 2 [EC:1.3.99.20] | | |
| K04110 | 22 | benzoate-CoA ligase [EC:6.2.1.25] | | |
| K04112 | 1 | benzoyl-CoA reductase subunit [EC:1.3.99.15] | | |
| K04113 | 10 | benzoyl-CoA reductase subunit [EC:1.3.99.15] | | |
| K04114 | 29 | benzoyl-CoA reductase subunit [EC:1.3.99.15] | | |
| K04115 | 16 | benzoyl-CoA reductase subunit [EC:1.3.99.15] | | |
| K04117 | 32 | cyclohexanecarboxyl-CoA dehydrogenase [EC:1.3.99.-] | | |
| K04118 | 4 | pimeloyl-CoA dehydrogenase [EC:1.3.1.62] | | |
| K04126 | 1 | isopenicillin-N synthase [EC:1.21.3.1] | | |
| K04127 | 15 | isopenicillin-N epimerase [EC:5.1.1.17] | | |
| K04128 | 1 | hydroxymethyl cephem carbamoyltransferase [EC:2.1.3.-] | | |
| K04333 | 1 | LuxR family transcriptional regulator, csgAB operon transcriptional | | |
| K04340 | 1 | scyllo-inosamine-4-phosphate amidinotransferase 1 [EC:2.1.4.2] | | |
| K04477 | 11 | putative hydrolase | | |
| K04485 | 37 | DNA repair protein RadA/Sms | | |
| K04486 | 6 | histidinol-phosphatase (PHP family) [EC:3.1.3.15] | | |
| K04487 | 57 | cysteine desulfurase [EC:2.8.1.7] | | |
| K04488 | 14 | nitrogen fixation protein NifU and related proteins | | |
| K04496 | 9 | C-terminal binding protein | | |

| | | | | |
|---|---|---|---|---|
| K04509 | 20 | formate dehydrogenase (cytochrome) [EC:1.2.2.1] | | |
| K04516 | 4 | chorismate mutase [EC:5.4.99.5] | | |
| K04517 | 16 | prephenate dehydrogenase [EC:1.3.1.12] | | |
| K04518 | 51 | prephenate dehydratase [EC:4.2.1.51] | | |
| K04561 | 21 | nitric-oxide reductase, cytochrome b-containing subunit I | | |
| K04562 | 441 | flagellar biosynthesis protein FlhG | | |
| K04564 | 27 | superoxide dismutase, Fe-Mn family [EC:1.15.1.1] | | |
| K04565 | 6 | Cu/Zn superoxide dismutase [EC:1.15.1.1] | | |
| K04566 | 6 | lysyl-tRNA synthetase, class I [EC:6.1.1.6] | | |
| K04567 | 51 | lysyl-tRNA synthetase, class II [EC:6.1.1.6] | | |
| K04568 | 7 | lysyl-tRNA synthetase, class II [EC:6.1.1.6] | | |
| K04651 | 3 | hydrogenase nickel incorporation protein HypA | | |
| K04652 | 9 | hydrogenase nickel incorporation protein HypB | | |
| K04653 | 11 | hydrogenase expression/formation protein HypC | | |
| K04654 | 11 | hydrogenase expression/formation protein HypD | | |
| K04655 | 20 | hydrogenase expression/formation protein HypE | | |
| K04656 | 44 | hydrogenase maturation protein HypF | | |
| K04691 | 13 | serine protease DegS [EC:3.4.21.-] | | |
| K04719 | 4 | 5,6-dimethylbenzimidazole biosynthesis protein BluB | | |
| K04744 | 27 | LPS-assembly protein | | |
| K04748 | 8 | nitric-oxide reductase NorQ protein [EC:1.7.99.7] | | |
| K04749 | 22 | anti-sigma B factor antagonist | | |
| K04750 | 25 | PhnB protein | | |
| K04751 | 13 | nitrogen regulatory protein P-II 1 | | |
| K04752 | 4 | nitrogen regulatory protein P-II 2 | | |
| K04753 | 14 | suppressor of ftsI | | |
| K04754 | 6 | lipoprotein | | |
| K04755 | 9 | ferredoxin, 2Fe-2S | | |
| K04756 | 11 | alkyl hydroperoxide reductase subunit D | | |
| K04757 | 15 | anti-sigma B factor [EC:2.7.11.1] | | |
| K04758 | 2 | ferrous iron transport protein A | | |
| K04759 | 35 | ferrous iron transport protein B | | |
| K04760 | 10 | transcription elongation factor GreB | | |
| K04761 | 32 | LysR family transcriptional regulator, hydrogen peroxide-inducible | | |
| K04762 | 7 | ribosome-associated heat shock protein Hsp15 | | |
| K04763 | 93 | integrase/recombinase XerD | | |
| K04764 | 11 | integration host factor subunit alpha | | |
| K04766 | 1 | acetoin utilization protein AcuA [EC:2.3.1.-] | | |
| K04767 | 18 | acetoin utilization protein AcuB | | |
| K04768 | 10 | acetoin utilization protein AcuC | | |
| K04769 | 1 | AbrB family transcriptional regulator, stage V sporulation protein T | | |
| K04770 | 25 | Lon-like ATP-dependent protease [EC:3.4.21.-] | | |
| K04771 | 6 | serine protease Do [EC:3.4.21.107] | | |
| K04772 | 20 | serine protease DegQ [EC:3.4.21.-] | | |
| K04773 | 37 | protease IV [EC:3.4.21.-] | | |
| K04774 | 3 | serine protease SohB [EC:3.4.21.-] | | |
| K04780 | 19 | nonribosomal peptide synthetase DhbF | | |
| K04782 | 8 | isochorismate pyruvate-lyase [EC:4.1.3.-] | | |
| K04783 | 2 | yersiniabactin salicyl-AMP ligase [EC:6.3.2.-] | | |
| K04786 | 3 | yersiniabactin nonribosomal peptide/polyketide synthase | | |

| | | | | |
|---|---|---|---|---|
| K04787 | 6 | mycobactin salicyl-AMP ligase [EC:6.3.2.-] | | |
| K04794 | 1 | peptidyl-tRNA hydrolase, PTH2 family [EC:3.1.1.29] | | |
| K05020 | 1 | glycine betaine transporter | | |
| K05275 | 26 | pyridoxine 4-dehydrogenase [EC:1.1.1.65] | | |
| K05281 | 4 | 2'-hydroxyisoflavone reductase [EC:1.3.1.45] | | |
| K05296 | 3 | 3(or 17)beta-hydroxysteroid dehydrogenase [EC:1.1.1.51] | | |
| K05297 | 6 | rubredoxin-NAD+ reductase [EC:1.18.1.1] | | |
| K05299 | 10 | formate dehydrogenase (NADP+) [EC:1.2.1.43] | | |
| K05301 | 55 | sulfite dehydrogenase [EC:1.8.2.1] | | |
| K05306 | 2 | phosphonoacetaldehyde hydrolase [EC:3.11.1.1] | | |
| K05311 | 1 | central glycolytic genes regulator | | |
| K05337 | 22 | ferredoxin | | |
| K05338 | 12 | holin-like protein | | |
| K05339 | 1 | holin-like protein LrgB | | |
| K05340 | 2 | glucose uptake protein | | |
| K05341 | 5 | amylosucrase [EC:2.4.1.4] | | |
| K05343 | 42 | maltose alpha-D-glucosyltransferase [EC:5.4.99.16] | | |
| K05344 | 1 | glucose-1-phosphate phosphodismutase [EC:2.7.1.41] | | |
| K05345 | 21 | putative cyclase [EC:4.6.1.-] | | |
| K05348 | 2 | 2-hydroxycinnamic acid beta-D-glucosylisomelase [EC:5.2.1.-] | | |
| K05349 | 79 | beta-glucosidase [EC:3.2.1.21] | | |
| K05350 | 31 | beta-glucosidase [EC:3.2.1.21] | | |
| K05351 | 2 | D-xylulose reductase [EC:1.1.1.9] | | |
| K05358 | 3 | quinate dehydrogenase (pyrroloquinoline-quinone) [EC:1.1.99.25] | | |
| K05359 | 1 | carboxycyclohexadienyl dehydratase [EC:4.2.1.91] | | |
| K05362 | 1 | UDP-N-acetylmuramoyl-L-alanyl-D-glutamate-L-lysine ligase | | |
| K05363 | 1 | serine/alanine adding enzyme [EC:2.3.2.10] | | |
| K05364 | 58 | peptidoglycan glycosyltransferase [EC:2.4.1.129] | | |
| K05365 | 12 | penicillin-binding protein 1B [EC:2.4.1.129 3.4.-.-] | | |
| K05366 | 129 | penicillin-binding protein 1A [EC:2.4.1.- 3.4.-.-] | | |
| K05367 | 18 | penicillin-binding protein 1C [EC:2.4.1.-] | | |
| K05368 | 1 | aquacobalamin reductase / NAD(P)H-flavin reductase [EC:1.16.1.3 | | |
| K05375 | 2 | MbtH protein | | |
| K05384 | 22 | bilin biosynthesis protein | | |
| K05386 | 8 | bilin biosynthesis protein | | |
| K05394 | 29 | atrazine chlorohydrolase [EC:3.8.1.8] | | |
| K05395 | 5 | cystine reductase [EC:1.8.1.6] | | |
| K05396 | 10 | D-cysteine desulfhydrase [EC:4.4.1.15] | | |
| K05499 | 15 | LacI family transcriptional regulator, repressor for deo operon, | | |
| K05501 | 11 | TetR/AcrR family transcriptional regulator | | |
| K05515 | 85 | penicillin-binding protein 2 | | |
| K05516 | 22 | curved DNA-binding protein | | |
| K05517 | 1 | nucleoside-specific channel-forming protein | | |
| K05520 | 21 | protease I [EC:3.2.-.-] | | |
| K05521 | 16 | ADP-ribosylglycohydrolase [EC:3.2.-.-] | | |
| K05522 | 12 | endonuclease VIII [EC:3.2.2.- 4.2.99.18] | | |
| K05524 | 6 | ferredoxin | | |
| K05525 | 30 | linalool 8-monooxygenase [EC:1.14.99.28] | | |
| K05527 | 6 | BolA protein | | |
| K05539 | 2 | tRNA-dihydrouridine synthase A [EC:1.-.-.-] | | |

| | | | | |
|---|---|---|---|---|
| K05540 | 28 | tRNA-dihydrouridine synthase B [EC:1.-.-.-] | | |
| K05541 | 1 | tRNA-dihydrouridine synthase C [EC:1.-.-.-] | | |
| K05549 | 11 | benzoate 1,2-dioxygenase alpha subunit [EC:1.14.12.10] | | |
| K05550 | 4 | benzoate 1,2-dioxygenase beta subunit [EC:1.14.12.10] | | |
| K05551 | 24 | 3-oxoacyl-ACP synthase I [EC:2.3.1.-] | | |
| K05552 | 22 | 3-oxoacyl-ACP synthase II [EC:2.3.1.-] | | |
| K05555 | 12 | cyclase [EC:4.-.-.-] | | |
| K05556 | 3 | hydroxylacyl-CoA dehydrogenase [EC:1.1.1.-] | | |
| K05557 | 16 | MFS transporter, DHA2 family, integral membrane protein | | |
| K05559 | 4 | multicomponent K+:H+ antiporter subunit A | | |
| K05561 | 2 | multicomponent K+:H+ antiporter subunit D | | |
| K05563 | 1 | multicomponent K+:H+ antiporter subunit F | | |
| K05564 | 1 | multicomponent K+:H+ antiporter subunit G | | |
| K05565 | 4 | multicomponent Na+:H+ antiporter subunit A | | |
| K05567 | 3 | multicomponent Na+:H+ antiporter subunit C | | |
| K05568 | 14 | multicomponent Na+:H+ antiporter subunit D | | |
| K05571 | 1 | multicomponent Na+:H+ antiporter subunit G | | |
| K05572 | 6 | NADH dehydrogenase I subunit 1 [EC:1.6.5.3] | | |
| K05573 | 9 | NADH dehydrogenase I subunit 2 [EC:1.6.5.3] | | |
| K05574 | 6 | NADH dehydrogenase I subunit 3 [EC:1.6.5.3] | | |
| K05575 | 8 | NADH dehydrogenase I subunit 4 [EC:1.6.5.3] | | |
| K05576 | 3 | NADH dehydrogenase I subunit 4L [EC:1.6.5.3] | | |
| K05577 | 7 | NADH dehydrogenase I subunit 5 [EC:1.6.5.3] | | |
| K05578 | 2 | NADH dehydrogenase I subunit 6 [EC:1.6.5.3] | | |
| K05579 | 2 | NADH dehydrogenase I subunit 7 [EC:1.6.5.3] | | |
| K05580 | 4 | NADH dehydrogenase I subunit I [EC:1.6.5.3] | | |
| K05586 | 3 | diaphorase subunit of the bidirectional hydrogenase [EC:1.6.5.3] | | |
| K05587 | 6 | diaphorase subunit of the bidirectional hydrogenase [EC:1.6.5.3] | | |
| K05588 | 3 | diaphorase subunit of the bidirectional hydrogenase [EC:1.6.5.3] | | |
| K05589 | 1 | cell division protein FtsB | | |
| K05590 | 1 | ATP-dependent RNA helicase SrmB [EC:2.7.7.-] | | |
| K05591 | 4 | ATP-independent RNA helicase DbpA [EC:3.6.4.13] | | |
| K05592 | 36 | ATP-dependent RNA helicase DeaD [EC:3.6.4.13] | | |
| K05595 | 33 | multiple antibiotic resistance protein | | |
| K05597 | 1 | glutamin-(asparagin-)ase [EC:3.5.1.38] | | |
| K05599 | 2 | anthranilate 1,2-dioxygenase (deaminating, decarboxylating) large | | |
| K05600 | 2 | anthranilate 1,2-dioxygenase (deaminating, decarboxylating) small | | |
| K05601 | 2 | hydroxylamine reductase [EC:1.7.99.1] | | |
| K05603 | 10 | formimidoylglutamate deiminase [EC:3.5.3.13] | | |
| K05606 | 12 | methylmalonyl-CoA epimerase [EC:5.1.99.1] | | |
| K05685 | 60 | macrolide transport system ATP-binding/permease protein [EC:3.6.3.-] | | |
| K05692 | 2 | actin beta/gamma 1 | | |
| K05708 | 9 | large terminal subunit of phenylpropionate dioxygenase | | |
| K05709 | 3 | small terminal subunit of phenylpropionate dioxygenase | | |
| K05710 | 16 | ferredoxin subunit of phenylpropionate dioxygenase | | |
| K05712 | 33 | 3-(3-hydroxy-phenyl)propionate hydroxylase [EC:1.14.13.-] | | |
| K05714 | 5 | 2-hydroxy-6-ketonona-2,4-dienedioic acid hydrolase [EC:3.7.1.-] | | |
| K05715 | 6 | 2-phosphoglycerate kinase [EC:2.7.2.-] | | |
| K05772 | 8 | putative tungstate transport system substrate-binding protein | | |
| K05773 | 8 | putative tungstate transport system permease protein | | |

| | | | | |
|---|---|---|---|---|
| K05776 | 4 | molybdate transport system ATP-binding protein | | |
| K05777 | 5 | putative thiamine transport system substrate-binding protein | | |
| K05780 | 2 | putative phosphonate transport system ATP-binding protein | | |
| K05781 | 1 | putative phosphonate transport system ATP-binding protein | | |
| K05782 | 6 | benzoate membrane transport protein | | |
| K05783 | 1 | 1,6-dihydroxycyclohexa-2,4-diene-1-carboxylate dehydrogenase | | |
| K05784 | 3 | benzoate 1,2-dioxygenase electron transfer component | | |
| K05785 | 1 | transcriptional antiterminator RfaH | | |
| K05786 | 10 | chloramphenicol-sensitive protein RarD | | |
| K05787 | 1 | DNA-binding protein HU-alpha | | |
| K05788 | 20 | integration host factor subunit beta | | |
| K05794 | 28 | tellurite resistance protein TerC | | |
| K05795 | 6 | tellurium resistance protein TerD | | |
| K05796 | 1 | electron transport protein HydN | | |
| K05797 | 3 | 4-cresol dehydrogenase (hydroxylating) [EC:1.17.99.1] | | |
| K05798 | 12 | LysR family transcriptional regulator, transcriptional activator for | | |
| K05799 | 23 | GntR family transcriptional regulator, transcriptional repressor for | | |
| K05800 | 2 | Lrp/AsnC family transcriptional regulator | | |
| K05802 | 8 | potassium efflux system protein KefA | | |
| K05803 | 4 | lipoprotein NlpI | | |
| K05807 | 19 | putative lipoprotein | | |
| K05808 | 17 | putative sigma-54 modulation protein | | |
| K05810 | 27 | conserved hypothetical protein | | |
| K05812 | 1 | conserved hypothetical protein | | |
| K05813 | 23 | sn-glycerol 3-phosphate transport system substrate-binding protein | | |
| K05814 | 17 | sn-glycerol 3-phosphate transport system permease protein | | |
| K05815 | 10 | sn-glycerol 3-phosphate transport system permease protein | | |
| K05816 | 7 | sn-glycerol 3-phosphate transport system ATP-binding protein | | |
| K05817 | 27 | LysR family transcriptional regulator, hca operon transcriptional | | |
| K05820 | 4 | MFS transporter, PPP family, 3-phenylpropionic acid transporter | | |
| K05823 | 1 | N-acetyldiaminopimelate deacetylase [EC:3.5.1.47] | | |
| K05825 | 2 | 2-aminoadipate transaminase [EC:2.6.1.-] | | |
| K05827 | 5 | lysine biosynthesis protein LysX | | |
| K05829 | 4 | N-acetyl-gamma-aminoadipyl-phosphate reductase [EC:1.2.1.-] | | |
| K05830 | 3 | acetylornithine/acetyl-lysine aminotransferase [EC:2.6.1.11 2.6.1.-] | | |
| K05831 | 2 | acetyl-lysine deacetylase [EC:3.5.1.-] | | |
| K05832 | 1 | putative ABC transport system permease protein | | |
| K05834 | 11 | homoserine/homoserine lactone efflux protein | | |
| K05835 | 1 | threonine efflux protein | | |
| K05836 | 9 | GntR family transcriptional regulator, histidine utilization | | |
| K05837 | 55 | rod shape determining protein RodA | | |
| K05838 | 12 | putative thioredoxin | | |
| K05841 | 12 | sterol 3beta-glucosyltransferase [EC:2.4.1.173] | | |
| K05844 | 10 | ribosomal protein S6 modification protein | | |
| K05845 | 22 | osmoprotectant transport system substrate-binding protein | | |
| K05846 | 27 | osmoprotectant transport system permease protein | | |
| K05847 | 6 | osmoprotectant transport system ATP-binding protein | | |
| K05873 | 1 | adenylate cyclase, class 2 [EC:4.6.1.1] | | |
| K05874 | 21 | methyl-accepting chemotaxis protein I, serine sensor receptor | | |
| K05875 | 8 | methyl-accepting chemotaxis protein II, aspartate sensor receptor | | |

| | | | | |
|---|---|---|---|---|
| K05876 | 2 | methyl-accepting chemotaxis protein III, ribose and galactose sensor | | |
| K05878 | 8 | dihydroxyacetone kinase, N-terminal domain [EC:2.7.1.-] | | |
| K05879 | 5 | dihydroxyacetone kinase, C-terminal domain [EC:2.7.1.-] | | |
| K05881 | 1 | PTS hybrid protein | | |
| K05882 | 45 | aryl-alcohol dehydrogenase (NADP+) [EC:1.1.1.91] | | |
| K05883 | 2 | Unclassified; E1.1.1.210 | | |
| K05885 | 35 | 2,5-diketo-D-gluconate reductase [EC:1.1.1.274] | | |
| K05886 | 5 | serine 3-dehydrogenase [EC:1.1.1.276] | | |
| K05888 | 11 | Unclassified; E1.1.2.- | | |
| K05889 | 3 | polyvinyl-alcohol dehydrogenase (acceptor) [EC:1.1.99.23] | | |
| K05893 | 3 | Unclassified; E1.3.1.35 | | |
| K05895 | 2 | precorrin-6X reductase [EC:1.3.1.54] | | |
| K05896 | 19 | segregation and condensation protein A | | |
| K05897 | 1 | Unclassified; E1.3.3.- | | |
| K05898 | 2 | 3-oxosteroid 1-dehydrogenase [EC:1.3.99.4] | | |
| K05899 | 2 | glycine oxidase [EC:1.4.3.19] | | |
| K05903 | 108 | NADH dehydrogenase (quinone) [EC:1.6.99.5] | | |
| K05905 | 17 | protein-disulfide reductase [EC:1.8.1.8] | | |
| K05909 | 5 | Unclassified; E1.10.3.2 | | |
| K05910 | 1 | NADH peroxidase [EC:1.11.1.1] | | |
| K05911 | 5 | quinone-reactive Ni/Fe-hydrogenase [EC:1.12.5.1] | | |
| K05912 | 20 | Unclassified; E1.12.-.- | | |
| K05914 | 35 | Unclassified; E1.13.12.7 | | |
| K05915 | 9 | Unclassified; E1.13.-.- | | |
| K05916 | 3 | nitric oxide dioxygenase [EC:1.14.12.17] | | |
| K05917 | 1 | cytochrome P450, family 51 (sterol 14-demethylase) [EC:1.14.13.70] | | |
| K05918 | 8 | Unclassified; E1.14.19.- | | |
| K05921 | 16 | 5-oxopent-3-ene-1,2,5-tricarboxylate decarboxylase / | | |
| K05922 | 19 | quinone-reactive Ni/Fe-hydrogenase large subunit [EC:1.12.5.1] | | |
| K05927 | 7 | quinone-reactive Ni/Fe-hydrogenase small subunit [EC:1.12.5.1] | | |
| K05928 | 8 | tocopherol O-methyltransferase [EC:2.1.1.95] | | |
| K05929 | 6 | phosphoethanolamine N-methyltransferase [EC:2.1.1.103] | | |
| K05934 | 6 | precorrin-3B C17-methyltransferase [EC:2.1.1.131] | | |
| K05936 | 4 | precorrin-4 C11-methyltransferase [EC:2.1.1.133] | | |
| K05939 | 12 | acyl-[acyl-carrier-protein]-phospholipid O-acyltransferase / | | |
| K05942 | 1 | Unclassified; E2.3.-.- | | |
| K05944 | 6 | Unclassified; E2.4.1.56 | | |
| K05945 | 1 | Unclassified; E2.4.1.58 | | |
| K05946 | 16 | N-acetylglucosaminyldiphosphoundecaprenol [EC:2.4.1.187] | | |
| K05949 | 5 | Unclassified; E2.4.1.227 | | |
| K05957 | 4 | L-glutamine:scyllo-inosose aminotransferase [EC:2.6.1.50] | | |
| K05961 | 1 | Unclassified; E2.7.1.145 | | |
| K05962 | 30 | protein-histidine pros-kinase [EC:2.7.13.1] | | |
| K05968 | 7 | Unclassified; E3.1.1.6 | | |
| K05969 | 8 | peptidyl-tRNA hydrolase [EC:3.1.1.29] | | |
| K05971 | 43 | Unclassified; E3.1.1.61 | | |
| K05972 | 1 | Unclassified; E3.1.1.72 | | |
| K05973 | 12 | poly(3-hydroxybutyrate) depolymerase [EC:3.1.1.75] | | |
| K05979 | 11 | 2-phosphosulfolactate phosphatase [EC:3.1.3.71] | | |
| K05982 | 8 | deoxyribonuclease V [EC:3.1.21.7] | | |

| | | | | |
|---|---|---|---|---|
| K05984 | 4 | excinuclease Cho [EC:3.1.25.-] | | |
| K05985 | 445 | ribonuclease M5 [EC:3.1.26.8] | | |
| K05993 | 29 | isochorismatase [EC:3.3.2.1] | | |
| K05994 | 2 | bacterial leucyl aminopeptidase [EC:3.4.11.10] | | |
| K05995 | 7 | dipeptidase E [EC:3.4.13.21] | | |
| K05996 | 15 | carboxypeptidase T [EC:3.4.17.18] | | |
| K05997 | 3 | Fe-S cluster assembly protein SufA | | |
| K05998 | 2 | pseudomonalisin [EC:3.4.21.100] | | |
| K05999 | 5 | xanthomonalisin [EC:3.4.21.101] | | |
| K06001 | 31 | tryptophan synthase beta chain [EC:4.2.1.20] | | |
| K06010 | 5 | Unclassified; E3.4.23.43 | | |
| K06013 | 29 | STE24 endopeptidase [EC:3.4.24.84] | | |
| K06015 | 85 | Unclassified; E3.5.1.81 | | |
| K06016 | 27 | N-carbamoyl-L-amino-acid hydrolase [EC:3.5.1.87] | | |
| K06019 | 4 | pyrophosphatase PpaX [EC:3.6.1.1] | | |
| K06020 | 53 | sulfate-transporting ATPase [EC:3.6.3.25] | | |
| K06021 | 11 | phosphate-transporting ATPase [EC:3.6.3.27] | | |
| K06022 | 6 | molybdate-transporting ATPase [EC:3.6.3.29] | | |
| K06023 | 16 | HPr kinase/phosphorylase [EC:2.7.11.- 2.7.4.-] | | |
| K06024 | 17 | segregation and condensation protein B | | |
| K06026 | 35 | Unclassified; E3.6.4.4 | | |
| K06027 | 29 | vesicle-fusing ATPase [EC:3.6.4.6] | | |
| K06031 | 6 | Unclassified; E3.8.1.1 | | |
| K06033 | 3 | Unclassified; E4.1.1.76 | | |
| K06034 | 5 | sulfopyruvate decarboxylase subunit alpha [EC:4.1.1.79] | | |
| K06037 | 1 | Unclassified; E4.2.3.- | | |
| K06039 | 4 | uncharacterized protein involved in oxidation of intracellular | | |
| K06041 | 24 | arabinose-5-phosphate isomerase [EC:5.3.1.13] | | |
| K06042 | 8 | precorrin-8X methylmutase [EC:5.4.1.2] | | |
| K06044 | 33 | (1->4)-alpha-D-glucan 1-alpha-D-glucosylmutase [EC:5.4.99.15] | | |
| K06045 | 23 | squalene-hopene cyclase [EC:5.4.99.17] | | |
| K06048 | 23 | carboxylate-amine ligase [EC:6.3.-.-] | | |
| K06049 | 10 | magnesium chelatase accessory protein | | |
| K06075 | 7 | MarR family transcriptional regulator, transcriptional regulator for | | |
| K06076 | 23 | long-chain fatty acid transport protein | | |
| K06077 | 1 | outer membrane lipoprotein SlyB | | |
| K06113 | 1 | arabinan endo-1,5-alpha-L-arabinosidase [EC:3.2.1.99] | | |
| K06118 | 5 | UDP-sulfoquinovose synthase [EC:3.13.1.1] | | |
| K06120 | 2 | glycerol dehydratase large subunit [EC:4.2.1.30] | | |
| K06121 | 1 | glycerol dehydratase medium subunit [EC:4.2.1.30] | | |
| K06131 | 35 | cardiolipin synthase [EC:2.7.8.-] | | |
| K06132 | 18 | putative cardiolipin synthase [EC:2.7.8.-] | | |
| K06134 | 3 | ubiquinone biosynthesis monooxygenase Coq7 [EC:1.14.13.-] | | |
| K06136 | 9 | pyrroloquinoline quinone biosynthesis protein B | | |
| K06137 | 5 | pyrroloquinoline-quinone synthase [EC:1.3.3.11] | | |
| K06138 | 1 | pyrroloquinoline quinone biosynthesis protein D | | |
| K06139 | 53 | pyrroloquinoline quinone biosynthesis protein E | | |
| K06140 | 4 | regulator of nucleoside diphosphate kinase | | |
| K06142 | 8 | outer membrane protein | | |
| K06143 | 10 | inner membrane protein | | |

| | | | | |
|---|---|---|---|---|
| K06147 | 326 | ATP-binding cassette, subfamily B, bacterial | | |
| K06148 | 122 | ATP-binding cassette, subfamily C, bacterial | | |
| K06149 | 46 | universal stress protein A | | |
| K06151 | 40 | gluconate 2-dehydrogenase alpha chain [EC:1.1.99.3] | | |
| K06153 | 39 | undecaprenyl-diphosphatase [EC:3.6.1.27] | | |
| K06155 | 4 | Gnt-I system high-affinity gluconate transporter | | |
| K06158 | 42 | ATP-binding cassette, sub-family F, member 3 | | |
| K06162 | 1 | PhnM protein | | |
| K06163 | 1 | PhnJ protein | | |
| K06164 | 3 | PhnI protein | | |
| K06167 | 14 | PhnP protein | | |
| K06168 | 67 | bifunctional enzyme involved in thiolation and methylation of tRNA | | |
| K06173 | 30 | tRNA pseudouridine synthase A [EC:5.4.99.12] | | |
| K06175 | 3 | tRNA pseudouridine synthase C [EC:5.4.99.12] | | |
| K06176 | 3 | tRNA pseudouridine synthase D [EC:5.4.99.12] | | |
| K06177 | 17 | ribosomal large subunit pseudouridine synthase A [EC:5.4.99.12] | | |
| K06178 | 41 | ribosomal large subunit pseudouridine synthase B [EC:5.4.99.12] | | |
| K06179 | 25 | ribosomal large subunit pseudouridine synthase C [EC:5.4.99.12] | | |
| K06180 | 45 | ribosomal large subunit pseudouridine synthase D [EC:5.4.99.12] | | |
| K06181 | 5 | ribosomal large subunit pseudouridine synthase E [EC:5.4.99.12] | | |
| K06182 | 2 | ribosomal large subunit pseudouridine synthase F [EC:5.4.99.12] | | |
| K06183 | 14 | ribosomal small subunit pseudouridine synthase A [EC:5.4.99.12] | | |
| K06186 | 4 | small protein A | | |
| K06187 | 21 | recombination protein RecR | | |
| K06188 | 15 | aquaporin Z | | |
| K06189 | 18 | magnesium and cobalt transporter | | |
| K06190 | 8 | intracellular septation protein | | |
| K06192 | 8 | paraquat-inducible protein B | | |
| K06193 | 1 | phosphonoacetate hydrolase [EC:3.11.1.2] | | |
| K06194 | 17 | lipoprotein NlpD | | |
| K06195 | 4 | ApaG protein | | |
| K06196 | 31 | cytochrome c-type biogenesis protein | | |
| K06197 | 3 | cation transport regulator | | |
| K06199 | 16 | CrcB protein | | |
| K06200 | 25 | carbon starvation protein | | |
| K06201 | 11 | copper homeostasis protein | | |
| K06202 | 1 | CyaY protein | | |
| K06204 | 29 | DnaK suppressor protein | | |
| K06205 | 2 | MioC protein | | |
| K06206 | 3 | sugar fermentation stimulation protein A | | |
| K06207 | 33 | GTP-binding protein | | |
| K06208 | 12 | chorismate mutase [EC:5.4.99.5] | | |
| K06211 | 1 | HipB family transcriptional regulator, involved in the regulation of | | |
| K06212 | 14 | formate transporter | | |
| K06213 | 42 | magnesium transporter | | |
| K06215 | 8 | pyridoxine biosynthesis protein [EC:4.-.-.-] | | |
| K06217 | 31 | phosphate starvation-inducible protein PhoH and related proteins | | |
| K06218 | 4 | RelE protein | | |
| K06219 | 4 | S-adenosylmethionine-dependent methyltransferase | | |
| K06221 | 8 | 2,5-diketo-D-gluconate reductase A [EC:1.1.1.274] | | |

| | | | | |
|---|---|---|---|---|
| K06222 | 1 | 2,5-diketo-D-gluconate reductase B [EC:1.1.1.274] | | |
| K06223 | 4 | DNA adenine methylase [EC:2.1.1.72] | | |
| K06281 | 38 | hydrogenase large subunit [EC:1.12.99.6] | | |
| K06282 | 22 | hydrogenase small subunit [EC:1.12.99.6] | | |
| K06284 | 5 | transcriptional pleiotropic regulator of transition state genes | | |
| K06287 | 26 | septum formation protein | | |
| K06294 | 1 | spore germination protein D | | |
| K06306 | 15 | spore germination protein | | |
| K06308 | 1 | spore germination protein | | |
| K06320 | 3 | spore maturation protein CgeB | | |
| K06324 | 41 | spore coat protein A | | |
| K06329 | 1 | spore coat protein F | | |
| K06330 | 1 | spore coat protein H | | |
| K06345 | 3 | spore cortex protein | | |
| K06346 | 9 | spoIIIJ-associated protein | | |
| K06348 | 5 | sporulation inhibitor KapD | | |
| K06350 | 5 | antagonist of KipI | | |
| K06351 | 3 | inhibitor of KinA | | |
| K06370 | 4 | morphogenetic protein associated with SpoVID | | |
| K06373 | 4 | spore maturation protein A | | |
| K06374 | 5 | spore maturation protein B | | |
| K06378 | 14 | stage II sporulation protein AA (anti-sigma F factor antagonist) | | |
| K06379 | 6 | stage II sporulation protein AB (anti-sigma F factor) [EC:2.7.11.1] | | |
| K06381 | 23 | stage II sporulation protein D | | |
| K06384 | 1 | stage II sporulation protein M | | |
| K06390 | 4 | stage III sporulation protein AA | | |
| K06391 | 1 | stage III sporulation protein AB | | |
| K06399 | 2 | stage IV sporulation protein B [EC:3.4.21.116] | | |
| K06400 | 21 | site-specific DNA recombinase | | |
| K06402 | 3 | stage IV sporulation protein FB [EC:3.4.24.-] | | |
| K06405 | 1 | stage V sporulation protein AC | | |
| K06407 | 1 | stage V sporulation protein AE | | |
| K06408 | 4 | stage V sporulation protein AF | | |
| K06412 | 4 | stage V sporulation protein G | | |
| K06413 | 5 | stage V sporulation protein K | | |
| K06415 | 10 | stage V sporulation protein R | | |
| K06419 | 1 | small acid-soluble spore protein B (major beta-type SASP) | | |
| K06422 | 11 | small acid-soluble spore protein E (minor gamma-type SASP) | | |
| K06423 | 1 | small acid-soluble spore protein F (minor alpha/beta-type SASP) | | |
| K06428 | 1 | small acid-soluble spore protein K (minor) | | |
| K06434 | 6 | small acid-soluble spore protein (thioredoxin-like protein) | | |
| K06442 | 20 | putative hemolysin | | |
| K06445 | 16 | acyl-CoA dehydrogenase [EC:1.3.99.-] | | |
| K06446 | 6 | acyl-CoA dehydrogenase [EC:1.3.99.-] | | |
| K06447 | 2 | succinylglutamic semialdehyde dehydrogenase [EC:1.2.1.71] | | |
| K06518 | 4 | holin-like protein | | |
| K06595 | 4 | heam-based aerotactic trancducer | | |
| K06596 | 35 | chemosensory pili system protein ChpA (sensor histidine | | |
| K06600 | 1 | chemosensory pili system protein ChpE | | |
| K06601 | 1 | flagellar protein FlbT | | |

| | | | | |
|---|---|---|---|---|
| K06602 | 1 | flagellar protein FlaF | | |
| K06603 | 1 | flagellar protein FlaG | | |
| K06605 | 1 | myo-inositol catabolism protein IolH | | |
| K06606 | 2 | inosose isomerase [EC:5.3.99.-] | | |
| K06607 | 2 | myo-inositol catabolism protein IolS [EC:1.1.1.-] | | |
| K06608 | 3 | DeoR family transcriptional regulator, myo-inositol catabolism | | |
| K06609 | 1 | MFS transporter, SP family, major inositol transporter | | |
| K06714 | 8 | arginine utilization regulatory protein | | |
| K06718 | 2 | L-2,4-diaminobutyric acid acetyltransferase [EC:2.3.1.178] | | |
| K06726 | 1 | D-ribose pyranase [EC:5.-.-.-] | | |
| K06857 | 10 | putative tungstate transport system ATP-binding protein | | |
| K06858 | 8 | vitamin B12 transport system substrate-binding protein | | |
| K06859 | 1 | glucose-6-phosphate isomerase, archaeal [EC:5.3.1.9] | | |
| K06860 | 19 | Unclassified; K06860 | | |
| K06861 | 16 | lipopolysaccharide export system ATP-binding protein [EC:3.6.3.-] | | |
| K06864 | 13 | Unclassified; K06864 | | |
| K06867 | 34 | Unclassified; K06867 | | |
| K06871 | 16 | Unclassified; K06871 | | |
| K06872 | 14 | Unclassified; K06872 | | |
| K06873 | 3 | Unclassified; K06873 | | |
| K06876 | 2 | Unclassified; K06876 | | |
| K06877 | 43 | Unclassified; K06877 | | |
| K06878 | 6 | Unclassified; K06878 | | |
| K06881 | 34 | Unclassified; K06881 | | |
| K06882 | 5 | Unclassified; K06882 | | |
| K06883 | 14 | Unclassified; K06883 | | |
| K06884 | 1 | Unclassified; K06884 | | |
| K06885 | 9 | Unclassified; K06885 | | |
| K06886 | 13 | hemoglobin | | |
| K06887 | 1 | Unclassified; K06887 | | |
| K06888 | 45 | Unclassified; K06888 | | |
| K06889 | 70 | Unclassified; K06889 | | |
| K06890 | 28 | Unclassified; K06890 | | |
| K06891 | 7 | ATP-dependent Clp protease adaptor protein ClpS | | |
| K06892 | 7 | Unclassified; K06892 | | |
| K06893 | 19 | Unclassified; K06893 | | |
| K06894 | 31 | Unclassified; K06894 | | |
| K06895 | 6 | Unclassified; K06895 | | |
| K06896 | 21 | Unclassified; K06896 | | |
| K06897 | 12 | Unclassified; K06897 | | |
| K06898 | 15 | Unclassified; K06898 | | |
| K06899 | 1 | Unclassified; K06899 | | |
| K06900 | 6 | Unclassified; K06900 | | |
| K06901 | 37 | putative MFS transporter, AGZA family, xanthine/uracil permease | | |
| K06902 | 19 | MFS transporter, UMF1 family | | |
| K06903 | 7 | Unclassified; K06903 | | |
| K06904 | 7 | Unclassified; K06904 | | |
| K06905 | 6 | Unclassified; K06905 | | |
| K06907 | 22 | Unclassified; K06907 | | |
| K06909 | 7 | Unclassified; K06909 | | |

| | | | | |
|---|---|---|---|---|
| K06910 | 25 | Unclassified; K06910 | | |
| K06911 | 105 | Unclassified; K06911 | | |
| K06912 | 17 | alpha-ketoglutarate-dependent 2,4-dichlorophenoxyacetate dioxygenase | | |
| K06915 | 44 | Unclassified; K06915 | | |
| K06916 | 7 | Unclassified; K06916 | | |
| K06917 | 3 | tRNA 2-selenouridine synthase [EC:2.9.1.-] | | |
| K06919 | 3 | Unclassified; K06919 | | |
| K06920 | 21 | queuosine biosynthesis protein QueC | | |
| K06921 | 5 | Unclassified; K06921 | | |
| K06922 | 4 | Unclassified; K06922 | | |
| K06923 | 9 | Unclassified; K06923 | | |
| K06925 | 17 | Unclassified; K06925 | | |
| K06927 | 3 | Unclassified; K06927 | | |
| K06929 | 33 | Unclassified; K06929 | | |
| K06931 | 1 | Unclassified; K06931 | | |
| K06934 | 2 | Unclassified; K06934 | | |
| K06936 | 2 | Unclassified; K06936 | | |
| K06937 | 23 | Unclassified; K06937 | | |
| K06938 | 1 | Unclassified; K06938 | | |
| K06940 | 16 | Unclassified; K06940 | | |
| K06941 | 40 | ribosomal RNA large subunit methyltransferase N [EC:2.1.1.-] | | |
| K06942 | 31 | Unclassified; K06942 | | |
| K06944 | 9 | Unclassified; K06944 | | |
| K06947 | 1 | Unclassified; K06947 | | |
| K06948 | 2 | Unclassified; K06948 | | |
| K06949 | 40 | ribosome biogenesis GTPase [EC:3.6.1.-] | | |
| K06950 | 36 | Unclassified; K06950 | | |
| K06951 | 7 | Unclassified; K06951 | | |
| K06952 | 1 | Unclassified; K06952 | | |
| K06953 | 2 | Unclassified; K06953 | | |
| K06954 | 16 | Unclassified; K06954 | | |
| K06955 | 5 | Unclassified; K06955 | | |
| K06956 | 7 | Unclassified; K06956 | | |
| K06958 | 20 | Unclassified; K06958 | | |
| K06959 | 17 | Unclassified; K06959 | | |
| K06960 | 10 | Unclassified; K06960 | | |
| K06962 | 2 | Unclassified; K06962 | | |
| K06966 | 34 | Unclassified; K06966 | | |
| K06967 | 6 | Unclassified; K06967 | | |
| K06968 | 3 | Unclassified; K06968 | | |
| K06969 | 37 | putative SAM-dependent methyltransferase | | |
| K06971 | 6 | Unclassified; K06971 | | |
| K06972 | 1 | Unclassified; K06972 | | |
| K06973 | 3 | Unclassified; K06973 | | |
| K06974 | 7 | Unclassified; K06974 | | |
| K06975 | 3 | Unclassified; K06975 | | |
| K06976 | 4 | Unclassified; K06976 | | |
| K06977 | 1 | Unclassified; K06977 | | |
| K06978 | 97 | Unclassified; K06978 | | |
| K06979 | 58 | Unclassified; K06979 | | |

| | | | | |
|---|---|---|---|---|
| K06980 | 27 | Unclassified; K06980 | | |
| K06981 | 1 | Unclassified; K06981 | | |
| K06983 | 1 | Unclassified; K06983 | | |
| K06985 | 5 | aspartyl protease family protein | | |
| K06986 | 1 | Unclassified; K06986 | | |
| K06987 | 5 | Unclassified; K06987 | | |
| K06988 | 27 | Unclassified; K06988 | | |
| K06989 | 7 | aspartate dehydrogenase [EC:1.4.1.21] | | |
| K06990 | 27 | Unclassified; K06990 | | |
| K06991 | 3 | Unclassified; K06991 | | |
| K06992 | 1 | Unclassified; K06992 | | |
| K06994 | 80 | putative drug exporter of the RND superfamily | | |
| K06996 | 29 | Unclassified; K06996 | | |
| K06997 | 22 | Unclassified; K06997 | | |
| K06998 | 53 | Unclassified; K06998 | | |
| K06999 | 29 | Unclassified; K06999 | | |
| K07000 | 5 | Unclassified; K07000 | | |
| K07001 | 116 | Unclassified; K07001 | | |
| K07002 | 5 | Unclassified; K07002 | | |
| K07003 | 53 | Unclassified; K07003 | | |
| K07004 | 22 | Unclassified; K07004 | | |
| K07005 | 13 | Unclassified; K07005 | | |
| K07006 | 21 | Unclassified; K07006 | | |
| K07007 | 10 | Unclassified; K07007 | | |
| K07008 | 22 | Unclassified; K07008 | | |
| K07009 | 8 | Unclassified; K07009 | | |
| K07010 | 19 | putative glutamine amidotransferase | | |
| K07011 | 57 | Unclassified; K07011 | | |
| K07012 | 6 | Unclassified; K07012 | | |
| K07014 | 4 | Unclassified; K07014 | | |
| K07015 | 1 | Unclassified; K07015 | | |
| K07017 | 5 | Unclassified; K07017 | | |
| K07018 | 12 | Unclassified; K07018 | | |
| K07019 | 16 | Unclassified; K07019 | | |
| K07020 | 7 | Unclassified; K07020 | | |
| K07021 | 40 | Unclassified; K07021 | | |
| K07024 | 51 | Unclassified; K07024 | | |
| K07025 | 74 | putative hydrolase of the HAD superfamily | | |
| K07027 | 59 | Unclassified; K07027 | | |
| K07028 | 17 | Unclassified; K07028 | | |
| K07029 | 47 | Unclassified; K07029 | | |
| K07030 | 32 | Unclassified; K07030 | | |
| K07031 | 8 | Unclassified; K07031 | | |
| K07032 | 15 | Unclassified; K07032 | | |
| K07033 | 8 | Unclassified; K07033 | | |
| K07034 | 6 | Unclassified; K07034 | | |
| K07037 | 17 | Unclassified; K07037 | | |
| K07038 | 7 | Unclassified; K07038 | | |
| K07039 | 2 | Unclassified; K07039 | | |
| K07040 | 23 | Unclassified; K07040 | | |

| | | | | |
|---|---|---|---|---|
| K07042 | 16 | Unclassified; K07042 | | |
| K07043 | 16 | Unclassified; K07043 | | |
| K07044 | 5 | Unclassified; K07044 | | |
| K07045 | 131 | Unclassified; K07045 | | |
| K07046 | 35 | Unclassified; K07046 | | |
| K07047 | 88 | Unclassified; K07047 | | |
| K07048 | 14 | Unclassified; K07048 | | |
| K07050 | 14 | Unclassified; K07050 | | |
| K07051 | 5 | Unclassified; K07051 | | |
| K07052 | 60 | Unclassified; K07052 | | |
| K07053 | 27 | Unclassified; K07053 | | |
| K07054 | 12 | Unclassified; K07054 | | |
| K07056 | 37 | Unclassified; K07056 | | |
| K07057 | 1 | Unclassified; K07057 | | |
| K07058 | 51 | Unclassified; K07058 | | |
| K07062 | 8 | Unclassified; K07062 | | |
| K07063 | 2 | Unclassified; K07063 | | |
| K07064 | 11 | Unclassified; K07064 | | |
| K07065 | 4 | Unclassified; K07065 | | |
| K07067 | 12 | Unclassified; K07067 | | |
| K07068 | 21 | Unclassified; K07068 | | |
| K07071 | 23 | Unclassified; K07071 | | |
| K07072 | 2 | Unclassified; K07072 | | |
| K07074 | 1 | Unclassified; K07074 | | |
| K07075 | 6 | Unclassified; K07075 | | |
| K07076 | 2 | Unclassified; K07076 | | |
| K07077 | 14 | Unclassified; K07077 | | |
| K07078 | 30 | Unclassified; K07078 | | |
| K07079 | 38 | Unclassified; K07079 | | |
| K07080 | 41 | Unclassified; K07080 | | |
| K07081 | 8 | Unclassified; K07081 | | |
| K07082 | 28 | Unclassified; K07082 | | |
| K07083 | 18 | Unclassified; K07083 | | |
| K07085 | 16 | Unclassified; K07085 | | |
| K07086 | 2 | Unclassified; K07086 | | |
| K07088 | 17 | Unclassified; K07088 | | |
| K07089 | 22 | Unclassified; K07089 | | |
| K07090 | 87 | Unclassified; K07090 | | |
| K07091 | 25 | lipopolysaccharide export system permease protein | | |
| K07093 | 7 | Unclassified; K07093 | | |
| K07094 | 1 | Unclassified; K07094 | | |
| K07095 | 21 | Unclassified; K07095 | | |
| K07096 | 25 | Unclassified; K07096 | | |
| K07098 | 27 | Unclassified; K07098 | | |
| K07100 | 43 | Unclassified; K07100 | | |
| K07101 | 5 | Unclassified; K07101 | | |
| K07102 | 16 | Unclassified; K07102 | | |
| K07104 | 17 | Unclassified; K07104 | | |
| K07106 | 20 | N-acetylmuramic acid 6-phosphate etherase [EC:4.2.-.-] | | |
| K07107 | 51 | Unclassified; K07107 | | |

| | | | | |
|---|---|---|---|---|
| K07110 | 12 | Unclassified; K07110 | | |
| K07112 | 18 | Unclassified; K07112 | | |
| K07113 | 5 | Unclassified; K07113 | | |
| K07114 | 83 | Unclassified; K07114 | | |
| K07115 | 2 | Unclassified; K07115 | | |
| K07116 | 73 | Unclassified; K07116 | | |
| K07117 | 6 | Unclassified; K07117 | | |
| K07118 | 4 | Unclassified; K07118 | | |
| K07119 | 19 | Unclassified; K07119 | | |
| K07120 | 8 | Unclassified; K07120 | | |
| K07121 | 4 | Unclassified; K07121 | | |
| K07122 | 1 | Unclassified; K07122 | | |
| K07124 | 34 | Unclassified; K07124 | | |
| K07125 | 1 | Unclassified; K07125 | | |
| K07126 | 43 | Unclassified; K07126 | | |
| K07127 | 6 | 5-hydroxyisourate hydrolase [EC:3.5.2.17] | | |
| K07130 | 24 | Unclassified; K07130 | | |
| K07131 | 6 | Unclassified; K07131 | | |
| K07133 | 13 | Unclassified; K07133 | | |
| K07137 | 10 | Unclassified; K07137 | | |
| K07138 | 10 | Unclassified; K07138 | | |
| K07139 | 2 | Unclassified; K07139 | | |
| K07140 | 12 | Unclassified; K07140 | | |
| K07141 | 16 | Unclassified; K07141 | | |
| K07145 | 11 | Unclassified; K07145 | | |
| K07146 | 1 | Unclassified; K07146 | | |
| K07147 | 73 | Unclassified; K07147 | | |
| K07148 | 4 | Unclassified; K07148 | | |
| K07149 | 5 | Unclassified; K07149 | | |
| K07150 | 2 | Unclassified; K07150 | | |
| K07152 | 50 | Unclassified; K07152 | | |
| K07153 | 1 | Unclassified; K07153 | | |
| K07154 | 7 | Unclassified; K07154 | | |
| K07155 | 4 | quercetin 2,3-dioxygenase [EC:1.13.11.24] | | |
| K07156 | 4 | Unclassified; pcoC | | |
| K07157 | 17 | Unclassified; K07157 | | |
| K07160 | 7 | Unclassified; K07160 | | |
| K07161 | 34 | Unclassified; K07161 | | |
| K07164 | 13 | Unclassified; K07164 | | |
| K07165 | 1 | transmembrane sensor | | |
| K07168 | 7 | CBS domain-containing membrane protein | | |
| K07169 | 39 | FHA domain-containing protein | | |
| K07170 | 8 | GAF domain-containing protein | | |
| K07171 | 5 | Unclassified; K07171 | | |
| K07172 | 1 | Unclassified; K07172 | | |
| K07173 | 15 | S-ribosylhomocysteine lyase [EC:4.4.1.21] | | |
| K07175 | 20 | PhoH-like ATPase | | |
| K07177 | 6 | PDZ domain-containing protein | | |
| K07178 | 2 | RIO kinase 1 [EC:2.7.11.1] | | |
| K07180 | 31 | serine protein kinase | | |

| | | | | |
|---|---|---|---|---|
| K07181 | 7 | putative signal transduction protein containing EAL and modified | | |
| K07182 | 60 | CBS domain-containing protein | | |
| K07183 | 14 | response regulator NasT | | |
| K07185 | 12 | tryptophan-rich sensory protein | | |
| K07186 | 2 | membrane protein | | |
| K07192 | 11 | flotillin | | |
| K07213 | 6 | Unclassified; K07213 | | |
| K07214 | 12 | enterochelin esterase and related enzymes | | |
| K07216 | 14 | hemerythrin | | |
| K07217 | 5 | Mn-containing catalase | | |
| K07218 | 10 | nitrous oxidase accessory protein | | |
| K07219 | 16 | putative molybdopterin biosynthesis protein | | |
| K07220 | 19 | hypothetical protein | | |
| K07221 | 1 | outer membrane porin | | |
| K07222 | 30 | putative flavoprotein involved in K+ transport | | |
| K07223 | 13 | putative iron-dependent peroxidase | | |
| K07224 | 2 | putative lipoprotein | | |
| K07226 | 3 | hypothetical protein | | |
| K07228 | 2 | TrkA domain protein | | |
| K07229 | 3 | Unclassified; K07229 | | |
| K07232 | 6 | cation transport protein ChaC | | |
| K07233 | 1 | copper resistance protein B | | |
| K07234 | 7 | uncharacterized protein involved in response to NO | | |
| K07236 | 1 | tRNA 2-thiouridine synthesizing protein C | | |
| K07237 | 3 | tRNA 2-thiouridine synthesizing protein B | | |
| K07238 | 18 | zinc transporter, ZIP family | | |
| K07239 | 116 | heavy-metal exporter, HME family | | |
| K07240 | 47 | chromate transporter | | |
| K07241 | 10 | high-affinity nickel-transport protein | | |
| K07243 | 27 | high-affinity iron transporter | | |
| K07245 | 21 | putative copper resistance protein D | | |
| K07246 | 8 | tartrate dehydrogenase/decarboxylase / D-malate dehydrogenase | | |
| K07248 | 4 | lactaldehyde dehydrogenase / glycolaldehyde dehydrogenase | | |
| K07250 | 30 | 4-aminobutyrate aminotransferase / (S)-3-amino-2-methylpropionate | | |
| K07257 | 5 | spore coat polysaccharide biosynthesis protein SpsF | | |
| K07258 | 136 | D-alanyl-D-alanine carboxypeptidase (penicillin-binding protein 5/6) | | |
| K07259 | 26 | D-alanyl-D-alanine carboxypeptidase / | | |
| K07260 | 7 | D-alanyl-D-alanine carboxypeptidase [EC:3.4.16.4] | | |
| K07262 | 11 | D-alanyl-D-alanine endopeptidase (penicillin-binding protein 7) | | |
| K07263 | 230 | zinc protease [EC:3.4.99.-] | | |
| K07264 | 23 | 4-amino-4-deoxy-L-arabinose transferase [EC:2.-.-.-] | | |
| K07265 | 3 | capsular polysaccharide export protein | | |
| K07266 | 2 | capsular polysaccharide export protein | | |
| K07267 | 1 | porin | | |
| K07273 | 5 | lysozyme | | |
| K07274 | 6 | outer membrane protein | | |
| K07276 | 2 | hypothetical protein | | |
| K07277 | 60 | outer membrane protein | | |
| K07278 | 43 | outer membrane protein | | |
| K07279 | 41 | hypothetical protein | | |

| | | | | |
|---|---|---|---|---|
| K07281 | 1 | Unclassified; K07281 | | |
| K07282 | 27 | poly-gamma-glutamate synthesis protein (capsule biosynthesis | | |
| K07283 | 1 | putative salt-induced outer membrane protein | | |
| K07284 | 9 | sortase A | | |
| K07285 | 2 | outer membrane lipoprotein | | |
| K07287 | 7 | lipoprotein-34 | | |
| K07288 | 17 | uncharacterized membrane protein | | |
| K07289 | 17 | AsmA protein | | |
| K07290 | 5 | hypothetical protein | | |
| K07300 | 25 | Ca2+:H+ antiporter | | |
| K07301 | 19 | Unclassified; yrbG | | |
| K07302 | 14 | isoquinoline 1-oxidoreductase, alpha subunit [EC:1.3.99.16] | | |
| K07303 | 95 | isoquinoline 1-oxidoreductase, beta subunit [EC:1.3.99.16] | | |
| K07304 | 33 | peptide-methionine (S)-S-oxide reductase [EC:1.8.4.11] | | |
| K07305 | 15 | peptide-methionine (R)-S-oxide reductase [EC:1.8.4.12] | | |
| K07306 | 10 | anaerobic dimethyl sulfoxide reductase subunit A [EC:1.8.99.-] | | |
| K07307 | 10 | anaerobic dimethyl sulfoxide reductase subunit B (DMSO reductase | | |
| K07308 | 1 | anaerobic dimethyl sulfoxide reductase subunit C (DMSO reductase | | |
| K07309 | 1 | putative dimethyl sulfoxide reductase subunit YnfE [EC:1.8.99.-] | | |
| K07310 | 1 | putative dimethyl sulfoxide reductase subunit YnfF [EC:1.8.99.-] | | |
| K07313 | 3 | serine/threonine protein phosphatase 1 [EC:3.1.3.16] | | |
| K07315 | 66 | sigma-B regulation protein RsbU (phosphoserine phosphatase) | | |
| K07316 | 4 | adenine-specific DNA-methyltransferase [EC:2.1.1.72] | | |
| K07318 | 2 | adenine-specific DNA-methyltransferase [EC:2.1.1.72] | | |
| K07319 | 22 | putative adenine-specific DNA-methyltransferase [EC:2.1.1.72] | | |
| K07320 | 7 | putative adenine-specific DNA-methyltransferase [EC:2.1.1.72] | | |
| K07321 | 9 | CO dehydrogenase maturation factor | | |
| K07322 | 13 | regulator of cell morphogenesis and NO signaling | | |
| K07323 | 15 | putative toluene tolerance protein | | |
| K07326 | 7 | hemolysin activation/secretion protein | | |
| K07334 | 3 | proteic killer suppression protein | | |
| K07335 | 28 | basic membrane protein A and related proteins | | |
| K07336 | 1 | PKHD-type hydroxylase [EC:1.14.11.-] | | |
| K07337 | 3 | hypothetical protein | | |
| K07339 | 2 | hypothetical protein | | |
| K07341 | 6 | death on curing protein | | |
| K07343 | 3 | DNA transformation protein and related proteins | | |
| K07345 | 1 | major type 1 subunit fimbrin (pilin) | | |
| K07346 | 3 | fimbrial chaperone protein | | |
| K07347 | 11 | outer membrane usher protein | | |
| K07357 | 1 | type 1 fimbriae regulatory protein FimB | | |
| K07386 | 54 | putative endopeptidase [EC:3.4.24.-] | | |
| K07387 | 22 | putative metalloprotease [EC:3.4.24.-] | | |
| K07390 | 4 | monothiol glutaredoxin | | |
| K07391 | 65 | magnesium chelatase family protein | | |
| K07392 | 4 | AAA family ATPase | | |
| K07393 | 11 | putative glutathione S-transferase | | |
| K07395 | 4 | putative proteasome-type protease | | |
| K07396 | 1 | putative protein-disulfide isomerase | | |
| K07397 | 21 | putative redox protein | | |

| | | | | |
|---|---|---|---|---|
| K07399 | 14 | cytochrome c biogenesis protein | | |
| K07400 | 2 | Fe/S biogenesis protein NfuA | | |
| K07401 | 1 | selenoprotein W-related protein | | |
| K07402 | 49 | xanthine dehydrogenase accessory factor | | |
| K07403 | 31 | membrane-bound serine protease (ClpP class) | | |
| K07404 | 25 | 6-phosphogluconolactonase [EC:3.1.1.31] | | |
| K07405 | 7 | alpha-amylase [EC:3.2.1.1] | | |
| K07406 | 10 | alpha-galactosidase [EC:3.2.1.22] | | |
| K07407 | 12 | alpha-galactosidase [EC:3.2.1.22] | | |
| K07442 | 6 | tRNA (adenine-N1-)-methyltransferase [EC:2.1.1.36] | | |
| K07443 | 9 | methylated-DNA-protein-cysteine methyltransferase related protein | | |
| K07444 | 21 | putative N6-adenine-specific DNA methylase | | |
| K07445 | 12 | putative DNA methylase | | |
| K07447 | 18 | putative holliday junction resolvase [EC:3.1.-.-] | | |
| K07448 | 1 | restriction system protein | | |
| K07450 | 3 | putative resolvase | | |
| K07452 | 2 | 5-methylcytosine-specific restriction enzyme B [EC:3.1.21.-] | | |
| K07454 | 6 | putative restriction endonuclease | | |
| K07455 | 9 | recombination protein RecT | | |
| K07456 | 34 | DNA mismatch repair protein MutS2 | | |
| K07457 | 3 | endonuclease III related protein | | |
| K07458 | 8 | DNA mismatch endonuclease, patch repair protein [EC:3.1.-.-] | | |
| K07459 | 2 | putative ATP-dependent endonuclease of the OLD family | | |
| K07460 | 17 | putative endonuclease | | |
| K07461 | 8 | putative endonuclease | | |
| K07462 | 59 | single-stranded-DNA-specific exonuclease [EC:3.1.-.-] | | |
| K07464 | 3 | putative RecB family exonuclease | | |
| K07465 | 7 | putative RecB family exonuclease | | |
| K07467 | 1 | phage replication initiation protein | | |
| K07469 | 22 | aldehyde dehydrogenase (FAD-independent) [EC:1.2.99.7] | | |
| K07477 | 1 | translin | | |
| K07478 | 38 | putative ATPase | | |
| K07479 | 2 | putative DNA topoisomerase | | |
| K07480 | 1 | insertion element IS1 protein InsB | | |
| K07481 | 18 | transposase, IS5 family | | |
| K07482 | 19 | transposase, IS30 family | | |
| K07483 | 33 | transposase | | |
| K07484 | 48 | transposase | | |
| K07485 | 29 | transposase | | |
| K07486 | 162 | transposase | | |
| K07487 | 46 | transposase | | |
| K07488 | 4 | transposase | | |
| K07491 | 40 | putative transposase | | |
| K07492 | 38 | putative transposase | | |
| K07493 | 63 | putative transposase | | |
| K07494 | 80 | putative transposase | | |
| K07495 | 29 | putative transposase | | |
| K07496 | 10 | putative transposase | | |
| K07497 | 187 | putative transposase | | |
| K07498 | 4 | putative transposase | | |

| | | | | |
|---|---|---|---|---|
| K07499 | 14 | putative transposase | | |
| K07501 | 8 | hypothetical protein | | |
| K07502 | 7 | hypothetical protein | | |
| K07503 | 8 | hypothetical protein | | |
| K07504 | 1 | hypothetical protein | | |
| K07505 | 1 | hypothetical protein | | |
| K07506 | 18 | AraC family transcriptional regulator | | |
| K07507 | 22 | putative Mg2+ transporter-C (MgtC) family protein | | |
| K07508 | 6 | acetyl-CoA acyltransferase 2 [EC:2.3.1.16] | | |
| K07516 | 43 | 3-hydroxyacyl-CoA dehydrogenase [EC:1.1.1.35] | | |
| K07519 | 9 | phthalate 4,5-dioxygenase [EC:1.14.12.7] | | |
| K07533 | 21 | foldase protein PrsA [EC:5.2.1.8] | | |
| K07534 | 4 | cyclohex-1-ene-1-carboxyl-CoA hydratase [EC:4.2.1.-] | | |
| K07535 | 35 | 2-hydroxycyclohexanecarboxyl-CoA dehydrogenase [EC:1.1.1.-] | | |
| K07537 | 4 | cyclohexa-1,5-dienecarbonyl-CoA hydratase [EC:4.2.1.100] | | |
| K07538 | 3 | 6-hydroxycyclohex-1-ene-1-carboxyl-CoA dehydrogenase [EC:1.1.1.-] | | |
| K07543 | 23 | benzylsuccinate CoA-transferase BbsE subunit [EC:2.8.3.15] | | |
| K07544 | 22 | benzylsuccinate CoA-transferase BbsF subunit [EC:2.8.3.15] | | |
| K07545 | 1 | (R)-benzylsuccinyl-CoA dehydro genase [EC:1.3.99.21] | | |
| K07546 | 8 | E-phenylitaconyl-CoA hydratase [EC:4.2.1.-] | | |
| K07547 | 16 | 2-[hydroxy(phenyl)methyl]-succinyl-CoA dehydrogenase BbsC subunit | | |
| K07548 | 10 | 2-[hydroxy(phenyl)methyl]-succinyl-CoA dehydrogenase BbsD subunit | | |
| K07550 | 12 | benzoylsuccinyl-CoA thiolase BbsB subunit [EC:2.3.1.-] | | |
| K07552 | 14 | MFS transporter, DHA1 family, bicyclomycin/chloramphenicol | | |
| K07559 | 1 | putative RNA 2'-phosphotransferase [EC:2.7.1.-] | | |
| K07560 | 41 | D-tyrosyl-tRNA(Tyr) deacylase [EC:3.1.-.-] | | |
| K07566 | 44 | putative translation factor | | |
| K07567 | 68 | TdcF protein | | |
| K07568 | 35 | S-adenosylmethionine:tRNA ribosyltransferase-isomerase [EC:5.-.-.-] | | |
| K07570 | 6 | general stress protein 13 | | |
| K07574 | 3 | putative RNA-binding protein containing KH domain | | |
| K07576 | 52 | metallo-beta-lactamase family protein | | |
| K07577 | 8 | putative mRNA 3-end processing factor | | |
| K07586 | 1 | hypothetical protein | | |
| K07588 | 27 | LAO/AO transport system kinase [EC:2.7.-.-] | | |
| K07592 | 4 | LysR family transcriptional regulator, tdc operon transcriptional | | |
| K07636 | 65 | two-component system, OmpR family, phosphate regulon sensor | | |
| K07637 | 1 | two-component system, OmpR family, sensor histidine kinase PhoQ | | |
| K07638 | 12 | two-component system, OmpR family, osmolarity sensor histidine | | |
| K07639 | 2 | two-component system, OmpR family, sensor histidine kinase RstB | | |
| K07640 | 6 | two-component system, OmpR family, sensor histidine kinase CpxA | | |
| K07641 | 4 | two-component system, OmpR family, sensor histidine kinase CreC | | |
| K07642 | 11 | two-component system, OmpR family, sensor histidine kinase BaeS | | |
| K07643 | 3 | two-component system, OmpR family, sensor histidine kinase BasS | | |
| K07644 | 26 | two-component system, OmpR family, heavy metal sensor histidine | | |
| K07645 | 33 | two-component system, OmpR family, sensor histidine kinase QseC | | |
| K07646 | 67 | two-component system, OmpR family, sensor histidine kinase KdpD | | |
| K07647 | 4 | two-component system, OmpR family, sensor histidine kinase TorS | | |
| K07648 | 1 | two-component system, OmpR family, aerobic respiration control | | |
| K07649 | 29 | two-component system, OmpR family, sensor histidine kinase TctE | | |

| | | | | |
|---|---|---|---|---|
| K07651 | 11 | two-component system, OmpR family, sensor histidine kinase ResE | | |
| K07652 | 42 | two-component system, OmpR family, sensor histidine kinase VicK | | |
| K07653 | 6 | two-component system, OmpR family, sensor histidine kinase MprB | | |
| K07654 | 5 | two-component system, OmpR family, sensor histidine kinase MtrB | | |
| K07656 | 1 | two-component system, OmpR family, sensor histidine kinase TrcS | | |
| K07657 | 68 | two-component system, OmpR family, phosphate regulon response | | |
| K07658 | 67 | two-component system, OmpR family, alkaline phosphatase synthesis | | |
| K07659 | 12 | two-component system, OmpR family, phosphate regulon response | | |
| K07660 | 1 | two-component system, OmpR family, response regulator PhoP | | |
| K07661 | 1 | two-component system, OmpR family, response regulator RstA | | |
| K07662 | 5 | two-component system, OmpR family, response regulator CpxR | | |
| K07663 | 1 | two-component system, OmpR family, catabolic regulation response | | |
| K07664 | 5 | two-component system, OmpR family, response regulator BaeR | | |
| K07665 | 23 | two-component system, OmpR family, copper resistance phosphate | | |
| K07666 | 3 | two-component system, OmpR family, response regulator QseB | | |
| K07667 | 53 | two-component system, OmpR family, KDP operon response regulator | | |
| K07668 | 44 | two-component system, OmpR family, response regulator VicR | | |
| K07669 | 30 | two-component system, OmpR family, response regulator MprA | | |
| K07670 | 18 | two-component system, OmpR family, response regulator MtrA | | |
| K07671 | 1 | two-component system, OmpR family, response regulator PrrA | | |
| K07672 | 1 | two-component system, OmpR family, response regulator TrcR | | |
| K07673 | 10 | two-component system, NarL family, nitrate/nitrite sensor histidine | | |
| K07674 | 4 | two-component system, NarL family, nitrate/nitrite sensor histidine | | |
| K07675 | 49 | two-component system, NarL family, sensor histidine kinase UhpB | | |
| K07677 | 11 | two-component system, NarL family, capsular synthesis sensor | | |
| K07678 | 26 | two-component system, NarL family, sensor histidine kinase BarA | | |
| K07679 | 4 | two-component system, NarL family, sensor histidine kinase EvgS | | |
| K07680 | 15 | two-component system, NarL family, sensor histidine kinase ComP | | |
| K07681 | 1 | two-component system, NarL family, vancomycin resistance sensor | | |
| K07682 | 15 | two-component system, NarL family, sensor histidine kinase DevS | | |
| K07683 | 4 | two-component system, NarL family, sensor histidine kinase NreB | | |
| K07684 | 73 | two-component system, NarL family, nitrate/nitrite response | | |
| K07685 | 6 | two-component system, NarL family, nitrate/nitrite response | | |
| K07686 | 7 | two-component system, NarL family, uhpT operon response regulator | | |
| K07687 | 9 | two-component system, NarL family, captular synthesis response | | |
| K07689 | 20 | two-component system, NarL family, invasion response regulator UvrY | | |
| K07690 | 5 | two-component system, NarL family, response regulator EvgA | | |
| K07691 | 24 | two-component system, NarL family, competent response regulator ComA | | |
| K07692 | 37 | two-component system, NarL family, response regulator DegU | | |
| K07693 | 3 | two-component system, NarL family, response regulator DesR | | |
| K07694 | 4 | two-component system, NarL family, vancomycin resistance associated | | |
| K07695 | 13 | two-component system, NarL family, response regulator DevR | | |
| K07696 | 4 | two-component system, NarL family, response regulator NreC | | |
| K07697 | 11 | two-component system, sporulation sensor kinase B [EC:2.7.13.3] | | |
| K07699 | 1 | two-component system, response regulator, stage 0 sporulation | | |
| K07700 | 2 | two-component system, CitB family, cit operon sensor histidine | | |
| K07703 | 1 | two-component system, CitB family, response regulator DcuR | | |
| K07704 | 7 | two-component system, LytT family, sensor histidine kinase LytS | | |
| K07705 | 5 | two-component system, LytT family, response regulator LytT | | |
| K07707 | 4 | two-component system, AgrA family, response regulator AgrA | | |

| | | | | |
|---|---|---|---|---|
| K07708 | 54 | two-component system, NtrC family, nitrogen regulation sensor | | |
| K07709 | 54 | two-component system, NtrC family, sensor histidine kinase HydH | | |
| K07710 | 39 | two-component system, NtrC family, sensor histidine kinase AtoS | | |
| K07711 | 10 | two-component system, NtrC family, sensor histidine kinase YfhK | | |
| K07712 | 112 | two-component system, NtrC family, nitrogen regulation response | | |
| K07713 | 64 | two-component system, NtrC family, response regulator HydG | | |
| K07714 | 48 | two-component system, NtrC family, response regulator AtoC | | |
| K07715 | 78 | two-component system, NtrC family, response regulator YfhA | | |
| K07716 | 16 | two-component system, cell cycle sensor histidine kinase PleC | | |
| K07718 | 1 | two-component system, sensor histidine kinase YesM [EC:2.7.13.3] | | |
| K07720 | 7 | two-component system, response regulator YesN | | |
| K07726 | 5 | putative transcriptional regulator | | |
| K07727 | 1 | putative transcriptional regulator | | |
| K07729 | 7 | putative transcriptional regulator | | |
| K07734 | 8 | transcriptional regulator | | |
| K07735 | 24 | putative transcriptional regulator | | |
| K07736 | 12 | CarD family transcriptional regulator | | |
| K07737 | 5 | putative transcriptional regulator | | |
| K07738 | 6 | transcriptional repressor NrdR | | |
| K07739 | 4 | elongator complex protein 3 [EC:2.3.1.48] | | |
| K07740 | 1 | regulator of sigma D | | |
| K07741 | 1 | anti-repressor protein | | |
| K07742 | 7 | hypothetical protein | | |
| K07743 | 2 | hypothetical protein | | |
| K07745 | 1 | hypothetical protein | | |
| K07746 | 2 | hypothetical protein | | |
| K07748 | 2 | sterol-4alpha-carboxylate 3-dehydrogenase (decarboxylating) | | |
| K07749 | 163 | formyl-CoA transferase [EC:2.8.3.16] | | |
| K07758 | 5 | pyridoxal phosphatase [EC:3.1.3.74] | | |
| K07768 | 3 | two-component system, OmpR family, sensor histidine kinase SenX3 | | |
| K07769 | 5 | two-component system, OmpR family, sensor histidine kinase NblS | | |
| K07771 | 1 | two-component system, OmpR family, response regulator BasR | | |
| K07773 | 1 | two-component system, OmpR family, aerobic respiration control | | |
| K07774 | 14 | two-component system, OmpR family, response regulator TctD | | |
| K07775 | 5 | two-component system, OmpR family, response regulator ResD | | |
| K07776 | 43 | two-component system, OmpR family, response regulator RegX3 | | |
| K07777 | 14 | two-component system, NarL family, sensor histidine kinase DegS | | |
| K07778 | 10 | two-component system, NarL family, sensor histidine kinase DesK | | |
| K07782 | 1 | LuxR family transcriptional regulator | | |
| K07783 | 2 | MFS transporter, OPA family, sugar phosphate sensor protein UhpC | | |
| K07785 | 1 | MFS transporter, NRE family, putaive nickel resistance protein | | |
| K07787 | 112 | Cu(I)/Ag(I) efflux system membrane protein CusA | | |
| K07788 | 66 | RND superfamily, multidrug transport protein MdtB | | |
| K07789 | 37 | RND superfamily, multidrug transport protein MdtC | | |
| K07790 | 1 | putative membrane protein PagO | | |
| K07791 | 3 | anaerobic C4-dicarboxylate transporter DcuA | | |
| K07792 | 4 | anaerobic C4-dicarboxylate transporter DcuB | | |
| K07793 | 90 | putative tricarboxylic transport membrane protein | | |
| K07795 | 87 | putative tricarboxylic transport membrane protein | | |
| K07796 | 16 | Cu(I)/Ag(I) efflux system outer membrane protein CusC | | |

| | | | | |
|---|---|---|---|---|
| K07798 | 51 | Cu(I)/Ag(I) efflux system membrane protein CusB | | |
| K07799 | 33 | putative multidrug efflux transporter MdtA | | |
| K07805 | 1 | putative membrane protein PagD | | |
| K07806 | 20 | UDP-4-amino-4-deoxy-L-arabinose-oxoglutarate aminotransferase | | |
| K07811 | 3 | trimethylamine-N-oxide reductase (cytochrome c) 1 [EC:1.7.2.3] | | |
| K07812 | 12 | trimethylamine-N-oxide reductase (cytochrome c) 2 [EC:1.7.2.3] | | |
| K07814 | 158 | putative two-component system response regulator | | |
| K07821 | 1 | trimethylamine-N-oxide reductase (cytochrome c) 2, cytochrome c-type | | |
| K07823 | 4 | 3-oxoadipyl-CoA thiolase [EC:2.3.1.174] | | |
| K07824 | 6 | benzoate 4-monooxygenase [EC:1.14.13.12] | | |
| K07978 | 4 | GntR family transcriptional regulator | | |
| K07979 | 9 | GntR family transcriptional regulator | | |
| K08068 | 1 | N-acetylglucosamine-6-phosphate 2-epimerase and phosphatase | | |
| K08070 | 95 | 2-alkenal reductase [EC:1.3.1.74] | | |
| K08078 | 2 | 3a,7a,12a-trihydroxy-5b-cholest-24-enoyl-CoA hydratase | | |
| K08082 | 4 | two-component system, LytT family, sensor histidine kinase AlgZ | | |
| K08083 | 11 | two-component system, LytT family, response regulator AlgR | | |
| K08084 | 6 | type IV fimbrial biogenesis protein FimT | | |
| K08085 | 2 | type IV fimbrial biogenesis protein FimU | | |
| K08093 | 1 | 3-hexulose-6-phosphate synthase [EC:4.1.2.43] | | |
| K08097 | 6 | phosphosulfolactate synthase [EC:4.4.1.19] | | |
| K08100 | 49 | bilirubin oxidase [EC:1.3.3.5] | | |
| K08137 | 1 | MFS transporter, SP family, galactose:H+ symporter | | |
| K08138 | 2 | MFS transporter, SP family, xylose:H+ symportor | | |
| K08139 | 13 | MFS transporter, SP family, sugar:H+ symporter | | |
| K08151 | 22 | MFS transporter, DHA1 family, tetracycline resistance protein | | |
| K08152 | 3 | MFS transporter, DHA1 family, multidrug resistance protein B | | |
| K08153 | 11 | MFS transporter, DHA1 family, multidrug resistance protein | | |
| K08154 | 1 | MFS transporter, DHA1 family, 2-module integral membrane pump EmrD | | |
| K08156 | 5 | MFS transporter, DHA1 family, arabinose polymer transporter | | |
| K08159 | 2 | MFS transporter, DHA1 family, | | |
| K08161 | 7 | MFS transporter, DHA1 family, multidrug resistance protein | | |
| K08162 | 18 | MFS transporter, DHA1 family, multidrug resistance protein | | |
| K08166 | 40 | MFS transporter, DHA2 family, methylenomycin A resistance protein | | |
| K08167 | 18 | MFS transporter, DHA2 family, methyl viologen resistance protein | | |
| K08169 | 16 | MFS transporter, DHA2 family, multidrug resistance protein | | |
| K08172 | 10 | MFS transporter, MHS family, shikimate and dehydroshikimate | | |
| K08173 | 7 | MFS transporter, MHS family, metabolite:H+ symporter | | |
| K08177 | 46 | MFS transporter, OFA family, oxalate/formate antiporter | | |
| K08178 | 5 | MFS transporter, SHS family, lactate transporter | | |
| K08191 | 15 | MFS transporter, ACS family, hexuronate transporter | | |
| K08194 | 23 | MFS transporter, ACS family, D-galactonate transporter | | |
| K08195 | 3 | MFS transporter, AAHS family, 4-hydroxybenzoate transporter | | |
| K08196 | 5 | MFS transporter, AAHS family, cis,cis-muconate transporter | | |
| K08217 | 15 | MFS transporter, DHA3 family, macrolide efflux protein | | |
| K08218 | 18 | MFS transporter, PAT family, beta-lactamase induction signal | | |
| K08221 | 3 | MFS transporter, ACDE family, multidrug resistance protein | | |
| K08223 | 20 | MFS transporter, FSR family, fosmidomycin resistance protein | | |
| K08224 | 9 | MFS transporter, YNFM family, putative membrane transport protein | | |
| K08225 | 95 | MFS transporter, ENTS family, enterobactin (siderophore) exporter | | |

| | | | | |
|---|---|---|---|---|
| K08226 | 1 | MFS transporter, BCD family, chlorophyll transporter | | |
| K08227 | 12 | MFS transporter, LPLT family, lysophospholipid transporter | | |
| K08234 | 21 | glyoxylase I family protein | | |
| K08252 | 50 | receptor protein-tyrosine kinase [EC:2.7.10.1] | | |
| K08253 | 118 | non-specific protein-tyrosine kinase [EC:2.7.10.2] | | |
| K08256 | 27 | phosphatidylinositol alpha-mannosyltransferase [EC:2.4.1.57] | | |
| K08259 | 50 | lysostaphin [EC:3.4.24.75] | | |
| K08261 | 13 | D-sorbitol dehydrogenase (acceptor) [EC:1.1.99.21] | | |
| K08278 | 4 | thr operon leader peptide | | |
| K08279 | 14 | carnitine operon protein CaiE | | |
| K08281 | 14 | nicotinamidase/pyrazinamidase [EC:3.5.1.19 3.5.1.-] | | |
| K08282 | 355 | non-specific serine/threonine protein kinase [EC:2.7.11.1] | | |
| K08289 | 4 | phosphoribosylglycinamide formyltransferase 2 [EC:2.1.2.2] | | |
| K08295 | 5 | 2-aminobenzoate-CoA ligase [EC:6.2.1.32] | | |
| K08296 | 12 | phosphohistidine phosphatase [EC:3.1.3.-] | | |
| K08297 | 2 | crotonobetainyl-CoA dehydrogenase [EC:1.3.99.-] | | |
| K08299 | 6 | carnitinyl-CoA dehydratase [EC:4.2.1.-] | | |
| K08300 | 19 | ribonuclease E [EC:3.1.26.12] | | |
| K08301 | 25 | ribonuclease G [EC:3.1.26.-] | | |
| K08302 | 1 | tagatose 1,6-diphosphate aldolase [EC:4.1.2.40] | | |
| K08303 | 23 | putative protease [EC:3.4.-.-] | | |
| K08304 | 13 | membrane-bound lytic murein transglycosylase A [EC:3.2.1.-] | | |
| K08305 | 5 | membrane-bound lytic murein transglycosylase B [EC:3.2.1.-] | | |
| K08307 | 30 | membrane-bound lytic murein transglycosylase D [EC:3.2.1.-] | | |
| K08309 | 44 | soluble lytic murein transglycosylase [EC:3.2.1.-] | | |
| K08310 | 5 | dATP pyrophosphohydrolase [EC:3.6.1.-] | | |
| K08311 | 4 | putative (di)nucleoside polyphosphate hydrolase [EC:3.6.1.-] | | |
| K08316 | 6 | ribosomal RNA small subunit methyltransferase D [EC:2.1.1.52] | | |
| K08318 | 2 | putative dehydrogenase [EC:1.1.-.-] | | |
| K08319 | 2 | putative dehydrogenase [EC:1.1.-.-] | | |
| K08320 | 3 | CTP pyrophosphohydrolase [EC:3.6.1.-] | | |
| K08321 | 3 | putative autoinducer-2 (AI-2) aldolase [EC:4.1.2.-] | | |
| K08322 | 8 | starvation sensing protein RspB [EC:1.1.1.-] | | |
| K08323 | 9 | starvation sensing protein RspA | | |
| K08324 | 3 | aldehyde dehydrogenase family protein [EC:1.2.1.-] | | |
| K08326 | 1 | aminopeptidase [EC:3.4.11.-] | | |
| K08344 | 1 | suppressor for copper-sensitivity B | | |
| K08345 | 2 | nitrate reductase 2, alpha subunit [EC:1.7.99.4] | | |
| K08346 | 6 | nitrate reductase 2, beta subunit [EC:1.7.99.4] | | |
| K08348 | 39 | formate dehydrogenase-N, alpha subunit [EC:1.2.1.2] | | |
| K08351 | 5 | biotin sulfoxide reductase [EC:1.-.-.-] | | |
| K08352 | 38 | thiosulfate reductase [EC:1.-.-.-] | | |
| K08353 | 5 | thiosulfate reductase electron transport protein | | |
| K08354 | 3 | thiosulfate reductase cytochrome b subunit | | |
| K08356 | 11 | arsenite oxidase large subunit [EC:1.20.98.1] | | |
| K08357 | 9 | tetrathionate reductase subunit A | | |
| K08358 | 27 | tetrathionate reductase subunit B | | |
| K08359 | 4 | tetrathionate reductase subunit C | | |
| K08364 | 10 | periplasmic mercuric ion binding protein | | |
| K08365 | 3 | MerR family transcriptional regulator, mercuric resistance operon | | |

| | | | | |
|---|---|---|---|---|
| K08368 | 1 | MFS transporter, putative metabolite transport protein | | |
| K08369 | 27 | MFS transporter, putative metabolite:H+ symporter | | |
| K08372 | 45 | putative serine protease PepD [EC:3.4.21.-] | | |
| K08384 | 8 | stage V sporulation protein D (sporulation-specific | | |
| K08477 | 2 | outer membrane protease E [EC:3.4.21.-] | | |
| K08478 | 1 | phosphoglycerate transport regulatory protein PgtC | | |
| K08479 | 3 | two-component system, OmpR family, clock-associated histidine kinase | | |
| K08481 | 4 | circadian clock protein KaiB | | |
| K08482 | 37 | circadian clock protein KaiC | | |
| K08483 | 32 | phosphotransferase system, enzyme I, PtsI [EC:2.7.3.9] | | |
| K08484 | 7 | phosphotransferase system, enzyme I, PtsP [EC:2.7.3.9] | | |
| K08485 | 1 | phosphocarrier protein NPr | | |
| K08567 | 9 | hydrogenase 2 maturation protease [EC:3.4.24.-] | | |
| K08590 | 4 | carbon-nitrogen hydrolase family protein | | |
| K08591 | 25 | glycerol-3-phosphate acyltransferase PlsY [EC:2.3.1.15] | | |
| K08600 | 2 | sortase B | | |
| K08602 | 47 | oligoendopeptidase F [EC:3.4.24.-] | | |
| K08603 | 6 | thermolysin [EC:3.4.24.27] | | |
| K08605 | 1 | coccolysin [EC:3.4.24.30] | | |
| K08641 | 17 | D-alanyl-D-alanine dipeptidase [EC:3.4.13.-] | | |
| K08651 | 23 | thermitase [EC:3.4.21.66] | | |
| K08676 | 43 | tricorn protease [EC:3.4.21.-] | | |
| K08677 | 19 | kumamolisin | | |
| K08678 | 5 | UDP-glucuronate decarboxylase [EC:4.1.1.35] | | |
| K08679 | 5 | UDP-glucuronate 4-epimerase [EC:5.1.3.6] | | |
| K08680 | 8 | 2-succinyl-6-hydroxy-2,4-cyclohexadiene-1-carboxylate synthase | | |
| K08681 | 4 | glutamine amidotransferase [EC:2.6.-.-] | | |
| K08682 | 2 | acyl carrier protein phosphodiesterase [EC:3.1.4.14] | | |
| K08684 | 2 | methane monooxygenase [EC:1.14.13.25] | | |
| K08686 | 15 | 2-chlorobenzoate 1,2-dioxygenase [EC:1.14.12.13] | | |
| K08687 | 4 | N-carbamoylsarcosine amidase [EC:3.5.1.59] | | |
| K08688 | 9 | creatinase [EC:3.5.3.3] | | |
| K08693 | 4 | 3'-nucleotidase [EC:3.1.3.6] | | |
| K08697 | 2 | carbon dioxide concentrating mechanism protein CcmL | | |
| K08698 | 1 | carbon dioxide concentrating mechanism protein CcmM | | |
| K08710 | 5 | N-isopropylammelide isopropylaminohydrolase [EC:3.5.99.4] | | |
| K08714 | 2 | voltage-gated sodium channel | | |
| K08720 | 3 | outer membrane protein OmpU | | |
| K08721 | 3 | multidrug resistance outer membrane protein OprJ | | |
| K08723 | 4 | 5'-nucleotidase [EC:3.1.3.5] | | |
| K08724 | 5 | penicillin-binding protein 2B | | |
| K08738 | 12 | cytochrome c | | |
| K08777 | 1 | neutral peptidase B [EC:3.4.24.-] | | |
| K08884 | 560 | serine/threonine protein kinase, bacterial [EC:2.7.11.1] | | |
| K08900 | 1 | mitochondrial chaperone BCS1 | | |
| K08942 | 8 | photosystem P840 reaction center cytochrome c551 | | |
| K08963 | 23 | methylthioribose-1-phosphate isomerase [EC:5.3.1.23] | | |
| K08964 | 2 | methylthioribulose-1-phosphate dehydratase [EC:4.2.1.109] | | |
| K08965 | 1 | 2,3-diketo-5-methylthiopentyl-1-phosphate enolase [EC:3.1.3.77] | | |
| K08966 | 3 | 2-hydroxy-3-keto-5-methylthiopentenyl-1-phosphate phosphatase | | |

| | | | | |
|---|---|---|---|---|
| K08967 | 4 | 1,2-dihydroxy-3-keto-5-methylthiopentene dioxygenase [EC:1.13.11.53 | | |
| K08969 | 1 | aminotransferase [EC:2.6.1.-] | | |
| K08970 | 5 | nickel/cobalt exporter | | |
| K08972 | 5 | putative membrane protein | | |
| K08973 | 5 | putative membrane protein | | |
| K08974 | 3 | putative membrane protein | | |
| K08977 | 3 | putative membrane protein | | |
| K08978 | 11 | putative membrane protein | | |
| K08981 | 2 | putative membrane protein | | |
| K08983 | 1 | putative membrane protein | | |
| K08984 | 7 | putative membrane protein | | |
| K08986 | 2 | putative membrane protein | | |
| K08987 | 2 | putative membrane protein | | |
| K08988 | 7 | putative membrane protein | | |
| K08989 | 3 | putative membrane protein | | |
| K08990 | 1 | putative membrane protein | | |
| K08992 | 1 | putative membrane protein | | |
| K08995 | 6 | putative membrane protein | | |
| K08997 | 11 | hypothetical protein | | |
| K08998 | 13 | hypothetical protein | | |
| K08999 | 19 | hypothetical protein | | |
| K09001 | 23 | anhydro-N-acetylmuramic acid kinase [EC:2.7.1.-] | | |
| K09003 | 8 | hypothetical protein | | |
| K09004 | 3 | hypothetical protein | | |
| K09005 | 16 | hypothetical protein | | |
| K09007 | 8 | hypothetical protein | | |
| K09008 | 4 | hypothetical protein | | |
| K09009 | 2 | hypothetical protein | | |
| K09011 | 7 | D-citramalate synthase [EC:2.3.1.182] | | |
| K09012 | 6 | DeoR family transcriptional regulator, suf operon transcriptional | | |
| K09013 | 19 | Fe-S cluster assembly ATP-binding protein | | |
| K09014 | 39 | Fe-S cluster assembly protein SufB | | |
| K09015 | 22 | Fe-S cluster assembly protein SufD | | |
| K09016 | 3 | putative pyrimidine permease RutG | | |
| K09017 | 38 | TetR/AcrR family transcriptional regulator | | |
| K09018 | 1 | putative monooxygenase RutA [EC:1.14.-.-] | | |
| K09019 | 4 | putative NADH dehydrogenase/NAD(P)H nitroreductase RutE [EC:1.-.-.-] | | |
| K09020 | 6 | putative isochorismatase family protein RutB [EC:3.-.-.-] | | |
| K09022 | 1 | UPF0076 protein YjgF | | |
| K09023 | 5 | protein RutD | | |
| K09024 | 2 | putative flavin reductase RutF [EC:1.5.1.-] | | |
| K09065 | 3 | N-acetylornithine carbamoyltransferase [EC:2.1.3.9] | | |
| K09116 | 3 | hypothetical protein | | |
| K09117 | 10 | hypothetical protein | | |
| K09118 | 29 | hypothetical protein | | |
| K09120 | 2 | hypothetical protein | | |
| K09121 | 25 | hypothetical protein | | |
| K09122 | 9 | hypothetical protein | | |
| K09123 | 12 | hypothetical protein | | |
| K09125 | 6 | hypothetical protein | | |

| | | | | |
|---|---|---|---|---|
| K09128 | 3 | hypothetical protein | | |
| K09129 | 5 | hypothetical protein | | |
| K09131 | 12 | hypothetical protein | | |
| K09132 | 1 | hypothetical protein | | |
| K09134 | 98 | hypothetical protein | | |
| K09136 | 1 | hypothetical protein | | |
| K09137 | 13 | hypothetical protein | | |
| K09141 | 10 | hypothetical protein | | |
| K09143 | 3 | hypothetical protein | | |
| K09144 | 1 | hypothetical protein | | |
| K09146 | 2 | hypothetical protein | | |
| K09151 | 9 | hypothetical protein | | |
| K09153 | 4 | hypothetical protein | | |
| K09155 | 3 | hypothetical protein | | |
| K09157 | 3 | hypothetical protein | | |
| K09158 | 2 | hypothetical protein | | |
| K09159 | 2 | hypothetical protein | | |
| K09160 | 3 | hypothetical protein | | |
| K09162 | 15 | hypothetical protein | | |
| K09163 | 2 | hypothetical protein | | |
| K09164 | 7 | hypothetical protein | | |
| K09165 | 6 | hypothetical protein | | |
| K09166 | 8 | hypothetical protein | | |
| K09167 | 1 | hypothetical protein | | |
| K09181 | 129 | hypothetical protein | | |
| K09190 | 1 | hypothetical protein | | |
| K09251 | 10 | putrescine aminotransferase [EC:2.6.1.82] | | |
| K09252 | 3 | feruloyl esterase [EC:3.1.1.73] | | |
| K09384 | 1 | hypothetical protein | | |
| K09386 | 19 | hypothetical protein | | |
| K09456 | 23 | putative acyl-CoA dehydrogenase | | |
| K09457 | 12 | 7-cyano-7-deazaguanine reductase [EC:1.7.1.13] | | |
| K09458 | 82 | 3-oxoacyl-[acyl-carrier-protein] synthase II [EC:2.3.1.179] | | |
| K09459 | 5 | phosphonopyruvate decarboxylase [EC:4.1.1.82] | | |
| K09461 | 29 | anthraniloyl-CoA monooxygenase [EC:1.14.13.40] | | |
| K09471 | 25 | gamma-glutamylputrescine oxidase [EC:1.4.3.-] | | |
| K09472 | 7 | gamma-glutamyl-gamma-aminobutyraldehyde dehydrogenase [EC:1.2.1.-] | | |
| K09474 | 2 | acid phosphatase (class A) [EC:3.1.3.2] | | |
| K09477 | 5 | citrate:succinate antiporter | | |
| K09516 | 1 | all-trans-retinol 13,14-reductase [EC:1.3.99.23] | | |
| K09607 | 31 | immune inhibitor A [EC:3.4.24.-] | | |
| K09680 | 3 | type II pantothenate kinase [EC:2.7.1.33] | | |
| K09681 | 11 | LysR family transcriptional regulator, transcription activator of | | |
| K09684 | 10 | purine catabolism regulatory protein | | |
| K09686 | 142 | antibiotic transport system permease protein | | |
| K09687 | 115 | antibiotic transport system ATP-binding protein | | |
| K09688 | 3 | capsular polysaccharide transport system permease protein | | |
| K09689 | 1 | capsular polysaccharide transport system ATP-binding protein | | |
| K09690 | 46 | lipopolysaccharide transport system permease protein | | |
| K09691 | 27 | lipopolysaccharide transport system ATP-binding protein | | |

| | | | | |
|---|---|---|---|---|
| K09693 | 8 | teichoic acid transport system ATP-binding protein [EC:3.6.3.40] | | |
| K09694 | 20 | lipooligosaccharide transport system permease protein | | |
| K09695 | 6 | lipooligosaccharide transport system ATP-binding protein | | |
| K09697 | 5 | sodium transport system ATP-binding protein | | |
| K09698 | 20 | nondiscriminating glutamyl-tRNA synthetase [EC:6.1.1.24] | | |
| K09699 | 12 | 2-oxoisovalerate dehydrogenase E2 component (dihydrolipoyl | | |
| K09700 | 2 | hypothetical protein | | |
| K09701 | 10 | hypothetical protein | | |
| K09702 | 2 | hypothetical protein | | |
| K09703 | 2 | hypothetical protein | | |
| K09704 | 1 | hypothetical protein | | |
| K09707 | 3 | hypothetical protein | | |
| K09709 | 9 | hypothetical protein | | |
| K09710 | 20 | hypothetical protein | | |
| K09711 | 3 | hypothetical protein | | |
| K09726 | 3 | hypothetical protein | | |
| K09729 | 2 | hypothetical protein | | |
| K09740 | 1 | hypothetical protein | | |
| K09747 | 16 | hypothetical protein | | |
| K09748 | 32 | hypothetical protein | | |
| K09749 | 6 | hypothetical protein | | |
| K09758 | 10 | aspartate 4-decarboxylase [EC:4.1.1.12] | | |
| K09759 | 2 | nondiscriminating aspartyl-tRNA synthetase [EC:6.1.1.23] | | |
| K09760 | 28 | DNA recombination protein RmuC | | |
| K09761 | 46 | ribosomal RNA small subunit methyltransferase E [EC:2.1.1.-] | | |
| K09762 | 5 | hypothetical protein | | |
| K09763 | 4 | hypothetical protein | | |
| K09764 | 6 | hypothetical protein | | |
| K09765 | 1 | hypothetical protein | | |
| K09766 | 3 | hypothetical protein | | |
| K09767 | 15 | hypothetical protein | | |
| K09768 | 8 | hypothetical protein | | |
| K09769 | 27 | hypothetical protein | | |
| K09770 | 9 | hypothetical protein | | |
| K09772 | 3 | cell division inhibitor SepF | | |
| K09773 | 10 | hypothetical protein | | |
| K09774 | 441 | lipopolysaccharide export system protein LptA | | |
| K09775 | 19 | hypothetical protein | | |
| K09776 | 1 | hypothetical protein | | |
| K09777 | 2 | hypothetical protein | | |
| K09778 | 5 | hypothetical protein | | |
| K09779 | 5 | hypothetical protein | | |
| K09780 | 2 | hypothetical protein | | |
| K09781 | 3 | hypothetical protein | | |
| K09786 | 18 | hypothetical protein | | |
| K09787 | 1 | hypothetical protein | | |
| K09788 | 41 | hypothetical protein | | |
| K09790 | 2 | hypothetical protein | | |
| K09791 | 3 | hypothetical protein | | |
| K09792 | 7 | hypothetical protein | | |

| | | | | |
|---|---|---|---|---|
| K09793 | 1 | hypothetical protein | | |
| K09795 | 3 | hypothetical protein | | |
| K09796 | 6 | hypothetical protein | | |
| K09798 | 3 | hypothetical protein | | |
| K09799 | 2 | hypothetical protein | | |
| K09800 | 10 | hypothetical protein | | |
| K09801 | 2 | hypothetical protein | | |
| K09802 | 1 | hypothetical protein | | |
| K09803 | 7 | hypothetical protein | | |
| K09806 | 1 | hypothetical protein | | |
| K09807 | 9 | hypothetical protein | | |
| K09808 | 44 | lipoprotein-releasing system permease protein | | |
| K09809 | 13 | CDP-glycerol glycerophosphotransferase [EC:2.7.8.12] | | |
| K09810 | 59 | lipoprotein-releasing system ATP-binding protein [EC:3.6.3.-] | | |
| K09811 | 32 | cell division transport system permease protein | | |
| K09812 | 13 | cell division transport system ATP-binding protein | | |
| K09815 | 10 | zinc transport system substrate-binding protein | | |
| K09816 | 9 | zinc transport system permease protein | | |
| K09817 | 8 | zinc transport system ATP-binding protein [EC:3.6.3.-] | | |
| K09818 | 5 | manganese/iron transport system substrate-binding protein | | |
| K09819 | 8 | manganese/iron transport system permease protein | | |
| K09820 | 12 | manganese/iron transport system ATP-binding protein | | |
| K09822 | 6 | hypothetical protein | | |
| K09825 | 6 | Fur family transcriptional regulator, peroxide stress response | | |
| K09826 | 4 | Fur family transcriptional regulator, iron response regulator | | |
| K09833 | 1 | homogenitisate phytyltransferase | | |
| K09835 | 2 | carotenoid isomerase [EC:5.-.-.-] | | |
| K09845 | 3 | methoxyneurosporene dehydrogenase [EC:1.14.99.-] | | |
| K09846 | 18 | hydroxyneurosporene methyltransferase [EC:2.1.1.-] | | |
| K09858 | 1 | SEC-C motif domain protein | | |
| K09859 | 1 | hypothetical protein | | |
| K09862 | 6 | hypothetical protein | | |
| K09879 | 1 | isorenieratene synthase | | |
| K09882 | 3 | cobaltochelatase CobS [EC:6.6.1.2] | | |
| K09883 | 6 | cobaltochelatase CobT [EC:6.6.1.2] | | |
| K09888 | 10 | cell division protein ZapA | | |
| K09889 | 3 | hypothetical protein | | |
| K09891 | 1 | hypothetical protein | | |
| K09895 | 4 | hypothetical protein | | |
| K09903 | 23 | uridylate kinase [EC:2.7.4.22] | | |
| K09906 | 1 | hypothetical protein | | |
| K09909 | 2 | hypothetical protein | | |
| K09913 | 1 | hypothetical protein | | |
| K09914 | 4 | putative lipoprotein | | |
| K09915 | 12 | hypothetical protein | | |
| K09916 | 5 | hypothetical protein | | |
| K09917 | 1 | hypothetical protein | | |
| K09918 | 2 | hypothetical protein | | |
| K09919 | 9 | hypothetical protein | | |
| K09921 | 6 | hypothetical protein | | |

| | | | | |
|---|---|---|---|---|
| K09922 | 3 | hypothetical protein | | |
| K09923 | 1 | hypothetical protein | | |
| K09924 | 5 | hypothetical protein | | |
| K09926 | 2 | hypothetical protein | | |
| K09927 | 30 | hypothetical protein | | |
| K09928 | 3 | hypothetical protein | | |
| K09930 | 9 | hypothetical protein | | |
| K09931 | 19 | hypothetical protein | | |
| K09933 | 8 | hypothetical protein | | |
| K09934 | 3 | hypothetical protein | | |
| K09935 | 1 | hypothetical protein | | |
| K09936 | 3 | hypothetical protein | | |
| K09937 | 2 | hypothetical protein | | |
| K09939 | 1 | hypothetical protein | | |
| K09941 | 1 | hypothetical protein | | |
| K09942 | 1 | hypothetical protein | | |
| K09949 | 15 | hypothetical protein | | |
| K09950 | 1 | hypothetical protein | | |
| K09951 | 1 | CRISPR-associated protein Cas2 | | |
| K09952 | 6 | hypothetical protein | | |
| K09953 | 2 | lipid A 3-O-deacylase | | |
| K09955 | 22 | hypothetical protein | | |
| K09958 | 8 | hypothetical protein | | |
| K09959 | 1 | hypothetical protein | | |
| K09962 | 1 | hypothetical protein | | |
| K09966 | 6 | hypothetical protein | | |
| K09967 | 7 | hypothetical protein | | |
| K09969 | 7 | general L-amino acid transport system substrate-binding protein | | |
| K09970 | 2 | general L-amino acid transport system permease protein | | |
| K09971 | 7 | general L-amino acid transport system permease protein | | |
| K09972 | 2 | general L-amino acid transport system ATP-binding protein | | |
| K09973 | 11 | hypothetical protein | | |
| K09974 | 1 | hypothetical protein | | |
| K09978 | 1 | hypothetical protein | | |
| K09979 | 1 | hypothetical protein | | |
| K09981 | 4 | hypothetical protein | | |
| K09983 | 6 | hypothetical protein | | |
| K09984 | 4 | hypothetical protein | | |
| K09986 | 2 | hypothetical protein | | |
| K09988 | 2 | hypothetical protein | | |
| K09989 | 5 | hypothetical protein | | |
| K09990 | 7 | hypothetical protein | | |
| K09992 | 5 | hypothetical protein | | |
| K09994 | 1 | PhnO protein [EC:2.3.1.-] | | |
| K09996 | 9 | arginine transport system substrate-binding protein | | |
| K09999 | 2 | arginine transport system permease protein | | |
| K10001 | 14 | glutamate/aspartate transport system substrate-binding protein | | |
| K10002 | 5 | glutamate/aspartate transport system permease protein | | |
| K10003 | 8 | glutamate/aspartate transport system permease protein | | |
| K10004 | 3 | glutamate/aspartate transport system ATP-binding protein | | |

| | | | | |
|---|---|---|---|---|
| K10005 | 1 | glutamate transport system substrate-binding protein | | |
| K10006 | 2 | glutamate transport system permease protein | | |
| K10007 | 2 | glutamate transport system permease protein | | |
| K10009 | 4 | cystine transport system permease protein | | |
| K10010 | 1 | cystine transport system ATP-binding protein [EC:3.6.3.-] | | |
| K10011 | 7 | UDP-GlcUA decarboxylase/UDP-L-Ara4N formyltransferase | | |
| K10012 | 8 | undecaprenyl-phosphate 4-deoxy-4-formamido-L-arabinose transferase | | |
| K10014 | 1 | histidine transport system substrate-binding protein | | |
| K10017 | 1 | histidine transport system ATP-binding protein [EC:3.6.3.21] | | |
| K10026 | 15 | queuosine biosynthesis protein QueE | | |
| K10027 | 11 | phytoene dehydrogenase [EC:1.14.99.-] | | |
| K10036 | 2 | glutamine transport system substrate-binding protein | | |
| K10037 | 3 | glutamine transport system permease protein | | |
| K10039 | 6 | putative glutamine transport system substrate-binding protein | | |
| K10040 | 3 | putative glutamine transport system permease protein | | |
| K10041 | 2 | putative glutamine transport system ATP-binding protein [EC:3.6.3.-] | | |
| K10108 | 11 | maltose/maltodextrin transport system substrate-binding protein | | |
| K10109 | 5 | maltose/maltodextrin transport system permease protein | | |
| K10110 | 13 | maltose/maltodextrin transport system permease protein | | |
| K10111 | 2 | maltose/maltodextrin transport system ATP-binding protein | | |
| K10112 | 10 | maltose/maltodextrin transport system ATP-binding protein | | |
| K10117 | 1 | multiple sugar transport system substrate-binding protein | | |
| K10118 | 3 | multiple sugar transport system permease protein | | |
| K10119 | 3 | multiple sugar transport system permease protein | | |
| K10120 | 2 | putative sugar transport system substrate-binding protein | | |
| K10121 | 12 | putative sugar transport system permease protein | | |
| K10122 | 6 | putative sugar transport system permease protein | | |
| K10125 | 16 | two-component system, NtrC family, C4-dicarboxylate transport sensor | | |
| K10126 | 65 | two-component system, NtrC family, C4-dicarboxylate transport | | |
| K10188 | 9 | lactose/L-arabinose transport system substrate-binding protein | | |
| K10189 | 8 | lactose/L-arabinose transport system permease protein | | |
| K10190 | 19 | lactose/L-arabinose transport system permease protein | | |
| K10191 | 1 | lactose/L-arabinose transport system ATP-binding protein | | |
| K10193 | 2 | oligogalacturonide transport system permease protein | | |
| K10200 | 3 | N-acetylglucosamine transport system substrate-binding protein | | |
| K10201 | 3 | N-acetylglucosamine transport system permease protein | | |
| K10206 | 25 | LL-diaminopimelate aminotransferase [EC:2.6.1.83] | | |
| K10209 | 1 | dehydrosqualene desaturase [EC:1.14.99.-] | | |
| K10210 | 3 | 4,4'-diaponeurosporene oxidase [EC:1.-.-.-] | | |
| K10215 | 11 | monooxygenase [EC:1.14.13.-] | | |
| K10217 | 4 | aminomuconate-semialdehyde dehydrogenase [EC:1.2.1.32] | | |
| K10218 | 5 | 4-hydroxy-4-methyl-2-oxoglutarate aldolase [EC:4.1.3.17] | | |
| K10219 | 34 | 4-carboxy-2-hydroxymuconate-6-semialdehyde dehydrogenase | | |
| K10220 | 52 | 4-oxalmesaconate hydratase [EC:4.2.1.83] | | |
| K10221 | 17 | 2-pyrone-4,6-dicarboxylate lactonase [EC:3.1.1.57] | | |
| K10227 | 10 | sorbitol/mannitol transport system substrate-binding protein | | |
| K10228 | 1 | sorbitol/mannitol transport system permease protein | | |
| K10229 | 2 | sorbitol/mannitol transport system permease protein | | |
| K10230 | 1 | sorbitol/mannitol transport system ATP-binding protein | | |
| K10231 | 10 | kojibiose phosphorylase [EC:2.4.1.230] | | |

| | | | | |
|---|---|---|---|---|
| K10232 | 15 | alpha-glucoside transport system substrate-binding protein | | |
| K10233 | 5 | alpha-glucoside transport system permease protein | | |
| K10234 | 8 | alpha-glucoside transport system permease protein | | |
| K10236 | 6 | trehalose/maltose transport system substrate-binding protein | | |
| K10237 | 4 | trehalose/maltose transport system permease protein | | |
| K10238 | 3 | trehalose/maltose transport system permease protein | | |
| K10239 | 1 | trehalose/maltose transport system ATP-binding protein | | |
| K10240 | 2 | cellobiose transport system substrate-binding protein | | |
| K10241 | 1 | cellobiose transport system permease protein | | |
| K10242 | 1 | cellobiose transport system permease protein | | |
| K10243 | 2 | cellobiose transport system ATP-binding protein | | |
| K10253 | 2 | DOPA 4,5-dioxygenase [EC:1.14.99.-] | | |
| K10254 | 1 | myosin-crossreactive antigen | | |
| K10255 | 15 | omega-6 fatty acid desaturase (delta-12 desaturase) [EC:1.14.19.-] | | |
| K10353 | 5 | deoxyadenosine kinase [EC:2.7.1.76] | | |
| K10439 | 21 | ribose transport system substrate-binding protein | | |
| K10440 | 29 | ribose transport system permease protein | | |
| K10441 | 27 | ribose transport system ATP-binding protein [EC:3.6.3.17] | | |
| K10533 | 3 | limonene-1,2-epoxide hydrolase [EC:3.3.2.8] | | |
| K10536 | 12 | agmatine deiminase [EC:3.5.3.12] | | |
| K10538 | 1 | L-arabinose transport system permease protein | | |
| K10541 | 3 | methyl-galactoside transport system permease protein | | |
| K10542 | 1 | methyl-galactoside transport system ATP-binding protein | | |
| K10543 | 5 | D-xylose transport system substrate-binding protein | | |
| K10544 | 14 | D-xylose transport system permease protein | | |
| K10545 | 1 | D-xylose transport system ATP-binding protein [EC:3.6.3.17] | | |
| K10547 | 1 | putative multiple sugar transport system permease protein | | |
| K10549 | 2 | D-allose transport system substrate-binding protein | | |
| K10552 | 6 | fructose transport system substrate-binding protein | | |
| K10553 | 2 | fructose transport system permease protein | | |
| K10554 | 2 | fructose transport system ATP-binding protein | | |
| K10555 | 9 | AI-2 transport system substrate-binding protein | | |
| K10556 | 4 | AI-2 transport system permease protein | | |
| K10557 | 2 | AI-2 transport system permease protein | | |
| K10559 | 7 | rhamnose transport system substrate-binding protein | | |
| K10560 | 3 | rhamnose transport system permease protein | | |
| K10561 | 1 | rhamnose transport system permease protein | | |
| K10563 | 62 | formamidopyrimidine-DNA glycosylase [EC:3.2.2.23 4.2.99.18] | | |
| K10616 | 19 | p-cymene monooxygenase [EC:1.14.13.-] | | |
| K10617 | 7 | p-cumic alcohol dehydrogenase | | |
| K10618 | 7 | p-cumic aldehyde dehydrogenase | | |
| K10619 | 17 | p-cumate dioxygenase [EC:1.14.12.-] | | |
| K10620 | 1 | 2,3-dihydroxy-2,3-dihydro-p-cumate dehydrogenase [EC:1.3.1.58] | | |
| K10621 | 5 | 2,3-dihydroxy-p-cumate-3,4-dioxygenase [EC:1.13.11.-] | | |
| K10622 | 2 | HCOMODA decarboxylase [EC:4.1.1.-] | | |
| K10674 | 2 | ectoine hydroxylase [EC:1.14.11.-] | | |
| K10676 | 13 | 2,4-dichlorophenol 6-monooxygenase [EC:1.14.13.20] | | |
| K10678 | 1 | nitroreductase [EC:1.-.-.-] | | |
| K10679 | 1 | nitroreductase / dihydropteridine reductase [EC:1.-.-.- 1.5.1.34] | | |
| K10680 | 11 | N-ethylmaleimide reductase [EC:1.-.-.-] | | |

| | | | | |
|---|---|---|---|---|
| K10697 | 6 | two-component system, OmpR family, response regulator RpaA | | |
| K10700 | 13 | ethylbenzene hydroxylase [EC:1.17.99.2] | | |
| K10701 | 20 | acetophenone carboxylase [EC:6.4.1.-] | | |
| K10708 | 1 | fructoselysine 6-phosphate deglycase [EC:3.5.-.-] | | |
| K10711 | 1 | GntR family transcriptional regulator, frlABCD operon | | |
| K10713 | 1 | formaldehyde-activating enzyme [EC:4.3.-.-] | | |
| K10715 | 7 | two-component system, sensor histidine kinase RpfC [EC:2.7.13.3] | | |
| K10716 | 1 | voltage-gated potassium channel | | |
| K10747 | 4 | DNA ligase 1 [EC:6.5.1.1] | | |
| K10754 | 4 | replication factor C subunit 1 | | |
| K10763 | 2 | DnaA-homolog protein | | |
| K10764 | 12 | O-succinylhomoserine sulfhydrylase [EC:2.5.1.-] | | |
| K10773 | 24 | endonuclease III [EC:4.2.99.18] | | |
| K10774 | 6 | tyrosine ammonia-lyase [EC:4.3.1.23] | | |
| K10775 | 1 | phenylalanine ammonia-lyase [EC:4.3.1.24] | | |
| K10778 | 19 | AraC family transcriptional regulator, regulatory protein of | | |
| K10780 | 3 | enoyl-[acyl carrier protein] reductase III [EC:1.3.1.-] | | |
| K10799 | 18 | tankyrase [EC:2.4.2.30] | | |
| K10804 | 10 | acyl-CoA thioesterase I [EC:3.1.2.- 3.1.1.5] | | |
| K10805 | 10 | acyl-CoA thioesterase II [EC:3.1.2.-] | | |
| K10806 | 4 | acyl-CoA thioesterase YciA [EC:3.1.2.-] | | |
| K10807 | 1 | ribonucleoside-diphosphate reductase subunit M1 [EC:1.17.4.1] | | |
| K10810 | 1 | transcriptional regulator TenI | | |
| K10815 | 1 | hydrogen cyanide synthase HcnB [EC:1.4.99.5] | | |
| K10816 | 2 | hydrogen cyanide synthase HcnC [EC:1.4.99.5] | | |
| K10817 | 32 | erythronolide synthase [EC:2.3.1.94] | | |
| K10819 | 143 | histidine kinase | | |
| K10820 | 13 | monosaccharide-transporting ATPase [EC:3.6.3.17] | | |
| K10822 | 21 | nonpolar-amino-acid-transporting ATPase [EC:3.6.3.22] | | |
| K10826 | 34 | Fe3+-transporting ATPase [EC:3.6.3.30] | | |
| K10828 | 1 | quaternary-amine-transporting ATPase [EC:3.6.3.32] | | |
| K10829 | 1 | iron-chelate-transporting ATPase [EC:3.6.3.34] | | |
| K10834 | 7 | heme-transporting ATPase [EC:3.6.3.41] | | |
| K10843 | 12 | DNA excision repair protein ERCC-3 [EC:3.6.1.-] | | |
| K10844 | 3 | DNA excision repair protein ERCC-2 [EC:3.6.1.-] | | |
| K10855 | 1 | acetone carboxylase, beta subunit [EC:6.4.1.6] | | |
| K10857 | 1 | exodeoxyribonuclease X [EC:3.1.11.-] | | |
| K10907 | 72 | aminotransferase [EC:2.6.1.-] | | |
| K10908 | 1 | DNA-directed RNA polymerase, mitochondrial [EC:2.7.7.6] | | |
| K10912 | 2 | two-component system, repressor protein LuxO | | |
| K10914 | 3 | CRP/FNR family transcriptional regulator, cyclic AMP receptor | | |
| K10915 | 1 | CAI-1 autoinducer synthase [EC:2.3.-.-] | | |
| K10917 | 8 | PadR family transcriptional regulator, regulatory protein AphA | | |
| K10918 | 14 | LysR family transcriptional regulator, transcriptional activator | | |
| K10941 | 18 | sigma-54 specific transcriptional regulator, flagellar regulatory | | |
| K10942 | 29 | two-component system, sensor histidine kinase FlrB [EC:2.7.13.3] | | |
| K10943 | 57 | two component system, response regulator FlrC | | |
| K10947 | 19 | PadR family transcriptional regulator, regulatory protein PadR | | |
| K10972 | 1 | LysR family transcriptional regulator, transcriptional activator of | | |
| K10979 | 36 | DNA end-binding protein Ku | | |

| | | | | |
|---|---|---|---|---|
| K11003 | 4 | hemolysin D | | |
| K11004 | 5 | ATP-binding cassette, subfamily B, bacterial HlyB/CyaB | | |
| K11005 | 15 | hemolysin A | | |
| K11016 | 4 | hemolysin | | |
| K11017 | 8 | hemolysin activation/secretion protein?? | | |
| K11029 | 4 | anthrax edema toxin adenylate cyclase [EC:4.6.1.1] | | |
| K11031 | 2 | thiol-activated cytolysin | | |
| K11041 | 2 | exfoliative toxin A/B | | |
| K11049 | 16 | CylG protein | | |
| K11053 | 3 | CylF protein | | |
| K11060 | 5 | probable enterotoxin B | | |
| K11062 | 1 | probable enterotoxin D | | |
| K11065 | 10 | thiol peroxidase, atypical 2-Cys peroxiredoxin [EC:1.11.1.15] | | |
| K11066 | 3 | N-acetylmuramoyl-L-alanine amidase [EC:3.5.1.28] | | |
| K11068 | 27 | hemolysin III | | |
| K11069 | 17 | spermidine/putrescine transport system substrate-binding protein | | |
| K11070 | 19 | spermidine/putrescine transport system permease protein | | |
| K11071 | 27 | spermidine/putrescine transport system permease protein | | |
| K11072 | 19 | spermidine/putrescine transport system ATP-binding protein | | |
| K11073 | 4 | putrescine transport system substrate-binding protein | | |
| K11074 | 10 | putrescine transport system permease protein | | |
| K11075 | 10 | putrescine transport system permease protein | | |
| K11076 | 10 | putrescine transport system ATP-binding protein | | |
| K11079 | 3 | mannopine transport system permease protein | | |
| K11082 | 1 | 2-aminoethylphosphonate transport system permease protein | | |
| K11085 | 118 | ATP-binding cassette, subfamily B, bacterial MsbA [EC:3.6.3.-] | | |
| K11102 | 12 | proton glutamate symport protein | | |
| K11103 | 9 | aerobic C4-dicarboxylate transport protein | | |
| K11105 | 21 | cell volume regulation protein A | | |
| K11106 | 6 | L-tartrate/succinate antiporter | | |
| K11144 | 8 | primosomal protein DnaI | | |
| K11145 | 2 | ribonuclease III family protein | | |
| K11159 | 3 | carotenoid cleavage dioxygenase | | |
| K11173 | 9 | hydroxyacid-oxoacid transhydrogenase [EC:1.1.99.24] | | |
| K11174 | 8 | carbon-monoxide dehydrogenase (ferredoxin) [EC:1.2.7.4] | | |
| K11175 | 32 | phosphoribosylglycinamide formyltransferase 1 [EC:2.1.2.2] | | |
| K11177 | 61 | xanthine dehydrogenase YagR molybdenum-binding subunit [EC:1.17.1.4] | | |
| K11178 | 19 | xanthine dehydrogenase YagS FAD-binding subunit [EC:1.17.1.4] | | |
| K11179 | 14 | tRNA 2-thiouridine synthesizing protein E [EC:2.8.1.-] | | |
| K11180 | 3 | sulfite reductase, dissimilatory-type alpha subunit [EC:1.8.99.3] | | |
| K11181 | 3 | sulfite reductase, dissimilatory-type beta subunit [EC:1.8.99.3] | | |
| K11183 | 16 | phosphocarrier protein FPr | | |
| K11189 | 29 | phosphocarrier protein | | |
| K11192 | 1 | PTS system, N-acetylmuramic acid-specific IIC component | | |
| K11201 | 2 | PTS system, fructose-specific IIA-like component [EC:2.7.1.69] | | |
| K11209 | 5 | GST-like protein | | |
| K11212 | 20 | LPPG:FO 2-phospho-L-lactate transferase [EC:2.7.8.-] | | |
| K11216 | 1 | autoinducer 2 (AI-2) kinase [EC:2.7.1.-] | | |
| K11249 | 1 | cysteine/O-acetylserine efflux protein | | |
| K11250 | 1 | leucine efflux protein | | |

| | | | | |
|---|---|---|---|---|
| K11258 | 2 | acetolactate synthase II small subunit [EC:2.2.1.6] | | |
| K11261 | 6 | formylmethanofuran dehydrogenase subunit E [EC:1.2.99.5] | | |
| K11263 | 26 | acetyl-/propionyl-CoA carboxylase, biotin carboxylase, biotin | | |
| K11264 | 2 | methylmalonyl-CoA decarboxylase [EC:4.1.1.41] | | |
| K11311 | 2 | anthranilate dioxygenase reductase | | |
| K11312 | 6 | cupin 2 domain-containing protein | | |
| K11325 | 6 | L-cysteine/cystine lyase | | |
| K11326 | 7 | cation efflux system protein involved in nickel and cobalt tolerance | | |
| K11329 | 27 | two-component system, OmpR family, response regulator RpaB | | |
| K11333 | 1 | chlorophyllide reductase iron protein subunit X [EC:1.18.6.1] | | |
| K11337 | 3 | 3-hydroxyethyl bacteriochlorophyllide a dehydrogenase [EC:1.-.-.-] | | |
| K11354 | 3 | two-component system, chemotaxis family, sensor kinase Cph1 | | |
| K11355 | 3 | two-component system, chemotaxis family, response regulator Rcp1 | | |
| K11356 | 2 | two-component system, sensor histidine kinase and response regulator | | |
| K11357 | 7 | two-component system, cell cycle sensor histidine kinase DivJ | | |
| K11358 | 4 | aspartate aminotransferase [EC:2.6.1.1] | | |
| K11381 | 62 | 2-oxoisovalerate dehydrogenase E1 component [EC:1.2.4.4] | | |
| K11383 | 3 | two-component system, NtrC family, sensor histidine kinase KinB | | |
| K11384 | 7 | two-component system, NtrC family, response regulator AlgB | | |
| K11418 | 13 | histone deacetylase 11 [EC:3.5.1.98] | | |
| K11443 | 7 | two-component system, cell cycle response regulator DivK | | |
| K11444 | 8 | two-component system, chemotaxis family, response regulator WspR | | |
| K11472 | 15 | glycolate oxidase FAD binding subunit | | |
| K11473 | 36 | glycolate oxidase iron-sulfur subunit | | |
| K11475 | 15 | GntR family transcriptional regulator, vanillate catabolism | | |
| K11476 | 4 | GntR family transcriptional regulator, gluconate operon | | |
| K11477 | 13 | glc operon protein GlcG | | |
| K11523 | 4 | two-component system, chemotaxis family, response regulator PixH | | |
| K11525 | 1 | methyl-accepting chemotaxis protein PixJ | | |
| K11526 | 1 | two-component system, chemotaxis family, sensor histidine kinase and | | |
| K11527 | 58 | two-component system, unclassified family, sensor histidine kinase | | |
| K11528 | 4 | UDP-N-acetylglucosamine pyrophosphorylase [EC:2.7.7.23] | | |
| K11531 | 1 | lsr operon transcriptional repressor | | |
| K11532 | 1 | fructose-1,6-bisphosphatase II / sedoheptulose-1,7-bisphosphatase | | |
| K11533 | 1 | fatty acid synthase, bacteria type [EC:2.3.1.-] | | |
| K11537 | 6 | MFS transporter, NHS family, xanthosine permease | | |
| K11603 | 1 | manganese transport system ATP-binding protein | | |
| K11604 | 1 | manganese/iron transport system substrate-binding protein | | |
| K11606 | 3 | manganese/iron transport system permease protein | | |
| K11608 | 1 | beta-ketoacyl ACP synthase [EC:2.3.1.-] | | |
| K11609 | 1 | beta-ketoacyl ACP synthase [EC:2.3.1.-] | | |
| K11610 | 1 | beta-ketoacyl ACP reductase [EC:1.1.1.100] | | |
| K11611 | 8 | enoyl ACP reductase [EC:1.3.1.9] | | |
| K11615 | 3 | two-component system, CitB family, response regulator MalR | | |
| K11616 | 1 | malate:Na+ symporter | | |
| K11617 | 3 | two-component system, NarL family, sensor histidine kinase LiaS | | |
| K11618 | 39 | two-component system, NarL family, response regulator LiaR | | |
| K11619 | 11 | lia operon protein LiaI | | |
| K11621 | 1 | lia operon protein LiaG | | |
| K11624 | 2 | two-component system, NarL family, response regulator YdfI | | |

| | | | | |
|---|---|---|---|---|
| K11625 | 9 | membrane protein YdfJ | | |
| K11634 | 1 | two-component system, OmpR family, response regulator YxdJ | | |
| K11646 | 1 | dehydroquinate synthase II [EC:1.4.1.-] | | |
| K11688 | 92 | C4-dicarboxylate-binding protein DctP | | |
| K11689 | 12 | C4-dicarboxylate transporter, DctQ subunit | | |
| K11690 | 144 | C4-dicarboxylate transporter, DctM subunit | | |
| K11693 | 2 | peptidoglycan pentaglycine glycine transferase (the first glycine) | | |
| K11710 | 3 | manganese/zinc/iron transport system ATP- binding protein | | |
| K11711 | 16 | two-component system, LuxR family, sensor histidine kinase DctS | | |
| K11712 | 12 | two-component system, LuxR family, response regulator DctR | | |
| K11717 | 72 | cysteine desulfurase / selenocysteine lyase [EC:2.8.1.7 4.4.1.16] | | |
| K11719 | 2 | lipopolysaccharide export system protein LptC | | |
| K11720 | 14 | lipopolysaccharide export system permease protein | | |
| K11731 | 6 | citronellyl-CoA dehydrogenase [EC:1.3.99.-] | | |
| K11740 | 2 | bacteriophage N4 adsorption protein B | | |
| K11741 | 6 | quaternary ammonium compound-resistance protein SugE | | |
| K11744 | 5 | AI-2 transport protein TqsA | | |
| K11745 | 10 | glutathione-regulated potassium-efflux system ancillary protein KefC | | |
| K11746 | 1 | glutathione-regulated potassium-efflux system ancillary protein KefF | | |
| K11747 | 9 | glutathione-regulated potassium-efflux system protein KefB | | |
| K11748 | 1 | glutathione-regulated potassium-efflux system ancillary protein KefG | | |
| K11749 | 54 | regulator of sigma E protease [EC:3.4.24.-] | | |
| K11750 | 11 | esterase FrsA [EC:3.1.-.-] | | |
| K11751 | 4 | 5'-nucleotidase / UDP-sugar diphosphatase [EC:3.1.3.5 3.6.1.45] | | |
| K11752 | 32 | diaminohydroxyphosphoribosylaminopyrimidine deaminase / | | |
| K11753 | 31 | riboflavin kinase / FMN adenylyltransferase [EC:2.7.1.26 2.7.7.2] | | |
| K11754 | 47 | dihydrofolate synthase / folylpolyglutamate synthase [EC:6.3.2.12 | | |
| K11755 | 12 | phosphoribosyl-ATP pyrophosphohydrolase / phosphoribosyl-AMP | | |
| K11777 | 1 | HAD superfamily phosphatase | | |
| K11779 | 20 | FO synthase [EC:2.5.1.-] | | |
| K11781 | 1 | FO synthase subunit 2 [EC:2.5.1.-] | | |
| K11782 | 5 | chorismate futalosine-lyase [EC:4.1.99.-] | | |
| K11784 | 17 | de-hypoxanthine futalosine synthase [EC:1.21.-.-] | | |
| K11785 | 2 | 1,4-dihydroxy-6-naphthoate synthase [EC:1.14.-.-] | | |
| K11890 | 5 | type VI secretion system protein ImpM | | |
| K11891 | 8 | type VI secretion system protein ImpL | | |
| K11892 | 8 | type VI secretion system protein ImpK | | |
| K11893 | 9 | type VI secretion system protein ImpJ | | |
| K11894 | 3 | type VI secretion system protein ImpI | | |
| K11895 | 4 | type VI secretion system protein ImpH | | |
| K11896 | 7 | type VI secretion system protein ImpG | | |
| K11897 | 2 | type VI secretion system protein ImpF | | |
| K11898 | 1 | type VI secretion system protein ImpE | | |
| K11899 | 2 | type VI secretion system protein ImpD | | |
| K11900 | 10 | type VI secretion system protein ImpC | | |
| K11901 | 8 | type VI secretion system protein ImpB | | |
| K11902 | 6 | type VI secretion system protein ImpA | | |
| K11903 | 1 | type VI secretion system secreted protein Hcp | | |
| K11904 | 9 | type VI secretion system secreted protein VgrG | | |
| K11905 | 1 | type VI secretion system protein | | |

| | | | | |
|---|---|---|---|---|
| K11906 | 7 | type VI secretion system protein VasD | | |
| K11907 | 13 | type VI secretion system protein VasG | | |
| K11910 | 2 | type VI secretion system protein VasJ | | |
| K11911 | 1 | type VI secretion system protein VasL | | |
| K11912 | 28 | serine/threonine-protein kinase PpkA [EC:2.7.11.1] | | |
| K11913 | 3 | type VI secretion system protein | | |
| K11914 | 2 | sigma-54 dependent transcriptional regulator | | |
| K11916 | 1 | serine/threonine-protein kinase Stk1 [EC:2.7.11.-] | | |
| K11917 | 3 | sigma-54 specific transcriptional regulator | | |
| K11921 | 10 | LysR family transcriptional regulator | | |
| K11922 | 1 | GntR family transcriptional regulator, mannosyl-D-glycerate | | |
| K11923 | 3 | MerR family transcriptional regulator, copper efflux regulator | | |
| K11924 | 4 | DtxR family transcriptional regulator, manganese transport regulator | | |
| K11925 | 1 | SgrR family transcriptional regulator | | |
| K11926 | 1 | sigma factor-binding protein Crl | | |
| K11927 | 27 | ATP-dependent RNA helicase RhlE [EC:3.6.4.13] | | |
| K11928 | 6 | sodium/proline symporter | | |
| K11930 | 1 | periplasmic protein TorT | | |
| K11931 | 5 | biofilm PGA synthesis lipoprotein PgaB [EC:3.-.-.-] | | |
| K11933 | 4 | NADH oxidoreductase Hcr [EC:1.-.-.-] | | |
| K11936 | 26 | biofilm PGA synthesis N-glycosyltransferase PgaC [EC:2.4.-.-] | | |
| K11938 | 1 | HMP-PP phosphatase [EC:3.6.1.-] | | |
| K11939 | 8 | inner membrane transporter RhtA | | |
| K11940 | 2 | heat shock protein HspQ | | |
| K11942 | 14 | methylmalonyl-CoA mutase [EC:5.4.99.2] | | |
| K11948 | 2 | 1-hydroxy-2-naphthoate dioxygenase [EC:1.13.11.38] | | |
| K11949 | 1 | 4-(2-carboxyphenyl)-2-oxobut-3-enoate aldolase [EC:4.1.2.34] | | |
| K11953 | 1 | bicarbonate transport system ATP-binding protein [EC:3.6.3.-] | | |
| K11954 | 2 | neutral amino acid transport system substrate-binding protein | | |
| K11955 | 1 | neutral amino acid transport system permease protein | | |
| K11956 | 1 | neutral amino acid transport system permease protein | | |
| K11957 | 2 | neutral amino acid transport system ATP-binding protein | | |
| K11958 | 2 | neutral amino acid transport system ATP-binding protein | | |
| K11959 | 4 | urea transport system substrate-binding protein | | |
| K11960 | 1 | urea transport system permease protein | | |
| K11961 | 2 | urea transport system permease protein | | |
| K11963 | 1 | urea transport system ATP-binding protein | | |
| K11987 | 1 | prostaglandin-endoperoxide synthase 2 [EC:1.14.99.1] | | |
| K11991 | 12 | tRNA-specific adenosine deaminase [EC:3.5.4.-] | | |
| K12059 | 1 | conjugal transfer pilus assembly protein TrbC | | |
| K12063 | 2 | conjugal transfer ATP-binding protein TraC | | |
| K12065 | 1 | conjugal transfer pilus assembly protein TraB | | |
| K12073 | 3 | 1,4-dihydroxy-2-naphthoyl-CoA thioesterase [EC:3.1.2.-] | | |
| K12111 | 2 | evolved beta-galactosidase subunit alpha [EC:3.2.1.23] | | |
| K12137 | 9 | hydrogenase-4 component B [EC:1.-.-.-] | | |
| K12138 | 10 | hydrogenase-4 component C [EC:1.-.-.-] | | |
| K12139 | 6 | hydrogenase-4 component D [EC:1.-.-.-] | | |
| K12140 | 6 | hydrogenase-4 component E [EC:1.-.-.-] | | |
| K12141 | 14 | hydrogenase-4 component F [EC:1.-.-.-] | | |
| K12142 | 1 | hydrogenase-4 component G [EC:1.-.-.-] | | |

| | | | | |
|---|---|---|---|---|
| K12143 | 4 | hydrogenase-4 component H | | |
| K12146 | 16 | hydrogenase-4 transcriptional activator | | |
| K12148 | 3 | biofilm regulator BssS | | |
| K12150 | 1 | HIT-like protein HinT | | |
| K12152 | 3 | phosphatase NudJ [EC:3.6.1.-] | | |
| K12203 | 34 | defect in organelle trafficking protein DotB | | |
| K12206 | 6 | intracellular multiplication protein IcmB | | |
| K12209 | 3 | intracellular multiplication protein IcmE | | |
| K12210 | 6 | intracellular multiplication protein IcmF | | |
| K12213 | 10 | intracellular multiplication protein IcmK | | |
| K12214 | 5 | intracellular multiplication protein IcmL | | |
| K12216 | 1 | intracellular multiplication protein IcmN | | |
| K12234 | 9 | F420-0:gamma-glutamyl ligase [EC:6.3.2.-] | | |
| K12238 | 5 | pyochelin biosynthesis protein PchD | | |
| K12240 | 1 | pyochelin synthetase | | |
| K12251 | 28 | N-carbamoylputrescine amidase [EC:3.5.1.53] | | |
| K12252 | 2 | arginine:pyruvate transaminase [EC:2.6.1.84] | | |
| K12253 | 4 | 5-guanidino-2-oxopentanoate decarboxylase [EC:4.1.1.75] | | |
| K12254 | 3 | 4-guanidinobutyraldehyde dehydrogenase / NAD-dependent aldehyde | | |
| K12255 | 4 | guanidinobutyrase [EC:3.5.3.7] | | |
| K12256 | 7 | putrescine aminotransferase [EC:2.6.1.-] | | |
| K12257 | 30 | SecD/SecF fusion protein | | |
| K12262 | 5 | cytochrome b561 | | |
| K12264 | 2 | anaerobic nitric oxide reductase flavorubredoxin | | |
| K12265 | 1 | nitric oxide reductase FlRd-NAD(+) reductase [EC:1.18.1.-] | | |
| K12266 | 6 | anaerobic nitric oxide reductase transcription regulator | | |
| K12267 | 22 | peptide methionine sulfoxide reductase msrA/msrB [EC:1.8.4.11 | | |
| K12276 | 10 | MSHA biogenesis protein MshE | | |
| K12278 | 8 | MSHA biogenesis protein MshG | | |
| K12282 | 9 | MSHA biogenesis protein MshL | | |
| K12283 | 7 | MSHA biogenesis protein MshM | | |
| K12287 | 4 | MSHA biogenesis protein MshQ | | |
| K12297 | 4 | ribosomal RNA large subunit methyltransferase L [EC:2.1.1.52] | | |
| K12299 | 1 | MFS transporter, ACS family, probable galactarate transporter | | |
| K12308 | 35 | beta-galactosidase [EC:3.2.1.23] | | |
| K12339 | 16 | cysteine synthase B [EC:2.5.1.47] | | |
| K12340 | 36 | outer membrane channel protein TolC | | |
| K12343 | 4 | 3-oxo-5-alpha-steroid 4-dehydrogenase 1 [EC:1.3.99.5] | | |
| K12344 | 4 | 3-oxo-5-alpha-steroid 4-dehydrogenase 2 [EC:1.3.99.5] | | |
| K12368 | 17 | dipeptide transport system substrate-binding protein | | |
| K12369 | 12 | dipeptide transport system permease protein | | |
| K12370 | 10 | dipeptide transport system permease protein | | |
| K12371 | 6 | dipeptide transport system ATP-binding protein | | |
| K12372 | 1 | dipeptide transport system ATP-binding protein | | |
| K12373 | 31 | beta-hexosaminidase [EC:3.2.1.52] | | |
| K12410 | 28 | NAD-dependent deacetylase [EC:3.5.1.-] | | |
| K12420 | 16 | ketoacyl reductase [EC:1.3.1.-] | | |
| K12421 | 1 | fatty acid CoA ligase FadD9 | | |
| K12423 | 2 | fatty acid CoA ligase FadD21 | | |
| K12424 | 2 | fatty acid CoA ligase FadD22 | | |

| | | | | |
|---|---|---|---|---|
| K12427 | 1 | fatty acid CoA ligase FadD28 | | |
| K12428 | 1 | fatty acid CoA ligase FadD32 | | |
| K12429 | 5 | fatty acid CoA ligase FadD36 | | |
| K12430 | 3 | polyketide synthase 1/15 | | |
| K12432 | 1 | polyketide synthase 3/4 | | |
| K12436 | 22 | polyketide synthase 12 | | |
| K12437 | 1 | polyketide synthase 13 | | |
| K12441 | 1 | phenolpthiocerol synthesis type-I polyketide synthase B | | |
| K12442 | 1 | phenolpthiocerol synthesis type-I polyketide synthase C | | |
| K12444 | 3 | phenolpthiocerol synthesis type-I polyketide synthase E | | |
| K12445 | 2 | trans enoyl reductase [EC:1.3.1.-] | | |
| K12452 | 6 | CDP-6-deoxy-D-xylo-4-hexulose-3-dehydrase | | |
| K12454 | 22 | CDP-paratose 2-epimerase [EC:5.1.3.10] | | |
| K12500 | 1 | thioesterase III [EC:3.1.2.-] | | |
| K12503 | 5 | short-chain Z-isoprenyl diphosphate synthase [EC:2.5.1.68] | | |
| K12506 | 29 | 2-C-methyl-D-erythritol 4-phosphate cytidylyltransferase / | | |
| K12507 | 2 | acyl-CoA synthetase [EC:6.2.1.-] | | |
| K12508 | 42 | feruloyl-CoA synthase [EC:6.2.1.34] | | |
| K12510 | 17 | tight adherence protein B | | |
| K12511 | 16 | tight adherence protein C | | |
| K12524 | 14 | bifunctional aspartokinase/homoserine dehydrogenase 1 [EC:2.7.2.4 | | |
| K12525 | 1 | bifunctional aspartokinase/homoserine dehydrogenase 2 [EC:2.7.2.4 | | |
| K12526 | 1 | bifunctional diaminopimelate decarboxylase/aspartate kinase | | |
| K12527 | 2 | putative selenate reductase [EC:1.97.1.9] | | |
| K12528 | 2 | putative selenate reductase molybdopterin-binding subunit | | |
| K12536 | 4 | ATP-binding cassette, subfamily C, bacterial HasD | | |
| K12537 | 2 | protease secretion protein HasE | | |
| K12538 | 1 | outer membrane protein HasF | | |
| K12541 | 3 | ATP-binding cassette, subfamily C, bacterial LapB | | |
| K12542 | 1 | membrane fusion protein LapC | | |
| K12543 | 1 | outer membrane protein LapE | | |
| K12544 | 1 | S-layer protein | | |
| K12549 | 12 | surface adhesion protein | | |
| K12555 | 1 | penicillin-binding protein 2A [EC:2.4.1.129 2.3.2.-] | | |
| K12573 | 53 | ribonuclease R [EC:3.1.-.-] | | |
| K12574 | 39 | ribonuclease J [EC:3.1.-.-] | | |
| K12583 | 6 | phosphatidylinositol alpha 1,6-mannosyltransferase [EC:2.4.1.-] | | |
| K12645 | 13 | epi-isozizaene 5-monooxygenase [EC:1.14.13.106] | | |
| K12658 | 5 | 4-hydroxyproline epimerase [EC:5.1.1.8] | | |
| K12660 | 5 | 2-dehydro-3-deoxy-L-rhamnonate aldolase [EC:4.1.2.-] | | |
| K12661 | 1 | L-rhamnonate dehydratase [EC:4.2.1.90] | | |
| K12673 | 2 | N2-(2-carboxyethyl)arginine synthase [EC:2.5.1.66] | | |
| K12686 | 2 | outer membrane lipase/esterase | | |
| K12688 | 2 | autotransporter serine protease [EC:3.4.21.-] | | |
| K12941 | 17 | aminobenzoyl-glutamate utilization protein B | | |
| K12942 | 5 | aminobenzoyl-glutamate transport protein | | |
| K12943 | 2 | lipoprotein YgeR | | |
| K12949 | 13 | cation-transporting P-type ATPase A/B [EC:3.6.3.-] | | |
| K12950 | 1 | cation-transporting P-type ATPase C [EC:3.6.3.-] | | |
| K12952 | 7 | cation-transporting ATPase E [EC:3.6.3.-] | | |

| | | | | |
|---|---|---|---|---|
| K12953 | 6 | cation-transporting ATPase F [EC:3.6.3.-] | | |
| K12956 | 10 | cation-transporting ATPase V [EC:3.6.3.-] | | |
| K12960 | 17 | 5-methylthioadenosine/S-adenosylhomocysteine deaminase | | |
| K12961 | 2 | DnaA initiator-associating protein | | |
| K12972 | 5 | gyoxylate/hydroxypyruvate reductase A [EC:1.1.1.79 1.1.1.81] | | |
| K12979 | 2 | beta-hydroxylase [EC:1.14.11.-] | | |
| K12982 | 2 | heptosyltransferase I [EC:2.4.-.-] | | |
| K12984 | 12 | (heptosyl)LPS beta-1,4-glucosyltransferase [EC:2.4.1.-] | | |
| K12987 | 3 | alpha-1,6-rhamnosyltransferase [EC:2.4.1.-] | | |
| K12988 | 2 | alpha-1,3-rhamnosyltransferase [EC:2.4.1.-] | | |
| K12989 | 2 | mannosyltransferase [EC:2.4.1.-] | | |
| K12990 | 3 | rhamnosyltransferase [EC:2.4.1.-] | | |
| K12992 | 1 | rhamnosyltransferase [EC:2.4.1.-] | | |
| K12994 | 6 | alpha-1,3-rhamnosyltransferase [EC:2.4.1.-] | | |
| K12995 | 3 | rhamnosyltransferase [EC:2.4.1.-] | | |
| K12996 | 4 | rhamnosyltransferase [EC:2.4.1.-] | | |
| K12997 | 1 | rhamnosyltransferase [EC:2.4.1.-] | | |
| K13001 | 4 | mannosyltransferase [EC:2.4.1.-] | | |
| K13002 | 3 | glycosyltransferase [EC:2.4.1.-] | | |
| K13004 | 5 | galacturonosyltransferase [EC:2.4.1.-] | | |
| K13006 | 4 | UDP-perosamine 4-acetyltransferase [EC:2.3.1.-] | | |
| K13007 | 1 | Fuc2NAc and GlcNAc transferase [EC:2.4.1.-] | | |
| K13010 | 16 | perosamine synthetase | | |
| K13013 | 17 | O-antigen biosynthesis protein WbqV | | |
| K13014 | 2 | undecaprenyl phosphate-alpha-L-ara4FN deformylase [EC:3.5.-.-] | | |
| K13015 | 28 | UDP-N-acetyl-D-glucosamine dehydrogenase [EC:1.1.1.-] | | |
| K13016 | 1 | UDP-D-GlcNAcA oxidase [EC:1.1.1.-] | | |
| K13017 | 16 | UDP-3-keto-D-GlcNAcA aminotransferase [EC:2.6.1.-] | | |
| K13018 | 4 | UDP-D-GlcNAc3NA acetyltransferase [EC:2.3.1.-] | | |
| K13019 | 9 | UDP-GlcNAc3NAcA epimerase [EC:5.1.3.23] | | |
| K13020 | 21 | UDP-D-GlcNAcA oxidase [EC:1.1.1.-] | | |
| K13021 | 6 | MFS transporter, ACS family, tartrate transporter | | |
| K13038 | 42 | phosphopantothenoylcysteine decarboxylase / | | |
| K13039 | 5 | sulfopyruvate decarboxylase subunit beta [EC:4.1.1.79] | | |
| K13041 | 1 | two-component system, LuxR family, response regulator TtrR | | |
| K13042 | 2 | dimethylglycine N-methyltransferase [EC:2.1.1.161] | | |
| K13043 | 3 | N-succinyl-L-ornithine transcarbamylase [EC:2.1.3.11] | | |
| K13049 | 48 | carboxypeptidase PM20D1 [EC:3.4.17.-] | | |
| K13051 | 19 | beta-aspartyl-peptidase (threonine type) [EC:3.4.19.5] | | |
| K13052 | 1 | cell division protein DivIC | | |
| K13057 | 14 | trehalose synthase [EC:2.4.1.245] | | |
| K13058 | 1 | mannosylfructose-phosphate synthase [EC:2.4.1.246] | | |
| K13063 | 1 | phenazine biosynthesis protein phzE [EC:2.6.1.86] | | |
| K13069 | 3 | diguanylate cyclase [EC:2.7.7.65] | | |
| K13074 | 8 | biflaviolin synthase [EC:1.14.21.7] | | |
| K13075 | 16 | N-acyl homoserine lactone hydrolase [EC:3.1.1.81] | | |
| K13237 | 3 | peroxisomal 2,4-dienoyl-CoA reductase [EC:1.3.1.34] | | |
| K13243 | 2 | c-di-GMP-specific phosphodiesterase [EC:3.1.4.52] | | |
| K13244 | 1 | c-di-GMP-specific phosphodiesterase [EC:3.1.4.52] | | |
| K13245 | 7 | c-di-GMP-specific phosphodiesterase [EC:3.1.4.52] | | |

| | | | | |
|---|---|---|---|---|
| K13252 | 1 | putrescine carbamoyltransferase [EC:2.1.3.6] | | |
| K13256 | 3 | protein PsiE | | |
| K13274 | 5 | cell wall-associated protease [EC:3.4.21.-] | | |
| K13275 | 2 | major intracellular serine protease [EC:3.4.21.-] | | |
| K13276 | 3 | bacillopeptidase F [EC:3.4.21.-] | | |
| K13277 | 3 | minor extracellular protease Epr [EC:3.4.21.-] | | |
| K13280 | 2 | signal peptidase, endoplasmic reticulum-type [EC:3.4.-.-] | | |
| K13281 | 2 | UV DNA damage endonuclease [EC:3.-.-.-] | | |
| K13282 | 2 | cyanophycinase [EC:3.4.15.6] | | |
| K13283 | 57 | ferrous-iron efflux pump FieF | | |
| K13285 | 1 | invasin B | | |
| K13288 | 10 | oligoribonuclease [EC:3.1.-.-] | | |
| K13292 | 27 | phosphatidylglycerol:prolipoprotein diacylglycerol transferase | | |
| K13317 | 3 | NDP-4-keto-2,6-dideoxyhexose 3-C-methyltransferase | | |
| K13318 | 6 | dTDP-4-keto-6-deoxy-L-hexose 4-reductase | | |
| K13378 | 29 | NADH dehydrogenase I subunit C/D [EC:1.6.5.3] | | |
| K13380 | 1 | NADH dehydrogenase I subunit B/C/D [EC:1.6.5.3] | | |
| K13408 | 2 | membrane fusion protein RaxA | | |
| K13409 | 1 | ATP-binding cassette, subfamily B, bacterial RaxB | | |
| K13419 | 13 | serine/threonine-protein kinase PknK [EC:2.7.11.1] | | |
| K13421 | 1 | uridine monophosphate synthetase [EC:2.4.2.10 4.1.1.23] | | |
| K13479 | 1 | xanthine dehydrogenase FAD-binding subunit [EC:1.17.1.4] | | |
| K13481 | 2 | xanthine dehydrogenase small subunit [EC:1.17.1.4] | | |
| K13482 | 10 | xanthine dehydrogenase large subunit [EC:1.17.1.4] | | |
| K13483 | 14 | xanthine dehydrogenase YagT iron-sulfur-binding subunit | | |
| K13486 | 2 | chemotaxis protein methyltransferase WspC | | |
| K13490 | 5 | two-component system, chemotaxis family, sensor histidine kinase and | | |
| K13491 | 1 | two-component system, chemotaxis family, response regulator WspF | | |
| K13497 | 7 | anthranilate synthase/phosphoribosyltransferase [EC:4.1.3.27 | | |
| K13498 | 7 | indole-3-glycerol phosphate synthase / phosphoribosylanthranilate | | |
| K13500 | 1 | chondroitin synthase [EC:2.4.1.175 2.4.1.226] | | |
| K13503 | 3 | anthranilate synthase [EC:4.1.3.27] | | |
| K13522 | 2 | bifunctional NMN adenylyltransferase/nudix hydrolase [EC:2.7.7.1 | | |
| K13525 | 23 | transitional endoplasmic reticulum ATPase | | |
| K13527 | 10 | proteasome-associated ATPase | | |
| K13529 | 16 | AraC family transcriptional regulator, regulatory protein of | | |
| K13530 | 4 | AraC family transcriptional regulator, regulatory protein of | | |
| K13531 | 1 | methylated-DNA-[protein]-cysteine S-methyltransferase [EC:2.1.1.63] | | |
| K13532 | 2 | two-component system, sporulation sensor kinase D [EC:2.7.13.3] | | |
| K13533 | 7 | two-component system, sporulation sensor kinase E [EC:2.7.13.3] | | |
| K13540 | 2 | precorrin-2 C20-methyltransferase / precorrin-3B | | |
| K13541 | 3 | cobalamin biosynthesis protein CbiG / precorrin-3B | | |
| K13542 | 25 | uroporphyrinogen III methyltransferase / synthase [EC:2.1.1.107 | | |
| K13543 | 6 | uroporphyrinogen III methyltransferase / synthase [EC:2.1.1.107 | | |
| K13571 | 4 | proteasome accessory factor A [EC:6.3.2.-] | | |
| K13572 | 7 | proteasome accessory factor B | | |
| K13573 | 11 | proteasome accessory factor C | | |
| K13574 | 10 | uncharacterized oxidoreductase [EC:1.1.1.-] | | |
| K13581 | 4 | modification methylase [EC:2.1.1.72] | | |
| K13582 | 2 | localization factor PodJL | | |

| | | | | |
|---|---|---|---|---|
| K13583 | 3 | GcrA cell cycle regulator | | |
| K13584 | 1 | two-component system, cell cycle response regulator CtrA | | |
| K13587 | 41 | two-component system, cell cycle sensor histidine kinase and | | |
| K13588 | 1 | histidine phosphotransferase ChpT | | |
| K13589 | 2 | two-component system, cell cycle response regulator CpdR | | |
| K13590 | 30 | diguanylate cyclase | | |
| K13592 | 1 | regulator of CtrA degradation | | |
| K13593 | 1 | cyclic-di-GMP phosphodiesterase, flagellum assembly factor TipF | | |
| K13598 | 35 | two-component system, NtrC family, nitrogen regulation sensor | | |
| K13599 | 57 | two-component system, NtrC family, nitrogen regulation response | | |
| K13601 | 26 | bacteriochlorophyll C8 methyltransferase [EC:2.1.1.-] | | |
| K13602 | 20 | bacteriochlorophyll C12 methyltransferase [EC:2.1.1.-] | | |
| K13604 | 7 | bacteriochlorophyll C20 methyltransferase [EC:2.1.1.-] | | |
| K13607 | 4 | cinnamoyl-CoA:phenyllactate CoA-transferase [EC:2.8.3.17] | | |
| K13611 | 6 | polyketide synthase PksJ | | |
| K13612 | 1 | polyketide synthase PksL | | |
| K13614 | 6 | polyketide synthase PksN | | |
| K13628 | 22 | iron-sulfur cluster assembly protein | | |
| K13633 | 20 | AraC family transcriptional regulator, transcriptional activator | | |
| K13634 | 7 | LysR family transcriptional regulator, cys regulon transcriptional | | |
| K13635 | 7 | LysR family transcriptional regulator, cys regulon transcriptional | | |
| K13637 | 1 | GntR family transcriptional regulator, uxuAB operon transcriptional | | |
| K13638 | 4 | MerR family transcriptional regulator, Zn(II)-responsive regulator | | |
| K13639 | 5 | MerR family transcriptional regulator, redox-sensitive | | |
| K13640 | 10 | MerR family transcriptional regulator, heat shock protein HspR | | |
| K13641 | 40 | IclR family transcriptional regulator, acetate operon repressor | | |
| K13642 | 7 | CRP/FNR family transcriptional regulator, transcriptional activator | | |
| K13643 | 24 | Rrf2 family transcriptional regulator, iron-sulfur cluster assembly | | |
| K13652 | 4 | AraC family transcriptional regulator | | |
| K13653 | 3 | AraC family transcriptional regulator | | |
| K13654 | 5 | GntR family transcriptional regulator, colanic acid and biofilm gene | | |
| K13660 | 1 | beta-1,4-glucosyltransferase [EC:2.4.1.-] | | |
| K13661 | 9 | GumC protein | | |
| K13668 | 21 | phosphatidylinositol alpha-1,6-mannosyltransferase [EC:2.4.1.-] | | |
| K13670 | 1 | alpha-1,6-mannosyltransferase [EC:2.4.1.-] | | |
| K13678 | 1 | monoglucosyldiacylglycerol glycosyltransferase [EC:2.4.1.-] | | |
| K13683 | 1 | putative colanic acid biosynthesis glycosyltransferase [EC:2.4.-.-] | | |
| K13684 | 1 | putative colanic acid biosynthesis glycosyltransferase [EC:2.4.-.-] | | |
| K13685 | 27 | UDP-N-acetylglucosamine:undecaprenyl-P N-acetylglucosaminyl 1-P | | |
| K13688 | 38 | cyclic beta-1,2-glucan synthetase [EC:2.4.1.-] | | |
| K13693 | 8 | glucosyl-3-phosphoglycerate synthase [EC:2.4.1.-] | | |
| K13694 | 3 | lipoprotein Spr | | |
| K13695 | 3 | probable lipoprotein NlpC | | |
| K13730 | 107 | internalin A | | |
| K13735 | 100 | adhesin/invasin | | |
| K13741 | 1 | guanine nucleotide exchange factor SopE | | |
| K13742 | 1 | protein IpgB1 | | |
| K13745 | 5 | L-2,4-diaminobutyrate decarboxylase [EC:4.1.1.86] | | |
| K13746 | 1 | carboxynorspermidine dehydrogenase | | |
| K13747 | 2 | carboxynorspermidine decarboxylase [EC:4.1.1.-] | | |

| | | | | |
|---|---|---|---|---|
| K13766 | 13 | methylglutaconyl-CoA hydratase [EC:4.2.1.18] | | |
| K13767 | 5 | enoyl-CoA hydratase [EC:4.2.1.17] | | |
| K13770 | 16 | TetR/AcrR family transcriptional regulator, fatty acid metabolism | | |
| K13771 | 2 | Rrf2 family transcriptional regulator, nitric oxide-sensitive | | |
| K13774 | 3 | citronellol/citronellal dehydrogenase | | |
| K13775 | 5 | citronellol/citronellal dehydrogenase | | |
| K13776 | 1 | citronellyl-CoA synthetase [EC:6.2.1.-] | | |
| K13777 | 1 | geranyl-CoA carboxylase alpha subunit [EC:6.4.1.5] | | |
| K13778 | 4 | geranyl-CoA carboxylase beta subunit [EC:6.4.1.5] | | |
| K13787 | 9 | geranylgeranyl diphosphate synthase, type I [EC:2.5.1.1 2.5.1.10 | | |
| K13788 | 5 | phosphate acetyltransferase [EC:2.3.1.8] | | |
| K13789 | 15 | geranylgeranyl diphosphate synthase, type II [EC:2.5.1.1 2.5.1.10 | | |
| K13794 | 1 | LysR family transcriptional regulator, regulatory protein for tcuABC | | |
| K13795 | 4 | citrate/tricarballylate utilization protein | | |
| K13796 | 11 | tricarballylate dehydrogenase | | |
| K13797 | 2 | DNA-directed RNA polymerase subunit beta-beta' [EC:2.7.7.6] | | |
| K13799 | 8 | pantoate ligase / cytidylate kinase [EC:6.3.2.1 2.7.4.14] | | |
| K13810 | 14 | transaldolase / glucose-6-phosphate isomerase [EC:2.2.1.2 5.3.1.9] | | |
| K13811 | 3 | 3'-phosphoadenosine 5'-phosphosulfate synthase [EC:2.7.7.4 2.7.1.25] | | |
| K13815 | 21 | two-component system, response regulator RpfG | | |
| K13816 | 7 | DSF synthase | | |
| K13819 | 3 | NifU-like protein | | |
| K13820 | 1 | flagellar biosynthetic protein FliR/FlhB | | |
| K13821 | 28 | proline dehydrogenase / delta 1-pyrroline-5-carboxylate | | |
| K13829 | 16 | shikimate kinase / 3-dehydroquinate synthase [EC:2.7.1.71 4.2.3.4] | | |
| K13832 | 9 | 3-dehydroquinate dehydratase / shikimate dehydrogenase [EC:4.2.1.10 | | |
| K13874 | 3 | L-arabinonolactonase [EC:3.1.1.15] | | |
| K13875 | 7 | L-arabonate dehydrase [EC:4.2.1.25] | | |
| K13876 | 5 | 2-keto-3-deoxy-L-arabinonate dehydratase [EC:4.2.1.43] | | |
| K13888 | 53 | macrolide-specific efflux protein MacA | | |
| K13889 | 13 | glutathione transport system substrate-binding protein | | |
| K13890 | 6 | glutathione transport system permease protein | | |
| K13891 | 2 | glutathione transport system permease protein | | |
| K13892 | 4 | glutathione transport system ATP-binding protein | | |
| K13893 | 9 | microcin C transport system substrate-binding protein | | |
| K13894 | 2 | microcin C transport system permease protein | | |
| K13895 | 4 | microcin C transport system permease protein | | |
| K13896 | 3 | microcin C transport system ATP-binding protein | | |
| K13919 | 1 | propanediol dehydratase medium subunit [EC:4.2.1.28] | | |
| K13922 | 1 | propionaldehyde dehydrogenase | | |
| K13924 | 124 | two-component system, chemotaxis family, CheB/CheR fusion protein | | |
| K13926 | 4 | ribosome-dependent ATPase | | |
| K13940 | 13 | dihydroneopterin aldolase / | | |
| K13950 | 9 | para-aminobenzoate synthetase [EC:2.6.1.85] | | |
| K13953 | 52 | alcohol dehydrogenase, propanol-preferring [EC:1.1.1.1] | | |
| K13954 | 5 | alcohol dehydrogenase [EC:1.1.1.1] | | |
| K13955 | 7 | zinc-binding alcohol dehydrogenase/oxidoreductase | | |
| K13963 | 11 | serpin B | | |
| K13979 | 9 | uncharacterized zinc-type alcohol dehydrogenase-like protein | | |
| K13990 | 19 | glutamate formiminotransferase / formiminotetrahydrofolate | | |

| | | | | |
|---|---|---|---|---|
| K13993 | 58 | HSP20 family protein | | |
| K13995 | 2 | maleamate amidohydrolase [EC:3.5.1.107] | | |

| KEGG ID | Sequences | KEGG pathway |
|---|---|---|
| K00001 | 181 | alcohol dehydrogenase [EC:1.1.1.1] |
| K00002 | 3 | alcohol dehydrogenase (NADP+) [EC:1.1.1.2] |
| K00003 | 74 | homoserine dehydrogenase [EC:1.1.1.3] |
| K00004 | 3 | (R,R)-butanediol dehydrogenase / diacetyl reductase [EC:1.1.1.4 |
| K00005 | 10 | glycerol dehydrogenase [EC:1.1.1.6] |
| K00008 | 37 | L-iditol 2-dehydrogenase [EC:1.1.1.14] |
| K00009 | 4 | mannitol-1-phosphate 5-dehydrogenase [EC:1.1.1.17] |
| K00010 | 96 | myo-inositol 2-dehydrogenase [EC:1.1.1.18] |
| K00011 | 4 | aldehyde reductase [EC:1.1.1.21] |
| K00012 | 45 | UDPglucose 6-dehydrogenase [EC:1.1.1.22] |
| K00013 | 43 | histidinol dehydrogenase [EC:1.1.1.23] |
| K00014 | 37 | shikimate dehydrogenase [EC:1.1.1.25] |
| K00015 | 39 | glyoxylate reductase [EC:1.1.1.26] |
| K00016 | 26 | L-lactate dehydrogenase [EC:1.1.1.27] |
| K00018 | 11 | glycerate dehydrogenase [EC:1.1.1.29] |
| K00019 | 33 | 3-hydroxybutyrate dehydrogenase [EC:1.1.1.30] |
| K00020 | 92 | 3-hydroxyisobutyrate dehydrogenase [EC:1.1.1.31] |
| K00021 | 14 | 3-hydroxy-3-methylglutaryl-CoA reductase [EC:1.1.1.34] |
| K00023 | 24 | acetoacetyl-CoA reductase [EC:1.1.1.36] |
| K00024 | 35 | malate dehydrogenase [EC:1.1.1.37] |
| K00027 | 40 | malate dehydrogenase (oxaloacetate-decarboxylating) [EC:1.1.1.38] |
| K00028 | 4 | malate dehydrogenase (decarboxylating) [EC:1.1.1.39] |
| K00029 | 39 | malate dehydrogenase (oxaloacetate-decarboxylating)(NADP+) |
| K00030 | 19 | isocitrate dehydrogenase (NAD+) [EC:1.1.1.41] |
| K00031 | 37 | isocitrate dehydrogenase [EC:1.1.1.42] |
| K00032 | 1 | phosphogluconate 2-dehydrogenase [EC:1.1.1.43] |
| K00033 | 37 | 6-phosphogluconate dehydrogenase [EC:1.1.1.44] |
| K00034 | 25 | glucose 1-dehydrogenase [EC:1.1.1.47] |
| K00035 | 1 | D-galactose 1-dehydrogenase [EC:1.1.1.48] |
| K00036 | 118 | glucose-6-phosphate 1-dehydrogenase [EC:1.1.1.49] |
| K00037 | 2 | 3-alpha-hydroxysteroid dehydrogenase [EC:1.1.1.50] |
| K00038 | 6 | 3alpha(or 20beta)-hydroxysteroid dehydrogenase [EC:1.1.1.53] |
| K00040 | 4 | fructuronate reductase [EC:1.1.1.57] |
| K00042 | 60 | 2-hydroxy-3-oxopropionate reductase [EC:1.1.1.60] |
| K00043 | 6 | 4-hydroxybutyrate dehydrogenase [EC:1.1.1.61] |
| K00044 | 10 | estradiol 17beta-dehydrogenase [EC:1.1.1.62] |
| K00045 | 4 | mannitol 2-dehydrogenase [EC:1.1.1.67] |
| K00046 | 33 | gluconate 5-dehydrogenase [EC:1.1.1.69] |
| K00048 | 20 | lactaldehyde reductase [EC:1.1.1.77] |
| K00050 | 46 | hydroxypyruvate reductase [EC:1.1.1.81] |
| K00051 | 10 | malate dehydrogenase (NADP+) [EC:1.1.1.82] |
| K00052 | 66 | 3-isopropylmalate dehydrogenase [EC:1.1.1.85] |
| K00053 | 17 | ketol-acid reductoisomerase [EC:1.1.1.86] |
| K00054 | 7 | hydroxymethylglutaryl-CoA reductase [EC:1.1.1.88] |
| K00055 | 2 | aryl-alcohol dehydrogenase [EC:1.1.1.90] |
| K00057 | 38 | glycerol-3-phosphate dehydrogenase (NAD(P)+) [EC:1.1.1.94] |
| K00058 | 145 | D-3-phosphoglycerate dehydrogenase [EC:1.1.1.95] |
| K00059 | 312 | 3-oxoacyl-[acyl-carrier protein] reductase [EC:1.1.1.100] |
| K00060 | 50 | threonine 3-dehydrogenase [EC:1.1.1.103] |
| K00064 | 7 | D-threo-aldose 1-dehydrogenase [EC:1.1.1.122] |
| K00065 | 29 | 2-deoxy-D-gluconate 3-dehydrogenase [EC:1.1.1.125] |
| K00066 | 25 | GDP-mannose 6-dehydrogenase [EC:1.1.1.132] |
| K00067 | 36 | dTDP-4-dehydrorhamnose reductase [EC:1.1.1.133] |
| K00068 | 7 | sorbitol-6-phosphate 2-dehydrogenase [EC:1.1.1.140] |
| K00071 | 3 | 11beta-hydroxysteroid dehydrogenase [EC:1.1.1.146] |
| K00073 | 35 | ureidoglycolate dehydrogenase [EC:1.1.1.154] |
| K00074 | 63 | 3-hydroxybutyryl-CoA dehydrogenase [EC:1.1.1.157] |
| K00075 | 37 | UDP-N-acetylmuramate dehydrogenase [EC:1.1.1.158] |

| | | | | |
|---|---|---|---|---|
| K00076 | 5 | 7-alpha-hydroxysteroid dehydrogenase [EC:1.1.1.159] | | |
| K00077 | 44 | 2-dehydropantoate 2-reductase [EC:1.1.1.169] | | |
| K00079 | 12 | carbonyl reductase (NADPH) [EC:1.1.1.184] | | |
| K00082 | 9 | 5-amino-6-(5-phosphoribosylamino)uracil reductase [EC:1.1.1.193] | | |
| K00083 | 2 | cinnamyl-alcohol dehydrogenase [EC:1.1.1.195] | | |
| K00086 | 5 | 1,3-propanediol dehydrogenase [EC:1.1.1.202] | | |
| K00087 | 105 | xanthine dehydrogenase molybdenum-binding subunit [EC:1.17.1.4] | | |
| K00088 | 84 | IMP dehydrogenase [EC:1.1.1.205] | | |
| K00090 | 13 | gluconate 2-dehydrogenase [EC:1.1.1.215] | | |
| K00091 | 22 | dihydroflavonol-4-reductase [EC:1.1.1.219] | | |
| K00094 | 1 | galactitol-1-phosphate 5-dehydrogenase [EC:1.1.1.251] | | |
| K00096 | 3 | glycerol-1-phosphate dehydrogenase [NAD(P)] [EC:1.1.1.261] | | |
| K00097 | 42 | 4-hydroxythreonine-4-phosphate dehydrogenase [EC:1.1.1.262] | | |
| K00098 | 4 | L-idonate 5-dehydrogenase [EC:1.1.1.264] | | |
| K00099 | 51 | 1-deoxy-D-xylulose-5-phosphate reductoisomerase [EC:1.1.1.267] | | |
| K00100 | 439 | Unclassified; E1.1.1.- | | |
| K00101 | 50 | L-lactate dehydrogenase (cytochrome) [EC:1.1.2.3] | | |
| K00102 | 85 | D-lactate dehydrogenase (cytochrome) [EC:1.1.2.4] | | |
| K00103 | 10 | L-gulonolactone oxidase [EC:1.1.3.8] | | |
| K00104 | 91 | glycolate oxidase [EC:1.1.3.15] | | |
| K00105 | 3 | alpha-glycerophosphate oxidase [EC:1.1.3.21] | | |
| K00108 | 51 | choline dehydrogenase [EC:1.1.99.1] | | |
| K00109 | 14 | 2-hydroxyglutarate dehydrogenase [EC:1.1.99.2] | | |
| K00111 | 53 | glycerol-3-phosphate dehydrogenase [EC:1.1.5.3] | | |
| K00112 | 2 | glycerol-3-phosphate dehydrogenase subunit B [EC:1.1.5.3] | | |
| K00113 | 10 | glycerol-3-phosphate dehydrogenase subunit C [EC:1.1.5.3] | | |
| K00114 | 80 | alcohol dehydrogenase (acceptor) [EC:1.1.99.8] | | |
| K00116 | 24 | malate dehydrogenase (quinone) [EC:1.1.5.4] | | |
| K00117 | 55 | quinoprotein glucose dehydrogenase [EC:1.1.5.2] | | |
| K00118 | 12 | glucose-fructose oxidoreductase [EC:1.1.99.28] | | |
| K00119 | 103 | Unclassified; E1.1.99.- | | |
| K00120 | 82 | Unclassified; E1.1.-.- | | |
| K00121 | 49 | S-(hydroxymethyl)glutathione dehydrogenase / alcohol dehydrogenase | | |
| K00122 | 100 | formate dehydrogenase [EC:1.2.1.2] | | |
| K00123 | 164 | formate dehydrogenase, alpha subunit [EC:1.2.1.2] | | |
| K00124 | 24 | formate dehydrogenase, beta subunit [EC:1.2.1.2] | | |
| K00127 | 21 | formate dehydrogenase, gamma subunit [EC:1.2.1.2] | | |
| K00128 | 152 | aldehyde dehydrogenase (NAD+) [EC:1.2.1.3] | | |
| K00129 | 2 | aldehyde dehydrogenase (NAD(P)+) [EC:1.2.1.5] | | |
| K00130 | 60 | betaine-aldehyde dehydrogenase [EC:1.2.1.8] | | |
| K00131 | 21 | glyceraldehyde-3-phosphate dehydrogenase (NADP) [EC:1.2.1.9] | | |
| K00132 | 6 | acetaldehyde dehydrogenase (acetylating) [EC:1.2.1.10] | | |
| K00133 | 35 | aspartate-semialdehyde dehydrogenase [EC:1.2.1.11] | | |
| K00134 | 29 | glyceraldehyde 3-phosphate dehydrogenase [EC:1.2.1.12] | | |
| K00135 | 94 | succinate-semialdehyde dehydrogenase (NADP+) [EC:1.2.1.16] | | |
| K00137 | 5 | aminobutyraldehyde dehydrogenase [EC:1.2.1.19] | | |
| K00138 | 6 | aldehyde dehydrogenase [EC:1.2.1.-] | | |
| K00140 | 32 | methylmalonate-semialdehyde dehydrogenase [EC:1.2.1.27] | | |
| K00141 | 6 | benzaldehyde dehydrogenase (NAD) [EC:1.2.1.28] | | |
| K00145 | 50 | N-acetyl-gamma-glutamyl-phosphate reductase [EC:1.2.1.38] | | |
| K00146 | 17 | phenylacetaldehyde dehydrogenase [EC:1.2.1.39] | | |
| K00147 | 25 | glutamate-5-semialdehyde dehydrogenase [EC:1.2.1.41] | | |
| K00148 | 5 | glutathione-independent formaldehyde dehydrogenase [EC:1.2.1.46] | | |
| K00150 | 2 | glyceraldehyde-3-phosphate dehydrogenase (NAD(P)) [EC:1.2.1.59] | | |
| K00151 | 15 | 5-carboxymethyl-2-hydroxymuconic-semialdehyde dehydrogenase | | |
| K00152 | 5 | salicylaldehyde dehydrogenase [EC:1.2.1.65] | | |
| K00153 | 7 | NAD/factor-dependent formaldehyde dehydrogenase [EC:1.2.1.66] | | |
| K00154 | 3 | coniferyl-aldehyde dehydrogenase [EC:1.2.1.68] | | |
| K00155 | 81 | Unclassified; E1.2.1.- | | |

| | | | | |
|---|---|---|---|---|
| K00156 | 27 | pyruvate dehydrogenase (cytochrome) [EC:1.2.2.2] | | |
| K00157 | 22 | aldehyde oxidase [EC:1.2.3.1] | | |
| K00158 | 6 | pyruvate oxidase [EC:1.2.3.3] | | |
| K00161 | 65 | pyruvate dehydrogenase E1 component subunit alpha [EC:1.2.4.1] | | |
| K00162 | 66 | pyruvate dehydrogenase E1 component subunit beta [EC:1.2.4.1] | | |
| K00163 | 45 | pyruvate dehydrogenase E1 component [EC:1.2.4.1] | | |
| K00164 | 41 | 2-oxoglutarate dehydrogenase E1 component [EC:1.2.4.2] | | |
| K00166 | 32 | 2-oxoisovalerate dehydrogenase E1 component, alpha subunit | | |
| K00167 | 31 | 2-oxoisovalerate dehydrogenase E1 component, beta subunit | | |
| K00169 | 37 | pyruvate ferredoxin oxidoreductase, alpha subunit [EC:1.2.7.1] | | |
| K00170 | 17 | pyruvate ferredoxin oxidoreductase, beta subunit [EC:1.2.7.1] | | |
| K00171 | 15 | pyruvate ferredoxin oxidoreductase, delta subunit [EC:1.2.7.1] | | |
| K00172 | 16 | pyruvate ferredoxin oxidoreductase, gamma subunit [EC:1.2.7.1] | | |
| K00174 | 70 | 2-oxoglutarate ferredoxin oxidoreductase subunit alpha [EC:1.2.7.3] | | |
| K00175 | 41 | 2-oxoglutarate ferredoxin oxidoreductase subunit beta [EC:1.2.7.3] | | |
| K00176 | 10 | 2-oxoglutarate ferredoxin oxidoreductase subunit delta [EC:1.2.7.3] | | |
| K00177 | 12 | 2-oxoglutarate ferredoxin oxidoreductase subunit gamma [EC:1.2.7.3] | | |
| K00178 | 23 | oxidoreductase containing iron-sulfur protein [EC:1.2.7.-] | | |
| K00179 | 43 | indolepyruvate ferredoxin oxidoreductase, alpha subunit [EC:1.2.7.8] | | |
| K00180 | 23 | indolepyruvate ferredoxin oxidoreductase, beta subunit [EC:1.2.7.8] | | |
| K00183 | 52 | molybdopterin oxidoreductase, molybdopterin binding subunit | | |
| K00184 | 53 | molybdopterin oxidoreductase, iron-sulfur binding subunit | | |
| K00185 | 44 | molybdopterin oxidoreductase, membrane subunit [EC:1.2.7.-] | | |
| K00186 | 10 | 2-oxoisovalerate ferredoxin oxidoreductase, alpha subunit | | |
| K00187 | 7 | 2-oxoisovalerate ferredoxin oxidoreductase, beta subunit | | |
| K00191 | 7 | CO-methylating acetyl-CoA synthase [EC:2.3.1.169] | | |
| K00194 | 1 | carbon-monoxide dehydrogenase delta subunit [EC:1.2.99.2] | | |
| K00197 | 5 | carbon-monoxide dehydrogenase gamma subunit [EC:1.2.99.2] | | |
| K00198 | 3 | carbon-monoxide dehydrogenase catalytic subunit [EC:1.2.99.2] | | |
| K00200 | 8 | formylmethanofuran dehydrogenase subunit A [EC:1.2.99.5] | | |
| K00201 | 1 | formylmethanofuran dehydrogenase subunit B [EC:1.2.99.5] | | |
| K00202 | 2 | formylmethanofuran dehydrogenase subunit C [EC:1.2.99.5] | | |
| K00206 | 1 | Unclassified; E1.2.-.- | | |
| K00207 | 10 | dihydropyrimidine dehydrogenase (NADP+) [EC:1.3.1.2] | | |
| K00208 | 71 | enoyl-[acyl-carrier protein] reductase I [EC:1.3.1.9] | | |
| K00209 | 3 | enoyl-[acyl-carrier-protein] reductase (NADPH2, B-specific) | | |
| K00210 | 23 | prephenate dehydrogenase [EC:1.3.1.12] | | |
| K00214 | 1 | biliverdin reductase [EC:1.3.1.24] | | |
| K00215 | 29 | dihydrodipicolinate reductase [EC:1.3.1.26] | | |
| K00216 | 2 | 2,3-dihydro-2,3-dihydroxybenzoate dehydrogenase [EC:1.3.1.28] | | |
| K00217 | 4 | maleylacetate reductase [EC:1.3.1.32] | | |
| K00218 | 9 | protochlorophyllide reductase [EC:1.3.1.33] | | |
| K00219 | 58 | 2,4-dienoyl-CoA reductase (NADPH2) [EC:1.3.1.34] | | |
| K00220 | 4 | cyclohexadienyl dehydrogenase [EC:1.3.1.43] | | |
| K00224 | 11 | Unclassified; E1.3.1.- | | |
| K00226 | 72 | dihydroorotate oxidase [EC:1.3.3.1] | | |
| K00227 | 3 | lathosterol oxidase [EC:1.14.21.6] | | |
| K00228 | 17 | coproporphyrinogen III oxidase [EC:1.3.3.3] | | |
| K00230 | 8 | protoporphyrinogen oxidase [EC:1.3.3.4] | | |
| K00231 | 27 | protoporphyrinogen oxidase [EC:1.3.3.4] | | |
| K00232 | 1 | acyl-CoA oxidase [EC:1.3.3.6] | | |
| K00239 | 70 | succinate dehydrogenase flavoprotein subunit [EC:1.3.99.1] | | |
| K00240 | 31 | succinate dehydrogenase iron-sulfur protein [EC:1.3.99.1] | | |
| K00241 | 21 | succinate dehydrogenase cytochrome b-556 subunit [EC:1.3.99.1] | | |
| K00242 | 5 | succinate dehydrogenase hydrophobic membrane anchor protein | | |
| K00244 | 39 | fumarate reductase flavoprotein subunit [EC:1.3.99.1] | | |
| K00245 | 7 | fumarate reductase iron-sulfur protein [EC:1.3.99.1] | | |
| K00246 | 13 | fumarate reductase subunit C [EC:1.3.99.1] | | |
| K00247 | 5 | fumarate reductase subunit D [EC:1.3.99.1] | | |

| | | | | |
|---|---|---|---|---|
| K00248 | 107 | butyryl-CoA dehydrogenase [EC:1.3.99.2] | | |
| K00249 | 223 | acyl-CoA dehydrogenase [EC:1.3.99.3] | | |
| K00252 | 32 | glutaryl-CoA dehydrogenase [EC:1.3.99.7] | | |
| K00253 | 67 | isovaleryl-CoA dehydrogenase [EC:1.3.99.10] | | |
| K00255 | 14 | long-chain-acyl-CoA dehydrogenase [EC:1.3.99.13] | | |
| K00256 | 109 | isoquinoline 1-oxidoreductase [EC:1.3.99.16] | | |
| K00257 | 333 | Unclassified; E1.3.99.- | | |
| K00258 | 29 | Unclassified; E1.3.-.- | | |
| K00259 | 29 | alanine dehydrogenase [EC:1.4.1.1] | | |
| K00260 | 63 | glutamate dehydrogenase [EC:1.4.1.2] | | |
| K00261 | 38 | glutamate dehydrogenase (NAD(P)+) [EC:1.4.1.3] | | |
| K00262 | 26 | glutamate dehydrogenase (NADP+) [EC:1.4.1.4] | | |
| K00263 | 17 | leucine dehydrogenase [EC:1.4.1.9] | | |
| K00265 | 89 | glutamate synthase (NADPH/NADH) large chain [EC:1.4.1.13 1.4.1.14] | | |
| K00266 | 101 | glutamate synthase (NADPH/NADH) small chain [EC:1.4.1.13 1.4.1.14] | | |
| K00271 | 14 | valine dehydrogenase [EC:1.4.1.-] | | |
| K00273 | 15 | D-amino-acid oxidase [EC:1.4.3.3] | | |
| K00274 | 21 | monoamine oxidase [EC:1.4.3.4] | | |
| K00275 | 18 | pyridoxamine 5'-phosphate oxidase [EC:1.4.3.5] | | |
| K00276 | 60 | primary-amine oxidase [EC:1.4.3.21] | | |
| K00278 | 44 | L-aspartate oxidase [EC:1.4.3.16] | | |
| K00279 | 15 | cytokinin dehydrogenase [EC:1.5.99.12] | | |
| K00280 | 8 | Unclassified; E1.4.3.- | | |
| K00281 | 24 | glycine dehydrogenase [EC:1.4.4.2] | | |
| K00282 | 30 | glycine dehydrogenase subunit 1 [EC:1.4.4.2] | | |
| K00283 | 25 | glycine dehydrogenase subunit 2 [EC:1.4.4.2] | | |
| K00284 | 28 | glutamate synthase (ferredoxin) [EC:1.4.7.1] | | |
| K00285 | 34 | D-amino-acid dehydrogenase [EC:1.4.99.1] | | |
| K00286 | 27 | pyrroline-5-carboxylate reductase [EC:1.5.1.2] | | |
| K00287 | 56 | dihydrofolate reductase [EC:1.5.1.3] | | |
| K00290 | 30 | saccharopine dehydrogenase (NAD+, L-lysine forming) [EC:1.5.1.7] | | |
| K00292 | 2 | saccharopine dehydrogenase (NAD+, L-glutamate forming) [EC:1.5.1.9] | | |
| K00294 | 69 | 1-pyrroline-5-carboxylate dehydrogenase [EC:1.5.1.12] | | |
| K00297 | 67 | methylenetetrahydrofolate reductase (NADPH) [EC:1.5.1.20] | | |
| K00299 | 34 | FMN reductase [EC:1.5.1.29] | | |
| K00301 | 35 | sarcosine oxidase [EC:1.5.3.1] | | |
| K00302 | 11 | sarcosine oxidase, subunit alpha [EC:1.5.3.1] | | |
| K00303 | 37 | sarcosine oxidase, subunit beta [EC:1.5.3.1] | | |
| K00304 | 2 | sarcosine oxidase, subunit delta [EC:1.5.3.1] | | |
| K00305 | 1 | sarcosine oxidase, subunit gamma [EC:1.5.3.1] | | |
| K00309 | 57 | Unclassified; E1.5.3.- | | |
| K00311 | 35 | electron-transferring-flavoprotein dehydrogenase [EC:1.5.5.1] | | |
| K00313 | 5 | electron transfer flavoprotein-quinone oxidoreductase [EC:1.5.5.-] | | |
| K00314 | 44 | sarcosine dehydrogenase [EC:1.5.99.1] | | |
| K00315 | 21 | dimethylglycine dehydrogenase [EC:1.5.99.2] | | |
| K00317 | 11 | trimethylamine dehydrogenase [EC:1.5.8.2] | | |
| K00318 | 21 | proline dehydrogenase [EC:1.5.99.8] | | |
| K00320 | 25 | coenzyme F420-dependent N5,N10-methenyltetrahydromethanopterin | | |
| K00321 | 1 | Unclassified; E1.5.99.- | | |
| K00322 | 12 | NAD(P) transhydrogenase [EC:1.6.1.1] | | |
| K00324 | 34 | NAD(P) transhydrogenase subunit alpha [EC:1.6.1.2] | | |
| K00325 | 18 | NAD(P) transhydrogenase subunit beta [EC:1.6.1.2] | | |
| K00326 | 1 | cytochrome-b5 reductase [EC:1.6.2.2] | | |
| K00327 | 1 | NADPH-ferrihemoprotein reductase [EC:1.6.2.4] | | |
| K00329 | 36 | NADH dehydrogenase [EC:1.6.5.3] | | |
| K00330 | 25 | NADH dehydrogenase I subunit A [EC:1.6.5.3] | | |
| K00331 | 27 | NADH dehydrogenase I subunit B [EC:1.6.5.3] | | |
| K00332 | 21 | NADH dehydrogenase I subunit C [EC:1.6.5.3] | | |
| K00333 | 64 | NADH dehydrogenase I subunit D [EC:1.6.5.3] | | |

| | | | | |
|---|---|---|---|---|
| K00334 | 43 | NADH dehydrogenase I subunit E [EC:1.6.5.3] | | |
| K00335 | 80 | NADH dehydrogenase I subunit F [EC:1.6.5.3] | | |
| K00336 | 63 | NADH dehydrogenase I subunit G [EC:1.6.5.3] | | |
| K00337 | 55 | NADH dehydrogenase I subunit H [EC:1.6.5.3] | | |
| K00338 | 26 | NADH dehydrogenase I subunit I [EC:1.6.5.3] | | |
| K00339 | 20 | NADH dehydrogenase I subunit J [EC:1.6.5.3] | | |
| K00340 | 15 | NADH dehydrogenase I subunit K [EC:1.6.5.3] | | |
| K00341 | 57 | NADH dehydrogenase I subunit L [EC:1.6.5.3] | | |
| K00342 | 63 | NADH dehydrogenase I subunit M [EC:1.6.5.3] | | |
| K00343 | 62 | NADH dehydrogenase I subunit N [EC:1.6.5.3] | | |
| K00344 | 170 | NADPH2:quinone reductase [EC:1.6.5.5] | | |
| K00346 | 2 | Na+-transporting NADH:ubiquinone oxidoreductase subunit A | | |
| K00347 | 10 | Na+-transporting NADH:ubiquinone oxidoreductase subunit B | | |
| K00348 | 7 | Na+-transporting NADH:ubiquinone oxidoreductase subunit C | | |
| K00349 | 2 | Na+-transporting NADH:ubiquinone oxidoreductase subunit D | | |
| K00350 | 9 | Na+-transporting NADH:ubiquinone oxidoreductase subunit E | | |
| K00351 | 4 | Na+-transporting NADH:ubiquinone oxidoreductase subunit F | | |
| K00353 | 1 | Unclassified; E1.6.6.- | | |
| K00354 | 18 | NADPH2 dehydrogenase [EC:1.6.99.1] | | |
| K00355 | 4 | NAD(P)H dehydrogenase (quinone) [EC:1.6.5.2] | | |
| K00356 | 59 | NADH dehydrogenase [EC:1.6.99.3] | | |
| K00358 | 10 | Unclassified; E1.6.99.- | | |
| K00359 | 38 | NADH oxidase [EC:1.6.-.-] | | |
| K00360 | 39 | nitrate reductase (NADH) [EC:1.7.1.1] | | |
| K00362 | 26 | nitrite reductase (NAD(P)H) large subunit [EC:1.7.1.4] | | |
| K00363 | 13 | nitrite reductase (NAD(P)H) small subunit [EC:1.7.1.4] | | |
| K00364 | 4 | GMP reductase [EC:1.7.1.7] | | |
| K00365 | 9 | urate oxidase [EC:1.7.3.3] | | |
| K00366 | 23 | ferredoxin-nitrite reductase [EC:1.7.7.1] | | |
| K00367 | 5 | ferredoxin-nitrate reductase [EC:1.7.7.2] | | |
| K00368 | 53 | nitrite reductase (NO-forming) [EC:1.7.2.1] | | |
| K00369 | 45 | nitrate reductase [EC:1.7.99.4] | | |
| K00370 | 34 | nitrate reductase 1, alpha subunit [EC:1.7.99.4] | | |
| K00371 | 12 | nitrate reductase 1, beta subunit [EC:1.7.99.4] | | |
| K00372 | 36 | nitrate reductase catalytic subunit [EC:1.7.99.4] | | |
| K00373 | 4 | nitrate reductase 1, delta subunit [EC:1.7.99.4] | | |
| K00374 | 4 | nitrate reductase 1, gamma subunit [EC:1.7.99.4] | | |
| K00375 | 33 | GntR family transcriptional regulator / MocR family aminotransferase | | |
| K00376 | 14 | nitrous-oxide reductase [EC:1.7.99.6] | | |
| K00378 | 3 | hydroxylamine reductase [EC:1.7.-.-] | | |
| K00380 | 19 | sulfite reductase (NADPH) flavoprotein alpha-component [EC:1.8.1.2] | | |
| K00381 | 24 | sulfite reductase (NADPH) hemoprotein beta-component [EC:1.8.1.2] | | |
| K00382 | 97 | dihydrolipoamide dehydrogenase [EC:1.8.1.4] | | |
| K00383 | 19 | glutathione reductase (NADPH) [EC:1.8.1.7] | | |
| K00384 | 111 | thioredoxin reductase (NADPH) [EC:1.8.1.9] | | |
| K00385 | 1 | anaerobic sulfite reductase subunit C [EC:1.8.1.-] | | |
| K00386 | 20 | Unclassified; E1.8.2.- | | |
| K00387 | 22 | sulfite oxidase [EC:1.8.3.1] | | |
| K00389 | 9 | putative membrane protein | | |
| K00390 | 17 | phosphoadenosine phosphosulfate reductase [EC:1.8.4.8] | | |
| K00391 | 5 | Unclassified; E1.8.4.- | | |
| K00392 | 14 | sulfite reductase (ferredoxin) [EC:1.8.7.1] | | |
| K00394 | 11 | adenylylsulfate reductase, subunit A [EC:1.8.99.2] | | |
| K00395 | 3 | adenylylsulfate reductase, subunit B [EC:1.8.99.2] | | |
| K00397 | 12 | anaerobic dimethyl sulfoxide reductase [EC:1.8.99.-] | | |
| K00404 | 16 | cb-type cytochrome c oxidase subunit I [EC:1.9.3.1] | | |
| K00405 | 17 | cb-type cytochrome c oxidase subunit II [EC:1.9.3.1] | | |
| K00406 | 34 | cb-type cytochrome c oxidase subunit III [EC:1.9.3.1] | | |
| K00407 | 6 | cb-type cytochrome c oxidase subunit IV [EC:1.9.3.1] | | |

| | | | | |
|---|---|---|---|---|
| K00411 | 10 | ubiquinol-cytochrome c reductase iron-sulfur subunit [EC:1.10.2.2] | | |
| K00412 | 35 | ubiquinol-cytochrome c reductase cytochrome b subunit [EC:1.10.2.2] | | |
| K00413 | 8 | ubiquinol-cytochrome c reductase cytochrome c1 subunit [EC:1.10.2.2] | | |
| K00421 | 4 | Unclassified; E1.10.2.- | | |
| K00423 | 22 | L-ascorbate oxidase [EC:1.10.3.3] | | |
| K00425 | 39 | cytochrome bd-I oxidase subunit I [EC:1.10.3.-] | | |
| K00426 | 23 | cytochrome bd-I oxidase subunit II [EC:1.10.3.-] | | |
| K00427 | 2 | L-lactate permease | | |
| K00428 | 51 | cytochrome c peroxidase [EC:1.11.1.5] | | |
| K00429 | 1 | catalase [EC:1.11.1.6] | | |
| K00432 | 7 | glutathione peroxidase [EC:1.11.1.9] | | |
| K00433 | 36 | chloride peroxidase [EC:1.11.1.10] | | |
| K00435 | 19 | peroxiredoxin [EC:1.11.1.-] | | |
| K00436 | 91 | hydrogen dehydrogenase [EC:1.12.1.2] | | |
| K00437 | 8 | cytochrome-c3 hydrogenase [EC:1.12.2.1] | | |
| K00441 | 4 | coenzyme F420 hydrogenase beta subunit [EC:1.12.98.1] | | |
| K00442 | 3 | coenzyme F420 hydrogenase delta subunit | | |
| K00446 | 7 | catechol 2,3-dioxygenase [EC:1.13.11.2] | | |
| K00448 | 10 | protocatechuate 3,4-dioxygenase, alpha subunit [EC:1.13.11.3] | | |
| K00449 | 6 | protocatechuate 3,4-dioxygenase, beta subunit [EC:1.13.11.3] | | |
| K00450 | 24 | gentisate 1,2-dioxygenase [EC:1.13.11.4] | | |
| K00451 | 27 | homogentisate 1,2-dioxygenase [EC:1.13.11.5] | | |
| K00453 | 11 | tryptophan 2,3-dioxygenase [EC:1.13.11.11] | | |
| K00455 | 1 | 3,4-dihydroxyphenylacetate 2,3-dioxygenase [EC:1.13.11.15] | | |
| K00457 | 19 | 4-hydroxyphenylpyruvate dioxygenase [EC:1.13.11.27] | | |
| K00459 | 54 | nitronate monooxygenase [EC:1.13.12.16] | | |
| K00462 | 4 | biphenyl-2,3-diol 1,2-dioxygenase [EC:1.13.11.39] | | |
| K00464 | 2 | lignostilbene-alpha,beta-dioxygenase [EC:1.13.11.43] | | |
| K00465 | 24 | Unclassified; E1.13.11.- | | |
| K00466 | 5 | tryptophan 2-monooxygenase [EC:1.13.12.3] | | |
| K00467 | 12 | lactate 2-monooxygenase [EC:1.13.12.4] | | |
| K00470 | 8 | Unclassified; E1.14.1.- | | |
| K00476 | 1 | aspartate beta-hydroxylase [EC:1.14.11.16] | | |
| K00477 | 2 | phytanoyl-CoA hydroxylase [EC:1.14.11.18] | | |
| K00478 | 6 | Unclassified; E1.14.11.- | | |
| K00479 | 6 | Rieske 2Fe-2S family protein | | |
| K00480 | 28 | salicylate hydroxylase [EC:1.14.13.1] | | |
| K00481 | 6 | p-hydroxybenzoate 3-monooxygenase [EC:1.14.13.2] | | |
| K00483 | 31 | 4-hydroxyphenylacetate-3-hydroxylase large chain [EC:1.14.13.3] | | |
| K00484 | 2 | 4-hydroxyphenylacetate-3-hydroxylase small chain [EC:1.14.13.3] | | |
| K00485 | 11 | dimethylaniline monooxygenase (N-oxide forming) [EC:1.14.13.8] | | |
| K00491 | 2 | nitric-oxide synthase, bacterial [EC:1.14.13.39] | | |
| K00492 | 81 | Unclassified; E1.14.13.- | | |
| K00493 | 46 | unspecific monooxygenase [EC:1.14.14.1] | | |
| K00494 | 70 | alkanal monooxygenase (FMN-linked) [EC:1.14.14.3] | | |
| K00495 | 10 | Unclassified; E1.14.14.- | | |
| K00499 | 10 | choline monooxygenase [EC:1.14.15.7] | | |
| K00500 | 17 | phenylalanine-4-hydroxylase [EC:1.14.16.1] | | |
| K00504 | 76 | peptidylglycine monooxygenase [EC:1.14.17.3] | | |
| K00507 | 40 | stearoyl-CoA desaturase (delta-9 desaturase) [EC:1.14.19.1] | | |
| K00508 | 4 | linoleoyl-CoA desaturase [EC:1.14.19.3] | | |
| K00514 | 1 | zeta-carotene desaturase [EC:1.14.99.30] | | |
| K00517 | 179 | Unclassified; E1.14.-.- | | |
| K00520 | 60 | mercuric reductase [EC:1.16.1.1] | | |
| K00523 | 25 | CDP-4-dehydro-6-deoxyglucose reductase [EC:1.17.1.1] | | |
| K00525 | 87 | ribonucleoside-diphosphate reductase alpha chain [EC:1.17.4.1] | | |
| K00526 | 14 | ribonucleoside-diphosphate reductase beta chain [EC:1.17.4.1] | | |
| K00527 | 5 | ribonucleoside-triphosphate reductase [EC:1.17.4.2] | | |
| K00528 | 42 | ferredoxin--NADP+ reductase [EC:1.18.1.2] | | |

| | | | | |
|---|---|---|---|---|
| K00529 | 61 | ferredoxin--NAD+ reductase [EC:1.18.1.3] | | |
| K00530 | 11 | Unclassified; E1.18.1.- | | |
| K00532 | 22 | ferredoxin hydrogenase [EC:1.12.7.2] | | |
| K00533 | 4 | ferredoxin hydrogenase large subunit [EC:1.12.7.2] | | |
| K00534 | 11 | ferredoxin hydrogenase small subunit [EC:1.12.7.2] | | |
| K00535 | 2 | Unclassified; E1.18.-.- | | |
| K00537 | 7 | arsenate reductase [EC:1.20.4.1] | | |
| K00538 | 5 | formate acetyltransferase activating enzyme [EC:1.97.1.4] | | |
| K00539 | 1 | Unclassified; E1.97.1.- | | |
| K00540 | 1132 | Unclassified; E1.-.-.- | | |
| K00544 | 5 | betaine-homocysteine S-methyltransferase [EC:2.1.1.5] | | |
| K00547 | 33 | homocysteine S-methyltransferase [EC:2.1.1.10] | | |
| K00548 | 117 | 5-methyltetrahydrofolate--homocysteine methyltransferase | | |
| K00549 | 72 | 5-methyltetrahydropteroyltriglutamate--homocysteine | | |
| K00551 | 21 | phosphatidylethanolamine N-methyltransferase [EC:2.1.1.17] | | |
| K00554 | 30 | tRNA (guanine-N1-)-methyltransferase [EC:2.1.1.31] | | |
| K00556 | 3 | tRNA (guanosine-2'-O-)-methyltransferase [EC:2.1.1.34] | | |
| K00557 | 12 | tRNA (uracil-5-)-methyltransferase [EC:2.1.1.35] | | |
| K00558 | 15 | DNA (cytosine-5-)-methyltransferase [EC:2.1.1.37] | | |
| K00559 | 1 | sterol 24-C-methyltransferase [EC:2.1.1.41] | | |
| K00560 | 9 | thymidylate synthase [EC:2.1.1.45] | | |
| K00561 | 14 | rRNA (adenine-N6-)-methyltransferase [EC:2.1.1.48] | | |
| K00563 | 4 | rRNA (guanine-N1-)-methyltransferase [EC:2.1.1.51] | | |
| K00564 | 1 | ribosomal RNA small subunit methyltransferase C [EC:2.1.1.52] | | |
| K00566 | 48 | tRNA (5-methylaminomethyl-2-thiouridylate)-methyltransferase | | |
| K00567 | 30 | methylated-DNA-[protein]-cysteine S-methyltransferase [EC:2.1.1.63] | | |
| K00568 | 65 | 3-demethylubiquinone-9 3-methyltransferase [EC:2.1.1.- 2.1.1.64] | | |
| K00569 | 4 | thiopurine S-methyltransferase [EC:2.1.1.67] | | |
| K00570 | 5 | phosphatidyl-N-methylethanolamine N-methyltransferase [EC:2.1.1.71] | | |
| K00571 | 26 | site-specific DNA-methyltransferase (adenine-specific) [EC:2.1.1.72] | | |
| K00573 | 52 | protein-L-isoaspartate(D-aspartate) O-methyltransferase | | |
| K00574 | 63 | cyclopropane-fatty-acyl-phospholipid synthase [EC:2.1.1.79] | | |
| K00575 | 79 | chemotaxis protein methyltransferase CheR [EC:2.1.1.80] | | |
| K00584 | 1 | tetrahydromethanopterin S-methyltransferase subunit H [EC:2.1.1.86] | | |
| K00587 | 7 | protein-S-isoprenylcysteine O-methyltransferase [EC:2.1.1.100] | | |
| K00588 | 15 | caffeoyl-CoA O-methyltransferase [EC:2.1.1.104] | | |
| K00590 | 11 | site-specific DNA-methyltransferase (cytosine-N4-specific) | | |
| K00594 | 3 | xylitol oxidase [EC:1.1.3.41] | | |
| K00595 | 6 | precorrin-6Y C5,15-methyltransferase / precorrin-8W decarboxylase | | |
| K00596 | 3 | 2,2-dialkylglycine decarboxylase (pyruvate) [EC:4.1.1.64] | | |
| K00598 | 16 | trans-aconitate 2-methyltransferase [EC:2.1.1.144] | | |
| K00599 | 435 | Unclassified; E2.1.1.- | | |
| K00600 | 49 | glycine hydroxymethyltransferase [EC:2.1.2.1] | | |
| K00602 | 28 | phosphoribosylaminoimidazolecarboxamide formyltransferase / IMP | | |
| K00603 | 20 | glutamate formiminotransferase [EC:2.1.2.5] | | |
| K00604 | 50 | methionyl-tRNA formyltransferase [EC:2.1.2.9] | | |
| K00605 | 69 | aminomethyltransferase [EC:2.1.2.10] | | |
| K00606 | 26 | 3-methyl-2-oxobutanoate hydroxymethyltransferase [EC:2.1.2.11] | | |
| K00607 | 3 | Unclassified; E2.1.2.- | | |
| K00609 | 35 | aspartate carbamoyltransferase catalytic subunit [EC:2.1.3.2] | | |
| K00610 | 12 | aspartate carbamoyltransferase regulatory subunit | | |
| K00611 | 50 | ornithine carbamoyltransferase [EC:2.1.3.3] | | |
| K00612 | 37 | carbamoyltransferase [EC:2.1.3.-] | | |
| K00613 | 1 | glycine amidinotransferase [EC:2.1.4.1] | | |
| K00614 | 13 | Unclassified; E2.1.-.- | | |
| K00615 | 102 | transketolase [EC:2.2.1.1] | | |
| K00616 | 40 | transaldolase [EC:2.2.1.2] | | |
| K00617 | 1 | Unclassified; E2.2.1.- | | |
| K00619 | 22 | amino-acid N-acetyltransferase [EC:2.3.1.1] | | |

| | | | | |
|---|---|---|---|---|
| K00620 | 31 | glutamate N-acetyltransferase / amino-acid N-acetyltransferase | | |
| K00622 | 5 | arylamine N-acetyltransferase [EC:2.3.1.5] | | |
| K00625 | 21 | phosphate acetyltransferase [EC:2.3.1.8] | | |
| K00626 | 211 | acetyl-CoA C-acetyltransferase [EC:2.3.1.9] | | |
| K00627 | 68 | pyruvate dehydrogenase E2 component (dihydrolipoamide | | |
| K00631 | 12 | glycerol-3-phosphate O-acyltransferase [EC:2.3.1.15] | | |
| K00632 | 93 | acetyl-CoA acyltransferase [EC:2.3.1.16] | | |
| K00633 | 18 | galactoside O-acetyltransferase [EC:2.3.1.18] | | |
| K00634 | 11 | phosphate butyryltransferase [EC:2.3.1.19] | | |
| K00638 | 6 | chloramphenicol O-acetyltransferase [EC:2.3.1.28] | | |
| K00639 | 42 | glycine C-acetyltransferase [EC:2.3.1.29] | | |
| K00640 | 27 | serine O-acetyltransferase [EC:2.3.1.30] | | |
| K00641 | 37 | homoserine O-acetyltransferase [EC:2.3.1.31] | | |
| K00643 | 12 | 5-aminolevulinate synthase [EC:2.3.1.37] | | |
| K00645 | 48 | [acyl-carrier-protein] S-malonyltransferase [EC:2.3.1.39] | | |
| K00647 | 39 | 3-oxoacyl-[acyl-carrier-protein] synthase I [EC:2.3.1.41] | | |
| K00648 | 79 | 3-oxoacyl-[acyl-carrier-protein] synthase III [EC:2.3.1.180] | | |
| K00651 | 3 | homoserine O-succinyltransferase [EC:2.3.1.46] | | |
| K00652 | 51 | 8-amino-7-oxononanoate synthase [EC:2.3.1.47] | | |
| K00654 | 7 | serine palmitoyltransferase [EC:2.3.1.50] | | |
| K00655 | 121 | 1-acyl-sn-glycerol-3-phosphate acyltransferase [EC:2.3.1.51] | | |
| K00656 | 38 | formate C-acetyltransferase [EC:2.3.1.54] | | |
| K00657 | 13 | diamine N-acetyltransferase [EC:2.3.1.57] | | |
| K00658 | 41 | 2-oxoglutarate dehydrogenase E2 component (dihydrolipoamide | | |
| K00660 | 12 | chalcone synthase [EC:2.3.1.74] | | |
| K00661 | 8 | maltose O-acetyltransferase [EC:2.3.1.79] | | |
| K00662 | 3 | aminoglycoside N3'-acetyltransferase [EC:2.3.1.81] | | |
| K00663 | 9 | aminoglycoside N6'-acetyltransferase [EC:2.3.1.82] | | |
| K00666 | 187 | fatty-acyl-CoA synthase [EC:6.2.1.-] | | |
| K00672 | 6 | formylmethanofuran--tetrahydromethanopterin N-formyltransferase | | |
| K00673 | 3 | arginine N-succinyltransferase [EC:2.3.1.109] | | |
| K00674 | 18 | 2,3,4,5-tetrahydropyridine-2-carboxylate N-succinyltransferase | | |
| K00675 | 16 | N-hydroxyarylamine O-acetyltransferase [EC:2.3.1.118] | | |
| K00676 | 20 | ribosomal-protein-alanine N-acetyltransferase [EC:2.3.1.128] | | |
| K00677 | 32 | UDP-N-acetylglucosamine acyltransferase [EC:2.3.1.129] | | |
| K00680 | 275 | Unclassified; E2.3.1.- | | |
| K00681 | 137 | gamma-glutamyltranspeptidase [EC:2.3.2.2] | | |
| K00683 | 3 | glutaminyl-peptide cyclotransferase [EC:2.3.2.5] | | |
| K00684 | 12 | leucyl/phenylalanyl-tRNA--protein transferase [EC:2.3.2.6] | | |
| K00685 | 7 | arginine-tRNA-protein transferase [EC:2.3.2.8] | | |
| K00688 | 90 | starch phosphorylase [EC:2.4.1.1] | | |
| K00690 | 3 | sucrose phosphorylase [EC:2.4.1.7] | | |
| K00691 | 17 | maltose phosphorylase [EC:2.4.1.8] | | |
| K00693 | 14 | glycogen(starch) synthase [EC:2.4.1.11] | | |
| K00694 | 12 | cellulose synthase (UDP-forming) [EC:2.4.1.12] | | |
| K00695 | 2 | sucrose synthase [EC:2.4.1.13] | | |
| K00696 | 1 | sucrose-phosphate synthase [EC:2.4.1.14] | | |
| K00697 | 44 | alpha,alpha-trehalose-phosphate synthase (UDP-forming) [EC:2.4.1.15] | | |
| K00700 | 62 | 1,4-alpha-glucan branching enzyme [EC:2.4.1.18] | | |
| K00701 | 2 | cyclomaltodextrin glucanotransferase [EC:2.4.1.19] | | |
| K00702 | 10 | cellobiose phosphorylase [EC:2.4.1.20] | | |
| K00703 | 39 | starch synthase [EC:2.4.1.21] | | |
| K00705 | 74 | 4-alpha-glucanotransferase [EC:2.4.1.25] | | |
| K00712 | 3 | poly(glycerol-phosphate) alpha-glucosyltransferase [EC:2.4.1.52] | | |
| K00720 | 5 | ceramide glucosyltransferase [EC:2.4.1.80] | | |
| K00721 | 123 | dolichol-phosphate mannosyltransferase [EC:2.4.1.83] | | |
| K00743 | 2 | N-acetyllactosaminide 3-alpha-galactosyltransferase [EC:2.4.1.87] | | |
| K00748 | 28 | lipid-A-disaccharide synthase [EC:2.4.1.182] | | |
| K00752 | 7 | hyaluronan synthase [EC:2.4.1.212] | | |

| | | | | |
|---|---|---|---|---|
| K00754 | 464 | Unclassified; E2.4.1.- | | |
| K00756 | 8 | pyrimidine-nucleoside phosphorylase [EC:2.4.2.2] | | |
| K00757 | 2 | uridine phosphorylase [EC:2.4.2.3] | | |
| K00758 | 21 | thymidine phosphorylase [EC:2.4.2.4] | | |
| K00759 | 26 | adenine phosphoribosyltransferase [EC:2.4.2.7] | | |
| K00760 | 33 | hypoxanthine phosphoribosyltransferase [EC:2.4.2.8] | | |
| K00761 | 13 | uracil phosphoribosyltransferase [EC:2.4.2.9] | | |
| K00762 | 22 | orotate phosphoribosyltransferase [EC:2.4.2.10] | | |
| K00763 | 180 | nicotinate phosphoribosyltransferase [EC:2.4.2.11] | | |
| K00764 | 58 | amidophosphoribosyltransferase [EC:2.4.2.14] | | |
| K00765 | 31 | ATP phosphoribosyltransferase [EC:2.4.2.17] | | |
| K00766 | 31 | anthranilate phosphoribosyltransferase [EC:2.4.2.18] | | |
| K00767 | 54 | nicotinate-nucleotide pyrophosphorylase (carboxylating) | | |
| K00768 | 8 | nicotinate-nucleotide--dimethylbenzimidazole | | |
| K00772 | 30 | 5'-methylthioadenosine phosphorylase [EC:2.4.2.28] | | |
| K00773 | 26 | queuine tRNA-ribosyltransferase [EC:2.4.2.29] | | |
| K00777 | 15 | Unclassified; E2.4.2.- | | |
| K00782 | 10 | hypothetical protein | | |
| K00783 | 10 | hypothetical protein | | |
| K00784 | 75 | ribonuclease Z [EC:3.1.26.11] | | |
| K00785 | 2 | beta-galactosamide-alpha-2,3-sialyltransferase [EC:2.4.99.-] | | |
| K00786 | 201 | Unclassified; E2.4.-.- | | |
| K00788 | 35 | thiamine-phosphate pyrophosphorylase [EC:2.5.1.3] | | |
| K00789 | 31 | S-adenosylmethionine synthetase [EC:2.5.1.6] | | |
| K00790 | 50 | UDP-N-acetylglucosamine 1-carboxyvinyltransferase [EC:2.5.1.7] | | |
| K00791 | 29 | tRNA dimethylallyltransferase [EC:2.5.1.75] | | |
| K00793 | 31 | riboflavin synthase alpha chain [EC:2.5.1.9] | | |
| K00794 | 15 | riboflavin synthase beta chain [EC:2.5.1.-] | | |
| K00795 | 8 | farnesyl diphosphate synthase [EC:2.5.1.1 2.5.1.10] | | |
| K00796 | 35 | dihydropteroate synthase [EC:2.5.1.15] | | |
| K00797 | 37 | spermidine synthase [EC:2.5.1.16] | | |
| K00798 | 27 | cob(I)alamin adenosyltransferase [EC:2.5.1.17] | | |
| K00799 | 108 | glutathione S-transferase [EC:2.5.1.18] | | |
| K00800 | 62 | 3-phosphoshikimate 1-carboxyvinyltransferase [EC:2.5.1.19] | | |
| K00801 | 13 | farnesyl-diphosphate farnesyltransferase [EC:2.5.1.21] | | |
| K00802 | 3 | spermine synthase [EC:2.5.1.22] | | |
| K00803 | 23 | alkyldihydroxyacetonephosphate synthase [EC:2.5.1.26] | | |
| K00805 | 9 | heptaprenyl diphosphate synthase [EC:2.5.1.30] | | |
| K00806 | 20 | undecaprenyl diphosphate synthase [EC:2.5.1.31] | | |
| K00808 | 4 | homospermidine synthase [EC:2.5.1.44] | | |
| K00809 | 18 | deoxyhypusine synthase [EC:2.5.1.46] | | |
| K00810 | 24 | Unclassified; E2.5.1.- | | |
| K00811 | 2 | aspartate aminotransferase [EC:2.6.1.1] | | |
| K00812 | 46 | aspartate aminotransferase [EC:2.6.1.1] | | |
| K00813 | 7 | aspartate aminotransferase [EC:2.6.1.1] | | |
| K00814 | 3 | alanine transaminase [EC:2.6.1.2] | | |
| K00817 | 59 | histidinol-phosphate aminotransferase [EC:2.6.1.9] | | |
| K00818 | 49 | acetylornithine aminotransferase [EC:2.6.1.11] | | |
| K00819 | 14 | ornithine--oxo-acid transaminase [EC:2.6.1.13] | | |
| K00820 | 67 | glucosamine--fructose-6-phosphate aminotransferase (isomerizing) | | |
| K00821 | 47 | acetylornithine/N-succinyldiaminopimelate aminotransferase | | |
| K00822 | 14 | beta-alanine--pyruvate transaminase [EC:2.6.1.18] | | |
| K00823 | 47 | 4-aminobutyrate aminotransferase [EC:2.6.1.19] | | |
| K00824 | 14 | D-alanine transaminase [EC:2.6.1.21] | | |
| K00825 | 17 | 2-aminoadipate transaminase [EC:2.6.1.39] | | |
| K00826 | 73 | branched-chain amino acid aminotransferase [EC:2.6.1.42] | | |
| K00828 | 29 | serine--glyoxylate transaminase [EC:2.6.1.45] | | |
| K00831 | 23 | phosphoserine aminotransferase [EC:2.6.1.52] | | |
| K00832 | 8 | aromatic-amino-acid transaminase [EC:2.6.1.57] | | |

| | | | | |
|---|---|---|---|---|
| K00833 | 19 | adenosylmethionine-8-amino-7-oxononanoate aminotransferase | | |
| K00835 | 12 | valine--pyruvate aminotransferase [EC:2.6.1.66] | | |
| K00836 | 5 | diaminobutyrate-2-oxoglutarate transaminase [EC:2.6.1.76] | | |
| K00837 | 274 | Unclassified; E2.6.1.- | | |
| K00839 | 38 | aminotransferase [EC:2.6.1.-] | | |
| K00840 | 1 | succinylornithine aminotransferase [EC:2.6.1.81] | | |
| K00841 | 1 | aminotransferase [EC:2.6.1.-] | | |
| K00842 | 11 | aminotransferase [EC:2.6.1.-] | | |
| K00843 | 6 | Unclassified; E2.6.-.- | | |
| K00845 | 73 | glucokinase [EC:2.7.1.2] | | |
| K00846 | 2 | ketohexokinase [EC:2.7.1.3] | | |
| K00847 | 33 | fructokinase [EC:2.7.1.4] | | |
| K00848 | 14 | rhamnulokinase [EC:2.7.1.5] | | |
| K00849 | 14 | galactokinase [EC:2.7.1.6] | | |
| K00850 | 56 | 6-phosphofructokinase [EC:2.7.1.11] | | |
| K00851 | 14 | gluconokinase [EC:2.7.1.12] | | |
| K00852 | 25 | ribokinase [EC:2.7.1.15] | | |
| K00853 | 8 | L-ribulokinase [EC:2.7.1.16] | | |
| K00854 | 34 | xylulokinase [EC:2.7.1.17] | | |
| K00855 | 13 | phosphoribulokinase [EC:2.7.1.19] | | |
| K00856 | 21 | adenosine kinase [EC:2.7.1.20] | | |
| K00857 | 8 | thymidine kinase [EC:2.7.1.21] | | |
| K00858 | 35 | NAD+ kinase [EC:2.7.1.23] | | |
| K00859 | 35 | dephospho-CoA kinase [EC:2.7.1.24] | | |
| K00860 | 18 | adenylylsulfate kinase [EC:2.7.1.25] | | |
| K00861 | 3 | riboflavin kinase [EC:2.7.1.26] | | |
| K00862 | 1 | erythritol kinase [EC:2.7.1.27] | | |
| K00863 | 8 | dihydroxyacetone kinase [EC:2.7.1.29] | | |
| K00864 | 41 | glycerol kinase [EC:2.7.1.30] | | |
| K00865 | 12 | glycerate kinase [EC:2.7.1.31] | | |
| K00866 | 1 | choline kinase [EC:2.7.1.32] | | |
| K00867 | 2 | type I pantothenate kinase [EC:2.7.1.33] | | |
| K00868 | 445 | pyridoxine kinase [EC:2.7.1.35] | | |
| K00869 | 1 | mevalonate kinase [EC:2.7.1.36] | | |
| K00870 | 68 | protein kinase [EC:2.7.1.37] | | |
| K00872 | 9 | homoserine kinase [EC:2.7.1.39] | | |
| K00873 | 44 | pyruvate kinase [EC:2.7.1.40] | | |
| K00874 | 20 | 2-dehydro-3-deoxygluconokinase [EC:2.7.1.45] | | |
| K00875 | 2 | D-ribulokinase [EC:2.7.1.47] | | |
| K00876 | 12 | uridine kinase [EC:2.7.1.48] | | |
| K00877 | 3 | hydroxymethylpyrimidine kinase [EC:2.7.1.49] | | |
| K00878 | 3 | hydroxyethylthiazole kinase [EC:2.7.1.50] | | |
| K00879 | 4 | L-fuculokinase [EC:2.7.1.51] | | |
| K00880 | 1 | L-xylulokinase [EC:2.7.1.53] | | |
| K00882 | 14 | 1-phosphofructokinase [EC:2.7.1.56] | | |
| K00883 | 1 | 2-dehydro-3-deoxygalactonokinase [EC:2.7.1.58] | | |
| K00884 | 7 | N-acetylglucosamine kinase [EC:2.7.1.59] | | |
| K00885 | 1 | N-acylmannosamine kinase [EC:2.7.1.60] | | |
| K00886 | 9 | polyphosphate glucokinase [EC:2.7.1.63] | | |
| K00887 | 10 | undecaprenol kinase [EC:2.7.1.66] | | |
| K00891 | 31 | shikimate kinase [EC:2.7.1.71] | | |
| K00894 | 3 | ethanolamine kinase [EC:2.7.1.82] | | |
| K00895 | 22 | pyrophosphate--fructose-6-phosphate 1-phosphotransferase | | |
| K00897 | 10 | aminoglycoside 3'-phosphotransferase [EC:2.7.1.95] | | |
| K00901 | 6 | diacylglycerol kinase [EC:2.7.1.107] | | |
| K00903 | 70 | protein-tyrosine kinase [EC:2.7.10.-] | | |
| K00904 | 18 | deoxyguanosine kinase [EC:2.7.1.113] | | |
| K00906 | 11 | isocitrate dehydrogenase kinase/phosphatase [EC:2.7.11.5 3.1.3.-] | | |
| K00908 | 216 | Ca2+/calmodulin-dependent protein kinase [EC:2.7.11.17] | | |

| | | | | |
|---|---|---|---|---|
| K00912 | 13 | tetraacyldisaccharide 4'-kinase [EC:2.7.1.130] | | |
| K00917 | 9 | tagatose 6-phosphate kinase [EC:2.7.1.144] | | |
| K00919 | 25 | 4-diphosphocytidyl-2-C-methyl-D-erythritol kinase [EC:2.7.1.148] | | |
| K00924 | 351 | Unclassified; E2.7.1.- | | |
| K00925 | 31 | acetate kinase [EC:2.7.2.1] | | |
| K00926 | 18 | carbamate kinase [EC:2.7.2.2] | | |
| K00927 | 35 | phosphoglycerate kinase [EC:2.7.2.3] | | |
| K00928 | 33 | aspartate kinase [EC:2.7.2.4] | | |
| K00929 | 3 | butyrate kinase [EC:2.7.2.7] | | |
| K00930 | 23 | acetylglutamate kinase [EC:2.7.2.8] | | |
| K00931 | 54 | glutamate 5-kinase [EC:2.7.2.11] | | |
| K00932 | 1 | propionate kinase [EC:2.7.2.15] | | |
| K00933 | 1 | creatine kinase [EC:2.7.3.2] | | |
| K00934 | 4 | arginine kinase [EC:2.7.3.3] | | |
| K00935 | 4 | Unclassified; E2.7.3.9 | | |
| K00936 | 1280 | Unclassified; E2.7.3.- | | |
| K00937 | 60 | polyphosphate kinase [EC:2.7.4.1] | | |
| K00938 | 5 | phosphomevalonate kinase [EC:2.7.4.2] | | |
| K00939 | 34 | adenylate kinase [EC:2.7.4.3] | | |
| K00940 | 20 | nucleoside-diphosphate kinase [EC:2.7.4.6] | | |
| K00941 | 29 | phosphomethylpyrimidine kinase [EC:2.7.4.7] | | |
| K00942 | 26 | guanylate kinase [EC:2.7.4.8] | | |
| K00943 | 33 | dTMP kinase [EC:2.7.4.9] | | |
| K00945 | 33 | cytidylate kinase [EC:2.7.4.14] | | |
| K00946 | 33 | thiamine-monophosphate kinase [EC:2.7.4.16] | | |
| K00947 | 27 | Unclassified; E2.7.4.- | | |
| K00948 | 177 | ribose-phosphate pyrophosphokinase [EC:2.7.6.1] | | |
| K00950 | 17 | 2-amino-4-hydroxy-6-hydroxymethyldihydropteridine diphosphokinase | | |
| K00951 | 90 | GTP pyrophosphokinase [EC:2.7.6.5] | | |
| K00954 | 23 | pantetheine-phosphate adenylyltransferase [EC:2.7.7.3] | | |
| K00955 | 28 | bifunctional enzyme CysN/CysC [EC:2.7.7.4 2.7.1.25] | | |
| K00956 | 29 | sulfate adenylyltransferase subunit 1 [EC:2.7.7.4] | | |
| K00957 | 10 | sulfate adenylyltransferase subunit 2 [EC:2.7.7.4] | | |
| K00958 | 25 | sulfate adenylyltransferase [EC:2.7.7.4] | | |
| K00960 | 7 | DNA-directed RNA polymerase [EC:2.7.7.6] | | |
| K00961 | 7 | DNA polymerase [EC:2.7.7.7] | | |
| K00962 | 39 | polyribonucleotide nucleotidyltransferase [EC:2.7.7.8] | | |
| K00963 | 23 | UTP--glucose-1-phosphate uridylyltransferase [EC:2.7.7.9] | | |
| K00964 | 23 | galactose-1-phosphate uridylyltransferase [EC:2.7.7.10] | | |
| K00965 | 24 | UDPglucose--hexose-1-phosphate uridylyltransferase [EC:2.7.7.12] | | |
| K00966 | 49 | mannose-1-phosphate guanylyltransferase [EC:2.7.7.13] | | |
| K00969 | 56 | nicotinate-nucleotide adenylyltransferase [EC:2.7.7.18] | | |
| K00970 | 57 | poly(A) polymerase [EC:2.7.7.19] | | |
| K00971 | 42 | mannose-1-phosphate guanylyltransferase [EC:2.7.7.22] | | |
| K00972 | 3 | UDP-N-acetylglucosamine pyrophosphorylase [EC:2.7.7.23] | | |
| K00973 | 45 | glucose-1-phosphate thymidylyltransferase [EC:2.7.7.24] | | |
| K00974 | 27 | tRNA nucleotidyltransferase (CCA-adding enzyme) [EC:2.7.7.25 | | |
| K00975 | 42 | glucose-1-phosphate adenylyltransferase [EC:2.7.7.27] | | |
| K00978 | 15 | glucose-1-phosphate cytidylyltransferase [EC:2.7.7.33] | | |
| K00979 | 26 | 3-deoxy-manno-octulosonate cytidylyltransferase (CMP-KDO synthetase) | | |
| K00980 | 8 | glycerol-3-phosphate cytidylyltransferase [EC:2.7.7.39] | | |
| K00981 | 27 | phosphatidate cytidylyltransferase [EC:2.7.7.41] | | |
| K00982 | 55 | glutamate-ammonia-ligase adenylyltransferase [EC:2.7.7.42] | | |
| K00983 | 2 | N-acylneuraminate cytidylyltransferase [EC:2.7.7.43] | | |
| K00985 | 6 | RNA-directed RNA polymerase [EC:2.7.7.48] | | |
| K00986 | 61 | RNA-directed DNA polymerase [EC:2.7.7.49] | | |
| K00989 | 19 | ribonuclease PH [EC:2.7.7.56] | | |
| K00990 | 45 | [protein-PII] uridylyltransferase [EC:2.7.7.59] | | |
| K00991 | 20 | 2-C-methyl-D-erythritol 4-phosphate cytidylyltransferase | | |

| | | | | |
|---|---|---|---|---|
| K00992 | 28 | Unclassified; E2.7.7.- | | |
| K00995 | 115 | CDP-diacylglycerol--glycerol-3-phosphate 3-phosphatidyltransferase | | |
| K00996 | 3 | undecaprenyl-phosphate galactose phosphotransferase [EC:2.7.8.6] | | |
| K00997 | 15 | holo-[acyl-carrier protein] synthase [EC:2.7.8.7] | | |
| K00998 | 20 | phosphatidylserine synthase [EC:2.7.8.8] | | |
| K00999 | 4 | CDP-diacylglycerol--inositol 3-phosphatidyltransferase [EC:2.7.8.11] | | |
| K01000 | 52 | phospho-N-acetylmuramoyl-pentapeptide-transferase [EC:2.7.8.13] | | |
| K01002 | 9 | phosphoglycerol transferase [EC:2.7.8.20] | | |
| K01003 | 30 | carboxyvinyl-carboxyphosphonate phosphorylmutase [EC:2.7.8.23] | | |
| K01005 | 35 | Unclassified; E2.7.8.- | | |
| K01006 | 55 | pyruvate,orthophosphate dikinase [EC:2.7.9.1] | | |
| K01007 | 80 | pyruvate, water dikinase [EC:2.7.9.2] | | |
| K01008 | 29 | selenide, water dikinase [EC:2.7.9.3] | | |
| K01010 | 60 | thiosulfate sulfurtransferase [EC:2.8.1.1] | | |
| K01011 | 21 | 3-mercaptopyruvate sulfurtransferase [EC:2.8.1.2] | | |
| K01012 | 22 | biotin synthetase [EC:2.8.1.6] | | |
| K01013 | 25 | Unclassified; E2.8.1.- | | |
| K01026 | 7 | propionate CoA-transferase [EC:2.8.3.1] | | |
| K01027 | 3 | 3-oxoacid CoA-transferase [EC:2.8.3.5] | | |
| K01028 | 6 | 3-oxoacid CoA-transferase subunit A [EC:2.8.3.5] | | |
| K01029 | 3 | 3-oxoacid CoA-transferase subunit B [EC:2.8.3.5] | | |
| K01031 | 3 | 3-oxoadipate CoA-transferase, alpha subunit [EC:2.8.3.6] | | |
| K01032 | 3 | 3-oxoadipate CoA-transferase, beta subunit [EC:2.8.3.6] | | |
| K01035 | 2 | acetate CoA-transferase beta subunit [EC:2.8.3.8] | | |
| K01036 | 1 | butyrate-acetoacetate CoA-transferase [EC:2.8.3.9] | | |
| K01039 | 25 | glutaconate CoA-transferase, subunit A [EC:2.8.3.12] | | |
| K01040 | 18 | glutaconate CoA-transferase, subunit B [EC:2.8.3.12] | | |
| K01041 | 111 | Unclassified; E2.8.3.- | | |
| K01042 | 37 | L-seryl-tRNA(Ser) seleniumtransferase [EC:2.9.1.1] | | |
| K01043 | 161 | Unclassified; E2.-.-.- | | |
| K01044 | 56 | carboxylesterase [EC:3.1.1.1] | | |
| K01046 | 68 | triacylglycerol lipase [EC:3.1.1.3] | | |
| K01048 | 6 | lysophospholipase [EC:3.1.1.5] | | |
| K01051 | 2 | pectinesterase [EC:3.1.1.11] | | |
| K01053 | 63 | gluconolactonase [EC:3.1.1.17] | | |
| K01054 | 3 | acylglycerol lipase [EC:3.1.1.23] | | |
| K01055 | 56 | 3-oxoadipate enol-lactonase [EC:3.1.1.24] | | |
| K01056 | 29 | peptidyl-tRNA hydrolase, PTH1 family [EC:3.1.1.29] | | |
| K01057 | 24 | 6-phosphogluconolactonase [EC:3.1.1.31] | | |
| K01058 | 8 | phospholipase A1 [EC:3.1.1.32] | | |
| K01060 | 3 | cephalosporin-C deacetylase [EC:3.1.1.41] | | |
| K01061 | 68 | carboxymethylenebutenolidase [EC:3.1.1.45] | | |
| K01062 | 1 | 1-alkyl-2-acetylglycerophosphocholine esterase [EC:3.1.1.47] | | |
| K01066 | 94 | esterase / lipase [EC:3.1.1.-] | | |
| K01067 | 12 | acetyl-CoA hydrolase [EC:3.1.2.1] | | |
| K01068 | 5 | palmitoyl-CoA hydrolase [EC:3.1.2.2] | | |
| K01069 | 110 | hydroxyacylglutathione hydrolase [EC:3.1.2.6] | | |
| K01070 | 5 | S-formylglutathione hydrolase [EC:3.1.2.12] | | |
| K01071 | 2 | oleoyl-[acyl-carrier-protein] hydrolase [EC:3.1.2.14] | | |
| K01073 | 7 | acyl-CoA hydrolase [EC:3.1.2.20] | | |
| K01075 | 12 | 4-hydroxybenzoyl-CoA thioesterase [EC:3.1.2.23] | | |
| K01076 | 26 | Unclassified; E3.1.2.- | | |
| K01077 | 90 | alkaline phosphatase [EC:3.1.3.1] | | |
| K01078 | 17 | acid phosphatase [EC:3.1.3.2] | | |
| K01079 | 34 | phosphoserine phosphatase [EC:3.1.3.3] | | |
| K01081 | 32 | 5'-nucleotidase [EC:3.1.3.5] | | |
| K01083 | 1 | 3-phytase [EC:3.1.3.8] | | |
| K01087 | 73 | trehalose-phosphatase [EC:3.1.3.12] | | |
| K01090 | 68 | protein phosphatase [EC:3.1.3.16] | | |

| | | | | |
|---|---|---|---|---|
| K01091 | 72 | phosphoglycolate phosphatase [EC:3.1.3.18] | | |
| K01092 | 58 | myo-inositol-1(or 4)-monophosphatase [EC:3.1.3.25] | | |
| K01093 | 4 | 4-phytase / acid phosphatase [EC:3.1.3.26 3.1.3.2] | | |
| K01095 | 8 | phosphatidylglycerophosphatase A [EC:3.1.3.27] | | |
| K01096 | 4 | phosphatidylglycerophosphatase B [EC:3.1.3.27] | | |
| K01101 | 7 | 4-nitrophenyl phosphatase [EC:3.1.3.41] | | |
| K01103 | 1 | fructose-2,6-bisphosphatase [EC:3.1.3.46] | | |
| K01104 | 39 | protein-tyrosine phosphatase [EC:3.1.3.48] | | |
| K01112 | 32 | Unclassified; E3.1.3.- | | |
| K01113 | 42 | phosphodiesterase/alkaline phosphatase D [EC:3.1.4.1] | | |
| K01114 | 33 | phospholipase C [EC:3.1.4.3] | | |
| K01115 | 8 | phospholipase D [EC:3.1.4.4] | | |
| K01118 | 3 | FMN-dependent NADH-azoreductase [EC:1.7.-.-] | | |
| K01119 | 13 | 2',3'-cyclic-nucleotide 2'-phosphodiesterase [EC:3.1.4.16] | | |
| K01120 | 22 | 3',5'-cyclic-nucleotide phosphodiesterase [EC:3.1.4.17] | | |
| K01121 | 1 | 2',3'-cyclic-nucleotide 3'-phosphodiesterase [EC:3.1.4.37] | | |
| K01126 | 47 | glycerophosphoryl diester phosphodiesterase [EC:3.1.4.46] | | |
| K01128 | 8 | Unclassified; E3.1.4.- | | |
| K01129 | 24 | dGTPase [EC:3.1.5.1] | | |
| K01130 | 86 | arylsulfatase [EC:3.1.6.1] | | |
| K01131 | 7 | steryl-sulfatase [EC:3.1.6.2] | | |
| K01132 | 17 | N-acetylgalactosamine-6-sulfatase [EC:3.1.6.4] | | |
| K01133 | 31 | choline-sulfatase [EC:3.1.6.6] | | |
| K01134 | 23 | arylsulfatase A [EC:3.1.6.8] | | |
| K01135 | 17 | arylsulfatase B [EC:3.1.6.12] | | |
| K01136 | 8 | iduronate 2-sulfatase [EC:3.1.6.13] | | |
| K01137 | 10 | N-acetylglucosamine-6-sulfatase [EC:3.1.6.14] | | |
| K01138 | 72 | Unclassified; E3.1.6.- | | |
| K01139 | 45 | guanosine-3',5'-bis(diphosphate) 3'-pyrophosphohydrolase | | |
| K01142 | 29 | exodeoxyribonuclease III [EC:3.1.11.2] | | |
| K01144 | 43 | exodeoxyribonuclease V [EC:3.1.11.5] | | |
| K01146 | 7 | Unclassified; E3.1.11.- | | |
| K01147 | 24 | exoribonuclease II [EC:3.1.13.1] | | |
| K01151 | 37 | deoxyribonuclease IV [EC:3.1.21.2] | | |
| K01152 | 2 | type I restriction enzyme [EC:3.1.21.3] | | |
| K01153 | 63 | type I restriction enzyme, R subunit [EC:3.1.21.3] | | |
| K01154 | 15 | type I restriction enzyme, S subunit [EC:3.1.21.3] | | |
| K01155 | 2 | type II restriction enzyme [EC:3.1.21.4] | | |
| K01156 | 7 | type III restriction enzyme [EC:3.1.21.5] | | |
| K01157 | 37 | Unclassified; E3.1.21.- | | |
| K01159 | 34 | crossover junction endodeoxyribonuclease RuvC [EC:3.1.22.4] | | |
| K01162 | 2 | ribonuclease III [EC:3.1.26.3] | | |
| K01166 | 1 | ribonuclease T2 [EC:3.1.27.1] | | |
| K01167 | 9 | ribonuclease T1 [EC:3.1.27.3] | | |
| K01172 | 1 | Unclassified; E3.1.27.- | | |
| K01173 | 1 | endonuclease [EC:3.1.30.-] | | |
| K01174 | 9 | micrococcal nuclease [EC:3.1.31.1] | | |
| K01175 | 109 | Unclassified; E3.1.-.- | | |
| K01176 | 36 | alpha-amylase [EC:3.2.1.1] | | |
| K01178 | 57 | glucoamylase [EC:3.2.1.3] | | |
| K01179 | 39 | endoglucanase [EC:3.2.1.4] | | |
| K01181 | 11 | endo-1,4-beta-xylanase [EC:3.2.1.8] | | |
| K01182 | 13 | oligo-1,6-glucosidase [EC:3.2.1.10] | | |
| K01183 | 28 | chitinase [EC:3.2.1.14] | | |
| K01184 | 1 | polygalacturonase [EC:3.2.1.15] | | |
| K01185 | 14 | lysozyme [EC:3.2.1.17] | | |
| K01186 | 2 | sialidase-1 [EC:3.2.1.18] | | |
| K01187 | 42 | alpha-glucosidase [EC:3.2.1.20] | | |
| K01188 | 77 | beta-glucosidase [EC:3.2.1.21] | | |

| | | | | |
|---|---|---|---|---|
| K01190 | 51 | beta-galactosidase [EC:3.2.1.23] | | |
| K01191 | 17 | alpha-mannosidase [EC:3.2.1.24] | | |
| K01192 | 9 | beta-mannosidase [EC:3.2.1.25] | | |
| K01193 | 2 | beta-fructofuranosidase [EC:3.2.1.26] | | |
| K01194 | 6 | alpha,alpha-trehalase [EC:3.2.1.28] | | |
| K01195 | 8 | beta-glucuronidase [EC:3.2.1.31] | | |
| K01197 | 1 | hyaluronoglucosaminidase [EC:3.2.1.35] | | |
| K01198 | 9 | xylan 1,4-beta-xylosidase [EC:3.2.1.37] | | |
| K01199 | 9 | glucan endo-1,3-beta-D-glucosidase [EC:3.2.1.39] | | |
| K01200 | 4 | pullulanase [EC:3.2.1.41] | | |
| K01201 | 13 | glucosylceramidase [EC:3.2.1.45] | | |
| K01206 | 21 | alpha-L-fucosidase [EC:3.2.1.51] | | |
| K01207 | 53 | beta-N-acetylhexosaminidase [EC:3.2.1.52] | | |
| K01208 | 1 | cyclomaltodextrinase [EC:3.2.1.54] | | |
| K01209 | 22 | alpha-N-arabinofuranosidase [EC:3.2.1.55] | | |
| K01210 | 10 | glucan 1,3-beta-glucosidase [EC:3.2.1.58] | | |
| K01212 | 11 | levanase [EC:3.2.1.65] | | |
| K01213 | 6 | galacturan 1,4-alpha-galacturonidase [EC:3.2.1.67] | | |
| K01215 | 2 | glucan 1,6-alpha-glucosidase [EC:3.2.1.70] | | |
| K01216 | 10 | licheninase [EC:3.2.1.73] | | |
| K01218 | 6 | mannan endo-1,4-beta-mannosidase [EC:3.2.1.78] | | |
| K01220 | 15 | 6-phospho-beta-galactosidase [EC:3.2.1.85] | | |
| K01222 | 6 | 6-phospho-beta-glucosidase [EC:3.2.1.86] | | |
| K01223 | 1 | 6-phospho-beta-glucosidase [EC:3.2.1.86] | | |
| K01224 | 2 | arabinogalactan endo-1,4-beta-galactosidase [EC:3.2.1.89] | | |
| K01225 | 25 | cellulose 1,4-beta-cellobiosidase [EC:3.2.1.91] | | |
| K01226 | 3 | trehalose-6-phosphate hydrolase [EC:3.2.1.93] | | |
| K01234 | 2 | neopullulanase [EC:3.2.1.135] | | |
| K01235 | 3 | alpha-glucuronidase [EC:3.2.1.139] | | |
| K01236 | 28 | maltooligosyltrehalose trehalohydrolase [EC:3.2.1.141] | | |
| K01238 | 177 | Unclassified; E3.2.1.- | | |
| K01239 | 17 | purine nucleosidase [EC:3.2.2.1] | | |
| K01241 | 4 | AMP nucleosidase [EC:3.2.2.4] | | |
| K01243 | 6 | S-adenosylhomocysteine/5'-methylthioadenosine nucleosidase | | |
| K01246 | 12 | DNA-3-methyladenine glycosylase I [EC:3.2.2.20] | | |
| K01247 | 18 | DNA-3-methyladenine glycosylase II [EC:3.2.2.21] | | |
| K01249 | 33 | DNA glycosylase [EC:3.2.2.-] | | |
| K01250 | 8 | pyrimidine-specific ribonucleoside hydrolase [EC:3.2.-.-] | | |
| K01251 | 29 | adenosylhomocysteinase [EC:3.3.1.1] | | |
| K01253 | 35 | microsomal epoxide hydrolase [EC:3.3.2.9] | | |
| K01255 | 52 | leucyl aminopeptidase [EC:3.4.11.1] | | |
| K01256 | 102 | aminopeptidase N [EC:3.4.11.2] | | |
| K01258 | 13 | tripeptide aminopeptidase [EC:3.4.11.4] | | |
| K01259 | 49 | proline iminopeptidase [EC:3.4.11.5] | | |
| K01262 | 80 | X-Pro aminopeptidase [EC:3.4.11.9] | | |
| K01263 | 9 | cytosol alanyl aminopeptidase [EC:3.4.11.14] | | |
| K01264 | 9 | aminopeptidase Y [EC:3.4.11.15] | | |
| K01265 | 30 | methionyl aminopeptidase [EC:3.4.11.18] | | |
| K01266 | 19 | D-aminopeptidase [EC:3.4.11.19] | | |
| K01267 | 2 | aspartyl aminopeptidase [EC:3.4.11.21] | | |
| K01268 | 1 | aminopeptidase I [EC:3.4.11.22] | | |
| K01269 | 174 | aminopeptidase [EC:3.4.11.-] | | |
| K01270 | 3 | aminoacylhistidine dipeptidase [EC:3.4.13.3] | | |
| K01271 | 52 | X-Pro dipeptidase [EC:3.4.13.9] | | |
| K01273 | 44 | membrane dipeptidase [EC:3.4.13.19] | | |
| K01274 | 10 | D-alanyl-D-alanine dipeptidase [EC:3.4.13.-] | | |
| K01277 | 4 | dipeptidyl-peptidase III [EC:3.4.14.4] | | |
| K01278 | 26 | dipeptidyl-peptidase 4 [EC:3.4.14.5] | | |
| K01281 | 8 | X-Pro dipeptidyl-peptidase [EC:3.4.14.11] | | |

| | | | | |
|---|---|---|---|---|
| K01283 | 4 | peptidyl-dipeptidase A [EC:3.4.15.1] | | |
| K01284 | 35 | peptidyl-dipeptidase Dcp [EC:3.4.15.5] | | |
| K01286 | 36 | D-alanyl-D-alanine carboxypeptidase [EC:3.4.16.4] | | |
| K01289 | 13 | carboxypeptidase [EC:3.4.16.-] | | |
| K01295 | 41 | glutamate carboxypeptidase [EC:3.4.17.11] | | |
| K01297 | 11 | muramoyltetrapeptide carboxypeptidase [EC:3.4.17.13] | | |
| K01299 | 20 | carboxypeptidase Taq [EC:3.4.17.19] | | |
| K01301 | 10 | glutamate carboxypeptidase II [EC:3.4.17.21] | | |
| K01302 | 1 | Unclassified; E3.4.17.- | | |
| K01303 | 93 | acylaminoacyl-peptidase [EC:3.4.19.1] | | |
| K01304 | 3 | pyroglutamyl-peptidase [EC:3.4.19.3] | | |
| K01312 | 9 | trypsin [EC:3.4.21.4] | | |
| K01318 | 1 | glutamyl endopeptidase [EC:3.4.21.19] | | |
| K01322 | 103 | prolyl oligopeptidase [EC:3.4.21.26] | | |
| K01325 | 2 | tissue kallikrein [EC:3.4.21.35] | | |
| K01338 | 158 | ATP-dependent Lon protease [EC:3.4.21.53] | | |
| K01341 | 5 | kexin [EC:3.4.21.61] | | |
| K01342 | 54 | subtilisin [EC:3.4.21.62] | | |
| K01344 | 1 | protein C (activated) [EC:3.4.21.69] | | |
| K01347 | 2 | IgA-specific serine endopeptidase [EC:3.4.21.72] | | |
| K01354 | 61 | oligopeptidase B [EC:3.4.21.83] | | |
| K01356 | 28 | repressor LexA [EC:3.4.21.88] | | |
| K01358 | 30 | ATP-dependent Clp protease, protease subunit [EC:3.4.21.92] | | |
| K01361 | 6 | lactocepin [EC:3.4.21.96] | | |
| K01362 | 487 | Unclassified; E3.4.21.- | | |
| K01372 | 2 | bleomycin hydrolase [EC:3.4.22.40] | | |
| K01376 | 12 | Unclassified; E3.4.22.- | | |
| K01387 | 13 | microbial collagenase [EC:3.4.24.3] | | |
| K01389 | 2 | neprilysin [EC:3.4.24.11] | | |
| K01392 | 9 | thimet oligopeptidase [EC:3.4.24.15] | | |
| K01400 | 2 | bacillolysin [EC:3.4.24.28] | | |
| K01401 | 1 | aureolysin [EC:3.4.24.29] | | |
| K01406 | 35 | serralysin [EC:3.4.24.40] | | |
| K01407 | 1 | protease III [EC:3.4.24.55] | | |
| K01409 | 44 | O-sialoglycoprotein endopeptidase [EC:3.4.24.57] | | |
| K01412 | 104 | mitochondrial processing peptidase [EC:3.4.24.64] | | |
| K01414 | 25 | oligopeptidase A [EC:3.4.24.70] | | |
| K01415 | 46 | endothelin-converting enzyme [EC:3.4.24.71] | | |
| K01416 | 2 | snapalysin [EC:3.4.24.77] | | |
| K01417 | 359 | Unclassified; E3.4.24.- | | |
| K01419 | 10 | ATP-dependent HslUV protease, peptidase subunit HslV [EC:3.4.25.-] | | |
| K01420 | 81 | CRP/FNR family transcriptional regulator, anaerobic regulatory | | |
| K01421 | 2 | putative membrane protein | | |
| K01422 | 101 | Unclassified; E3.4.99.- | | |
| K01423 | 395 | Unclassified; E3.4.-.- | | |
| K01424 | 54 | L-asparaginase [EC:3.5.1.1] | | |
| K01425 | 10 | glutaminase [EC:3.5.1.2] | | |
| K01426 | 156 | amidase [EC:3.5.1.4] | | |
| K01428 | 11 | urease alpha subunit [EC:3.5.1.5] | | |
| K01429 | 3 | urease beta subunit [EC:3.5.1.5] | | |
| K01430 | 6 | urease gamma subunit [EC:3.5.1.5] | | |
| K01431 | 27 | beta-ureidopropionase [EC:3.5.1.6] | | |
| K01433 | 7 | formyltetrahydrofolate deformylase [EC:3.5.1.10] | | |
| K01434 | 69 | penicillin amidase [EC:3.5.1.11] | | |
| K01436 | 74 | aminoacylase [EC:3.5.1.14] | | |
| K01438 | 28 | acetylornithine deacetylase [EC:3.5.1.16] | | |
| K01439 | 82 | succinyl-diaminopimelate desuccinylase [EC:3.5.1.18] | | |
| K01443 | 68 | N-acetylglucosamine-6-phosphate deacetylase [EC:3.5.1.25] | | |
| K01444 | 9 | N4-(beta-N-acetylglucosaminyl)-L-asparaginase [EC:3.5.1.26] | | |

| | | | | |
|---|---|---|---|---|
| K01446 | 2 | N-acetylmuramoyl-L-alanine amidase [EC:3.5.1.28] | | |
| K01447 | 31 | N-acetylmuramoyl-L-alanine amidase [EC:3.5.1.28] | | |
| K01448 | 611 | N-acetylmuramoyl-L-alanine amidase [EC:3.5.1.28] | | |
| K01449 | 4 | N-acetylmuramoyl-L-alanine amidase [EC:3.5.1.28] | | |
| K01451 | 45 | hippurate hydrolase [EC:3.5.1.32] | | |
| K01452 | 38 | chitin deacetylase [EC:3.5.1.41] | | |
| K01453 | 19 | Unclassified; E3.5.1.46 | | |
| K01454 | 11 | Unclassified; E3.5.1.48 | | |
| K01455 | 26 | formamidase [EC:3.5.1.49] | | |
| K01457 | 9 | allophanate hydrolase [EC:3.5.1.54] | | |
| K01458 | 6 | N-formylglutamate deformylase [EC:3.5.1.68] | | |
| K01459 | 6 | Unclassified; E3.5.1.77 | | |
| K01461 | 67 | Unclassified; E3.5.1.82 | | |
| K01462 | 44 | Unclassified; E3.5.1.88 | | |
| K01463 | 176 | Unclassified; E3.5.1.- | | |
| K01464 | 42 | dihydropyrimidinase [EC:3.5.2.2] | | |
| K01465 | 147 | dihydroorotase [EC:3.5.2.3] | | |
| K01466 | 28 | allantoinase [EC:3.5.2.5] | | |
| K01467 | 153 | beta-lactamase [EC:3.5.2.6] | | |
| K01468 | 45 | imidazolonepropionase [EC:3.5.2.7] | | |
| K01469 | 35 | 5-oxoprolinase (ATP-hydrolysing) [EC:3.5.2.9] | | |
| K01470 | 24 | creatinine amidohydrolase [EC:3.5.2.10] | | |
| K01471 | 12 | Unclassified; E3.5.2.12 | | |
| K01473 | 68 | N-methylhydantoinase A [EC:3.5.2.14] | | |
| K01474 | 76 | N-methylhydantoinase B [EC:3.5.2.14] | | |
| K01476 | 13 | arginase [EC:3.5.3.1] | | |
| K01477 | 8 | allantoicase [EC:3.5.3.4] | | |
| K01478 | 10 | arginine deiminase [EC:3.5.3.6] | | |
| K01479 | 4 | formiminoglutamase [EC:3.5.3.8] | | |
| K01480 | 40 | agmatinase [EC:3.5.3.11] | | |
| K01482 | 9 | dimethylargininase [EC:3.5.3.18] | | |
| K01483 | 1 | ureidoglycolate hydrolase [EC:3.5.3.19] | | |
| K01484 | 1 | succinylarginine dihydrolase [EC:3.5.3.23] | | |
| K01485 | 25 | cytosine deaminase [EC:3.5.4.1] | | |
| K01486 | 15 | adenine deaminase [EC:3.5.4.2] | | |
| K01487 | 12 | guanine deaminase [EC:3.5.4.3] | | |
| K01488 | 35 | adenosine deaminase [EC:3.5.4.4] | | |
| K01489 | 21 | cytidine deaminase [EC:3.5.4.5] | | |
| K01491 | 34 | methylenetetrahydrofolate dehydrogenase (NADP+) / | | |
| K01492 | 2 | phosphoribosylaminoimidazolecarboxamide formyltransferase | | |
| K01493 | 13 | dCMP deaminase [EC:3.5.4.12] | | |
| K01494 | 20 | dCTP deaminase [EC:3.5.4.13] | | |
| K01495 | 28 | GTP cyclohydrolase I [EC:3.5.4.16] | | |
| K01496 | 6 | phosphoribosyl-AMP cyclohydrolase [EC:3.5.4.19] | | |
| K01497 | 24 | GTP cyclohydrolase II [EC:3.5.4.25] | | |
| K01498 | 1 | diaminohydroxyphosphoribosylaminopyrimidine deaminase [EC:3.5.4.26] | | |
| K01499 | 3 | methenyltetrahydromethanopterin cyclohydrolase [EC:3.5.4.27] | | |
| K01500 | 18 | Unclassified; E3.5.4.- | | |
| K01501 | 28 | nitrilase [EC:3.5.5.1] | | |
| K01502 | 12 | aliphatic nitrilase [EC:3.5.5.7] | | |
| K01503 | 10 | Unclassified; E3.5.5.- | | |
| K01504 | 6 | glucosamine-6-phosphate isomerase [EC:3.5.99.6] | | |
| K01505 | 4 | 1-aminocyclopropane-1-carboxylate deaminase [EC:3.5.99.7] | | |
| K01506 | 64 | Unclassified; E3.5.-.- | | |
| K01507 | 54 | inorganic pyrophosphatase [EC:3.6.1.1] | | |
| K01512 | 16 | acylphosphatase [EC:3.6.1.7] | | |
| K01514 | 57 | exopolyphosphatase [EC:3.6.1.11] | | |
| K01515 | 41 | ADP-ribose pyrophosphatase [EC:3.6.1.13] | | |
| K01516 | 30 | nucleoside-triphosphatase [EC:3.6.1.15] | | |

| | | | | |
|---|---|---|---|---|
| K01518 | 13 | bis(5'-nucleosidyl)-tetraphosphatase [EC:3.6.1.17] | | |
| K01520 | 14 | dUTP pyrophosphatase [EC:3.6.1.23] | | |
| K01523 | 4 | phosphoribosyl-ATP pyrophosphohydrolase [EC:3.6.1.31] | | |
| K01524 | 18 | guanosine-5'-triphosphate,3'-diphosphate pyrophosphatase | | |
| K01525 | 13 | bis(5'-nucleosyl)-tetraphosphatase (symmetrical) [EC:3.6.1.41] | | |
| K01529 | 223 | Unclassified; E3.6.1.- | | |
| K01531 | 33 | Mg2+-importing ATPase [EC:3.6.3.2] | | |
| K01533 | 135 | Cu2+-exporting ATPase [EC:3.6.3.4] | | |
| K01534 | 28 | Cd2+/Zn2+-exporting ATPase [EC:3.6.3.3 3.6.3.5] | | |
| K01535 | 2 | H+-transporting ATPase [EC:3.6.3.6] | | |
| K01537 | 113 | Ca2+-transporting ATPase [EC:3.6.3.8] | | |
| K01539 | 6 | sodium/potassium-transporting ATPase subunit alpha [EC:3.6.3.9] | | |
| K01546 | 18 | K+-transporting ATPase ATPase A chain [EC:3.6.3.12] | | |
| K01547 | 19 | K+-transporting ATPase ATPase B chain [EC:3.6.3.12] | | |
| K01548 | 14 | K+-transporting ATPase ATPase C chain [EC:3.6.3.12] | | |
| K01551 | 26 | arsenite-transporting ATPase [EC:3.6.3.16] | | |
| K01552 | 141 | Unclassified; E3.6.3.- | | |
| K01554 | 4 | Unclassified; E3.6.-.- | | |
| K01555 | 12 | fumarylacetoacetase [EC:3.7.1.2] | | |
| K01556 | 30 | kynureninase [EC:3.7.1.3] | | |
| K01557 | 5 | acylpyruvate hydrolase [EC:3.7.1.5] | | |
| K01559 | 14 | Unclassified; E3.7.1.- | | |
| K01560 | 34 | 2-haloacid dehalogenase [EC:3.8.1.2] | | |
| K01561 | 20 | haloacetate dehalogenase [EC:3.8.1.3] | | |
| K01563 | 44 | haloalkane dehalogenase [EC:3.8.1.5] | | |
| K01564 | 47 | Unclassified; E3.8.1.- | | |
| K01567 | 169 | Unclassified; E3.-.-.- | | |
| K01568 | 1 | pyruvate decarboxylase [EC:4.1.1.1] | | |
| K01571 | 11 | oxaloacetate decarboxylase, alpha subunit [EC:4.1.1.3] | | |
| K01572 | 9 | oxaloacetate decarboxylase, beta subunit [EC:4.1.1.3] | | |
| K01576 | 25 | benzoylformate decarboxylase [EC:4.1.1.7] | | |
| K01577 | 9 | oxalyl-CoA decarboxylase [EC:4.1.1.8] | | |
| K01578 | 19 | malonyl-CoA decarboxylase [EC:4.1.1.9] | | |
| K01579 | 12 | aspartate 1-decarboxylase [EC:4.1.1.11] | | |
| K01580 | 8 | glutamate decarboxylase [EC:4.1.1.15] | | |
| K01581 | 17 | ornithine decarboxylase [EC:4.1.1.17] | | |
| K01582 | 22 | lysine decarboxylase [EC:4.1.1.18] | | |
| K01583 | 17 | arginine decarboxylase [EC:4.1.1.19] | | |
| K01584 | 17 | arginine decarboxylase [EC:4.1.1.19] | | |
| K01585 | 30 | arginine decarboxylase [EC:4.1.1.19] | | |
| K01586 | 61 | diaminopimelate decarboxylase [EC:4.1.1.20] | | |
| K01588 | 20 | 5-(carboxyamino)imidazole ribonucleotide mutase [EC:5.4.99.18] | | |
| K01589 | 16 | 5-(carboxyamino)imidazole ribonucleotide synthase [EC:6.3.4.18] | | |
| K01591 | 30 | orotidine-5'-phosphate decarboxylase [EC:4.1.1.23] | | |
| K01592 | 14 | tyrosine decarboxylase [EC:4.1.1.25] | | |
| K01593 | 61 | aromatic-L-amino-acid decarboxylase [EC:4.1.1.28] | | |
| K01594 | 14 | sulfinoalanine decarboxylase [EC:4.1.1.29] | | |
| K01595 | 40 | phosphoenolpyruvate carboxylase [EC:4.1.1.31] | | |
| K01596 | 30 | phosphoenolpyruvate carboxykinase (GTP) [EC:4.1.1.32] | | |
| K01598 | 9 | phosphopantothenoylcysteine decarboxylase [EC:4.1.1.36] | | |
| K01599 | 38 | uroporphyrinogen decarboxylase [EC:4.1.1.37] | | |
| K01601 | 7 | ribulose-bisphosphate carboxylase large chain [EC:4.1.1.39] | | |
| K01602 | 3 | ribulose-bisphosphate carboxylase small chain [EC:4.1.1.39] | | |
| K01605 | 1 | methylmalonyl-CoA decarboxylase beta chain [EC:4.1.1.41] | | |
| K01606 | 11 | methylmalonyl-CoA decarboxylase gamma chain [EC:4.1.1.41] | | |
| K01607 | 55 | 4-carboxymuconolactone decarboxylase [EC:4.1.1.44] | | |
| K01608 | 8 | tartronate-semialdehyde synthase [EC:4.1.1.47] | | |
| K01609 | 19 | indole-3-glycerol phosphate synthase [EC:4.1.1.48] | | |
| K01610 | 22 | phosphoenolpyruvate carboxykinase (ATP) [EC:4.1.1.49] | | |

| | | | | |
|---|---|---|---|---|
| K01611 | 4 | S-adenosylmethionine decarboxylase [EC:4.1.1.50] | | |
| K01612 | 5 | 4-hydroxybenzoate decarboxylase [EC:4.1.1.61] | | |
| K01613 | 14 | phosphatidylserine decarboxylase [EC:4.1.1.65] | | |
| K01615 | 12 | glutaconyl-CoA decarboxylase [EC:4.1.1.70] | | |
| K01616 | 9 | 2-oxoglutarate decarboxylase [EC:4.1.1.71] | | |
| K01617 | 5 | 4-oxalocrotonate decarboxylase [EC:4.1.1.77] | | |
| K01618 | 62 | Unclassified; E4.1.1.- | | |
| K01619 | 13 | deoxyribose-phosphate aldolase [EC:4.1.2.4] | | |
| K01620 | 32 | threonine aldolase [EC:4.1.2.5] | | |
| K01621 | 20 | phosphoketolase [EC:4.1.2.9] | | |
| K01623 | 28 | fructose-bisphosphate aldolase, class I [EC:4.1.2.13] | | |
| K01624 | 8 | fructose-bisphosphate aldolase, class II [EC:4.1.2.13] | | |
| K01625 | 11 | 2-dehydro-3-deoxyphosphogluconate aldolase / | | |
| K01626 | 26 | 3-deoxy-7-phosphoheptulonate synthase [EC:2.5.1.54] | | |
| K01627 | 29 | 2-dehydro-3-deoxyphosphooctonate aldolase (KDO 8-P synthase) | | |
| K01628 | 51 | L-fuculose-phosphate aldolase [EC:4.1.2.17] | | |
| K01629 | 6 | rhamnulose-1-phosphate aldolase [EC:4.1.2.19] | | |
| K01630 | 13 | 2-dehydro-3-deoxyglucarate aldolase [EC:4.1.2.20] | | |
| K01631 | 11 | 2-dehydro-3-deoxyphosphogalactonate aldolase [EC:4.1.2.21] | | |
| K01632 | 12 | fructose-6-phosphate phosphoketolase [EC:4.1.2.22] | | |
| K01633 | 13 | dihydroneopterin aldolase [EC:4.1.2.25] | | |
| K01634 | 7 | sphinganine-1-phosphate aldolase [EC:4.1.2.27] | | |
| K01635 | 10 | tagatose 1,6-diphosphate aldolase [EC:4.1.2.40] | | |
| K01636 | 37 | Unclassified; E4.1.2.- | | |
| K01637 | 13 | isocitrate lyase [EC:4.1.3.1] | | |
| K01638 | 23 | malate synthase [EC:2.3.3.9] | | |
| K01639 | 16 | N-acetylneuraminate lyase [EC:4.1.3.3] | | |
| K01640 | 23 | hydroxymethylglutaryl-CoA lyase [EC:4.1.3.4] | | |
| K01641 | 3 | hydroxymethylglutaryl-CoA synthase [EC:2.3.3.10] | | |
| K01643 | 11 | citrate lyase subunit alpha / citrate CoA-transferase [EC:4.1.3.6 | | |
| K01644 | 42 | citrate lyase subunit beta / citryl-CoA lyase [EC:4.1.3.6 4.1.3.34] | | |
| K01647 | 50 | citrate synthase [EC:2.3.3.1] | | |
| K01648 | 1 | ATP citrate (pro-S)-lyase [EC:2.3.3.8] | | |
| K01649 | 82 | 2-isopropylmalate synthase [EC:2.3.3.13] | | |
| K01652 | 178 | acetolactate synthase I/II/III large subunit [EC:2.2.1.6] | | |
| K01653 | 22 | acetolactate synthase I/III small subunit [EC:2.2.1.6] | | |
| K01654 | 14 | N-acetylneuraminate synthase [EC:2.5.1.56] | | |
| K01655 | 8 | homocitrate synthase [EC:2.3.3.14] | | |
| K01657 | 59 | anthranilate synthase component I [EC:4.1.3.27] | | |
| K01658 | 28 | anthranilate synthase component II [EC:4.1.3.27] | | |
| K01659 | 6 | 2-methylcitrate synthase [EC:2.3.3.5] | | |
| K01661 | 17 | naphthoate synthase [EC:4.1.3.36] | | |
| K01662 | 55 | 1-deoxy-D-xylulose-5-phosphate synthase [EC:2.2.1.7] | | |
| K01664 | 14 | para-aminobenzoate synthetase component II [EC:2.6.1.85] | | |
| K01665 | 22 | para-aminobenzoate synthetase component I [EC:2.6.1.85] | | |
| K01666 | 4 | 4-hydroxy 2-oxovalerate aldolase [EC:4.1.3.39] | | |
| K01667 | 15 | tryptophanase [EC:4.1.99.1] | | |
| K01668 | 12 | tyrosine phenol-lyase [EC:4.1.99.2] | | |
| K01669 | 28 | deoxyribodipyrimidine photo-lyase [EC:4.1.99.3] | | |
| K01673 | 27 | carbonic anhydrase [EC:4.2.1.1] | | |
| K01676 | 23 | fumarate hydratase, class I [EC:4.2.1.2] | | |
| K01677 | 4 | fumarate hydratase subunit alpha [EC:4.2.1.2] | | |
| K01679 | 37 | fumarate hydratase, class II [EC:4.2.1.2] | | |
| K01681 | 81 | aconitate hydratase 1 [EC:4.2.1.3] | | |
| K01682 | 7 | aconitate hydratase 2 [EC:4.2.1.3] | | |
| K01684 | 33 | galactonate dehydratase [EC:4.2.1.6] | | |
| K01685 | 24 | altronate hydrolase [EC:4.2.1.7] | | |
| K01686 | 9 | mannonate dehydratase [EC:4.2.1.8] | | |
| K01687 | 73 | dihydroxy-acid dehydratase [EC:4.2.1.9] | | |

| | | | | |
|---|---|---|---|---|
| K01689 | 42 | enolase [EC:4.2.1.11] | | |
| K01690 | 2 | phosphogluconate dehydratase [EC:4.2.1.12] | | |
| K01692 | 266 | enoyl-CoA hydratase [EC:4.2.1.17] | | |
| K01693 | 14 | imidazoleglycerol-phosphate dehydratase [EC:4.2.1.19] | | |
| K01695 | 29 | tryptophan synthase alpha chain [EC:4.2.1.20] | | |
| K01696 | 29 | tryptophan synthase beta chain [EC:4.2.1.20] | | |
| K01697 | 25 | cystathionine beta-synthase [EC:4.2.1.22] | | |
| K01698 | 18 | porphobilinogen synthase [EC:4.2.1.24] | | |
| K01699 | 4 | propanediol dehydratase large subunit [EC:4.2.1.28] | | |
| K01703 | 59 | 3-isopropylmalate/(R)-2-methylmalate dehydratase large subunit | | |
| K01704 | 24 | 3-isopropylmalate/(R)-2-methylmalate dehydratase small subunit | | |
| K01705 | 1 | homoaconitate hydratase [EC:4.2.1.36] | | |
| K01706 | 7 | glucarate dehydratase [EC:4.2.1.40] | | |
| K01708 | 22 | galactarate dehydratase [EC:4.2.1.42] | | |
| K01709 | 15 | CDP-glucose 4,6-dehydratase [EC:4.2.1.45] | | |
| K01710 | 121 | dTDP-glucose 4,6-dehydratase [EC:4.2.1.46] | | |
| K01711 | 34 | GDPmannose 4,6-dehydratase [EC:4.2.1.47] | | |
| K01712 | 17 | urocanate hydratase [EC:4.2.1.49] | | |
| K01713 | 19 | prephenate dehydratase [EC:4.2.1.51] | | |
| K01714 | 67 | dihydrodipicolinate synthase [EC:4.2.1.52] | | |
| K01715 | 61 | 3-hydroxybutyryl-CoA dehydratase [EC:4.2.1.55] | | |
| K01716 | 1 | 3-hydroxydecanoyl-[acyl-carrier-protein] dehydratase [EC:4.2.1.60] | | |
| K01718 | 4 | pseudouridylate synthase [EC:4.2.1.70] | | |
| K01719 | 22 | uroporphyrinogen-III synthase [EC:4.2.1.75] | | |
| K01720 | 63 | 2-methylcitrate dehydratase [EC:4.2.1.79] | | |
| K01722 | 3 | Unclassified; E4.2.1.89 | | |
| K01724 | 20 | 4a-hydroxytetrahydrobiopterin dehydratase [EC:4.2.1.96] | | |
| K01725 | 1 | cyanate lyase [EC:4.2.1.104] | | |
| K01726 | 99 | Unclassified; E4.2.1.- | | |
| K01728 | 1 | pectate lyase [EC:4.2.2.2] | | |
| K01733 | 66 | threonine synthase [EC:4.2.3.1] | | |
| K01734 | 6 | methylglyoxal synthase [EC:4.2.3.3] | | |
| K01735 | 30 | 3-dehydroquinate synthase [EC:4.2.3.4] | | |
| K01736 | 40 | chorismate synthase [EC:4.2.3.5] | | |
| K01737 | 25 | 6-pyruvoyl tetrahydrobiopterin synthase [EC:4.2.3.12] | | |
| K01738 | 73 | cysteine synthase A [EC:2.5.1.47] | | |
| K01739 | 49 | cystathionine gamma-synthase [EC:2.5.1.48] | | |
| K01740 | 36 | O-acetylhomoserine (thiol)-lyase [EC:2.5.1.49] | | |
| K01741 | 3 | DNA-(apurinic or apyrimidinic site) lyase [EC:4.2.99.18] | | |
| K01743 | 3 | Unclassified; E4.2.-.- | | |
| K01744 | 18 | aspartate ammonia-lyase [EC:4.3.1.1] | | |
| K01745 | 32 | histidine ammonia-lyase [EC:4.3.1.3] | | |
| K01746 | 14 | formiminotetrahydrofolate cyclodeaminase [EC:4.3.1.4] | | |
| K01749 | 28 | hydroxymethylbilane synthase [EC:2.5.1.61] | | |
| K01750 | 39 | ornithine cyclodeaminase [EC:4.3.1.12] | | |
| K01751 | 4 | diaminopropionate ammonia-lyase [EC:4.3.1.15] | | |
| K01752 | 8 | L-serine dehydratase [EC:4.3.1.17] | | |
| K01754 | 80 | threonine dehydratase [EC:4.3.1.19] | | |
| K01755 | 59 | argininosuccinate lyase [EC:4.3.2.1] | | |
| K01756 | 53 | adenylosuccinate lyase [EC:4.3.2.2] | | |
| K01757 | 6 | strictosidine synthase [EC:4.3.3.2] | | |
| K01758 | 28 | cystathionine gamma-lyase [EC:4.4.1.1] | | |
| K01759 | 62 | lactoylglutathione lyase [EC:4.4.1.5] | | |
| K01760 | 59 | cystathionine beta-lyase [EC:4.4.1.8] | | |
| K01761 | 21 | methionine-gamma-lyase [EC:4.4.1.11] | | |
| K01768 | 403 | adenylate cyclase [EC:4.6.1.1] | | |
| K01769 | 7 | guanylate cyclase, other [EC:4.6.1.2] | | |
| K01770 | 23 | 2-C-methyl-D-erythritol 2,4-cyclodiphosphate synthase [EC:4.6.1.12] | | |
| K01772 | 32 | ferrochelatase [EC:4.99.1.1] | | |

| | | | | |
|---|---|---|---|---|
| K01774 | 3 | Unclassified; E4.-.-.- | | |
| K01775 | 45 | alanine racemase [EC:5.1.1.1] | | |
| K01776 | 47 | glutamate racemase [EC:5.1.1.3] | | |
| K01777 | 12 | proline racemase [EC:5.1.1.4] | | |
| K01778 | 33 | diaminopimelate epimerase [EC:5.1.1.7] | | |
| K01779 | 30 | aspartate racemase [EC:5.1.1.13] | | |
| K01780 | 26 | Unclassified; E5.1.1.- | | |
| K01781 | 68 | mandelate racemase [EC:5.1.2.2] | | |
| K01782 | 36 | 3-hydroxyacyl-CoA dehydrogenase / enoyl-CoA hydratase / | | |
| K01783 | 21 | ribulose-phosphate 3-epimerase [EC:5.1.3.1] | | |
| K01784 | 233 | UDP-glucose 4-epimerase [EC:5.1.3.2] | | |
| K01785 | 23 | aldose 1-epimerase [EC:5.1.3.3] | | |
| K01786 | 5 | L-ribulose-5-phosphate 4-epimerase [EC:5.1.3.4] | | |
| K01787 | 7 | N-acylglucosamine 2-epimerase [EC:5.1.3.8] | | |
| K01789 | 11 | UDP-glucuronate 5'-epimerase [EC:5.1.3.12] | | |
| K01790 | 33 | dTDP-4-dehydrorhamnose 3,5-epimerase [EC:5.1.3.13] | | |
| K01791 | 43 | UDP-N-acetylglucosamine 2-epimerase [EC:5.1.3.14] | | |
| K01792 | 3 | glucose-6-phosphate 1-epimerase [EC:5.1.3.15] | | |
| K01795 | 128 | Unclassified; E5.1.3.- | | |
| K01796 | 35 | alpha-methylacyl-CoA racemase [EC:5.1.99.4] | | |
| K01797 | 89 | Unclassified; E5.1.99.- | | |
| K01798 | 3 | Unclassified; E5.1.-.- | | |
| K01799 | 14 | maleate isomerase [EC:5.2.1.1] | | |
| K01800 | 11 | maleylacetoacetate isomerase [EC:5.2.1.2] | | |
| K01801 | 8 | maleylpyruvate isomerase [EC:5.2.1.4] | | |
| K01802 | 87 | peptidylprolyl isomerase [EC:5.2.1.8] | | |
| K01803 | 29 | triosephosphate isomerase (TIM) [EC:5.3.1.1] | | |
| K01804 | 10 | L-arabinose isomerase [EC:5.3.1.4] | | |
| K01805 | 13 | xylose isomerase [EC:5.3.1.5] | | |
| K01807 | 12 | ribose 5-phosphate isomerase A [EC:5.3.1.6] | | |
| K01808 | 26 | ribose 5-phosphate isomerase B [EC:5.3.1.6] | | |
| K01809 | 44 | mannose-6-phosphate isomerase [EC:5.3.1.8] | | |
| K01810 | 53 | glucose-6-phosphate isomerase [EC:5.3.1.9] | | |
| K01811 | 10 | putative family 31 glucosidase | | |
| K01812 | 7 | glucuronate isomerase [EC:5.3.1.12] | | |
| K01813 | 9 | L-rhamnose isomerase [EC:5.3.1.14] | | |
| K01814 | 30 | phosphoribosylformimino-5-aminoimidazole carboxamide ribotide | | |
| K01816 | 12 | hydroxypyruvate isomerase [EC:5.3.1.22] | | |
| K01817 | 27 | phosphoribosylanthranilate isomerase [EC:5.3.1.24] | | |
| K01818 | 6 | L-fucose isomerase [EC:5.3.1.25] | | |
| K01820 | 9 | Unclassified; E5.3.1.- | | |
| K01821 | 5 | 4-oxalocrotonate tautomerase [EC:5.3.2.-] | | |
| K01822 | 1 | steroid delta-isomerase [EC:5.3.3.1] | | |
| K01823 | 12 | isopentenyl-diphosphate delta-isomerase [EC:5.3.3.2] | | |
| K01825 | 4 | 3-hydroxyacyl-CoA dehydrogenase / enoyl-CoA hydratase / | | |
| K01826 | 20 | 5-carboxymethyl-2-hydroxymuconate isomerase [EC:5.3.3.10] | | |
| K01828 | 20 | Unclassified; E5.3.3.- | | |
| K01829 | 20 | protein disulfide-isomerase [EC:5.3.4.1] | | |
| K01834 | 100 | phosphoglycerate mutase [EC:5.4.2.1] | | |
| K01835 | 41 | phosphoglucomutase [EC:5.4.2.2] | | |
| K01838 | 4 | beta-phosphoglucomutase [EC:5.4.2.6] | | |
| K01839 | 18 | phosphopentomutase [EC:5.4.2.7] | | |
| K01840 | 87 | phosphomannomutase [EC:5.4.2.8] | | |
| K01841 | 3 | phosphoenolpyruvate phosphomutase [EC:5.4.2.9] | | |
| K01842 | 5 | Unclassified; E5.4.2.- | | |
| K01843 | 25 | lysine 2,3-aminomutase [EC:5.4.3.2] | | |
| K01844 | 3 | beta-lysine 5,6-aminomutase [EC:5.4.3.3] | | |
| K01845 | 65 | glutamate-1-semialdehyde 2,1-aminomutase [EC:5.4.3.8] | | |
| K01846 | 4 | methylaspartate mutase [EC:5.4.99.1] | | |

| | | | | |
|---|---|---|---|---|
| K01847 | 14 | methylmalonyl-CoA mutase [EC:5.4.99.2] | | |
| K01848 | 52 | methylmalonyl-CoA mutase, N-terminal domain [EC:5.4.99.2] | | |
| K01849 | 12 | methylmalonyl-CoA mutase, C-terminal domain [EC:5.4.99.2] | | |
| K01851 | 1 | salicylate biosynthesis isochorismate synthase [EC:5.4.4.2] | | |
| K01854 | 46 | UDP-galactopyranose mutase [EC:5.4.99.9] | | |
| K01856 | 36 | muconate cycloisomerase [EC:5.5.1.1] | | |
| K01857 | 15 | 3-carboxy-cis,cis-muconate cycloisomerase [EC:5.5.1.2] | | |
| K01858 | 12 | myo-inositol-1-phosphate synthase [EC:5.5.1.4] | | |
| K01860 | 15 | chloromuconate cycloisomerase [EC:5.5.1.7] | | |
| K01865 | 17 | Unclassified; E5.-.-.- | | |
| K01866 | 51 | tyrosyl-tRNA synthetase [EC:6.1.1.1] | | |
| K01867 | 46 | tryptophanyl-tRNA synthetase [EC:6.1.1.2] | | |
| K01868 | 67 | threonyl-tRNA synthetase [EC:6.1.1.3] | | |
| K01869 | 73 | leucyl-tRNA synthetase [EC:6.1.1.4] | | |
| K01870 | 85 | isoleucyl-tRNA synthetase [EC:6.1.1.5] | | |
| K01872 | 71 | alanyl-tRNA synthetase [EC:6.1.1.7] | | |
| K01873 | 99 | valyl-tRNA synthetase [EC:6.1.1.9] | | |
| K01874 | 56 | methionyl-tRNA synthetase [EC:6.1.1.10] | | |
| K01875 | 59 | seryl-tRNA synthetase [EC:6.1.1.11] | | |
| K01876 | 57 | aspartyl-tRNA synthetase [EC:6.1.1.12] | | |
| K01878 | 12 | glycyl-tRNA synthetase alpha chain [EC:6.1.1.14] | | |
| K01879 | 36 | glycyl-tRNA synthetase beta chain [EC:6.1.1.14] | | |
| K01880 | 17 | glycyl-tRNA synthetase [EC:6.1.1.14] | | |
| K01881 | 50 | prolyl-tRNA synthetase [EC:6.1.1.15] | | |
| K01883 | 69 | cysteinyl-tRNA synthetase [EC:6.1.1.16] | | |
| K01885 | 74 | glutamyl-tRNA synthetase [EC:6.1.1.17] | | |
| K01886 | 28 | glutaminyl-tRNA synthetase [EC:6.1.1.18] | | |
| K01887 | 498 | arginyl-tRNA synthetase [EC:6.1.1.19] | | |
| K01889 | 40 | phenylalanyl-tRNA synthetase alpha chain [EC:6.1.1.20] | | |
| K01890 | 515 | phenylalanyl-tRNA synthetase beta chain [EC:6.1.1.20] | | |
| K01892 | 46 | histidyl-tRNA synthetase [EC:6.1.1.21] | | |
| K01893 | 25 | asparaginyl-tRNA synthetase [EC:6.1.1.22] | | |
| K01894 | 20 | glutamyl-Q tRNA(Asp) synthetase [EC:6.1.1.-] | | |
| K01895 | 175 | acetyl-CoA synthetase [EC:6.2.1.1] | | |
| K01897 | 294 | long-chain acyl-CoA synthetase [EC:6.2.1.3] | | |
| K01902 | 19 | succinyl-CoA synthetase alpha subunit [EC:6.2.1.5] | | |
| K01903 | 30 | succinyl-CoA synthetase beta subunit [EC:6.2.1.5] | | |
| K01904 | 37 | 4-coumarate--CoA ligase [EC:6.2.1.12] | | |
| K01905 | 37 | acetyl-CoA synthetase (ADP-forming) [EC:6.2.1.13] | | |
| K01906 | 43 | 6-carboxyhexanoate--CoA ligase [EC:6.2.1.14] | | |
| K01907 | 38 | acetoacetyl-CoA synthetase [EC:6.2.1.16] | | |
| K01908 | 12 | propionyl-CoA synthetase [EC:6.2.1.17] | | |
| K01911 | 22 | O-succinylbenzoic acid--CoA ligase [EC:6.2.1.26] | | |
| K01912 | 88 | phenylacetate-CoA ligase [EC:6.2.1.30] | | |
| K01913 | 125 | Unclassified; E6.2.1.- | | |
| K01914 | 7 | aspartate--ammonia ligase [EC:6.3.1.1] | | |
| K01915 | 141 | glutamine synthetase [EC:6.3.1.2] | | |
| K01916 | 33 | NAD+ synthase [EC:6.3.1.5] | | |
| K01917 | 15 | glutathionylspermidine synthase [EC:6.3.1.8] | | |
| K01918 | 21 | pantoate--beta-alanine ligase [EC:6.3.2.1] | | |
| K01919 | 16 | glutamate--cysteine ligase [EC:6.3.2.2] | | |
| K01920 | 20 | glutathione synthase [EC:6.3.2.3] | | |
| K01921 | 88 | D-alanine-D-alanine ligase [EC:6.3.2.4] | | |
| K01922 | 6 | phosphopantothenate-cysteine ligase [EC:6.3.2.5] | | |
| K01923 | 35 | phosphoribosylaminoimidazole-succinocarboxamide synthase | | |
| K01924 | 35 | UDP-N-acetylmuramate--alanine ligase [EC:6.3.2.8] | | |
| K01925 | 33 | UDP-N-acetylmuramoylalanine--D-glutamate ligase [EC:6.3.2.9] | | |
| K01926 | 7 | AT-rich DNA-binding protein | | |
| K01928 | 42 | UDP-N-acetylmuramoylalanyl-D-glutamate--2,6-diaminopimelate ligase | | |

| | | | | |
|---|---|---|---|---|
| K01929 | 58 | UDP-N-acetylmuramoylalanyl-D-glutamyl-2,6-diaminopimelate--D-alanyl- | | |
| K01932 | 7 | Unclassified; E6.3.2.- | | |
| K01933 | 32 | phosphoribosylformylglycinamidine cyclo-ligase [EC:6.3.3.1] | | |
| K01934 | 15 | 5-formyltetrahydrofolate cyclo-ligase [EC:6.3.3.2] | | |
| K01935 | 11 | dethiobiotin synthetase [EC:6.3.3.3] | | |
| K01937 | 42 | CTP synthase [EC:6.3.4.2] | | |
| K01938 | 23 | formate--tetrahydrofolate ligase [EC:6.3.4.3] | | |
| K01939 | 47 | adenylosuccinate synthase [EC:6.3.4.4] | | |
| K01940 | 34 | argininosuccinate synthase [EC:6.3.4.5] | | |
| K01941 | 8 | urea carboxylase [EC:6.3.4.6] | | |
| K01945 | 31 | phosphoribosylamine--glycine ligase [EC:6.3.4.13] | | |
| K01947 | 4 | biotin-[acetyl-CoA-carboxylase] ligase / type III pantothenate | | |
| K01950 | 63 | NAD+ synthase (glutamine-hydrolysing) [EC:6.3.5.1] | | |
| K01951 | 50 | GMP synthase (glutamine-hydrolysing) [EC:6.3.5.2] | | |
| K01952 | 89 | phosphoribosylformylglycinamidine synthase [EC:6.3.5.3] | | |
| K01953 | 160 | asparagine synthase (glutamine-hydrolysing) [EC:6.3.5.4] | | |
| K01955 | 66 | carbamoyl-phosphate synthase large subunit [EC:6.3.5.5] | | |
| K01956 | 39 | carbamoyl-phosphate synthase small subunit [EC:6.3.5.5] | | |
| K01957 | 10 | glutamyl-tRNA (Gln) amidotransferase [EC:6.3.5.-] | | |
| K01958 | 22 | pyruvate carboxylase [EC:6.4.1.1] | | |
| K01959 | 2 | pyruvate carboxylase subunit A [EC:6.4.1.1] | | |
| K01960 | 23 | pyruvate carboxylase subunit B [EC:6.4.1.1] | | |
| K01961 | 38 | acetyl-CoA carboxylase, biotin carboxylase subunit [EC:6.4.1.2 | | |
| K01962 | 21 | acetyl-CoA carboxylase carboxyl transferase subunit alpha | | |
| K01963 | 31 | acetyl-CoA carboxylase carboxyl transferase subunit beta | | |
| K01965 | 36 | propionyl-CoA carboxylase alpha chain [EC:6.4.1.3] | | |
| K01966 | 69 | propionyl-CoA carboxylase beta chain [EC:6.4.1.3] | | |
| K01968 | 21 | 3-methylcrotonyl-CoA carboxylase alpha subunit [EC:6.4.1.4] | | |
| K01969 | 19 | 3-methylcrotonyl-CoA carboxylase beta subunit [EC:6.4.1.4] | | |
| K01970 | 3 | Unclassified; E6.4.-.- | | |
| K01971 | 172 | DNA ligase (ATP) [EC:6.5.1.1] | | |
| K01972 | 63 | DNA ligase (NAD+) [EC:6.5.1.2] | | |
| K01974 | 6 | RNA 3'-terminal phosphate cyclase [EC:6.5.1.4] | | |
| K01975 | 8 | 2'-5' RNA ligase [EC:6.5.1.-] | | |
| K01976 | 26 | Unclassified; E6.-.-.- | | |
| K01989 | 47 | putative ABC transport system substrate-binding protein | | |
| K01990 | 199 | ABC-2 type transport system ATP-binding protein | | |
| K01991 | 50 | polysaccharide export outer membrane protein | | |
| K01992 | 215 | ABC-2 type transport system permease protein | | |
| K01993 | 36 | HlyD family secretion protein | | |
| K01994 | 1 | LuxR family transcriptional regulator, transcriptional regulator of | | |
| K01995 | 126 | branched-chain amino acid transport system ATP-binding protein | | |
| K01996 | 125 | branched-chain amino acid transport system ATP-binding protein | | |
| K01997 | 153 | branched-chain amino acid transport system permease protein | | |
| K01998 | 326 | branched-chain amino acid transport system permease protein | | |
| K01999 | 254 | branched-chain amino acid transport system substrate-binding protein | | |
| K02000 | 5 | glycine betaine/proline transport system ATP-binding protein | | |
| K02001 | 2 | glycine betaine/proline transport system permease protein | | |
| K02002 | 2 | glycine betaine/proline transport system substrate-binding protein | | |
| K02003 | 126 | Unclassified; ABC.CD.A | | |
| K02004 | 274 | Unclassified; ABC.CD.P | | |
| K02005 | 128 | HlyD family secretion protein | | |
| K02006 | 28 | cobalt/nickel transport system ATP-binding protein | | |
| K02007 | 5 | cobalt/nickel transport system permease protein | | |
| K02008 | 17 | cobalt/nickel transport system permease protein | | |
| K02010 | 19 | iron(III) transport system ATP-binding protein [EC:3.6.3.30] | | |
| K02011 | 71 | iron(III) transport system permease protein | | |
| K02012 | 46 | iron(III) transport system substrate-binding protein | | |
| K02013 | 25 | iron complex transport system ATP-binding protein [EC:3.6.3.34] | | |

| | | | | |
|---|---|---|---|---|
| K02014 | 205 | iron complex outermembrane recepter protein | | |
| K02015 | 23 | iron complex transport system permease protein | | |
| K02016 | 43 | iron complex transport system substrate-binding protein | | |
| K02017 | 11 | molybdate transport system ATP-binding protein [EC:3.6.3.29] | | |
| K02018 | 15 | molybdate transport system permease protein | | |
| K02019 | 7 | molybdate transport system regulatory protein | | |
| K02020 | 55 | molybdate transport system substrate-binding protein | | |
| K02021 | 41 | putative ABC transport system ATP-binding protein | | |
| K02022 | 18 | Unclassified; ABC.MR.TX | | |
| K02023 | 24 | multiple sugar transport system ATP-binding protein | | |
| K02024 | 1 | maltoporin | | |
| K02025 | 139 | multiple sugar transport system permease protein | | |
| K02026 | 137 | multiple sugar transport system permease protein | | |
| K02027 | 138 | multiple sugar transport system substrate-binding protein | | |
| K02028 | 18 | polar amino acid transport system ATP-binding protein [EC:3.6.3.21] | | |
| K02029 | 26 | polar amino acid transport system permease protein | | |
| K02030 | 96 | polar amino acid transport system substrate-binding protein | | |
| K02031 | 66 | peptide/nickel transport system ATP-binding protein | | |
| K02032 | 185 | peptide/nickel transport system ATP-binding protein | | |
| K02033 | 207 | peptide/nickel transport system permease protein | | |
| K02034 | 165 | peptide/nickel transport system permease protein | | |
| K02035 | 407 | peptide/nickel transport system substrate-binding protein | | |
| K02036 | 24 | phosphate transport system ATP-binding protein [EC:3.6.3.27] | | |
| K02037 | 43 | phosphate transport system permease protein | | |
| K02038 | 42 | phosphate transport system permease protein | | |
| K02039 | 31 | phosphate transport system protein | | |
| K02040 | 62 | phosphate transport system substrate-binding protein | | |
| K02041 | 11 | phosphonate transport system ATP-binding protein | | |
| K02042 | 4 | phosphonate transport system permease protein | | |
| K02044 | 24 | phosphonate transport system substrate-binding protein | | |
| K02045 | 11 | sulfate transport system ATP-binding protein [EC:3.6.3.25] | | |
| K02046 | 14 | sulfate transport system permease protein | | |
| K02047 | 9 | sulfate transport system permease protein | | |
| K02048 | 16 | sulfate transport system substrate-binding protein | | |
| K02049 | 46 | sulfonate/nitrate/taurine transport system ATP-binding protein | | |
| K02050 | 86 | sulfonate/nitrate/taurine transport system permease protein | | |
| K02051 | 109 | sulfonate/nitrate/taurine transport system substrate-binding protein | | |
| K02052 | 32 | putative spermidine/putrescine transport system ATP-binding protein | | |
| K02053 | 39 | putative spermidine/putrescine transport system permease protein | | |
| K02054 | 31 | putative spermidine/putrescine transport system permease protein | | |
| K02055 | 46 | putative spermidine/putrescine transport system substrate-binding | | |
| K02056 | 66 | simple sugar transport system ATP-binding protein [EC:3.6.3.17] | | |
| K02057 | 106 | simple sugar transport system permease protein | | |
| K02058 | 54 | simple sugar transport system substrate-binding protein | | |
| K02059 | 4 | putative sulfate transport system ATP-binding protein | | |
| K02060 | 7 | putative sulfate transport system permease protein | | |
| K02061 | 3 | putative sulfate transport system substrate-binding protein | | |
| K02063 | 7 | thiamine transport system permease protein | | |
| K02064 | 3 | thiamine transport system substrate-binding protein | | |
| K02065 | 37 | putative ABC transport system ATP-binding protein | | |
| K02066 | 51 | putative ABC transport system permease protein | | |
| K02067 | 57 | putative ABC transport system substrate-binding protein | | |
| K02068 | 1 | putative ABC transport system ATP-binding protein | | |
| K02069 | 5 | putative ABC transport system permease protein | | |
| K02071 | 4 | D-methionine transport system ATP-binding protein | | |
| K02073 | 2 | D-methionine transport system substrate-binding protein | | |
| K02074 | 9 | zinc/manganese transport system ATP-binding protein | | |
| K02075 | 17 | zinc/manganese transport system permease protein | | |
| K02076 | 2 | Fur family transcriptional regulator, zinc uptake regulator | | |

| | | | | |
|---|---|---|---|---|
| K02077 | 30 | zinc/manganese transport system substrate-binding protein | | |
| K02078 | 15 | acyl carrier protein | | |
| K02079 | 2 | N-acetylgalactosamine-6-phosphate deacetylase [EC:3.5.1.25] | | |
| K02081 | 13 | DeoR family transcriptional regulator, aga operon transcriptional | | |
| K02082 | 2 | tagatose-6-phosphate ketose/aldose isomerase [EC:5.-.-.-] | | |
| K02083 | 5 | allantoate deiminase [EC:3.5.3.9] | | |
| K02099 | 3 | AraC family transcriptional regulator, arabinose operon regulatory | | |
| K02103 | 1 | GntR family transcriptional regulator, arabinose operon | | |
| K02106 | 4 | short-chain fatty acids transporter | | |
| K02108 | 50 | F-type H+-transporting ATPase subunit a [EC:3.6.3.14] | | |
| K02109 | 12 | F-type H+-transporting ATPase subunit b [EC:3.6.3.14] | | |
| K02110 | 10 | F-type H+-transporting ATPase subunit c [EC:3.6.3.14] | | |
| K02111 | 32 | F-type H+-transporting ATPase subunit alpha [EC:3.6.3.14] | | |
| K02112 | 21 | F-type H+-transporting ATPase subunit beta [EC:3.6.3.14] | | |
| K02113 | 26 | F-type H+-transporting ATPase subunit delta [EC:3.6.3.14] | | |
| K02114 | 7 | F-type H+-transporting ATPase subunit epsilon [EC:3.6.3.14] | | |
| K02115 | 35 | F-type H+-transporting ATPase subunit gamma [EC:3.6.3.14] | | |
| K02116 | 2 | ATP synthase protein I | | |
| K02117 | 20 | V-type H+-transporting ATPase subunit A [EC:3.6.3.14] | | |
| K02118 | 13 | V-type H+-transporting ATPase subunit B [EC:3.6.3.14] | | |
| K02120 | 3 | V-type H+-transporting ATPase subunit D [EC:3.6.3.14] | | |
| K02121 | 2 | V-type H+-transporting ATPase subunit E [EC:3.6.3.14] | | |
| K02123 | 15 | V-type H+-transporting ATPase subunit I [EC:3.6.3.14] | | |
| K02124 | 4 | V-type H+-transporting ATPase subunit K [EC:3.6.3.14] | | |
| K02160 | 11 | acetyl-CoA carboxylase biotin carboxyl carrier protein | | |
| K02164 | 4 | nitric-oxide reductase NorE protein [EC:1.7.99.7] | | |
| K02169 | 17 | biotin synthesis protein BioC | | |
| K02170 | 4 | biotin biosynthesis protein BioH | | |
| K02182 | 16 | crotonobetaine/carnitine-CoA ligase [EC:6.2.1.-] | | |
| K02188 | 4 | cobalamin biosynthesis protein CbiD | | |
| K02189 | 1 | cobalamin biosynthesis protein CbiG | | |
| K02193 | 29 | heme exporter protein A [EC:3.6.3.41] | | |
| K02194 | 15 | heme exporter protein B | | |
| K02195 | 42 | heme exporter protein C | | |
| K02197 | 10 | cytochrome c-type biogenesis protein CcmE | | |
| K02198 | 81 | cytochrome c-type biogenesis protein CcmF | | |
| K02199 | 52 | cytochrome c biogenesis protein CcmG, thiol:disulfide interchange | | |
| K02200 | 19 | cytochrome c-type biogenesis protein CcmH | | |
| K02203 | 6 | phosphoserine / homoserine phosphotransferase [EC:3.1.3.3 2.7.1.39] | | |
| K02204 | 5 | homoserine kinase type II [EC:2.7.1.39] | | |
| K02217 | 12 | ferritin [EC:1.16.3.1] | | |
| K02221 | 26 | YggT family protein | | |
| K02224 | 18 | cobyrinic acid a,c-diamide synthase [EC:6.3.5.9 6.3.1.-] | | |
| K02226 | 2 | alpha-ribazole phosphatase [EC:3.1.3.73] | | |
| K02227 | 11 | adenosylcobinamide-phosphate synthase CobD [EC:6.3.1.10] | | |
| K02229 | 1 | precorrin-3B synthase [EC:1.14.13.83] | | |
| K02230 | 13 | cobaltochelatase CobN [EC:6.6.1.2] | | |
| K02231 | 13 | adenosylcobinamide kinase / adenosylcobinamide-phosphate | | |
| K02232 | 9 | adenosylcobyric acid synthase [EC:6.3.5.10] | | |
| K02233 | 2 | adenosylcobinamide-GDP ribazoletransferase [EC:2.7.8.26] | | |
| K02234 | 13 | cobalamin biosynthesis protein CobW | | |
| K02236 | 9 | leader peptidase (prepilin peptidase) / N-methyltransferase | | |
| K02237 | 16 | competence protein ComEA | | |
| K02238 | 57 | competence protein ComEC | | |
| K02240 | 1 | competence protein ComFA | | |
| K02242 | 4 | competence protein ComFC | | |
| K02244 | 3 | competence protein ComGB | | |
| K02248 | 3 | competence protein ComGF | | |
| K02258 | 6 | cytochrome c oxidase subunit XI assembly protein | | |

| | | | | |
|---|---|---|---|---|
| K02259 | 27 | cytochrome c oxidase subunit XV assembly protein | | |
| K02274 | 88 | cytochrome c oxidase subunit I [EC:1.9.3.1] | | |
| K02275 | 66 | cytochrome c oxidase subunit II [EC:1.9.3.1] | | |
| K02276 | 37 | cytochrome c oxidase subunit III [EC:1.9.3.1] | | |
| K02278 | 1 | prepilin peptidase CpaA [EC:3.4.23.43] | | |
| K02279 | 5 | pilus assembly protein CpaB | | |
| K02280 | 15 | pilus assembly protein CpaC | | |
| K02282 | 37 | pilus assembly protein CpaE | | |
| K02283 | 46 | pilus assembly protein CpaF | | |
| K02287 | 2 | phycocyanin-associated, rod | | |
| K02288 | 2 | phycocyanobilin lyase alpha subunit [EC:4.-.-.-] | | |
| K02291 | 30 | phytoene synthase [EC:2.5.1.32] | | |
| K02292 | 50 | beta-carotene ketolase (CrtO type) | | |
| K02293 | 3 | phytoene dehydrogenase, phytoene desaturase [EC:1.14.99.-] | | |
| K02297 | 3 | cytochrome o ubiquinol oxidase subunit II [EC:1.10.3.-] | | |
| K02298 | 6 | cytochrome o ubiquinol oxidase subunit I [EC:1.10.3.-] | | |
| K02299 | 4 | cytochrome o ubiquinol oxidase subunit III [EC:1.10.3.-] | | |
| K02301 | 31 | protoheme IX farnesyltransferase [EC:2.5.1.-] | | |
| K02302 | 27 | uroporphyrin-III C-methyltransferase / precorrin-2 dehydrogenase / | | |
| K02303 | 22 | uroporphyrin-III C-methyltransferase [EC:2.1.1.107] | | |
| K02304 | 6 | precorrin-2 dehydrogenase / sirohydrochlorin ferrochelatase | | |
| K02305 | 3 | nitric-oxide reductase, cytochrome c-containing subunit II | | |
| K02312 | 5 | 2,3-dihydroxybenzoate-AMP ligase [EC:2.7.7.58] | | |
| K02313 | 51 | chromosomal replication initiator protein | | |
| K02314 | 78 | replicative DNA helicase [EC:3.6.1.-] | | |
| K02315 | 8 | DNA replication protein DnaC | | |
| K02316 | 65 | DNA primase [EC:2.7.7.-] | | |
| K02319 | 11 | DNA polymerase I [EC:2.7.7.7] | | |
| K02330 | 6 | DNA polymerase beta subunit [EC:2.7.7.7 4.2.99.-] | | |
| K02334 | 43 | DNA polymerase bacteriophage-type [EC:2.7.7.7] | | |
| K02335 | 90 | DNA polymerase I [EC:2.7.7.7] | | |
| K02336 | 4 | DNA polymerase II [EC:2.7.7.7] | | |
| K02337 | 211 | DNA polymerase III subunit alpha [EC:2.7.7.7] | | |
| K02338 | 59 | DNA polymerase III subunit beta [EC:2.7.7.7] | | |
| K02339 | 6 | DNA polymerase III subunit chi [EC:2.7.7.7] | | |
| K02340 | 11 | DNA polymerase III subunit delta [EC:2.7.7.7] | | |
| K02341 | 35 | DNA polymerase III subunit delta' [EC:2.7.7.7] | | |
| K02342 | 60 | DNA polymerase III subunit epsilon [EC:2.7.7.7] | | |
| K02343 | 65 | DNA polymerase III subunit gamma/tau [EC:2.7.7.7] | | |
| K02345 | 1 | DNA polymerase III subunit theta [EC:2.7.7.7] | | |
| K02346 | 46 | DNA polymerase IV [EC:2.7.7.7] | | |
| K02347 | 45 | DNA polymerase (family X) | | |
| K02348 | 2 | ElaA protein | | |
| K02351 | 7 | putative membrane protein | | |
| K02352 | 1 | drp35 | | |
| K02355 | 154 | elongation factor EF-G [EC:3.6.5.3] | | |
| K02356 | 32 | elongation factor EF-P | | |
| K02357 | 44 | elongation factor EF-Ts | | |
| K02358 | 138 | elongation factor EF-Tu [EC:3.6.5.3] | | |
| K02361 | 19 | isochorismate synthase [EC:5.4.4.2] | | |
| K02363 | 1 | enterobactin 2,3-dihydroxybenzoate-AMP ligase / | | |
| K02364 | 7 | enterobactin synthetase component F [EC:2.7.7.-] | | |
| K02371 | 8 | enoyl-[acyl carrier protein] reductase II [EC:1.3.1.-] | | |
| K02372 | 22 | 3R-hydroxymyristoyl ACP dehydrase [EC:4.2.1.-] | | |
| K02377 | 24 | GDP-L-fucose synthase [EC:1.1.1.271] | | |
| K02379 | 19 | FdhD protein | | |
| K02380 | 5 | FdhE protein | | |
| K02381 | 6 | FdrA protein | | |
| K02382 | 3 | flagellar protein FlbA | | |

| | | | | |
|---|---|---|---|---|
| K02384 | 3 | flagellar protein FlbC | | |
| K02386 | 1 | flagella basal body P-ring formation protein FlgA | | |
| K02387 | 12 | flagellar basal-body rod protein FlgB | | |
| K02388 | 133 | flagellar basal-body rod protein FlgC | | |
| K02389 | 14 | flagellar basal-body rod modification protein FlgD | | |
| K02390 | 14 | flagellar hook protein FlgE | | |
| K02391 | 4 | flagellar basal-body rod protein FlgF | | |
| K02392 | 15 | flagellar basal-body rod protein FlgG | | |
| K02393 | 4 | flagellar L-ring protein precursor FlgH | | |
| K02394 | 6 | flagellar P-ring protein precursor FlgI | | |
| K02395 | 11 | flagellar protein FlgJ | | |
| K02396 | 12 | flagellar hook-associated protein 1 FlgK | | |
| K02397 | 8 | flagellar hook-associated protein 3 FlgL | | |
| K02400 | 16 | flagellar biosynthesis protein FlhA | | |
| K02401 | 8 | flagellar biosynthetic protein FlhB | | |
| K02402 | 1 | flagellar transcriptional activator FlhC | | |
| K02404 | 6 | flagellar biosynthesis protein FlhF | | |
| K02405 | 21 | RNA polymerase sigma factor for flagellar operon FliA | | |
| K02406 | 10 | flagellin | | |
| K02407 | 17 | flagellar hook-associated protein 2 | | |
| K02408 | 2 | flagellar hook-basal body complex protein FliE | | |
| K02409 | 14 | flagellar M-ring protein FliF | | |
| K02410 | 16 | flagellar motor switch protein FliG | | |
| K02411 | 3 | flagellar assembly protein FliH | | |
| K02412 | 12 | flagellum-specific ATP synthase [EC:3.6.3.14] | | |
| K02413 | 1 | flagellar FliJ protein | | |
| K02414 | 1 | flagellar hook-length control protein FliK | | |
| K02415 | 3 | flagellar FliL protein | | |
| K02416 | 15 | flagellar motor switch protein FliM | | |
| K02417 | 6 | flagellar motor switch protein FliN/FliY | | |
| K02418 | 2 | flagellar protein FliO/FliZ | | |
| K02419 | 7 | flagellar biosynthetic protein FliP | | |
| K02420 | 2 | flagellar biosynthetic protein FliQ | | |
| K02421 | 2 | flagellar biosynthetic protein FliR | | |
| K02422 | 3 | flagellar protein FliS | | |
| K02424 | 2 | cystine transport system substrate-binding protein | | |
| K02426 | 10 | cysteine desulfuration protein SufE | | |
| K02427 | 13 | ribosomal RNA large subunit methyltransferase E [EC:2.1.1.-] | | |
| K02428 | 30 | nucleoside-triphosphate pyrophosphatase [EC:3.6.1.19] | | |
| K02429 | 11 | MFS transporter, FHS family, L-fucose permease | | |
| K02431 | 1 | L-fucose mutarotase [EC:5.1.3.-] | | |
| K02433 | 141 | aspartyl-tRNA(Asn)/glutamyl-tRNA (Gln) amidotransferase subunit A | | |
| K02434 | 54 | aspartyl-tRNA(Asn)/glutamyl-tRNA (Gln) amidotransferase subunit B | | |
| K02435 | 18 | aspartyl-tRNA(Asn)/glutamyl-tRNA (Gln) amidotransferase subunit C | | |
| K02436 | 2 | DeoR family transcriptional regulator, galactitol utilization operon | | |
| K02437 | 29 | glycine cleavage system H protein | | |
| K02438 | 33 | glycogen operon protein GlgX [EC:3.2.1.-] | | |
| K02439 | 2 | thiosulfate sulfurtransferase [EC:2.8.1.1] | | |
| K02440 | 14 | glycerol uptake facilitator protein | | |
| K02441 | 1 | GlpG protein | | |
| K02444 | 5 | DeoR family transcriptional regulator, glycerol-3-phosphate regulon | | |
| K02445 | 1 | MFS transporter, OPA family, glycerol-3-phosphate transporter | | |
| K02446 | 28 | fructose-1,6-bisphosphatase II [EC:3.1.3.11] | | |
| K02448 | 3 | nitric-oxide reductase NorD protein [EC:1.7.99.7] | | |
| K02450 | 12 | general secretion pathway protein A | | |
| K02452 | 1 | general secretion pathway protein C | | |
| K02453 | 90 | general secretion pathway protein D | | |
| K02454 | 48 | general secretion pathway protein E | | |
| K02455 | 102 | general secretion pathway protein F | | |

| | | | | |
|---|---|---|---|---|
| K02456 | 16 | general secretion pathway protein G | | |
| K02457 | 2 | general secretion pathway protein H | | |
| K02458 | 2 | general secretion pathway protein I | | |
| K02459 | 2 | general secretion pathway protein J | | |
| K02460 | 10 | general secretion pathway protein K | | |
| K02461 | 7 | general secretion pathway protein L | | |
| K02464 | 6 | general secretion pathway protein O [EC:3.4.23.43 2.1.1.-] | | |
| K02469 | 80 | DNA gyrase subunit A [EC:5.99.1.3] | | |
| K02470 | 50 | DNA gyrase subunit B [EC:5.99.1.3] | | |
| K02471 | 16 | putative ATP-binding cassette transporter | | |
| K02472 | 10 | UDP-N-acetyl-D-mannosaminuronic acid dehydrogenase [EC:1.1.1.-] | | |
| K02473 | 35 | UDP-N-acetylglucosamine 4-epimerase [EC:5.1.3.7] | | |
| K02474 | 11 | UDP-N-acetyl-D-galactosamine dehydrogenase [EC:1.1.1.-] | | |
| K02475 | 2 | two-component system, CitB family, response regulator | | |
| K02477 | 67 | two-component system, LytT family, response regulator | | |
| K02478 | 4 | two-component system, LytT family, sensor kinase [EC:2.7.13.3] | | |
| K02479 | 79 | two-component system, NarL family, response regulator | | |
| K02480 | 32 | two-component system, NarL family, sensor kinase [EC:2.7.13.3] | | |
| K02481 | 134 | two-component system, NtrC family, response regulator | | |
| K02482 | 143 | two-component system, NtrC family, sensor kinase [EC:2.7.13.3] | | |
| K02483 | 128 | two-component system, OmpR family, response regulator | | |
| K02484 | 139 | two-component system, OmpR family, sensor kinase [EC:2.7.13.3] | | |
| K02485 | 11 | two-component system, unclassified family, response regulator | | |
| K02486 | 15 | two-component system, unclassified family, sensor kinase | | |
| K02487 | 1 | type IV pili sensor histidine kinase and response regulator | | |
| K02488 | 217 | two-component system, cell cycle response regulator | | |
| K02489 | 49 | two-component system, cell cycle sensor kinase and response | | |
| K02490 | 30 | two-component system, response regulator, stage 0 sporulation | | |
| K02491 | 8 | two-component system, sporulation sensor kinase A [EC:2.7.13.3] | | |
| K02492 | 42 | glutamyl-tRNA reductase [EC:1.2.1.70] | | |
| K02493 | 32 | methyltransferase [EC:2.1.1.-] | | |
| K02494 | 3 | outer membrane lipoprotein LolB | | |
| K02495 | 53 | oxygen-independent coproporphyrinogen III oxidase [EC:1.3.99.22] | | |
| K02496 | 3 | uroporphyrin-III C-methyltransferase [EC:2.1.1.107] | | |
| K02497 | 2 | HemX protein | | |
| K02498 | 3 | HemY protein | | |
| K02499 | 13 | tetrapyrrole methylase family protein / MazG family protein | | |
| K02500 | 27 | cyclase HisF [EC:4.1.3.-] | | |
| K02501 | 31 | glutamine amidotransferase [EC:2.4.2.-] | | |
| K02502 | 22 | ATP phosphoribosyltransferase regulatory subunit | | |
| K02503 | 30 | Hit-like protein involved in cell-cycle regulation | | |
| K02504 | 1 | protein transport protein HofB | | |
| K02507 | 4 | protein transport protein HofQ | | |
| K02508 | 2 | AraC family transcriptional regulator, 4-hydroxyphenylacetate | | |
| K02509 | 7 | 2-oxo-hept-3-ene-1,7-dioate hydratase [EC:4.2.1.-] | | |
| K02510 | 35 | 2,4-dihydroxyhept-2-ene-1,7-dioic acid aldolase [EC:4.1.2.-] | | |
| K02511 | 1 | MFS transporter, ACS family, 4-hydroxyphenylacetate permease | | |
| K02517 | 28 | lipid A biosynthesis lauroyl acyltransferase [EC:2.3.1.-] | | |
| K02518 | 13 | translation initiation factor IF-1 | | |
| K02519 | 44 | translation initiation factor IF-2 | | |
| K02520 | 31 | translation initiation factor IF-3 | | |
| K02523 | 22 | octaprenyl diphosphate synthase [EC:2.5.1.-] | | |
| K02525 | 1 | LacI family transcriptional regulator, kdg operon repressor | | |
| K02526 | 3 | 2-keto-3-deoxygluconate permease | | |
| K02527 | 16 | 3-deoxy-D-manno-octulosonic-acid transferase [EC:2.-.-.-] | | |
| K02528 | 35 | dimethyladenosine transferase [EC:2.1.1.-] | | |
| K02529 | 80 | LacI family transcriptional regulator | | |
| K02532 | 1 | MFS transporter, OHS family, lactose permease | | |
| K02533 | 19 | tRNA/rRNA methyltransferase [EC:2.1.1.-] | | |

| | | | | |
|---|---|---|---|---|
| K02535 | 25 | UDP-3-O-[3-hydroxymyristoyl] N-acetylglucosamine deacetylase | | |
| K02536 | 33 | UDP-3-O-[3-hydroxymyristoyl] glucosamine N-acyltransferase | | |
| K02545 | 2 | penicillin-binding protein 2 prime | | |
| K02547 | 1 | methicillin resistance protein | | |
| K02548 | 10 | 1,4-dihydroxy-2-naphthoate octaprenyltransferase [EC:2.5.1.-] | | |
| K02549 | 20 | O-succinylbenzoate synthase [EC:4.2.1.113] | | |
| K02550 | 1 | glycolate permease | | |
| K02551 | 32 | 2-succinyl-5-enolpyruvyl-6-hydroxy-3-cyclohexene-1-carboxylate | | |
| K02552 | 12 | menaquinone-specific isochorismate synthase [EC:5.4.4.2] | | |
| K02553 | 1 | regulator of ribonuclease activity A | | |
| K02554 | 5 | 2-keto-4-pentenoate hydratase [EC:4.2.1.80] | | |
| K02556 | 5 | chemotaxis protein MotA | | |
| K02557 | 23 | chemotaxis protein MotB | | |
| K02558 | 24 | UDP-N-acetylmuramate: L-alanyl-gamma-D-glutamyl-meso-diaminopimelate | | |
| K02562 | 2 | mannitol operon repressor | | |
| K02563 | 30 | UDP-N-acetylglucosamine--N-acetylmuramyl-(pentapeptide) | | |
| K02564 | 23 | glucosamine-6-phosphate deaminase [EC:3.5.99.6] | | |
| K02565 | 5 | N-acetylglucosamine repressor | | |
| K02566 | 16 | NagD protein | | |
| K02567 | 28 | periplasmic nitrate reductase NapA [EC:1.7.99.4] | | |
| K02568 | 8 | cytochrome c-type protein NapB | | |
| K02569 | 3 | cytochrome c-type protein NapC | | |
| K02571 | 1 | periplasmic nitrate reductase NapE | | |
| K02572 | 2 | ferredoxin-type protein NapF | | |
| K02573 | 7 | ferredoxin-type protein NapG | | |
| K02574 | 7 | ferredoxin-type protein NapH | | |
| K02575 | 27 | MFS transporter, NNP family, nitrate/nitrite transporter | | |
| K02584 | 38 | Nif-specific regulatory protein | | |
| K02585 | 2 | nitrogen fixation protein NifB | | |
| K02586 | 4 | nitrogenase molybdenum-iron protein alpha chain [EC:1.18.6.1] | | |
| K02587 | 1 | nitrogenase molybdenum-cofactor synthesis protein NifE | | |
| K02588 | 1 | nitrogenase iron protein NifH [EC:1.18.6.1] | | |
| K02591 | 3 | nitrogenase molybdenum-iron protein beta chain [EC:1.18.6.1] | | |
| K02592 | 1 | nitrogenase molybdenum-iron protein NifN | | |
| K02594 | 3 | homocitrate synthase NifV | | |
| K02597 | 5 | nitrogen fixation protein NifZ | | |
| K02598 | 1 | nitrite transporter NirC | | |
| K02600 | 28 | N utilization substance protein A | | |
| K02601 | 16 | transcriptional antiterminator NusG | | |
| K02609 | 16 | phenylacetic acid degradation protein | | |
| K02610 | 4 | phenylacetic acid degradation protein | | |
| K02611 | 10 | phenylacetic acid degradation protein | | |
| K02612 | 14 | phenylacetic acid degradation protein | | |
| K02613 | 11 | phenylacetic acid degradation NADH oxidoreductase | | |
| K02614 | 21 | phenylacetic acid degradation protein | | |
| K02615 | 3 | acetyl-CoA acetyltransferase [EC:2.3.1.-] | | |
| K02616 | 6 | phenylacetic acid degradation operon negative regulatory protein | | |
| K02617 | 6 | phenylacetic acid degradation protein | | |
| K02618 | 19 | phenylacetic acid degradation protein | | |
| K02619 | 44 | 4-amino-4-deoxychorismate lyase [EC:4.1.3.38] | | |
| K02621 | 13 | topoisomerase IV subunit A [EC:5.99.1.-] | | |
| K02622 | 18 | topoisomerase IV subunit B [EC:5.99.1.-] | | |
| K02623 | 10 | LysR family transcriptional regulator, pca operon transcriptional | | |
| K02624 | 13 | IclR family transcriptional regulator, pca regulon regulatory | | |
| K02626 | 5 | arginine decarboxylase [EC:4.1.1.19] | | |
| K02635 | 19 | cytochrome b6 | | |
| K02636 | 14 | cytochrome b6-f complex iron-sulfur subunit [EC:1.10.99.1] | | |
| K02637 | 1 | cytochrome b6-f complex subunit 4 | | |
| K02638 | 2 | plastocyanin | | |

| | | | | |
|---|---|---|---|---|
| K02641 | 2 | ferredoxin--NADP+ reductase [EC:1.18.1.2] | | |
| K02647 | 2 | carbohydrate diacid regulator | | |
| K02650 | 7 | type IV pilus assembly protein PilA | | |
| K02651 | 5 | pilus assembly protein Flp/PilA | | |
| K02652 | 69 | type IV pilus assembly protein PilB | | |
| K02653 | 77 | type IV pilus assembly protein PilC | | |
| K02654 | 24 | leader peptidase (prepilin peptidase) / N-methyltransferase | | |
| K02655 | 6 | type IV pilus assembly protein PilE | | |
| K02656 | 15 | type IV pilus assembly protein PilF | | |
| K02657 | 13 | twitching motility two-component system response regulator PilG | | |
| K02658 | 37 | twitching motility two-component system response regulator PilH | | |
| K02659 | 5 | twitching motility protein PilI | | |
| K02660 | 18 | twitching motility protein PilJ | | |
| K02661 | 2 | type IV pilus assembly protein PilK | | |
| K02662 | 35 | type IV pilus assembly protein PilM | | |
| K02663 | 6 | type IV pilus assembly protein PilN | | |
| K02664 | 9 | type IV pilus assembly protein PilO | | |
| K02665 | 4 | type IV pilus assembly protein PilP | | |
| K02666 | 66 | type IV pilus assembly protein PilQ | | |
| K02667 | 64 | two-component system, NtrC family, response regulator PilR | | |
| K02668 | 66 | two-component system, NtrC family, sensor histidine kinase PilS | | |
| K02669 | 68 | twitching motility protein PilT | | |
| K02670 | 13 | twitching motility protein PilU | | |
| K02671 | 2 | type IV pilus assembly protein PilV | | |
| K02672 | 4 | type IV pilus assembly protein PilW | | |
| K02673 | 7 | type IV pilus assembly protein PilX | | |
| K02674 | 38 | type IV pilus assembly protein PilY1 | | |
| K02676 | 2 | type IV pilus assembly protein PilZ | | |
| K02686 | 4 | primosomal replication protein N | | |
| K02687 | 27 | ribosomal protein L11 methyltransferase [EC:2.1.1.-] | | |
| K02688 | 2 | transcriptional regulator, propionate catabolism operon regulatory | | |
| K02690 | 1 | photosystem I core protein Ib | | |
| K02745 | 1 | PTS system, N-acetylgalactosamine-specific IIB component | | |
| K02755 | 1 | PTS system, beta-glucosides-specific IIA component [EC:2.7.1.69] | | |
| K02759 | 4 | PTS system, cellobiose-specific IIA component [EC:2.7.1.69] | | |
| K02761 | 9 | PTS system, cellobiose-specific IIC component | | |
| K02765 | 3 | PTS system, D-glucosamine-specific IIC component | | |
| K02768 | 6 | PTS system, fructose-specific IIA component [EC:2.7.1.69] | | |
| K02769 | 1 | PTS system, fructose-specific IIB component [EC:2.7.1.69] | | |
| K02770 | 12 | PTS system, fructose-specific IIC component | | |
| K02774 | 5 | PTS system, galactitol-specific IIB component [EC:2.7.1.69] | | |
| K02775 | 2 | PTS system, galactitol-specific IIC component | | |
| K02777 | 109 | PTS system, glucose-specific IIA component [EC:2.7.1.69] | | |
| K02779 | 2 | PTS system, glucose-specific IIC component | | |
| K02784 | 4 | phosphocarrier protein HPr | | |
| K02793 | 6 | PTS system, mannose-specific IIA component [EC:2.7.1.69] | | |
| K02794 | 3 | PTS system, mannose-specific IIB component [EC:2.7.1.69] | | |
| K02795 | 3 | PTS system, mannose-specific IIC component | | |
| K02796 | 6 | PTS system, mannose-specific IID component | | |
| K02799 | 2 | PTS system, mannitol-specific IIB component [EC:2.7.1.69] | | |
| K02800 | 3 | PTS system, mannitol-specific IIC component | | |
| K02804 | 1 | PTS system, N-acetylglucosamine-specific IIC component | | |
| K02805 | 4 | lipopolysaccharide biosynthesis protein | | |
| K02806 | 15 | PTS system, nitrogen regulatory IIA component [EC:2.7.1.69] | | |
| K02821 | 9 | PTS system, ascorbate-specific IIA component [EC:2.7.1.69] | | |
| K02823 | 18 | dihydroorotate dehydrogenase electron transfer subunit | | |
| K02824 | 6 | uracil permease | | |
| K02825 | 13 | pyrimidine operon attenuation protein / uracil | | |
| K02826 | 1 | quinol oxidase polypeptide II [EC:1.9.3.-] | | |

| | | | | |
|---|---|---|---|---|
| K02827 | 1 | quinol oxidase polypeptide I [EC:1.9.3.-] | | |
| K02828 | 1 | quinol oxidase polypeptide III [EC:1.9.3.-] | | |
| K02834 | 16 | ribosome-binding factor A | | |
| K02835 | 26 | peptide chain release factor RF-1 | | |
| K02836 | 23 | peptide chain release factor RF-2 | | |
| K02837 | 18 | peptide chain release factor RF-3 | | |
| K02838 | 30 | ribosome recycling factor | | |
| K02839 | 2 | peptide chain release factor RF-H | | |
| K02841 | 22 | heptosyltransferase I [EC:2.4.-.-] | | |
| K02843 | 38 | heptosyltransferase II [EC:2.4.-.-] | | |
| K02844 | 36 | UDP-glucose:(heptosyl)LPS alpha-1,3-glucosyltransferase [EC:2.4.1.-] | | |
| K02846 | 9 | N-methyl-L-tryptophan oxidase [EC:1.5.3.-] | | |
| K02849 | 21 | heptosyltransferase III [EC:2.4.-.-] | | |
| K02852 | 8 | UDP-N-acetyl-D-mannosaminuronic acid transferase [EC:2.4.1.-] | | |
| K02854 | 1 | AraC family transcriptional regulator, L-rhamnose operon | | |
| K02855 | 3 | AraC family transcriptional regulator, L-rhamnose operon regulatory | | |
| K02856 | 3 | L-rhamnose-H+ transport protein | | |
| K02858 | 40 | 3,4-dihydroxy 2-butanone 4-phosphate synthase [EC:4.1.99.12] | | |
| K02860 | 41 | 16S rRNA processing protein RimM | | |
| K02862 | 6 | putative membrane protein | | |
| K02863 | 21 | large subunit ribosomal protein L1 | | |
| K02864 | 19 | large subunit ribosomal protein L10 | | |
| K02867 | 17 | large subunit ribosomal protein L11 | | |
| K02871 | 16 | large subunit ribosomal protein L13 | | |
| K02874 | 22 | large subunit ribosomal protein L14 | | |
| K02876 | 12 | large subunit ribosomal protein L15 | | |
| K02878 | 19 | large subunit ribosomal protein L16 | | |
| K02879 | 21 | large subunit ribosomal protein L17 | | |
| K02881 | 11 | large subunit ribosomal protein L18 | | |
| K02884 | 16 | large subunit ribosomal protein L19 | | |
| K02886 | 47 | large subunit ribosomal protein L2 | | |
| K02887 | 6 | large subunit ribosomal protein L20 | | |
| K02888 | 23 | large subunit ribosomal protein L21 | | |
| K02890 | 18 | large subunit ribosomal protein L22 | | |
| K02892 | 34 | large subunit ribosomal protein L23 | | |
| K02895 | 14 | large subunit ribosomal protein L24 | | |
| K02897 | 28 | large subunit ribosomal protein L25 | | |
| K02899 | 20 | large subunit ribosomal protein L27 | | |
| K02902 | 9 | large subunit ribosomal protein L28 | | |
| K02904 | 6 | large subunit ribosomal protein L29 | | |
| K02906 | 36 | large subunit ribosomal protein L3 | | |
| K02907 | 9 | large subunit ribosomal protein L30 | | |
| K02909 | 22 | large subunit ribosomal protein L31 | | |
| K02911 | 73 | large subunit ribosomal protein L32 | | |
| K02913 | 9 | large subunit ribosomal protein L33 | | |
| K02914 | 15 | large subunit ribosomal protein L34 | | |
| K02916 | 7 | large subunit ribosomal protein L35 | | |
| K02919 | 2 | large subunit ribosomal protein L36 | | |
| K02926 | 30 | large subunit ribosomal protein L4 | | |
| K02931 | 15 | large subunit ribosomal protein L5 | | |
| K02933 | 28 | large subunit ribosomal protein L6 | | |
| K02935 | 6 | large subunit ribosomal protein L7/L12 | | |
| K02939 | 23 | large subunit ribosomal protein L9 | | |
| K02945 | 47 | small subunit ribosomal protein S1 | | |
| K02946 | 441 | small subunit ribosomal protein S10 | | |
| K02948 | 11 | small subunit ribosomal protein S11 | | |
| K02950 | 7 | small subunit ribosomal protein S12 | | |
| K02952 | 15 | small subunit ribosomal protein S13 | | |
| K02954 | 7 | small subunit ribosomal protein S14 | | |

| | | | | |
|---|---|---|---|---|
| K02956 | 8 | small subunit ribosomal protein S15 | | |
| K02959 | 23 | small subunit ribosomal protein S16 | | |
| K02961 | 10 | small subunit ribosomal protein S17 | | |
| K02963 | 15 | small subunit ribosomal protein S18 | | |
| K02965 | 4 | small subunit ribosomal protein S19 | | |
| K02967 | 68 | small subunit ribosomal protein S2 | | |
| K02968 | 28 | small subunit ribosomal protein S20 | | |
| K02970 | 4 | small subunit ribosomal protein S21 | | |
| K02982 | 20 | small subunit ribosomal protein S3 | | |
| K02986 | 30 | small subunit ribosomal protein S4 | | |
| K02988 | 13 | small subunit ribosomal protein S5 | | |
| K02990 | 23 | small subunit ribosomal protein S6 | | |
| K02992 | 15 | small subunit ribosomal protein S7 | | |
| K02994 | 23 | small subunit ribosomal protein S8 | | |
| K02996 | 16 | small subunit ribosomal protein S9 | | |
| K03040 | 34 | DNA-directed RNA polymerase subunit alpha [EC:2.7.7.6] | | |
| K03043 | 77 | DNA-directed RNA polymerase subunit beta [EC:2.7.7.6] | | |
| K03046 | 516 | DNA-directed RNA polymerase subunit beta' [EC:2.7.7.6] | | |
| K03060 | 10 | DNA-directed RNA polymerase subunit omega [EC:2.7.7.6] | | |
| K03070 | 74 | preprotein translocase subunit SecA | | |
| K03071 | 7 | preprotein translocase subunit SecB | | |
| K03072 | 476 | preprotein translocase subunit SecD | | |
| K03073 | 2 | preprotein translocase subunit SecE | | |
| K03074 | 41 | preprotein translocase subunit SecF | | |
| K03075 | 3 | preprotein translocase subunit SecG | | |
| K03076 | 36 | preprotein translocase subunit SecY | | |
| K03077 | 3 | L-ribulose-5-phosphate 4-epimerase [EC:5.1.3.4] | | |
| K03080 | 2 | L-ribulose-5-phosphate 4-epimerase [EC:5.1.3.4] | | |
| K03086 | 56 | RNA polymerase primary sigma factor | | |
| K03087 | 22 | RNA polymerase nonessential primary-like sigma factor | | |
| K03088 | 405 | RNA polymerase sigma-70 factor, ECF subfamily | | |
| K03089 | 23 | RNA polymerase sigma-32 factor | | |
| K03090 | 20 | RNA polymerase sigma-B factor | | |
| K03091 | 8 | RNA polymerase sporulation-specific sigma factor | | |
| K03092 | 47 | RNA polymerase sigma-54 factor | | |
| K03093 | 27 | RNA polymerase sigma factor | | |
| K03098 | 2 | outer membrane lipoprotein Blc | | |
| K03100 | 92 | signal peptidase I [EC:3.4.21.89] | | |
| K03101 | 20 | signal peptidase II [EC:3.4.23.36] | | |
| K03106 | 47 | signal recognition particle subunit SRP54 | | |
| K03110 | 23 | fused signal recognition particle receptor | | |
| K03111 | 34 | single-strand DNA-binding protein | | |
| K03113 | 2 | translation initiation factor SUI1 | | |
| K03116 | 10 | sec-independent protein translocase protein TatA | | |
| K03117 | 4 | sec-independent protein translocase protein TatB | | |
| K03118 | 57 | sec-independent protein translocase protein TatC | | |
| K03119 | 43 | taurine dioxygenase [EC:1.14.11.17] | | |
| K03146 | 1 | thiamine biosynthetic enzyme | | |
| K03147 | 11 | thiamine biosynthesis protein ThiC | | |
| K03148 | 16 | adenylyltransferase ThiF [EC:2.7.7.-] | | |
| K03149 | 27 | thiamine biosynthesis ThiG | | |
| K03150 | 1 | thiamine biosynthesis ThiH | | |
| K03151 | 4 | thiamine biosynthesis protein ThiI | | |
| K03152 | 3 | 4-methyl-5(b-hydroxyethyl)-thiazole monophosphate biosynthesis | | |
| K03153 | 10 | glycine oxidase [EC:1.4.3.19] | | |
| K03154 | 5 | thiamine biosynthesis ThiS | | |
| K03166 | 6 | DNA topoisomerase VI subunit A [EC:5.99.1.3] | | |
| K03167 | 4 | DNA topoisomerase VI subunit B [EC:5.99.1.3] | | |
| K03168 | 100 | DNA topoisomerase I [EC:5.99.1.2] | | |

| | | | | |
|---|---|---|---|---|
| K03169 | 32 | DNA topoisomerase III [EC:5.99.1.2] | | |
| K03177 | 42 | tRNA pseudouridine synthase B [EC:5.4.99.12] | | |
| K03179 | 26 | 4-hydroxybenzoate octaprenyltransferase [EC:2.5.1.-] | | |
| K03182 | 88 | 3-octaprenyl-4-hydroxybenzoate carboxy-lyase UbiD [EC:4.1.1.-] | | |
| K03183 | 77 | ubiquinone/menaquinone biosynthesis methyltransferase [EC:2.1.1.-] | | |
| K03184 | 1 | 2-octaprenyl-3-methyl-6-methoxy-1,4-benzoquinol hydroxylase | | |
| K03185 | 16 | 2-octaprenyl-6-methoxyphenol hydroxylase [EC:1.14.13.-] | | |
| K03186 | 20 | 3-octaprenyl-4-hydroxybenzoate carboxy-lyase UbiX [EC:4.1.1.-] | | |
| K03187 | 2 | urease accessory protein | | |
| K03188 | 1 | urease accessory protein | | |
| K03189 | 3 | urease accessory protein | | |
| K03190 | 2 | urease accessory protein | | |
| K03192 | 1 | urease accessory protein | | |
| K03196 | 1 | type IV secretion system protein VirB11 | | |
| K03197 | 2 | type IV secretion system protein VirB2 | | |
| K03198 | 1 | type IV secretion system protein VirB3 | | |
| K03199 | 8 | type IV secretion system protein VirB4 | | |
| K03200 | 1 | type IV secretion system protein VirB5 | | |
| K03201 | 2 | type IV secretion system protein VirB6 | | |
| K03205 | 7 | type IV secretion system protein VirD4 | | |
| K03208 | 1 | colanic acid biosynthesis glycosyl transferase WcaI | | |
| K03210 | 16 | preprotein translocase subunit YajC | | |
| K03215 | 31 | RNA methyltransferase, TrmA family [EC:2.1.1.-] | | |
| K03216 | 14 | RNA methyltransferase, TrmH family, group 2 [EC:2.1.1.-] | | |
| K03217 | 71 | preprotein translocase subunit YidC | | |
| K03218 | 28 | RNA methyltransferase, TrmH family [EC:2.1.1.-] | | |
| K03219 | 1 | type III secretion protein SctC | | |
| K03221 | 1 | type III secretion protein SctF | | |
| K03222 | 3 | type III secretion protein SctJ | | |
| K03223 | 1 | type III secretion protein SctL | | |
| K03224 | 1 | ATP synthase in type III secretion protein SctN [EC:3.6.3.14] | | |
| K03225 | 2 | type III secretion protein SctQ | | |
| K03227 | 2 | type III secretion protein SctS | | |
| K03228 | 2 | type III secretion protein SctT | | |
| K03229 | 1 | type III secretion protein SctU | | |
| K03230 | 2 | type III secretion protein SctV | | |
| K03265 | 1 | peptide chain release factor eRF subunit 1 | | |
| K03269 | 5 | UDP-2,3-diacylglucosamine hydrolase [EC:3.6.1.-] | | |
| K03270 | 14 | 3-deoxy-D-manno-octulosonate 8-phosphate phosphatase (KDO 8-P | | |
| K03271 | 12 | phosphoheptose isomerase [EC:5.-.-.-] | | |
| K03272 | 33 | D-beta-D-heptose 7-phosphate kinase / D-beta-D-heptose 1-phosphate | | |
| K03273 | 14 | D-glycero-D-manno-heptose 1,7-bisphosphate phosphatase [EC:3.1.3.-] | | |
| K03274 | 9 | ADP-L-glycero-D-manno-heptose 6-epimerase [EC:5.1.3.20] | | |
| K03280 | 1 | UDP-N-acetylglucosamine:(glucosyl)LPS | | |
| K03281 | 27 | chloride channel protein, CIC family | | |
| K03282 | 26 | large conductance mechanosensitive channel | | |
| K03284 | 57 | metal ion transporter, MIT family | | |
| K03285 | 10 | general bacterial porin, GBP family | | |
| K03286 | 45 | OmpA-OmpF porin, OOP family | | |
| K03287 | 84 | outer membrane factor, OMF family | | |
| K03288 | 4 | MFS transporter, MHS family, citrate/tricarballylate:H+ symporter | | |
| K03289 | 2 | MFS transporter, NHS family, nucleoside permease | | |
| K03290 | 3 | MFS transporter, SHS family, sialic acid transporter | | |
| K03292 | 11 | glycoside/pentoside/hexuronide:cation symporter, GPH family | | |
| K03293 | 16 | amino acid transporter, AAT family | | |
| K03294 | 140 | basic amino acid/polyamine antiporter, APA family | | |
| K03295 | 35 | cation efflux system protein, CDF family | | |
| K03296 | 415 | hydrophobic/amphiphilic exporter-1 (mainly G- bacteria), HAE1 family | | |
| K03297 | 2 | small multidrug resistance protein, SMR family | | |

| | | | | |
|---|---|---|---|---|
| K03298 | 13 | drug/metabolite transporter, DME family | | |
| K03299 | 35 | gluconate:H+ symporter, GntP family | | |
| K03302 | 3 | anaerobic C4-dicarboxylate transporter, Dcu family | | |
| K03303 | 17 | lactate transporter, LctP family | | |
| K03304 | 4 | tellurite resistance/dicarboxylate transporter, TDT family | | |
| K03305 | 27 | proton-dependent oligopeptide transporter, POT family | | |
| K03306 | 31 | inorganic phosphate transporter, PiT family | | |
| K03307 | 78 | solute:Na+ symporter, SSS family | | |
| K03308 | 31 | neurotransmitter:Na+ symporter, NSS family | | |
| K03309 | 14 | dicarboxylate/amino acid:cation (Na+ or H+) symporter, DAACS family | | |
| K03312 | 1 | glutamate:Na+ symporter, ESS family | | |
| K03313 | 15 | Na+:H+ antiporter, NhaA family | | |
| K03315 | 2 | Na+:H+ antiporter, NhaC family | | |
| K03316 | 31 | monovalent cation:H+ antiporter, CPA1 family | | |
| K03317 | 19 | concentrative nucleoside transporter, CNT family | | |
| K03319 | 2 | divalent anion:Na+ symporter, DASS family | | |
| K03320 | 62 | ammonium transporter, Amt family | | |
| K03321 | 52 | sulfate permease, SulP family | | |
| K03322 | 54 | manganese transport protein | | |
| K03324 | 10 | phosphate:Na+ symporter, PNaS family | | |
| K03325 | 22 | arsenite transporter, ACR3 family | | |
| K03326 | 1 | C4-dicarboxylate transporter, DcuC family | | |
| K03327 | 27 | multidrug resistance protein, MATE family | | |
| K03328 | 25 | polysaccharide transporter, PST family | | |
| K03331 | 1 | L-xylulose reductase [EC:1.1.1.10] | | |
| K03332 | 2 | fructan beta-fructosidase [EC:3.2.1.80] | | |
| K03333 | 41 | cholesterol oxidase [EC:1.1.3.6] | | |
| K03335 | 20 | inosose dehydratase [EC:4.2.1.44] | | |
| K03336 | 12 | 3D-(3,5/4)-trihydroxycyclohexane-1,2-dione hydrolase [EC:3.7.1.-] | | |
| K03337 | 2 | 5-deoxy-glucuronate isomerase [EC:5.3.1.-] | | |
| K03338 | 5 | 5-dehydro-2-deoxygluconokinase [EC:2.7.1.92] | | |
| K03339 | 1 | 6-phospho-5-dehydro-2-deoxy-D-gluconate aldolase [EC:4.1.2.29] | | |
| K03342 | 7 | para-aminobenzoate synthetase / 4-amino-4-deoxychorismate lyase | | |
| K03343 | 8 | putrescine oxidase [EC:1.4.3.10] | | |
| K03366 | 4 | (R,R)-butanediol dehydrogenase / diacetyl reductase [EC:1.1.1.4 | | |
| K03367 | 32 | D-alanine-poly(phosphoribitol) ligase [EC:6.1.1.13] | | |
| K03379 | 37 | cyclohexanone monooxygenase [EC:1.14.13.22] | | |
| K03380 | 35 | phenol 2-monooxygenase [EC:1.14.13.7] | | |
| K03381 | 13 | catechol 1,2-dioxygenase [EC:1.13.11.1] | | |
| K03382 | 5 | hydroxyatrazine ethylaminohydrolase [EC:3.5.99.3] | | |
| K03384 | 10 | Unclassified; E1.14.12.- | | |
| K03385 | 9 | formate-dependent nitrite reductase, periplasmic cytochrome c552 | | |
| K03386 | 31 | peroxiredoxin (alkyl hydroperoxide reductase subunit C) | | |
| K03387 | 2 | alkyl hydroperoxide reductase subunit F [EC:1.6.4.-] | | |
| K03388 | 65 | heterodisulfide reductase subunit A [EC:1.8.98.1] | | |
| K03389 | 22 | heterodisulfide reductase subunit B [EC:1.8.98.1] | | |
| K03390 | 14 | heterodisulfide reductase subunit C [EC:1.8.98.1] | | |
| K03391 | 42 | pentachlorophenol monooxygenase [EC:1.14.13.50] | | |
| K03392 | 61 | aminocarboxymuconate-semialdehyde decarboxylase [EC:4.1.1.45] | | |
| K03394 | 1 | precorrin-2 C20-methyltransferase / cobalt-factor-2 | | |
| K03396 | 1 | S-(hydroxymethyl)glutathione synthase [EC:4.4.1.22] | | |
| K03399 | 2 | precorrin-6Y C5,15-methyltransferase [EC:2.1.1.132] | | |
| K03402 | 7 | transcriptional regulator of arginine metabolism | | |
| K03403 | 4 | magnesium chelatase subunit H [EC:6.6.1.1] | | |
| K03404 | 10 | magnesium chelatase subunit D [EC:6.6.1.1] | | |
| K03405 | 29 | magnesium chelatase subunit I [EC:6.6.1.1] | | |
| K03406 | 186 | methyl-accepting chemotaxis protein | | |
| K03407 | 82 | two-component system, chemotaxis family, sensor kinase CheA | | |
| K03408 | 21 | purine-binding chemotaxis protein CheW | | |

| | | | | |
|---|---|---|---|---|
| K03410 | 4 | chemotaxis protein CheC | | |
| K03411 | 16 | chemotaxis protein CheD [EC:3.5.1.44] | | |
| K03412 | 64 | two-component system, chemotaxis family, response regulator CheB | | |
| K03413 | 117 | two-component system, chemotaxis family, response regulator CheY | | |
| K03414 | 3 | chemotaxis protein CheZ | | |
| K03416 | 11 | methylmalonyl-CoA carboxyltransferase [EC:2.1.3.1] | | |
| K03417 | 36 | methylisocitrate lyase [EC:4.1.3.30] | | |
| K03423 | 57 | Unclassified; E1.8.-.- | | |
| K03424 | 47 | TatD DNase family protein [EC:3.1.21.-] | | |
| K03426 | 21 | NAD+ diphosphatase [EC:3.6.1.22] | | |
| K03427 | 48 | type I restriction enzyme M protein [EC:2.1.1.72] | | |
| K03429 | 22 | 1,2-diacylglycerol 3-glucosyltransferase [EC:2.4.1.157] | | |
| K03430 | 5 | 2-aminoethylphosphonate-pyruvate transaminase [EC:2.6.1.37] | | |
| K03431 | 33 | phosphoglucosamine mutase [EC:5.4.2.10] | | |
| K03432 | 2 | proteasome alpha subunit [EC:3.4.25.1] | | |
| K03433 | 9 | proteasome beta subunit [EC:3.4.25.1] | | |
| K03434 | 14 | N-acetylglucosaminylphosphatidylinositol deacetylase [EC:3.5.1.89] | | |
| K03435 | 5 | LacI family transcriptional regulator, fructose operon | | |
| K03436 | 5 | DeoR family transcriptional regulator, fructose operon | | |
| K03437 | 25 | RNA methyltransferase, TrmH family | | |
| K03438 | 31 | S-adenosyl-methyltransferase [EC:2.1.1.-] | | |
| K03439 | 7 | tRNA (guanine-N7-)-methyltransferase [EC:2.1.1.33] | | |
| K03442 | 38 | small conductance mechanosensitive channel | | |
| K03445 | 2 | MFS transporter, DHA1 family, purine ribonucleoside efflux pump | | |
| K03446 | 127 | MFS transporter, DHA2 family, multidrug resistance protein B | | |
| K03449 | 8 | MFS transporter, CP family, cyanate transporter | | |
| K03453 | 3 | bile acid:Na+ symporter, BASS family | | |
| K03455 | 84 | monovalent cation:H+ antiporter-2, CPA2 family | | |
| K03457 | 23 | nucleobase:cation symporter-1, NCS1 family | | |
| K03458 | 12 | nucleobase:cation symporter-2, NCS2 family | | |
| K03463 | 5 | Unclassified; E5.5.1.- | | |
| K03464 | 2 | muconolactone D-isomerase [EC:5.3.3.4] | | |
| K03465 | 12 | thymidylate synthase (FAD) [EC:2.1.1.148] | | |
| K03466 | 493 | DNA segregation ATPase FtsK/SpoIIIE, S-DNA-T family | | |
| K03469 | 41 | ribonuclease HI [EC:3.1.26.4] | | |
| K03470 | 44 | ribonuclease HII [EC:3.1.26.4] | | |
| K03471 | 3 | ribonuclease HIII [EC:3.1.26.4] | | |
| K03473 | 1 | erythronate-4-phosphate dehydrogenase [EC:1.1.1.290] | | |
| K03474 | 23 | pyridoxine 5-phosphate synthase [EC:2.6.99.2] | | |
| K03475 | 1 | PTS system, ascorbate-specific IIC component | | |
| K03476 | 18 | L-ascorbate 6-phosphate lactonase [EC:3.1.1.-] | | |
| K03478 | 5 | hypothetical protein | | |
| K03484 | 4 | LacI family transcriptional regulator, sucrose operon repressor | | |
| K03495 | 39 | glucose inhibited division protein A | | |
| K03496 | 82 | chromosome partitioning protein | | |
| K03497 | 69 | chromosome partitioning protein, ParB family | | |
| K03498 | 19 | trk system potassium uptake protein TrkH | | |
| K03499 | 59 | trk system potassium uptake protein TrkA | | |
| K03500 | 54 | ribosomal RNA small subunit methyltransferase B [EC:2.1.1.-] | | |
| K03501 | 26 | glucose inhibited division protein B [EC:2.1.-.-] | | |
| K03502 | 3 | DNA polymerase V | | |
| K03517 | 23 | quinolinate synthase [EC:2.5.1.72] | | |
| K03518 | 27 | carbon-monoxide dehydrogenase small subunit [EC:1.2.99.2] | | |
| K03519 | 52 | carbon-monoxide dehydrogenase medium subunit [EC:1.2.99.2] | | |
| K03520 | 196 | carbon-monoxide dehydrogenase large subunit [EC:1.2.99.2] | | |
| K03521 | 32 | electron transfer flavoprotein beta subunit | | |
| K03522 | 30 | electron transfer flavoprotein alpha subunit | | |
| K03523 | 5 | putative biotin biosynthesis protein BioY | | |
| K03524 | 31 | BirA family transcriptional regulator, biotin operon repressor / | | |

| | | | | |
|---|---|---|---|---|
| K03525 | 31 | type III pantothenate kinase [EC:2.7.1.33] | | |
| K03526 | 24 | (E)-4-hydroxy-3-methylbut-2-enyl-diphosphate synthase [EC:1.17.7.1] | | |
| K03527 | 34 | 4-hydroxy-3-methylbut-2-enyl diphosphate reductase [EC:1.17.1.2] | | |
| K03529 | 61 | chromosome segregation protein | | |
| K03530 | 59 | DNA-binding protein HU-beta | | |
| K03531 | 44 | cell division protein FtsZ | | |
| K03532 | 3 | trimethylamine-N-oxide reductase (cytochrome c) 1, cytochrome c-type | | |
| K03534 | 1 | L-rhamnose mutarotase [EC:5.1.3.-] | | |
| K03535 | 12 | MFS transporter, ACS family, glucarate transporter | | |
| K03536 | 21 | ribonuclease P protein component [EC:3.1.26.5] | | |
| K03540 | 15 | ribonuclease P subunit RPR2 [EC:3.1.26.5] | | |
| K03543 | 22 | multidrug resistance protein A | | |
| K03544 | 21 | ATP-dependent Clp protease ATP-binding subunit ClpX | | |
| K03545 | 53 | trigger factor | | |
| K03546 | 16 | exonuclease SbcC | | |
| K03547 | 5 | exonuclease SbcD | | |
| K03548 | 14 | putative permease | | |
| K03549 | 37 | KUP system potassium uptake protein | | |
| K03550 | 21 | holliday junction DNA helicase RuvA | | |
| K03551 | 35 | holliday junction DNA helicase RuvB | | |
| K03553 | 41 | recombination protein RecA | | |
| K03555 | 79 | DNA mismatch repair protein MutS | | |
| K03556 | 63 | LuxR family transcriptional regulator, maltose regulon positive | | |
| K03557 | 4 | Fis family transcriptional regulator, factor for inversion | | |
| K03558 | 4 | membrane protein required for colicin V production | | |
| K03559 | 16 | biopolymer transport protein ExbD | | |
| K03560 | 7 | biopolymer transport protein TolR | | |
| K03561 | 29 | biopolymer transport protein ExbB | | |
| K03562 | 14 | biopolymer transport protein TolQ | | |
| K03563 | 5 | carbon storage regulator | | |
| K03564 | 47 | peroxiredoxin Q/BCP [EC:1.11.1.15] | | |
| K03565 | 29 | regulatory protein | | |
| K03566 | 22 | LysR family transcriptional regulator, glycine cleavage system | | |
| K03567 | 1 | glycine cleavage system transcriptional repressor | | |
| K03568 | 39 | TldD protein | | |
| K03569 | 45 | rod shape-determining protein MreB and related proteins | | |
| K03570 | 25 | rod shape-determining protein MreC | | |
| K03571 | 3 | rod shape-determining protein MreD | | |
| K03572 | 44 | DNA mismatch repair protein MutL | | |
| K03574 | 44 | 7,8-dihydro-8-oxoguanine triphosphatase [EC:3.6.1.-] | | |
| K03575 | 41 | A/G-specific adenine glycosylase [EC:3.2.2.-] | | |
| K03576 | 19 | LysR family transcriptional regulator, regulator for metE and metH | | |
| K03577 | 16 | TetR/AcrR family transcriptional regulator, acrAB operon repressor | | |
| K03578 | 38 | ATP-dependent helicase HrpA [EC:3.6.1.-] | | |
| K03579 | 15 | ATP-dependent helicase HrpB [EC:3.6.1.-] | | |
| K03580 | 7 | ATP-dependent helicase HepA [EC:3.6.1.-] | | |
| K03581 | 29 | exodeoxyribonuclease V alpha subunit [EC:3.1.11.5] | | |
| K03582 | 8 | exodeoxyribonuclease V beta subunit [EC:3.1.11.5] | | |
| K03583 | 15 | exodeoxyribonuclease V gamma subunit [EC:3.1.11.5] | | |
| K03584 | 34 | DNA repair protein RecO (recombination protein O) | | |
| K03585 | 50 | membrane fusion protein | | |
| K03586 | 1 | cell division protein FtsL | | |
| K03587 | 74 | cell division protein FtsI (penicillin-binding protein 3) | | |
| K03588 | 46 | cell division protein FtsW | | |
| K03589 | 17 | cell division protein FtsQ | | |
| K03590 | 36 | cell division protein FtsA | | |
| K03592 | 45 | PmbA protein | | |
| K03593 | 48 | ATP-binding protein involved in chromosome partitioning | | |
| K03594 | 20 | bacterioferritin | | |

| | | | | |
|---|---|---|---|---|
| K03595 | 464 | GTP-binding protein Era | | |
| K03596 | 50 | GTP-binding protein LepA | | |
| K03597 | 1 | sigma-E factor negative regulatory protein RseA | | |
| K03598 | 9 | sigma-E factor negative regulatory protein RseB | | |
| K03599 | 4 | stringent starvation protein A | | |
| K03600 | 4 | stringent starvation protein B | | |
| K03601 | 45 | exodeoxyribonuclease VII large subunit [EC:3.1.11.6] | | |
| K03602 | 5 | exodeoxyribonuclease VII small subunit [EC:3.1.11.6] | | |
| K03604 | 5 | LacI family transcriptional regulator, purine nucleotide synthesis | | |
| K03605 | 10 | hydrogenase 1 maturation protease [EC:3.4.24.-] | | |
| K03606 | 8 | putative colanic acid biosysnthesis UDP-glucose lipid carrier | | |
| K03608 | 2 | cell division topological specificity factor | | |
| K03609 | 12 | septum site-determining protein MinD | | |
| K03610 | 2 | septum site-determining protein MinC | | |
| K03611 | 2 | disulfide bond formation protein DsbB | | |
| K03612 | 2 | electron transport complex protein RnfG | | |
| K03613 | 26 | electron transport complex protein RnfE | | |
| K03614 | 3 | electron transport complex protein RnfD | | |
| K03615 | 13 | electron transport complex protein RnfC | | |
| K03616 | 8 | electron transport complex protein RnfB | | |
| K03617 | 4 | electron transport complex protein RnfA | | |
| K03618 | 1 | hydrogenase-1 operon protein HyaF | | |
| K03620 | 12 | Ni/Fe-hydrogenase 1 B-type cytochrome subunit | | |
| K03621 | 30 | glycerol-3-phosphate acyltransferase PlsX [EC:2.3.1.15] | | |
| K03624 | 24 | transcription elongation factor GreA | | |
| K03625 | 21 | N utilization substance protein B | | |
| K03628 | 36 | transcription termination factor Rho | | |
| K03629 | 18 | DNA replication and repair protein RecF | | |
| K03630 | 39 | DNA repair protein RadC | | |
| K03631 | 48 | DNA repair protein RecN (Recombination protein N) | | |
| K03634 | 10 | outer membrane lipoprotein carrier protein | | |
| K03635 | 22 | molybdenum cofactor biosynthesis protein E | | |
| K03636 | 10 | molybdenum cofactor biosynthesis protein D | | |
| K03637 | 16 | molybdenum cofactor biosynthesis protein C | | |
| K03638 | 9 | molybdenum cofactor biosynthesis protein B | | |
| K03639 | 47 | molybdenum cofactor biosynthesis protein A | | |
| K03640 | 41 | peptidoglycan-associated lipoprotein | | |
| K03641 | 109 | TolB protein | | |
| K03642 | 13 | rare lipoprotein A | | |
| K03643 | 6 | LPS-assembly lipoprotein | | |
| K03644 | 32 | lipoic acid synthetase [EC:2.8.1.8] | | |
| K03648 | 29 | uracil-DNA glycosylase [EC:3.2.2.-] | | |
| K03649 | 18 | TDG/mug DNA glycosylase family protein [EC:3.2.2.-] | | |
| K03650 | 36 | tRNA modification GTPase | | |
| K03651 | 6 | Icc protein | | |
| K03652 | 17 | DNA-3-methyladenine glycosylase [EC:3.2.2.21] | | |
| K03654 | 47 | ATP-dependent DNA helicase RecQ [EC:3.6.1.-] | | |
| K03655 | 58 | ATP-dependent DNA helicase RecG [EC:3.6.1.-] | | |
| K03656 | 16 | ATP-dependent DNA helicase Rep [EC:3.6.1.-] | | |
| K03657 | 133 | DNA helicase II / ATP-dependent DNA helicase PcrA [EC:3.6.1.-] | | |
| K03664 | 27 | SsrA-binding protein | | |
| K03665 | 26 | GTP-binding protein HflX | | |
| K03666 | 10 | host factor-I protein | | |
| K03667 | 21 | ATP-dependent HslUV protease ATP-binding subunit HslU | | |
| K03668 | 3 | heat shock protein HslJ | | |
| K03669 | 4 | membrane glycosyltransferase [EC:2.4.1.-] | | |
| K03670 | 4 | periplasmic glucans biosynthesis protein | | |
| K03671 | 33 | thioredoxin 1 | | |
| K03672 | 8 | thioredoxin 2 [EC:1.8.1.8] | | |

| | | | | |
|---|---|---|---|---|
| K03673 | 13 | thiol:disulfide interchange protein DsbA | | |
| K03676 | 7 | glutaredoxin 3 | | |
| K03677 | 11 | CysQ protein | | |
| K03684 | 17 | ribonuclease D [EC:3.1.13.5] | | |
| K03685 | 34 | ribonuclease III [EC:3.1.26.3] | | |
| K03686 | 68 | molecular chaperone DnaJ | | |
| K03687 | 21 | molecular chaperone GrpE | | |
| K03688 | 57 | ubiquinone biosynthesis protein | | |
| K03690 | 5 | hypothetical protein | | |
| K03693 | 1 | penicillin-binding protein | | |
| K03694 | 10 | ATP-dependent Clp protease ATP-binding subunit ClpA | | |
| K03695 | 75 | ATP-dependent Clp protease ATP-binding subunit ClpB | | |
| K03696 | 39 | ATP-dependent Clp protease ATP-binding subunit ClpC | | |
| K03697 | 1 | ATP-dependent Clp protease ATP-binding subunit ClpE | | |
| K03698 | 6 | CMP-binding protein | | |
| K03699 | 57 | putative hemolysin | | |
| K03701 | 117 | excinuclease ABC subunit A | | |
| K03702 | 42 | excinuclease ABC subunit B | | |
| K03703 | 61 | excinuclease ABC subunit C | | |
| K03704 | 67 | cold shock protein (beta-ribbon, CspA family) | | |
| K03705 | 36 | heat-inducible transcriptional repressor | | |
| K03707 | 6 | transcriptional activator TenA [EC:3.5.99.2] | | |
| K03709 | 23 | DtxR family transcriptional regulator, Mn-dependent transcriptional | | |
| K03710 | 43 | GntR family transcriptional regulator | | |
| K03711 | 30 | Fur family transcriptional regulator, ferric uptake regulator | | |
| K03712 | 51 | MarR family transcriptional regulator | | |
| K03713 | 4 | MerR family transcriptional regulator, glutamine synthetase | | |
| K03715 | 6 | 1,2-diacylglycerol 3-beta-galactosyltransferase [EC:2.4.1.46] | | |
| K03717 | 10 | LysR family transcriptional regulator, transcriptional activator of | | |
| K03718 | 11 | Lrp/AsnC family transcriptional regulator, regulator for asnA, asnC | | |
| K03719 | 24 | Lrp/AsnC family transcriptional regulator, leucine-responsive | | |
| K03721 | 11 | transcriptional regulator of aroF, aroG, tyrA and aromatic amino | | |
| K03722 | 55 | ATP-dependent DNA helicase DinG [EC:3.6.1.-] | | |
| K03723 | 74 | transcription-repair coupling factor (superfamily II helicase) | | |
| K03724 | 91 | ATP-dependent helicase Lhr and Lhr-like helicase [EC:3.6.1.-] | | |
| K03726 | 1 | helicase [EC:3.6.1.-] | | |
| K03727 | 17 | ATP-dependent RNA helicase HelY [EC:3.6.1.-] | | |
| K03731 | 2 | trehalose 6-phosphate phosphorylase [EC:2.4.1.216] | | |
| K03732 | 1 | ATP-dependent RNA helicase RhlB [EC:3.6.4.13] | | |
| K03733 | 65 | integrase/recombinase XerC | | |
| K03734 | 31 | thiamine biosynthesis lipoprotein | | |
| K03735 | 4 | ethanolamine ammonia-lyase large subunit [EC:4.3.1.7] | | |
| K03736 | 1 | ethanolamine ammonia-lyase small subunit [EC:4.3.1.7] | | |
| K03737 | 30 | putative pyruvate-flavodoxin oxidoreductase [EC:1.2.7.-] | | |
| K03738 | 68 | aldehyde:ferredoxin oxidoreductase [EC:1.2.7.5] | | |
| K03740 | 1 | D-alanine transfer protein | | |
| K03741 | 27 | arsenate reductase [EC:1.20.4.1] | | |
| K03742 | 58 | competence/damage-inducible protein CinA | | |
| K03743 | 19 | Unclassified; K03743 | | |
| K03744 | 31 | LemA protein | | |
| K03746 | 2 | DNA-binding protein H-NS | | |
| K03747 | 1 | Smg protein | | |
| K03748 | 3 | SanA protein | | |
| K03749 | 2 | DedD protein | | |
| K03750 | 62 | molybdopterin biosynthesis protein MoeA | | |
| K03751 | 39 | molybdopterin biosynthesis protein MoeB | | |
| K03752 | 10 | molybdopterin-guanine dinucleotide biosynthesis protein A | | |
| K03753 | 6 | molybdopterin-guanine dinucleotide biosynthesis protein B | | |
| K03756 | 3 | putrescine:ornithine antiporter | | |

| | | | | |
|---|---|---|---|---|
| K03758 | 2 | arginine:ornithine antiporter | | |
| K03759 | 5 | arginine:agmatine antiporter | | |
| K03760 | 1 | phosphoethanolamine transferase | | |
| K03761 | 2 | MFS transporter, MHS family, alpha-ketoglutarate permease | | |
| K03762 | 32 | MFS transporter, MHS family, proline/betaine transporter | | |
| K03763 | 10 | DNA polymerase III subunit alpha, Gram-positive type [EC:2.7.7.7] | | |
| K03765 | 5 | transcriptional activator of cad operon | | |
| K03767 | 15 | peptidyl-prolyl cis-trans isomerase A (cyclophilin A) [EC:5.2.1.8] | | |
| K03768 | 25 | peptidyl-prolyl cis-trans isomerase B (cyclophilin B) [EC:5.2.1.8] | | |
| K03769 | 56 | peptidyl-prolyl cis-trans isomerase C [EC:5.2.1.8] | | |
| K03770 | 44 | peptidyl-prolyl cis-trans isomerase D [EC:5.2.1.8] | | |
| K03771 | 59 | peptidyl-prolyl cis-trans isomerase SurA [EC:5.2.1.8] | | |
| K03772 | 10 | FKBP-type peptidyl-prolyl cis-trans isomerase FkpA [EC:5.2.1.8] | | |
| K03773 | 4 | FKBP-type peptidyl-prolyl cis-trans isomerase FklB [EC:5.2.1.8] | | |
| K03774 | 1 | FKBP-type peptidyl-prolyl cis-trans isomerase SlpA [EC:5.2.1.8] | | |
| K03775 | 9 | FKBP-type peptidyl-prolyl cis-trans isomerase SlyD [EC:5.2.1.8] | | |
| K03776 | 16 | aerotaxis receptor | | |
| K03777 | 7 | D-lactate dehydrogenase [EC:1.1.1.28] | | |
| K03778 | 7 | D-lactate dehydrogenase [EC:1.1.1.28] | | |
| K03779 | 2 | L(+)-tartrate dehydratase alpha subunit [EC:4.2.1.32] | | |
| K03780 | 3 | L(+)-tartrate dehydratase beta subunit [EC:4.2.1.32] | | |
| K03781 | 12 | catalase [EC:1.11.1.6] | | |
| K03782 | 24 | catalase/peroxidase [EC:1.11.1.6 1.11.1.7] | | |
| K03783 | 27 | purine-nucleoside phosphorylase [EC:2.4.2.1] | | |
| K03784 | 5 | purine-nucleoside phosphorylase [EC:2.4.2.1] | | |
| K03785 | 2 | 3-dehydroquinate dehydratase I [EC:4.2.1.10] | | |
| K03786 | 17 | 3-dehydroquinate dehydratase II [EC:4.2.1.10] | | |
| K03787 | 26 | 5'-nucleotidase [EC:3.1.3.5] | | |
| K03789 | 28 | ribosomal-protein-alanine N-acetyltransferase [EC:2.3.1.128] | | |
| K03790 | 10 | ribosomal-protein-alanine N-acetyltransferase [EC:2.3.1.128] | | |
| K03791 | 7 | putative chitinase | | |
| K03793 | 3 | pteridine reductase [EC:1.5.1.33] | | |
| K03795 | 10 | sirohydrochlorin cobaltochelatase [EC:4.99.1.3] | | |
| K03797 | 79 | carboxyl-terminal processing protease [EC:3.4.21.102] | | |
| K03798 | 73 | cell division protease FtsH [EC:3.4.24.-] | | |
| K03799 | 40 | heat shock protein HtpX [EC:3.4.24.-] | | |
| K03800 | 12 | lipoate-protein ligase A [EC:2.7.7.63] | | |
| K03801 | 27 | lipoyl(octanoyl) transferase [EC:2.3.1.181] | | |
| K03802 | 15 | cyanophycin synthetase [EC:6.-.-.-] | | |
| K03806 | 4 | AmpD protein | | |
| K03808 | 5 | paraquat-inducible protein A | | |
| K03809 | 6 | Trp repressor binding protein | | |
| K03810 | 12 | virulence factor | | |
| K03811 | 3 | nicotinamide mononucleotide transporter | | |
| K03814 | 19 | monofunctional biosynthetic peptidoglycan transglycosylase | | |
| K03815 | 1 | xanthosine phosphorylase [EC:2.4.2.-] | | |
| K03818 | 8 | putative colanic acid biosynthesis acetyltransferase WcaF | | |
| K03819 | 1 | putative colanic acid biosynthesis acetyltransferase WcaB | | |
| K03820 | 42 | apolipoprotein N-acyltransferase [EC:2.3.1.-] | | |
| K03821 | 52 | polyhydroxyalkanoate synthase [EC:2.3.1.-] | | |
| K03822 | 3 | putative long chain acyl-CoA synthase [EC:6.2.1.-] | | |
| K03823 | 23 | phosphinothricin acetyltransferase [EC:2.3.1.183] | | |
| K03824 | 4 | putative acetyltransferase [EC:2.3.1.-] | | |
| K03825 | 4 | putative acetyltransferase [EC:2.3.1.-] | | |
| K03826 | 2 | putative acetyltransferase [EC:2.3.1.-] | | |
| K03827 | 2 | putative acetyltransferase [EC:2.3.1.-] | | |
| K03828 | 9 | putative acetyltransferase [EC:2.3.1.-] | | |
| K03829 | 8 | putative acetyltransferase [EC:2.3.1.-] | | |
| K03831 | 8 | molybdopterin biosynthesis protein Mog | | |

| | | | | |
|---|---|---|---|---|
| K03832 | 28 | periplasmic protein TonB | | |
| K03833 | 28 | selenocysteine-specific elongation factor | | |
| K03837 | 1 | serine transporter | | |
| K03839 | 5 | flavodoxin I | | |
| K03841 | 11 | fructose-1,6-bisphosphatase I [EC:3.1.3.11] | | |
| K03851 | 2 | taurine-pyruvate aminotransferase [EC:2.6.1.77] | | |
| K03852 | 6 | sulfoacetaldehyde acetyltransferase [EC:2.3.3.15] | | |
| K03856 | 30 | 3-deoxy-7-phosphoheptulonate synthase [EC:2.5.1.54] | | |
| K03862 | 13 | vanillate monooxygenase [EC:1.14.13.82] | | |
| K03863 | 2 | vanillate monooxygenase [EC:1.14.13.82] | | |
| K03885 | 77 | NADH dehydrogenase [EC:1.6.99.3] | | |
| K03886 | 3 | menaquinol-cytochrome c reductase iron-sulfur subunit [EC:1.10.2.-] | | |
| K03887 | 9 | menaquinol-cytochrome c reductase cytochrome b subunit [EC:1.10.2.-] | | |
| K03888 | 2 | menaquinol-cytochrome c reductase cytochrome b/c subunit | | |
| K03889 | 6 | ubiquinol-cytochrome c reductase cytochrome c subunit | | |
| K03890 | 3 | ubiquinol-cytochrome c reductase iron-sulfur subunit | | |
| K03891 | 15 | ubiquinol-cytochrome c reductase cytochrome b subunit | | |
| K03892 | 63 | ArsR family transcriptional regulator | | |
| K03893 | 12 | arsenical pump membrane protein | | |
| K03897 | 1 | lysine N6-hydroxylase [EC:1.14.13.59] | | |
| K03918 | 4 | L-lysine 6-transaminase [EC:2.6.1.36] | | |
| K03924 | 61 | MoxR-like ATPase [EC:3.6.3.-] | | |
| K03925 | 28 | MraZ protein | | |
| K03926 | 9 | periplasmic divalent cation tolerance protein | | |
| K03927 | 17 | carboxylesterase type B [EC:3.1.1.1] | | |
| K03928 | 9 | carboxylesterase [EC:3.1.1.1] | | |
| K03930 | 1 | putative tributyrin esterase [EC:3.1.1.-] | | |
| K03931 | 6 | putative isomerase | | |
| K03932 | 12 | polyhydroxybutyrate depolymerase | | |
| K03933 | 3 | chitin-binding protein | | |
| K03943 | 6 | NADH dehydrogenase (ubiquinone) flavoprotein 2 [EC:1.6.5.3 1.6.99.3] | | |
| K03969 | 18 | phage shock protein A | | |
| K03972 | 3 | phage shock protein E | | |
| K03973 | 8 | phage shock protein C | | |
| K03974 | 2 | psp operon transcriptional activator | | |
| K03975 | 32 | membrane-associated protein | | |
| K03977 | 56 | GTP-binding protein | | |
| K03978 | 19 | GTP-binding protein | | |
| K03979 | 43 | GTP-binding protein | | |
| K03980 | 50 | virulence factor | | |
| K03981 | 8 | thiol:disulfide interchange protein DsbC [EC:5.3.4.1] | | |
| K04014 | 24 | formate-dependent nitrite reductase, Fe-S protein | | |
| K04015 | 1 | formate-dependent nitrate reductase complex, transmembrane protein | | |
| K04016 | 4 | formate-dependent nitrite reductase, possible assembly protein | | |
| K04019 | 11 | ethanolamine utilization protein EutA | | |
| K04020 | 4 | phosphotransacetylase | | |
| K04021 | 1 | aldehyde dehydrogenase | | |
| K04027 | 5 | ethanolamine utilization protein EutM | | |
| K04028 | 4 | ethanolamine utilization protein EutN | | |
| K04033 | 1 | AraC family transcriptional regulator, ethanolamine operon | | |
| K04034 | 54 | anaerobic magnesium-protoporphyrin IX monomethyl ester cyclase | | |
| K04035 | 62 | magnesium-protoporphyrin IX monomethyl ester (oxidative) cyclase | | |
| K04041 | 6 | fructose-1,6-bisphosphatase III [EC:3.1.3.11] | | |
| K04042 | 55 | bifunctional protein GlmU [EC:2.7.7.23 2.3.1.157] | | |
| K04043 | 64 | molecular chaperone DnaK | | |
| K04044 | 18 | molecular chaperone HscA | | |
| K04045 | 1 | molecular chaperone HscC | | |
| K04046 | 1 | hypothetical chaperone protein | | |
| K04047 | 11 | starvation-inducible DNA-binding protein | | |

| | | | | |
|---|---|---|---|---|
| K04061 | 2 | flagellar biosynthesis protein | | |
| K04063 | 19 | osmotically inducible protein OsmC | | |
| K04065 | 12 | hyperosmotically inducible periplasmic protein | | |
| K04066 | 67 | primosomal protein N' (replication factor Y) (superfamily II | | |
| K04067 | 9 | primosomal replication protein N'' | | |
| K04069 | 36 | pyruvate formate lyase activating enzyme [EC:1.97.1.4] | | |
| K04070 | 12 | putative pyruvate formate lyase activating enzyme [EC:1.97.1.4] | | |
| K04072 | 15 | acetaldehyde dehydrogenase / alcohol dehydrogenase [EC:1.2.1.10 | | |
| K04073 | 3 | acetaldehyde dehydrogenase [EC:1.2.1.10] | | |
| K04074 | 6 | cell division initiation protein | | |
| K04075 | 46 | tRNA(Ile)-lysidine synthase [EC:6.3.4.-] | | |
| K04076 | 10 | Lon-like ATP-dependent protease [EC:3.4.21.-] | | |
| K04077 | 43 | chaperonin GroEL | | |
| K04078 | 11 | chaperonin GroES | | |
| K04079 | 17 | molecular chaperone HtpG | | |
| K04080 | 2 | molecular chaperone IbpA | | |
| K04082 | 9 | molecular chaperone HscB | | |
| K04083 | 6 | molecular chaperone Hsp33 | | |
| K04084 | 26 | thiol:disulfide interchange protein DsbD [EC:1.8.1.8] | | |
| K04085 | 8 | tRNA 2-thiouridine synthesizing protein A [EC:2.8.1.-] | | |
| K04087 | 11 | membrane protease subunit HflC [EC:3.4.-.-] | | |
| K04088 | 14 | membrane protease subunit HflK [EC:3.4.-.-] | | |
| K04090 | 44 | indolepyruvate ferredoxin oxidoreductase [EC:1.2.7.8] | | |
| K04091 | 50 | alkanesulfonate monooxygenase [EC:1.14.14.5] | | |
| K04094 | 26 | glucose inhibited division protein Gid | | |
| K04095 | 1 | cell filamentation protein | | |
| K04096 | 36 | DNA processing protein | | |
| K04097 | 7 | glutathione S-transferase [EC:2.5.1.18] | | |
| K04098 | 2 | hydroxyquinol 1,2-dioxygenase [EC:1.13.11.37] | | |
| K04103 | 3 | indolepyruvate decarboxylase [EC:4.1.1.74] | | |
| K04107 | 6 | 4-hydroxybenzoyl-CoA reductase subunit 1 [EC:1.3.99.20] | | |
| K04108 | 42 | 4-hydroxybenzoyl-CoA reductase subunit 2 [EC:1.3.99.20] | | |
| K04110 | 22 | benzoate-CoA ligase [EC:6.2.1.25] | | |
| K04112 | 1 | benzoyl-CoA reductase subunit [EC:1.3.99.15] | | |
| K04113 | 10 | benzoyl-CoA reductase subunit [EC:1.3.99.15] | | |
| K04114 | 29 | benzoyl-CoA reductase subunit [EC:1.3.99.15] | | |
| K04115 | 16 | benzoyl-CoA reductase subunit [EC:1.3.99.15] | | |
| K04117 | 32 | cyclohexanecarboxyl-CoA dehydrogenase [EC:1.3.99.-] | | |
| K04118 | 4 | pimeloyl-CoA dehydrogenase [EC:1.3.1.62] | | |
| K04126 | 1 | isopenicillin-N synthase [EC:1.21.3.1] | | |
| K04127 | 15 | isopenicillin-N epimerase [EC:5.1.1.17] | | |
| K04128 | 1 | hydroxymethyl cephem carbamoyltransferase [EC:2.1.3.-] | | |
| K04333 | 1 | LuxR family transcriptional regulator, csgAB operon transcriptional | | |
| K04340 | 1 | scyllo-inosamine-4-phosphate amidinotransferase 1 [EC:2.1.4.2] | | |
| K04477 | 11 | putative hydrolase | | |
| K04485 | 37 | DNA repair protein RadA/Sms | | |
| K04486 | 6 | histidinol-phosphatase (PHP family) [EC:3.1.3.15] | | |
| K04487 | 57 | cysteine desulfurase [EC:2.8.1.7] | | |
| K04488 | 14 | nitrogen fixation protein NifU and related proteins | | |
| K04496 | 9 | C-terminal binding protein | | |
| K04509 | 20 | formate dehydrogenase (cytochrome) [EC:1.2.2.1] | | |
| K04516 | 4 | chorismate mutase [EC:5.4.99.5] | | |
| K04517 | 16 | prephenate dehydrogenase [EC:1.3.1.12] | | |
| K04518 | 51 | prephenate dehydratase [EC:4.2.1.51] | | |
| K04561 | 21 | nitric-oxide reductase, cytochrome b-containing subunit I | | |
| K04562 | 441 | flagellar biosynthesis protein FlhG | | |
| K04564 | 27 | superoxide dismutase, Fe-Mn family [EC:1.15.1.1] | | |
| K04565 | 6 | Cu/Zn superoxide dismutase [EC:1.15.1.1] | | |
| K04566 | 6 | lysyl-tRNA synthetase, class I [EC:6.1.1.6] | | |

| | | | | |
|---|---|---|---|---|
| K04567 | 51 | lysyl-tRNA synthetase, class II [EC:6.1.1.6] | | |
| K04568 | 7 | lysyl-tRNA synthetase, class II [EC:6.1.1.6] | | |
| K04651 | 3 | hydrogenase nickel incorporation protein HypA | | |
| K04652 | 9 | hydrogenase nickel incorporation protein HypB | | |
| K04653 | 11 | hydrogenase expression/formation protein HypC | | |
| K04654 | 11 | hydrogenase expression/formation protein HypD | | |
| K04655 | 20 | hydrogenase expression/formation protein HypE | | |
| K04656 | 44 | hydrogenase maturation protein HypF | | |
| K04691 | 13 | serine protease DegS [EC:3.4.21.-] | | |
| K04719 | 4 | 5,6-dimethylbenzimidazole biosynthesis protein BluB | | |
| K04744 | 27 | LPS-assembly protein | | |
| K04748 | 8 | nitric-oxide reductase NorQ protein [EC:1.7.99.7] | | |
| K04749 | 22 | anti-sigma B factor antagonist | | |
| K04750 | 25 | PhnB protein | | |
| K04751 | 13 | nitrogen regulatory protein P-II 1 | | |
| K04752 | 4 | nitrogen regulatory protein P-II 2 | | |
| K04753 | 14 | suppressor of ftsI | | |
| K04754 | 6 | lipoprotein | | |
| K04755 | 9 | ferredoxin, 2Fe-2S | | |
| K04756 | 11 | alkyl hydroperoxide reductase subunit D | | |
| K04757 | 15 | anti-sigma B factor [EC:2.7.11.1] | | |
| K04758 | 2 | ferrous iron transport protein A | | |
| K04759 | 35 | ferrous iron transport protein B | | |
| K04760 | 10 | transcription elongation factor GreB | | |
| K04761 | 32 | LysR family transcriptional regulator, hydrogen peroxide-inducible | | |
| K04762 | 7 | ribosome-associated heat shock protein Hsp15 | | |
| K04763 | 93 | integrase/recombinase XerD | | |
| K04764 | 11 | integration host factor subunit alpha | | |
| K04766 | 1 | acetoin utilization protein AcuA [EC:2.3.1.-] | | |
| K04767 | 18 | acetoin utilization protein AcuB | | |
| K04768 | 10 | acetoin utilization protein AcuC | | |
| K04769 | 1 | AbrB family transcriptional regulator, stage V sporulation protein T | | |
| K04770 | 25 | Lon-like ATP-dependent protease [EC:3.4.21.-] | | |
| K04771 | 6 | serine protease Do [EC:3.4.21.107] | | |
| K04772 | 20 | serine protease DegQ [EC:3.4.21.-] | | |
| K04773 | 37 | protease IV [EC:3.4.21.-] | | |
| K04774 | 3 | serine protease SohB [EC:3.4.21.-] | | |
| K04780 | 19 | nonribosomal peptide synthetase DhbF | | |
| K04782 | 8 | isochorismate pyruvate-lyase [EC:4.1.3.-] | | |
| K04783 | 2 | yersiniabactin salicyl-AMP ligase [EC:6.3.2.-] | | |
| K04786 | 3 | yersiniabactin nonribosomal peptide/polyketide synthase | | |
| K04787 | 6 | mycobactin salicyl-AMP ligase [EC:6.3.2.-] | | |
| K04794 | 1 | peptidyl-tRNA hydrolase, PTH2 family [EC:3.1.1.29] | | |
| K05020 | 1 | glycine betaine transporter | | |
| K05275 | 26 | pyridoxine 4-dehydrogenase [EC:1.1.1.65] | | |
| K05281 | 4 | 2'-hydroxyisoflavone reductase [EC:1.3.1.45] | | |
| K05296 | 3 | 3(or 17)beta-hydroxysteroid dehydrogenase [EC:1.1.1.51] | | |
| K05297 | 6 | rubredoxin-NAD+ reductase [EC:1.18.1.1] | | |
| K05299 | 10 | formate dehydrogenase (NADP+) [EC:1.2.1.43] | | |
| K05301 | 55 | sulfite dehydrogenase [EC:1.8.2.1] | | |
| K05306 | 2 | phosphonoacetaldehyde hydrolase [EC:3.11.1.1] | | |
| K05311 | 1 | central glycolytic genes regulator | | |
| K05337 | 22 | ferredoxin | | |
| K05338 | 12 | holin-like protein | | |
| K05339 | 1 | holin-like protein LrgB | | |
| K05340 | 2 | glucose uptake protein | | |
| K05341 | 5 | amylosucrase [EC:2.4.1.4] | | |
| K05343 | 42 | maltose alpha-D-glucosyltransferase [EC:5.4.99.16] | | |
| K05344 | 1 | glucose-1-phosphate phosphodismutase [EC:2.7.1.41] | | |

| | | | | |
|---|---|---|---|---|
| K05345 | 21 | putative cyclase [EC:4.6.1.-] | | |
| K05348 | 2 | 2-hydroxycinnamic acid beta-D-glucosylisomelase [EC:5.2.1.-] | | |
| K05349 | 79 | beta-glucosidase [EC:3.2.1.21] | | |
| K05350 | 31 | beta-glucosidase [EC:3.2.1.21] | | |
| K05351 | 2 | D-xylulose reductase [EC:1.1.1.9] | | |
| K05358 | 3 | quinate dehydrogenase (pyrroloquinoline-quinone) [EC:1.1.99.25] | | |
| K05359 | 1 | carboxycyclohexadienyl dehydratase [EC:4.2.1.91] | | |
| K05362 | 1 | UDP-N-acetylmuramoyl-L-alanyl-D-glutamate-L-lysine ligase | | |
| K05363 | 1 | serine/alanine adding enzyme [EC:2.3.2.10] | | |
| K05364 | 58 | peptidoglycan glycosyltransferase [EC:2.4.1.129] | | |
| K05365 | 12 | penicillin-binding protein 1B [EC:2.4.1.129 3.4.-.-] | | |
| K05366 | 129 | penicillin-binding protein 1A [EC:2.4.1.- 3.4.-.-] | | |
| K05367 | 18 | penicillin-binding protein 1C [EC:2.4.1.-] | | |
| K05368 | 1 | aquacobalamin reductase / NAD(P)H-flavin reductase [EC:1.16.1.3 | | |
| K05375 | 2 | MbtH protein | | |
| K05384 | 22 | bilin biosynthesis protein | | |
| K05386 | 8 | bilin biosynthesis protein | | |
| K05394 | 29 | atrazine chlorohydrolase [EC:3.8.1.8] | | |
| K05395 | 5 | cystine reductase [EC:1.8.1.6] | | |
| K05396 | 10 | D-cysteine desulfhydrase [EC:4.4.1.15] | | |
| K05499 | 15 | LacI family transcriptional regulator, repressor for deo operon, | | |
| K05501 | 11 | TetR/AcrR family transcriptional regulator | | |
| K05515 | 85 | penicillin-binding protein 2 | | |
| K05516 | 22 | curved DNA-binding protein | | |
| K05517 | 1 | nucleoside-specific channel-forming protein | | |
| K05520 | 21 | protease I [EC:3.2.-.-] | | |
| K05521 | 16 | ADP-ribosylglycohydrolase [EC:3.2.-.-] | | |
| K05522 | 12 | endonuclease VIII [EC:3.2.2.- 4.2.99.18] | | |
| K05524 | 6 | ferredoxin | | |
| K05525 | 30 | linalool 8-monooxygenase [EC:1.14.99.28] | | |
| K05527 | 6 | BolA protein | | |
| K05539 | 2 | tRNA-dihydrouridine synthase A [EC:1.-.-.-] | | |
| K05540 | 28 | tRNA-dihydrouridine synthase B [EC:1.-.-.-] | | |
| K05541 | 1 | tRNA-dihydrouridine synthase C [EC:1.-.-.-] | | |
| K05549 | 11 | benzoate 1,2-dioxygenase alpha subunit [EC:1.14.12.10] | | |
| K05550 | 4 | benzoate 1,2-dioxygenase beta subunit [EC:1.14.12.10] | | |
| K05551 | 24 | 3-oxoacyl-ACP synthase I [EC:2.3.1.-] | | |
| K05552 | 22 | 3-oxoacyl-ACP synthase II [EC:2.3.1.-] | | |
| K05555 | 12 | cyclase [EC:4.-.-.-] | | |
| K05556 | 3 | hydroxylacyl-CoA dehydrogenase [EC:1.1.1.-] | | |
| K05557 | 16 | MFS transporter, DHA2 family, integral membrane protein | | |
| K05559 | 4 | multicomponent K+:H+ antiporter subunit A | | |
| K05561 | 2 | multicomponent K+:H+ antiporter subunit D | | |
| K05563 | 1 | multicomponent K+:H+ antiporter subunit F | | |
| K05564 | 1 | multicomponent K+:H+ antiporter subunit G | | |
| K05565 | 4 | multicomponent Na+:H+ antiporter subunit A | | |
| K05567 | 3 | multicomponent Na+:H+ antiporter subunit C | | |
| K05568 | 14 | multicomponent Na+:H+ antiporter subunit D | | |
| K05571 | 1 | multicomponent Na+:H+ antiporter subunit G | | |
| K05572 | 6 | NADH dehydrogenase I subunit 1 [EC:1.6.5.3] | | |
| K05573 | 9 | NADH dehydrogenase I subunit 2 [EC:1.6.5.3] | | |
| K05574 | 6 | NADH dehydrogenase I subunit 3 [EC:1.6.5.3] | | |
| K05575 | 8 | NADH dehydrogenase I subunit 4 [EC:1.6.5.3] | | |
| K05576 | 3 | NADH dehydrogenase I subunit 4L [EC:1.6.5.3] | | |
| K05577 | 7 | NADH dehydrogenase I subunit 5 [EC:1.6.5.3] | | |
| K05578 | 2 | NADH dehydrogenase I subunit 6 [EC:1.6.5.3] | | |
| K05579 | 2 | NADH dehydrogenase I subunit 7 [EC:1.6.5.3] | | |
| K05580 | 4 | NADH dehydrogenase I subunit I [EC:1.6.5.3] | | |
| K05586 | 3 | diaphorase subunit of the bidirectional hydrogenase [EC:1.6.5.3] | | |

| | | | | |
|---|---|---|---|---|
| K05587 | 6 | diaphorase subunit of the bidirectional hydrogenase [EC:1.6.5.3] | | |
| K05588 | 3 | diaphorase subunit of the bidirectional hydrogenase [EC:1.6.5.3] | | |
| K05589 | 1 | cell division protein FtsB | | |
| K05590 | 1 | ATP-dependent RNA helicase SrmB [EC:2.7.7.-] | | |
| K05591 | 4 | ATP-independent RNA helicase DbpA [EC:3.6.4.13] | | |
| K05592 | 36 | ATP-dependent RNA helicase DeaD [EC:3.6.4.13] | | |
| K05595 | 33 | multiple antibiotic resistance protein | | |
| K05597 | 1 | glutamin-(asparagin-)ase [EC:3.5.1.38] | | |
| K05599 | 2 | anthranilate 1,2-dioxygenase (deaminating, decarboxylating) large | | |
| K05600 | 2 | anthranilate 1,2-dioxygenase (deaminating, decarboxylating) small | | |
| K05601 | 2 | hydroxylamine reductase [EC:1.7.99.1] | | |
| K05603 | 10 | formimidoylglutamate deiminase [EC:3.5.3.13] | | |
| K05606 | 12 | methylmalonyl-CoA epimerase [EC:5.1.99.1] | | |
| K05685 | 60 | macrolide transport system ATP-binding/permease protein [EC:3.6.3.-] | | |
| K05692 | 2 | actin beta/gamma 1 | | |
| K05708 | 9 | large terminal subunit of phenylpropionate dioxygenase | | |
| K05709 | 3 | small terminal subunit of phenylpropionate dioxygenase | | |
| K05710 | 16 | ferredoxin subunit of phenylpropionate dioxygenase | | |
| K05712 | 33 | 3-(3-hydroxy-phenyl)propionate hydroxylase [EC:1.14.13.-] | | |
| K05714 | 5 | 2-hydroxy-6-ketonona-2,4-dienedioic acid hydrolase [EC:3.7.1.-] | | |
| K05715 | 6 | 2-phosphoglycerate kinase [EC:2.7.2.-] | | |
| K05772 | 8 | putative tungstate transport system substrate-binding protein | | |
| K05773 | 8 | putative tungstate transport system permease protein | | |
| K05776 | 4 | molybdate transport system ATP-binding protein | | |
| K05777 | 5 | putative thiamine transport system substrate-binding protein | | |
| K05780 | 2 | putative phosphonate transport system ATP-binding protein | | |
| K05781 | 1 | putative phosphonate transport system ATP-binding protein | | |
| K05782 | 6 | benzoate membrane transport protein | | |
| K05783 | 1 | 1,6-dihydroxycyclohexa-2,4-diene-1-carboxylate dehydrogenase | | |
| K05784 | 3 | benzoate 1,2-dioxygenase electron transfer component | | |
| K05785 | 1 | transcriptional antiterminator RfaH | | |
| K05786 | 10 | chloramphenicol-sensitive protein RarD | | |
| K05787 | 1 | DNA-binding protein HU-alpha | | |
| K05788 | 20 | integration host factor subunit beta | | |
| K05794 | 28 | tellurite resistance protein TerC | | |
| K05795 | 6 | tellurium resistance protein TerD | | |
| K05796 | 1 | electron transport protein HydN | | |
| K05797 | 3 | 4-cresol dehydrogenase (hydroxylating) [EC:1.17.99.1] | | |
| K05798 | 12 | LysR family transcriptional regulator, transcriptional activator for | | |
| K05799 | 23 | GntR family transcriptional regulator, transcriptional repressor for | | |
| K05800 | 2 | Lrp/AsnC family transcriptional regulator | | |
| K05802 | 8 | potassium efflux system protein KefA | | |
| K05803 | 4 | lipoprotein NlpI | | |
| K05807 | 19 | putative lipoprotein | | |
| K05808 | 17 | putative sigma-54 modulation protein | | |
| K05810 | 27 | conserved hypothetical protein | | |
| K05812 | 1 | conserved hypothetical protein | | |
| K05813 | 23 | sn-glycerol 3-phosphate transport system substrate-binding protein | | |
| K05814 | 17 | sn-glycerol 3-phosphate transport system permease protein | | |
| K05815 | 10 | sn-glycerol 3-phosphate transport system permease protein | | |
| K05816 | 7 | sn-glycerol 3-phosphate transport system ATP-binding protein | | |
| K05817 | 27 | LysR family transcriptional regulator, hca operon transcriptional | | |
| K05820 | 4 | MFS transporter, PPP family, 3-phenylpropionic acid transporter | | |
| K05823 | 1 | N-acetyldiaminopimelate deacetylase [EC:3.5.1.47] | | |
| K05825 | 2 | 2-aminoadipate transaminase [EC:2.6.1.-] | | |
| K05827 | 5 | lysine biosynthesis protein LysX | | |
| K05829 | 4 | N-acetyl-gamma-aminoadipyl-phosphate reductase [EC:1.2.1.-] | | |
| K05830 | 3 | acetylornithine/acetyl-lysine aminotransferase [EC:2.6.1.11 2.6.1.-] | | |
| K05831 | 2 | acetyl-lysine deacetylase [EC:3.5.1.-] | | |

| | | | | |
|---|---|---|---|---|
| K05832 | 1 | putative ABC transport system permease protein | | |
| K05834 | 11 | homoserine/homoserine lactone efflux protein | | |
| K05835 | 1 | threonine efflux protein | | |
| K05836 | 9 | GntR family transcriptional regulator, histidine utilization | | |
| K05837 | 55 | rod shape determining protein RodA | | |
| K05838 | 12 | putative thioredoxin | | |
| K05841 | 12 | sterol 3beta-glucosyltransferase [EC:2.4.1.173] | | |
| K05844 | 10 | ribosomal protein S6 modification protein | | |
| K05845 | 22 | osmoprotectant transport system substrate-binding protein | | |
| K05846 | 27 | osmoprotectant transport system permease protein | | |
| K05847 | 6 | osmoprotectant transport system ATP-binding protein | | |
| K05873 | 1 | adenylate cyclase, class 2 [EC:4.6.1.1] | | |
| K05874 | 21 | methyl-accepting chemotaxis protein I, serine sensor receptor | | |
| K05875 | 8 | methyl-accepting chemotaxis protein II, aspartate sensor receptor | | |
| K05876 | 2 | methyl-accepting chemotaxis protein III, ribose and galactose sensor | | |
| K05878 | 8 | dihydroxyacetone kinase, N-terminal domain [EC:2.7.1.-] | | |
| K05879 | 5 | dihydroxyacetone kinase, C-terminal domain [EC:2.7.1.-] | | |
| K05881 | 1 | PTS hybrid protein | | |
| K05882 | 45 | aryl-alcohol dehydrogenase (NADP+) [EC:1.1.1.91] | | |
| K05883 | 2 | Unclassified; E1.1.1.210 | | |
| K05885 | 35 | 2,5-diketo-D-gluconate reductase [EC:1.1.1.274] | | |
| K05886 | 5 | serine 3-dehydrogenase [EC:1.1.1.276] | | |
| K05888 | 11 | Unclassified; E1.1.2.- | | |
| K05889 | 3 | polyvinyl-alcohol dehydrogenase (acceptor) [EC:1.1.99.23] | | |
| K05893 | 3 | Unclassified; E1.3.1.35 | | |
| K05895 | 2 | precorrin-6X reductase [EC:1.3.1.54] | | |
| K05896 | 19 | segregation and condensation protein A | | |
| K05897 | 1 | Unclassified; E1.3.3.- | | |
| K05898 | 2 | 3-oxosteroid 1-dehydrogenase [EC:1.3.99.4] | | |
| K05899 | 2 | glycine oxidase [EC:1.4.3.19] | | |
| K05903 | 108 | NADH dehydrogenase (quinone) [EC:1.6.99.5] | | |
| K05905 | 17 | protein-disulfide reductase [EC:1.8.1.8] | | |
| K05909 | 5 | Unclassified; E1.10.3.2 | | |
| K05910 | 1 | NADH peroxidase [EC:1.11.1.1] | | |
| K05911 | 5 | quinone-reactive Ni/Fe-hydrogenase [EC:1.12.5.1] | | |
| K05912 | 20 | Unclassified; E1.12.-.- | | |
| K05914 | 35 | Unclassified; E1.13.12.7 | | |
| K05915 | 9 | Unclassified; E1.13.-.- | | |
| K05916 | 3 | nitric oxide dioxygenase [EC:1.14.12.17] | | |
| K05917 | 1 | cytochrome P450, family 51 (sterol 14-demethylase) [EC:1.14.13.70] | | |
| K05918 | 8 | Unclassified; E1.14.19.- | | |
| K05921 | 16 | 5-oxopent-3-ene-1,2,5-tricarboxylate decarboxylase / | | |
| K05922 | 19 | quinone-reactive Ni/Fe-hydrogenase large subunit [EC:1.12.5.1] | | |
| K05927 | 7 | quinone-reactive Ni/Fe-hydrogenase small subunit [EC:1.12.5.1] | | |
| K05928 | 8 | tocopherol O-methyltransferase [EC:2.1.1.95] | | |
| K05929 | 6 | phosphoethanolamine N-methyltransferase [EC:2.1.1.103] | | |
| K05934 | 6 | precorrin-3B C17-methyltransferase [EC:2.1.1.131] | | |
| K05936 | 4 | precorrin-4 C11-methyltransferase [EC:2.1.1.133] | | |
| K05939 | 12 | acyl-[acyl-carrier-protein]-phospholipid O-acyltransferase / | | |
| K05942 | 1 | Unclassified; E2.3.-.- | | |
| K05944 | 6 | Unclassified; E2.4.1.56 | | |
| K05945 | 1 | Unclassified; E2.4.1.58 | | |
| K05946 | 16 | N-acetylglucosaminyldiphosphoundecaprenol [EC:2.4.1.187] | | |
| K05949 | 5 | Unclassified; E2.4.1.227 | | |
| K05957 | 4 | L-glutamine:scyllo-inosose aminotransferase [EC:2.6.1.50] | | |
| K05961 | 1 | Unclassified; E2.7.1.145 | | |
| K05962 | 30 | protein-histidine pros-kinase [EC:2.7.13.1] | | |
| K05968 | 7 | Unclassified; E3.1.1.6 | | |
| K05969 | 8 | peptidyl-tRNA hydrolase [EC:3.1.1.29] | | |

| | | | | |
|---|---|---|---|---|
| K05971 | 43 | Unclassified; E3.1.1.61 | | |
| K05972 | 1 | Unclassified; E3.1.1.72 | | |
| K05973 | 12 | poly(3-hydroxybutyrate) depolymerase [EC:3.1.1.75] | | |
| K05979 | 11 | 2-phosphosulfolactate phosphatase [EC:3.1.3.71] | | |
| K05982 | 8 | deoxyribonuclease V [EC:3.1.21.7] | | |
| K05984 | 4 | excinuclease Cho [EC:3.1.25.-] | | |
| K05985 | 445 | ribonuclease M5 [EC:3.1.26.8] | | |
| K05993 | 29 | isochorismatase [EC:3.3.2.1] | | |
| K05994 | 2 | bacterial leucyl aminopeptidase [EC:3.4.11.10] | | |
| K05995 | 7 | dipeptidase E [EC:3.4.13.21] | | |
| K05996 | 15 | carboxypeptidase T [EC:3.4.17.18] | | |
| K05997 | 3 | Fe-S cluster assembly protein SufA | | |
| K05998 | 2 | pseudomonalisin [EC:3.4.21.100] | | |
| K05999 | 5 | xanthomonalisin [EC:3.4.21.101] | | |
| K06001 | 31 | tryptophan synthase beta chain [EC:4.2.1.20] | | |
| K06010 | 5 | Unclassified; E3.4.23.43 | | |
| K06013 | 29 | STE24 endopeptidase [EC:3.4.24.84] | | |
| K06015 | 85 | Unclassified; E3.5.1.81 | | |
| K06016 | 27 | N-carbamoyl-L-amino-acid hydrolase [EC:3.5.1.87] | | |
| K06019 | 4 | pyrophosphatase PpaX [EC:3.6.1.1] | | |
| K06020 | 53 | sulfate-transporting ATPase [EC:3.6.3.25] | | |
| K06021 | 11 | phosphate-transporting ATPase [EC:3.6.3.27] | | |
| K06022 | 6 | molybdate-transporting ATPase [EC:3.6.3.29] | | |
| K06023 | 16 | HPr kinase/phosphorylase [EC:2.7.11.- 2.7.4.-] | | |
| K06024 | 17 | segregation and condensation protein B | | |
| K06026 | 35 | Unclassified; E3.6.4.4 | | |
| K06027 | 29 | vesicle-fusing ATPase [EC:3.6.4.6] | | |
| K06031 | 6 | Unclassified; E3.8.1.1 | | |
| K06033 | 3 | Unclassified; E4.1.1.76 | | |
| K06034 | 5 | sulfopyruvate decarboxylase subunit alpha [EC:4.1.1.79] | | |
| K06037 | 1 | Unclassified; E4.2.3.- | | |
| K06039 | 4 | uncharacterized protein involved in oxidation of intracellular | | |
| K06041 | 24 | arabinose-5-phosphate isomerase [EC:5.3.1.13] | | |
| K06042 | 8 | precorrin-8X methylmutase [EC:5.4.1.2] | | |
| K06044 | 33 | (1->4)-alpha-D-glucan 1-alpha-D-glucosylmutase [EC:5.4.99.15] | | |
| K06045 | 23 | squalene-hopene cyclase [EC:5.4.99.17] | | |
| K06048 | 23 | carboxylate-amine ligase [EC:6.3.-.-] | | |
| K06049 | 10 | magnesium chelatase accessory protein | | |
| K06075 | 7 | MarR family transcriptional regulator, transcriptional regulator for | | |
| K06076 | 23 | long-chain fatty acid transport protein | | |
| K06077 | 1 | outer membrane lipoprotein SlyB | | |
| K06113 | 1 | arabinan endo-1,5-alpha-L-arabinosidase [EC:3.2.1.99] | | |
| K06118 | 5 | UDP-sulfoquinovose synthase [EC:3.13.1.1] | | |
| K06120 | 2 | glycerol dehydratase large subunit [EC:4.2.1.30] | | |
| K06121 | 1 | glycerol dehydratase medium subunit [EC:4.2.1.30] | | |
| K06131 | 35 | cardiolipin synthase [EC:2.7.8.-] | | |
| K06132 | 18 | putative cardiolipin synthase [EC:2.7.8.-] | | |
| K06134 | 3 | ubiquinone biosynthesis monooxygenase Coq7 [EC:1.14.13.-] | | |
| K06136 | 9 | pyrroloquinoline quinone biosynthesis protein B | | |
| K06137 | 5 | pyrroloquinoline-quinone synthase [EC:1.3.3.11] | | |
| K06138 | 1 | pyrroloquinoline quinone biosynthesis protein D | | |
| K06139 | 53 | pyrroloquinoline quinone biosynthesis protein E | | |
| K06140 | 4 | regulator of nucleoside diphosphate kinase | | |
| K06142 | 8 | outer membrane protein | | |
| K06143 | 10 | inner membrane protein | | |
| K06147 | 326 | ATP-binding cassette, subfamily B, bacterial | | |
| K06148 | 122 | ATP-binding cassette, subfamily C, bacterial | | |
| K06149 | 46 | universal stress protein A | | |
| K06151 | 40 | gluconate 2-dehydrogenase alpha chain [EC:1.1.99.3] | | |

| | | | | |
|---|---|---|---|---|
| K06153 | 39 | undecaprenyl-diphosphatase [EC:3.6.1.27] | | |
| K06155 | 4 | Gnt-I system high-affinity gluconate transporter | | |
| K06158 | 42 | ATP-binding cassette, sub-family F, member 3 | | |
| K06162 | 1 | PhnM protein | | |
| K06163 | 1 | PhnJ protein | | |
| K06164 | 3 | PhnI protein | | |
| K06167 | 14 | PhnP protein | | |
| K06168 | 67 | bifunctional enzyme involved in thiolation and methylation of tRNA | | |
| K06173 | 30 | tRNA pseudouridine synthase A [EC:5.4.99.12] | | |
| K06175 | 3 | tRNA pseudouridine synthase C [EC:5.4.99.12] | | |
| K06176 | 3 | tRNA pseudouridine synthase D [EC:5.4.99.12] | | |
| K06177 | 17 | ribosomal large subunit pseudouridine synthase A [EC:5.4.99.12] | | |
| K06178 | 41 | ribosomal large subunit pseudouridine synthase B [EC:5.4.99.12] | | |
| K06179 | 25 | ribosomal large subunit pseudouridine synthase C [EC:5.4.99.12] | | |
| K06180 | 45 | ribosomal large subunit pseudouridine synthase D [EC:5.4.99.12] | | |
| K06181 | 5 | ribosomal large subunit pseudouridine synthase E [EC:5.4.99.12] | | |
| K06182 | 2 | ribosomal large subunit pseudouridine synthase F [EC:5.4.99.12] | | |
| K06183 | 14 | ribosomal small subunit pseudouridine synthase A [EC:5.4.99.12] | | |
| K06186 | 4 | small protein A | | |
| K06187 | 21 | recombination protein RecR | | |
| K06188 | 15 | aquaporin Z | | |
| K06189 | 18 | magnesium and cobalt transporter | | |
| K06190 | 8 | intracellular septation protein | | |
| K06192 | 8 | paraquat-inducible protein B | | |
| K06193 | 1 | phosphonoacetate hydrolase [EC:3.11.1.2] | | |
| K06194 | 17 | lipoprotein NlpD | | |
| K06195 | 4 | ApaG protein | | |
| K06196 | 31 | cytochrome c-type biogenesis protein | | |
| K06197 | 3 | cation transport regulator | | |
| K06199 | 16 | CrcB protein | | |
| K06200 | 25 | carbon starvation protein | | |
| K06201 | 11 | copper homeostasis protein | | |
| K06202 | 1 | CyaY protein | | |
| K06204 | 29 | DnaK suppressor protein | | |
| K06205 | 2 | MioC protein | | |
| K06206 | 3 | sugar fermentation stimulation protein A | | |
| K06207 | 33 | GTP-binding protein | | |
| K06208 | 12 | chorismate mutase [EC:5.4.99.5] | | |
| K06211 | 1 | HipB family transcriptional regulator, involved in the regulation of | | |
| K06212 | 14 | formate transporter | | |
| K06213 | 42 | magnesium transporter | | |
| K06215 | 8 | pyridoxine biosynthesis protein [EC:4.-.-.-] | | |
| K06217 | 31 | phosphate starvation-inducible protein PhoH and related proteins | | |
| K06218 | 4 | RelE protein | | |
| K06219 | 4 | S-adenosylmethionine-dependent methyltransferase | | |
| K06221 | 8 | 2,5-diketo-D-gluconate reductase A [EC:1.1.1.274] | | |
| K06222 | 1 | 2,5-diketo-D-gluconate reductase B [EC:1.1.1.274] | | |
| K06223 | 4 | DNA adenine methylase [EC:2.1.1.72] | | |
| K06281 | 38 | hydrogenase large subunit [EC:1.12.99.6] | | |
| K06282 | 22 | hydrogenase small subunit [EC:1.12.99.6] | | |
| K06284 | 5 | transcriptional pleiotropic regulator of transition state genes | | |
| K06287 | 26 | septum formation protein | | |
| K06294 | 1 | spore germination protein D | | |
| K06306 | 15 | spore germination protein | | |
| K06308 | 1 | spore germination protein | | |
| K06320 | 3 | spore maturation protein CgeB | | |
| K06324 | 41 | spore coat protein A | | |
| K06329 | 1 | spore coat protein F | | |
| K06330 | 1 | spore coat protein H | | |

| | | | | |
|---|---|---|---|---|
| K06345 | 3 | spore cortex protein | | |
| K06346 | 9 | spoIIIJ-associated protein | | |
| K06348 | 5 | sporulation inhibitor KapD | | |
| K06350 | 5 | antagonist of KipI | | |
| K06351 | 3 | inhibitor of KinA | | |
| K06370 | 4 | morphogenetic protein associated with SpoVID | | |
| K06373 | 4 | spore maturation protein A | | |
| K06374 | 5 | spore maturation protein B | | |
| K06378 | 14 | stage II sporulation protein AA (anti-sigma F factor antagonist) | | |
| K06379 | 6 | stage II sporulation protein AB (anti-sigma F factor) [EC:2.7.11.1] | | |
| K06381 | 23 | stage II sporulation protein D | | |
| K06384 | 1 | stage II sporulation protein M | | |
| K06390 | 4 | stage III sporulation protein AA | | |
| K06391 | 1 | stage III sporulation protein AB | | |
| K06399 | 2 | stage IV sporulation protein B [EC:3.4.21.116] | | |
| K06400 | 21 | site-specific DNA recombinase | | |
| K06402 | 3 | stage IV sporulation protein FB [EC:3.4.24.-] | | |
| K06405 | 1 | stage V sporulation protein AC | | |
| K06407 | 1 | stage V sporulation protein AE | | |
| K06408 | 4 | stage V sporulation protein AF | | |
| K06412 | 4 | stage V sporulation protein G | | |
| K06413 | 5 | stage V sporulation protein K | | |
| K06415 | 10 | stage V sporulation protein R | | |
| K06419 | 1 | small acid-soluble spore protein B (major beta-type SASP) | | |
| K06422 | 11 | small acid-soluble spore protein E (minor gamma-type SASP) | | |
| K06423 | 1 | small acid-soluble spore protein F (minor alpha/beta-type SASP) | | |
| K06428 | 1 | small acid-soluble spore protein K (minor) | | |
| K06434 | 6 | small acid-soluble spore protein (thioredoxin-like protein) | | |
| K06442 | 20 | putative hemolysin | | |
| K06445 | 16 | acyl-CoA dehydrogenase [EC:1.3.99.-] | | |
| K06446 | 6 | acyl-CoA dehydrogenase [EC:1.3.99.-] | | |
| K06447 | 2 | succinylglutamic semialdehyde dehydrogenase [EC:1.2.1.71] | | |
| K06518 | 4 | holin-like protein | | |
| K06595 | 4 | heam-based aerotactic trancducer | | |
| K06596 | 35 | chemosensory pili system protein ChpA (sensor histidine | | |
| K06600 | 1 | chemosensory pili system protein ChpE | | |
| K06601 | 1 | flagellar protein FlbT | | |
| K06602 | 1 | flagellar protein FlaF | | |
| K06603 | 1 | flagellar protein FlaG | | |
| K06605 | 1 | myo-inositol catabolism protein IolH | | |
| K06606 | 2 | inosose isomerase [EC:5.3.99.-] | | |
| K06607 | 2 | myo-inositol catabolism protein IolS [EC:1.1.1.-] | | |
| K06608 | 3 | DeoR family transcriptional regulator, myo-inositol catabolism | | |
| K06609 | 1 | MFS transporter, SP family, major inositol transporter | | |
| K06714 | 8 | arginine utilization regulatory protein | | |
| K06718 | 2 | L-2,4-diaminobutyric acid acetyltransferase [EC:2.3.1.178] | | |
| K06726 | 1 | D-ribose pyranase [EC:5.-.-.-] | | |
| K06857 | 10 | putative tungstate transport system ATP-binding protein | | |
| K06858 | 8 | vitamin B12 transport system substrate-binding protein | | |
| K06859 | 1 | glucose-6-phosphate isomerase, archaeal [EC:5.3.1.9] | | |
| K06860 | 19 | Unclassified; K06860 | | |
| K06861 | 16 | lipopolysaccharide export system ATP-binding protein [EC:3.6.3.-] | | |
| K06864 | 13 | Unclassified; K06864 | | |
| K06867 | 34 | Unclassified; K06867 | | |
| K06871 | 16 | Unclassified; K06871 | | |
| K06872 | 14 | Unclassified; K06872 | | |
| K06873 | 3 | Unclassified; K06873 | | |
| K06876 | 2 | Unclassified; K06876 | | |
| K06877 | 43 | Unclassified; K06877 | | |

| | | | | |
|---|---|---|---|---|
| K06878 | 6 | Unclassified; K06878 | | |
| K06881 | 34 | Unclassified; K06881 | | |
| K06882 | 5 | Unclassified; K06882 | | |
| K06883 | 14 | Unclassified; K06883 | | |
| K06884 | 1 | Unclassified; K06884 | | |
| K06885 | 9 | Unclassified; K06885 | | |
| K06886 | 13 | hemoglobin | | |
| K06887 | 1 | Unclassified; K06887 | | |
| K06888 | 45 | Unclassified; K06888 | | |
| K06889 | 70 | Unclassified; K06889 | | |
| K06890 | 28 | Unclassified; K06890 | | |
| K06891 | 7 | ATP-dependent Clp protease adaptor protein ClpS | | |
| K06892 | 7 | Unclassified; K06892 | | |
| K06893 | 19 | Unclassified; K06893 | | |
| K06894 | 31 | Unclassified; K06894 | | |
| K06895 | 6 | Unclassified; K06895 | | |
| K06896 | 21 | Unclassified; K06896 | | |
| K06897 | 12 | Unclassified; K06897 | | |
| K06898 | 15 | Unclassified; K06898 | | |
| K06899 | 1 | Unclassified; K06899 | | |
| K06900 | 6 | Unclassified; K06900 | | |
| K06901 | 37 | putative MFS transporter, AGZA family, xanthine/uracil permease | | |
| K06902 | 19 | MFS transporter, UMF1 family | | |
| K06903 | 7 | Unclassified; K06903 | | |
| K06904 | 7 | Unclassified; K06904 | | |
| K06905 | 6 | Unclassified; K06905 | | |
| K06907 | 22 | Unclassified; K06907 | | |
| K06909 | 7 | Unclassified; K06909 | | |
| K06910 | 25 | Unclassified; K06910 | | |
| K06911 | 105 | Unclassified; K06911 | | |
| K06912 | 17 | alpha-ketoglutarate-dependent 2,4-dichlorophenoxyacetate dioxygenase | | |
| K06915 | 44 | Unclassified; K06915 | | |
| K06916 | 7 | Unclassified; K06916 | | |
| K06917 | 3 | tRNA 2-selenouridine synthase [EC:2.9.1.-] | | |
| K06919 | 3 | Unclassified; K06919 | | |
| K06920 | 21 | queuosine biosynthesis protein QueC | | |
| K06921 | 5 | Unclassified; K06921 | | |
| K06922 | 4 | Unclassified; K06922 | | |
| K06923 | 9 | Unclassified; K06923 | | |
| K06925 | 17 | Unclassified; K06925 | | |
| K06927 | 3 | Unclassified; K06927 | | |
| K06929 | 33 | Unclassified; K06929 | | |
| K06931 | 1 | Unclassified; K06931 | | |
| K06934 | 2 | Unclassified; K06934 | | |
| K06936 | 2 | Unclassified; K06936 | | |
| K06937 | 23 | Unclassified; K06937 | | |
| K06938 | 1 | Unclassified; K06938 | | |
| K06940 | 16 | Unclassified; K06940 | | |
| K06941 | 40 | ribosomal RNA large subunit methyltransferase N [EC:2.1.1.-] | | |
| K06942 | 31 | Unclassified; K06942 | | |
| K06944 | 9 | Unclassified; K06944 | | |
| K06947 | 1 | Unclassified; K06947 | | |
| K06948 | 2 | Unclassified; K06948 | | |
| K06949 | 40 | ribosome biogenesis GTPase [EC:3.6.1.-] | | |
| K06950 | 36 | Unclassified; K06950 | | |
| K06951 | 7 | Unclassified; K06951 | | |
| K06952 | 1 | Unclassified; K06952 | | |
| K06953 | 2 | Unclassified; K06953 | | |
| K06954 | 16 | Unclassified; K06954 | | |

| | | | | |
|---|---|---|---|---|
| K06955 | 5 | Unclassified; K06955 | | |
| K06956 | 7 | Unclassified; K06956 | | |
| K06958 | 20 | Unclassified; K06958 | | |
| K06959 | 17 | Unclassified; K06959 | | |
| K06960 | 10 | Unclassified; K06960 | | |
| K06962 | 2 | Unclassified; K06962 | | |
| K06966 | 34 | Unclassified; K06966 | | |
| K06967 | 6 | Unclassified; K06967 | | |
| K06968 | 3 | Unclassified; K06968 | | |
| K06969 | 37 | putative SAM-dependent methyltransferase | | |
| K06971 | 6 | Unclassified; K06971 | | |
| K06972 | 1 | Unclassified; K06972 | | |
| K06973 | 3 | Unclassified; K06973 | | |
| K06974 | 7 | Unclassified; K06974 | | |
| K06975 | 3 | Unclassified; K06975 | | |
| K06976 | 4 | Unclassified; K06976 | | |
| K06977 | 1 | Unclassified; K06977 | | |
| K06978 | 97 | Unclassified; K06978 | | |
| K06979 | 58 | Unclassified; K06979 | | |
| K06980 | 27 | Unclassified; K06980 | | |
| K06981 | 1 | Unclassified; K06981 | | |
| K06983 | 1 | Unclassified; K06983 | | |
| K06985 | 5 | aspartyl protease family protein | | |
| K06986 | 1 | Unclassified; K06986 | | |
| K06987 | 5 | Unclassified; K06987 | | |
| K06988 | 27 | Unclassified; K06988 | | |
| K06989 | 7 | aspartate dehydrogenase [EC:1.4.1.21] | | |
| K06990 | 27 | Unclassified; K06990 | | |
| K06991 | 3 | Unclassified; K06991 | | |
| K06992 | 1 | Unclassified; K06992 | | |
| K06994 | 80 | putative drug exporter of the RND superfamily | | |
| K06996 | 29 | Unclassified; K06996 | | |
| K06997 | 22 | Unclassified; K06997 | | |
| K06998 | 53 | Unclassified; K06998 | | |
| K06999 | 29 | Unclassified; K06999 | | |
| K07000 | 5 | Unclassified; K07000 | | |
| K07001 | 116 | Unclassified; K07001 | | |
| K07002 | 5 | Unclassified; K07002 | | |
| K07003 | 53 | Unclassified; K07003 | | |
| K07004 | 22 | Unclassified; K07004 | | |
| K07005 | 13 | Unclassified; K07005 | | |
| K07006 | 21 | Unclassified; K07006 | | |
| K07007 | 10 | Unclassified; K07007 | | |
| K07008 | 22 | Unclassified; K07008 | | |
| K07009 | 8 | Unclassified; K07009 | | |
| K07010 | 19 | putative glutamine amidotransferase | | |
| K07011 | 57 | Unclassified; K07011 | | |
| K07012 | 6 | Unclassified; K07012 | | |
| K07014 | 4 | Unclassified; K07014 | | |
| K07015 | 1 | Unclassified; K07015 | | |
| K07017 | 5 | Unclassified; K07017 | | |
| K07018 | 12 | Unclassified; K07018 | | |
| K07019 | 16 | Unclassified; K07019 | | |
| K07020 | 7 | Unclassified; K07020 | | |
| K07021 | 40 | Unclassified; K07021 | | |
| K07024 | 51 | Unclassified; K07024 | | |
| K07025 | 74 | putative hydrolase of the HAD superfamily | | |
| K07027 | 59 | Unclassified; K07027 | | |
| K07028 | 17 | Unclassified; K07028 | | |

| | | | | |
|---|---|---|---|---|
| K07029 | 47 | Unclassified; K07029 | | |
| K07030 | 32 | Unclassified; K07030 | | |
| K07031 | 8 | Unclassified; K07031 | | |
| K07032 | 15 | Unclassified; K07032 | | |
| K07033 | 8 | Unclassified; K07033 | | |
| K07034 | 6 | Unclassified; K07034 | | |
| K07037 | 17 | Unclassified; K07037 | | |
| K07038 | 7 | Unclassified; K07038 | | |
| K07039 | 2 | Unclassified; K07039 | | |
| K07040 | 23 | Unclassified; K07040 | | |
| K07042 | 16 | Unclassified; K07042 | | |
| K07043 | 16 | Unclassified; K07043 | | |
| K07044 | 5 | Unclassified; K07044 | | |
| K07045 | 131 | Unclassified; K07045 | | |
| K07046 | 35 | Unclassified; K07046 | | |
| K07047 | 88 | Unclassified; K07047 | | |
| K07048 | 14 | Unclassified; K07048 | | |
| K07050 | 14 | Unclassified; K07050 | | |
| K07051 | 5 | Unclassified; K07051 | | |
| K07052 | 60 | Unclassified; K07052 | | |
| K07053 | 27 | Unclassified; K07053 | | |
| K07054 | 12 | Unclassified; K07054 | | |
| K07056 | 37 | Unclassified; K07056 | | |
| K07057 | 1 | Unclassified; K07057 | | |
| K07058 | 51 | Unclassified; K07058 | | |
| K07062 | 8 | Unclassified; K07062 | | |
| K07063 | 2 | Unclassified; K07063 | | |
| K07064 | 11 | Unclassified; K07064 | | |
| K07065 | 4 | Unclassified; K07065 | | |
| K07067 | 12 | Unclassified; K07067 | | |
| K07068 | 21 | Unclassified; K07068 | | |
| K07071 | 23 | Unclassified; K07071 | | |
| K07072 | 2 | Unclassified; K07072 | | |
| K07074 | 1 | Unclassified; K07074 | | |
| K07075 | 6 | Unclassified; K07075 | | |
| K07076 | 2 | Unclassified; K07076 | | |
| K07077 | 14 | Unclassified; K07077 | | |
| K07078 | 30 | Unclassified; K07078 | | |
| K07079 | 38 | Unclassified; K07079 | | |
| K07080 | 41 | Unclassified; K07080 | | |
| K07081 | 8 | Unclassified; K07081 | | |
| K07082 | 28 | Unclassified; K07082 | | |
| K07083 | 18 | Unclassified; K07083 | | |
| K07085 | 16 | Unclassified; K07085 | | |
| K07086 | 2 | Unclassified; K07086 | | |
| K07088 | 17 | Unclassified; K07088 | | |
| K07089 | 22 | Unclassified; K07089 | | |
| K07090 | 87 | Unclassified; K07090 | | |
| K07091 | 25 | lipopolysaccharide export system permease protein | | |
| K07093 | 7 | Unclassified; K07093 | | |
| K07094 | 1 | Unclassified; K07094 | | |
| K07095 | 21 | Unclassified; K07095 | | |
| K07096 | 25 | Unclassified; K07096 | | |
| K07098 | 27 | Unclassified; K07098 | | |
| K07100 | 43 | Unclassified; K07100 | | |
| K07101 | 5 | Unclassified; K07101 | | |
| K07102 | 16 | Unclassified; K07102 | | |
| K07104 | 17 | Unclassified; K07104 | | |
| K07106 | 20 | N-acetylmuramic acid 6-phosphate etherase [EC:4.2.-.-] | | |

| | | | | |
|---|---|---|---|---|
| K07107 | 51 | Unclassified; K07107 | | |
| K07110 | 12 | Unclassified; K07110 | | |
| K07112 | 18 | Unclassified; K07112 | | |
| K07113 | 5 | Unclassified; K07113 | | |
| K07114 | 83 | Unclassified; K07114 | | |
| K07115 | 2 | Unclassified; K07115 | | |
| K07116 | 73 | Unclassified; K07116 | | |
| K07117 | 6 | Unclassified; K07117 | | |
| K07118 | 4 | Unclassified; K07118 | | |
| K07119 | 19 | Unclassified; K07119 | | |
| K07120 | 8 | Unclassified; K07120 | | |
| K07121 | 4 | Unclassified; K07121 | | |
| K07122 | 1 | Unclassified; K07122 | | |
| K07124 | 34 | Unclassified; K07124 | | |
| K07125 | 1 | Unclassified; K07125 | | |
| K07126 | 43 | Unclassified; K07126 | | |
| K07127 | 6 | 5-hydroxyisourate hydrolase [EC:3.5.2.17] | | |
| K07130 | 24 | Unclassified; K07130 | | |
| K07131 | 6 | Unclassified; K07131 | | |
| K07133 | 13 | Unclassified; K07133 | | |
| K07137 | 10 | Unclassified; K07137 | | |
| K07138 | 10 | Unclassified; K07138 | | |
| K07139 | 2 | Unclassified; K07139 | | |
| K07140 | 12 | Unclassified; K07140 | | |
| K07141 | 16 | Unclassified; K07141 | | |
| K07145 | 11 | Unclassified; K07145 | | |
| K07146 | 1 | Unclassified; K07146 | | |
| K07147 | 73 | Unclassified; K07147 | | |
| K07148 | 4 | Unclassified; K07148 | | |
| K07149 | 5 | Unclassified; K07149 | | |
| K07150 | 2 | Unclassified; K07150 | | |
| K07152 | 50 | Unclassified; K07152 | | |
| K07153 | 1 | Unclassified; K07153 | | |
| K07154 | 7 | Unclassified; K07154 | | |
| K07155 | 4 | quercetin 2,3-dioxygenase [EC:1.13.11.24] | | |
| K07156 | 4 | Unclassified; pcoC | | |
| K07157 | 17 | Unclassified; K07157 | | |
| K07160 | 7 | Unclassified; K07160 | | |
| K07161 | 34 | Unclassified; K07161 | | |
| K07164 | 13 | Unclassified; K07164 | | |
| K07165 | 1 | transmembrane sensor | | |
| K07168 | 7 | CBS domain-containing membrane protein | | |
| K07169 | 39 | FHA domain-containing protein | | |
| K07170 | 8 | GAF domain-containing protein | | |
| K07171 | 5 | Unclassified; K07171 | | |
| K07172 | 1 | Unclassified; K07172 | | |
| K07173 | 15 | S-ribosylhomocysteine lyase [EC:4.4.1.21] | | |
| K07175 | 20 | PhoH-like ATPase | | |
| K07177 | 6 | PDZ domain-containing protein | | |
| K07178 | 2 | RIO kinase 1 [EC:2.7.11.1] | | |
| K07180 | 31 | serine protein kinase | | |
| K07181 | 7 | putative signal transduction protein containing EAL and modified | | |
| K07182 | 60 | CBS domain-containing protein | | |
| K07183 | 14 | response regulator NasT | | |
| K07185 | 12 | tryptophan-rich sensory protein | | |
| K07186 | 2 | membrane protein | | |
| K07192 | 11 | flotillin | | |
| K07213 | 6 | Unclassified; K07213 | | |
| K07214 | 12 | enterochelin esterase and related enzymes | | |

| | | | | |
|---|---|---|---|---|
| K07216 | 14 | hemerythrin | | |
| K07217 | 5 | Mn-containing catalase | | |
| K07218 | 10 | nitrous oxidase accessory protein | | |
| K07219 | 16 | putative molybdopterin biosynthesis protein | | |
| K07220 | 19 | hypothetical protein | | |
| K07221 | 1 | outer membrane porin | | |
| K07222 | 30 | putative flavoprotein involved in K+ transport | | |
| K07223 | 13 | putative iron-dependent peroxidase | | |
| K07224 | 2 | putative lipoprotein | | |
| K07226 | 3 | hypothetical protein | | |
| K07228 | 2 | TrkA domain protein | | |
| K07229 | 3 | Unclassified; K07229 | | |
| K07232 | 6 | cation transport protein ChaC | | |
| K07233 | 1 | copper resistance protein B | | |
| K07234 | 7 | uncharacterized protein involved in response to NO | | |
| K07236 | 1 | tRNA 2-thiouridine synthesizing protein C | | |
| K07237 | 3 | tRNA 2-thiouridine synthesizing protein B | | |
| K07238 | 18 | zinc transporter, ZIP family | | |
| K07239 | 116 | heavy-metal exporter, HME family | | |
| K07240 | 47 | chromate transporter | | |
| K07241 | 10 | high-affinity nickel-transport protein | | |
| K07243 | 27 | high-affinity iron transporter | | |
| K07245 | 21 | putative copper resistance protein D | | |
| K07246 | 8 | tartrate dehydrogenase/decarboxylase / D-malate dehydrogenase | | |
| K07248 | 4 | lactaldehyde dehydrogenase / glycolaldehyde dehydrogenase | | |
| K07250 | 30 | 4-aminobutyrate aminotransferase / (S)-3-amino-2-methylpropionate | | |
| K07257 | 5 | spore coat polysaccharide biosynthesis protein SpsF | | |
| K07258 | 136 | D-alanyl-D-alanine carboxypeptidase (penicillin-binding protein 5/6) | | |
| K07259 | 26 | D-alanyl-D-alanine carboxypeptidase / | | |
| K07260 | 7 | D-alanyl-D-alanine carboxypeptidase [EC:3.4.16.4] | | |
| K07262 | 11 | D-alanyl-D-alanine endopeptidase (penicillin-binding protein 7) | | |
| K07263 | 230 | zinc protease [EC:3.4.99.-] | | |
| K07264 | 23 | 4-amino-4-deoxy-L-arabinose transferase [EC:2.-.-.-] | | |
| K07265 | 3 | capsular polysaccharide export protein | | |
| K07266 | 2 | capsular polysaccharide export protein | | |
| K07267 | 1 | porin | | |
| K07273 | 5 | lysozyme | | |
| K07274 | 6 | outer membrane protein | | |
| K07276 | 2 | hypothetical protein | | |
| K07277 | 60 | outer membrane protein | | |
| K07278 | 43 | outer membrane protein | | |
| K07279 | 41 | hypothetical protein | | |
| K07281 | 1 | Unclassified; K07281 | | |
| K07282 | 27 | poly-gamma-glutamate synthesis protein (capsule biosynthesis | | |
| K07283 | 1 | putative salt-induced outer membrane protein | | |
| K07284 | 9 | sortase A | | |
| K07285 | 2 | outer membrane lipoprotein | | |
| K07287 | 7 | lipoprotein-34 | | |
| K07288 | 17 | uncharacterized membrane protein | | |
| K07289 | 17 | AsmA protein | | |
| K07290 | 5 | hypothetical protein | | |
| K07300 | 25 | Ca2+:H+ antiporter | | |
| K07301 | 19 | Unclassified; yrbG | | |
| K07302 | 14 | isoquinoline 1-oxidoreductase, alpha subunit [EC:1.3.99.16] | | |
| K07303 | 95 | isoquinoline 1-oxidoreductase, beta subunit [EC:1.3.99.16] | | |
| K07304 | 33 | peptide-methionine (S)-S-oxide reductase [EC:1.8.4.11] | | |
| K07305 | 15 | peptide-methionine (R)-S-oxide reductase [EC:1.8.4.12] | | |
| K07306 | 10 | anaerobic dimethyl sulfoxide reductase subunit A [EC:1.8.99.-] | | |
| K07307 | 10 | anaerobic dimethyl sulfoxide reductase subunit B (DMSO reductase | | |

| | | | | |
|---|---|---|---|---|
| K07308 | 1 | anaerobic dimethyl sulfoxide reductase subunit C (DMSO reductase | | |
| K07309 | 1 | putative dimethyl sulfoxide reductase subunit YnfE [EC:1.8.99.-] | | |
| K07310 | 1 | putative dimethyl sulfoxide reductase subunit YnfF [EC:1.8.99.-] | | |
| K07313 | 3 | serine/threonine protein phosphatase 1 [EC:3.1.3.16] | | |
| K07315 | 66 | sigma-B regulation protein RsbU (phosphoserine phosphatase) | | |
| K07316 | 4 | adenine-specific DNA-methyltransferase [EC:2.1.1.72] | | |
| K07318 | 2 | adenine-specific DNA-methyltransferase [EC:2.1.1.72] | | |
| K07319 | 22 | putative adenine-specific DNA-methyltransferase [EC:2.1.1.72] | | |
| K07320 | 7 | putative adenine-specific DNA-methyltransferase [EC:2.1.1.72] | | |
| K07321 | 9 | CO dehydrogenase maturation factor | | |
| K07322 | 13 | regulator of cell morphogenesis and NO signaling | | |
| K07323 | 15 | putative toluene tolerance protein | | |
| K07326 | 7 | hemolysin activation/secretion protein | | |
| K07334 | 3 | proteic killer suppression protein | | |
| K07335 | 28 | basic membrane protein A and related proteins | | |
| K07336 | 1 | PKHD-type hydroxylase [EC:1.14.11.-] | | |
| K07337 | 3 | hypothetical protein | | |
| K07339 | 2 | hypothetical protein | | |
| K07341 | 6 | death on curing protein | | |
| K07343 | 3 | DNA transformation protein and related proteins | | |
| K07345 | 1 | major type 1 subunit fimbrin (pilin) | | |
| K07346 | 3 | fimbrial chaperone protein | | |
| K07347 | 11 | outer membrane usher protein | | |
| K07357 | 1 | type 1 fimbriae regulatory protein FimB | | |
| K07386 | 54 | putative endopeptidase [EC:3.4.24.-] | | |
| K07387 | 22 | putative metalloprotease [EC:3.4.24.-] | | |
| K07390 | 4 | monothiol glutaredoxin | | |
| K07391 | 65 | magnesium chelatase family protein | | |
| K07392 | 4 | AAA family ATPase | | |
| K07393 | 11 | putative glutathione S-transferase | | |
| K07395 | 4 | putative proteasome-type protease | | |
| K07396 | 1 | putative protein-disulfide isomerase | | |
| K07397 | 21 | putative redox protein | | |
| K07399 | 14 | cytochrome c biogenesis protein | | |
| K07400 | 2 | Fe/S biogenesis protein NfuA | | |
| K07401 | 1 | selenoprotein W-related protein | | |
| K07402 | 49 | xanthine dehydrogenase accessory factor | | |
| K07403 | 31 | membrane-bound serine protease (ClpP class) | | |
| K07404 | 25 | 6-phosphogluconolactonase [EC:3.1.1.31] | | |
| K07405 | 7 | alpha-amylase [EC:3.2.1.1] | | |
| K07406 | 10 | alpha-galactosidase [EC:3.2.1.22] | | |
| K07407 | 12 | alpha-galactosidase [EC:3.2.1.22] | | |
| K07442 | 6 | tRNA (adenine-N1-)-methyltransferase [EC:2.1.1.36] | | |
| K07443 | 9 | methylated-DNA-protein-cysteine methyltransferase related protein | | |
| K07444 | 21 | putative N6-adenine-specific DNA methylase | | |
| K07445 | 12 | putative DNA methylase | | |
| K07447 | 18 | putative holliday junction resolvase [EC:3.1.-.-] | | |
| K07448 | 1 | restriction system protein | | |
| K07450 | 3 | putative resolvase | | |
| K07452 | 2 | 5-methylcytosine-specific restriction enzyme B [EC:3.1.21.-] | | |
| K07454 | 6 | putative restriction endonuclease | | |
| K07455 | 9 | recombination protein RecT | | |
| K07456 | 34 | DNA mismatch repair protein MutS2 | | |
| K07457 | 3 | endonuclease III related protein | | |
| K07458 | 8 | DNA mismatch endonuclease, patch repair protein [EC:3.1.-.-] | | |
| K07459 | 2 | putative ATP-dependent endonuclease of the OLD family | | |
| K07460 | 17 | putative endonuclease | | |
| K07461 | 8 | putative endonuclease | | |
| K07462 | 59 | single-stranded-DNA-specific exonuclease [EC:3.1.-.-] | | |

| | | | | |
|---|---|---|---|---|
| K07464 | 3 | putative RecB family exonuclease | | |
| K07465 | 7 | putative RecB family exonuclease | | |
| K07467 | 1 | phage replication initiation protein | | |
| K07469 | 22 | aldehyde dehydrogenase (FAD-independent) [EC:1.2.99.7] | | |
| K07477 | 1 | translin | | |
| K07478 | 38 | putative ATPase | | |
| K07479 | 2 | putative DNA topoisomerase | | |
| K07480 | 1 | insertion element IS1 protein InsB | | |
| K07481 | 18 | transposase, IS5 family | | |
| K07482 | 19 | transposase, IS30 family | | |
| K07483 | 33 | transposase | | |
| K07484 | 48 | transposase | | |
| K07485 | 29 | transposase | | |
| K07486 | 162 | transposase | | |
| K07487 | 46 | transposase | | |
| K07488 | 4 | transposase | | |
| K07491 | 40 | putative transposase | | |
| K07492 | 38 | putative transposase | | |
| K07493 | 63 | putative transposase | | |
| K07494 | 80 | putative transposase | | |
| K07495 | 29 | putative transposase | | |
| K07496 | 10 | putative transposase | | |
| K07497 | 187 | putative transposase | | |
| K07498 | 4 | putative transposase | | |
| K07499 | 14 | putative transposase | | |
| K07501 | 8 | hypothetical protein | | |
| K07502 | 7 | hypothetical protein | | |
| K07503 | 8 | hypothetical protein | | |
| K07504 | 1 | hypothetical protein | | |
| K07505 | 1 | hypothetical protein | | |
| K07506 | 18 | AraC family transcriptional regulator | | |
| K07507 | 22 | putative Mg2+ transporter-C (MgtC) family protein | | |
| K07508 | 6 | acetyl-CoA acyltransferase 2 [EC:2.3.1.16] | | |
| K07516 | 43 | 3-hydroxyacyl-CoA dehydrogenase [EC:1.1.1.35] | | |
| K07519 | 9 | phthalate 4,5-dioxygenase [EC:1.14.12.7] | | |
| K07533 | 21 | foldase protein PrsA [EC:5.2.1.8] | | |
| K07534 | 4 | cyclohex-1-ene-1-carboxyl-CoA hydratase [EC:4.2.1.-] | | |
| K07535 | 35 | 2-hydroxycyclohexanecarboxyl-CoA dehydrogenase [EC:1.1.1.-] | | |
| K07537 | 4 | cyclohexa-1,5-dienecarbonyl-CoA hydratase [EC:4.2.1.100] | | |
| K07538 | 3 | 6-hydroxycyclohex-1-ene-1-carboxyl-CoA dehydrogenase [EC:1.1.1.-] | | |
| K07543 | 23 | benzylsuccinate CoA-transferase BbsE subunit [EC:2.8.3.15] | | |
| K07544 | 22 | benzylsuccinate CoA-transferase BbsF subunit [EC:2.8.3.15] | | |
| K07545 | 1 | (R)-benzylsuccinyl-CoA dehydro genase [EC:1.3.99.21] | | |
| K07546 | 8 | E-phenylitaconyl-CoA hydratase [EC:4.2.1.-] | | |
| K07547 | 16 | 2-[hydroxy(phenyl)methyl]-succinyl-CoA dehydrogenase BbsC subunit | | |
| K07548 | 10 | 2-[hydroxy(phenyl)methyl]-succinyl-CoA dehydrogenase BbsD subunit | | |
| K07550 | 12 | benzoylsuccinyl-CoA thiolase BbsB subunit [EC:2.3.1.-] | | |
| K07552 | 14 | MFS transporter, DHA1 family, bicyclomycin/chloramphenicol | | |
| K07559 | 1 | putative RNA 2'-phosphotransferase [EC:2.7.1.-] | | |
| K07560 | 41 | D-tyrosyl-tRNA(Tyr) deacylase [EC:3.1.-.-] | | |
| K07566 | 44 | putative translation factor | | |
| K07567 | 68 | TdcF protein | | |
| K07568 | 35 | S-adenosylmethionine:tRNA ribosyltransferase-isomerase [EC:5.-.-.-] | | |
| K07570 | 6 | general stress protein 13 | | |
| K07574 | 3 | putative RNA-binding protein containing KH domain | | |
| K07576 | 52 | metallo-beta-lactamase family protein | | |
| K07577 | 8 | putative mRNA 3-end processing factor | | |
| K07586 | 1 | hypothetical protein | | |
| K07588 | 27 | LAO/AO transport system kinase [EC:2.7.-.-] | | |

| | | | | |
|---|---|---|---|---|
| K07592 | 4 | LysR family transcriptional regulator, tdc operon transcriptional | | |
| K07636 | 65 | two-component system, OmpR family, phosphate regulon sensor | | |
| K07637 | 1 | two-component system, OmpR family, sensor histidine kinase PhoQ | | |
| K07638 | 12 | two-component system, OmpR family, osmolarity sensor histidine | | |
| K07639 | 2 | two-component system, OmpR family, sensor histidine kinase RstB | | |
| K07640 | 6 | two-component system, OmpR family, sensor histidine kinase CpxA | | |
| K07641 | 4 | two-component system, OmpR family, sensor histidine kinase CreC | | |
| K07642 | 11 | two-component system, OmpR family, sensor histidine kinase BaeS | | |
| K07643 | 3 | two-component system, OmpR family, sensor histidine kinase BasS | | |
| K07644 | 26 | two-component system, OmpR family, heavy metal sensor histidine | | |
| K07645 | 33 | two-component system, OmpR family, sensor histidine kinase QseC | | |
| K07646 | 67 | two-component system, OmpR family, sensor histidine kinase KdpD | | |
| K07647 | 4 | two-component system, OmpR family, sensor histidine kinase TorS | | |
| K07648 | 1 | two-component system, OmpR family, aerobic respiration control | | |
| K07649 | 29 | two-component system, OmpR family, sensor histidine kinase TctE | | |
| K07651 | 11 | two-component system, OmpR family, sensor histidine kinase ResE | | |
| K07652 | 42 | two-component system, OmpR family, sensor histidine kinase VicK | | |
| K07653 | 6 | two-component system, OmpR family, sensor histidine kinase MprB | | |
| K07654 | 5 | two-component system, OmpR family, sensor histidine kinase MtrB | | |
| K07656 | 1 | two-component system, OmpR family, sensor histidine kinase TrcS | | |
| K07657 | 68 | two-component system, OmpR family, phosphate regulon response | | |
| K07658 | 67 | two-component system, OmpR family, alkaline phosphatase synthesis | | |
| K07659 | 12 | two-component system, OmpR family, phosphate regulon response | | |
| K07660 | 1 | two-component system, OmpR family, response regulator PhoP | | |
| K07661 | 1 | two-component system, OmpR family, response regulator RstA | | |
| K07662 | 5 | two-component system, OmpR family, response regulator CpxR | | |
| K07663 | 1 | two-component system, OmpR family, catabolic regulation response | | |
| K07664 | 5 | two-component system, OmpR family, response regulator BaeR | | |
| K07665 | 23 | two-component system, OmpR family, copper resistance phosphate | | |
| K07666 | 3 | two-component system, OmpR family, response regulator QseB | | |
| K07667 | 53 | two-component system, OmpR family, KDP operon response regulator | | |
| K07668 | 44 | two-component system, OmpR family, response regulator VicR | | |
| K07669 | 30 | two-component system, OmpR family, response regulator MprA | | |
| K07670 | 18 | two-component system, OmpR family, response regulator MtrA | | |
| K07671 | 1 | two-component system, OmpR family, response regulator PrrA | | |
| K07672 | 1 | two-component system, OmpR family, response regulator TrcR | | |
| K07673 | 10 | two-component system, NarL family, nitrate/nitrite sensor histidine | | |
| K07674 | 4 | two-component system, NarL family, nitrate/nitrite sensor histidine | | |
| K07675 | 49 | two-component system, NarL family, sensor histidine kinase UhpB | | |
| K07677 | 11 | two-component system, NarL family, capsular synthesis sensor | | |
| K07678 | 26 | two-component system, NarL family, sensor histidine kinase BarA | | |
| K07679 | 4 | two-component system, NarL family, sensor histidine kinase EvgS | | |
| K07680 | 15 | two-component system, NarL family, sensor histidine kinase ComP | | |
| K07681 | 1 | two-component system, NarL family, vancomycin resistance sensor | | |
| K07682 | 15 | two-component system, NarL family, sensor histidine kinase DevS | | |
| K07683 | 4 | two-component system, NarL family, sensor histidine kinase NreB | | |
| K07684 | 73 | two-component system, NarL family, nitrate/nitrite response | | |
| K07685 | 6 | two-component system, NarL family, nitrate/nitrite response | | |
| K07686 | 7 | two-component system, NarL family, uhpT operon response regulator | | |
| K07687 | 9 | two-component system, NarL family, captular synthesis response | | |
| K07689 | 20 | two-component system, NarL family, invasion response regulator UvrY | | |
| K07690 | 5 | two-component system, NarL family, response regulator EvgA | | |
| K07691 | 24 | two-component system, NarL family, competent response regulator ComA | | |
| K07692 | 37 | two-component system, NarL family, response regulator DegU | | |
| K07693 | 3 | two-component system, NarL family, response regulator DesR | | |
| K07694 | 4 | two-component system, NarL family, vancomycin resistance associated | | |
| K07695 | 13 | two-component system, NarL family, response regulator DevR | | |
| K07696 | 4 | two-component system, NarL family, response regulator NreC | | |
| K07697 | 11 | two-component system, sporulation sensor kinase B [EC:2.7.13.3] | | |

| | | | | |
|---|---|---|---|---|
| K07699 | 1 | two-component system, response regulator, stage 0 sporulation | | |
| K07700 | 2 | two-component system, CitB family, cit operon sensor histidine | | |
| K07703 | 1 | two-component system, CitB family, response regulator DcuR | | |
| K07704 | 7 | two-component system, LytT family, sensor histidine kinase LytS | | |
| K07705 | 5 | two-component system, LytT family, response regulator LytT | | |
| K07707 | 4 | two-component system, AgrA family, response regulator AgrA | | |
| K07708 | 54 | two-component system, NtrC family, nitrogen regulation sensor | | |
| K07709 | 54 | two-component system, NtrC family, sensor histidine kinase HydH | | |
| K07710 | 39 | two-component system, NtrC family, sensor histidine kinase AtoS | | |
| K07711 | 10 | two-component system, NtrC family, sensor histidine kinase YfhK | | |
| K07712 | 112 | two-component system, NtrC family, nitrogen regulation response | | |
| K07713 | 64 | two-component system, NtrC family, response regulator HydG | | |
| K07714 | 48 | two-component system, NtrC family, response regulator AtoC | | |
| K07715 | 78 | two-component system, NtrC family, response regulator YfhA | | |
| K07716 | 16 | two-component system, cell cycle sensor histidine kinase PleC | | |
| K07718 | 1 | two-component system, sensor histidine kinase YesM [EC:2.7.13.3] | | |
| K07720 | 7 | two-component system, response regulator YesN | | |
| K07726 | 5 | putative transcriptional regulator | | |
| K07727 | 1 | putative transcriptional regulator | | |
| K07729 | 7 | putative transcriptional regulator | | |
| K07734 | 8 | transcriptional regulator | | |
| K07735 | 24 | putative transcriptional regulator | | |
| K07736 | 12 | CarD family transcriptional regulator | | |
| K07737 | 5 | putative transcriptional regulator | | |
| K07738 | 6 | transcriptional repressor NrdR | | |
| K07739 | 4 | elongator complex protein 3 [EC:2.3.1.48] | | |
| K07740 | 1 | regulator of sigma D | | |
| K07741 | 1 | anti-repressor protein | | |
| K07742 | 7 | hypothetical protein | | |
| K07743 | 2 | hypothetical protein | | |
| K07745 | 1 | hypothetical protein | | |
| K07746 | 2 | hypothetical protein | | |
| K07748 | 2 | sterol-4alpha-carboxylate 3-dehydrogenase (decarboxylating) | | |
| K07749 | 163 | formyl-CoA transferase [EC:2.8.3.16] | | |
| K07758 | 5 | pyridoxal phosphatase [EC:3.1.3.74] | | |
| K07768 | 3 | two-component system, OmpR family, sensor histidine kinase SenX3 | | |
| K07769 | 5 | two-component system, OmpR family, sensor histidine kinase NblS | | |
| K07771 | 1 | two-component system, OmpR family, response regulator BasR | | |
| K07773 | 1 | two-component system, OmpR family, aerobic respiration control | | |
| K07774 | 14 | two-component system, OmpR family, response regulator TctD | | |
| K07775 | 5 | two-component system, OmpR family, response regulator ResD | | |
| K07776 | 43 | two-component system, OmpR family, response regulator RegX3 | | |
| K07777 | 14 | two-component system, NarL family, sensor histidine kinase DegS | | |
| K07778 | 10 | two-component system, NarL family, sensor histidine kinase DesK | | |
| K07782 | 1 | LuxR family transcriptional regulator | | |
| K07783 | 2 | MFS transporter, OPA family, sugar phosphate sensor protein UhpC | | |
| K07785 | 1 | MFS transporter, NRE family, putaive nickel resistance protein | | |
| K07787 | 112 | Cu(I)/Ag(I) efflux system membrane protein CusA | | |
| K07788 | 66 | RND superfamily, multidrug transport protein MdtB | | |
| K07789 | 37 | RND superfamily, multidrug transport protein MdtC | | |
| K07790 | 1 | putative membrane protein PagO | | |
| K07791 | 3 | anaerobic C4-dicarboxylate transporter DcuA | | |
| K07792 | 4 | anaerobic C4-dicarboxylate transporter DcuB | | |
| K07793 | 90 | putative tricarboxylic transport membrane protein | | |
| K07795 | 87 | putative tricarboxylic transport membrane protein | | |
| K07796 | 16 | Cu(I)/Ag(I) efflux system outer membrane protein CusC | | |
| K07798 | 51 | Cu(I)/Ag(I) efflux system membrane protein CusB | | |
| K07799 | 33 | putative multidrug efflux transporter MdtA | | |
| K07805 | 1 | putative membrane protein PagD | | |

| | | | | |
|---|---|---|---|---|
| K07806 | 20 | UDP-4-amino-4-deoxy-L-arabinose-oxoglutarate aminotransferase | | |
| K07811 | 3 | trimethylamine-N-oxide reductase (cytochrome c) 1 [EC:1.7.2.3] | | |
| K07812 | 12 | trimethylamine-N-oxide reductase (cytochrome c) 2 [EC:1.7.2.3] | | |
| K07814 | 158 | putative two-component system response regulator | | |
| K07821 | 1 | trimethylamine-N-oxide reductase (cytochrome c) 2, cytochrome c-type | | |
| K07823 | 4 | 3-oxoadipyl-CoA thiolase [EC:2.3.1.174] | | |
| K07824 | 6 | benzoate 4-monooxygenase [EC:1.14.13.12] | | |
| K07978 | 4 | GntR family transcriptional regulator | | |
| K07979 | 9 | GntR family transcriptional regulator | | |
| K08068 | 1 | N-acetylglucosamine-6-phosphate 2-epimerase and phosphatase | | |
| K08070 | 95 | 2-alkenal reductase [EC:1.3.1.74] | | |
| K08078 | 2 | 3a,7a,12a-trihydroxy-5b-cholest-24-enoyl-CoA hydratase | | |
| K08082 | 4 | two-component system, LytT family, sensor histidine kinase AlgZ | | |
| K08083 | 11 | two-component system, LytT family, response regulator AlgR | | |
| K08084 | 6 | type IV fimbrial biogenesis protein FimT | | |
| K08085 | 2 | type IV fimbrial biogenesis protein FimU | | |
| K08093 | 1 | 3-hexulose-6-phosphate synthase [EC:4.1.2.43] | | |
| K08097 | 6 | phosphosulfolactate synthase [EC:4.4.1.19] | | |
| K08100 | 49 | bilirubin oxidase [EC:1.3.3.5] | | |
| K08137 | 1 | MFS transporter, SP family, galactose:H+ symporter | | |
| K08138 | 2 | MFS transporter, SP family, xylose:H+ symportor | | |
| K08139 | 13 | MFS transporter, SP family, sugar:H+ symporter | | |
| K08151 | 22 | MFS transporter, DHA1 family, tetracycline resistance protein | | |
| K08152 | 3 | MFS transporter, DHA1 family, multidrug resistance protein B | | |
| K08153 | 11 | MFS transporter, DHA1 family, multidrug resistance protein | | |
| K08154 | 1 | MFS transporter, DHA1 family, 2-module integral membrane pump EmrD | | |
| K08156 | 5 | MFS transporter, DHA1 family, arabinose polymer transporter | | |
| K08159 | 2 | MFS transporter, DHA1 family, | | |
| K08161 | 7 | MFS transporter, DHA1 family, multidrug resistance protein | | |
| K08162 | 18 | MFS transporter, DHA1 family, multidrug resistance protein | | |
| K08166 | 40 | MFS transporter, DHA2 family, methylenomycin A resistance protein | | |
| K08167 | 18 | MFS transporter, DHA2 family, methyl viologen resistance protein | | |
| K08169 | 16 | MFS transporter, DHA2 family, multidrug resistance protein | | |
| K08172 | 10 | MFS transporter, MHS family, shikimate and dehydroshikimate | | |
| K08173 | 7 | MFS transporter, MHS family, metabolite:H+ symporter | | |
| K08177 | 46 | MFS transporter, OFA family, oxalate/formate antiporter | | |
| K08178 | 5 | MFS transporter, SHS family, lactate transporter | | |
| K08191 | 15 | MFS transporter, ACS family, hexuronate transporter | | |
| K08194 | 23 | MFS transporter, ACS family, D-galactonate transporter | | |
| K08195 | 3 | MFS transporter, AAHS family, 4-hydroxybenzoate transporter | | |
| K08196 | 5 | MFS transporter, AAHS family, cis,cis-muconate transporter | | |
| K08217 | 15 | MFS transporter, DHA3 family, macrolide efflux protein | | |
| K08218 | 18 | MFS transporter, PAT family, beta-lactamase induction signal | | |
| K08221 | 3 | MFS transporter, ACDE family, multidrug resistance protein | | |
| K08223 | 20 | MFS transporter, FSR family, fosmidomycin resistance protein | | |
| K08224 | 9 | MFS transporter, YNFM family, putative membrane transport protein | | |
| K08225 | 95 | MFS transporter, ENTS family, enterobactin (siderophore) exporter | | |
| K08226 | 1 | MFS transporter, BCD family, chlorophyll transporter | | |
| K08227 | 12 | MFS transporter, LPLT family, lysophospholipid transporter | | |
| K08234 | 21 | glyoxylase I family protein | | |
| K08252 | 50 | receptor protein-tyrosine kinase [EC:2.7.10.1] | | |
| K08253 | 118 | non-specific protein-tyrosine kinase [EC:2.7.10.2] | | |
| K08256 | 27 | phosphatidylinositol alpha-mannosyltransferase [EC:2.4.1.57] | | |
| K08259 | 50 | lysostaphin [EC:3.4.24.75] | | |
| K08261 | 13 | D-sorbitol dehydrogenase (acceptor) [EC:1.1.99.21] | | |
| K08278 | 4 | thr operon leader peptide | | |
| K08279 | 14 | carnitine operon protein CaiE | | |
| K08281 | 14 | nicotinamidase/pyrazinamidase [EC:3.5.1.19 3.5.1.-] | | |
| K08282 | 355 | non-specific serine/threonine protein kinase [EC:2.7.11.1] | | |

| | | | | |
|---|---|---|---|---|
| K08289 | 4 | phosphoribosylglycinamide formyltransferase 2 [EC:2.1.2.2] | | |
| K08295 | 5 | 2-aminobenzoate-CoA ligase [EC:6.2.1.32] | | |
| K08296 | 12 | phosphohistidine phosphatase [EC:3.1.3.-] | | |
| K08297 | 2 | crotonobetainyl-CoA dehydrogenase [EC:1.3.99.-] | | |
| K08299 | 6 | carnitinyl-CoA dehydratase [EC:4.2.1.-] | | |
| K08300 | 19 | ribonuclease E [EC:3.1.26.12] | | |
| K08301 | 25 | ribonuclease G [EC:3.1.26.-] | | |
| K08302 | 1 | tagatose 1,6-diphosphate aldolase [EC:4.1.2.40] | | |
| K08303 | 23 | putative protease [EC:3.4.-.-] | | |
| K08304 | 13 | membrane-bound lytic murein transglycosylase A [EC:3.2.1.-] | | |
| K08305 | 5 | membrane-bound lytic murein transglycosylase B [EC:3.2.1.-] | | |
| K08307 | 30 | membrane-bound lytic murein transglycosylase D [EC:3.2.1.-] | | |
| K08309 | 44 | soluble lytic murein transglycosylase [EC:3.2.1.-] | | |
| K08310 | 5 | dATP pyrophosphohydrolase [EC:3.6.1.-] | | |
| K08311 | 4 | putative (di)nucleoside polyphosphate hydrolase [EC:3.6.1.-] | | |
| K08316 | 6 | ribosomal RNA small subunit methyltransferase D [EC:2.1.1.52] | | |
| K08318 | 2 | putative dehydrogenase [EC:1.1.-.-] | | |
| K08319 | 2 | putative dehydrogenase [EC:1.1.-.-] | | |
| K08320 | 3 | CTP pyrophosphohydrolase [EC:3.6.1.-] | | |
| K08321 | 3 | putative autoinducer-2 (AI-2) aldolase [EC:4.1.2.-] | | |
| K08322 | 8 | starvation sensing protein RspB [EC:1.1.1.-] | | |
| K08323 | 9 | starvation sensing protein RspA | | |
| K08324 | 3 | aldehyde dehydrogenase family protein [EC:1.2.1.-] | | |
| K08326 | 1 | aminopeptidase [EC:3.4.11.-] | | |
| K08344 | 1 | suppressor for copper-sensitivity B | | |
| K08345 | 2 | nitrate reductase 2, alpha subunit [EC:1.7.99.4] | | |
| K08346 | 6 | nitrate reductase 2, beta subunit [EC:1.7.99.4] | | |
| K08348 | 39 | formate dehydrogenase-N, alpha subunit [EC:1.2.1.2] | | |
| K08351 | 5 | biotin sulfoxide reductase [EC:1.-.-.-] | | |
| K08352 | 38 | thiosulfate reductase [EC:1.-.-.-] | | |
| K08353 | 5 | thiosulfate reductase electron transport protein | | |
| K08354 | 3 | thiosulfate reductase cytochrome b subunit | | |
| K08356 | 11 | arsenite oxidase large subunit [EC:1.20.98.1] | | |
| K08357 | 9 | tetrathionate reductase subunit A | | |
| K08358 | 27 | tetrathionate reductase subunit B | | |
| K08359 | 4 | tetrathionate reductase subunit C | | |
| K08364 | 10 | periplasmic mercuric ion binding protein | | |
| K08365 | 3 | MerR family transcriptional regulator, mercuric resistance operon | | |
| K08368 | 1 | MFS transporter, putative metabolite transport protein | | |
| K08369 | 27 | MFS transporter, putative metabolite:H+ symporter | | |
| K08372 | 45 | putative serine protease PepD [EC:3.4.21.-] | | |
| K08384 | 8 | stage V sporulation protein D (sporulation-specific | | |
| K08477 | 2 | outer membrane protease E [EC:3.4.21.-] | | |
| K08478 | 1 | phosphoglycerate transport regulatory protein PgtC | | |
| K08479 | 3 | two-component system, OmpR family, clock-associated histidine kinase | | |
| K08481 | 4 | circadian clock protein KaiB | | |
| K08482 | 37 | circadian clock protein KaiC | | |
| K08483 | 32 | phosphotransferase system, enzyme I, PtsI [EC:2.7.3.9] | | |
| K08484 | 7 | phosphotransferase system, enzyme I, PtsP [EC:2.7.3.9] | | |
| K08485 | 1 | phosphocarrier protein NPr | | |
| K08567 | 9 | hydrogenase 2 maturation protease [EC:3.4.24.-] | | |
| K08590 | 4 | carbon-nitrogen hydrolase family protein | | |
| K08591 | 25 | glycerol-3-phosphate acyltransferase PlsY [EC:2.3.1.15] | | |
| K08600 | 2 | sortase B | | |
| K08602 | 47 | oligoendopeptidase F [EC:3.4.24.-] | | |
| K08603 | 6 | thermolysin [EC:3.4.24.27] | | |
| K08605 | 1 | coccolysin [EC:3.4.24.30] | | |
| K08641 | 17 | D-alanyl-D-alanine dipeptidase [EC:3.4.13.-] | | |
| K08651 | 23 | thermitase [EC:3.4.21.66] | | |

| | | | | |
|---|---|---|---|---|
| K08676 | 43 | tricorn protease [EC:3.4.21.-] | | |
| K08677 | 19 | kumamolisin | | |
| K08678 | 5 | UDP-glucuronate decarboxylase [EC:4.1.1.35] | | |
| K08679 | 5 | UDP-glucuronate 4-epimerase [EC:5.1.3.6] | | |
| K08680 | 8 | 2-succinyl-6-hydroxy-2,4-cyclohexadiene-1-carboxylate synthase | | |
| K08681 | 4 | glutamine amidotransferase [EC:2.6.-.-] | | |
| K08682 | 2 | acyl carrier protein phosphodiesterase [EC:3.1.4.14] | | |
| K08684 | 2 | methane monooxygenase [EC:1.14.13.25] | | |
| K08686 | 15 | 2-chlorobenzoate 1,2-dioxygenase [EC:1.14.12.13] | | |
| K08687 | 4 | N-carbamoylsarcosine amidase [EC:3.5.1.59] | | |
| K08688 | 9 | creatinase [EC:3.5.3.3] | | |
| K08693 | 4 | 3'-nucleotidase [EC:3.1.3.6] | | |
| K08697 | 2 | carbon dioxide concentrating mechanism protein CcmL | | |
| K08698 | 1 | carbon dioxide concentrating mechanism protein CcmM | | |
| K08710 | 5 | N-isopropylammelide isopropylaminohydrolase [EC:3.5.99.4] | | |
| K08714 | 2 | voltage-gated sodium channel | | |
| K08720 | 3 | outer membrane protein OmpU | | |
| K08721 | 3 | multidrug resistance outer membrane protein OprJ | | |
| K08723 | 4 | 5'-nucleotidase [EC:3.1.3.5] | | |
| K08724 | 5 | penicillin-binding protein 2B | | |
| K08738 | 12 | cytochrome c | | |
| K08777 | 1 | neutral peptidase B [EC:3.4.24.-] | | |
| K08884 | 560 | serine/threonine protein kinase, bacterial [EC:2.7.11.1] | | |
| K08900 | 1 | mitochondrial chaperone BCS1 | | |
| K08942 | 8 | photosystem P840 reaction center cytochrome c551 | | |
| K08963 | 23 | methylthioribose-1-phosphate isomerase [EC:5.3.1.23] | | |
| K08964 | 2 | methylthioribulose-1-phosphate dehydratase [EC:4.2.1.109] | | |
| K08965 | 1 | 2,3-diketo-5-methylthiopentyl-1-phosphate enolase [EC:3.1.3.77] | | |
| K08966 | 3 | 2-hydroxy-3-keto-5-methylthiopentenyl-1-phosphate phosphatase | | |
| K08967 | 4 | 1,2-dihydroxy-3-keto-5-methylthiopentene dioxygenase [EC:1.13.11.53 | | |
| K08969 | 1 | aminotransferase [EC:2.6.1.-] | | |
| K08970 | 5 | nickel/cobalt exporter | | |
| K08972 | 5 | putative membrane protein | | |
| K08973 | 5 | putative membrane protein | | |
| K08974 | 3 | putative membrane protein | | |
| K08977 | 3 | putative membrane protein | | |
| K08978 | 11 | putative membrane protein | | |
| K08981 | 2 | putative membrane protein | | |
| K08983 | 1 | putative membrane protein | | |
| K08984 | 7 | putative membrane protein | | |
| K08986 | 2 | putative membrane protein | | |
| K08987 | 2 | putative membrane protein | | |
| K08988 | 7 | putative membrane protein | | |
| K08989 | 3 | putative membrane protein | | |
| K08990 | 1 | putative membrane protein | | |
| K08992 | 1 | putative membrane protein | | |
| K08995 | 6 | putative membrane protein | | |
| K08997 | 11 | hypothetical protein | | |
| K08998 | 13 | hypothetical protein | | |
| K08999 | 19 | hypothetical protein | | |
| K09001 | 23 | anhydro-N-acetylmuramic acid kinase [EC:2.7.1.-] | | |
| K09003 | 8 | hypothetical protein | | |
| K09004 | 3 | hypothetical protein | | |
| K09005 | 16 | hypothetical protein | | |
| K09007 | 8 | hypothetical protein | | |
| K09008 | 4 | hypothetical protein | | |
| K09009 | 2 | hypothetical protein | | |
| K09011 | 7 | D-citramalate synthase [EC:2.3.1.182] | | |
| K09012 | 6 | DeoR family transcriptional regulator, suf operon transcriptional | | |

| | | | | |
|---|---|---|---|---|
| K09013 | 19 | Fe-S cluster assembly ATP-binding protein | | |
| K09014 | 39 | Fe-S cluster assembly protein SufB | | |
| K09015 | 22 | Fe-S cluster assembly protein SufD | | |
| K09016 | 3 | putative pyrimidine permease RutG | | |
| K09017 | 38 | TetR/AcrR family transcriptional regulator | | |
| K09018 | 1 | putative monooxygenase RutA [EC:1.14.-.-] | | |
| K09019 | 4 | putative NADH dehydrogenase/NAD(P)H nitroreductase RutE [EC:1.-.-.-] | | |
| K09020 | 6 | putative isochorismatase family protein RutB [EC:3.-.-.-] | | |
| K09022 | 1 | UPF0076 protein YjgF | | |
| K09023 | 5 | protein RutD | | |
| K09024 | 2 | putative flavin reductase RutF [EC:1.5.1.-] | | |
| K09065 | 3 | N-acetylornithine carbamoyltransferase [EC:2.1.3.9] | | |
| K09116 | 3 | hypothetical protein | | |
| K09117 | 10 | hypothetical protein | | |
| K09118 | 29 | hypothetical protein | | |
| K09120 | 2 | hypothetical protein | | |
| K09121 | 25 | hypothetical protein | | |
| K09122 | 9 | hypothetical protein | | |
| K09123 | 12 | hypothetical protein | | |
| K09125 | 6 | hypothetical protein | | |
| K09128 | 3 | hypothetical protein | | |
| K09129 | 5 | hypothetical protein | | |
| K09131 | 12 | hypothetical protein | | |
| K09132 | 1 | hypothetical protein | | |
| K09134 | 98 | hypothetical protein | | |
| K09136 | 1 | hypothetical protein | | |
| K09137 | 13 | hypothetical protein | | |
| K09141 | 10 | hypothetical protein | | |
| K09143 | 3 | hypothetical protein | | |
| K09144 | 1 | hypothetical protein | | |
| K09146 | 2 | hypothetical protein | | |
| K09151 | 9 | hypothetical protein | | |
| K09153 | 4 | hypothetical protein | | |
| K09155 | 3 | hypothetical protein | | |
| K09157 | 3 | hypothetical protein | | |
| K09158 | 2 | hypothetical protein | | |
| K09159 | 2 | hypothetical protein | | |
| K09160 | 3 | hypothetical protein | | |
| K09162 | 15 | hypothetical protein | | |
| K09163 | 2 | hypothetical protein | | |
| K09164 | 7 | hypothetical protein | | |
| K09165 | 6 | hypothetical protein | | |
| K09166 | 8 | hypothetical protein | | |
| K09167 | 1 | hypothetical protein | | |
| K09181 | 129 | hypothetical protein | | |
| K09190 | 1 | hypothetical protein | | |
| K09251 | 10 | putrescine aminotransferase [EC:2.6.1.82] | | |
| K09252 | 3 | feruloyl esterase [EC:3.1.1.73] | | |
| K09384 | 1 | hypothetical protein | | |
| K09386 | 19 | hypothetical protein | | |
| K09456 | 23 | putative acyl-CoA dehydrogenase | | |
| K09457 | 12 | 7-cyano-7-deazaguanine reductase [EC:1.7.1.13] | | |
| K09458 | 82 | 3-oxoacyl-[acyl-carrier-protein] synthase II [EC:2.3.1.179] | | |
| K09459 | 5 | phosphonopyruvate decarboxylase [EC:4.1.1.82] | | |
| K09461 | 29 | anthraniloyl-CoA monooxygenase [EC:1.14.13.40] | | |
| K09471 | 25 | gamma-glutamylputrescine oxidase [EC:1.4.3.-] | | |
| K09472 | 7 | gamma-glutamyl-gamma-aminobutyraldehyde dehydrogenase [EC:1.2.1.-] | | |
| K09474 | 2 | acid phosphatase (class A) [EC:3.1.3.2] | | |
| K09477 | 5 | citrate:succinate antiporter | | |

| | | | | |
|---|---|---|---|---|
| K09516 | 1 | all-trans-retinol 13,14-reductase [EC:1.3.99.23] | | |
| K09607 | 31 | immune inhibitor A [EC:3.4.24.-] | | |
| K09680 | 3 | type II pantothenate kinase [EC:2.7.1.33] | | |
| K09681 | 11 | LysR family transcriptional regulator, transcription activator of | | |
| K09684 | 10 | purine catabolism regulatory protein | | |
| K09686 | 142 | antibiotic transport system permease protein | | |
| K09687 | 115 | antibiotic transport system ATP-binding protein | | |
| K09688 | 3 | capsular polysaccharide transport system permease protein | | |
| K09689 | 1 | capsular polysaccharide transport system ATP-binding protein | | |
| K09690 | 46 | lipopolysaccharide transport system permease protein | | |
| K09691 | 27 | lipopolysaccharide transport system ATP-binding protein | | |
| K09693 | 8 | teichoic acid transport system ATP-binding protein [EC:3.6.3.40] | | |
| K09694 | 20 | lipooligosaccharide transport system permease protein | | |
| K09695 | 6 | lipooligosaccharide transport system ATP-binding protein | | |
| K09697 | 5 | sodium transport system ATP-binding protein | | |
| K09698 | 20 | nondiscriminating glutamyl-tRNA synthetase [EC:6.1.1.24] | | |
| K09699 | 12 | 2-oxoisovalerate dehydrogenase E2 component (dihydrolipoyl | | |
| K09700 | 2 | hypothetical protein | | |
| K09701 | 10 | hypothetical protein | | |
| K09702 | 2 | hypothetical protein | | |
| K09703 | 2 | hypothetical protein | | |
| K09704 | 1 | hypothetical protein | | |
| K09707 | 3 | hypothetical protein | | |
| K09709 | 9 | hypothetical protein | | |
| K09710 | 20 | hypothetical protein | | |
| K09711 | 3 | hypothetical protein | | |
| K09726 | 3 | hypothetical protein | | |
| K09729 | 2 | hypothetical protein | | |
| K09740 | 1 | hypothetical protein | | |
| K09747 | 16 | hypothetical protein | | |
| K09748 | 32 | hypothetical protein | | |
| K09749 | 6 | hypothetical protein | | |
| K09758 | 10 | aspartate 4-decarboxylase [EC:4.1.1.12] | | |
| K09759 | 2 | nondiscriminating aspartyl-tRNA synthetase [EC:6.1.1.23] | | |
| K09760 | 28 | DNA recombination protein RmuC | | |
| K09761 | 46 | ribosomal RNA small subunit methyltransferase E [EC:2.1.1.-] | | |
| K09762 | 5 | hypothetical protein | | |
| K09763 | 4 | hypothetical protein | | |
| K09764 | 6 | hypothetical protein | | |
| K09765 | 1 | hypothetical protein | | |
| K09766 | 3 | hypothetical protein | | |
| K09767 | 15 | hypothetical protein | | |
| K09768 | 8 | hypothetical protein | | |
| K09769 | 27 | hypothetical protein | | |
| K09770 | 9 | hypothetical protein | | |
| K09772 | 3 | cell division inhibitor SepF | | |
| K09773 | 10 | hypothetical protein | | |
| K09774 | 441 | lipopolysaccharide export system protein LptA | | |
| K09775 | 19 | hypothetical protein | | |
| K09776 | 1 | hypothetical protein | | |
| K09777 | 2 | hypothetical protein | | |
| K09778 | 5 | hypothetical protein | | |
| K09779 | 5 | hypothetical protein | | |
| K09780 | 2 | hypothetical protein | | |
| K09781 | 3 | hypothetical protein | | |
| K09786 | 18 | hypothetical protein | | |
| K09787 | 1 | hypothetical protein | | |
| K09788 | 41 | hypothetical protein | | |
| K09790 | 2 | hypothetical protein | | |

| | | | | |
|---|---|---|---|---|
| K09791 | 3 | hypothetical protein | | |
| K09792 | 7 | hypothetical protein | | |
| K09793 | 1 | hypothetical protein | | |
| K09795 | 3 | hypothetical protein | | |
| K09796 | 6 | hypothetical protein | | |
| K09798 | 3 | hypothetical protein | | |
| K09799 | 2 | hypothetical protein | | |
| K09800 | 10 | hypothetical protein | | |
| K09801 | 2 | hypothetical protein | | |
| K09802 | 1 | hypothetical protein | | |
| K09803 | 7 | hypothetical protein | | |
| K09806 | 1 | hypothetical protein | | |
| K09807 | 9 | hypothetical protein | | |
| K09808 | 44 | lipoprotein-releasing system permease protein | | |
| K09809 | 13 | CDP-glycerol glycerophosphotransferase [EC:2.7.8.12] | | |
| K09810 | 59 | lipoprotein-releasing system ATP-binding protein [EC:3.6.3.-] | | |
| K09811 | 32 | cell division transport system permease protein | | |
| K09812 | 13 | cell division transport system ATP-binding protein | | |
| K09815 | 10 | zinc transport system substrate-binding protein | | |
| K09816 | 9 | zinc transport system permease protein | | |
| K09817 | 8 | zinc transport system ATP-binding protein [EC:3.6.3.-] | | |
| K09818 | 5 | manganese/iron transport system substrate-binding protein | | |
| K09819 | 8 | manganese/iron transport system permease protein | | |
| K09820 | 12 | manganese/iron transport system ATP-binding protein | | |
| K09822 | 6 | hypothetical protein | | |
| K09825 | 6 | Fur family transcriptional regulator, peroxide stress response | | |
| K09826 | 4 | Fur family transcriptional regulator, iron response regulator | | |
| K09833 | 1 | homogenitisate phytyltransferase | | |
| K09835 | 2 | carotenoid isomerase [EC:5.-.-.-] | | |
| K09845 | 3 | methoxyneurosporene dehydrogenase [EC:1.14.99.-] | | |
| K09846 | 18 | hydroxyneurosporene methyltransferase [EC:2.1.1.-] | | |
| K09858 | 1 | SEC-C motif domain protein | | |
| K09859 | 1 | hypothetical protein | | |
| K09862 | 6 | hypothetical protein | | |
| K09879 | 1 | isorenieratene synthase | | |
| K09882 | 3 | cobaltochelatase CobS [EC:6.6.1.2] | | |
| K09883 | 6 | cobaltochelatase CobT [EC:6.6.1.2] | | |
| K09888 | 10 | cell division protein ZapA | | |
| K09889 | 3 | hypothetical protein | | |
| K09891 | 1 | hypothetical protein | | |
| K09895 | 4 | hypothetical protein | | |
| K09903 | 23 | uridylate kinase [EC:2.7.4.22] | | |
| K09906 | 1 | hypothetical protein | | |
| K09909 | 2 | hypothetical protein | | |
| K09913 | 1 | hypothetical protein | | |
| K09914 | 4 | putative lipoprotein | | |
| K09915 | 12 | hypothetical protein | | |
| K09916 | 5 | hypothetical protein | | |
| K09917 | 1 | hypothetical protein | | |
| K09918 | 2 | hypothetical protein | | |
| K09919 | 9 | hypothetical protein | | |
| K09921 | 6 | hypothetical protein | | |
| K09922 | 3 | hypothetical protein | | |
| K09923 | 1 | hypothetical protein | | |
| K09924 | 5 | hypothetical protein | | |
| K09926 | 2 | hypothetical protein | | |
| K09927 | 30 | hypothetical protein | | |
| K09928 | 3 | hypothetical protein | | |
| K09930 | 9 | hypothetical protein | | |

| | | | | |
|---|---|---|---|---|
| K09931 | 19 | hypothetical protein | | |
| K09933 | 8 | hypothetical protein | | |
| K09934 | 3 | hypothetical protein | | |
| K09935 | 1 | hypothetical protein | | |
| K09936 | 3 | hypothetical protein | | |
| K09937 | 2 | hypothetical protein | | |
| K09939 | 1 | hypothetical protein | | |
| K09941 | 1 | hypothetical protein | | |
| K09942 | 1 | hypothetical protein | | |
| K09949 | 15 | hypothetical protein | | |
| K09950 | 1 | hypothetical protein | | |
| K09951 | 1 | CRISPR-associated protein Cas2 | | |
| K09952 | 6 | hypothetical protein | | |
| K09953 | 2 | lipid A 3-O-deacylase | | |
| K09955 | 22 | hypothetical protein | | |
| K09958 | 8 | hypothetical protein | | |
| K09959 | 1 | hypothetical protein | | |
| K09962 | 1 | hypothetical protein | | |
| K09966 | 6 | hypothetical protein | | |
| K09967 | 7 | hypothetical protein | | |
| K09969 | 7 | general L-amino acid transport system substrate-binding protein | | |
| K09970 | 2 | general L-amino acid transport system permease protein | | |
| K09971 | 7 | general L-amino acid transport system permease protein | | |
| K09972 | 2 | general L-amino acid transport system ATP-binding protein | | |
| K09973 | 11 | hypothetical protein | | |
| K09974 | 1 | hypothetical protein | | |
| K09978 | 1 | hypothetical protein | | |
| K09979 | 1 | hypothetical protein | | |
| K09981 | 4 | hypothetical protein | | |
| K09983 | 6 | hypothetical protein | | |
| K09984 | 4 | hypothetical protein | | |
| K09986 | 2 | hypothetical protein | | |
| K09988 | 2 | hypothetical protein | | |
| K09989 | 5 | hypothetical protein | | |
| K09990 | 7 | hypothetical protein | | |
| K09992 | 5 | hypothetical protein | | |
| K09994 | 1 | PhnO protein [EC:2.3.1.-] | | |
| K09996 | 9 | arginine transport system substrate-binding protein | | |
| K09999 | 2 | arginine transport system permease protein | | |
| K10001 | 14 | glutamate/aspartate transport system substrate-binding protein | | |
| K10002 | 5 | glutamate/aspartate transport system permease protein | | |
| K10003 | 8 | glutamate/aspartate transport system permease protein | | |
| K10004 | 3 | glutamate/aspartate transport system ATP-binding protein | | |
| K10005 | 1 | glutamate transport system substrate-binding protein | | |
| K10006 | 2 | glutamate transport system permease protein | | |
| K10007 | 2 | glutamate transport system permease protein | | |
| K10009 | 4 | cystine transport system permease protein | | |
| K10010 | 1 | cystine transport system ATP-binding protein [EC:3.6.3.-] | | |
| K10011 | 7 | UDP-GlcUA decarboxylase/UDP-L-Ara4N formyltransferase | | |
| K10012 | 8 | undecaprenyl-phosphate 4-deoxy-4-formamido-L-arabinose transferase | | |
| K10014 | 1 | histidine transport system substrate-binding protein | | |
| K10017 | 1 | histidine transport system ATP-binding protein [EC:3.6.3.21] | | |
| K10026 | 15 | queuosine biosynthesis protein QueE | | |
| K10027 | 11 | phytoene dehydrogenase [EC:1.14.99.-] | | |
| K10036 | 2 | glutamine transport system substrate-binding protein | | |
| K10037 | 3 | glutamine transport system permease protein | | |
| K10039 | 6 | putative glutamine transport system substrate-binding protein | | |
| K10040 | 3 | putative glutamine transport system permease protein | | |
| K10041 | 2 | putative glutamine transport system ATP-binding protein [EC:3.6.3.-] | | |

| | | | | |
|---|---|---|---|---|
| K10108 | 11 | maltose/maltodextrin transport system substrate-binding protein | | |
| K10109 | 5 | maltose/maltodextrin transport system permease protein | | |
| K10110 | 13 | maltose/maltodextrin transport system permease protein | | |
| K10111 | 2 | maltose/maltodextrin transport system ATP-binding protein | | |
| K10112 | 10 | maltose/maltodextrin transport system ATP-binding protein | | |
| K10117 | 1 | multiple sugar transport system substrate-binding protein | | |
| K10118 | 3 | multiple sugar transport system permease protein | | |
| K10119 | 3 | multiple sugar transport system permease protein | | |
| K10120 | 2 | putative sugar transport system substrate-binding protein | | |
| K10121 | 12 | putative sugar transport system permease protein | | |
| K10122 | 6 | putative sugar transport system permease protein | | |
| K10125 | 16 | two-component system, NtrC family, C4-dicarboxylate transport sensor | | |
| K10126 | 65 | two-component system, NtrC family, C4-dicarboxylate transport | | |
| K10188 | 9 | lactose/L-arabinose transport system substrate-binding protein | | |
| K10189 | 8 | lactose/L-arabinose transport system permease protein | | |
| K10190 | 19 | lactose/L-arabinose transport system permease protein | | |
| K10191 | 1 | lactose/L-arabinose transport system ATP-binding protein | | |
| K10193 | 2 | oligogalacturonide transport system permease protein | | |
| K10200 | 3 | N-acetylglucosamine transport system substrate-binding protein | | |
| K10201 | 3 | N-acetylglucosamine transport system permease protein | | |
| K10206 | 25 | LL-diaminopimelate aminotransferase [EC:2.6.1.83] | | |
| K10209 | 1 | dehydrosqualene desaturase [EC:1.14.99.-] | | |
| K10210 | 3 | 4,4'-diaponeurosporene oxidase [EC:1.-.-.-] | | |
| K10215 | 11 | monooxygenase [EC:1.14.13.-] | | |
| K10217 | 4 | aminomuconate-semialdehyde dehydrogenase [EC:1.2.1.32] | | |
| K10218 | 5 | 4-hydroxy-4-methyl-2-oxoglutarate aldolase [EC:4.1.3.17] | | |
| K10219 | 34 | 4-carboxy-2-hydroxymuconate-6-semialdehyde dehydrogenase | | |
| K10220 | 52 | 4-oxalmesaconate hydratase [EC:4.2.1.83] | | |
| K10221 | 17 | 2-pyrone-4,6-dicarboxylate lactonase [EC:3.1.1.57] | | |
| K10227 | 10 | sorbitol/mannitol transport system substrate-binding protein | | |
| K10228 | 1 | sorbitol/mannitol transport system permease protein | | |
| K10229 | 2 | sorbitol/mannitol transport system permease protein | | |
| K10230 | 1 | sorbitol/mannitol transport system ATP-binding protein | | |
| K10231 | 10 | kojibiose phosphorylase [EC:2.4.1.230] | | |
| K10232 | 15 | alpha-glucoside transport system substrate-binding protein | | |
| K10233 | 5 | alpha-glucoside transport system permease protein | | |
| K10234 | 8 | alpha-glucoside transport system permease protein | | |
| K10236 | 6 | trehalose/maltose transport system substrate-binding protein | | |
| K10237 | 4 | trehalose/maltose transport system permease protein | | |
| K10238 | 3 | trehalose/maltose transport system permease protein | | |
| K10239 | 1 | trehalose/maltose transport system ATP-binding protein | | |
| K10240 | 2 | cellobiose transport system substrate-binding protein | | |
| K10241 | 1 | cellobiose transport system permease protein | | |
| K10242 | 1 | cellobiose transport system permease protein | | |
| K10243 | 2 | cellobiose transport system ATP-binding protein | | |
| K10253 | 2 | DOPA 4,5-dioxygenase [EC:1.14.99.-] | | |
| K10254 | 1 | myosin-crossreactive antigen | | |
| K10255 | 15 | omega-6 fatty acid desaturase (delta-12 desaturase) [EC:1.14.19.-] | | |
| K10353 | 5 | deoxyadenosine kinase [EC:2.7.1.76] | | |
| K10439 | 21 | ribose transport system substrate-binding protein | | |
| K10440 | 29 | ribose transport system permease protein | | |
| K10441 | 27 | ribose transport system ATP-binding protein [EC:3.6.3.17] | | |
| K10533 | 3 | limonene-1,2-epoxide hydrolase [EC:3.3.2.8] | | |
| K10536 | 12 | agmatine deiminase [EC:3.5.3.12] | | |
| K10538 | 1 | L-arabinose transport system permease protein | | |
| K10541 | 3 | methyl-galactoside transport system permease protein | | |
| K10542 | 1 | methyl-galactoside transport system ATP-binding protein | | |
| K10543 | 5 | D-xylose transport system substrate-binding protein | | |
| K10544 | 14 | D-xylose transport system permease protein | | |

| | | | | |
|---|---|---|---|---|
| K10545 | 1 | D-xylose transport system ATP-binding protein [EC:3.6.3.17] | | |
| K10547 | 1 | putative multiple sugar transport system permease protein | | |
| K10549 | 2 | D-allose transport system substrate-binding protein | | |
| K10552 | 6 | fructose transport system substrate-binding protein | | |
| K10553 | 2 | fructose transport system permease protein | | |
| K10554 | 2 | fructose transport system ATP-binding protein | | |
| K10555 | 9 | AI-2 transport system substrate-binding protein | | |
| K10556 | 4 | AI-2 transport system permease protein | | |
| K10557 | 2 | AI-2 transport system permease protein | | |
| K10559 | 7 | rhamnose transport system substrate-binding protein | | |
| K10560 | 3 | rhamnose transport system permease protein | | |
| K10561 | 1 | rhamnose transport system permease protein | | |
| K10563 | 62 | formamidopyrimidine-DNA glycosylase [EC:3.2.2.23 4.2.99.18] | | |
| K10616 | 19 | p-cymene monooxygenase [EC:1.14.13.-] | | |
| K10617 | 7 | p-cumic alcohol dehydrogenase | | |
| K10618 | 7 | p-cumic aldehyde dehydrogenase | | |
| K10619 | 17 | p-cumate dioxygenase [EC:1.14.12.-] | | |
| K10620 | 1 | 2,3-dihydroxy-2,3-dihydro-p-cumate dehydrogenase [EC:1.3.1.58] | | |
| K10621 | 5 | 2,3-dihydroxy-p-cumate-3,4-dioxygenase [EC:1.13.11.-] | | |
| K10622 | 2 | HCOMODA decarboxylase [EC:4.1.1.-] | | |
| K10674 | 2 | ectoine hydroxylase [EC:1.14.11.-] | | |
| K10676 | 13 | 2,4-dichlorophenol 6-monooxygenase [EC:1.14.13.20] | | |
| K10678 | 1 | nitroreductase [EC:1.-.-.-] | | |
| K10679 | 1 | nitroreductase / dihydropteridine reductase [EC:1.-.-.- 1.5.1.34] | | |
| K10680 | 11 | N-ethylmaleimide reductase [EC:1.-.-.-] | | |
| K10697 | 6 | two-component system, OmpR family, response regulator RpaA | | |
| K10700 | 13 | ethylbenzene hydroxylase [EC:1.17.99.2] | | |
| K10701 | 20 | acetophenone carboxylase [EC:6.4.1.-] | | |
| K10708 | 1 | fructoselysine 6-phosphate deglycase [EC:3.5.-.-] | | |
| K10711 | 1 | GntR family transcriptional regulator, frlABCD operon | | |
| K10713 | 1 | formaldehyde-activating enzyme [EC:4.3.-.-] | | |
| K10715 | 7 | two-component system, sensor histidine kinase RpfC [EC:2.7.13.3] | | |
| K10716 | 1 | voltage-gated potassium channel | | |
| K10747 | 4 | DNA ligase 1 [EC:6.5.1.1] | | |
| K10754 | 4 | replication factor C subunit 1 | | |
| K10763 | 2 | DnaA-homolog protein | | |
| K10764 | 12 | O-succinylhomoserine sulfhydrylase [EC:2.5.1.-] | | |
| K10773 | 24 | endonuclease III [EC:4.2.99.18] | | |
| K10774 | 6 | tyrosine ammonia-lyase [EC:4.3.1.23] | | |
| K10775 | 1 | phenylalanine ammonia-lyase [EC:4.3.1.24] | | |
| K10778 | 19 | AraC family transcriptional regulator, regulatory protein of | | |
| K10780 | 3 | enoyl-[acyl carrier protein] reductase III [EC:1.3.1.-] | | |
| K10799 | 18 | tankyrase [EC:2.4.2.30] | | |
| K10804 | 10 | acyl-CoA thioesterase I [EC:3.1.2.- 3.1.1.5] | | |
| K10805 | 10 | acyl-CoA thioesterase II [EC:3.1.2.-] | | |
| K10806 | 4 | acyl-CoA thioesterase YciA [EC:3.1.2.-] | | |
| K10807 | 1 | ribonucleoside-diphosphate reductase subunit M1 [EC:1.17.4.1] | | |
| K10810 | 1 | transcriptional regulator TenI | | |
| K10815 | 1 | hydrogen cyanide synthase HcnB [EC:1.4.99.5] | | |
| K10816 | 2 | hydrogen cyanide synthase HcnC [EC:1.4.99.5] | | |
| K10817 | 32 | erythronolide synthase [EC:2.3.1.94] | | |
| K10819 | 143 | histidine kinase | | |
| K10820 | 13 | monosaccharide-transporting ATPase [EC:3.6.3.17] | | |
| K10822 | 21 | nonpolar-amino-acid-transporting ATPase [EC:3.6.3.22] | | |
| K10826 | 34 | Fe3+-transporting ATPase [EC:3.6.3.30] | | |
| K10828 | 1 | quaternary-amine-transporting ATPase [EC:3.6.3.32] | | |
| K10829 | 1 | iron-chelate-transporting ATPase [EC:3.6.3.34] | | |
| K10834 | 7 | heme-transporting ATPase [EC:3.6.3.41] | | |
| K10843 | 12 | DNA excision repair protein ERCC-3 [EC:3.6.1.-] | | |

| | | | | |
|---|---|---|---|---|
| K10844 | 3 | DNA excision repair protein ERCC-2 [EC:3.6.1.-] | | |
| K10855 | 1 | acetone carboxylase, beta subunit [EC:6.4.1.6] | | |
| K10857 | 1 | exodeoxyribonuclease X [EC:3.1.11.-] | | |
| K10907 | 72 | aminotransferase [EC:2.6.1.-] | | |
| K10908 | 1 | DNA-directed RNA polymerase, mitochondrial [EC:2.7.7.6] | | |
| K10912 | 2 | two-component system, repressor protein LuxO | | |
| K10914 | 3 | CRP/FNR family transcriptional regulator, cyclic AMP receptor | | |
| K10915 | 1 | CAI-1 autoinducer synthase [EC:2.3.-.-] | | |
| K10917 | 8 | PadR family transcriptional regulator, regulatory protein AphA | | |
| K10918 | 14 | LysR family transcriptional regulator, transcriptional activator | | |
| K10941 | 18 | sigma-54 specific transcriptional regulator, flagellar regulatory | | |
| K10942 | 29 | two-component system, sensor histidine kinase FlrB [EC:2.7.13.3] | | |
| K10943 | 57 | two component system, response regulator FlrC | | |
| K10947 | 19 | PadR family transcriptional regulator, regulatory protein PadR | | |
| K10972 | 1 | LysR family transcriptional regulator, transcriptional activator of | | |
| K10979 | 36 | DNA end-binding protein Ku | | |
| K11003 | 4 | hemolysin D | | |
| K11004 | 5 | ATP-binding cassette, subfamily B, bacterial HlyB/CyaB | | |
| K11005 | 15 | hemolysin A | | |
| K11016 | 4 | hemolysin | | |
| K11017 | 8 | hemolysin activation/secretion protein?? | | |
| K11029 | 4 | anthrax edema toxin adenylate cyclase [EC:4.6.1.1] | | |
| K11031 | 2 | thiol-activated cytolysin | | |
| K11041 | 2 | exfoliative toxin A/B | | |
| K11049 | 16 | CylG protein | | |
| K11053 | 3 | CylF protein | | |
| K11060 | 5 | probable enterotoxin B | | |
| K11062 | 1 | probable enterotoxin D | | |
| K11065 | 10 | thiol peroxidase, atypical 2-Cys peroxiredoxin [EC:1.11.1.15] | | |
| K11066 | 3 | N-acetylmuramoyl-L-alanine amidase [EC:3.5.1.28] | | |
| K11068 | 27 | hemolysin III | | |
| K11069 | 17 | spermidine/putrescine transport system substrate-binding protein | | |
| K11070 | 19 | spermidine/putrescine transport system permease protein | | |
| K11071 | 27 | spermidine/putrescine transport system permease protein | | |
| K11072 | 19 | spermidine/putrescine transport system ATP-binding protein | | |
| K11073 | 4 | putrescine transport system substrate-binding protein | | |
| K11074 | 10 | putrescine transport system permease protein | | |
| K11075 | 10 | putrescine transport system permease protein | | |
| K11076 | 10 | putrescine transport system ATP-binding protein | | |
| K11079 | 3 | mannopine transport system permease protein | | |
| K11082 | 1 | 2-aminoethylphosphonate transport system permease protein | | |
| K11085 | 118 | ATP-binding cassette, subfamily B, bacterial MsbA [EC:3.6.3.-] | | |
| K11102 | 12 | proton glutamate symport protein | | |
| K11103 | 9 | aerobic C4-dicarboxylate transport protein | | |
| K11105 | 21 | cell volume regulation protein A | | |
| K11106 | 6 | L-tartrate/succinate antiporter | | |
| K11144 | 8 | primosomal protein DnaI | | |
| K11145 | 2 | ribonuclease III family protein | | |
| K11159 | 3 | carotenoid cleavage dioxygenase | | |
| K11173 | 9 | hydroxyacid-oxoacid transhydrogenase [EC:1.1.99.24] | | |
| K11174 | 8 | carbon-monoxide dehydrogenase (ferredoxin) [EC:1.2.7.4] | | |
| K11175 | 32 | phosphoribosylglycinamide formyltransferase 1 [EC:2.1.2.2] | | |
| K11177 | 61 | xanthine dehydrogenase YagR molybdenum-binding subunit [EC:1.17.1.4] | | |
| K11178 | 19 | xanthine dehydrogenase YagS FAD-binding subunit [EC:1.17.1.4] | | |
| K11179 | 14 | tRNA 2-thiouridine synthesizing protein E [EC:2.8.1.-] | | |
| K11180 | 3 | sulfite reductase, dissimilatory-type alpha subunit [EC:1.8.99.3] | | |
| K11181 | 3 | sulfite reductase, dissimilatory-type beta subunit [EC:1.8.99.3] | | |
| K11183 | 16 | phosphocarrier protein FPr | | |
| K11189 | 29 | phosphocarrier protein | | |

| | | | | |
|---|---|---|---|---|
| K11192 | 1 | PTS system, N-acetylmuramic acid-specific IIC component | | |
| K11201 | 2 | PTS system, fructose-specific IIA-like component [EC:2.7.1.69] | | |
| K11209 | 5 | GST-like protein | | |
| K11212 | 20 | LPPG:FO 2-phospho-L-lactate transferase [EC:2.7.8.-] | | |
| K11216 | 1 | autoinducer 2 (AI-2) kinase [EC:2.7.1.-] | | |
| K11249 | 1 | cysteine/O-acetylserine efflux protein | | |
| K11250 | 1 | leucine efflux protein | | |
| K11258 | 2 | acetolactate synthase II small subunit [EC:2.2.1.6] | | |
| K11261 | 6 | formylmethanofuran dehydrogenase subunit E [EC:1.2.99.5] | | |
| K11263 | 26 | acetyl-/propionyl-CoA carboxylase, biotin carboxylase, biotin | | |
| K11264 | 2 | methylmalonyl-CoA decarboxylase [EC:4.1.1.41] | | |
| K11311 | 2 | anthranilate dioxygenase reductase | | |
| K11312 | 6 | cupin 2 domain-containing protein | | |
| K11325 | 6 | L-cysteine/cystine lyase | | |
| K11326 | 7 | cation efflux system protein involved in nickel and cobalt tolerance | | |
| K11329 | 27 | two-component system, OmpR family, response regulator RpaB | | |
| K11333 | 1 | chlorophyllide reductase iron protein subunit X [EC:1.18.6.1] | | |
| K11337 | 3 | 3-hydroxyethyl bacteriochlorophyllide a dehydrogenase [EC:1.-.-.-] | | |
| K11354 | 3 | two-component system, chemotaxis family, sensor kinase Cph1 | | |
| K11355 | 3 | two-component system, chemotaxis family, response regulator Rcp1 | | |
| K11356 | 2 | two-component system, sensor histidine kinase and response regulator | | |
| K11357 | 7 | two-component system, cell cycle sensor histidine kinase DivJ | | |
| K11358 | 4 | aspartate aminotransferase [EC:2.6.1.1] | | |
| K11381 | 62 | 2-oxoisovalerate dehydrogenase E1 component [EC:1.2.4.4] | | |
| K11383 | 3 | two-component system, NtrC family, sensor histidine kinase KinB | | |
| K11384 | 7 | two-component system, NtrC family, response regulator AlgB | | |
| K11418 | 13 | histone deacetylase 11 [EC:3.5.1.98] | | |
| K11443 | 7 | two-component system, cell cycle response regulator DivK | | |
| K11444 | 8 | two-component system, chemotaxis family, response regulator WspR | | |
| K11472 | 15 | glycolate oxidase FAD binding subunit | | |
| K11473 | 36 | glycolate oxidase iron-sulfur subunit | | |
| K11475 | 15 | GntR family transcriptional regulator, vanillate catabolism | | |
| K11476 | 4 | GntR family transcriptional regulator, gluconate operon | | |
| K11477 | 13 | glc operon protein GlcG | | |
| K11523 | 4 | two-component system, chemotaxis family, response regulator PixH | | |
| K11525 | 1 | methyl-accepting chemotaxis protein PixJ | | |
| K11526 | 1 | two-component system, chemotaxis family, sensor histidine kinase and | | |
| K11527 | 58 | two-component system, unclassified family, sensor histidine kinase | | |
| K11528 | 4 | UDP-N-acetylglucosamine pyrophosphorylase [EC:2.7.7.23] | | |
| K11531 | 1 | lsr operon transcriptional repressor | | |
| K11532 | 1 | fructose-1,6-bisphosphatase II / sedoheptulose-1,7-bisphosphatase | | |
| K11533 | 1 | fatty acid synthase, bacteria type [EC:2.3.1.-] | | |
| K11537 | 6 | MFS transporter, NHS family, xanthosine permease | | |
| K11603 | 1 | manganese transport system ATP-binding protein | | |
| K11604 | 1 | manganese/iron transport system substrate-binding protein | | |
| K11606 | 3 | manganese/iron transport system permease protein | | |
| K11608 | 1 | beta-ketoacyl ACP synthase [EC:2.3.1.-] | | |
| K11609 | 1 | beta-ketoacyl ACP synthase [EC:2.3.1.-] | | |
| K11610 | 1 | beta-ketoacyl ACP reductase [EC:1.1.1.100] | | |
| K11611 | 8 | enoyl ACP reductase [EC:1.3.1.9] | | |
| K11615 | 3 | two-component system, CitB family, response regulator MalR | | |
| K11616 | 1 | malate:Na+ symporter | | |
| K11617 | 3 | two-component system, NarL family, sensor histidine kinase LiaS | | |
| K11618 | 39 | two-component system, NarL family, response regulator LiaR | | |
| K11619 | 11 | lia operon protein LiaI | | |
| K11621 | 1 | lia operon protein LiaG | | |
| K11624 | 2 | two-component system, NarL family, response regulator YdfI | | |
| K11625 | 9 | membrane protein YdfJ | | |
| K11634 | 1 | two-component system, OmpR family, response regulator YxdJ | | |

| | | | | |
|---|---|---|---|---|
| K11646 | 1 | dehydroquinate synthase II [EC:1.4.1.-] | | |
| K11688 | 92 | C4-dicarboxylate-binding protein DctP | | |
| K11689 | 12 | C4-dicarboxylate transporter, DctQ subunit | | |
| K11690 | 144 | C4-dicarboxylate transporter, DctM subunit | | |
| K11693 | 2 | peptidoglycan pentaglycine glycine transferase (the first glycine) | | |
| K11710 | 3 | manganese/zinc/iron transport system ATP- binding protein | | |
| K11711 | 16 | two-component system, LuxR family, sensor histidine kinase DctS | | |
| K11712 | 12 | two-component system, LuxR family, response regulator DctR | | |
| K11717 | 72 | cysteine desulfurase / selenocysteine lyase [EC:2.8.1.7 4.4.1.16] | | |
| K11719 | 2 | lipopolysaccharide export system protein LptC | | |
| K11720 | 14 | lipopolysaccharide export system permease protein | | |
| K11731 | 6 | citronellyl-CoA dehydrogenase [EC:1.3.99.-] | | |
| K11740 | 2 | bacteriophage N4 adsorption protein B | | |
| K11741 | 6 | quaternary ammonium compound-resistance protein SugE | | |
| K11744 | 5 | AI-2 transport protein TqsA | | |
| K11745 | 10 | glutathione-regulated potassium-efflux system ancillary protein KefC | | |
| K11746 | 1 | glutathione-regulated potassium-efflux system ancillary protein KefF | | |
| K11747 | 9 | glutathione-regulated potassium-efflux system protein KefB | | |
| K11748 | 1 | glutathione-regulated potassium-efflux system ancillary protein KefG | | |
| K11749 | 54 | regulator of sigma E protease [EC:3.4.24.-] | | |
| K11750 | 11 | esterase FrsA [EC:3.1.-.-] | | |
| K11751 | 4 | 5'-nucleotidase / UDP-sugar diphosphatase [EC:3.1.3.5 3.6.1.45] | | |
| K11752 | 32 | diaminohydroxyphosphoribosylaminopyrimidine deaminase / | | |
| K11753 | 31 | riboflavin kinase / FMN adenylyltransferase [EC:2.7.1.26 2.7.7.2] | | |
| K11754 | 47 | dihydrofolate synthase / folylpolyglutamate synthase [EC:6.3.2.12 | | |
| K11755 | 12 | phosphoribosyl-ATP pyrophosphohydrolase / phosphoribosyl-AMP | | |
| K11777 | 1 | HAD superfamily phosphatase | | |
| K11779 | 20 | FO synthase [EC:2.5.1.-] | | |
| K11781 | 1 | FO synthase subunit 2 [EC:2.5.1.-] | | |
| K11782 | 5 | chorismate futalosine-lyase [EC:4.1.99.-] | | |
| K11784 | 17 | de-hypoxanthine futalosine synthase [EC:1.21.-.-] | | |
| K11785 | 2 | 1,4-dihydroxy-6-naphthoate synthase [EC:1.14.-.-] | | |
| K11890 | 5 | type VI secretion system protein ImpM | | |
| K11891 | 8 | type VI secretion system protein ImpL | | |
| K11892 | 8 | type VI secretion system protein ImpK | | |
| K11893 | 9 | type VI secretion system protein ImpJ | | |
| K11894 | 3 | type VI secretion system protein ImpI | | |
| K11895 | 4 | type VI secretion system protein ImpH | | |
| K11896 | 7 | type VI secretion system protein ImpG | | |
| K11897 | 2 | type VI secretion system protein ImpF | | |
| K11898 | 1 | type VI secretion system protein ImpE | | |
| K11899 | 2 | type VI secretion system protein ImpD | | |
| K11900 | 10 | type VI secretion system protein ImpC | | |
| K11901 | 8 | type VI secretion system protein ImpB | | |
| K11902 | 6 | type VI secretion system protein ImpA | | |
| K11903 | 1 | type VI secretion system secreted protein Hcp | | |
| K11904 | 9 | type VI secretion system secreted protein VgrG | | |
| K11905 | 1 | type VI secretion system protein | | |
| K11906 | 7 | type VI secretion system protein VasD | | |
| K11907 | 13 | type VI secretion system protein VasG | | |
| K11910 | 2 | type VI secretion system protein VasJ | | |
| K11911 | 1 | type VI secretion system protein VasL | | |
| K11912 | 28 | serine/threonine-protein kinase PpkA [EC:2.7.11.1] | | |
| K11913 | 3 | type VI secretion system protein | | |
| K11914 | 2 | sigma-54 dependent transcriptional regulator | | |
| K11916 | 1 | serine/threonine-protein kinase Stk1 [EC:2.7.11.-] | | |
| K11917 | 3 | sigma-54 specific transcriptional regulator | | |
| K11921 | 10 | LysR family transcriptional regulator | | |
| K11922 | 1 | GntR family transcriptional regulator, mannosyl-D-glycerate | | |

| | | | | |
|---|---|---|---|---|
| K11923 | 3 | MerR family transcriptional regulator, copper efflux regulator | | |
| K11924 | 4 | DtxR family transcriptional regulator, manganese transport regulator | | |
| K11925 | 1 | SgrR family transcriptional regulator | | |
| K11926 | 1 | sigma factor-binding protein Crl | | |
| K11927 | 27 | ATP-dependent RNA helicase RhlE [EC:3.6.4.13] | | |
| K11928 | 6 | sodium/proline symporter | | |
| K11930 | 1 | periplasmic protein TorT | | |
| K11931 | 5 | biofilm PGA synthesis lipoprotein PgaB [EC:3.-.-.-] | | |
| K11933 | 4 | NADH oxidoreductase Hcr [EC:1.-.-.-] | | |
| K11936 | 26 | biofilm PGA synthesis N-glycosyltransferase PgaC [EC:2.4.-.-] | | |
| K11938 | 1 | HMP-PP phosphatase [EC:3.6.1.-] | | |
| K11939 | 8 | inner membrane transporter RhtA | | |
| K11940 | 2 | heat shock protein HspQ | | |
| K11942 | 14 | methylmalonyl-CoA mutase [EC:5.4.99.2] | | |
| K11948 | 2 | 1-hydroxy-2-naphthoate dioxygenase [EC:1.13.11.38] | | |
| K11949 | 1 | 4-(2-carboxyphenyl)-2-oxobut-3-enoate aldolase [EC:4.1.2.34] | | |
| K11953 | 1 | bicarbonate transport system ATP-binding protein [EC:3.6.3.-] | | |
| K11954 | 2 | neutral amino acid transport system substrate-binding protein | | |
| K11955 | 1 | neutral amino acid transport system permease protein | | |
| K11956 | 1 | neutral amino acid transport system permease protein | | |
| K11957 | 2 | neutral amino acid transport system ATP-binding protein | | |
| K11958 | 2 | neutral amino acid transport system ATP-binding protein | | |
| K11959 | 4 | urea transport system substrate-binding protein | | |
| K11960 | 1 | urea transport system permease protein | | |
| K11961 | 2 | urea transport system permease protein | | |
| K11963 | 1 | urea transport system ATP-binding protein | | |
| K11987 | 1 | prostaglandin-endoperoxide synthase 2 [EC:1.14.99.1] | | |
| K11991 | 12 | tRNA-specific adenosine deaminase [EC:3.5.4.-] | | |
| K12059 | 1 | conjugal transfer pilus assembly protein TrbC | | |
| K12063 | 2 | conjugal transfer ATP-binding protein TraC | | |
| K12065 | 1 | conjugal transfer pilus assembly protein TraB | | |
| K12073 | 3 | 1,4-dihydroxy-2-naphthoyl-CoA thioesterase [EC:3.1.2.-] | | |
| K12111 | 2 | evolved beta-galactosidase subunit alpha [EC:3.2.1.23] | | |
| K12137 | 9 | hydrogenase-4 component B [EC:1.-.-.-] | | |
| K12138 | 10 | hydrogenase-4 component C [EC:1.-.-.-] | | |
| K12139 | 6 | hydrogenase-4 component D [EC:1.-.-.-] | | |
| K12140 | 6 | hydrogenase-4 component E [EC:1.-.-.-] | | |
| K12141 | 14 | hydrogenase-4 component F [EC:1.-.-.-] | | |
| K12142 | 1 | hydrogenase-4 component G [EC:1.-.-.-] | | |
| K12143 | 4 | hydrogenase-4 component H | | |
| K12146 | 16 | hydrogenase-4 transcriptional activator | | |
| K12148 | 3 | biofilm regulator BssS | | |
| K12150 | 1 | HIT-like protein HinT | | |
| K12152 | 3 | phosphatase NudJ [EC:3.6.1.-] | | |
| K12203 | 34 | defect in organelle trafficking protein DotB | | |
| K12206 | 6 | intracellular multiplication protein IcmB | | |
| K12209 | 3 | intracellular multiplication protein IcmE | | |
| K12210 | 6 | intracellular multiplication protein IcmF | | |
| K12213 | 10 | intracellular multiplication protein IcmK | | |
| K12214 | 5 | intracellular multiplication protein IcmL | | |
| K12216 | 1 | intracellular multiplication protein IcmN | | |
| K12234 | 9 | F420-0:gamma-glutamyl ligase [EC:6.3.2.-] | | |
| K12238 | 5 | pyochelin biosynthesis protein PchD | | |
| K12240 | 1 | pyochelin synthetase | | |
| K12251 | 28 | N-carbamoylputrescine amidase [EC:3.5.1.53] | | |
| K12252 | 2 | arginine:pyruvate transaminase [EC:2.6.1.84] | | |
| K12253 | 4 | 5-guanidino-2-oxopentanoate decarboxylase [EC:4.1.1.75] | | |
| K12254 | 3 | 4-guanidinobutyraldehyde dehydrogenase / NAD-dependent aldehyde | | |
| K12255 | 4 | guanidinobutyrase [EC:3.5.3.7] | | |

| | | | | |
|---|---|---|---|---|
| K12256 | 7 | putrescine aminotransferase [EC:2.6.1.-] | | |
| K12257 | 30 | SecD/SecF fusion protein | | |
| K12262 | 5 | cytochrome b561 | | |
| K12264 | 2 | anaerobic nitric oxide reductase flavorubredoxin | | |
| K12265 | 1 | nitric oxide reductase FlRd-NAD(+) reductase [EC:1.18.1.-] | | |
| K12266 | 6 | anaerobic nitric oxide reductase transcription regulator | | |
| K12267 | 22 | peptide methionine sulfoxide reductase msrA/msrB [EC:1.8.4.11 | | |
| K12276 | 10 | MSHA biogenesis protein MshE | | |
| K12278 | 8 | MSHA biogenesis protein MshG | | |
| K12282 | 9 | MSHA biogenesis protein MshL | | |
| K12283 | 7 | MSHA biogenesis protein MshM | | |
| K12287 | 4 | MSHA biogenesis protein MshQ | | |
| K12297 | 4 | ribosomal RNA large subunit methyltransferase L [EC:2.1.1.52] | | |
| K12299 | 1 | MFS transporter, ACS family, probable galactarate transporter | | |
| K12308 | 35 | beta-galactosidase [EC:3.2.1.23] | | |
| K12339 | 16 | cysteine synthase B [EC:2.5.1.47] | | |
| K12340 | 36 | outer membrane channel protein TolC | | |
| K12343 | 4 | 3-oxo-5-alpha-steroid 4-dehydrogenase 1 [EC:1.3.99.5] | | |
| K12344 | 4 | 3-oxo-5-alpha-steroid 4-dehydrogenase 2 [EC:1.3.99.5] | | |
| K12368 | 17 | dipeptide transport system substrate-binding protein | | |
| K12369 | 12 | dipeptide transport system permease protein | | |
| K12370 | 10 | dipeptide transport system permease protein | | |
| K12371 | 6 | dipeptide transport system ATP-binding protein | | |
| K12372 | 1 | dipeptide transport system ATP-binding protein | | |
| K12373 | 31 | beta-hexosaminidase [EC:3.2.1.52] | | |
| K12410 | 28 | NAD-dependent deacetylase [EC:3.5.1.-] | | |
| K12420 | 16 | ketoacyl reductase [EC:1.3.1.-] | | |
| K12421 | 1 | fatty acid CoA ligase FadD9 | | |
| K12423 | 2 | fatty acid CoA ligase FadD21 | | |
| K12424 | 2 | fatty acid CoA ligase FadD22 | | |
| K12427 | 1 | fatty acid CoA ligase FadD28 | | |
| K12428 | 1 | fatty acid CoA ligase FadD32 | | |
| K12429 | 5 | fatty acid CoA ligase FadD36 | | |
| K12430 | 3 | polyketide synthase 1/15 | | |
| K12432 | 1 | polyketide synthase 3/4 | | |
| K12436 | 22 | polyketide synthase 12 | | |
| K12437 | 1 | polyketide synthase 13 | | |
| K12441 | 1 | phenolpthiocerol synthesis type-I polyketide synthase B | | |
| K12442 | 1 | phenolpthiocerol synthesis type-I polyketide synthase C | | |
| K12444 | 3 | phenolpthiocerol synthesis type-I polyketide synthase E | | |
| K12445 | 2 | trans enoyl reductase [EC:1.3.1.-] | | |
| K12452 | 6 | CDP-6-deoxy-D-xylo-4-hexulose-3-dehydrase | | |
| K12454 | 22 | CDP-paratose 2-epimerase [EC:5.1.3.10] | | |
| K12500 | 1 | thioesterase III [EC:3.1.2.-] | | |
| K12503 | 5 | short-chain Z-isoprenyl diphosphate synthase [EC:2.5.1.68] | | |
| K12506 | 29 | 2-C-methyl-D-erythritol 4-phosphate cytidylyltransferase / | | |
| K12507 | 2 | acyl-CoA synthetase [EC:6.2.1.-] | | |
| K12508 | 42 | feruloyl-CoA synthase [EC:6.2.1.34] | | |
| K12510 | 17 | tight adherence protein B | | |
| K12511 | 16 | tight adherence protein C | | |
| K12524 | 14 | bifunctional aspartokinase/homoserine dehydrogenase 1 [EC:2.7.2.4 | | |
| K12525 | 1 | bifunctional aspartokinase/homoserine dehydrogenase 2 [EC:2.7.2.4 | | |
| K12526 | 1 | bifunctional diaminopimelate decarboxylase/aspartate kinase | | |
| K12527 | 2 | putative selenate reductase [EC:1.97.1.9] | | |
| K12528 | 2 | putative selenate reductase molybdopterin-binding subunit | | |
| K12536 | 4 | ATP-binding cassette, subfamily C, bacterial HasD | | |
| K12537 | 2 | protease secretion protein HasE | | |
| K12538 | 1 | outer membrane protein HasF | | |
| K12541 | 3 | ATP-binding cassette, subfamily C, bacterial LapB | | |

| | | | | |
|---|---|---|---|---|
| K12542 | 1 | membrane fusion protein LapC | | |
| K12543 | 1 | outer membrane protein LapE | | |
| K12544 | 1 | S-layer protein | | |
| K12549 | 12 | surface adhesion protein | | |
| K12555 | 1 | penicillin-binding protein 2A [EC:2.4.1.129 2.3.2.-] | | |
| K12573 | 53 | ribonuclease R [EC:3.1.-.-] | | |
| K12574 | 39 | ribonuclease J [EC:3.1.-.-] | | |
| K12583 | 6 | phosphatidylinositol alpha 1,6-mannosyltransferase [EC:2.4.1.-] | | |
| K12645 | 13 | epi-isozizaene 5-monooxygenase [EC:1.14.13.106] | | |
| K12658 | 5 | 4-hydroxyproline epimerase [EC:5.1.1.8] | | |
| K12660 | 5 | 2-dehydro-3-deoxy-L-rhamnonate aldolase [EC:4.1.2.-] | | |
| K12661 | 1 | L-rhamnonate dehydratase [EC:4.2.1.90] | | |
| K12673 | 2 | N2-(2-carboxyethyl)arginine synthase [EC:2.5.1.66] | | |
| K12686 | 2 | outer membrane lipase/esterase | | |
| K12688 | 2 | autotransporter serine protease [EC:3.4.21.-] | | |
| K12941 | 17 | aminobenzoyl-glutamate utilization protein B | | |
| K12942 | 5 | aminobenzoyl-glutamate transport protein | | |
| K12943 | 2 | lipoprotein YgeR | | |
| K12949 | 13 | cation-transporting P-type ATPase A/B [EC:3.6.3.-] | | |
| K12950 | 1 | cation-transporting P-type ATPase C [EC:3.6.3.-] | | |
| K12952 | 7 | cation-transporting ATPase E [EC:3.6.3.-] | | |
| K12953 | 6 | cation-transporting ATPase F [EC:3.6.3.-] | | |
| K12956 | 10 | cation-transporting ATPase V [EC:3.6.3.-] | | |
| K12960 | 17 | 5-methylthioadenosine/S-adenosylhomocysteine deaminase | | |
| K12961 | 2 | DnaA initiator-associating protein | | |
| K12972 | 5 | gyoxylate/hydroxypyruvate reductase A [EC:1.1.1.79 1.1.1.81] | | |
| K12979 | 2 | beta-hydroxylase [EC:1.14.11.-] | | |
| K12982 | 2 | heptosyltransferase I [EC:2.4.-.-] | | |
| K12984 | 12 | (heptosyl)LPS beta-1,4-glucosyltransferase [EC:2.4.1.-] | | |
| K12987 | 3 | alpha-1,6-rhamnosyltransferase [EC:2.4.1.-] | | |
| K12988 | 2 | alpha-1,3-rhamnosyltransferase [EC:2.4.1.-] | | |
| K12989 | 2 | mannosyltransferase [EC:2.4.1.-] | | |
| K12990 | 3 | rhamnosyltransferase [EC:2.4.1.-] | | |
| K12992 | 1 | rhamnosyltransferase [EC:2.4.1.-] | | |
| K12994 | 6 | alpha-1,3-rhamnosyltransferase [EC:2.4.1.-] | | |
| K12995 | 3 | rhamnosyltransferase [EC:2.4.1.-] | | |
| K12996 | 4 | rhamnosyltransferase [EC:2.4.1.-] | | |
| K12997 | 1 | rhamnosyltransferase [EC:2.4.1.-] | | |
| K13001 | 4 | mannosyltransferase [EC:2.4.1.-] | | |
| K13002 | 3 | glycosyltransferase [EC:2.4.1.-] | | |
| K13004 | 5 | galacturonosyltransferase [EC:2.4.1.-] | | |
| K13006 | 4 | UDP-perosamine 4-acetyltransferase [EC:2.3.1.-] | | |
| K13007 | 1 | Fuc2NAc and GlcNAc transferase [EC:2.4.1.-] | | |
| K13010 | 16 | perosamine synthetase | | |
| K13013 | 17 | O-antigen biosynthesis protein WbqV | | |
| K13014 | 2 | undecaprenyl phosphate-alpha-L-ara4FN deformylase [EC:3.5.-.-] | | |
| K13015 | 28 | UDP-N-acetyl-D-glucosamine dehydrogenase [EC:1.1.1.-] | | |
| K13016 | 1 | UDP-D-GlcNAcA oxidase [EC:1.1.1.-] | | |
| K13017 | 16 | UDP-3-keto-D-GlcNAcA aminotransferase [EC:2.6.1.-] | | |
| K13018 | 4 | UDP-D-GlcNAc3NA acetyltransferase [EC:2.3.1.-] | | |
| K13019 | 9 | UDP-GlcNAc3NAcA epimerase [EC:5.1.3.23] | | |
| K13020 | 21 | UDP-D-GlcNAcA oxidase [EC:1.1.1.-] | | |
| K13021 | 6 | MFS transporter, ACS family, tartrate transporter | | |
| K13038 | 42 | phosphopantothenoylcysteine decarboxylase / | | |
| K13039 | 5 | sulfopyruvate decarboxylase subunit beta [EC:4.1.1.79] | | |
| K13041 | 1 | two-component system, LuxR family, response regulator TtrR | | |
| K13042 | 2 | dimethylglycine N-methyltransferase [EC:2.1.1.161] | | |
| K13043 | 3 | N-succinyl-L-ornithine transcarbamylase [EC:2.1.3.11] | | |
| K13049 | 48 | carboxypeptidase PM20D1 [EC:3.4.17.-] | | |

| | | | | |
|---|---|---|---|---|
| K13051 | 19 | beta-aspartyl-peptidase (threonine type) [EC:3.4.19.5] | | |
| K13052 | 1 | cell division protein DivIC | | |
| K13057 | 14 | trehalose synthase [EC:2.4.1.245] | | |
| K13058 | 1 | mannosylfructose-phosphate synthase [EC:2.4.1.246] | | |
| K13063 | 1 | phenazine biosynthesis protein phzE [EC:2.6.1.86] | | |
| K13069 | 3 | diguanylate cyclase [EC:2.7.7.65] | | |
| K13074 | 8 | biflaviolin synthase [EC:1.14.21.7] | | |
| K13075 | 16 | N-acyl homoserine lactone hydrolase [EC:3.1.1.81] | | |
| K13237 | 3 | peroxisomal 2,4-dienoyl-CoA reductase [EC:1.3.1.34] | | |
| K13243 | 2 | c-di-GMP-specific phosphodiesterase [EC:3.1.4.52] | | |
| K13244 | 1 | c-di-GMP-specific phosphodiesterase [EC:3.1.4.52] | | |
| K13245 | 7 | c-di-GMP-specific phosphodiesterase [EC:3.1.4.52] | | |
| K13252 | 1 | putrescine carbamoyltransferase [EC:2.1.3.6] | | |
| K13256 | 3 | protein PsiE | | |
| K13274 | 5 | cell wall-associated protease [EC:3.4.21.-] | | |
| K13275 | 2 | major intracellular serine protease [EC:3.4.21.-] | | |
| K13276 | 3 | bacillopeptidase F [EC:3.4.21.-] | | |
| K13277 | 3 | minor extracellular protease Epr [EC:3.4.21.-] | | |
| K13280 | 2 | signal peptidase, endoplasmic reticulum-type [EC:3.4.-.-] | | |
| K13281 | 2 | UV DNA damage endonuclease [EC:3.-.-.-] | | |
| K13282 | 2 | cyanophycinase [EC:3.4.15.6] | | |
| K13283 | 57 | ferrous-iron efflux pump FieF | | |
| K13285 | 1 | invasin B | | |
| K13288 | 10 | oligoribonuclease [EC:3.1.-.-] | | |
| K13292 | 27 | phosphatidylglycerol:prolipoprotein diacylglycerol transferase | | |
| K13317 | 3 | NDP-4-keto-2,6-dideoxyhexose 3-C-methyltransferase | | |
| K13318 | 6 | dTDP-4-keto-6-deoxy-L-hexose 4-reductase | | |
| K13378 | 29 | NADH dehydrogenase I subunit C/D [EC:1.6.5.3] | | |
| K13380 | 1 | NADH dehydrogenase I subunit B/C/D [EC:1.6.5.3] | | |
| K13408 | 2 | membrane fusion protein RaxA | | |
| K13409 | 1 | ATP-binding cassette, subfamily B, bacterial RaxB | | |
| K13419 | 13 | serine/threonine-protein kinase PknK [EC:2.7.11.1] | | |
| K13421 | 1 | uridine monophosphate synthetase [EC:2.4.2.10 4.1.1.23] | | |
| K13479 | 1 | xanthine dehydrogenase FAD-binding subunit [EC:1.17.1.4] | | |
| K13481 | 2 | xanthine dehydrogenase small subunit [EC:1.17.1.4] | | |
| K13482 | 10 | xanthine dehydrogenase large subunit [EC:1.17.1.4] | | |
| K13483 | 14 | xanthine dehydrogenase YagT iron-sulfur-binding subunit | | |
| K13486 | 2 | chemotaxis protein methyltransferase WspC | | |
| K13490 | 5 | two-component system, chemotaxis family, sensor histidine kinase and | | |
| K13491 | 1 | two-component system, chemotaxis family, response regulator WspF | | |
| K13497 | 7 | anthranilate synthase/phosphoribosyltransferase [EC:4.1.3.27 | | |
| K13498 | 7 | indole-3-glycerol phosphate synthase / phosphoribosylanthranilate | | |
| K13500 | 1 | chondroitin synthase [EC:2.4.1.175 2.4.1.226] | | |
| K13503 | 3 | anthranilate synthase [EC:4.1.3.27] | | |
| K13522 | 2 | bifunctional NMN adenylyltransferase/nudix hydrolase [EC:2.7.7.1 | | |
| K13525 | 23 | transitional endoplasmic reticulum ATPase | | |
| K13527 | 10 | proteasome-associated ATPase | | |
| K13529 | 16 | AraC family transcriptional regulator, regulatory protein of | | |
| K13530 | 4 | AraC family transcriptional regulator, regulatory protein of | | |
| K13531 | 1 | methylated-DNA-[protein]-cysteine S-methyltransferase [EC:2.1.1.63] | | |
| K13532 | 2 | two-component system, sporulation sensor kinase D [EC:2.7.13.3] | | |
| K13533 | 7 | two-component system, sporulation sensor kinase E [EC:2.7.13.3] | | |
| K13540 | 2 | precorrin-2 C20-methyltransferase / precorrin-3B | | |
| K13541 | 3 | cobalamin biosynthesis protein CbiG / precorrin-3B | | |
| K13542 | 25 | uroporphyrinogen III methyltransferase / synthase [EC:2.1.1.107 | | |
| K13543 | 6 | uroporphyrinogen III methyltransferase / synthase [EC:2.1.1.107 | | |
| K13571 | 4 | proteasome accessory factor A [EC:6.3.2.-] | | |
| K13572 | 7 | proteasome accessory factor B | | |
| K13573 | 11 | proteasome accessory factor C | | |

| | | | | |
|---|---|---|---|---|
| K13574 | 10 | uncharacterized oxidoreductase [EC:1.1.1.-] | | |
| K13581 | 4 | modification methylase [EC:2.1.1.72] | | |
| K13582 | 2 | localization factor PodJL | | |
| K13583 | 3 | GcrA cell cycle regulator | | |
| K13584 | 1 | two-component system, cell cycle response regulator CtrA | | |
| K13587 | 41 | two-component system, cell cycle sensor histidine kinase and | | |
| K13588 | 1 | histidine phosphotransferase ChpT | | |
| K13589 | 2 | two-component system, cell cycle response regulator CpdR | | |
| K13590 | 30 | diguanylate cyclase | | |
| K13592 | 1 | regulator of CtrA degradation | | |
| K13593 | 1 | cyclic-di-GMP phosphodiesterase, flagellum assembly factor TipF | | |
| K13598 | 35 | two-component system, NtrC family, nitrogen regulation sensor | | |
| K13599 | 57 | two-component system, NtrC family, nitrogen regulation response | | |
| K13601 | 26 | bacteriochlorophyll C8 methyltransferase [EC:2.1.1.-] | | |
| K13602 | 20 | bacteriochlorophyll C12 methyltransferase [EC:2.1.1.-] | | |
| K13604 | 7 | bacteriochlorophyll C20 methyltransferase [EC:2.1.1.-] | | |
| K13607 | 4 | cinnamoyl-CoA:phenyllactate CoA-transferase [EC:2.8.3.17] | | |
| K13611 | 6 | polyketide synthase PksJ | | |
| K13612 | 1 | polyketide synthase PksL | | |
| K13614 | 6 | polyketide synthase PksN | | |
| K13628 | 22 | iron-sulfur cluster assembly protein | | |
| K13633 | 20 | AraC family transcriptional regulator, transcriptional activator | | |
| K13634 | 7 | LysR family transcriptional regulator, cys regulon transcriptional | | |
| K13635 | 7 | LysR family transcriptional regulator, cys regulon transcriptional | | |
| K13637 | 1 | GntR family transcriptional regulator, uxuAB operon transcriptional | | |
| K13638 | 4 | MerR family transcriptional regulator, Zn(II)-responsive regulator | | |
| K13639 | 5 | MerR family transcriptional regulator, redox-sensitive | | |
| K13640 | 10 | MerR family transcriptional regulator, heat shock protein HspR | | |
| K13641 | 40 | IclR family transcriptional regulator, acetate operon repressor | | |
| K13642 | 7 | CRP/FNR family transcriptional regulator, transcriptional activator | | |
| K13643 | 24 | Rrf2 family transcriptional regulator, iron-sulfur cluster assembly | | |
| K13652 | 4 | AraC family transcriptional regulator | | |
| K13653 | 3 | AraC family transcriptional regulator | | |
| K13654 | 5 | GntR family transcriptional regulator, colanic acid and biofilm gene | | |
| K13660 | 1 | beta-1,4-glucosyltransferase [EC:2.4.1.-] | | |
| K13661 | 9 | GumC protein | | |
| K13668 | 21 | phosphatidylinositol alpha-1,6-mannosyltransferase [EC:2.4.1.-] | | |
| K13670 | 1 | alpha-1,6-mannosyltransferase [EC:2.4.1.-] | | |
| K13678 | 1 | monoglucosyldiacylglycerol glycosyltransferase [EC:2.4.1.-] | | |
| K13683 | 1 | putative colanic acid biosynthesis glycosyltransferase [EC:2.4.-.-] | | |
| K13684 | 1 | putative colanic acid biosynthesis glycosyltransferase [EC:2.4.-.-] | | |
| K13685 | 27 | UDP-N-acetylglucosamine:undecaprenyl-P N-acetylglucosaminyl 1-P | | |
| K13688 | 38 | cyclic beta-1,2-glucan synthetase [EC:2.4.1.-] | | |
| K13693 | 8 | glucosyl-3-phosphoglycerate synthase [EC:2.4.1.-] | | |
| K13694 | 3 | lipoprotein Spr | | |
| K13695 | 3 | probable lipoprotein NlpC | | |
| K13730 | 107 | internalin A | | |
| K13735 | 100 | adhesin/invasin | | |
| K13741 | 1 | guanine nucleotide exchange factor SopE | | |
| K13742 | 1 | protein IpgB1 | | |
| K13745 | 5 | L-2,4-diaminobutyrate decarboxylase [EC:4.1.1.86] | | |
| K13746 | 1 | carboxynorspermidine dehydrogenase | | |
| K13747 | 2 | carboxynorspermidine decarboxylase [EC:4.1.1.-] | | |
| K13766 | 13 | methylglutaconyl-CoA hydratase [EC:4.2.1.18] | | |
| K13767 | 5 | enoyl-CoA hydratase [EC:4.2.1.17] | | |
| K13770 | 16 | TetR/AcrR family transcriptional regulator, fatty acid metabolism | | |
| K13771 | 2 | Rrf2 family transcriptional regulator, nitric oxide-sensitive | | |
| K13774 | 3 | citronellol/citronellal dehydrogenase | | |
| K13775 | 5 | citronellol/citronellal dehydrogenase | | |

| | | | | |
|---|---|---|---|---|
| K13776 | 1 | citronellyl-CoA synthetase [EC:6.2.1.-] | | |
| K13777 | 1 | geranyl-CoA carboxylase alpha subunit [EC:6.4.1.5] | | |
| K13778 | 4 | geranyl-CoA carboxylase beta subunit [EC:6.4.1.5] | | |
| K13787 | 9 | geranylgeranyl diphosphate synthase, type I [EC:2.5.1.1 2.5.1.10 | | |
| K13788 | 5 | phosphate acetyltransferase [EC:2.3.1.8] | | |
| K13789 | 15 | geranylgeranyl diphosphate synthase, type II [EC:2.5.1.1 2.5.1.10 | | |
| K13794 | 1 | LysR family transcriptional regulator, regulatory protein for tcuABC | | |
| K13795 | 4 | citrate/tricarballylate utilization protein | | |
| K13796 | 11 | tricarballylate dehydrogenase | | |
| K13797 | 2 | DNA-directed RNA polymerase subunit beta-beta' [EC:2.7.7.6] | | |
| K13799 | 8 | pantoate ligase / cytidylate kinase [EC:6.3.2.1 2.7.4.14] | | |
| K13810 | 14 | transaldolase / glucose-6-phosphate isomerase [EC:2.2.1.2 5.3.1.9] | | |
| K13811 | 3 | 3'-phosphoadenosine 5'-phosphosulfate synthase [EC:2.7.7.4 2.7.1.25] | | |
| K13815 | 21 | two-component system, response regulator RpfG | | |
| K13816 | 7 | DSF synthase | | |
| K13819 | 3 | NifU-like protein | | |
| K13820 | 1 | flagellar biosynthetic protein FliR/FlhB | | |
| K13821 | 28 | proline dehydrogenase / delta 1-pyrroline-5-carboxylate | | |
| K13829 | 16 | shikimate kinase / 3-dehydroquinate synthase [EC:2.7.1.71 4.2.3.4] | | |
| K13832 | 9 | 3-dehydroquinate dehydratase / shikimate dehydrogenase [EC:4.2.1.10 | | |
| K13874 | 3 | L-arabinonolactonase [EC:3.1.1.15] | | |
| K13875 | 7 | L-arabonate dehydrase [EC:4.2.1.25] | | |
| K13876 | 5 | 2-keto-3-deoxy-L-arabinonate dehydratase [EC:4.2.1.43] | | |
| K13888 | 53 | macrolide-specific efflux protein MacA | | |
| K13889 | 13 | glutathione transport system substrate-binding protein | | |
| K13890 | 6 | glutathione transport system permease protein | | |
| K13891 | 2 | glutathione transport system permease protein | | |
| K13892 | 4 | glutathione transport system ATP-binding protein | | |
| K13893 | 9 | microcin C transport system substrate-binding protein | | |
| K13894 | 2 | microcin C transport system permease protein | | |
| K13895 | 4 | microcin C transport system permease protein | | |
| K13896 | 3 | microcin C transport system ATP-binding protein | | |
| K13919 | 1 | propanediol dehydratase medium subunit [EC:4.2.1.28] | | |
| K13922 | 1 | propionaldehyde dehydrogenase | | |
| K13924 | 124 | two-component system, chemotaxis family, CheB/CheR fusion protein | | |
| K13926 | 4 | ribosome-dependent ATPase | | |
| K13940 | 13 | dihydroneopterin aldolase / | | |
| K13950 | 9 | para-aminobenzoate synthetase [EC:2.6.1.85] | | |
| K13953 | 52 | alcohol dehydrogenase, propanol-preferring [EC:1.1.1.1] | | |
| K13954 | 5 | alcohol dehydrogenase [EC:1.1.1.1] | | |
| K13955 | 7 | zinc-binding alcohol dehydrogenase/oxidoreductase | | |
| K13963 | 11 | serpin B | | |
| K13979 | 9 | uncharacterized zinc-type alcohol dehydrogenase-like protein | | |
| K13990 | 19 | glutamate formiminotransferase / formiminotetrahydrofolate | | |
| K13993 | 58 | HSP20 family protein | | |
| K13995 | 2 | maleamate amidohydrolase [EC:3.5.1.107] | | |

| KEGG ID | Sequences | KEGG pathway | KEGG category | |
|---|---|---|---|---|
| K00001 | 181 | alcohol dehydrogenase [EC:1.1.1.1] | Metabolism | |
| K00002 | 3 | alcohol dehydrogenase (NADP+) [EC:1.1.1.2] | Metabolism | |
| K00003 | 74 | homoserine dehydrogenase [EC:1.1.1.3] | Metabolism | |
| K00004 | 3 | (R,R)-butanediol dehydrogenase / diacetyl reductase [EC:1.1.1.4 | Metabolism | |
| K00005 | 10 | glycerol dehydrogenase [EC:1.1.1.6] | Metabolism | |
| K00008 | 37 | L-iditol 2-dehydrogenase [EC:1.1.1.14] | Metabolism | |
| K00009 | 4 | mannitol-1-phosphate 5-dehydrogenase [EC:1.1.1.17] | Metabolism | |
| K00010 | 96 | myo-inositol 2-dehydrogenase [EC:1.1.1.18] | Metabolism | |
| K00011 | 4 | aldehyde reductase [EC:1.1.1.21] | Metabolism | |
| K00012 | 45 | UDPglucose 6-dehydrogenase [EC:1.1.1.22] | Metabolism | |
| K00013 | 43 | histidinol dehydrogenase [EC:1.1.1.23] | Metabolism | |
| K00014 | 37 | shikimate dehydrogenase [EC:1.1.1.25] | Metabolism | |
| K00015 | 39 | glyoxylate reductase [EC:1.1.1.26] | Metabolism | |
| K00016 | 26 | L-lactate dehydrogenase [EC:1.1.1.27] | Metabolism | |
| K00018 | 11 | glycerate dehydrogenase [EC:1.1.1.29] | Metabolism | |
| K00019 | 33 | 3-hydroxybutyrate dehydrogenase [EC:1.1.1.30] | Metabolism | |
| K00020 | 92 | 3-hydroxyisobutyrate dehydrogenase [EC:1.1.1.31] | Metabolism | |
| K00021 | 14 | 3-hydroxy-3-methylglutaryl-CoA reductase [EC:1.1.1.34] | Metabolism | |
| K00023 | 24 | acetoacetyl-CoA reductase [EC:1.1.1.36] | Metabolism | |
| K00024 | 35 | malate dehydrogenase [EC:1.1.1.37] | Metabolism | |
| K00027 | 40 | malate dehydrogenase (oxaloacetate-decarboxylating) [EC:1.1.1.38] | Metabolism | |
| K00028 | 4 | malate dehydrogenase (decarboxylating) [EC:1.1.1.39] | Metabolism | |
| K00029 | 39 | malate dehydrogenase (oxaloacetate-decarboxylating)(NADP+) | Metabolism | |
| K00030 | 19 | isocitrate dehydrogenase (NAD+) [EC:1.1.1.41] | Metabolism | |
| K00031 | 37 | isocitrate dehydrogenase [EC:1.1.1.42] | Metabolism | |
| K00032 | 1 | phosphogluconate 2-dehydrogenase [EC:1.1.1.43] | Metabolism | |
| K00033 | 37 | 6-phosphogluconate dehydrogenase [EC:1.1.1.44] | Metabolism | |
| K00034 | 25 | glucose 1-dehydrogenase [EC:1.1.1.47] | Metabolism | |
| K00035 | 1 | D-galactose 1-dehydrogenase [EC:1.1.1.48] | Metabolism | |
| K00036 | 118 | glucose-6-phosphate 1-dehydrogenase [EC:1.1.1.49] | Metabolism | |
| K00037 | 2 | 3-alpha-hydroxysteroid dehydrogenase [EC:1.1.1.50] | Metabolism | |
| K00038 | 6 | 3alpha(or 20beta)-hydroxysteroid dehydrogenase [EC:1.1.1.53] | Metabolism | |
| K00040 | 4 | fructuronate reductase [EC:1.1.1.57] | Metabolism | |
| K00042 | 60 | 2-hydroxy-3-oxopropionate reductase [EC:1.1.1.60] | Metabolism | |
| K00043 | 6 | 4-hydroxybutyrate dehydrogenase [EC:1.1.1.61] | Metabolism | |
| K00044 | 10 | estradiol 17beta-dehydrogenase [EC:1.1.1.62] | Metabolism | |
| K00045 | 4 | mannitol 2-dehydrogenase [EC:1.1.1.67] | Metabolism | |
| K00048 | 20 | lactaldehyde reductase [EC:1.1.1.77] | Metabolism | |
| K00050 | 46 | hydroxypyruvate reductase [EC:1.1.1.81] | Metabolism | |
| K00051 | 10 | malate dehydrogenase (NADP+) [EC:1.1.1.82] | Metabolism | |
| K00052 | 66 | 3-isopropylmalate dehydrogenase [EC:1.1.1.85] | Metabolism | |
| K00053 | 17 | ketol-acid reductoisomerase [EC:1.1.1.86] | Metabolism | |
| K00054 | 7 | hydroxymethylglutaryl-CoA reductase [EC:1.1.1.88] | Metabolism | |
| K00055 | 2 | aryl-alcohol dehydrogenase [EC:1.1.1.90] | Metabolism | |
| K00057 | 38 | glycerol-3-phosphate dehydrogenase (NAD(P)+) [EC:1.1.1.94] | Metabolism | |
| K00058 | 145 | D-3-phosphoglycerate dehydrogenase [EC:1.1.1.95] | Metabolism | |
| K00059 | 312 | 3-oxoacyl-[acyl-carrier protein] reductase [EC:1.1.1.100] | Metabolism | |
| K00060 | 50 | threonine 3-dehydrogenase [EC:1.1.1.103] | Metabolism | |
| K00064 | 7 | D-threo-aldose 1-dehydrogenase [EC:1.1.1.122] | Metabolism | |
| K00065 | 29 | 2-deoxy-D-gluconate 3-dehydrogenase [EC:1.1.1.125] | Metabolism | |
| K00066 | 25 | GDP-mannose 6-dehydrogenase [EC:1.1.1.132] | Metabolism | |
| K00067 | 36 | dTDP-4-dehydrorhamnose reductase [EC:1.1.1.133] | Metabolism | |
| K00068 | 7 | sorbitol-6-phosphate 2-dehydrogenase [EC:1.1.1.140] | Metabolism | |
| K00071 | 3 | 11beta-hydroxysteroid dehydrogenase [EC:1.1.1.146] | Metabolism | |
| K00073 | 35 | ureidoglycolate dehydrogenase [EC:1.1.1.154] | Metabolism | |
| K00074 | 63 | 3-hydroxybutyryl-CoA dehydrogenase [EC:1.1.1.157] | Metabolism | |

| | | | | |
|---|---|---|---|---|
| K00075 | 37 | UDP-N-acetylmuramate dehydrogenase [EC:1.1.1.158] | Metabolism | |
| K00077 | 44 | 2-dehydropantoate 2-reductase [EC:1.1.1.169] | Metabolism | |
| K00079 | 12 | carbonyl reductase (NADPH) [EC:1.1.1.184] | Metabolism | |
| K00082 | 9 | 5-amino-6-(5-phosphoribosylamino)uracil reductase [EC:1.1.1.193] | Metabolism | |
| K00083 | 2 | cinnamyl-alcohol dehydrogenase [EC:1.1.1.195] | Metabolism | |
| K00086 | 5 | 1,3-propanediol dehydrogenase [EC:1.1.1.202] | Metabolism | |
| K00087 | 105 | xanthine dehydrogenase molybdenum-binding subunit [EC:1.17.1.4] | Metabolism | |
| K00088 | 84 | IMP dehydrogenase [EC:1.1.1.205] | Metabolism | |
| K00090 | 13 | gluconate 2-dehydrogenase [EC:1.1.1.215] | Metabolism | |
| K00094 | 1 | galactitol-1-phosphate 5-dehydrogenase [EC:1.1.1.251] | Metabolism | |
| K00096 | 3 | glycerol-1-phosphate dehydrogenase [NAD(P)] [EC:1.1.1.261] | Metabolism | |
| K00097 | 42 | 4-hydroxythreonine-4-phosphate dehydrogenase [EC:1.1.1.262] | Metabolism | |
| K00099 | 51 | 1-deoxy-D-xylulose-5-phosphate reductoisomerase [EC:1.1.1.267] | Metabolism | |
| K00100 | 439 | Unclassified; E1.1.1.- | Metabolism | |
| K00101 | 50 | L-lactate dehydrogenase (cytochrome) [EC:1.1.2.3] | Metabolism | |
| K00102 | 85 | D-lactate dehydrogenase (cytochrome) [EC:1.1.2.4] | Metabolism | |
| K00103 | 10 | L-gulonolactone oxidase [EC:1.1.3.8] | Metabolism | |
| K00104 | 91 | glycolate oxidase [EC:1.1.3.15] | Metabolism | |
| K00105 | 3 | alpha-glycerophosphate oxidase [EC:1.1.3.21] | Metabolism | |
| K00108 | 51 | choline dehydrogenase [EC:1.1.99.1] | Metabolism | |
| K00109 | 14 | 2-hydroxyglutarate dehydrogenase [EC:1.1.99.2] | Metabolism | |
| K00111 | 53 | glycerol-3-phosphate dehydrogenase [EC:1.1.5.3] | Metabolism | |
| K00112 | 2 | glycerol-3-phosphate dehydrogenase subunit B [EC:1.1.5.3] | Metabolism | |
| K00113 | 10 | glycerol-3-phosphate dehydrogenase subunit C [EC:1.1.5.3] | Metabolism | |
| K00114 | 80 | alcohol dehydrogenase (acceptor) [EC:1.1.99.8] | Metabolism | |
| K00116 | 24 | malate dehydrogenase (quinone) [EC:1.1.5.4] | Metabolism | |
| K00117 | 55 | quinoprotein glucose dehydrogenase [EC:1.1.5.2] | Metabolism | |
| K00121 | 49 | S-(hydroxymethyl)glutathione dehydrogenase / alcohol dehydrogenase | Metabolism | |
| K00122 | 100 | formate dehydrogenase [EC:1.2.1.2] | Metabolism | |
| K00123 | 164 | formate dehydrogenase, alpha subunit [EC:1.2.1.2] | Metabolism | |
| K00124 | 24 | formate dehydrogenase, beta subunit [EC:1.2.1.2] | Metabolism | |
| K00127 | 21 | formate dehydrogenase, gamma subunit [EC:1.2.1.2] | Metabolism | |
| K00128 | 152 | aldehyde dehydrogenase (NAD+) [EC:1.2.1.3] | Metabolism | |
| K00129 | 2 | aldehyde dehydrogenase (NAD(P)+) [EC:1.2.1.5] | Metabolism | |
| K00130 | 60 | betaine-aldehyde dehydrogenase [EC:1.2.1.8] | Metabolism | |
| K00131 | 21 | glyceraldehyde-3-phosphate dehydrogenase (NADP) [EC:1.2.1.9] | Metabolism | |
| K00132 | 6 | acetaldehyde dehydrogenase (acetylating) [EC:1.2.1.10] | Metabolism | |
| K00133 | 35 | aspartate-semialdehyde dehydrogenase [EC:1.2.1.11] | Metabolism | |
| K00134 | 29 | glyceraldehyde 3-phosphate dehydrogenase [EC:1.2.1.12] | Metabolism | |
| K00135 | 94 | succinate-semialdehyde dehydrogenase (NADP+) [EC:1.2.1.16] | Metabolism | |
| K00137 | 5 | aminobutyraldehyde dehydrogenase [EC:1.2.1.19] | Metabolism | |
| K00138 | 6 | aldehyde dehydrogenase [EC:1.2.1.-] | Metabolism | |
| K00140 | 32 | methylmalonate-semialdehyde dehydrogenase [EC:1.2.1.27] | Metabolism | |
| K00141 | 6 | benzaldehyde dehydrogenase (NAD) [EC:1.2.1.28] | Metabolism | |
| K00145 | 50 | N-acetyl-gamma-glutamyl-phosphate reductase [EC:1.2.1.38] | Metabolism | |
| K00146 | 17 | phenylacetaldehyde dehydrogenase [EC:1.2.1.39] | Metabolism | |
| K00147 | 25 | glutamate-5-semialdehyde dehydrogenase [EC:1.2.1.41] | Metabolism | |
| K00148 | 5 | glutathione-independent formaldehyde dehydrogenase [EC:1.2.1.46] | Metabolism | |
| K00150 | 2 | glyceraldehyde-3-phosphate dehydrogenase (NAD(P)) [EC:1.2.1.59] | Metabolism | |
| K00151 | 15 | 5-carboxymethyl-2-hydroxymuconic-semialdehyde dehydrogenase | Metabolism | |
| K00152 | 5 | salicylaldehyde dehydrogenase [EC:1.2.1.65] | Metabolism | |
| K00156 | 27 | pyruvate dehydrogenase (cytochrome) [EC:1.2.2.2] | Metabolism | |
| K00157 | 22 | aldehyde oxidase [EC:1.2.3.1] | Metabolism | |
| K00158 | 6 | pyruvate oxidase [EC:1.2.3.3] | Metabolism | |
| K00161 | 65 | pyruvate dehydrogenase E1 component subunit alpha [EC:1.2.4.1] | Metabolism | |
| K00162 | 66 | pyruvate dehydrogenase E1 component subunit beta [EC:1.2.4.1] | Metabolism | |
| K00163 | 45 | pyruvate dehydrogenase E1 component [EC:1.2.4.1] | Metabolism | |

| | | | | |
|---|---|---|---|---|
| K00164 | 41 | 2-oxoglutarate dehydrogenase E1 component [EC:1.2.4.2] | Metabolism | |
| K00166 | 32 | 2-oxoisovalerate dehydrogenase E1 component, alpha subunit | Metabolism | |
| K00167 | 31 | 2-oxoisovalerate dehydrogenase E1 component, beta subunit | Metabolism | |
| K00169 | 37 | pyruvate ferredoxin oxidoreductase, alpha subunit [EC:1.2.7.1] | Metabolism | |
| K00170 | 17 | pyruvate ferredoxin oxidoreductase, beta subunit [EC:1.2.7.1] | Metabolism | |
| K00171 | 15 | pyruvate ferredoxin oxidoreductase, delta subunit [EC:1.2.7.1] | Metabolism | |
| K00172 | 16 | pyruvate ferredoxin oxidoreductase, gamma subunit [EC:1.2.7.1] | Metabolism | |
| K00174 | 70 | 2-oxoglutarate ferredoxin oxidoreductase subunit alpha [EC:1.2.7.3] | Metabolism | |
| K00175 | 41 | 2-oxoglutarate ferredoxin oxidoreductase subunit beta [EC:1.2.7.3] | Metabolism | |
| K00176 | 10 | 2-oxoglutarate ferredoxin oxidoreductase subunit delta [EC:1.2.7.3] | Metabolism | |
| K00177 | 12 | 2-oxoglutarate ferredoxin oxidoreductase subunit gamma [EC:1.2.7.3] | Metabolism | |
| K00186 | 10 | 2-oxoisovalerate ferredoxin oxidoreductase, alpha subunit | Metabolism | |
| K00187 | 7 | 2-oxoisovalerate ferredoxin oxidoreductase, beta subunit | Metabolism | |
| K00194 | 1 | carbon-monoxide dehydrogenase delta subunit [EC:1.2.99.2] | Metabolism | |
| K00197 | 5 | carbon-monoxide dehydrogenase gamma subunit [EC:1.2.99.2] | Metabolism | |
| K00198 | 3 | carbon-monoxide dehydrogenase catalytic subunit [EC:1.2.99.2] | Metabolism | |
| K00200 | 8 | formylmethanofuran dehydrogenase subunit A [EC:1.2.99.5] | Metabolism | |
| K00201 | 1 | formylmethanofuran dehydrogenase subunit B [EC:1.2.99.5] | Metabolism | |
| K00202 | 2 | formylmethanofuran dehydrogenase subunit C [EC:1.2.99.5] | Metabolism | |
| K00207 | 10 | dihydropyrimidine dehydrogenase (NADP+) [EC:1.3.1.2] | Metabolism | |
| K00208 | 71 | enoyl-[acyl-carrier protein] reductase I [EC:1.3.1.9] | Metabolism | |
| K00209 | 3 | enoyl-[acyl-carrier-protein] reductase (NADPH2, B-specific) | Metabolism | |
| K00210 | 23 | prephenate dehydrogenase [EC:1.3.1.12] | Metabolism | |
| K00214 | 1 | biliverdin reductase [EC:1.3.1.24] | Metabolism | |
| K00215 | 29 | dihydrodipicolinate reductase [EC:1.3.1.26] | Metabolism | |
| K00216 | 2 | 2,3-dihydro-2,3-dihydroxybenzoate dehydrogenase [EC:1.3.1.28] | Metabolism | |
| K00217 | 4 | maleylacetate reductase [EC:1.3.1.32] | Metabolism | |
| K00218 | 9 | protochlorophyllide reductase [EC:1.3.1.33] | Metabolism | |
| K00220 | 4 | cyclohexadienyl dehydrogenase [EC:1.3.1.43] | Metabolism | |
| K00226 | 72 | dihydroorotate oxidase [EC:1.3.3.1] | Metabolism | |
| K00227 | 3 | lathosterol oxidase [EC:1.14.21.6] | Metabolism | |
| K00228 | 17 | coproporphyrinogen III oxidase [EC:1.3.3.3] | Metabolism | |
| K00230 | 8 | protoporphyrinogen oxidase [EC:1.3.3.4] | Metabolism | |
| K00231 | 27 | protoporphyrinogen oxidase [EC:1.3.3.4] | Metabolism | |
| K00232 | 1 | acyl-CoA oxidase [EC:1.3.3.6] | Metabolism | |
| K00239 | 70 | succinate dehydrogenase flavoprotein subunit [EC:1.3.99.1] | Metabolism | |
| K00240 | 31 | succinate dehydrogenase iron-sulfur protein [EC:1.3.99.1] | Metabolism | |
| K00241 | 21 | succinate dehydrogenase cytochrome b-556 subunit [EC:1.3.99.1] | Metabolism | |
| K00242 | 5 | succinate dehydrogenase hydrophobic membrane anchor protein | Metabolism | |
| K00244 | 39 | fumarate reductase flavoprotein subunit [EC:1.3.99.1] | Metabolism | |
| K00245 | 7 | fumarate reductase iron-sulfur protein [EC:1.3.99.1] | Metabolism | |
| K00246 | 13 | fumarate reductase subunit C [EC:1.3.99.1] | Metabolism | |
| K00247 | 5 | fumarate reductase subunit D [EC:1.3.99.1] | Metabolism | |
| K00248 | 107 | butyryl-CoA dehydrogenase [EC:1.3.99.2] | Metabolism | |
| K00249 | 223 | acyl-CoA dehydrogenase [EC:1.3.99.3] | Metabolism | |
| K00252 | 32 | glutaryl-CoA dehydrogenase [EC:1.3.99.7] | Metabolism | |
| K00253 | 67 | isovaleryl-CoA dehydrogenase [EC:1.3.99.10] | Metabolism | |
| K00255 | 14 | long-chain-acyl-CoA dehydrogenase [EC:1.3.99.13] | Metabolism | |
| K00257 | 333 | Unclassified; E1.3.99.- | Metabolism | |
| K00259 | 29 | alanine dehydrogenase [EC:1.4.1.1] | Metabolism | |
| K00260 | 63 | glutamate dehydrogenase [EC:1.4.1.2] | Metabolism | |
| K00261 | 38 | glutamate dehydrogenase (NAD(P)+) [EC:1.4.1.3] | Metabolism | |
| K00262 | 26 | glutamate dehydrogenase (NADP+) [EC:1.4.1.4] | Metabolism | |
| K00263 | 17 | leucine dehydrogenase [EC:1.4.1.9] | Metabolism | |
| K00265 | 89 | glutamate synthase (NADPH/NADH) large chain [EC:1.4.1.13 1.4.1.14] | Metabolism | |
| K00266 | 101 | glutamate synthase (NADPH/NADH) small chain [EC:1.4.1.13 1.4.1.14] | Metabolism | |
| K00271 | 14 | valine dehydrogenase [EC:1.4.1.-] | Metabolism | |

| | | | | |
|---|---|---|---|---|
| K00273 | 15 | D-amino-acid oxidase [EC:1.4.3.3] | Metabolism | |
| K00274 | 21 | monoamine oxidase [EC:1.4.3.4] | Metabolism | |
| K00275 | 18 | pyridoxamine 5'-phosphate oxidase [EC:1.4.3.5] | Metabolism | |
| K00276 | 60 | primary-amine oxidase [EC:1.4.3.21] | Metabolism | |
| K00278 | 44 | L-aspartate oxidase [EC:1.4.3.16] | Metabolism | |
| K00279 | 15 | cytokinin dehydrogenase [EC:1.5.99.12] | Metabolism | |
| K00281 | 24 | glycine dehydrogenase [EC:1.4.4.2] | Metabolism | |
| K00282 | 30 | glycine dehydrogenase subunit 1 [EC:1.4.4.2] | Metabolism | |
| K00283 | 25 | glycine dehydrogenase subunit 2 [EC:1.4.4.2] | Metabolism | |
| K00284 | 28 | glutamate synthase (ferredoxin) [EC:1.4.7.1] | Metabolism | |
| K00285 | 34 | D-amino-acid dehydrogenase [EC:1.4.99.1] | Metabolism | |
| K00286 | 27 | pyrroline-5-carboxylate reductase [EC:1.5.1.2] | Metabolism | |
| K00287 | 56 | dihydrofolate reductase [EC:1.5.1.3] | Metabolism | |
| K00290 | 30 | saccharopine dehydrogenase (NAD+, L-lysine forming) [EC:1.5.1.7] | Metabolism | |
| K00294 | 69 | 1-pyrroline-5-carboxylate dehydrogenase [EC:1.5.1.12] | Metabolism | |
| K00297 | 67 | methylenetetrahydrofolate reductase (NADPH) [EC:1.5.1.20] | Metabolism | |
| K00299 | 34 | FMN reductase [EC:1.5.1.29] | Metabolism | |
| K00301 | 35 | sarcosine oxidase [EC:1.5.3.1] | Metabolism | |
| K00302 | 11 | sarcosine oxidase, subunit alpha [EC:1.5.3.1] | Metabolism | |
| K00303 | 37 | sarcosine oxidase, subunit beta [EC:1.5.3.1] | Metabolism | |
| K00304 | 2 | sarcosine oxidase, subunit delta [EC:1.5.3.1] | Metabolism | |
| K00305 | 1 | sarcosine oxidase, subunit gamma [EC:1.5.3.1] | Metabolism | |
| K00314 | 44 | sarcosine dehydrogenase [EC:1.5.99.1] | Metabolism | |
| K00315 | 21 | dimethylglycine dehydrogenase [EC:1.5.99.2] | Metabolism | |
| K00317 | 11 | trimethylamine dehydrogenase [EC:1.5.8.2] | Metabolism | |
| K00318 | 21 | proline dehydrogenase [EC:1.5.99.8] | Metabolism | |
| K00320 | 25 | coenzyme F420-dependent N5,N10-methenyltetrahydromethanopterin | Metabolism | |
| K00322 | 12 | NAD(P) transhydrogenase [EC:1.6.1.1] | Metabolism | |
| K00324 | 34 | NAD(P) transhydrogenase subunit alpha [EC:1.6.1.2] | Metabolism | |
| K00325 | 18 | NAD(P) transhydrogenase subunit beta [EC:1.6.1.2] | Metabolism | |
| K00326 | 1 | cytochrome-b5 reductase [EC:1.6.2.2] | Metabolism | |
| K00329 | 36 | NADH dehydrogenase [EC:1.6.5.3] | Metabolism | |
| K00330 | 25 | NADH dehydrogenase I subunit A [EC:1.6.5.3] | Metabolism | |
| K00331 | 27 | NADH dehydrogenase I subunit B [EC:1.6.5.3] | Metabolism | |
| K00332 | 21 | NADH dehydrogenase I subunit C [EC:1.6.5.3] | Metabolism | |
| K00333 | 64 | NADH dehydrogenase I subunit D [EC:1.6.5.3] | Metabolism | |
| K00334 | 43 | NADH dehydrogenase I subunit E [EC:1.6.5.3] | Metabolism | |
| K00335 | 80 | NADH dehydrogenase I subunit F [EC:1.6.5.3] | Metabolism | |
| K00336 | 63 | NADH dehydrogenase I subunit G [EC:1.6.5.3] | Metabolism | |
| K00337 | 55 | NADH dehydrogenase I subunit H [EC:1.6.5.3] | Metabolism | |
| K00338 | 26 | NADH dehydrogenase I subunit I [EC:1.6.5.3] | Metabolism | |
| K00339 | 20 | NADH dehydrogenase I subunit J [EC:1.6.5.3] | Metabolism | |
| K00340 | 15 | NADH dehydrogenase I subunit K [EC:1.6.5.3] | Metabolism | |
| K00341 | 57 | NADH dehydrogenase I subunit L [EC:1.6.5.3] | Metabolism | |
| K00342 | 63 | NADH dehydrogenase I subunit M [EC:1.6.5.3] | Metabolism | |
| K00343 | 62 | NADH dehydrogenase I subunit N [EC:1.6.5.3] | Metabolism | |
| K00355 | 4 | NAD(P)H dehydrogenase (quinone) [EC:1.6.5.2] | Metabolism | |
| K00356 | 59 | NADH dehydrogenase [EC:1.6.99.3] | Metabolism | |
| K00360 | 39 | nitrate reductase (NADH) [EC:1.7.1.1] | Metabolism | |
| K00362 | 26 | nitrite reductase (NAD(P)H) large subunit [EC:1.7.1.4] | Metabolism | |
| K00363 | 13 | nitrite reductase (NAD(P)H) small subunit [EC:1.7.1.4] | Metabolism | |
| K00364 | 4 | GMP reductase [EC:1.7.1.7] | Metabolism | |
| K00365 | 9 | urate oxidase [EC:1.7.3.3] | Metabolism | |
| K00366 | 23 | ferredoxin-nitrite reductase [EC:1.7.7.1] | Metabolism | |
| K00367 | 5 | ferredoxin-nitrate reductase [EC:1.7.7.2] | Metabolism | |
| K00368 | 53 | nitrite reductase (NO-forming) [EC:1.7.2.1] | Metabolism | |
| K00370 | 34 | nitrate reductase 1, alpha subunit [EC:1.7.99.4] | Metabolism | |

| | | | | |
|---|---|---|---|---|
| K00371 | 12 | nitrate reductase 1, beta subunit [EC:1.7.99.4] | Metabolism | |
| K00372 | 36 | nitrate reductase catalytic subunit [EC:1.7.99.4] | Metabolism | |
| K00373 | 4 | nitrate reductase 1, delta subunit [EC:1.7.99.4] | Metabolism | |
| K00374 | 4 | nitrate reductase 1, gamma subunit [EC:1.7.99.4] | Metabolism | |
| K00376 | 14 | nitrous-oxide reductase [EC:1.7.99.6] | Metabolism | |
| K00380 | 19 | sulfite reductase (NADPH) flavoprotein alpha-component [EC:1.8.1.2] | Metabolism | |
| K00381 | 24 | sulfite reductase (NADPH) hemoprotein beta-component [EC:1.8.1.2] | Metabolism | |
| K00382 | 97 | dihydrolipoamide dehydrogenase [EC:1.8.1.4] | Metabolism | |
| K00383 | 19 | glutathione reductase (NADPH) [EC:1.8.1.7] | Metabolism | |
| K00384 | 111 | thioredoxin reductase (NADPH) [EC:1.8.1.9] | Metabolism | |
| K00385 | 1 | anaerobic sulfite reductase subunit C [EC:1.8.1.-] | Metabolism | |
| K00387 | 22 | sulfite oxidase [EC:1.8.3.1] | Metabolism | |
| K00390 | 17 | phosphoadenosine phosphosulfate reductase [EC:1.8.4.8] | Metabolism | |
| K00392 | 14 | sulfite reductase (ferredoxin) [EC:1.8.7.1] | Metabolism | |
| K00394 | 11 | adenylylsulfate reductase, subunit A [EC:1.8.99.2] | Metabolism | |
| K00395 | 3 | adenylylsulfate reductase, subunit B [EC:1.8.99.2] | Metabolism | |
| K00404 | 16 | cb-type cytochrome c oxidase subunit I [EC:1.9.3.1] | Metabolism | |
| K00405 | 17 | cb-type cytochrome c oxidase subunit II [EC:1.9.3.1] | Metabolism | |
| K00406 | 34 | cb-type cytochrome c oxidase subunit III [EC:1.9.3.1] | Metabolism | |
| K00407 | 6 | cb-type cytochrome c oxidase subunit IV [EC:1.9.3.1] | Metabolism | |
| K00411 | 10 | ubiquinol-cytochrome c reductase iron-sulfur subunit [EC:1.10.2.2] | Metabolism | |
| K00412 | 35 | ubiquinol-cytochrome c reductase cytochrome b subunit [EC:1.10.2.2] | Metabolism | |
| K00413 | 8 | ubiquinol-cytochrome c reductase cytochrome c1 subunit [EC:1.10.2.2] | Metabolism | |
| K00423 | 22 | L-ascorbate oxidase [EC:1.10.3.3] | Metabolism | |
| K00425 | 39 | cytochrome bd-I oxidase subunit I [EC:1.10.3.-] | Metabolism | |
| K00426 | 23 | cytochrome bd-I oxidase subunit II [EC:1.10.3.-] | Metabolism | |
| K00432 | 7 | glutathione peroxidase [EC:1.11.1.9] | Metabolism | |
| K00441 | 4 | coenzyme F420 hydrogenase beta subunit [EC:1.12.98.1] | Metabolism | |
| K00442 | 3 | coenzyme F420 hydrogenase delta subunit | Metabolism | |
| K00446 | 7 | catechol 2,3-dioxygenase [EC:1.13.11.2] | Metabolism | |
| K00448 | 10 | protocatechuate 3,4-dioxygenase, alpha subunit [EC:1.13.11.3] | Metabolism | |
| K00449 | 6 | protocatechuate 3,4-dioxygenase, beta subunit [EC:1.13.11.3] | Metabolism | |
| K00450 | 24 | gentisate 1,2-dioxygenase [EC:1.13.11.4] | Metabolism | |
| K00451 | 27 | homogentisate 1,2-dioxygenase [EC:1.13.11.5] | Metabolism | |
| K00453 | 11 | tryptophan 2,3-dioxygenase [EC:1.13.11.11] | Metabolism | |
| K00455 | 1 | 3,4-dihydroxyphenylacetate 2,3-dioxygenase [EC:1.13.11.15] | Metabolism | |
| K00457 | 19 | 4-hydroxyphenylpyruvate dioxygenase [EC:1.13.11.27] | Metabolism | |
| K00459 | 54 | nitronate monooxygenase [EC:1.13.12.16] | Metabolism | |
| K00462 | 4 | biphenyl-2,3-diol 1,2-dioxygenase [EC:1.13.11.39] | Metabolism | |
| K00466 | 5 | tryptophan 2-monooxygenase [EC:1.13.12.3] | Metabolism | |
| K00467 | 12 | lactate 2-monooxygenase [EC:1.13.12.4] | Metabolism | |
| K00480 | 28 | salicylate hydroxylase [EC:1.14.13.1] | Metabolism | |
| K00481 | 6 | p-hydroxybenzoate 3-monooxygenase [EC:1.14.13.2] | Metabolism | |
| K00483 | 31 | 4-hydroxyphenylacetate-3-hydroxylase large chain [EC:1.14.13.3] | Metabolism | |
| K00484 | 2 | 4-hydroxyphenylacetate-3-hydroxylase small chain [EC:1.14.13.3] | Metabolism | |
| K00485 | 11 | dimethylaniline monooxygenase (N-oxide forming) [EC:1.14.13.8] | Metabolism | |
| K00491 | 2 | nitric-oxide synthase, bacterial [EC:1.14.13.39] | Metabolism | |
| K00493 | 46 | unspecific monooxygenase [EC:1.14.14.1] | Metabolism | |
| K00499 | 10 | choline monooxygenase [EC:1.14.15.7] | Metabolism | |
| K00500 | 17 | phenylalanine-4-hydroxylase [EC:1.14.16.1] | Metabolism | |
| K00507 | 40 | stearoyl-CoA desaturase (delta-9 desaturase) [EC:1.14.19.1] | Metabolism | |
| K00508 | 4 | linoleoyl-CoA desaturase [EC:1.14.19.3] | Metabolism | |
| K00514 | 1 | zeta-carotene desaturase [EC:1.14.99.30] | Metabolism | |
| K00517 | 179 | Unclassified; E1.14.-.- | Metabolism | |
| K00523 | 25 | CDP-4-dehydro-6-deoxyglucose reductase [EC:1.17.1.1] | Metabolism | |
| K00525 | 87 | ribonucleoside-diphosphate reductase alpha chain [EC:1.17.4.1] | Metabolism | |
| K00526 | 14 | ribonucleoside-diphosphate reductase beta chain [EC:1.17.4.1] | Metabolism | |

| | | | | |
|---|---|---|---|---|
| K00527 | 5 | ribonucleoside-triphosphate reductase [EC:1.17.4.2] | Metabolism | |
| K00529 | 61 | ferredoxin--NAD+ reductase [EC:1.18.1.3] | Metabolism | |
| K00544 | 5 | betaine-homocysteine S-methyltransferase [EC:2.1.1.5] | Metabolism | |
| K00547 | 33 | homocysteine S-methyltransferase [EC:2.1.1.10] | Metabolism | |
| K00548 | 117 | 5-methyltetrahydrofolate--homocysteine methyltransferase | Metabolism | |
| K00549 | 72 | 5-methyltetrahydropteroyltriglutamate--homocysteine | Metabolism | |
| K00551 | 21 | phosphatidylethanolamine N-methyltransferase [EC:2.1.1.17] | Metabolism | |
| K00558 | 15 | DNA (cytosine-5-)-methyltransferase [EC:2.1.1.37] | Metabolism | |
| K00559 | 1 | sterol 24-C-methyltransferase [EC:2.1.1.41] | Metabolism | |
| K00560 | 9 | thymidylate synthase [EC:2.1.1.45] | Metabolism | |
| K00568 | 65 | 3-demethylubiquinone-9 3-methyltransferase [EC:2.1.1.- 2.1.1.64] | Metabolism | |
| K00569 | 4 | thiopurine S-methyltransferase [EC:2.1.1.67] | Metabolism | |
| K00570 | 5 | phosphatidyl-N-methylethanolamine N-methyltransferase [EC:2.1.1.71] | Metabolism | |
| K00584 | 1 | tetrahydromethanopterin S-methyltransferase subunit H [EC:2.1.1.86] | Metabolism | |
| K00587 | 7 | protein-S-isoprenylcysteine O-methyltransferase [EC:2.1.1.100] | Metabolism | |
| K00588 | 15 | caffeoyl-CoA O-methyltransferase [EC:2.1.1.104] | Metabolism | |
| K00595 | 6 | precorrin-6Y C5,15-methyltransferase / precorrin-8W decarboxylase | Metabolism | |
| K00600 | 49 | glycine hydroxymethyltransferase [EC:2.1.2.1] | Metabolism | |
| K00602 | 28 | phosphoribosylaminoimidazolecarboxamide formyltransferase / IMP | Metabolism | |
| K00603 | 20 | glutamate formiminotransferase [EC:2.1.2.5] | Metabolism | |
| K00604 | 50 | methionyl-tRNA formyltransferase [EC:2.1.2.9] | Metabolism | |
| K00605 | 69 | aminomethyltransferase [EC:2.1.2.10] | Metabolism | |
| K00606 | 26 | 3-methyl-2-oxobutanoate hydroxymethyltransferase [EC:2.1.2.11] | Metabolism | |
| K00609 | 35 | aspartate carbamoyltransferase catalytic subunit [EC:2.1.3.2] | Metabolism | |
| K00610 | 12 | aspartate carbamoyltransferase regulatory subunit | Metabolism | |
| K00611 | 50 | ornithine carbamoyltransferase [EC:2.1.3.3] | Metabolism | |
| K00613 | 1 | glycine amidinotransferase [EC:2.1.4.1] | Metabolism | |
| K00615 | 102 | transketolase [EC:2.2.1.1] | Metabolism | |
| K00616 | 40 | transaldolase [EC:2.2.1.2] | Metabolism | |
| K00619 | 22 | amino-acid N-acetyltransferase [EC:2.3.1.1] | Metabolism | |
| K00620 | 31 | glutamate N-acetyltransferase / amino-acid N-acetyltransferase | Metabolism | |
| K00622 | 5 | arylamine N-acetyltransferase [EC:2.3.1.5] | Metabolism | |
| K00625 | 21 | phosphate acetyltransferase [EC:2.3.1.8] | Metabolism | |
| K00626 | 211 | acetyl-CoA C-acetyltransferase [EC:2.3.1.9] | Metabolism | |
| K00627 | 68 | pyruvate dehydrogenase E2 component (dihydrolipoamide | Metabolism | |
| K00631 | 12 | glycerol-3-phosphate O-acyltransferase [EC:2.3.1.15] | Metabolism | |
| K00632 | 93 | acetyl-CoA acyltransferase [EC:2.3.1.16] | Metabolism | |
| K00634 | 11 | phosphate butyryltransferase [EC:2.3.1.19] | Metabolism | |
| K00639 | 42 | glycine C-acetyltransferase [EC:2.3.1.29] | Metabolism | |
| K00640 | 27 | serine O-acetyltransferase [EC:2.3.1.30] | Metabolism | |
| K00641 | 37 | homoserine O-acetyltransferase [EC:2.3.1.31] | Metabolism | |
| K00643 | 12 | 5-aminolevulinate synthase [EC:2.3.1.37] | Metabolism | |
| K00645 | 48 | [acyl-carrier-protein] S-malonyltransferase [EC:2.3.1.39] | Metabolism | |
| K00647 | 39 | 3-oxoacyl-[acyl-carrier-protein] synthase I [EC:2.3.1.41] | Metabolism | |
| K00648 | 79 | 3-oxoacyl-[acyl-carrier-protein] synthase III [EC:2.3.1.180] | Metabolism | |
| K00651 | 3 | homoserine O-succinyltransferase [EC:2.3.1.46] | Metabolism | |
| K00652 | 51 | 8-amino-7-oxononanoate synthase [EC:2.3.1.47] | Metabolism | |
| K00654 | 7 | serine palmitoyltransferase [EC:2.3.1.50] | Metabolism | |
| K00655 | 121 | 1-acyl-sn-glycerol-3-phosphate acyltransferase [EC:2.3.1.51] | Metabolism | |
| K00656 | 38 | formate C-acetyltransferase [EC:2.3.1.54] | Metabolism | |
| K00657 | 13 | diamine N-acetyltransferase [EC:2.3.1.57] | Metabolism | |
| K00658 | 41 | 2-oxoglutarate dehydrogenase E2 component (dihydrolipoamide | Metabolism | |
| K00660 | 12 | chalcone synthase [EC:2.3.1.74] | Metabolism | |
| K00672 | 6 | formylmethanofuran--tetrahydromethanopterin N-formyltransferase | Metabolism | |
| K00673 | 3 | arginine N-succinyltransferase [EC:2.3.1.109] | Metabolism | |
| K00674 | 18 | 2,3,4,5-tetrahydropyridine-2-carboxylate N-succinyltransferase | Metabolism | |
| K00677 | 32 | UDP-N-acetylglucosamine acyltransferase [EC:2.3.1.129] | Metabolism | |

| | | | | |
|---|---|---|---|---|
| K00681 | 137 | gamma-glutamyltranspeptidase [EC:2.3.2.2] | Metabolism | |
| K00688 | 90 | starch phosphorylase [EC:2.4.1.1] | Metabolism | |
| K00690 | 3 | sucrose phosphorylase [EC:2.4.1.7] | Metabolism | |
| K00691 | 17 | maltose phosphorylase [EC:2.4.1.8] | Metabolism | |
| K00693 | 14 | glycogen(starch) synthase [EC:2.4.1.11] | Metabolism | |
| K00694 | 12 | cellulose synthase (UDP-forming) [EC:2.4.1.12] | Metabolism | |
| K00695 | 2 | sucrose synthase [EC:2.4.1.13] | Metabolism | |
| K00696 | 1 | sucrose-phosphate synthase [EC:2.4.1.14] | Metabolism | |
| K00697 | 44 | alpha,alpha-trehalose-phosphate synthase (UDP-forming) [EC:2.4.1.15] | Metabolism | |
| K00700 | 62 | 1,4-alpha-glucan branching enzyme [EC:2.4.1.18] | Metabolism | |
| K00702 | 10 | cellobiose phosphorylase [EC:2.4.1.20] | Metabolism | |
| K00703 | 39 | starch synthase [EC:2.4.1.21] | Metabolism | |
| K00705 | 74 | 4-alpha-glucanotransferase [EC:2.4.1.25] | Metabolism | |
| K00720 | 5 | ceramide glucosyltransferase [EC:2.4.1.80] | Metabolism | |
| K00721 | 123 | dolichol-phosphate mannosyltransferase [EC:2.4.1.83] | Metabolism | |
| K00743 | 2 | N-acetyllactosaminide 3-alpha-galactosyltransferase [EC:2.4.1.87] | Metabolism | |
| K00748 | 28 | lipid-A-disaccharide synthase [EC:2.4.1.182] | Metabolism | |
| K00756 | 8 | pyrimidine-nucleoside phosphorylase [EC:2.4.2.2] | Metabolism | |
| K00757 | 2 | uridine phosphorylase [EC:2.4.2.3] | Metabolism | |
| K00758 | 21 | thymidine phosphorylase [EC:2.4.2.4] | Metabolism | |
| K00759 | 26 | adenine phosphoribosyltransferase [EC:2.4.2.7] | Metabolism | |
| K00760 | 33 | hypoxanthine phosphoribosyltransferase [EC:2.4.2.8] | Metabolism | |
| K00761 | 13 | uracil phosphoribosyltransferase [EC:2.4.2.9] | Metabolism | |
| K00762 | 22 | orotate phosphoribosyltransferase [EC:2.4.2.10] | Metabolism | |
| K00763 | 180 | nicotinate phosphoribosyltransferase [EC:2.4.2.11] | Metabolism | |
| K00764 | 58 | amidophosphoribosyltransferase [EC:2.4.2.14] | Metabolism | |
| K00765 | 31 | ATP phosphoribosyltransferase [EC:2.4.2.17] | Metabolism | |
| K00766 | 31 | anthranilate phosphoribosyltransferase [EC:2.4.2.18] | Metabolism | |
| K00767 | 54 | nicotinate-nucleotide pyrophosphorylase (carboxylating) | Metabolism | |
| K00768 | 8 | nicotinate-nucleotide--dimethylbenzimidazole | Metabolism | |
| K00772 | 30 | 5'-methylthioadenosine phosphorylase [EC:2.4.2.28] | Metabolism | |
| K00788 | 35 | thiamine-phosphate pyrophosphorylase [EC:2.5.1.3] | Metabolism | |
| K00789 | 31 | S-adenosylmethionine synthetase [EC:2.5.1.6] | Metabolism | |
| K00790 | 50 | UDP-N-acetylglucosamine 1-carboxyvinyltransferase [EC:2.5.1.7] | Metabolism | |
| K00791 | 29 | tRNA dimethylallyltransferase [EC:2.5.1.75] | Metabolism | |
| K00793 | 31 | riboflavin synthase alpha chain [EC:2.5.1.9] | Metabolism | |
| K00794 | 15 | riboflavin synthase beta chain [EC:2.5.1.-] | Metabolism | |
| K00795 | 8 | farnesyl diphosphate synthase [EC:2.5.1.1 2.5.1.10] | Metabolism | |
| K00796 | 35 | dihydropteroate synthase [EC:2.5.1.15] | Metabolism | |
| K00797 | 37 | spermidine synthase [EC:2.5.1.16] | Metabolism | |
| K00798 | 27 | cob(I)alamin adenosyltransferase [EC:2.5.1.17] | Metabolism | |
| K00799 | 108 | glutathione S-transferase [EC:2.5.1.18] | Metabolism | |
| K00800 | 62 | 3-phosphoshikimate 1-carboxyvinyltransferase [EC:2.5.1.19] | Metabolism | |
| K00801 | 13 | farnesyl-diphosphate farnesyltransferase [EC:2.5.1.21] | Metabolism | |
| K00802 | 3 | spermine synthase [EC:2.5.1.22] | Metabolism | |
| K00803 | 23 | alkyldihydroxyacetonephosphate synthase [EC:2.5.1.26] | Metabolism | |
| K00805 | 9 | heptaprenyl diphosphate synthase [EC:2.5.1.30] | Metabolism | |
| K00806 | 20 | undecaprenyl diphosphate synthase [EC:2.5.1.31] | Metabolism | |
| K00808 | 4 | homospermidine synthase [EC:2.5.1.44] | Metabolism | |
| K00811 | 2 | aspartate aminotransferase [EC:2.6.1.1] | Metabolism | |
| K00812 | 46 | aspartate aminotransferase [EC:2.6.1.1] | Metabolism | |
| K00813 | 7 | aspartate aminotransferase [EC:2.6.1.1] | Metabolism | |
| K00814 | 3 | alanine transaminase [EC:2.6.1.2] | Metabolism | |
| K00817 | 59 | histidinol-phosphate aminotransferase [EC:2.6.1.9] | Metabolism | |
| K00818 | 49 | acetylornithine aminotransferase [EC:2.6.1.11] | Metabolism | |
| K00819 | 14 | ornithine--oxo-acid transaminase [EC:2.6.1.13] | Metabolism | |
| K00820 | 67 | glucosamine--fructose-6-phosphate aminotransferase (isomerizing) | Metabolism | |

| | | | | |
|---|---|---|---|---|
| K00821 | 47 | acetylornithine/N-succinyldiaminopimelate aminotransferase | Metabolism | |
| K00822 | 14 | beta-alanine--pyruvate transaminase [EC:2.6.1.18] | Metabolism | |
| K00823 | 47 | 4-aminobutyrate aminotransferase [EC:2.6.1.19] | Metabolism | |
| K00824 | 14 | D-alanine transaminase [EC:2.6.1.21] | Metabolism | |
| K00825 | 17 | 2-aminoadipate transaminase [EC:2.6.1.39] | Metabolism | |
| K00826 | 73 | branched-chain amino acid aminotransferase [EC:2.6.1.42] | Metabolism | |
| K00831 | 23 | phosphoserine aminotransferase [EC:2.6.1.52] | Metabolism | |
| K00832 | 8 | aromatic-amino-acid transaminase [EC:2.6.1.57] | Metabolism | |
| K00833 | 19 | adenosylmethionine-8-amino-7-oxononanoate aminotransferase | Metabolism | |
| K00835 | 12 | valine--pyruvate aminotransferase [EC:2.6.1.66] | Metabolism | |
| K00836 | 5 | diaminobutyrate-2-oxoglutarate transaminase [EC:2.6.1.76] | Metabolism | |
| K00839 | 38 | aminotransferase [EC:2.6.1.-] | Metabolism | |
| K00840 | 1 | succinylornithine aminotransferase [EC:2.6.1.81] | Metabolism | |
| K00841 | 1 | aminotransferase [EC:2.6.1.-] | Metabolism | |
| K00845 | 73 | glucokinase [EC:2.7.1.2] | Metabolism | |
| K00846 | 2 | ketohexokinase [EC:2.7.1.3] | Metabolism | |
| K00847 | 33 | fructokinase [EC:2.7.1.4] | Metabolism | |
| K00848 | 14 | rhamnulokinase [EC:2.7.1.5] | Metabolism | |
| K00849 | 14 | galactokinase [EC:2.7.1.6] | Metabolism | |
| K00850 | 56 | 6-phosphofructokinase [EC:2.7.1.11] | Metabolism | |
| K00851 | 14 | gluconokinase [EC:2.7.1.12] | Metabolism | |
| K00852 | 25 | ribokinase [EC:2.7.1.15] | Metabolism | |
| K00853 | 8 | L-ribulokinase [EC:2.7.1.16] | Metabolism | |
| K00854 | 34 | xylulokinase [EC:2.7.1.17] | Metabolism | |
| K00855 | 13 | phosphoribulokinase [EC:2.7.1.19] | Metabolism | |
| K00856 | 21 | adenosine kinase [EC:2.7.1.20] | Metabolism | |
| K00857 | 8 | thymidine kinase [EC:2.7.1.21] | Metabolism | |
| K00858 | 35 | NAD+ kinase [EC:2.7.1.23] | Metabolism | |
| K00859 | 35 | dephospho-CoA kinase [EC:2.7.1.24] | Metabolism | |
| K00860 | 18 | adenylylsulfate kinase [EC:2.7.1.25] | Metabolism | |
| K00861 | 3 | riboflavin kinase [EC:2.7.1.26] | Metabolism | |
| K00863 | 8 | dihydroxyacetone kinase [EC:2.7.1.29] | Metabolism | |
| K00864 | 41 | glycerol kinase [EC:2.7.1.30] | Metabolism | |
| K00865 | 12 | glycerate kinase [EC:2.7.1.31] | Metabolism | |
| K00866 | 1 | choline kinase [EC:2.7.1.32] | Metabolism | |
| K00867 | 2 | type I pantothenate kinase [EC:2.7.1.33] | Metabolism | |
| K00868 | 445 | pyridoxine kinase [EC:2.7.1.35] | Metabolism | |
| K00869 | 1 | mevalonate kinase [EC:2.7.1.36] | Metabolism | |
| K00872 | 9 | homoserine kinase [EC:2.7.1.39] | Metabolism | |
| K00873 | 44 | pyruvate kinase [EC:2.7.1.40] | Metabolism | |
| K00874 | 20 | 2-dehydro-3-deoxygluconokinase [EC:2.7.1.45] | Metabolism | |
| K00875 | 2 | D-ribulokinase [EC:2.7.1.47] | Metabolism | |
| K00876 | 12 | uridine kinase [EC:2.7.1.48] | Metabolism | |
| K00877 | 3 | hydroxymethylpyrimidine kinase [EC:2.7.1.49] | Metabolism | |
| K00878 | 3 | hydroxyethylthiazole kinase [EC:2.7.1.50] | Metabolism | |
| K00879 | 4 | L-fuculokinase [EC:2.7.1.51] | Metabolism | |
| K00880 | 1 | L-xylulokinase [EC:2.7.1.53] | Metabolism | |
| K00882 | 14 | 1-phosphofructokinase [EC:2.7.1.56] | Metabolism | |
| K00883 | 1 | 2-dehydro-3-deoxygalactonokinase [EC:2.7.1.58] | Metabolism | |
| K00884 | 7 | N-acetylglucosamine kinase [EC:2.7.1.59] | Metabolism | |
| K00885 | 1 | N-acylmannosamine kinase [EC:2.7.1.60] | Metabolism | |
| K00886 | 9 | polyphosphate glucokinase [EC:2.7.1.63] | Metabolism | |
| K00887 | 10 | undecaprenol kinase [EC:2.7.1.66] | Metabolism | |
| K00891 | 31 | shikimate kinase [EC:2.7.1.71] | Metabolism | |
| K00894 | 3 | ethanolamine kinase [EC:2.7.1.82] | Metabolism | |
| K00895 | 22 | pyrophosphate--fructose-6-phosphate 1-phosphotransferase | Metabolism | |
| K00901 | 6 | diacylglycerol kinase [EC:2.7.1.107] | Metabolism | |

| | | | | |
|---|---|---|---|---|
| K00904 | 18 | deoxyguanosine kinase [EC:2.7.1.113] | Metabolism | |
| K00912 | 13 | tetraacyldisaccharide 4'-kinase [EC:2.7.1.130] | Metabolism | |
| K00917 | 9 | tagatose 6-phosphate kinase [EC:2.7.1.144] | Metabolism | |
| K00919 | 25 | 4-diphosphocytidyl-2-C-methyl-D-erythritol kinase [EC:2.7.1.148] | Metabolism | |
| K00925 | 31 | acetate kinase [EC:2.7.2.1] | Metabolism | |
| K00926 | 18 | carbamate kinase [EC:2.7.2.2] | Metabolism | |
| K00927 | 35 | phosphoglycerate kinase [EC:2.7.2.3] | Metabolism | |
| K00928 | 33 | aspartate kinase [EC:2.7.2.4] | Metabolism | |
| K00929 | 3 | butyrate kinase [EC:2.7.2.7] | Metabolism | |
| K00930 | 23 | acetylglutamate kinase [EC:2.7.2.8] | Metabolism | |
| K00931 | 54 | glutamate 5-kinase [EC:2.7.2.11] | Metabolism | |
| K00932 | 1 | propionate kinase [EC:2.7.2.15] | Metabolism | |
| K00933 | 1 | creatine kinase [EC:2.7.3.2] | Metabolism | |
| K00934 | 4 | arginine kinase [EC:2.7.3.3] | Metabolism | |
| K00937 | 60 | polyphosphate kinase [EC:2.7.4.1] | Metabolism | |
| K00938 | 5 | phosphomevalonate kinase [EC:2.7.4.2] | Metabolism | |
| K00939 | 34 | adenylate kinase [EC:2.7.4.3] | Metabolism | |
| K00940 | 20 | nucleoside-diphosphate kinase [EC:2.7.4.6] | Metabolism | |
| K00941 | 29 | phosphomethylpyrimidine kinase [EC:2.7.4.7] | Metabolism | |
| K00942 | 26 | guanylate kinase [EC:2.7.4.8] | Metabolism | |
| K00943 | 33 | dTMP kinase [EC:2.7.4.9] | Metabolism | |
| K00945 | 33 | cytidylate kinase [EC:2.7.4.14] | Metabolism | |
| K00946 | 33 | thiamine-monophosphate kinase [EC:2.7.4.16] | Metabolism | |
| K00948 | 177 | ribose-phosphate pyrophosphokinase [EC:2.7.6.1] | Metabolism | |
| K00950 | 17 | 2-amino-4-hydroxy-6-hydroxymethyldihydropteridine diphosphokinase | Metabolism | |
| K00951 | 90 | GTP pyrophosphokinase [EC:2.7.6.5] | Metabolism | |
| K00954 | 23 | pantetheine-phosphate adenylyltransferase [EC:2.7.7.3] | Metabolism | |
| K00955 | 28 | bifunctional enzyme CysN/CysC [EC:2.7.7.4 2.7.1.25] | Metabolism | |
| K00956 | 29 | sulfate adenylyltransferase subunit 1 [EC:2.7.7.4] | Metabolism | |
| K00957 | 10 | sulfate adenylyltransferase subunit 2 [EC:2.7.7.4] | Metabolism | |
| K00958 | 25 | sulfate adenylyltransferase [EC:2.7.7.4] | Metabolism | |
| K00962 | 39 | polyribonucleotide nucleotidyltransferase [EC:2.7.7.8] | Metabolism | |
| K00963 | 23 | UTP--glucose-1-phosphate uridylyltransferase [EC:2.7.7.9] | Metabolism | |
| K00965 | 24 | UDPglucose--hexose-1-phosphate uridylyltransferase [EC:2.7.7.12] | Metabolism | |
| K00966 | 49 | mannose-1-phosphate guanylyltransferase [EC:2.7.7.13] | Metabolism | |
| K00969 | 56 | nicotinate-nucleotide adenylyltransferase [EC:2.7.7.18] | Metabolism | |
| K00971 | 42 | mannose-1-phosphate guanylyltransferase [EC:2.7.7.22] | Metabolism | |
| K00972 | 3 | UDP-N-acetylglucosamine pyrophosphorylase [EC:2.7.7.23] | Metabolism | |
| K00973 | 45 | glucose-1-phosphate thymidylyltransferase [EC:2.7.7.24] | Metabolism | |
| K00975 | 42 | glucose-1-phosphate adenylyltransferase [EC:2.7.7.27] | Metabolism | |
| K00978 | 15 | glucose-1-phosphate cytidylyltransferase [EC:2.7.7.33] | Metabolism | |
| K00979 | 26 | 3-deoxy-manno-octulosonate cytidylyltransferase (CMP-KDO synthetase) | Metabolism | |
| K00980 | 8 | glycerol-3-phosphate cytidylyltransferase [EC:2.7.7.39] | Metabolism | |
| K00981 | 27 | phosphatidate cytidylyltransferase [EC:2.7.7.41] | Metabolism | |
| K00983 | 2 | N-acylneuraminate cytidylyltransferase [EC:2.7.7.43] | Metabolism | |
| K00991 | 20 | 2-C-methyl-D-erythritol 4-phosphate cytidylyltransferase | Metabolism | |
| K00992 | 28 | Unclassified; E2.7.7.- | Metabolism | |
| K00995 | 115 | CDP-diacylglycerol--glycerol-3-phosphate 3-phosphatidyltransferase | Metabolism | |
| K00997 | 15 | holo-[acyl-carrier protein] synthase [EC:2.7.8.7] | Metabolism | |
| K00998 | 20 | phosphatidylserine synthase [EC:2.7.8.8] | Metabolism | |
| K00999 | 4 | CDP-diacylglycerol--inositol 3-phosphatidyltransferase [EC:2.7.8.11] | Metabolism | |
| K01000 | 52 | phospho-N-acetylmuramoyl-pentapeptide-transferase [EC:2.7.8.13] | Metabolism | |
| K01003 | 30 | carboxyvinyl-carboxyphosphonate phosphorylmutase [EC:2.7.8.23] | Metabolism | |
| K01006 | 55 | pyruvate,orthophosphate dikinase [EC:2.7.9.1] | Metabolism | |
| K01007 | 80 | pyruvate, water dikinase [EC:2.7.9.2] | Metabolism | |
| K01008 | 29 | selenide, water dikinase [EC:2.7.9.3] | Metabolism | |
| K01011 | 21 | 3-mercaptopyruvate sulfurtransferase [EC:2.8.1.2] | Metabolism | |

| | | | | |
|---|---|---|---|---|
| K01012 | 22 | biotin synthetase [EC:2.8.1.6] | Metabolism | |
| K01026 | 7 | propionate CoA-transferase [EC:2.8.3.1] | Metabolism | |
| K01027 | 3 | 3-oxoacid CoA-transferase [EC:2.8.3.5] | Metabolism | |
| K01028 | 6 | 3-oxoacid CoA-transferase subunit A [EC:2.8.3.5] | Metabolism | |
| K01029 | 3 | 3-oxoacid CoA-transferase subunit B [EC:2.8.3.5] | Metabolism | |
| K01031 | 3 | 3-oxoadipate CoA-transferase, alpha subunit [EC:2.8.3.6] | Metabolism | |
| K01032 | 3 | 3-oxoadipate CoA-transferase, beta subunit [EC:2.8.3.6] | Metabolism | |
| K01035 | 2 | acetate CoA-transferase beta subunit [EC:2.8.3.8] | Metabolism | |
| K01039 | 25 | glutaconate CoA-transferase, subunit A [EC:2.8.3.12] | Metabolism | |
| K01040 | 18 | glutaconate CoA-transferase, subunit B [EC:2.8.3.12] | Metabolism | |
| K01042 | 37 | L-seryl-tRNA(Ser) seleniumtransferase [EC:2.9.1.1] | Metabolism | |
| K01044 | 56 | carboxylesterase [EC:3.1.1.1] | Metabolism | |
| K01046 | 68 | triacylglycerol lipase [EC:3.1.1.3] | Metabolism | |
| K01048 | 6 | lysophospholipase [EC:3.1.1.5] | Metabolism | |
| K01051 | 2 | pectinesterase [EC:3.1.1.11] | Metabolism | |
| K01053 | 63 | gluconolactonase [EC:3.1.1.17] | Metabolism | |
| K01054 | 3 | acylglycerol lipase [EC:3.1.1.23] | Metabolism | |
| K01055 | 56 | 3-oxoadipate enol-lactonase [EC:3.1.1.24] | Metabolism | |
| K01057 | 24 | 6-phosphogluconolactonase [EC:3.1.1.31] | Metabolism | |
| K01058 | 8 | phospholipase A1 [EC:3.1.1.32] | Metabolism | |
| K01060 | 3 | cephalosporin-C deacetylase [EC:3.1.1.41] | Metabolism | |
| K01061 | 68 | carboxymethylenebutenolidase [EC:3.1.1.45] | Metabolism | |
| K01062 | 1 | 1-alkyl-2-acetylglycerophosphocholine esterase [EC:3.1.1.47] | Metabolism | |
| K01067 | 12 | acetyl-CoA hydrolase [EC:3.1.2.1] | Metabolism | |
| K01068 | 5 | palmitoyl-CoA hydrolase [EC:3.1.2.2] | Metabolism | |
| K01069 | 110 | hydroxyacylglutathione hydrolase [EC:3.1.2.6] | Metabolism | |
| K01070 | 5 | S-formylglutathione hydrolase [EC:3.1.2.12] | Metabolism | |
| K01071 | 2 | oleoyl-[acyl-carrier-protein] hydrolase [EC:3.1.2.14] | Metabolism | |
| K01075 | 12 | 4-hydroxybenzoyl-CoA thioesterase [EC:3.1.2.23] | Metabolism | |
| K01077 | 90 | alkaline phosphatase [EC:3.1.3.1] | Metabolism | |
| K01078 | 17 | acid phosphatase [EC:3.1.3.2] | Metabolism | |
| K01079 | 34 | phosphoserine phosphatase [EC:3.1.3.3] | Metabolism | |
| K01081 | 32 | 5'-nucleotidase [EC:3.1.3.5] | Metabolism | |
| K01083 | 1 | 3-phytase [EC:3.1.3.8] | Metabolism | |
| K01087 | 73 | trehalose-phosphatase [EC:3.1.3.12] | Metabolism | |
| K01091 | 72 | phosphoglycolate phosphatase [EC:3.1.3.18] | Metabolism | |
| K01092 | 58 | myo-inositol-1(or 4)-monophosphatase [EC:3.1.3.25] | Metabolism | |
| K01093 | 4 | 4-phytase / acid phosphatase [EC:3.1.3.26 3.1.3.2] | Metabolism | |
| K01095 | 8 | phosphatidylglycerophosphatase A [EC:3.1.3.27] | Metabolism | |
| K01096 | 4 | phosphatidylglycerophosphatase B [EC:3.1.3.27] | Metabolism | |
| K01101 | 7 | 4-nitrophenyl phosphatase [EC:3.1.3.41] | Metabolism | |
| K01103 | 1 | fructose-2,6-bisphosphatase [EC:3.1.3.46] | Metabolism | |
| K01113 | 42 | phosphodiesterase/alkaline phosphatase D [EC:3.1.4.1] | Metabolism | |
| K01114 | 33 | phospholipase C [EC:3.1.4.3] | Metabolism | |
| K01115 | 8 | phospholipase D [EC:3.1.4.4] | Metabolism | |
| K01119 | 13 | 2',3'-cyclic-nucleotide 2'-phosphodiesterase [EC:3.1.4.16] | Metabolism | |
| K01120 | 22 | 3',5'-cyclic-nucleotide phosphodiesterase [EC:3.1.4.17] | Metabolism | |
| K01126 | 47 | glycerophosphoryl diester phosphodiesterase [EC:3.1.4.46] | Metabolism | |
| K01129 | 24 | dGTPase [EC:3.1.5.1] | Metabolism | |
| K01130 | 86 | arylsulfatase [EC:3.1.6.1] | Metabolism | |
| K01131 | 7 | steryl-sulfatase [EC:3.1.6.2] | Metabolism | |
| K01132 | 17 | N-acetylgalactosamine-6-sulfatase [EC:3.1.6.4] | Metabolism | |
| K01134 | 23 | arylsulfatase A [EC:3.1.6.8] | Metabolism | |
| K01135 | 17 | arylsulfatase B [EC:3.1.6.12] | Metabolism | |
| K01136 | 8 | iduronate 2-sulfatase [EC:3.1.6.13] | Metabolism | |
| K01137 | 10 | N-acetylglucosamine-6-sulfatase [EC:3.1.6.14] | Metabolism | |
| K01139 | 45 | guanosine-3',5'-bis(diphosphate) 3'-pyrophosphohydrolase | Metabolism | |

| | | | | |
|---|---|---|---|---|
| K01176 | 36 | alpha-amylase [EC:3.2.1.1] | Metabolism | |
| K01178 | 57 | glucoamylase [EC:3.2.1.3] | Metabolism | |
| K01179 | 39 | endoglucanase [EC:3.2.1.4] | Metabolism | |
| K01182 | 13 | oligo-1,6-glucosidase [EC:3.2.1.10] | Metabolism | |
| K01183 | 28 | chitinase [EC:3.2.1.14] | Metabolism | |
| K01184 | 1 | polygalacturonase [EC:3.2.1.15] | Metabolism | |
| K01186 | 2 | sialidase-1 [EC:3.2.1.18] | Metabolism | |
| K01187 | 42 | alpha-glucosidase [EC:3.2.1.20] | Metabolism | |
| K01188 | 77 | beta-glucosidase [EC:3.2.1.21] | Metabolism | |
| K01190 | 51 | beta-galactosidase [EC:3.2.1.23] | Metabolism | |
| K01191 | 17 | alpha-mannosidase [EC:3.2.1.24] | Metabolism | |
| K01192 | 9 | beta-mannosidase [EC:3.2.1.25] | Metabolism | |
| K01193 | 2 | beta-fructofuranosidase [EC:3.2.1.26] | Metabolism | |
| K01194 | 6 | alpha,alpha-trehalase [EC:3.2.1.28] | Metabolism | |
| K01195 | 8 | beta-glucuronidase [EC:3.2.1.31] | Metabolism | |
| K01197 | 1 | hyaluronoglucosaminidase [EC:3.2.1.35] | Metabolism | |
| K01198 | 9 | xylan 1,4-beta-xylosidase [EC:3.2.1.37] | Metabolism | |
| K01199 | 9 | glucan endo-1,3-beta-D-glucosidase [EC:3.2.1.39] | Metabolism | |
| K01201 | 13 | glucosylceramidase [EC:3.2.1.45] | Metabolism | |
| K01206 | 21 | alpha-L-fucosidase [EC:3.2.1.51] | Metabolism | |
| K01207 | 53 | beta-N-acetylhexosaminidase [EC:3.2.1.52] | Metabolism | |
| K01208 | 1 | cyclomaltodextrinase [EC:3.2.1.54] | Metabolism | |
| K01209 | 22 | alpha-N-arabinofuranosidase [EC:3.2.1.55] | Metabolism | |
| K01210 | 10 | glucan 1,3-beta-glucosidase [EC:3.2.1.58] | Metabolism | |
| K01212 | 11 | levanase [EC:3.2.1.65] | Metabolism | |
| K01213 | 6 | galacturan 1,4-alpha-galacturonidase [EC:3.2.1.67] | Metabolism | |
| K01218 | 6 | mannan endo-1,4-beta-mannosidase [EC:3.2.1.78] | Metabolism | |
| K01220 | 15 | 6-phospho-beta-galactosidase [EC:3.2.1.85] | Metabolism | |
| K01222 | 6 | 6-phospho-beta-glucosidase [EC:3.2.1.86] | Metabolism | |
| K01223 | 1 | 6-phospho-beta-glucosidase [EC:3.2.1.86] | Metabolism | |
| K01225 | 25 | cellulose 1,4-beta-cellobiosidase [EC:3.2.1.91] | Metabolism | |
| K01226 | 3 | trehalose-6-phosphate hydrolase [EC:3.2.1.93] | Metabolism | |
| K01236 | 28 | maltooligosyltrehalose trehalohydrolase [EC:3.2.1.141] | Metabolism | |
| K01239 | 17 | purine nucleosidase [EC:3.2.2.1] | Metabolism | |
| K01241 | 4 | AMP nucleosidase [EC:3.2.2.4] | Metabolism | |
| K01243 | 6 | S-adenosylhomocysteine/5'-methylthioadenosine nucleosidase | Metabolism | |
| K01251 | 29 | adenosylhomocysteinase [EC:3.3.1.1] | Metabolism | |
| K01253 | 35 | microsomal epoxide hydrolase [EC:3.3.2.9] | Metabolism | |
| K01255 | 52 | leucyl aminopeptidase [EC:3.4.11.1] | Metabolism | |
| K01256 | 102 | aminopeptidase N [EC:3.4.11.2] | Metabolism | |
| K01259 | 49 | proline iminopeptidase [EC:3.4.11.5] | Metabolism | |
| K01270 | 3 | aminoacylhistidine dipeptidase [EC:3.4.13.3] | Metabolism | |
| K01424 | 54 | L-asparaginase [EC:3.5.1.1] | Metabolism | |
| K01425 | 10 | glutaminase [EC:3.5.1.2] | Metabolism | |
| K01426 | 156 | amidase [EC:3.5.1.4] | Metabolism | |
| K01428 | 11 | urease alpha subunit [EC:3.5.1.5] | Metabolism | |
| K01429 | 3 | urease beta subunit [EC:3.5.1.5] | Metabolism | |
| K01430 | 6 | urease gamma subunit [EC:3.5.1.5] | Metabolism | |
| K01431 | 27 | beta-ureidopropionase [EC:3.5.1.6] | Metabolism | |
| K01433 | 7 | formyltetrahydrofolate deformylase [EC:3.5.1.10] | Metabolism | |
| K01434 | 69 | penicillin amidase [EC:3.5.1.11] | Metabolism | |
| K01438 | 28 | acetylornithine deacetylase [EC:3.5.1.16] | Metabolism | |
| K01439 | 82 | succinyl-diaminopimelate desuccinylase [EC:3.5.1.18] | Metabolism | |
| K01443 | 68 | N-acetylglucosamine-6-phosphate deacetylase [EC:3.5.1.25] | Metabolism | |
| K01444 | 9 | N4-(beta-N-acetylglucosaminyl)-L-asparaginase [EC:3.5.1.26] | Metabolism | |
| K01451 | 45 | hippurate hydrolase [EC:3.5.1.32] | Metabolism | |
| K01452 | 38 | chitin deacetylase [EC:3.5.1.41] | Metabolism | |

| | | | | |
|---|---|---|---|---|
| K01453 | 19 | Unclassified; E3.5.1.46 | Metabolism | |
| K01455 | 26 | formamidase [EC:3.5.1.49] | Metabolism | |
| K01457 | 9 | allophanate hydrolase [EC:3.5.1.54] | Metabolism | |
| K01458 | 6 | N-formylglutamate deformylase [EC:3.5.1.68] | Metabolism | |
| K01464 | 42 | dihydropyrimidinase [EC:3.5.2.2] | Metabolism | |
| K01465 | 147 | dihydroorotase [EC:3.5.2.3] | Metabolism | |
| K01466 | 28 | allantoinase [EC:3.5.2.5] | Metabolism | |
| K01468 | 45 | imidazolonepropionase [EC:3.5.2.7] | Metabolism | |
| K01469 | 35 | 5-oxoprolinase (ATP-hydrolysing) [EC:3.5.2.9] | Metabolism | |
| K01470 | 24 | creatinine amidohydrolase [EC:3.5.2.10] | Metabolism | |
| K01471 | 12 | Unclassified; E3.5.2.12 | Metabolism | |
| K01473 | 68 | N-methylhydantoinase A [EC:3.5.2.14] | Metabolism | |
| K01474 | 76 | N-methylhydantoinase B [EC:3.5.2.14] | Metabolism | |
| K01476 | 13 | arginase [EC:3.5.3.1] | Metabolism | |
| K01477 | 8 | allantoicase [EC:3.5.3.4] | Metabolism | |
| K01478 | 10 | arginine deiminase [EC:3.5.3.6] | Metabolism | |
| K01479 | 4 | formiminoglutamase [EC:3.5.3.8] | Metabolism | |
| K01480 | 40 | agmatinase [EC:3.5.3.11] | Metabolism | |
| K01483 | 1 | ureidoglycolate hydrolase [EC:3.5.3.19] | Metabolism | |
| K01484 | 1 | succinylarginine dihydrolase [EC:3.5.3.23] | Metabolism | |
| K01485 | 25 | cytosine deaminase [EC:3.5.4.1] | Metabolism | |
| K01486 | 15 | adenine deaminase [EC:3.5.4.2] | Metabolism | |
| K01487 | 12 | guanine deaminase [EC:3.5.4.3] | Metabolism | |
| K01488 | 35 | adenosine deaminase [EC:3.5.4.4] | Metabolism | |
| K01489 | 21 | cytidine deaminase [EC:3.5.4.5] | Metabolism | |
| K01491 | 34 | methylenetetrahydrofolate dehydrogenase (NADP+) / | Metabolism | |
| K01492 | 2 | phosphoribosylaminoimidazolecarboxamide formyltransferase | Metabolism | |
| K01493 | 13 | dCMP deaminase [EC:3.5.4.12] | Metabolism | |
| K01494 | 20 | dCTP deaminase [EC:3.5.4.13] | Metabolism | |
| K01495 | 28 | GTP cyclohydrolase I [EC:3.5.4.16] | Metabolism | |
| K01496 | 6 | phosphoribosyl-AMP cyclohydrolase [EC:3.5.4.19] | Metabolism | |
| K01497 | 24 | GTP cyclohydrolase II [EC:3.5.4.25] | Metabolism | |
| K01498 | 1 | diaminohydroxyphosphoribosylaminopyrimidine deaminase [EC:3.5.4.26] | Metabolism | |
| K01499 | 3 | methenyltetrahydromethanopterin cyclohydrolase [EC:3.5.4.27] | Metabolism | |
| K01501 | 28 | nitrilase [EC:3.5.5.1] | Metabolism | |
| K01502 | 12 | aliphatic nitrilase [EC:3.5.5.7] | Metabolism | |
| K01505 | 4 | 1-aminocyclopropane-1-carboxylate deaminase [EC:3.5.99.7] | Metabolism | |
| K01507 | 54 | inorganic pyrophosphatase [EC:3.6.1.1] | Metabolism | |
| K01512 | 16 | acylphosphatase [EC:3.6.1.7] | Metabolism | |
| K01514 | 57 | exopolyphosphatase [EC:3.6.1.11] | Metabolism | |
| K01515 | 41 | ADP-ribose pyrophosphatase [EC:3.6.1.13] | Metabolism | |
| K01518 | 13 | bis(5'-nucleosidyl)-tetraphosphatase [EC:3.6.1.17] | Metabolism | |
| K01520 | 14 | dUTP pyrophosphatase [EC:3.6.1.23] | Metabolism | |
| K01523 | 4 | phosphoribosyl-ATP pyrophosphohydrolase [EC:3.6.1.31] | Metabolism | |
| K01524 | 18 | guanosine-5'-triphosphate,3'-diphosphate pyrophosphatase | Metabolism | |
| K01525 | 13 | bis(5'-nucleosyl)-tetraphosphatase (symmetrical) [EC:3.6.1.41] | Metabolism | |
| K01535 | 2 | H+-transporting ATPase [EC:3.6.3.6] | Metabolism | |
| K01555 | 12 | fumarylacetoacetase [EC:3.7.1.2] | Metabolism | |
| K01556 | 30 | kynureninase [EC:3.7.1.3] | Metabolism | |
| K01557 | 5 | acylpyruvate hydrolase [EC:3.7.1.5] | Metabolism | |
| K01560 | 34 | 2-haloacid dehalogenase [EC:3.8.1.2] | Metabolism | |
| K01561 | 20 | haloacetate dehalogenase [EC:3.8.1.3] | Metabolism | |
| K01563 | 44 | haloalkane dehalogenase [EC:3.8.1.5] | Metabolism | |
| K01568 | 1 | pyruvate decarboxylase [EC:4.1.1.1] | Metabolism | |
| K01571 | 11 | oxaloacetate decarboxylase, alpha subunit [EC:4.1.1.3] | Metabolism | |
| K01572 | 9 | oxaloacetate decarboxylase, beta subunit [EC:4.1.1.3] | Metabolism | |
| K01576 | 25 | benzoylformate decarboxylase [EC:4.1.1.7] | Metabolism | |

| | | | | |
|---|---|---|---|---|
| K01577 | 9 | oxalyl-CoA decarboxylase [EC:4.1.1.8] | Metabolism | |
| K01578 | 19 | malonyl-CoA decarboxylase [EC:4.1.1.9] | Metabolism | |
| K01579 | 12 | aspartate 1-decarboxylase [EC:4.1.1.11] | Metabolism | |
| K01580 | 8 | glutamate decarboxylase [EC:4.1.1.15] | Metabolism | |
| K01581 | 17 | ornithine decarboxylase [EC:4.1.1.17] | Metabolism | |
| K01582 | 22 | lysine decarboxylase [EC:4.1.1.18] | Metabolism | |
| K01583 | 17 | arginine decarboxylase [EC:4.1.1.19] | Metabolism | |
| K01584 | 17 | arginine decarboxylase [EC:4.1.1.19] | Metabolism | |
| K01585 | 30 | arginine decarboxylase [EC:4.1.1.19] | Metabolism | |
| K01586 | 61 | diaminopimelate decarboxylase [EC:4.1.1.20] | Metabolism | |
| K01588 | 20 | 5-(carboxyamino)imidazole ribonucleotide mutase [EC:5.4.99.18] | Metabolism | |
| K01589 | 16 | 5-(carboxyamino)imidazole ribonucleotide synthase [EC:6.3.4.18] | Metabolism | |
| K01591 | 30 | orotidine-5'-phosphate decarboxylase [EC:4.1.1.23] | Metabolism | |
| K01592 | 14 | tyrosine decarboxylase [EC:4.1.1.25] | Metabolism | |
| K01593 | 61 | aromatic-L-amino-acid decarboxylase [EC:4.1.1.28] | Metabolism | |
| K01594 | 14 | sulfinoalanine decarboxylase [EC:4.1.1.29] | Metabolism | |
| K01595 | 40 | phosphoenolpyruvate carboxylase [EC:4.1.1.31] | Metabolism | |
| K01596 | 30 | phosphoenolpyruvate carboxykinase (GTP) [EC:4.1.1.32] | Metabolism | |
| K01598 | 9 | phosphopantothenoylcysteine decarboxylase [EC:4.1.1.36] | Metabolism | |
| K01599 | 38 | uroporphyrinogen decarboxylase [EC:4.1.1.37] | Metabolism | |
| K01601 | 7 | ribulose-bisphosphate carboxylase large chain [EC:4.1.1.39] | Metabolism | |
| K01602 | 3 | ribulose-bisphosphate carboxylase small chain [EC:4.1.1.39] | Metabolism | |
| K01607 | 55 | 4-carboxymuconolactone decarboxylase [EC:4.1.1.44] | Metabolism | |
| K01608 | 8 | tartronate-semialdehyde synthase [EC:4.1.1.47] | Metabolism | |
| K01609 | 19 | indole-3-glycerol phosphate synthase [EC:4.1.1.48] | Metabolism | |
| K01610 | 22 | phosphoenolpyruvate carboxykinase (ATP) [EC:4.1.1.49] | Metabolism | |
| K01611 | 4 | S-adenosylmethionine decarboxylase [EC:4.1.1.50] | Metabolism | |
| K01612 | 5 | 4-hydroxybenzoate decarboxylase [EC:4.1.1.61] | Metabolism | |
| K01613 | 14 | phosphatidylserine decarboxylase [EC:4.1.1.65] | Metabolism | |
| K01615 | 12 | glutaconyl-CoA decarboxylase [EC:4.1.1.70] | Metabolism | |
| K01617 | 5 | 4-oxalocrotonate decarboxylase [EC:4.1.1.77] | Metabolism | |
| K01619 | 13 | deoxyribose-phosphate aldolase [EC:4.1.2.4] | Metabolism | |
| K01620 | 32 | threonine aldolase [EC:4.1.2.5] | Metabolism | |
| K01621 | 20 | phosphoketolase [EC:4.1.2.9] | Metabolism | |
| K01623 | 28 | fructose-bisphosphate aldolase, class I [EC:4.1.2.13] | Metabolism | |
| K01624 | 8 | fructose-bisphosphate aldolase, class II [EC:4.1.2.13] | Metabolism | |
| K01625 | 11 | 2-dehydro-3-deoxyphosphogluconate aldolase / | Metabolism | |
| K01626 | 26 | 3-deoxy-7-phosphoheptulonate synthase [EC:2.5.1.54] | Metabolism | |
| K01627 | 29 | 2-dehydro-3-deoxyphosphooctonate aldolase (KDO 8-P synthase) | Metabolism | |
| K01628 | 51 | L-fuculose-phosphate aldolase [EC:4.1.2.17] | Metabolism | |
| K01629 | 6 | rhamnulose-1-phosphate aldolase [EC:4.1.2.19] | Metabolism | |
| K01630 | 13 | 2-dehydro-3-deoxyglucarate aldolase [EC:4.1.2.20] | Metabolism | |
| K01631 | 11 | 2-dehydro-3-deoxyphosphogalactonate aldolase [EC:4.1.2.21] | Metabolism | |
| K01633 | 13 | dihydroneopterin aldolase [EC:4.1.2.25] | Metabolism | |
| K01634 | 7 | sphinganine-1-phosphate aldolase [EC:4.1.2.27] | Metabolism | |
| K01635 | 10 | tagatose 1,6-diphosphate aldolase [EC:4.1.2.40] | Metabolism | |
| K01637 | 13 | isocitrate lyase [EC:4.1.3.1] | Metabolism | |
| K01638 | 23 | malate synthase [EC:2.3.3.9] | Metabolism | |
| K01639 | 16 | N-acetylneuraminate lyase [EC:4.1.3.3] | Metabolism | |
| K01640 | 23 | hydroxymethylglutaryl-CoA lyase [EC:4.1.3.4] | Metabolism | |
| K01641 | 3 | hydroxymethylglutaryl-CoA synthase [EC:2.3.3.10] | Metabolism | |
| K01647 | 50 | citrate synthase [EC:2.3.3.1] | Metabolism | |
| K01648 | 1 | ATP citrate (pro-S)-lyase [EC:2.3.3.8] | Metabolism | |
| K01649 | 82 | 2-isopropylmalate synthase [EC:2.3.3.13] | Metabolism | |
| K01652 | 178 | acetolactate synthase I/II/III large subunit [EC:2.2.1.6] | Metabolism | |
| K01653 | 22 | acetolactate synthase I/III small subunit [EC:2.2.1.6] | Metabolism | |
| K01654 | 14 | N-acetylneuraminate synthase [EC:2.5.1.56] | Metabolism | |

| | | | | |
|---|---|---|---|---|
| K01655 | 8 | homocitrate synthase [EC:2.3.3.14] | Metabolism | |
| K01657 | 59 | anthranilate synthase component I [EC:4.1.3.27] | Metabolism | |
| K01658 | 28 | anthranilate synthase component II [EC:4.1.3.27] | Metabolism | |
| K01659 | 6 | 2-methylcitrate synthase [EC:2.3.3.5] | Metabolism | |
| K01661 | 17 | naphthoate synthase [EC:4.1.3.36] | Metabolism | |
| K01662 | 55 | 1-deoxy-D-xylulose-5-phosphate synthase [EC:2.2.1.7] | Metabolism | |
| K01664 | 14 | para-aminobenzoate synthetase component II [EC:2.6.1.85] | Metabolism | |
| K01665 | 22 | para-aminobenzoate synthetase component I [EC:2.6.1.85] | Metabolism | |
| K01666 | 4 | 4-hydroxy 2-oxovalerate aldolase [EC:4.1.3.39] | Metabolism | |
| K01667 | 15 | tryptophanase [EC:4.1.99.1] | Metabolism | |
| K01668 | 12 | tyrosine phenol-lyase [EC:4.1.99.2] | Metabolism | |
| K01673 | 27 | carbonic anhydrase [EC:4.2.1.1] | Metabolism | |
| K01676 | 23 | fumarate hydratase, class I [EC:4.2.1.2] | Metabolism | |
| K01677 | 4 | fumarate hydratase subunit alpha [EC:4.2.1.2] | Metabolism | |
| K01679 | 37 | fumarate hydratase, class II [EC:4.2.1.2] | Metabolism | |
| K01681 | 81 | aconitate hydratase 1 [EC:4.2.1.3] | Metabolism | |
| K01682 | 7 | aconitate hydratase 2 [EC:4.2.1.3] | Metabolism | |
| K01684 | 33 | galactonate dehydratase [EC:4.2.1.6] | Metabolism | |
| K01685 | 24 | altronate hydrolase [EC:4.2.1.7] | Metabolism | |
| K01686 | 9 | mannonate dehydratase [EC:4.2.1.8] | Metabolism | |
| K01687 | 73 | dihydroxy-acid dehydratase [EC:4.2.1.9] | Metabolism | |
| K01689 | 42 | enolase [EC:4.2.1.11] | Metabolism | |
| K01690 | 2 | phosphogluconate dehydratase [EC:4.2.1.12] | Metabolism | |
| K01692 | 266 | enoyl-CoA hydratase [EC:4.2.1.17] | Metabolism | |
| K01693 | 14 | imidazoleglycerol-phosphate dehydratase [EC:4.2.1.19] | Metabolism | |
| K01695 | 29 | tryptophan synthase alpha chain [EC:4.2.1.20] | Metabolism | |
| K01696 | 29 | tryptophan synthase beta chain [EC:4.2.1.20] | Metabolism | |
| K01697 | 25 | cystathionine beta-synthase [EC:4.2.1.22] | Metabolism | |
| K01698 | 18 | porphobilinogen synthase [EC:4.2.1.24] | Metabolism | |
| K01699 | 4 | propanediol dehydratase large subunit [EC:4.2.1.28] | Metabolism | |
| K01703 | 59 | 3-isopropylmalate/(R)-2-methylmalate dehydratase large subunit | Metabolism | |
| K01704 | 24 | 3-isopropylmalate/(R)-2-methylmalate dehydratase small subunit | Metabolism | |
| K01705 | 1 | homoaconitate hydratase [EC:4.2.1.36] | Metabolism | |
| K01706 | 7 | glucarate dehydratase [EC:4.2.1.40] | Metabolism | |
| K01708 | 22 | galactarate dehydratase [EC:4.2.1.42] | Metabolism | |
| K01709 | 15 | CDP-glucose 4,6-dehydratase [EC:4.2.1.45] | Metabolism | |
| K01710 | 121 | dTDP-glucose 4,6-dehydratase [EC:4.2.1.46] | Metabolism | |
| K01711 | 34 | GDPmannose 4,6-dehydratase [EC:4.2.1.47] | Metabolism | |
| K01712 | 17 | urocanate hydratase [EC:4.2.1.49] | Metabolism | |
| K01713 | 19 | prephenate dehydratase [EC:4.2.1.51] | Metabolism | |
| K01714 | 67 | dihydrodipicolinate synthase [EC:4.2.1.52] | Metabolism | |
| K01715 | 61 | 3-hydroxybutyryl-CoA dehydratase [EC:4.2.1.55] | Metabolism | |
| K01716 | 1 | 3-hydroxydecanoyl-[acyl-carrier-protein] dehydratase [EC:4.2.1.60] | Metabolism | |
| K01719 | 22 | uroporphyrinogen-III synthase [EC:4.2.1.75] | Metabolism | |
| K01720 | 63 | 2-methylcitrate dehydratase [EC:4.2.1.79] | Metabolism | |
| K01725 | 1 | cyanate lyase [EC:4.2.1.104] | Metabolism | |
| K01728 | 1 | pectate lyase [EC:4.2.2.2] | Metabolism | |
| K01733 | 66 | threonine synthase [EC:4.2.3.1] | Metabolism | |
| K01734 | 6 | methylglyoxal synthase [EC:4.2.3.3] | Metabolism | |
| K01735 | 30 | 3-dehydroquinate synthase [EC:4.2.3.4] | Metabolism | |
| K01736 | 40 | chorismate synthase [EC:4.2.3.5] | Metabolism | |
| K01737 | 25 | 6-pyruvoyl tetrahydrobiopterin synthase [EC:4.2.3.12] | Metabolism | |
| K01738 | 73 | cysteine synthase A [EC:2.5.1.47] | Metabolism | |
| K01739 | 49 | cystathionine gamma-synthase [EC:2.5.1.48] | Metabolism | |
| K01740 | 36 | O-acetylhomoserine (thiol)-lyase [EC:2.5.1.49] | Metabolism | |
| K01744 | 18 | aspartate ammonia-lyase [EC:4.3.1.1] | Metabolism | |
| K01745 | 32 | histidine ammonia-lyase [EC:4.3.1.3] | Metabolism | |

| | | | | |
|---|---|---|---|---|
| K01746 | 14 | formiminotetrahydrofolate cyclodeaminase [EC:4.3.1.4] | Metabolism | |
| K01749 | 28 | hydroxymethylbilane synthase [EC:2.5.1.61] | Metabolism | |
| K01750 | 39 | ornithine cyclodeaminase [EC:4.3.1.12] | Metabolism | |
| K01752 | 8 | L-serine dehydratase [EC:4.3.1.17] | Metabolism | |
| K01754 | 80 | threonine dehydratase [EC:4.3.1.19] | Metabolism | |
| K01755 | 59 | argininosuccinate lyase [EC:4.3.2.1] | Metabolism | |
| K01756 | 53 | adenylosuccinate lyase [EC:4.3.2.2] | Metabolism | |
| K01757 | 6 | strictosidine synthase [EC:4.3.3.2] | Metabolism | |
| K01758 | 28 | cystathionine gamma-lyase [EC:4.4.1.1] | Metabolism | |
| K01759 | 62 | lactoylglutathione lyase [EC:4.4.1.5] | Metabolism | |
| K01760 | 59 | cystathionine beta-lyase [EC:4.4.1.8] | Metabolism | |
| K01761 | 21 | methionine-gamma-lyase [EC:4.4.1.11] | Metabolism | |
| K01768 | 403 | adenylate cyclase [EC:4.6.1.1] | Metabolism | |
| K01769 | 7 | guanylate cyclase, other [EC:4.6.1.2] | Metabolism | |
| K01770 | 23 | 2-C-methyl-D-erythritol 2,4-cyclodiphosphate synthase [EC:4.6.1.12] | Metabolism | |
| K01772 | 32 | ferrochelatase [EC:4.99.1.1] | Metabolism | |
| K01775 | 45 | alanine racemase [EC:5.1.1.1] | Metabolism | |
| K01776 | 47 | glutamate racemase [EC:5.1.1.3] | Metabolism | |
| K01777 | 12 | proline racemase [EC:5.1.1.4] | Metabolism | |
| K01778 | 33 | diaminopimelate epimerase [EC:5.1.1.7] | Metabolism | |
| K01779 | 30 | aspartate racemase [EC:5.1.1.13] | Metabolism | |
| K01781 | 68 | mandelate racemase [EC:5.1.2.2] | Metabolism | |
| K01782 | 36 | 3-hydroxyacyl-CoA dehydrogenase / enoyl-CoA hydratase / | Metabolism | |
| K01783 | 21 | ribulose-phosphate 3-epimerase [EC:5.1.3.1] | Metabolism | |
| K01784 | 233 | UDP-glucose 4-epimerase [EC:5.1.3.2] | Metabolism | |
| K01785 | 23 | aldose 1-epimerase [EC:5.1.3.3] | Metabolism | |
| K01786 | 5 | L-ribulose-5-phosphate 4-epimerase [EC:5.1.3.4] | Metabolism | |
| K01787 | 7 | N-acylglucosamine 2-epimerase [EC:5.1.3.8] | Metabolism | |
| K01790 | 33 | dTDP-4-dehydrorhamnose 3,5-epimerase [EC:5.1.3.13] | Metabolism | |
| K01791 | 43 | UDP-N-acetylglucosamine 2-epimerase [EC:5.1.3.14] | Metabolism | |
| K01792 | 3 | glucose-6-phosphate 1-epimerase [EC:5.1.3.15] | Metabolism | |
| K01795 | 128 | Unclassified; E5.1.3.- | Metabolism | |
| K01796 | 35 | alpha-methylacyl-CoA racemase [EC:5.1.99.4] | Metabolism | |
| K01799 | 14 | maleate isomerase [EC:5.2.1.1] | Metabolism | |
| K01800 | 11 | maleylacetoacetate isomerase [EC:5.2.1.2] | Metabolism | |
| K01801 | 8 | maleylpyruvate isomerase [EC:5.2.1.4] | Metabolism | |
| K01803 | 29 | triosephosphate isomerase (TIM) [EC:5.3.1.1] | Metabolism | |
| K01804 | 10 | L-arabinose isomerase [EC:5.3.1.4] | Metabolism | |
| K01805 | 13 | xylose isomerase [EC:5.3.1.5] | Metabolism | |
| K01807 | 12 | ribose 5-phosphate isomerase A [EC:5.3.1.6] | Metabolism | |
| K01808 | 26 | ribose 5-phosphate isomerase B [EC:5.3.1.6] | Metabolism | |
| K01809 | 44 | mannose-6-phosphate isomerase [EC:5.3.1.8] | Metabolism | |
| K01810 | 53 | glucose-6-phosphate isomerase [EC:5.3.1.9] | Metabolism | |
| K01812 | 7 | glucuronate isomerase [EC:5.3.1.12] | Metabolism | |
| K01813 | 9 | L-rhamnose isomerase [EC:5.3.1.14] | Metabolism | |
| K01814 | 30 | phosphoribosylformimino-5-aminoimidazole carboxamide ribotide | Metabolism | |
| K01816 | 12 | hydroxypyruvate isomerase [EC:5.3.1.22] | Metabolism | |
| K01817 | 27 | phosphoribosylanthranilate isomerase [EC:5.3.1.24] | Metabolism | |
| K01818 | 6 | L-fucose isomerase [EC:5.3.1.25] | Metabolism | |
| K01820 | 9 | Unclassified; E5.3.1.- | Metabolism | |
| K01821 | 5 | 4-oxalocrotonate tautomerase [EC:5.3.2.-] | Metabolism | |
| K01822 | 1 | steroid delta-isomerase [EC:5.3.3.1] | Metabolism | |
| K01823 | 12 | isopentenyl-diphosphate delta-isomerase [EC:5.3.3.2] | Metabolism | |
| K01825 | 4 | 3-hydroxyacyl-CoA dehydrogenase / enoyl-CoA hydratase / | Metabolism | |
| K01826 | 20 | 5-carboxymethyl-2-hydroxymuconate isomerase [EC:5.3.3.10] | Metabolism | |
| K01834 | 100 | phosphoglycerate mutase [EC:5.4.2.1] | Metabolism | |
| K01835 | 41 | phosphoglucomutase [EC:5.4.2.2] | Metabolism | |

| | | | | |
|---|---|---|---|---|
| K01838 | 4 | beta-phosphoglucomutase [EC:5.4.2.6] | Metabolism | |
| K01839 | 18 | phosphopentomutase [EC:5.4.2.7] | Metabolism | |
| K01840 | 87 | phosphomannomutase [EC:5.4.2.8] | Metabolism | |
| K01841 | 3 | phosphoenolpyruvate phosphomutase [EC:5.4.2.9] | Metabolism | |
| K01843 | 25 | lysine 2,3-aminomutase [EC:5.4.3.2] | Metabolism | |
| K01844 | 3 | beta-lysine 5,6-aminomutase [EC:5.4.3.3] | Metabolism | |
| K01845 | 65 | glutamate-1-semialdehyde 2,1-aminomutase [EC:5.4.3.8] | Metabolism | |
| K01846 | 4 | methylaspartate mutase [EC:5.4.99.1] | Metabolism | |
| K01847 | 14 | methylmalonyl-CoA mutase [EC:5.4.99.2] | Metabolism | |
| K01848 | 52 | methylmalonyl-CoA mutase, N-terminal domain [EC:5.4.99.2] | Metabolism | |
| K01849 | 12 | methylmalonyl-CoA mutase, C-terminal domain [EC:5.4.99.2] | Metabolism | |
| K01851 | 1 | salicylate biosynthesis isochorismate synthase [EC:5.4.4.2] | Metabolism | |
| K01854 | 46 | UDP-galactopyranose mutase [EC:5.4.99.9] | Metabolism | |
| K01856 | 36 | muconate cycloisomerase [EC:5.5.1.1] | Metabolism | |
| K01857 | 15 | 3-carboxy-cis,cis-muconate cycloisomerase [EC:5.5.1.2] | Metabolism | |
| K01858 | 12 | myo-inositol-1-phosphate synthase [EC:5.5.1.4] | Metabolism | |
| K01860 | 15 | chloromuconate cycloisomerase [EC:5.5.1.7] | Metabolism | |
| K01874 | 56 | methionyl-tRNA synthetase [EC:6.1.1.10] | Metabolism | |
| K01885 | 74 | glutamyl-tRNA synthetase [EC:6.1.1.17] | Metabolism | |
| K01895 | 175 | acetyl-CoA synthetase [EC:6.2.1.1] | Metabolism | |
| K01897 | 294 | long-chain acyl-CoA synthetase [EC:6.2.1.3] | Metabolism | |
| K01902 | 19 | succinyl-CoA synthetase alpha subunit [EC:6.2.1.5] | Metabolism | |
| K01903 | 30 | succinyl-CoA synthetase beta subunit [EC:6.2.1.5] | Metabolism | |
| K01904 | 37 | 4-coumarate--CoA ligase [EC:6.2.1.12] | Metabolism | |
| K01905 | 37 | acetyl-CoA synthetase (ADP-forming) [EC:6.2.1.13] | Metabolism | |
| K01906 | 43 | 6-carboxyhexanoate--CoA ligase [EC:6.2.1.14] | Metabolism | |
| K01907 | 38 | acetoacetyl-CoA synthetase [EC:6.2.1.16] | Metabolism | |
| K01908 | 12 | propionyl-CoA synthetase [EC:6.2.1.17] | Metabolism | |
| K01911 | 22 | O-succinylbenzoic acid--CoA ligase [EC:6.2.1.26] | Metabolism | |
| K01912 | 88 | phenylacetate-CoA ligase [EC:6.2.1.30] | Metabolism | |
| K01914 | 7 | aspartate--ammonia ligase [EC:6.3.1.1] | Metabolism | |
| K01915 | 141 | glutamine synthetase [EC:6.3.1.2] | Metabolism | |
| K01916 | 33 | NAD+ synthase [EC:6.3.1.5] | Metabolism | |
| K01917 | 15 | glutathionylspermidine synthase [EC:6.3.1.8] | Metabolism | |
| K01918 | 21 | pantoate--beta-alanine ligase [EC:6.3.2.1] | Metabolism | |
| K01919 | 16 | glutamate--cysteine ligase [EC:6.3.2.2] | Metabolism | |
| K01920 | 20 | glutathione synthase [EC:6.3.2.3] | Metabolism | |
| K01921 | 88 | D-alanine-D-alanine ligase [EC:6.3.2.4] | Metabolism | |
| K01922 | 6 | phosphopantothenate-cysteine ligase [EC:6.3.2.5] | Metabolism | |
| K01923 | 35 | phosphoribosylaminoimidazole-succinocarboxamide synthase | Metabolism | |
| K01924 | 35 | UDP-N-acetylmuramate--alanine ligase [EC:6.3.2.8] | Metabolism | |
| K01925 | 33 | UDP-N-acetylmuramoylalanine--D-glutamate ligase [EC:6.3.2.9] | Metabolism | |
| K01928 | 42 | UDP-N-acetylmuramoylalanyl-D-glutamate--2,6-diaminopimelate ligase | Metabolism | |
| K01929 | 58 | UDP-N-acetylmuramoylalanyl-D-glutamyl-2,6-diaminopimelate--D-alanyl- | Metabolism | |
| K01933 | 32 | phosphoribosylformylglycinamidine cyclo-ligase [EC:6.3.3.1] | Metabolism | |
| K01934 | 15 | 5-formyltetrahydrofolate cyclo-ligase [EC:6.3.3.2] | Metabolism | |
| K01935 | 11 | dethiobiotin synthetase [EC:6.3.3.3] | Metabolism | |
| K01937 | 42 | CTP synthase [EC:6.3.4.2] | Metabolism | |
| K01938 | 23 | formate--tetrahydrofolate ligase [EC:6.3.4.3] | Metabolism | |
| K01939 | 47 | adenylosuccinate synthase [EC:6.3.4.4] | Metabolism | |
| K01940 | 34 | argininosuccinate synthase [EC:6.3.4.5] | Metabolism | |
| K01941 | 8 | urea carboxylase [EC:6.3.4.6] | Metabolism | |
| K01945 | 31 | phosphoribosylamine--glycine ligase [EC:6.3.4.13] | Metabolism | |
| K01947 | 4 | biotin-[acetyl-CoA-carboxylase] ligase / type III pantothenate | Metabolism | |
| K01950 | 63 | NAD+ synthase (glutamine-hydrolysing) [EC:6.3.5.1] | Metabolism | |
| K01951 | 50 | GMP synthase (glutamine-hydrolysing) [EC:6.3.5.2] | Metabolism | |
| K01952 | 89 | phosphoribosylformylglycinamidine synthase [EC:6.3.5.3] | Metabolism | |

| | | | | |
|---|---|---|---|---|
| K01953 | 160 | asparagine synthase (glutamine-hydrolysing) [EC:6.3.5.4] | Metabolism | |
| K01955 | 66 | carbamoyl-phosphate synthase large subunit [EC:6.3.5.5] | Metabolism | |
| K01956 | 39 | carbamoyl-phosphate synthase small subunit [EC:6.3.5.5] | Metabolism | |
| K01958 | 22 | pyruvate carboxylase [EC:6.4.1.1] | Metabolism | |
| K01959 | 2 | pyruvate carboxylase subunit A [EC:6.4.1.1] | Metabolism | |
| K01960 | 23 | pyruvate carboxylase subunit B [EC:6.4.1.1] | Metabolism | |
| K01961 | 38 | acetyl-CoA carboxylase, biotin carboxylase subunit [EC:6.4.1.2 | Metabolism | |
| K01962 | 21 | acetyl-CoA carboxylase carboxyl transferase subunit alpha | Metabolism | |
| K01963 | 31 | acetyl-CoA carboxylase carboxyl transferase subunit beta | Metabolism | |
| K01965 | 36 | propionyl-CoA carboxylase alpha chain [EC:6.4.1.3] | Metabolism | |
| K01966 | 69 | propionyl-CoA carboxylase beta chain [EC:6.4.1.3] | Metabolism | |
| K01968 | 21 | 3-methylcrotonyl-CoA carboxylase alpha subunit [EC:6.4.1.4] | Metabolism | |
| K01969 | 19 | 3-methylcrotonyl-CoA carboxylase beta subunit [EC:6.4.1.4] | Metabolism | |
| K02045 | 11 | sulfate transport system ATP-binding protein [EC:3.6.3.25] | Metabolism | |
| K02046 | 14 | sulfate transport system permease protein | Metabolism | |
| K02047 | 9 | sulfate transport system permease protein | Metabolism | |
| K02048 | 16 | sulfate transport system substrate-binding protein | Metabolism | |
| K02079 | 2 | N-acetylgalactosamine-6-phosphate deacetylase [EC:3.5.1.25] | Metabolism | |
| K02083 | 5 | allantoate deiminase [EC:3.5.3.9] | Metabolism | |
| K02108 | 50 | F-type H+-transporting ATPase subunit a [EC:3.6.3.14] | Metabolism | |
| K02109 | 12 | F-type H+-transporting ATPase subunit b [EC:3.6.3.14] | Metabolism | |
| K02110 | 10 | F-type H+-transporting ATPase subunit c [EC:3.6.3.14] | Metabolism | |
| K02111 | 32 | F-type H+-transporting ATPase subunit alpha [EC:3.6.3.14] | Metabolism | |
| K02112 | 21 | F-type H+-transporting ATPase subunit beta [EC:3.6.3.14] | Metabolism | |
| K02113 | 26 | F-type H+-transporting ATPase subunit delta [EC:3.6.3.14] | Metabolism | |
| K02114 | 7 | F-type H+-transporting ATPase subunit epsilon [EC:3.6.3.14] | Metabolism | |
| K02115 | 35 | F-type H+-transporting ATPase subunit gamma [EC:3.6.3.14] | Metabolism | |
| K02117 | 20 | V-type H+-transporting ATPase subunit A [EC:3.6.3.14] | Metabolism | |
| K02118 | 13 | V-type H+-transporting ATPase subunit B [EC:3.6.3.14] | Metabolism | |
| K02120 | 3 | V-type H+-transporting ATPase subunit D [EC:3.6.3.14] | Metabolism | |
| K02121 | 2 | V-type H+-transporting ATPase subunit E [EC:3.6.3.14] | Metabolism | |
| K02123 | 15 | V-type H+-transporting ATPase subunit I [EC:3.6.3.14] | Metabolism | |
| K02124 | 4 | V-type H+-transporting ATPase subunit K [EC:3.6.3.14] | Metabolism | |
| K02160 | 11 | acetyl-CoA carboxylase biotin carboxyl carrier protein | Metabolism | |
| K02169 | 17 | biotin synthesis protein BioC | Metabolism | |
| K02170 | 4 | biotin biosynthesis protein BioH | Metabolism | |
| K02188 | 4 | cobalamin biosynthesis protein CbiD | Metabolism | |
| K02189 | 1 | cobalamin biosynthesis protein CbiG | Metabolism | |
| K02203 | 6 | phosphoserine / homoserine phosphotransferase [EC:3.1.3.3 2.7.1.39] | Metabolism | |
| K02204 | 5 | homoserine kinase type II [EC:2.7.1.39] | Metabolism | |
| K02224 | 18 | cobyrinic acid a,c-diamide synthase [EC:6.3.5.9 6.3.1.-] | Metabolism | |
| K02226 | 2 | alpha-ribazole phosphatase [EC:3.1.3.73] | Metabolism | |
| K02227 | 11 | adenosylcobinamide-phosphate synthase CobD [EC:6.3.1.10] | Metabolism | |
| K02229 | 1 | precorrin-3B synthase [EC:1.14.13.83] | Metabolism | |
| K02230 | 13 | cobaltochelatase CobN [EC:6.6.1.2] | Metabolism | |
| K02231 | 13 | adenosylcobinamide kinase / adenosylcobinamide-phosphate | Metabolism | |
| K02232 | 9 | adenosylcobyric acid synthase [EC:6.3.5.10] | Metabolism | |
| K02233 | 2 | adenosylcobinamide-GDP ribazoletransferase [EC:2.7.8.26] | Metabolism | |
| K02234 | 13 | cobalamin biosynthesis protein CobW | Metabolism | |
| K02258 | 6 | cytochrome c oxidase subunit XI assembly protein | Metabolism | |
| K02259 | 27 | cytochrome c oxidase subunit XV assembly protein | Metabolism | |
| K02274 | 88 | cytochrome c oxidase subunit I [EC:1.9.3.1] | Metabolism | |
| K02275 | 66 | cytochrome c oxidase subunit II [EC:1.9.3.1] | Metabolism | |
| K02276 | 37 | cytochrome c oxidase subunit III [EC:1.9.3.1] | Metabolism | |
| K02287 | 2 | phycocyanin-associated, rod | Metabolism | |
| K02288 | 2 | phycocyanobilin lyase alpha subunit [EC:4.-.-.-] | Metabolism | |
| K02291 | 30 | phytoene synthase [EC:2.5.1.32] | Metabolism | |

| | | | | |
|---|---|---|---|---|
| K02292 | 50 | beta-carotene ketolase (CrtO type) | Metabolism | |
| K02293 | 3 | phytoene dehydrogenase, phytoene desaturase [EC:1.14.99.-] | Metabolism | |
| K02297 | 3 | cytochrome o ubiquinol oxidase subunit II [EC:1.10.3.-] | Metabolism | |
| K02298 | 6 | cytochrome o ubiquinol oxidase subunit I [EC:1.10.3.-] | Metabolism | |
| K02299 | 4 | cytochrome o ubiquinol oxidase subunit III [EC:1.10.3.-] | Metabolism | |
| K02301 | 31 | protoheme IX farnesyltransferase [EC:2.5.1.-] | Metabolism | |
| K02302 | 27 | uroporphyrin-III C-methyltransferase / precorrin-2 dehydrogenase / | Metabolism | |
| K02303 | 22 | uroporphyrin-III C-methyltransferase [EC:2.1.1.107] | Metabolism | |
| K02304 | 6 | precorrin-2 dehydrogenase / sirohydrochlorin ferrochelatase | Metabolism | |
| K02305 | 3 | nitric-oxide reductase, cytochrome c-containing subunit II | Metabolism | |
| K02319 | 11 | DNA polymerase I [EC:2.7.7.7] | Metabolism | |
| K02335 | 90 | DNA polymerase I [EC:2.7.7.7] | Metabolism | |
| K02337 | 211 | DNA polymerase III subunit alpha [EC:2.7.7.7] | Metabolism | |
| K02338 | 59 | DNA polymerase III subunit beta [EC:2.7.7.7] | Metabolism | |
| K02339 | 6 | DNA polymerase III subunit chi [EC:2.7.7.7] | Metabolism | |
| K02340 | 11 | DNA polymerase III subunit delta [EC:2.7.7.7] | Metabolism | |
| K02341 | 35 | DNA polymerase III subunit delta' [EC:2.7.7.7] | Metabolism | |
| K02342 | 60 | DNA polymerase III subunit epsilon [EC:2.7.7.7] | Metabolism | |
| K02343 | 65 | DNA polymerase III subunit gamma/tau [EC:2.7.7.7] | Metabolism | |
| K02345 | 1 | DNA polymerase III subunit theta [EC:2.7.7.7] | Metabolism | |
| K02361 | 19 | isochorismate synthase [EC:5.4.4.2] | Metabolism | |
| K02363 | 1 | enterobactin 2,3-dihydroxybenzoate-AMP ligase / | Metabolism | |
| K02364 | 7 | enterobactin synthetase component F [EC:2.7.7.-] | Metabolism | |
| K02371 | 8 | enoyl-[acyl carrier protein] reductase II [EC:1.3.1.-] | Metabolism | |
| K02372 | 22 | 3R-hydroxymyristoyl ACP dehydrase [EC:4.2.1.-] | Metabolism | |
| K02377 | 24 | GDP-L-fucose synthase [EC:1.1.1.271] | Metabolism | |
| K02428 | 30 | nucleoside-triphosphate pyrophosphatase [EC:3.6.1.19] | Metabolism | |
| K02437 | 29 | glycine cleavage system H protein | Metabolism | |
| K02438 | 33 | glycogen operon protein GlgX [EC:3.2.1.-] | Metabolism | |
| K02439 | 2 | thiosulfate sulfurtransferase [EC:2.8.1.1] | Metabolism | |
| K02446 | 28 | fructose-1,6-bisphosphatase II [EC:3.1.3.11] | Metabolism | |
| K02472 | 10 | UDP-N-acetyl-D-mannosaminuronic acid dehydrogenase [EC:1.1.1.-] | Metabolism | |
| K02473 | 35 | UDP-N-acetylglucosamine 4-epimerase [EC:5.1.3.7] | Metabolism | |
| K02474 | 11 | UDP-N-acetyl-D-galactosamine dehydrogenase [EC:1.1.1.-] | Metabolism | |
| K02492 | 42 | glutamyl-tRNA reductase [EC:1.2.1.70] | Metabolism | |
| K02495 | 53 | oxygen-independent coproporphyrinogen III oxidase [EC:1.3.99.22] | Metabolism | |
| K02496 | 3 | uroporphyrin-III C-methyltransferase [EC:2.1.1.107] | Metabolism | |
| K02500 | 27 | cyclase HisF [EC:4.1.3.-] | Metabolism | |
| K02501 | 31 | glutamine amidotransferase [EC:2.4.2.-] | Metabolism | |
| K02502 | 22 | ATP phosphoribosyltransferase regulatory subunit | Metabolism | |
| K02509 | 7 | 2-oxo-hept-3-ene-1,7-dioate hydratase [EC:4.2.1.-] | Metabolism | |
| K02510 | 35 | 2,4-dihydroxyhept-2-ene-1,7-dioic acid aldolase [EC:4.1.2.-] | Metabolism | |
| K02517 | 28 | lipid A biosynthesis lauroyl acyltransferase [EC:2.3.1.-] | Metabolism | |
| K02523 | 22 | octaprenyl diphosphate synthase [EC:2.5.1.-] | Metabolism | |
| K02527 | 16 | 3-deoxy-D-manno-octulosonic-acid transferase [EC:2.-.-.-] | Metabolism | |
| K02535 | 25 | UDP-3-O-[3-hydroxymyristoyl] N-acetylglucosamine deacetylase | Metabolism | |
| K02536 | 33 | UDP-3-O-[3-hydroxymyristoyl] glucosamine N-acyltransferase | Metabolism | |
| K02548 | 10 | 1,4-dihydroxy-2-naphthoate octaprenyltransferase [EC:2.5.1.-] | Metabolism | |
| K02549 | 20 | O-succinylbenzoate synthase [EC:4.2.1.113] | Metabolism | |
| K02551 | 32 | 2-succinyl-5-enolpyruvyl-6-hydroxy-3-cyclohexene-1-carboxylate | Metabolism | |
| K02552 | 12 | menaquinone-specific isochorismate synthase [EC:5.4.4.2] | Metabolism | |
| K02554 | 5 | 2-keto-4-pentenoate hydratase [EC:4.2.1.80] | Metabolism | |
| K02563 | 30 | UDP-N-acetylglucosamine--N-acetylmuramyl-(pentapeptide) | Metabolism | |
| K02564 | 23 | glucosamine-6-phosphate deaminase [EC:3.5.99.6] | Metabolism | |
| K02567 | 28 | periplasmic nitrate reductase NapA [EC:1.7.99.4] | Metabolism | |
| K02568 | 8 | cytochrome c-type protein NapB | Metabolism | |
| K02575 | 27 | MFS transporter, NNP family, nitrate/nitrite transporter | Metabolism | |

| | | | | |
|---|---|---|---|---|
| K02586 | 4 | nitrogenase molybdenum-iron protein alpha chain [EC:1.18.6.1] | Metabolism | |
| K02588 | 1 | nitrogenase iron protein NifH [EC:1.18.6.1] | Metabolism | |
| K02591 | 3 | nitrogenase molybdenum-iron protein beta chain [EC:1.18.6.1] | Metabolism | |
| K02594 | 3 | homocitrate synthase NifV | Metabolism | |
| K02609 | 16 | phenylacetic acid degradation protein | Metabolism | |
| K02610 | 4 | phenylacetic acid degradation protein | Metabolism | |
| K02611 | 10 | phenylacetic acid degradation protein | Metabolism | |
| K02612 | 14 | phenylacetic acid degradation protein | Metabolism | |
| K02613 | 11 | phenylacetic acid degradation NADH oxidoreductase | Metabolism | |
| K02614 | 21 | phenylacetic acid degradation protein | Metabolism | |
| K02615 | 3 | acetyl-CoA acetyltransferase [EC:2.3.1.-] | Metabolism | |
| K02618 | 19 | phenylacetic acid degradation protein | Metabolism | |
| K02619 | 44 | 4-amino-4-deoxychorismate lyase [EC:4.1.3.38] | Metabolism | |
| K02626 | 5 | arginine decarboxylase [EC:4.1.1.19] | Metabolism | |
| K02635 | 19 | cytochrome b6 | Metabolism | |
| K02636 | 14 | cytochrome b6-f complex iron-sulfur subunit [EC:1.10.99.1] | Metabolism | |
| K02637 | 1 | cytochrome b6-f complex subunit 4 | Metabolism | |
| K02638 | 2 | plastocyanin | Metabolism | |
| K02641 | 2 | ferredoxin--NADP+ reductase [EC:1.18.1.2] | Metabolism | |
| K02690 | 1 | photosystem I core protein Ib | Metabolism | |
| K02745 | 1 | PTS system, N-acetylgalactosamine-specific IIB component | Metabolism | |
| K02765 | 3 | PTS system, D-glucosamine-specific IIC component | Metabolism | |
| K02768 | 6 | PTS system, fructose-specific IIA component [EC:2.7.1.69] | Metabolism | |
| K02769 | 1 | PTS system, fructose-specific IIB component [EC:2.7.1.69] | Metabolism | |
| K02770 | 12 | PTS system, fructose-specific IIC component | Metabolism | |
| K02774 | 5 | PTS system, galactitol-specific IIB component [EC:2.7.1.69] | Metabolism | |
| K02775 | 2 | PTS system, galactitol-specific IIC component | Metabolism | |
| K02777 | 109 | PTS system, glucose-specific IIA component [EC:2.7.1.69] | Metabolism | |
| K02779 | 2 | PTS system, glucose-specific IIC component | Metabolism | |
| K02793 | 6 | PTS system, mannose-specific IIA component [EC:2.7.1.69] | Metabolism | |
| K02794 | 3 | PTS system, mannose-specific IIB component [EC:2.7.1.69] | Metabolism | |
| K02795 | 3 | PTS system, mannose-specific IIC component | Metabolism | |
| K02796 | 6 | PTS system, mannose-specific IID component | Metabolism | |
| K02799 | 2 | PTS system, mannitol-specific IIB component [EC:2.7.1.69] | Metabolism | |
| K02800 | 3 | PTS system, mannitol-specific IIC component | Metabolism | |
| K02804 | 1 | PTS system, N-acetylglucosamine-specific IIC component | Metabolism | |
| K02821 | 9 | PTS system, ascorbate-specific IIA component [EC:2.7.1.69] | Metabolism | |
| K02823 | 18 | dihydroorotate dehydrogenase electron transfer subunit | Metabolism | |
| K02825 | 13 | pyrimidine operon attenuation protein / uracil | Metabolism | |
| K02826 | 1 | quinol oxidase polypeptide II [EC:1.9.3.-] | Metabolism | |
| K02827 | 1 | quinol oxidase polypeptide I [EC:1.9.3.-] | Metabolism | |
| K02828 | 1 | quinol oxidase polypeptide III [EC:1.9.3.-] | Metabolism | |
| K02841 | 22 | heptosyltransferase I [EC:2.4.-.-] | Metabolism | |
| K02843 | 38 | heptosyltransferase II [EC:2.4.-.-] | Metabolism | |
| K02844 | 36 | UDP-glucose:(heptosyl)LPS alpha-1,3-glucosyltransferase [EC:2.4.1.-] | Metabolism | |
| K02849 | 21 | heptosyltransferase III [EC:2.4.-.-] | Metabolism | |
| K02858 | 40 | 3,4-dihydroxy 2-butanone 4-phosphate synthase [EC:4.1.99.12] | Metabolism | |
| K03040 | 34 | DNA-directed RNA polymerase subunit alpha [EC:2.7.7.6] | Metabolism | |
| K03043 | 77 | DNA-directed RNA polymerase subunit beta [EC:2.7.7.6] | Metabolism | |
| K03046 | 516 | DNA-directed RNA polymerase subunit beta' [EC:2.7.7.6] | Metabolism | |
| K03060 | 10 | DNA-directed RNA polymerase subunit omega [EC:2.7.7.6] | Metabolism | |
| K03077 | 3 | L-ribulose-5-phosphate 4-epimerase [EC:5.1.3.4] | Metabolism | |
| K03080 | 2 | L-ribulose-5-phosphate 4-epimerase [EC:5.1.3.4] | Metabolism | |
| K03119 | 43 | taurine dioxygenase [EC:1.14.11.17] | Metabolism | |
| K03146 | 1 | thiamine biosynthetic enzyme | Metabolism | |
| K03147 | 11 | thiamine biosynthesis protein ThiC | Metabolism | |
| K03148 | 16 | adenylyltransferase ThiF [EC:2.7.7.-] | Metabolism | |

| | | | | |
|---|---|---|---|---|
| K03149 | 27 | thiamine biosynthesis ThiG | Metabolism | |
| K03150 | 1 | thiamine biosynthesis ThiH | Metabolism | |
| K03151 | 4 | thiamine biosynthesis protein ThiI | Metabolism | |
| K03153 | 10 | glycine oxidase [EC:1.4.3.19] | Metabolism | |
| K03179 | 26 | 4-hydroxybenzoate octaprenyltransferase [EC:2.5.1.-] | Metabolism | |
| K03182 | 88 | 3-octaprenyl-4-hydroxybenzoate carboxy-lyase UbiD [EC:4.1.1.-] | Metabolism | |
| K03183 | 77 | ubiquinone/menaquinone biosynthesis methyltransferase [EC:2.1.1.-] | Metabolism | |
| K03184 | 1 | 2-octaprenyl-3-methyl-6-methoxy-1,4-benzoquinol hydroxylase | Metabolism | |
| K03185 | 16 | 2-octaprenyl-6-methoxyphenol hydroxylase [EC:1.14.13.-] | Metabolism | |
| K03186 | 20 | 3-octaprenyl-4-hydroxybenzoate carboxy-lyase UbiX [EC:4.1.1.-] | Metabolism | |
| K03269 | 5 | UDP-2,3-diacylglucosamine hydrolase [EC:3.6.1.-] | Metabolism | |
| K03270 | 14 | 3-deoxy-D-manno-octulosonate 8-phosphate phosphatase (KDO 8-P | Metabolism | |
| K03271 | 12 | phosphoheptose isomerase [EC:5.-.-.-] | Metabolism | |
| K03272 | 33 | D-beta-D-heptose 7-phosphate kinase / D-beta-D-heptose 1-phosphate | Metabolism | |
| K03273 | 14 | D-glycero-D-manno-heptose 1,7-bisphosphate phosphatase [EC:3.1.3.-] | Metabolism | |
| K03274 | 9 | ADP-L-glycero-D-manno-heptose 6-epimerase [EC:5.1.3.20] | Metabolism | |
| K03280 | 1 | UDP-N-acetylglucosamine:(glucosyl)LPS | Metabolism | |
| K03331 | 1 | L-xylulose reductase [EC:1.1.1.10] | Metabolism | |
| K03332 | 2 | fructan beta-fructosidase [EC:3.2.1.80] | Metabolism | |
| K03333 | 41 | cholesterol oxidase [EC:1.1.3.6] | Metabolism | |
| K03335 | 20 | inosose dehydratase [EC:4.2.1.44] | Metabolism | |
| K03336 | 12 | 3D-(3,5/4)-trihydroxycyclohexane-1,2-dione hydrolase [EC:3.7.1.-] | Metabolism | |
| K03337 | 2 | 5-deoxy-glucuronate isomerase [EC:5.3.1.-] | Metabolism | |
| K03338 | 5 | 5-dehydro-2-deoxygluconokinase [EC:2.7.1.92] | Metabolism | |
| K03339 | 1 | 6-phospho-5-dehydro-2-deoxy-D-gluconate aldolase [EC:4.1.2.29] | Metabolism | |
| K03342 | 7 | para-aminobenzoate synthetase / 4-amino-4-deoxychorismate lyase | Metabolism | |
| K03343 | 8 | putrescine oxidase [EC:1.4.3.10] | Metabolism | |
| K03366 | 4 | (R,R)-butanediol dehydrogenase / diacetyl reductase [EC:1.1.1.4 | Metabolism | |
| K03367 | 32 | D-alanine-poly(phosphoribitol) ligase [EC:6.1.1.13] | Metabolism | |
| K03379 | 37 | cyclohexanone monooxygenase [EC:1.14.13.22] | Metabolism | |
| K03380 | 35 | phenol 2-monooxygenase [EC:1.14.13.7] | Metabolism | |
| K03381 | 13 | catechol 1,2-dioxygenase [EC:1.13.11.1] | Metabolism | |
| K03382 | 5 | hydroxyatrazine ethylaminohydrolase [EC:3.5.99.3] | Metabolism | |
| K03385 | 9 | formate-dependent nitrite reductase, periplasmic cytochrome c552 | Metabolism | |
| K03388 | 65 | heterodisulfide reductase subunit A [EC:1.8.98.1] | Metabolism | |
| K03389 | 22 | heterodisulfide reductase subunit B [EC:1.8.98.1] | Metabolism | |
| K03390 | 14 | heterodisulfide reductase subunit C [EC:1.8.98.1] | Metabolism | |
| K03391 | 42 | pentachlorophenol monooxygenase [EC:1.14.13.50] | Metabolism | |
| K03392 | 61 | aminocarboxymuconate-semialdehyde decarboxylase [EC:4.1.1.45] | Metabolism | |
| K03394 | 1 | precorrin-2 C20-methyltransferase / cobalt-factor-2 | Metabolism | |
| K03396 | 1 | S-(hydroxymethyl)glutathione synthase [EC:4.4.1.22] | Metabolism | |
| K03399 | 2 | precorrin-6Y C5,15-methyltransferase [EC:2.1.1.132] | Metabolism | |
| K03403 | 4 | magnesium chelatase subunit H [EC:6.6.1.1] | Metabolism | |
| K03404 | 10 | magnesium chelatase subunit D [EC:6.6.1.1] | Metabolism | |
| K03405 | 29 | magnesium chelatase subunit I [EC:6.6.1.1] | Metabolism | |
| K03416 | 11 | methylmalonyl-CoA carboxyltransferase [EC:2.1.3.1] | Metabolism | |
| K03417 | 36 | methylisocitrate lyase [EC:4.1.3.30] | Metabolism | |
| K03426 | 21 | NAD+ diphosphatase [EC:3.6.1.22] | Metabolism | |
| K03429 | 22 | 1,2-diacylglycerol 3-glucosyltransferase [EC:2.4.1.157] | Metabolism | |
| K03430 | 5 | 2-aminoethylphosphonate-pyruvate transaminase [EC:2.6.1.37] | Metabolism | |
| K03431 | 33 | phosphoglucosamine mutase [EC:5.4.2.10] | Metabolism | |
| K03434 | 14 | N-acetylglucosaminylphosphatidylinositol deacetylase [EC:3.5.1.89] | Metabolism | |
| K03464 | 2 | muconolactone D-isomerase [EC:5.3.3.4] | Metabolism | |
| K03465 | 12 | thymidylate synthase (FAD) [EC:2.1.1.148] | Metabolism | |
| K03473 | 1 | erythronate-4-phosphate dehydrogenase [EC:1.1.1.290] | Metabolism | |
| K03474 | 23 | pyridoxine 5-phosphate synthase [EC:2.6.99.2] | Metabolism | |
| K03475 | 1 | PTS system, ascorbate-specific IIC component | Metabolism | |

| | | | | |
|---|---|---|---|---|
| K03476 | 18 | L-ascorbate 6-phosphate lactonase [EC:3.1.1.-] | Metabolism | |
| K03517 | 23 | quinolinate synthase [EC:2.5.1.72] | Metabolism | |
| K03518 | 27 | carbon-monoxide dehydrogenase small subunit [EC:1.2.99.2] | Metabolism | |
| K03519 | 52 | carbon-monoxide dehydrogenase medium subunit [EC:1.2.99.2] | Metabolism | |
| K03520 | 196 | carbon-monoxide dehydrogenase large subunit [EC:1.2.99.2] | Metabolism | |
| K03524 | 31 | BirA family transcriptional regulator, biotin operon repressor / | Metabolism | |
| K03525 | 31 | type III pantothenate kinase [EC:2.7.1.33] | Metabolism | |
| K03526 | 24 | (E)-4-hydroxy-3-methylbut-2-enyl-diphosphate synthase [EC:1.17.7.1] | Metabolism | |
| K03527 | 34 | 4-hydroxy-3-methylbut-2-enyl diphosphate reductase [EC:1.17.1.2] | Metabolism | |
| K03587 | 74 | cell division protein FtsI (penicillin-binding protein 3) | Metabolism | |
| K03594 | 20 | bacterioferritin | Metabolism | |
| K03621 | 30 | glycerol-3-phosphate acyltransferase PlsX [EC:2.3.1.15] | Metabolism | |
| K03635 | 22 | molybdenum cofactor biosynthesis protein E | Metabolism | |
| K03636 | 10 | molybdenum cofactor biosynthesis protein D | Metabolism | |
| K03637 | 16 | molybdenum cofactor biosynthesis protein C | Metabolism | |
| K03639 | 47 | molybdenum cofactor biosynthesis protein A | Metabolism | |
| K03644 | 32 | lipoic acid synthetase [EC:2.8.1.8] | Metabolism | |
| K03693 | 1 | penicillin-binding protein | Metabolism | |
| K03707 | 6 | transcriptional activator TenA [EC:3.5.99.2] | Metabolism | |
| K03715 | 6 | 1,2-diacylglycerol 3-beta-galactosyltransferase [EC:2.4.1.46] | Metabolism | |
| K03735 | 4 | ethanolamine ammonia-lyase large subunit [EC:4.3.1.7] | Metabolism | |
| K03736 | 1 | ethanolamine ammonia-lyase small subunit [EC:4.3.1.7] | Metabolism | |
| K03737 | 30 | putative pyruvate-flavodoxin oxidoreductase [EC:1.2.7.-] | Metabolism | |
| K03738 | 68 | aldehyde:ferredoxin oxidoreductase [EC:1.2.7.5] | Metabolism | |
| K03742 | 58 | competence/damage-inducible protein CinA | Metabolism | |
| K03743 | 19 | Unclassified; K03743 | Metabolism | |
| K03763 | 10 | DNA polymerase III subunit alpha, Gram-positive type [EC:2.7.7.7] | Metabolism | |
| K03777 | 7 | D-lactate dehydrogenase [EC:1.1.1.28] | Metabolism | |
| K03778 | 7 | D-lactate dehydrogenase [EC:1.1.1.28] | Metabolism | |
| K03779 | 2 | L(+)-tartrate dehydratase alpha subunit [EC:4.2.1.32] | Metabolism | |
| K03780 | 3 | L(+)-tartrate dehydratase beta subunit [EC:4.2.1.32] | Metabolism | |
| K03781 | 12 | catalase [EC:1.11.1.6] | Metabolism | |
| K03782 | 24 | catalase/peroxidase [EC:1.11.1.6 1.11.1.7] | Metabolism | |
| K03783 | 27 | purine-nucleoside phosphorylase [EC:2.4.2.1] | Metabolism | |
| K03784 | 5 | purine-nucleoside phosphorylase [EC:2.4.2.1] | Metabolism | |
| K03785 | 2 | 3-dehydroquinate dehydratase I [EC:4.2.1.10] | Metabolism | |
| K03786 | 17 | 3-dehydroquinate dehydratase II [EC:4.2.1.10] | Metabolism | |
| K03787 | 26 | 5'-nucleotidase [EC:3.1.3.5] | Metabolism | |
| K03795 | 10 | sirohydrochlorin cobaltochelatase [EC:4.99.1.3] | Metabolism | |
| K03800 | 12 | lipoate-protein ligase A [EC:2.7.7.63] | Metabolism | |
| K03801 | 27 | lipoyl(octanoyl) transferase [EC:2.3.1.181] | Metabolism | |
| K03809 | 6 | Trp repressor binding protein | Metabolism | |
| K03814 | 19 | monofunctional biosynthetic peptidoglycan transglycosylase | Metabolism | |
| K03815 | 1 | xanthosine phosphorylase [EC:2.4.2.-] | Metabolism | |
| K03821 | 52 | polyhydroxyalkanoate synthase [EC:2.3.1.-] | Metabolism | |
| K03823 | 23 | phosphinothricin acetyltransferase [EC:2.3.1.183] | Metabolism | |
| K03841 | 11 | fructose-1,6-bisphosphatase I [EC:3.1.3.11] | Metabolism | |
| K03851 | 2 | taurine-pyruvate aminotransferase [EC:2.6.1.77] | Metabolism | |
| K03852 | 6 | sulfoacetaldehyde acetyltransferase [EC:2.3.3.15] | Metabolism | |
| K03856 | 30 | 3-deoxy-7-phosphoheptulonate synthase [EC:2.5.1.54] | Metabolism | |
| K03862 | 13 | vanillate monooxygenase [EC:1.14.13.82] | Metabolism | |
| K03863 | 2 | vanillate monooxygenase [EC:1.14.13.82] | Metabolism | |
| K03885 | 77 | NADH dehydrogenase [EC:1.6.99.3] | Metabolism | |
| K03886 | 3 | menaquinol-cytochrome c reductase iron-sulfur subunit [EC:1.10.2.-] | Metabolism | |
| K03887 | 9 | menaquinol-cytochrome c reductase cytochrome b subunit [EC:1.10.2.-] | Metabolism | |
| K03888 | 2 | menaquinol-cytochrome c reductase cytochrome b/c subunit | Metabolism | |
| K03889 | 6 | ubiquinol-cytochrome c reductase cytochrome c subunit | Metabolism | |

| | | | | |
|---|---|---|---|---|
| K03890 | 3 | ubiquinol-cytochrome c reductase iron-sulfur subunit | Metabolism | |
| K03891 | 15 | ubiquinol-cytochrome c reductase cytochrome b subunit | Metabolism | |
| K03897 | 1 | lysine N6-hydroxylase [EC:1.14.13.59] | Metabolism | |
| K03927 | 17 | carboxylesterase type B [EC:3.1.1.1] | Metabolism | |
| K03943 | 6 | NADH dehydrogenase (ubiquinone) flavoprotein 2 [EC:1.6.5.3 1.6.99.3] | Metabolism | |
| K03980 | 50 | virulence factor | Metabolism | |
| K04019 | 11 | ethanolamine utilization protein EutA | Metabolism | |
| K04020 | 4 | phosphotransacetylase | Metabolism | |
| K04021 | 1 | aldehyde dehydrogenase | Metabolism | |
| K04034 | 54 | anaerobic magnesium-protoporphyrin IX monomethyl ester cyclase | Metabolism | |
| K04035 | 62 | magnesium-protoporphyrin IX monomethyl ester (oxidative) cyclase | Metabolism | |
| K04041 | 6 | fructose-1,6-bisphosphatase III [EC:3.1.3.11] | Metabolism | |
| K04042 | 55 | bifunctional protein GlmU [EC:2.7.7.23 2.3.1.157] | Metabolism | |
| K04072 | 15 | acetaldehyde dehydrogenase / alcohol dehydrogenase [EC:1.2.1.10 | Metabolism | |
| K04073 | 3 | acetaldehyde dehydrogenase [EC:1.2.1.10] | Metabolism | |
| K04091 | 50 | alkanesulfonate monooxygenase [EC:1.14.14.5] | Metabolism | |
| K04097 | 7 | glutathione S-transferase [EC:2.5.1.18] | Metabolism | |
| K04098 | 2 | hydroxyquinol 1,2-dioxygenase [EC:1.13.11.37] | Metabolism | |
| K04103 | 3 | indolepyruvate decarboxylase [EC:4.1.1.74] | Metabolism | |
| K04107 | 6 | 4-hydroxybenzoyl-CoA reductase subunit 1 [EC:1.3.99.20] | Metabolism | |
| K04108 | 42 | 4-hydroxybenzoyl-CoA reductase subunit 2 [EC:1.3.99.20] | Metabolism | |
| K04110 | 22 | benzoate-CoA ligase [EC:6.2.1.25] | Metabolism | |
| K04112 | 1 | benzoyl-CoA reductase subunit [EC:1.3.99.15] | Metabolism | |
| K04113 | 10 | benzoyl-CoA reductase subunit [EC:1.3.99.15] | Metabolism | |
| K04114 | 29 | benzoyl-CoA reductase subunit [EC:1.3.99.15] | Metabolism | |
| K04115 | 16 | benzoyl-CoA reductase subunit [EC:1.3.99.15] | Metabolism | |
| K04117 | 32 | cyclohexanecarboxyl-CoA dehydrogenase [EC:1.3.99.-] | Metabolism | |
| K04118 | 4 | pimeloyl-CoA dehydrogenase [EC:1.3.1.62] | Metabolism | |
| K04126 | 1 | isopenicillin-N synthase [EC:1.21.3.1] | Metabolism | |
| K04127 | 15 | isopenicillin-N epimerase [EC:5.1.1.17] | Metabolism | |
| K04128 | 1 | hydroxymethyl cephem carbamoyltransferase [EC:2.1.3.-] | Metabolism | |
| K04340 | 1 | scyllo-inosamine-4-phosphate amidinotransferase 1 [EC:2.1.4.2] | Metabolism | |
| K04486 | 6 | histidinol-phosphatase (PHP family) [EC:3.1.3.15] | Metabolism | |
| K04487 | 57 | cysteine desulfurase [EC:2.8.1.7] | Metabolism | |
| K04516 | 4 | chorismate mutase [EC:5.4.99.5] | Metabolism | |
| K04517 | 16 | prephenate dehydrogenase [EC:1.3.1.12] | Metabolism | |
| K04518 | 51 | prephenate dehydratase [EC:4.2.1.51] | Metabolism | |
| K04561 | 21 | nitric-oxide reductase, cytochrome b-containing subunit I | Metabolism | |
| K04719 | 4 | 5,6-dimethylbenzimidazole biosynthesis protein BluB | Metabolism | |
| K04780 | 19 | nonribosomal peptide synthetase DhbF | Metabolism | |
| K04782 | 8 | isochorismate pyruvate-lyase [EC:4.1.3.-] | Metabolism | |
| K04783 | 2 | yersiniabactin salicyl-AMP ligase [EC:6.3.2.-] | Metabolism | |
| K04786 | 3 | yersiniabactin nonribosomal peptide/polyketide synthase | Metabolism | |
| K04787 | 6 | mycobactin salicyl-AMP ligase [EC:6.3.2.-] | Metabolism | |
| K05275 | 26 | pyridoxine 4-dehydrogenase [EC:1.1.1.65] | Metabolism | |
| K05281 | 4 | 2'-hydroxyisoflavone reductase [EC:1.3.1.45] | Metabolism | |
| K05296 | 3 | 3(or 17)beta-hydroxysteroid dehydrogenase [EC:1.1.1.51] | Metabolism | |
| K05297 | 6 | rubredoxin-NAD+ reductase [EC:1.18.1.1] | Metabolism | |
| K05299 | 10 | formate dehydrogenase (NADP+) [EC:1.2.1.43] | Metabolism | |
| K05301 | 55 | sulfite dehydrogenase [EC:1.8.2.1] | Metabolism | |
| K05306 | 2 | phosphonoacetaldehyde hydrolase [EC:3.11.1.1] | Metabolism | |
| K05341 | 5 | amylosucrase [EC:2.4.1.4] | Metabolism | |
| K05343 | 42 | maltose alpha-D-glucosyltransferase [EC:5.4.99.16] | Metabolism | |
| K05344 | 1 | glucose-1-phosphate phosphodismutase [EC:2.7.1.41] | Metabolism | |
| K05349 | 79 | beta-glucosidase [EC:3.2.1.21] | Metabolism | |
| K05350 | 31 | beta-glucosidase [EC:3.2.1.21] | Metabolism | |
| K05351 | 2 | D-xylulose reductase [EC:1.1.1.9] | Metabolism | |

| | | | | |
|---|---|---|---|---|
| K05358 | 3 | quinate dehydrogenase (pyrroloquinoline-quinone) [EC:1.1.99.25] | Metabolism | |
| K05359 | 1 | carboxycyclohexadienyl dehydratase [EC:4.2.1.91] | Metabolism | |
| K05362 | 1 | UDP-N-acetylmuramoyl-L-alanyl-D-glutamate-L-lysine ligase | Metabolism | |
| K05363 | 1 | serine/alanine adding enzyme [EC:2.3.2.10] | Metabolism | |
| K05364 | 58 | peptidoglycan glycosyltransferase [EC:2.4.1.129] | Metabolism | |
| K05365 | 12 | penicillin-binding protein 1B [EC:2.4.1.129 3.4.-.-] | Metabolism | |
| K05366 | 129 | penicillin-binding protein 1A [EC:2.4.1.- 3.4.-.-] | Metabolism | |
| K05367 | 18 | penicillin-binding protein 1C [EC:2.4.1.-] | Metabolism | |
| K05368 | 1 | aquacobalamin reductase / NAD(P)H-flavin reductase [EC:1.16.1.3 | Metabolism | |
| K05375 | 2 | MbtH protein | Metabolism | |
| K05384 | 22 | bilin biosynthesis protein | Metabolism | |
| K05386 | 8 | bilin biosynthesis protein | Metabolism | |
| K05394 | 29 | atrazine chlorohydrolase [EC:3.8.1.8] | Metabolism | |
| K05396 | 10 | D-cysteine desulfhydrase [EC:4.4.1.15] | Metabolism | |
| K05515 | 85 | penicillin-binding protein 2 | Metabolism | |
| K05525 | 30 | linalool 8-monooxygenase [EC:1.14.99.28] | Metabolism | |
| K05549 | 11 | benzoate 1,2-dioxygenase alpha subunit [EC:1.14.12.10] | Metabolism | |
| K05550 | 4 | benzoate 1,2-dioxygenase beta subunit [EC:1.14.12.10] | Metabolism | |
| K05551 | 24 | 3-oxoacyl-ACP synthase I [EC:2.3.1.-] | Metabolism | |
| K05552 | 22 | 3-oxoacyl-ACP synthase II [EC:2.3.1.-] | Metabolism | |
| K05555 | 12 | cyclase [EC:4.-.-.-] | Metabolism | |
| K05556 | 3 | hydroxylacyl-CoA dehydrogenase [EC:1.1.1.-] | Metabolism | |
| K05572 | 6 | NADH dehydrogenase I subunit 1 [EC:1.6.5.3] | Metabolism | |
| K05573 | 9 | NADH dehydrogenase I subunit 2 [EC:1.6.5.3] | Metabolism | |
| K05574 | 6 | NADH dehydrogenase I subunit 3 [EC:1.6.5.3] | Metabolism | |
| K05575 | 8 | NADH dehydrogenase I subunit 4 [EC:1.6.5.3] | Metabolism | |
| K05576 | 3 | NADH dehydrogenase I subunit 4L [EC:1.6.5.3] | Metabolism | |
| K05577 | 7 | NADH dehydrogenase I subunit 5 [EC:1.6.5.3] | Metabolism | |
| K05578 | 2 | NADH dehydrogenase I subunit 6 [EC:1.6.5.3] | Metabolism | |
| K05579 | 2 | NADH dehydrogenase I subunit 7 [EC:1.6.5.3] | Metabolism | |
| K05580 | 4 | NADH dehydrogenase I subunit I [EC:1.6.5.3] | Metabolism | |
| K05586 | 3 | diaphorase subunit of the bidirectional hydrogenase [EC:1.6.5.3] | Metabolism | |
| K05587 | 6 | diaphorase subunit of the bidirectional hydrogenase [EC:1.6.5.3] | Metabolism | |
| K05588 | 3 | diaphorase subunit of the bidirectional hydrogenase [EC:1.6.5.3] | Metabolism | |
| K05597 | 1 | glutamin-(asparagin-)ase [EC:3.5.1.38] | Metabolism | |
| K05599 | 2 | anthranilate 1,2-dioxygenase (deaminating, decarboxylating) large | Metabolism | |
| K05600 | 2 | anthranilate 1,2-dioxygenase (deaminating, decarboxylating) small | Metabolism | |
| K05601 | 2 | hydroxylamine reductase [EC:1.7.99.1] | Metabolism | |
| K05603 | 10 | formimidoylglutamate deiminase [EC:3.5.3.13] | Metabolism | |
| K05606 | 12 | methylmalonyl-CoA epimerase [EC:5.1.99.1] | Metabolism | |
| K05708 | 9 | large terminal subunit of phenylpropionate dioxygenase | Metabolism | |
| K05709 | 3 | small terminal subunit of phenylpropionate dioxygenase | Metabolism | |
| K05710 | 16 | ferredoxin subunit of phenylpropionate dioxygenase | Metabolism | |
| K05712 | 33 | 3-(3-hydroxy-phenyl)propionate hydroxylase [EC:1.14.13.-] | Metabolism | |
| K05714 | 5 | 2-hydroxy-6-ketonona-2,4-dienedioic acid hydrolase [EC:3.7.1.-] | Metabolism | |
| K05780 | 2 | putative phosphonate transport system ATP-binding protein | Metabolism | |
| K05783 | 1 | 1,6-dihydroxycyclohexa-2,4-diene-1-carboxylate dehydrogenase | Metabolism | |
| K05784 | 3 | benzoate 1,2-dioxygenase electron transfer component | Metabolism | |
| K05797 | 3 | 4-cresol dehydrogenase (hydroxylating) [EC:1.17.99.1] | Metabolism | |
| K05823 | 1 | N-acetyldiaminopimelate deacetylase [EC:3.5.1.47] | Metabolism | |
| K05825 | 2 | 2-aminoadipate transaminase [EC:2.6.1.-] | Metabolism | |
| K05827 | 5 | lysine biosynthesis protein LysX | Metabolism | |
| K05829 | 4 | N-acetyl-gamma-aminoadipyl-phosphate reductase [EC:1.2.1.-] | Metabolism | |
| K05830 | 3 | acetylornithine/acetyl-lysine aminotransferase [EC:2.6.1.11 2.6.1.-] | Metabolism | |
| K05831 | 2 | acetyl-lysine deacetylase [EC:3.5.1.-] | Metabolism | |
| K05873 | 1 | adenylate cyclase, class 2 [EC:4.6.1.1] | Metabolism | |
| K05878 | 8 | dihydroxyacetone kinase, N-terminal domain [EC:2.7.1.-] | Metabolism | |

| | | | | |
|---|---|---|---|---|
| K05879 | 5 | dihydroxyacetone kinase, C-terminal domain [EC:2.7.1.-] | Metabolism | |
| K05895 | 2 | precorrin-6X reductase [EC:1.3.1.54] | Metabolism | |
| K05898 | 2 | 3-oxosteroid 1-dehydrogenase [EC:1.3.99.4] | Metabolism | |
| K05917 | 1 | cytochrome P450, family 51 (sterol 14-demethylase) [EC:1.14.13.70] | Metabolism | |
| K05921 | 16 | 5-oxopent-3-ene-1,2,5-tricarboxylate decarboxylase / | Metabolism | |
| K05928 | 8 | tocopherol O-methyltransferase [EC:2.1.1.95] | Metabolism | |
| K05929 | 6 | phosphoethanolamine N-methyltransferase [EC:2.1.1.103] | Metabolism | |
| K05934 | 6 | precorrin-3B C17-methyltransferase [EC:2.1.1.131] | Metabolism | |
| K05936 | 4 | precorrin-4 C11-methyltransferase [EC:2.1.1.133] | Metabolism | |
| K05939 | 12 | acyl-[acyl-carrier-protein]-phospholipid O-acyltransferase / | Metabolism | |
| K05957 | 4 | L-glutamine:scyllo-inosose aminotransferase [EC:2.6.1.50] | Metabolism | |
| K05973 | 12 | poly(3-hydroxybutyrate) depolymerase [EC:3.1.1.75] | Metabolism | |
| K05979 | 11 | 2-phosphosulfolactate phosphatase [EC:3.1.3.71] | Metabolism | |
| K06001 | 31 | tryptophan synthase beta chain [EC:4.2.1.20] | Metabolism | |
| K06013 | 29 | STE24 endopeptidase [EC:3.4.24.84] | Metabolism | |
| K06016 | 27 | N-carbamoyl-L-amino-acid hydrolase [EC:3.5.1.87] | Metabolism | |
| K06019 | 4 | pyrophosphatase PpaX [EC:3.6.1.1] | Metabolism | |
| K06034 | 5 | sulfopyruvate decarboxylase subunit alpha [EC:4.1.1.79] | Metabolism | |
| K06041 | 24 | arabinose-5-phosphate isomerase [EC:5.3.1.13] | Metabolism | |
| K06042 | 8 | precorrin-8X methylmutase [EC:5.4.1.2] | Metabolism | |
| K06044 | 33 | (1->4)-alpha-D-glucan 1-alpha-D-glucosylmutase [EC:5.4.99.15] | Metabolism | |
| K06045 | 23 | squalene-hopene cyclase [EC:5.4.99.17] | Metabolism | |
| K06118 | 5 | UDP-sulfoquinovose synthase [EC:3.13.1.1] | Metabolism | |
| K06120 | 2 | glycerol dehydratase large subunit [EC:4.2.1.30] | Metabolism | |
| K06121 | 1 | glycerol dehydratase medium subunit [EC:4.2.1.30] | Metabolism | |
| K06131 | 35 | cardiolipin synthase [EC:2.7.8.-] | Metabolism | |
| K06132 | 18 | putative cardiolipin synthase [EC:2.7.8.-] | Metabolism | |
| K06134 | 3 | ubiquinone biosynthesis monooxygenase Coq7 [EC:1.14.13.-] | Metabolism | |
| K06151 | 40 | gluconate 2-dehydrogenase alpha chain [EC:1.1.99.3] | Metabolism | |
| K06153 | 39 | undecaprenyl-diphosphatase [EC:3.6.1.27] | Metabolism | |
| K06162 | 1 | PhnM protein | Metabolism | |
| K06163 | 1 | PhnJ protein | Metabolism | |
| K06164 | 3 | PhnI protein | Metabolism | |
| K06167 | 14 | PhnP protein | Metabolism | |
| K06208 | 12 | chorismate mutase [EC:5.4.99.5] | Metabolism | |
| K06211 | 1 | HipB family transcriptional regulator, involved in the regulation of | Metabolism | |
| K06215 | 8 | pyridoxine biosynthesis protein [EC:4.-.-.-] | Metabolism | |
| K06281 | 38 | hydrogenase large subunit [EC:1.12.99.6] | Metabolism | |
| K06282 | 22 | hydrogenase small subunit [EC:1.12.99.6] | Metabolism | |
| K06445 | 16 | acyl-CoA dehydrogenase [EC:1.3.99.-] | Metabolism | |
| K06446 | 6 | acyl-CoA dehydrogenase [EC:1.3.99.-] | Metabolism | |
| K06447 | 2 | succinylglutamic semialdehyde dehydrogenase [EC:1.2.1.71] | Metabolism | |
| K06606 | 2 | inosose isomerase [EC:5.3.99.-] | Metabolism | |
| K06718 | 2 | L-2,4-diaminobutyric acid acetyltransferase [EC:2.3.1.178] | Metabolism | |
| K06859 | 1 | glucose-6-phosphate isomerase, archaeal [EC:5.3.1.9] | Metabolism | |
| K06881 | 34 | Unclassified; K06881 | Metabolism | |
| K06896 | 21 | Unclassified; K06896 | Metabolism | |
| K06912 | 17 | alpha-ketoglutarate-dependent 2,4-dichlorophenoxyacetate dioxygenase | Metabolism | |
| K06920 | 21 | queuosine biosynthesis protein QueC | Metabolism | |
| K06981 | 1 | Unclassified; K06981 | Metabolism | |
| K06989 | 7 | aspartate dehydrogenase [EC:1.4.1.21] | Metabolism | |
| K07008 | 22 | Unclassified; K07008 | Metabolism | |
| K07029 | 47 | Unclassified; K07029 | Metabolism | |
| K07031 | 8 | Unclassified; K07031 | Metabolism | |
| K07046 | 35 | Unclassified; K07046 | Metabolism | |
| K07072 | 2 | Unclassified; K07072 | Metabolism | |
| K07102 | 16 | Unclassified; K07102 | Metabolism | |

| | | | | |
|---|---|---|---|---|
| K07104 | 17 | Unclassified; K07104 | Metabolism | |
| K07106 | 20 | N-acetylmuramic acid 6-phosphate etherase [EC:4.2.-.-] | Metabolism | |
| K07127 | 6 | 5-hydroxyisourate hydrolase [EC:3.5.2.17] | Metabolism | |
| K07130 | 24 | Unclassified; K07130 | Metabolism | |
| K07173 | 15 | S-ribosylhomocysteine lyase [EC:4.4.1.21] | Metabolism | |
| K07246 | 8 | tartrate dehydrogenase/decarboxylase / D-malate dehydrogenase | Metabolism | |
| K07248 | 4 | lactaldehyde dehydrogenase / glycolaldehyde dehydrogenase | Metabolism | |
| K07250 | 30 | 4-aminobutyrate aminotransferase / (S)-3-amino-2-methylpropionate | Metabolism | |
| K07258 | 136 | D-alanyl-D-alanine carboxypeptidase (penicillin-binding protein 5/6) | Metabolism | |
| K07259 | 26 | D-alanyl-D-alanine carboxypeptidase / | Metabolism | |
| K07260 | 7 | D-alanyl-D-alanine carboxypeptidase [EC:3.4.16.4] | Metabolism | |
| K07281 | 1 | Unclassified; K07281 | Metabolism | |
| K07306 | 10 | anaerobic dimethyl sulfoxide reductase subunit A [EC:1.8.99.-] | Metabolism | |
| K07307 | 10 | anaerobic dimethyl sulfoxide reductase subunit B (DMSO reductase | Metabolism | |
| K07308 | 1 | anaerobic dimethyl sulfoxide reductase subunit C (DMSO reductase | Metabolism | |
| K07309 | 1 | putative dimethyl sulfoxide reductase subunit YnfE [EC:1.8.99.-] | Metabolism | |
| K07310 | 1 | putative dimethyl sulfoxide reductase subunit YnfF [EC:1.8.99.-] | Metabolism | |
| K07404 | 25 | 6-phosphogluconolactonase [EC:3.1.1.31] | Metabolism | |
| K07405 | 7 | alpha-amylase [EC:3.2.1.1] | Metabolism | |
| K07406 | 10 | alpha-galactosidase [EC:3.2.1.22] | Metabolism | |
| K07407 | 12 | alpha-galactosidase [EC:3.2.1.22] | Metabolism | |
| K07508 | 6 | acetyl-CoA acyltransferase 2 [EC:2.3.1.16] | Metabolism | |
| K07516 | 43 | 3-hydroxyacyl-CoA dehydrogenase [EC:1.1.1.35] | Metabolism | |
| K07534 | 4 | cyclohex-1-ene-1-carboxyl-CoA hydratase [EC:4.2.1.-] | Metabolism | |
| K07535 | 35 | 2-hydroxycyclohexanecarboxyl-CoA dehydrogenase [EC:1.1.1.-] | Metabolism | |
| K07537 | 4 | cyclohexa-1,5-dienecarbonyl-CoA hydratase [EC:4.2.1.100] | Metabolism | |
| K07538 | 3 | 6-hydroxycyclohex-1-ene-1-carboxyl-CoA dehydrogenase [EC:1.1.1.-] | Metabolism | |
| K07543 | 23 | benzylsuccinate CoA-transferase BbsE subunit [EC:2.8.3.15] | Metabolism | |
| K07544 | 22 | benzylsuccinate CoA-transferase BbsF subunit [EC:2.8.3.15] | Metabolism | |
| K07545 | 1 | (R)-benzylsuccinyl-CoA dehydro genase [EC:1.3.99.21] | Metabolism | |
| K07546 | 8 | E-phenylitaconyl-CoA hydratase [EC:4.2.1.-] | Metabolism | |
| K07547 | 16 | 2-[hydroxy(phenyl)methyl]-succinyl-CoA dehydrogenase BbsC subunit | Metabolism | |
| K07548 | 10 | 2-[hydroxy(phenyl)methyl]-succinyl-CoA dehydrogenase BbsD subunit | Metabolism | |
| K07550 | 12 | benzoylsuccinyl-CoA thiolase BbsB subunit [EC:2.3.1.-] | Metabolism | |
| K07748 | 2 | sterol-4alpha-carboxylate 3-dehydrogenase (decarboxylating) | Metabolism | |
| K07758 | 5 | pyridoxal phosphatase [EC:3.1.3.74] | Metabolism | |
| K07806 | 20 | UDP-4-amino-4-deoxy-L-arabinose-oxoglutarate aminotransferase | Metabolism | |
| K07823 | 4 | 3-oxoadipyl-CoA thiolase [EC:2.3.1.174] | Metabolism | |
| K07824 | 6 | benzoate 4-monooxygenase [EC:1.14.13.12] | Metabolism | |
| K08068 | 1 | N-acetylglucosamine-6-phosphate 2-epimerase and phosphatase | Metabolism | |
| K08093 | 1 | 3-hexulose-6-phosphate synthase [EC:4.1.2.43] | Metabolism | |
| K08097 | 6 | phosphosulfolactate synthase [EC:4.4.1.19] | Metabolism | |
| K08100 | 49 | bilirubin oxidase [EC:1.3.3.5] | Metabolism | |
| K08261 | 13 | D-sorbitol dehydrogenase (acceptor) [EC:1.1.99.21] | Metabolism | |
| K08281 | 14 | nicotinamidase/pyrazinamidase [EC:3.5.1.19 3.5.1.-] | Metabolism | |
| K08289 | 4 | phosphoribosylglycinamide formyltransferase 2 [EC:2.1.2.2] | Metabolism | |
| K08295 | 5 | 2-aminobenzoate-CoA ligase [EC:6.2.1.32] | Metabolism | |
| K08302 | 1 | tagatose 1,6-diphosphate aldolase [EC:4.1.2.40] | Metabolism | |
| K08310 | 5 | dATP pyrophosphohydrolase [EC:3.6.1.-] | Metabolism | |
| K08318 | 2 | putative dehydrogenase [EC:1.1.-.-] | Metabolism | |
| K08322 | 8 | starvation sensing protein RspB [EC:1.1.1.-] | Metabolism | |
| K08323 | 9 | starvation sensing protein RspA | Metabolism | |
| K08324 | 3 | aldehyde dehydrogenase family protein [EC:1.2.1.-] | Metabolism | |
| K08351 | 5 | biotin sulfoxide reductase [EC:1.-.-.-] | Metabolism | |
| K08352 | 38 | thiosulfate reductase [EC:1.-.-.-] | Metabolism | |
| K08353 | 5 | thiosulfate reductase electron transport protein | Metabolism | |
| K08354 | 3 | thiosulfate reductase cytochrome b subunit | Metabolism | |

| | | | | |
|---|---|---|---|---|
| K08357 | 9 | tetrathionate reductase subunit A | Metabolism | |
| K08358 | 27 | tetrathionate reductase subunit B | Metabolism | |
| K08359 | 4 | tetrathionate reductase subunit C | Metabolism | |
| K08590 | 4 | carbon-nitrogen hydrolase family protein | Metabolism | |
| K08591 | 25 | glycerol-3-phosphate acyltransferase PlsY [EC:2.3.1.15] | Metabolism | |
| K08678 | 5 | UDP-glucuronate decarboxylase [EC:4.1.1.35] | Metabolism | |
| K08679 | 5 | UDP-glucuronate 4-epimerase [EC:5.1.3.6] | Metabolism | |
| K08680 | 8 | 2-succinyl-6-hydroxy-2,4-cyclohexadiene-1-carboxylate synthase | Metabolism | |
| K08681 | 4 | glutamine amidotransferase [EC:2.6.-.-] | Metabolism | |
| K08682 | 2 | acyl carrier protein phosphodiesterase [EC:3.1.4.14] | Metabolism | |
| K08686 | 15 | 2-chlorobenzoate 1,2-dioxygenase [EC:1.14.12.13] | Metabolism | |
| K08687 | 4 | N-carbamoylsarcosine amidase [EC:3.5.1.59] | Metabolism | |
| K08688 | 9 | creatinase [EC:3.5.3.3] | Metabolism | |
| K08693 | 4 | 3'-nucleotidase [EC:3.1.3.6] | Metabolism | |
| K08710 | 5 | N-isopropylammelide isopropylaminohydrolase [EC:3.5.99.4] | Metabolism | |
| K08723 | 4 | 5'-nucleotidase [EC:3.1.3.5] | Metabolism | |
| K08724 | 5 | penicillin-binding protein 2B | Metabolism | |
| K08738 | 12 | cytochrome c | Metabolism | |
| K08963 | 23 | methylthioribose-1-phosphate isomerase [EC:5.3.1.23] | Metabolism | |
| K08964 | 2 | methylthioribulose-1-phosphate dehydratase [EC:4.2.1.109] | Metabolism | |
| K08965 | 1 | 2,3-diketo-5-methylthiopentyl-1-phosphate enolase [EC:3.1.3.77] | Metabolism | |
| K08966 | 3 | 2-hydroxy-3-keto-5-methylthiopentenyl-1-phosphate phosphatase | Metabolism | |
| K08967 | 4 | 1,2-dihydroxy-3-keto-5-methylthiopentene dioxygenase [EC:1.13.11.53 | Metabolism | |
| K08969 | 1 | aminotransferase [EC:2.6.1.-] | Metabolism | |
| K09007 | 8 | hypothetical protein | Metabolism | |
| K09011 | 7 | D-citramalate synthase [EC:2.3.1.182] | Metabolism | |
| K09018 | 1 | putative monooxygenase RutA [EC:1.14.-.-] | Metabolism | |
| K09019 | 4 | putative NADH dehydrogenase/NAD(P)H nitroreductase RutE [EC:1.-.-.-] | Metabolism | |
| K09020 | 6 | putative isochorismatase family protein RutB [EC:3.-.-.-] | Metabolism | |
| K09023 | 5 | protein RutD | Metabolism | |
| K09024 | 2 | putative flavin reductase RutF [EC:1.5.1.-] | Metabolism | |
| K09065 | 3 | N-acetylornithine carbamoyltransferase [EC:2.1.3.9] | Metabolism | |
| K09251 | 10 | putrescine aminotransferase [EC:2.6.1.82] | Metabolism | |
| K09457 | 12 | 7-cyano-7-deazaguanine reductase [EC:1.7.1.13] | Metabolism | |
| K09458 | 82 | 3-oxoacyl-[acyl-carrier-protein] synthase II [EC:2.3.1.179] | Metabolism | |
| K09459 | 5 | phosphonopyruvate decarboxylase [EC:4.1.1.82] | Metabolism | |
| K09461 | 29 | anthraniloyl-CoA monooxygenase [EC:1.14.13.40] | Metabolism | |
| K09471 | 25 | gamma-glutamylputrescine oxidase [EC:1.4.3.-] | Metabolism | |
| K09472 | 7 | gamma-glutamyl-gamma-aminobutyraldehyde dehydrogenase [EC:1.2.1.-] | Metabolism | |
| K09474 | 2 | acid phosphatase (class A) [EC:3.1.3.2] | Metabolism | |
| K09516 | 1 | all-trans-retinol 13,14-reductase [EC:1.3.99.23] | Metabolism | |
| K09680 | 3 | type II pantothenate kinase [EC:2.7.1.33] | Metabolism | |
| K09699 | 12 | 2-oxoisovalerate dehydrogenase E2 component (dihydrolipoyl | Metabolism | |
| K09709 | 9 | hypothetical protein | Metabolism | |
| K09758 | 10 | aspartate 4-decarboxylase [EC:4.1.1.12] | Metabolism | |
| K09788 | 41 | hypothetical protein | Metabolism | |
| K09833 | 1 | homogenitisate phytyltransferase | Metabolism | |
| K09835 | 2 | carotenoid isomerase [EC:5.-.-.-] | Metabolism | |
| K09845 | 3 | methoxyneurosporene dehydrogenase [EC:1.14.99.-] | Metabolism | |
| K09846 | 18 | hydroxyneurosporene methyltransferase [EC:2.1.1.-] | Metabolism | |
| K09879 | 1 | isorenieratene synthase | Metabolism | |
| K09882 | 3 | cobaltochelatase CobS [EC:6.6.1.2] | Metabolism | |
| K09883 | 6 | cobaltochelatase CobT [EC:6.6.1.2] | Metabolism | |
| K09903 | 23 | uridylate kinase [EC:2.7.4.22] | Metabolism | |
| K10011 | 7 | UDP-GlcUA decarboxylase/UDP-L-Ara4N formyltransferase | Metabolism | |
| K10012 | 8 | undecaprenyl-phosphate 4-deoxy-4-formamido-L-arabinose transferase | Metabolism | |
| K10026 | 15 | queuosine biosynthesis protein QueE | Metabolism | |

| | | | | |
|---|---|---|---|---|
| K10027 | 11 | phytoene dehydrogenase [EC:1.14.99.-] | Metabolism | |
| K10206 | 25 | LL-diaminopimelate aminotransferase [EC:2.6.1.83] | Metabolism | |
| K10209 | 1 | dehydrosqualene desaturase [EC:1.14.99.-] | Metabolism | |
| K10210 | 3 | 4,4'-diaponeurosporene oxidase [EC:1.-.-.-] | Metabolism | |
| K10215 | 11 | monooxygenase [EC:1.14.13.-] | Metabolism | |
| K10217 | 4 | aminomuconate-semialdehyde dehydrogenase [EC:1.2.1.32] | Metabolism | |
| K10218 | 5 | 4-hydroxy-4-methyl-2-oxoglutarate aldolase [EC:4.1.3.17] | Metabolism | |
| K10219 | 34 | 4-carboxy-2-hydroxymuconate-6-semialdehyde dehydrogenase | Metabolism | |
| K10220 | 52 | 4-oxalmesaconate hydratase [EC:4.2.1.83] | Metabolism | |
| K10221 | 17 | 2-pyrone-4,6-dicarboxylate lactonase [EC:3.1.1.57] | Metabolism | |
| K10353 | 5 | deoxyadenosine kinase [EC:2.7.1.76] | Metabolism | |
| K10533 | 3 | limonene-1,2-epoxide hydrolase [EC:3.3.2.8] | Metabolism | |
| K10536 | 12 | agmatine deiminase [EC:3.5.3.12] | Metabolism | |
| K10616 | 19 | p-cymene monooxygenase [EC:1.14.13.-] | Metabolism | |
| K10617 | 7 | p-cumic alcohol dehydrogenase | Metabolism | |
| K10618 | 7 | p-cumic aldehyde dehydrogenase | Metabolism | |
| K10619 | 17 | p-cumate dioxygenase [EC:1.14.12.-] | Metabolism | |
| K10620 | 1 | 2,3-dihydroxy-2,3-dihydro-p-cumate dehydrogenase [EC:1.3.1.58] | Metabolism | |
| K10621 | 5 | 2,3-dihydroxy-p-cumate-3,4-dioxygenase [EC:1.13.11.-] | Metabolism | |
| K10622 | 2 | HCOMODA decarboxylase [EC:4.1.1.-] | Metabolism | |
| K10674 | 2 | ectoine hydroxylase [EC:1.14.11.-] | Metabolism | |
| K10676 | 13 | 2,4-dichlorophenol 6-monooxygenase [EC:1.14.13.20] | Metabolism | |
| K10678 | 1 | nitroreductase [EC:1.-.-.-] | Metabolism | |
| K10679 | 1 | nitroreductase / dihydropteridine reductase [EC:1.-.-.- 1.5.1.34] | Metabolism | |
| K10680 | 11 | N-ethylmaleimide reductase [EC:1.-.-.-] | Metabolism | |
| K10700 | 13 | ethylbenzene hydroxylase [EC:1.17.99.2] | Metabolism | |
| K10701 | 20 | acetophenone carboxylase [EC:6.4.1.-] | Metabolism | |
| K10713 | 1 | formaldehyde-activating enzyme [EC:4.3.-.-] | Metabolism | |
| K10764 | 12 | O-succinylhomoserine sulfhydrylase [EC:2.5.1.-] | Metabolism | |
| K10774 | 6 | tyrosine ammonia-lyase [EC:4.3.1.23] | Metabolism | |
| K10775 | 1 | phenylalanine ammonia-lyase [EC:4.3.1.24] | Metabolism | |
| K10780 | 3 | enoyl-[acyl carrier protein] reductase III [EC:1.3.1.-] | Metabolism | |
| K10804 | 10 | acyl-CoA thioesterase I [EC:3.1.2.- 3.1.1.5] | Metabolism | |
| K10805 | 10 | acyl-CoA thioesterase II [EC:3.1.2.-] | Metabolism | |
| K10806 | 4 | acyl-CoA thioesterase YciA [EC:3.1.2.-] | Metabolism | |
| K10807 | 1 | ribonucleoside-diphosphate reductase subunit M1 [EC:1.17.4.1] | Metabolism | |
| K10810 | 1 | transcriptional regulator TenI | Metabolism | |
| K10815 | 1 | hydrogen cyanide synthase HcnB [EC:1.4.99.5] | Metabolism | |
| K10816 | 2 | hydrogen cyanide synthase HcnC [EC:1.4.99.5] | Metabolism | |
| K10817 | 32 | erythronolide synthase [EC:2.3.1.94] | Metabolism | |
| K11029 | 4 | anthrax edema toxin adenylate cyclase [EC:4.6.1.1] | Metabolism | |
| K11175 | 32 | phosphoribosylglycinamide formyltransferase 1 [EC:2.1.2.2] | Metabolism | |
| K11177 | 61 | xanthine dehydrogenase YagR molybdenum-binding subunit [EC:1.17.1.4] | Metabolism | |
| K11178 | 19 | xanthine dehydrogenase YagS FAD-binding subunit [EC:1.17.1.4] | Metabolism | |
| K11180 | 3 | sulfite reductase, dissimilatory-type alpha subunit [EC:1.8.99.3] | Metabolism | |
| K11181 | 3 | sulfite reductase, dissimilatory-type beta subunit [EC:1.8.99.3] | Metabolism | |
| K11192 | 1 | PTS system, N-acetylmuramic acid-specific IIC component | Metabolism | |
| K11212 | 20 | LPPG:FO 2-phospho-L-lactate transferase [EC:2.7.8.-] | Metabolism | |
| K11258 | 2 | acetolactate synthase II small subunit [EC:2.2.1.6] | Metabolism | |
| K11261 | 6 | formylmethanofuran dehydrogenase subunit E [EC:1.2.99.5] | Metabolism | |
| K11263 | 26 | acetyl-/propionyl-CoA carboxylase, biotin carboxylase, biotin | Metabolism | |
| K11264 | 2 | methylmalonyl-CoA decarboxylase [EC:4.1.1.41] | Metabolism | |
| K11311 | 2 | anthranilate dioxygenase reductase | Metabolism | |
| K11333 | 1 | chlorophyllide reductase iron protein subunit X [EC:1.18.6.1] | Metabolism | |
| K11337 | 3 | 3-hydroxyethyl bacteriochlorophyllide a dehydrogenase [EC:1.-.-.-] | Metabolism | |
| K11358 | 4 | aspartate aminotransferase [EC:2.6.1.1] | Metabolism | |
| K11381 | 62 | 2-oxoisovalerate dehydrogenase E1 component [EC:1.2.4.4] | Metabolism | |

| | | | | |
|---|---|---|---|---|
| K11472 | 15 | glycolate oxidase FAD binding subunit | Metabolism | |
| K11473 | 36 | glycolate oxidase iron-sulfur subunit | Metabolism | |
| K11528 | 4 | UDP-N-acetylglucosamine pyrophosphorylase [EC:2.7.7.23] | Metabolism | |
| K11532 | 1 | fructose-1,6-bisphosphatase II / sedoheptulose-1,7-bisphosphatase | Metabolism | |
| K11533 | 1 | fatty acid synthase, bacteria type [EC:2.3.1.-] | Metabolism | |
| K11646 | 1 | dehydroquinate synthase II [EC:1.4.1.-] | Metabolism | |
| K11693 | 2 | peptidoglycan pentaglycine glycine transferase (the first glycine) | Metabolism | |
| K11717 | 72 | cysteine desulfurase / selenocysteine lyase [EC:2.8.1.7 4.4.1.16] | Metabolism | |
| K11731 | 6 | citronellyl-CoA dehydrogenase [EC:1.3.99.-] | Metabolism | |
| K11751 | 4 | 5'-nucleotidase / UDP-sugar diphosphatase [EC:3.1.3.5 3.6.1.45] | Metabolism | |
| K11752 | 32 | diaminohydroxyphosphoribosylaminopyrimidine deaminase / | Metabolism | |
| K11753 | 31 | riboflavin kinase / FMN adenylyltransferase [EC:2.7.1.26 2.7.7.2] | Metabolism | |
| K11754 | 47 | dihydrofolate synthase / folylpolyglutamate synthase [EC:6.3.2.12 | Metabolism | |
| K11755 | 12 | phosphoribosyl-ATP pyrophosphohydrolase / phosphoribosyl-AMP | Metabolism | |
| K11779 | 20 | FO synthase [EC:2.5.1.-] | Metabolism | |
| K11781 | 1 | FO synthase subunit 2 [EC:2.5.1.-] | Metabolism | |
| K11782 | 5 | chorismate futalosine-lyase [EC:4.1.99.-] | Metabolism | |
| K11784 | 17 | de-hypoxanthine futalosine synthase [EC:1.21.-.-] | Metabolism | |
| K11785 | 2 | 1,4-dihydroxy-6-naphthoate synthase [EC:1.14.-.-] | Metabolism | |
| K11942 | 14 | methylmalonyl-CoA mutase [EC:5.4.99.2] | Metabolism | |
| K11948 | 2 | 1-hydroxy-2-naphthoate dioxygenase [EC:1.13.11.38] | Metabolism | |
| K11949 | 1 | 4-(2-carboxyphenyl)-2-oxobut-3-enoate aldolase [EC:4.1.2.34] | Metabolism | |
| K11987 | 1 | prostaglandin-endoperoxide synthase 2 [EC:1.14.99.1] | Metabolism | |
| K12073 | 3 | 1,4-dihydroxy-2-naphthoyl-CoA thioesterase [EC:3.1.2.-] | Metabolism | |
| K12111 | 2 | evolved beta-galactosidase subunit alpha [EC:3.2.1.23] | Metabolism | |
| K12234 | 9 | F420-0:gamma-glutamyl ligase [EC:6.3.2.-] | Metabolism | |
| K12238 | 5 | pyochelin biosynthesis protein PchD | Metabolism | |
| K12240 | 1 | pyochelin synthetase | Metabolism | |
| K12251 | 28 | N-carbamoylputrescine amidase [EC:3.5.1.53] | Metabolism | |
| K12252 | 2 | arginine:pyruvate transaminase [EC:2.6.1.84] | Metabolism | |
| K12253 | 4 | 5-guanidino-2-oxopentanoate decarboxylase [EC:4.1.1.75] | Metabolism | |
| K12254 | 3 | 4-guanidinobutyraldehyde dehydrogenase / NAD-dependent aldehyde | Metabolism | |
| K12255 | 4 | guanidinobutyrase [EC:3.5.3.7] | Metabolism | |
| K12256 | 7 | putrescine aminotransferase [EC:2.6.1.-] | Metabolism | |
| K12308 | 35 | beta-galactosidase [EC:3.2.1.23] | Metabolism | |
| K12339 | 16 | cysteine synthase B [EC:2.5.1.47] | Metabolism | |
| K12343 | 4 | 3-oxo-5-alpha-steroid 4-dehydrogenase 1 [EC:1.3.99.5] | Metabolism | |
| K12344 | 4 | 3-oxo-5-alpha-steroid 4-dehydrogenase 2 [EC:1.3.99.5] | Metabolism | |
| K12373 | 31 | beta-hexosaminidase [EC:3.2.1.52] | Metabolism | |
| K12420 | 16 | ketoacyl reductase [EC:1.3.1.-] | Metabolism | |
| K12452 | 6 | CDP-6-deoxy-D-xylo-4-hexulose-3-dehydrase | Metabolism | |
| K12454 | 22 | CDP-paratose 2-epimerase [EC:5.1.3.10] | Metabolism | |
| K12503 | 5 | short-chain Z-isoprenyl diphosphate synthase [EC:2.5.1.68] | Metabolism | |
| K12506 | 29 | 2-C-methyl-D-erythritol 4-phosphate cytidylyltransferase / | Metabolism | |
| K12524 | 14 | bifunctional aspartokinase/homoserine dehydrogenase 1 [EC:2.7.2.4 | Metabolism | |
| K12525 | 1 | bifunctional aspartokinase/homoserine dehydrogenase 2 [EC:2.7.2.4 | Metabolism | |
| K12526 | 1 | bifunctional diaminopimelate decarboxylase/aspartate kinase | Metabolism | |
| K12527 | 2 | putative selenate reductase [EC:1.97.1.9] | Metabolism | |
| K12528 | 2 | putative selenate reductase molybdopterin-binding subunit | Metabolism | |
| K12555 | 1 | penicillin-binding protein 2A [EC:2.4.1.129 2.3.2.-] | Metabolism | |
| K12645 | 13 | epi-isozizaene 5-monooxygenase [EC:1.14.13.106] | Metabolism | |
| K12658 | 5 | 4-hydroxyproline epimerase [EC:5.1.1.8] | Metabolism | |
| K12660 | 5 | 2-dehydro-3-deoxy-L-rhamnonate aldolase [EC:4.1.2.-] | Metabolism | |
| K12661 | 1 | L-rhamnonate dehydratase [EC:4.2.1.90] | Metabolism | |
| K12673 | 2 | N2-(2-carboxyethyl)arginine synthase [EC:2.5.1.66] | Metabolism | |
| K12960 | 17 | 5-methylthioadenosine/S-adenosylhomocysteine deaminase | Metabolism | |
| K12972 | 5 | gyoxylate/hydroxypyruvate reductase A [EC:1.1.1.79 1.1.1.81] | Metabolism | |

| | | | | |
|---|---|---|---|---|
| K13010 | 16 | perosamine synthetase | Metabolism | |
| K13014 | 2 | undecaprenyl phosphate-alpha-L-ara4FN deformylase [EC:3.5.-.-] | Metabolism | |
| K13015 | 28 | UDP-N-acetyl-D-glucosamine dehydrogenase [EC:1.1.1.-] | Metabolism | |
| K13016 | 1 | UDP-D-GlcNAcA oxidase [EC:1.1.1.-] | Metabolism | |
| K13017 | 16 | UDP-3-keto-D-GlcNAcA aminotransferase [EC:2.6.1.-] | Metabolism | |
| K13018 | 4 | UDP-D-GlcNAc3NA acetyltransferase [EC:2.3.1.-] | Metabolism | |
| K13019 | 9 | UDP-GlcNAc3NAcA epimerase [EC:5.1.3.23] | Metabolism | |
| K13020 | 21 | UDP-D-GlcNAcA oxidase [EC:1.1.1.-] | Metabolism | |
| K13038 | 42 | phosphopantothenoylcysteine decarboxylase / | Metabolism | |
| K13039 | 5 | sulfopyruvate decarboxylase subunit beta [EC:4.1.1.79] | Metabolism | |
| K13042 | 2 | dimethylglycine N-methyltransferase [EC:2.1.1.161] | Metabolism | |
| K13057 | 14 | trehalose synthase [EC:2.4.1.245] | Metabolism | |
| K13317 | 3 | NDP-4-keto-2,6-dideoxyhexose 3-C-methyltransferase | Metabolism | |
| K13318 | 6 | dTDP-4-keto-6-deoxy-L-hexose 4-reductase | Metabolism | |
| K13378 | 29 | NADH dehydrogenase I subunit C/D [EC:1.6.5.3] | Metabolism | |
| K13380 | 1 | NADH dehydrogenase I subunit B/C/D [EC:1.6.5.3] | Metabolism | |
| K13421 | 1 | uridine monophosphate synthetase [EC:2.4.2.10 4.1.1.23] | Metabolism | |
| K13479 | 1 | xanthine dehydrogenase FAD-binding subunit [EC:1.17.1.4] | Metabolism | |
| K13481 | 2 | xanthine dehydrogenase small subunit [EC:1.17.1.4] | Metabolism | |
| K13482 | 10 | xanthine dehydrogenase large subunit [EC:1.17.1.4] | Metabolism | |
| K13483 | 14 | xanthine dehydrogenase YagT iron-sulfur-binding subunit | Metabolism | |
| K13497 | 7 | anthranilate synthase/phosphoribosyltransferase [EC:4.1.3.27 | Metabolism | |
| K13498 | 7 | indole-3-glycerol phosphate synthase / phosphoribosylanthranilate | Metabolism | |
| K13500 | 1 | chondroitin synthase [EC:2.4.1.175 2.4.1.226] | Metabolism | |
| K13503 | 3 | anthranilate synthase [EC:4.1.3.27] | Metabolism | |
| K13522 | 2 | bifunctional NMN adenylyltransferase/nudix hydrolase [EC:2.7.7.1 | Metabolism | |
| K13540 | 2 | precorrin-2 C20-methyltransferase / precorrin-3B | Metabolism | |
| K13541 | 3 | cobalamin biosynthesis protein CbiG / precorrin-3B | Metabolism | |
| K13542 | 25 | uroporphyrinogen III methyltransferase / synthase [EC:2.1.1.107 | Metabolism | |
| K13543 | 6 | uroporphyrinogen III methyltransferase / synthase [EC:2.1.1.107 | Metabolism | |
| K13601 | 26 | bacteriochlorophyll C8 methyltransferase [EC:2.1.1.-] | Metabolism | |
| K13602 | 20 | bacteriochlorophyll C12 methyltransferase [EC:2.1.1.-] | Metabolism | |
| K13604 | 7 | bacteriochlorophyll C20 methyltransferase [EC:2.1.1.-] | Metabolism | |
| K13607 | 4 | cinnamoyl-CoA:phenyllactate CoA-transferase [EC:2.8.3.17] | Metabolism | |
| K13745 | 5 | L-2,4-diaminobutyrate decarboxylase [EC:4.1.1.86] | Metabolism | |
| K13746 | 1 | carboxynorspermidine dehydrogenase | Metabolism | |
| K13747 | 2 | carboxynorspermidine decarboxylase [EC:4.1.1.-] | Metabolism | |
| K13766 | 13 | methylglutaconyl-CoA hydratase [EC:4.2.1.18] | Metabolism | |
| K13767 | 5 | enoyl-CoA hydratase [EC:4.2.1.17] | Metabolism | |
| K13774 | 3 | citronellol/citronellal dehydrogenase | Metabolism | |
| K13775 | 5 | citronellol/citronellal dehydrogenase | Metabolism | |
| K13776 | 1 | citronellyl-CoA synthetase [EC:6.2.1.-] | Metabolism | |
| K13777 | 1 | geranyl-CoA carboxylase alpha subunit [EC:6.4.1.5] | Metabolism | |
| K13778 | 4 | geranyl-CoA carboxylase beta subunit [EC:6.4.1.5] | Metabolism | |
| K13787 | 9 | geranylgeranyl diphosphate synthase, type I [EC:2.5.1.1 2.5.1.10 | Metabolism | |
| K13788 | 5 | phosphate acetyltransferase [EC:2.3.1.8] | Metabolism | |
| K13789 | 15 | geranylgeranyl diphosphate synthase, type II [EC:2.5.1.1 2.5.1.10 | Metabolism | |
| K13797 | 2 | DNA-directed RNA polymerase subunit beta-beta' [EC:2.7.7.6] | Metabolism | |
| K13799 | 8 | pantoate ligase / cytidylate kinase [EC:6.3.2.1 2.7.4.14] | Metabolism | |
| K13810 | 14 | transaldolase / glucose-6-phosphate isomerase [EC:2.2.1.2 5.3.1.9] | Metabolism | |
| K13811 | 3 | 3'-phosphoadenosine 5'-phosphosulfate synthase [EC:2.7.7.4 2.7.1.25] | Metabolism | |
| K13821 | 28 | proline dehydrogenase / delta 1-pyrroline-5-carboxylate | Metabolism | |
| K13829 | 16 | shikimate kinase / 3-dehydroquinate synthase [EC:2.7.1.71 4.2.3.4] | Metabolism | |
| K13832 | 9 | 3-dehydroquinate dehydratase / shikimate dehydrogenase [EC:4.2.1.10 | Metabolism | |
| K13874 | 3 | L-arabinonolactonase [EC:3.1.1.15] | Metabolism | |
| K13875 | 7 | L-arabonate dehydrase [EC:4.2.1.25] | Metabolism | |
| K13876 | 5 | 2-keto-3-deoxy-L-arabinonate dehydratase [EC:4.2.1.43] | Metabolism | |

| | | | | |
|---|---|---|---|---|
| K13919 | 1 | propanediol dehydratase medium subunit [EC:4.2.1.28] | Metabolism | |
| K13922 | 1 | propionaldehyde dehydrogenase | Metabolism | |
| K13940 | 13 | dihydroneopterin aldolase / | Metabolism | |
| K13950 | 9 | para-aminobenzoate synthetase [EC:2.6.1.85] | Metabolism | |
| K13953 | 52 | alcohol dehydrogenase, propanol-preferring [EC:1.1.1.1] | Metabolism | |
| K13954 | 5 | alcohol dehydrogenase [EC:1.1.1.1] | Metabolism | |
| K13990 | 19 | glutamate formiminotransferase / formiminotetrahydrofolate | Metabolism | |
| K13995 | 2 | maleamate amidohydrolase [EC:3.5.1.107] | Metabolism | |

| KEGG ID | Sequences | KEGG pathway | KEGG category |
|---|---|---|---|
| K00021 | 14 | 3-hydroxy-3-methylglutaryl-CoA reductase [EC:1.1.1.34] | Environmental Information Processing |
| K00027 | 40 | malate dehydrogenase (oxaloacetate-decarboxylating) [EC:1.1.1.38] | Environmental Information Processing |
| K00066 | 25 | GDP-mannose 6-dehydrogenase [EC:1.1.1.132] | Environmental Information Processing |
| K00134 | 29 | glyceraldehyde 3-phosphate dehydrogenase [EC:1.2.1.12] | Environmental Information Processing |
| K00157 | 22 | aldehyde oxidase [EC:1.2.3.1] | Environmental Information Processing |
| K00161 | 65 | pyruvate dehydrogenase E1 component subunit alpha [EC:1.2.4.1] | Environmental Information Processing |
| K00162 | 66 | pyruvate dehydrogenase E1 component subunit beta [EC:1.2.4.1] | Environmental Information Processing |
| K00232 | 1 | acyl-CoA oxidase [EC:1.3.3.6] | Environmental Information Processing |
| K00244 | 39 | fumarate reductase flavoprotein subunit [EC:1.3.99.1] | Environmental Information Processing |
| K00245 | 7 | fumarate reductase iron-sulfur protein [EC:1.3.99.1] | Environmental Information Processing |
| K00246 | 13 | fumarate reductase subunit C [EC:1.3.99.1] | Environmental Information Processing |
| K00247 | 5 | fumarate reductase subunit D [EC:1.3.99.1] | Environmental Information Processing |
| K00370 | 34 | nitrate reductase 1, alpha subunit [EC:1.7.99.4] | Environmental Information Processing |
| K00371 | 12 | nitrate reductase 1, beta subunit [EC:1.7.99.4] | Environmental Information Processing |
| K00373 | 4 | nitrate reductase 1, delta subunit [EC:1.7.99.4] | Environmental Information Processing |
| K00374 | 4 | nitrate reductase 1, gamma subunit [EC:1.7.99.4] | Environmental Information Processing |
| K00404 | 16 | cb-type cytochrome c oxidase subunit I [EC:1.9.3.1] | Environmental Information Processing |
| K00405 | 17 | cb-type cytochrome c oxidase subunit II [EC:1.9.3.1] | Environmental Information Processing |
| K00406 | 34 | cb-type cytochrome c oxidase subunit III [EC:1.9.3.1] | Environmental Information Processing |
| K00407 | 6 | cb-type cytochrome c oxidase subunit IV [EC:1.9.3.1] | Environmental Information Processing |
| K00411 | 10 | ubiquinol-cytochrome c reductase iron-sulfur subunit [EC:1.10.2.2] | Environmental Information Processing |
| K00412 | 35 | ubiquinol-cytochrome c reductase cytochrome b subunit [EC:1.10.2.2] | Environmental Information Processing |
| K00413 | 8 | ubiquinol-cytochrome c reductase cytochrome c1 subunit [EC:1.10.2.2] | Environmental Information Processing |
| K00425 | 39 | cytochrome bd-I oxidase subunit I [EC:1.10.3.-] | Environmental Information Processing |
| K00426 | 23 | cytochrome bd-I oxidase subunit II [EC:1.10.3.-] | Environmental Information Processing |
| K00494 | 70 | alkanal monooxygenase (FMN-linked) [EC:1.14.14.3] | Environmental Information Processing |
| K00507 | 40 | stearoyl-CoA desaturase (delta-9 desaturase) [EC:1.14.19.1] | Environmental Information Processing |
| K00575 | 79 | chemotaxis protein methyltransferase CheR [EC:2.1.1.80] | Environmental Information Processing |
| K00626 | 211 | acetyl-CoA C-acetyltransferase [EC:2.3.1.9] | Environmental Information Processing |
| K00654 | 7 | serine palmitoyltransferase [EC:2.3.1.50] | Environmental Information Processing |
| K00693 | 14 | glycogen(starch) synthase [EC:2.4.1.11] | Environmental Information Processing |
| K00850 | 56 | 6-phosphofructokinase [EC:2.7.1.11] | Environmental Information Processing |
| K00901 | 6 | diacylglycerol kinase [EC:2.7.1.107] | Environmental Information Processing |
| K00981 | 27 | phosphatidate cytidylyltransferase [EC:2.7.7.41] | Environmental Information Processing |
| K00990 | 45 | [protein-PII] uridylyltransferase [EC:2.7.7.59] | Environmental Information Processing |
| K00999 | 4 | CDP-diacylglycerol--inositol 3-phosphatidyltransferase [EC:2.7.8.11] | Environmental Information Processing |
| K01035 | 2 | acetate CoA-transferase beta subunit [EC:2.8.3.8] | Environmental Information Processing |
| K01077 | 90 | alkaline phosphatase [EC:3.1.3.1] | Environmental Information Processing |
| K01092 | 58 | myo-inositol-1(or 4)-monophosphatase [EC:3.1.3.25] | Environmental Information Processing |
| K01103 | 1 | fructose-2,6-bisphosphatase [EC:3.1.3.46] | Environmental Information Processing |
| K01113 | 42 | phosphodiesterase/alkaline phosphatase D [EC:3.1.4.1] | Environmental Information Processing |
| K01115 | 8 | phospholipase D [EC:3.1.4.4] | Environmental Information Processing |
| K01312 | 9 | trypsin [EC:3.4.21.4] | Environmental Information Processing |
| K01467 | 153 | beta-lactamase [EC:3.5.2.6] | Environmental Information Processing |
| K01539 | 6 | sodium/potassium-transporting ATPase subunit alpha [EC:3.6.3.9] | Environmental Information Processing |
| K01546 | 18 | K+-transporting ATPase ATPase A chain [EC:3.6.3.12] | Environmental Information Processing |
| K01547 | 19 | K+-transporting ATPase ATPase B chain [EC:3.6.3.12] | Environmental Information Processing |
| K01548 | 14 | K+-transporting ATPase ATPase C chain [EC:3.6.3.12] | Environmental Information Processing |
| K01578 | 19 | malonyl-CoA decarboxylase [EC:4.1.1.9] | Environmental Information Processing |
| K01596 | 30 | phosphoenolpyruvate carboxykinase (GTP) [EC:4.1.1.32] | Environmental Information Processing |
| K01634 | 7 | sphinganine-1-phosphate aldolase [EC:4.1.2.27] | Environmental Information Processing |
| K01643 | 11 | citrate lyase subunit alpha / citrate CoA-transferase [EC:4.1.3.6 | Environmental Information Processing |
| K01644 | 42 | citrate lyase subunit beta / citryl-CoA lyase [EC:4.1.3.6 4.1.3.34] | Environmental Information Processing |
| K01689 | 42 | enolase [EC:4.2.1.11] | Environmental Information Processing |
| K01915 | 141 | glutamine synthetase [EC:6.3.1.2] | Environmental Information Processing |
| K01995 | 126 | branched-chain amino acid transport system ATP-binding protein | Environmental Information Processing |
| K01996 | 125 | branched-chain amino acid transport system ATP-binding protein | Environmental Information Processing |
| K01997 | 153 | branched-chain amino acid transport system permease protein | Environmental Information Processing |
| K01998 | 326 | branched-chain amino acid transport system permease protein | Environmental Information Processing |
| K01999 | 254 | branched-chain amino acid transport system substrate-binding protein | Environmental Information Processing |
| K02000 | 5 | glycine betaine/proline transport system ATP-binding protein | Environmental Information Processing |
| K02001 | 2 | glycine betaine/proline transport system permease protein | Environmental Information Processing |
| K02002 | 2 | glycine betaine/proline transport system substrate-binding protein | Environmental Information Processing |
| K02006 | 28 | cobalt/nickel transport system ATP-binding protein | Environmental Information Processing |
| K02007 | 5 | cobalt/nickel transport system permease protein | Environmental Information Processing |
| K02008 | 17 | cobalt/nickel transport system permease protein | Environmental Information Processing |
| K02010 | 19 | iron(III) transport system ATP-binding protein [EC:3.6.3.30] | Environmental Information Processing |
| K02011 | 71 | iron(III) transport system permease protein | Environmental Information Processing |
| K02012 | 46 | iron(III) transport system substrate-binding protein | Environmental Information Processing |

| | | | | |
|---|---|---|---|---|
| K02013 | 25 | iron complex transport system ATP-binding protein [EC:3.6.3.34] | Environmental Information Processing | |
| K02015 | 23 | iron complex transport system permease protein | Environmental Information Processing | |
| K02016 | 43 | iron complex transport system substrate-binding protein | Environmental Information Processing | |
| K02017 | 11 | molybdate transport system ATP-binding protein [EC:3.6.3.29] | Environmental Information Processing | |
| K02018 | 15 | molybdate transport system permease protein | Environmental Information Processing | |
| K02020 | 55 | molybdate transport system substrate-binding protein | Environmental Information Processing | |
| K02036 | 24 | phosphate transport system ATP-binding protein [EC:3.6.3.27] | Environmental Information Processing | |
| K02037 | 43 | phosphate transport system permease protein | Environmental Information Processing | |
| K02038 | 42 | phosphate transport system permease protein | Environmental Information Processing | |
| K02040 | 62 | phosphate transport system substrate-binding protein | Environmental Information Processing | |
| K02041 | 11 | phosphonate transport system ATP-binding protein | Environmental Information Processing | |
| K02042 | 4 | phosphonate transport system permease protein | Environmental Information Processing | |
| K02044 | 24 | phosphonate transport system substrate-binding protein | Environmental Information Processing | |
| K02045 | 11 | sulfate transport system ATP-binding protein [EC:3.6.3.25] | Environmental Information Processing | |
| K02046 | 14 | sulfate transport system permease protein | Environmental Information Processing | |
| K02047 | 9 | sulfate transport system permease protein | Environmental Information Processing | |
| K02048 | 16 | sulfate transport system substrate-binding protein | Environmental Information Processing | |
| K02063 | 7 | thiamine transport system permease protein | Environmental Information Processing | |
| K02064 | 3 | thiamine transport system substrate-binding protein | Environmental Information Processing | |
| K02065 | 37 | putative ABC transport system ATP-binding protein | Environmental Information Processing | |
| K02066 | 51 | putative ABC transport system permease protein | Environmental Information Processing | |
| K02067 | 57 | putative ABC transport system substrate-binding protein | Environmental Information Processing | |
| K02071 | 4 | D-methionine transport system ATP-binding protein | Environmental Information Processing | |
| K02073 | 2 | D-methionine transport system substrate-binding protein | Environmental Information Processing | |
| K02106 | 4 | short-chain fatty acids transporter | Environmental Information Processing | |
| K02193 | 29 | heme exporter protein A [EC:3.6.3.41] | Environmental Information Processing | |
| K02194 | 15 | heme exporter protein B | Environmental Information Processing | |
| K02195 | 42 | heme exporter protein C | Environmental Information Processing | |
| K02259 | 27 | cytochrome c oxidase subunit XV assembly protein | Environmental Information Processing | |
| K02313 | 51 | chromosomal replication initiator protein | Environmental Information Processing | |
| K02402 | 1 | flagellar transcriptional activator FlhC | Environmental Information Processing | |
| K02405 | 21 | RNA polymerase sigma factor for flagellar operon FliA | Environmental Information Processing | |
| K02406 | 10 | flagellin | Environmental Information Processing | |
| K02424 | 2 | cystine transport system substrate-binding protein | Environmental Information Processing | |
| K02452 | 1 | general secretion pathway protein C | Environmental Information Processing | |
| K02453 | 90 | general secretion pathway protein D | Environmental Information Processing | |
| K02454 | 48 | general secretion pathway protein E | Environmental Information Processing | |
| K02455 | 102 | general secretion pathway protein F | Environmental Information Processing | |
| K02456 | 16 | general secretion pathway protein G | Environmental Information Processing | |
| K02457 | 2 | general secretion pathway protein H | Environmental Information Processing | |
| K02458 | 2 | general secretion pathway protein I | Environmental Information Processing | |
| K02459 | 2 | general secretion pathway protein J | Environmental Information Processing | |
| K02460 | 10 | general secretion pathway protein K | Environmental Information Processing | |
| K02461 | 7 | general secretion pathway protein L | Environmental Information Processing | |
| K02464 | 6 | general secretion pathway protein O [EC:3.4.23.43 2.1.1.-] | Environmental Information Processing | |
| K02471 | 16 | putative ATP-binding cassette transporter | Environmental Information Processing | |
| K02487 | 1 | type IV pili sensor histidine kinase and response regulator | Environmental Information Processing | |
| K02488 | 217 | two-component system, cell cycle response regulator | Environmental Information Processing | |
| K02489 | 49 | two-component system, cell cycle sensor kinase and response | Environmental Information Processing | |
| K02490 | 30 | two-component system, response regulator, stage 0 sporulation | Environmental Information Processing | |
| K02491 | 8 | two-component system, sporulation sensor kinase A [EC:2.7.13.3] | Environmental Information Processing | |
| K02556 | 5 | chemotaxis protein MotA | Environmental Information Processing | |
| K02584 | 38 | Nif-specific regulatory protein | Environmental Information Processing | |
| K02650 | 7 | type IV pilus assembly protein PilA | Environmental Information Processing | |
| K02657 | 13 | twitching motility two-component system response regulator PilG | Environmental Information Processing | |
| K02658 | 37 | twitching motility two-component system response regulator PilH | Environmental Information Processing | |
| K02659 | 5 | twitching motility protein PilI | Environmental Information Processing | |
| K02660 | 18 | twitching motility protein PilJ | Environmental Information Processing | |
| K02661 | 2 | type IV pilus assembly protein PilK | Environmental Information Processing | |
| K02667 | 64 | two-component system, NtrC family, response regulator PilR | Environmental Information Processing | |
| K02668 | 66 | two-component system, NtrC family, sensor histidine kinase PilS | Environmental Information Processing | |
| K02745 | 1 | PTS system, N-acetylgalactosamine-specific IIB component | Environmental Information Processing | |
| K02755 | 1 | PTS system, beta-glucosides-specific IIA component [EC:2.7.1.69] | Environmental Information Processing | |
| K02759 | 4 | PTS system, cellobiose-specific IIA component [EC:2.7.1.69] | Environmental Information Processing | |
| K02761 | 9 | PTS system, cellobiose-specific IIC component | Environmental Information Processing | |
| K02765 | 3 | PTS system, D-glucosamine-specific IIC component | Environmental Information Processing | |
| K02768 | 6 | PTS system, fructose-specific IIA component [EC:2.7.1.69] | Environmental Information Processing | |
| K02769 | 1 | PTS system, fructose-specific IIB component [EC:2.7.1.69] | Environmental Information Processing | |
| K02770 | 12 | PTS system, fructose-specific IIC component | Environmental Information Processing | |

| | | | | |
|---|---|---|---|---|
| K02774 | 5 | PTS system, galactitol-specific IIB component [EC:2.7.1.69] | Environmental Information Processing | |
| K02775 | 2 | PTS system, galactitol-specific IIC component | Environmental Information Processing | |
| K02777 | 109 | PTS system, glucose-specific IIA component [EC:2.7.1.69] | Environmental Information Processing | |
| K02779 | 2 | PTS system, glucose-specific IIC component | Environmental Information Processing | |
| K02784 | 4 | phosphocarrier protein HPr | Environmental Information Processing | |
| K02793 | 6 | PTS system, mannose-specific IIA component [EC:2.7.1.69] | Environmental Information Processing | |
| K02794 | 3 | PTS system, mannose-specific IIB component [EC:2.7.1.69] | Environmental Information Processing | |
| K02795 | 3 | PTS system, mannose-specific IIC component | Environmental Information Processing | |
| K02796 | 6 | PTS system, mannose-specific IID component | Environmental Information Processing | |
| K02799 | 2 | PTS system, mannitol-specific IIB component [EC:2.7.1.69] | Environmental Information Processing | |
| K02800 | 3 | PTS system, mannitol-specific IIC component | Environmental Information Processing | |
| K02804 | 1 | PTS system, N-acetylglucosamine-specific IIC component | Environmental Information Processing | |
| K02806 | 15 | PTS system, nitrogen regulatory IIA component [EC:2.7.1.69] | Environmental Information Processing | |
| K02821 | 9 | PTS system, ascorbate-specific IIA component [EC:2.7.1.69] | Environmental Information Processing | |
| K03070 | 74 | preprotein translocase subunit SecA | Environmental Information Processing | |
| K03071 | 7 | preprotein translocase subunit SecB | Environmental Information Processing | |
| K03072 | 476 | preprotein translocase subunit SecD | Environmental Information Processing | |
| K03073 | 2 | preprotein translocase subunit SecE | Environmental Information Processing | |
| K03074 | 41 | preprotein translocase subunit SecF | Environmental Information Processing | |
| K03075 | 3 | preprotein translocase subunit SecG | Environmental Information Processing | |
| K03076 | 36 | preprotein translocase subunit SecY | Environmental Information Processing | |
| K03092 | 47 | RNA polymerase sigma-54 factor | Environmental Information Processing | |
| K03106 | 47 | signal recognition particle subunit SRP54 | Environmental Information Processing | |
| K03110 | 23 | fused signal recognition particle receptor | Environmental Information Processing | |
| K03116 | 10 | sec-independent protein translocase protein TatA | Environmental Information Processing | |
| K03117 | 4 | sec-independent protein translocase protein TatB | Environmental Information Processing | |
| K03118 | 57 | sec-independent protein translocase protein TatC | Environmental Information Processing | |
| K03196 | 1 | type IV secretion system protein VirB11 | Environmental Information Processing | |
| K03197 | 2 | type IV secretion system protein VirB2 | Environmental Information Processing | |
| K03198 | 1 | type IV secretion system protein VirB3 | Environmental Information Processing | |
| K03199 | 8 | type IV secretion system protein VirB4 | Environmental Information Processing | |
| K03200 | 1 | type IV secretion system protein VirB5 | Environmental Information Processing | |
| K03201 | 2 | type IV secretion system protein VirB6 | Environmental Information Processing | |
| K03205 | 7 | type IV secretion system protein VirD4 | Environmental Information Processing | |
| K03210 | 16 | preprotein translocase subunit YajC | Environmental Information Processing | |
| K03217 | 71 | preprotein translocase subunit YidC | Environmental Information Processing | |
| K03219 | 1 | type III secretion protein SctC | Environmental Information Processing | |
| K03221 | 1 | type III secretion protein SctF | Environmental Information Processing | |
| K03222 | 3 | type III secretion protein SctJ | Environmental Information Processing | |
| K03223 | 1 | type III secretion protein SctL | Environmental Information Processing | |
| K03224 | 1 | ATP synthase in type III secretion protein SctN [EC:3.6.3.14] | Environmental Information Processing | |
| K03225 | 2 | type III secretion protein SctQ | Environmental Information Processing | |
| K03227 | 2 | type III secretion protein SctS | Environmental Information Processing | |
| K03228 | 2 | type III secretion protein SctT | Environmental Information Processing | |
| K03229 | 1 | type III secretion protein SctU | Environmental Information Processing | |
| K03230 | 2 | type III secretion protein SctV | Environmental Information Processing | |
| K03367 | 32 | D-alanine-poly(phosphoribitol) ligase [EC:6.1.1.13] | Environmental Information Processing | |
| K03406 | 186 | methyl-accepting chemotaxis protein | Environmental Information Processing | |
| K03407 | 82 | two-component system, chemotaxis family, sensor kinase CheA | Environmental Information Processing | |
| K03408 | 21 | purine-binding chemotaxis protein CheW | Environmental Information Processing | |
| K03412 | 64 | two-component system, chemotaxis family, response regulator CheB | Environmental Information Processing | |
| K03413 | 117 | two-component system, chemotaxis family, response regulator CheY | Environmental Information Processing | |
| K03475 | 1 | PTS system, ascorbate-specific IIC component | Environmental Information Processing | |
| K03523 | 5 | putative biotin biosynthesis protein BioY | Environmental Information Processing | |
| K03532 | 3 | trimethylamine-N-oxide reductase (cytochrome c) 1, cytochrome c-type | Environmental Information Processing | |
| K03563 | 5 | carbon storage regulator | Environmental Information Processing | |
| K03620 | 12 | Ni/Fe-hydrogenase 1 B-type cytochrome subunit | Environmental Information Processing | |
| K03740 | 1 | D-alanine transfer protein | Environmental Information Processing | |
| K03776 | 16 | aerotaxis receptor | Environmental Information Processing | |
| K03781 | 12 | catalase [EC:1.11.1.6] | Environmental Information Processing | |
| K03841 | 11 | fructose-1,6-bisphosphatase I [EC:3.1.3.11] | Environmental Information Processing | |
| K04079 | 17 | molecular chaperone HtpG | Environmental Information Processing | |
| K04496 | 9 | C-terminal binding protein | Environmental Information Processing | |
| K04564 | 27 | superoxide dismutase, Fe-Mn family [EC:1.15.1.1] | Environmental Information Processing | |
| K04751 | 13 | nitrogen regulatory protein P-II 1 | Environmental Information Processing | |
| K04771 | 6 | serine protease Do [EC:3.4.21.107] | Environmental Information Processing | |
| K05338 | 12 | holin-like protein | Environmental Information Processing | |
| K05339 | 1 | holin-like protein LrgB | Environmental Information Processing | |
| K05597 | 1 | glutamin-(asparagin-)ase [EC:3.5.1.38] | Environmental Information Processing | |

| | | | | |
|---|---|---|---|---|
| K05685 | 60 | macrolide transport system ATP-binding/permease protein [EC:3.6.3.-] | Environmental Information Processing | |
| K05692 | 2 | actin beta/gamma 1 | Environmental Information Processing | |
| K05772 | 8 | putative tungstate transport system substrate-binding protein | Environmental Information Processing | |
| K05773 | 8 | putative tungstate transport system permease protein | Environmental Information Processing | |
| K05776 | 4 | molybdate transport system ATP-binding protein | Environmental Information Processing | |
| K05813 | 23 | sn-glycerol 3-phosphate transport system substrate-binding protein | Environmental Information Processing | |
| K05814 | 17 | sn-glycerol 3-phosphate transport system permease protein | Environmental Information Processing | |
| K05815 | 10 | sn-glycerol 3-phosphate transport system permease protein | Environmental Information Processing | |
| K05816 | 7 | sn-glycerol 3-phosphate transport system ATP-binding protein | Environmental Information Processing | |
| K05845 | 22 | osmoprotectant transport system substrate-binding protein | Environmental Information Processing | |
| K05846 | 27 | osmoprotectant transport system permease protein | Environmental Information Processing | |
| K05847 | 6 | osmoprotectant transport system ATP-binding protein | Environmental Information Processing | |
| K05874 | 21 | methyl-accepting chemotaxis protein I, serine sensor receptor | Environmental Information Processing | |
| K05875 | 8 | methyl-accepting chemotaxis protein II, aspartate sensor receptor | Environmental Information Processing | |
| K05876 | 2 | methyl-accepting chemotaxis protein III, ribose and galactose sensor | Environmental Information Processing | |
| K06596 | 35 | chemosensory pili system protein ChpA (sensor histidine | Environmental Information Processing | |
| K06726 | 1 | D-ribose pyranase [EC:5.-.-.-] | Environmental Information Processing | |
| K06857 | 10 | putative tungstate transport system ATP-binding protein | Environmental Information Processing | |
| K06858 | 8 | vitamin B12 transport system substrate-binding protein | Environmental Information Processing | |
| K06861 | 16 | lipopolysaccharide export system ATP-binding protein [EC:3.6.3.-] | Environmental Information Processing | |
| K07091 | 25 | lipopolysaccharide export system permease protein | Environmental Information Processing | |
| K07122 | 1 | Unclassified; K07122 | Environmental Information Processing | |
| K07260 | 7 | D-alanyl-D-alanine carboxypeptidase [EC:3.4.16.4] | Environmental Information Processing | |
| K07323 | 15 | putative toluene tolerance protein | Environmental Information Processing | |
| K07636 | 65 | two-component system, OmpR family, phosphate regulon sensor | Environmental Information Processing | |
| K07637 | 1 | two-component system, OmpR family, sensor histidine kinase PhoQ | Environmental Information Processing | |
| K07638 | 12 | two-component system, OmpR family, osmolarity sensor histidine | Environmental Information Processing | |
| K07639 | 2 | two-component system, OmpR family, sensor histidine kinase RstB | Environmental Information Processing | |
| K07640 | 6 | two-component system, OmpR family, sensor histidine kinase CpxA | Environmental Information Processing | |
| K07641 | 4 | two-component system, OmpR family, sensor histidine kinase CreC | Environmental Information Processing | |
| K07642 | 11 | two-component system, OmpR family, sensor histidine kinase BaeS | Environmental Information Processing | |
| K07643 | 3 | two-component system, OmpR family, sensor histidine kinase BasS | Environmental Information Processing | |
| K07644 | 26 | two-component system, OmpR family, heavy metal sensor histidine | Environmental Information Processing | |
| K07645 | 33 | two-component system, OmpR family, sensor histidine kinase QseC | Environmental Information Processing | |
| K07646 | 67 | two-component system, OmpR family, sensor histidine kinase KdpD | Environmental Information Processing | |
| K07647 | 4 | two-component system, OmpR family, sensor histidine kinase TorS | Environmental Information Processing | |
| K07648 | 1 | two-component system, OmpR family, aerobic respiration control | Environmental Information Processing | |
| K07649 | 29 | two-component system, OmpR family, sensor histidine kinase TctE | Environmental Information Processing | |
| K07651 | 11 | two-component system, OmpR family, sensor histidine kinase ResE | Environmental Information Processing | |
| K07652 | 42 | two-component system, OmpR family, sensor histidine kinase VicK | Environmental Information Processing | |
| K07653 | 6 | two-component system, OmpR family, sensor histidine kinase MprB | Environmental Information Processing | |
| K07654 | 5 | two-component system, OmpR family, sensor histidine kinase MtrB | Environmental Information Processing | |
| K07656 | 1 | two-component system, OmpR family, sensor histidine kinase TrcS | Environmental Information Processing | |
| K07657 | 68 | two-component system, OmpR family, phosphate regulon response | Environmental Information Processing | |
| K07658 | 67 | two-component system, OmpR family, alkaline phosphatase synthesis | Environmental Information Processing | |
| K07659 | 12 | two-component system, OmpR family, phosphate regulon response | Environmental Information Processing | |
| K07660 | 1 | two-component system, OmpR family, response regulator PhoP | Environmental Information Processing | |
| K07661 | 1 | two-component system, OmpR family, response regulator RstA | Environmental Information Processing | |
| K07662 | 5 | two-component system, OmpR family, response regulator CpxR | Environmental Information Processing | |
| K07663 | 1 | two-component system, OmpR family, catabolic regulation response | Environmental Information Processing | |
| K07664 | 5 | two-component system, OmpR family, response regulator BaeR | Environmental Information Processing | |
| K07665 | 23 | two-component system, OmpR family, copper resistance phosphate | Environmental Information Processing | |
| K07666 | 3 | two-component system, OmpR family, response regulator QseB | Environmental Information Processing | |
| K07667 | 53 | two-component system, OmpR family, KDP operon response regulator | Environmental Information Processing | |
| K07668 | 44 | two-component system, OmpR family, response regulator VicR | Environmental Information Processing | |
| K07669 | 30 | two-component system, OmpR family, response regulator MprA | Environmental Information Processing | |
| K07670 | 18 | two-component system, OmpR family, response regulator MtrA | Environmental Information Processing | |
| K07671 | 1 | two-component system, OmpR family, response regulator PrrA | Environmental Information Processing | |
| K07672 | 1 | two-component system, OmpR family, response regulator TrcR | Environmental Information Processing | |
| K07673 | 10 | two-component system, NarL family, nitrate/nitrite sensor histidine | Environmental Information Processing | |
| K07674 | 4 | two-component system, NarL family, nitrate/nitrite sensor histidine | Environmental Information Processing | |
| K07675 | 49 | two-component system, NarL family, sensor histidine kinase UhpB | Environmental Information Processing | |
| K07677 | 11 | two-component system, NarL family, capsular synthesis sensor | Environmental Information Processing | |
| K07678 | 26 | two-component system, NarL family, sensor histidine kinase BarA | Environmental Information Processing | |
| K07679 | 4 | two-component system, NarL family, sensor histidine kinase EvgS | Environmental Information Processing | |
| K07680 | 15 | two-component system, NarL family, sensor histidine kinase ComP | Environmental Information Processing | |
| K07681 | 1 | two-component system, NarL family, vancomycin resistance sensor | Environmental Information Processing | |
| K07682 | 15 | two-component system, NarL family, sensor histidine kinase DevS | Environmental Information Processing | |
| K07683 | 4 | two-component system, NarL family, sensor histidine kinase NreB | Environmental Information Processing | |

| | | | | |
|---|---|---|---|---|
| K07684 | 73 | two-component system, NarL family, nitrate/nitrite response | Environmental Information Processing | |
| K07685 | 6 | two-component system, NarL family, nitrate/nitrite response | Environmental Information Processing | |
| K07686 | 7 | two-component system, NarL family, uhpT operon response regulator | Environmental Information Processing | |
| K07687 | 9 | two-component system, NarL family, captular synthesis response | Environmental Information Processing | |
| K07689 | 20 | two-component system, NarL family, invasion response regulator UvrY | Environmental Information Processing | |
| K07690 | 5 | two-component system, NarL family, response regulator EvgA | Environmental Information Processing | |
| K07691 | 24 | two-component system, NarL family, competent response regulator ComA | Environmental Information Processing | |
| K07692 | 37 | two-component system, NarL family, response regulator DegU | Environmental Information Processing | |
| K07693 | 3 | two-component system, NarL family, response regulator DesR | Environmental Information Processing | |
| K07694 | 4 | two-component system, NarL family, vancomycin resistance associated | Environmental Information Processing | |
| K07695 | 13 | two-component system, NarL family, response regulator DevR | Environmental Information Processing | |
| K07696 | 4 | two-component system, NarL family, response regulator NreC | Environmental Information Processing | |
| K07697 | 11 | two-component system, sporulation sensor kinase B [EC:2.7.13.3] | Environmental Information Processing | |
| K07699 | 1 | two-component system, response regulator, stage 0 sporulation | Environmental Information Processing | |
| K07700 | 2 | two-component system, CitB family, cit operon sensor histidine | Environmental Information Processing | |
| K07703 | 1 | two-component system, CitB family, response regulator DcuR | Environmental Information Processing | |
| K07704 | 7 | two-component system, LytT family, sensor histidine kinase LytS | Environmental Information Processing | |
| K07705 | 5 | two-component system, LytT family, response regulator LytT | Environmental Information Processing | |
| K07707 | 4 | two-component system, AgrA family, response regulator AgrA | Environmental Information Processing | |
| K07708 | 54 | two-component system, NtrC family, nitrogen regulation sensor | Environmental Information Processing | |
| K07709 | 54 | two-component system, NtrC family, sensor histidine kinase HydH | Environmental Information Processing | |
| K07710 | 39 | two-component system, NtrC family, sensor histidine kinase AtoS | Environmental Information Processing | |
| K07711 | 10 | two-component system, NtrC family, sensor histidine kinase YfhK | Environmental Information Processing | |
| K07712 | 112 | two-component system, NtrC family, nitrogen regulation response | Environmental Information Processing | |
| K07713 | 64 | two-component system, NtrC family, response regulator HydG | Environmental Information Processing | |
| K07714 | 48 | two-component system, NtrC family, response regulator AtoC | Environmental Information Processing | |
| K07715 | 78 | two-component system, NtrC family, response regulator YfhA | Environmental Information Processing | |
| K07716 | 16 | two-component system, cell cycle sensor histidine kinase PleC | Environmental Information Processing | |
| K07718 | 1 | two-component system, sensor histidine kinase YesM [EC:2.7.13.3] | Environmental Information Processing | |
| K07720 | 7 | two-component system, response regulator YesN | Environmental Information Processing | |
| K07768 | 3 | two-component system, OmpR family, sensor histidine kinase SenX3 | Environmental Information Processing | |
| K07769 | 5 | two-component system, OmpR family, sensor histidine kinase NblS | Environmental Information Processing | |
| K07771 | 1 | two-component system, OmpR family, response regulator BasR | Environmental Information Processing | |
| K07773 | 1 | two-component system, OmpR family, aerobic respiration control | Environmental Information Processing | |
| K07774 | 14 | two-component system, OmpR family, response regulator TctD | Environmental Information Processing | |
| K07775 | 5 | two-component system, OmpR family, response regulator ResD | Environmental Information Processing | |
| K07776 | 43 | two-component system, OmpR family, response regulator RegX3 | Environmental Information Processing | |
| K07777 | 14 | two-component system, NarL family, sensor histidine kinase DegS | Environmental Information Processing | |
| K07778 | 10 | two-component system, NarL family, sensor histidine kinase DesK | Environmental Information Processing | |
| K07782 | 1 | LuxR family transcriptional regulator | Environmental Information Processing | |
| K07783 | 2 | MFS transporter, OPA family, sugar phosphate sensor protein UhpC | Environmental Information Processing | |
| K07785 | 1 | MFS transporter, NRE family, putaive nickel resistance protein | Environmental Information Processing | |
| K07787 | 112 | Cu(I)/Ag(I) efflux system membrane protein CusA | Environmental Information Processing | |
| K07788 | 66 | RND superfamily, multidrug transport protein MdtB | Environmental Information Processing | |
| K07789 | 37 | RND superfamily, multidrug transport protein MdtC | Environmental Information Processing | |
| K07790 | 1 | putative membrane protein PagO | Environmental Information Processing | |
| K07792 | 4 | anaerobic C4-dicarboxylate transporter DcuB | Environmental Information Processing | |
| K07793 | 90 | putative tricarboxylic transport membrane protein | Environmental Information Processing | |
| K07795 | 87 | putative tricarboxylic transport membrane protein | Environmental Information Processing | |
| K07796 | 16 | Cu(I)/Ag(I) efflux system outer membrane protein CusC | Environmental Information Processing | |
| K07798 | 51 | Cu(I)/Ag(I) efflux system membrane protein CusB | Environmental Information Processing | |
| K07799 | 33 | putative multidrug efflux transporter MdtA | Environmental Information Processing | |
| K07805 | 1 | putative membrane protein PagD | Environmental Information Processing | |
| K07806 | 20 | UDP-4-amino-4-deoxy-L-arabinose-oxoglutarate aminotransferase | Environmental Information Processing | |
| K07811 | 3 | trimethylamine-N-oxide reductase (cytochrome c) 1 [EC:1.7.2.3] | Environmental Information Processing | |
| K08082 | 4 | two-component system, LytT family, sensor histidine kinase AlgZ | Environmental Information Processing | |
| K08083 | 11 | two-component system, LytT family, response regulator AlgR | Environmental Information Processing | |
| K08348 | 39 | formate dehydrogenase-N, alpha subunit [EC:1.2.1.2] | Environmental Information Processing | |
| K08357 | 9 | tetrathionate reductase subunit A | Environmental Information Processing | |
| K08358 | 27 | tetrathionate reductase subunit B | Environmental Information Processing | |
| K08359 | 4 | tetrathionate reductase subunit C | Environmental Information Processing | |
| K08372 | 45 | putative serine protease PepD [EC:3.4.21.-] | Environmental Information Processing | |
| K08477 | 2 | outer membrane protease E [EC:3.4.21.-] | Environmental Information Processing | |
| K08478 | 1 | phosphoglycerate transport regulatory protein PgtC | Environmental Information Processing | |
| K08479 | 3 | two-component system, OmpR family, clock-associated histidine kinase | Environmental Information Processing | |
| K08483 | 32 | phosphotransferase system, enzyme I, PtsI [EC:2.7.3.9] | Environmental Information Processing | |
| K08484 | 7 | phosphotransferase system, enzyme I, PtsP [EC:2.7.3.9] | Environmental Information Processing | |
| K08485 | 1 | phosphocarrier protein NPr | Environmental Information Processing | |
| K08641 | 17 | D-alanyl-D-alanine dipeptidase [EC:3.4.13.-] | Environmental Information Processing | |

| | | | | |
|---|---|---|---|---|
| K08738 | 12 | cytochrome c | Environmental Information Processing | |
| K09474 | 2 | acid phosphatase (class A) [EC:3.1.3.2] | Environmental Information Processing | |
| K09477 | 5 | citrate:succinate antiporter | Environmental Information Processing | |
| K09688 | 3 | capsular polysaccharide transport system permease protein | Environmental Information Processing | |
| K09689 | 1 | capsular polysaccharide transport system ATP-binding protein | Environmental Information Processing | |
| K09690 | 46 | lipopolysaccharide transport system permease protein | Environmental Information Processing | |
| K09691 | 27 | lipopolysaccharide transport system ATP-binding protein | Environmental Information Processing | |
| K09693 | 8 | teichoic acid transport system ATP-binding protein [EC:3.6.3.40] | Environmental Information Processing | |
| K09694 | 20 | lipooligosaccharide transport system permease protein | Environmental Information Processing | |
| K09695 | 6 | lipooligosaccharide transport system ATP-binding protein | Environmental Information Processing | |
| K09697 | 5 | sodium transport system ATP-binding protein | Environmental Information Processing | |
| K09808 | 44 | lipoprotein-releasing system permease protein | Environmental Information Processing | |
| K09810 | 59 | lipoprotein-releasing system ATP-binding protein [EC:3.6.3.-] | Environmental Information Processing | |
| K09811 | 32 | cell division transport system permease protein | Environmental Information Processing | |
| K09812 | 13 | cell division transport system ATP-binding protein | Environmental Information Processing | |
| K09815 | 10 | zinc transport system substrate-binding protein | Environmental Information Processing | |
| K09816 | 9 | zinc transport system permease protein | Environmental Information Processing | |
| K09817 | 8 | zinc transport system ATP-binding protein [EC:3.6.3.-] | Environmental Information Processing | |
| K09969 | 7 | general L-amino acid transport system substrate-binding protein | Environmental Information Processing | |
| K09970 | 2 | general L-amino acid transport system permease protein | Environmental Information Processing | |
| K09971 | 7 | general L-amino acid transport system permease protein | Environmental Information Processing | |
| K09972 | 2 | general L-amino acid transport system ATP-binding protein | Environmental Information Processing | |
| K09996 | 9 | arginine transport system substrate-binding protein | Environmental Information Processing | |
| K09999 | 2 | arginine transport system permease protein | Environmental Information Processing | |
| K10001 | 14 | glutamate/aspartate transport system substrate-binding protein | Environmental Information Processing | |
| K10002 | 5 | glutamate/aspartate transport system permease protein | Environmental Information Processing | |
| K10003 | 8 | glutamate/aspartate transport system permease protein | Environmental Information Processing | |
| K10004 | 3 | glutamate/aspartate transport system ATP-binding protein | Environmental Information Processing | |
| K10005 | 1 | glutamate transport system substrate-binding protein | Environmental Information Processing | |
| K10006 | 2 | glutamate transport system permease protein | Environmental Information Processing | |
| K10007 | 2 | glutamate transport system permease protein | Environmental Information Processing | |
| K10009 | 4 | cystine transport system permease protein | Environmental Information Processing | |
| K10010 | 1 | cystine transport system ATP-binding protein [EC:3.6.3.-] | Environmental Information Processing | |
| K10014 | 1 | histidine transport system substrate-binding protein | Environmental Information Processing | |
| K10017 | 1 | histidine transport system ATP-binding protein [EC:3.6.3.21] | Environmental Information Processing | |
| K10036 | 2 | glutamine transport system substrate-binding protein | Environmental Information Processing | |
| K10037 | 3 | glutamine transport system permease protein | Environmental Information Processing | |
| K10039 | 6 | putative glutamine transport system substrate-binding protein | Environmental Information Processing | |
| K10040 | 3 | putative glutamine transport system permease protein | Environmental Information Processing | |
| K10041 | 2 | putative glutamine transport system ATP-binding protein [EC:3.6.3.-] | Environmental Information Processing | |
| K10108 | 11 | maltose/maltodextrin transport system substrate-binding protein | Environmental Information Processing | |
| K10109 | 5 | maltose/maltodextrin transport system permease protein | Environmental Information Processing | |
| K10110 | 13 | maltose/maltodextrin transport system permease protein | Environmental Information Processing | |
| K10111 | 2 | maltose/maltodextrin transport system ATP-binding protein | Environmental Information Processing | |
| K10112 | 10 | maltose/maltodextrin transport system ATP-binding protein | Environmental Information Processing | |
| K10117 | 1 | multiple sugar transport system substrate-binding protein | Environmental Information Processing | |
| K10118 | 3 | multiple sugar transport system permease protein | Environmental Information Processing | |
| K10119 | 3 | multiple sugar transport system permease protein | Environmental Information Processing | |
| K10125 | 16 | two-component system, NtrC family, C4-dicarboxylate transport sensor | Environmental Information Processing | |
| K10126 | 65 | two-component system, NtrC family, C4-dicarboxylate transport | Environmental Information Processing | |
| K10188 | 9 | lactose/L-arabinose transport system substrate-binding protein | Environmental Information Processing | |
| K10189 | 8 | lactose/L-arabinose transport system permease protein | Environmental Information Processing | |
| K10190 | 19 | lactose/L-arabinose transport system permease protein | Environmental Information Processing | |
| K10191 | 1 | lactose/L-arabinose transport system ATP-binding protein | Environmental Information Processing | |
| K10193 | 2 | oligogalacturonide transport system permease protein | Environmental Information Processing | |
| K10200 | 3 | N-acetylglucosamine transport system substrate-binding protein | Environmental Information Processing | |
| K10201 | 3 | N-acetylglucosamine transport system permease protein | Environmental Information Processing | |
| K10227 | 10 | sorbitol/mannitol transport system substrate-binding protein | Environmental Information Processing | |
| K10228 | 1 | sorbitol/mannitol transport system permease protein | Environmental Information Processing | |
| K10229 | 2 | sorbitol/mannitol transport system permease protein | Environmental Information Processing | |
| K10232 | 15 | alpha-glucoside transport system substrate-binding protein | Environmental Information Processing | |
| K10233 | 5 | alpha-glucoside transport system permease protein | Environmental Information Processing | |
| K10234 | 8 | alpha-glucoside transport system permease protein | Environmental Information Processing | |
| K10236 | 6 | trehalose/maltose transport system substrate-binding protein | Environmental Information Processing | |
| K10237 | 4 | trehalose/maltose transport system permease protein | Environmental Information Processing | |
| K10238 | 3 | trehalose/maltose transport system permease protein | Environmental Information Processing | |
| K10240 | 2 | cellobiose transport system substrate-binding protein | Environmental Information Processing | |
| K10241 | 1 | cellobiose transport system permease protein | Environmental Information Processing | |
| K10242 | 1 | cellobiose transport system permease protein | Environmental Information Processing | |

| | | | | |
|---|---|---|---|---|
| K10255 | 15 | omega-6 fatty acid desaturase (delta-12 desaturase) [EC:1.14.19.-] | Environmental Information Processing | |
| K10439 | 21 | ribose transport system substrate-binding protein | Environmental Information Processing | |
| K10440 | 29 | ribose transport system permease protein | Environmental Information Processing | |
| K10441 | 27 | ribose transport system ATP-binding protein [EC:3.6.3.17] | Environmental Information Processing | |
| K10538 | 1 | L-arabinose transport system permease protein | Environmental Information Processing | |
| K10541 | 3 | methyl-galactoside transport system permease protein | Environmental Information Processing | |
| K10542 | 1 | methyl-galactoside transport system ATP-binding protein | Environmental Information Processing | |
| K10543 | 5 | D-xylose transport system substrate-binding protein | Environmental Information Processing | |
| K10544 | 14 | D-xylose transport system permease protein | Environmental Information Processing | |
| K10545 | 1 | D-xylose transport system ATP-binding protein [EC:3.6.3.17] | Environmental Information Processing | |
| K10547 | 1 | putative multiple sugar transport system permease protein | Environmental Information Processing | |
| K10549 | 2 | D-allose transport system substrate-binding protein | Environmental Information Processing | |
| K10552 | 6 | fructose transport system substrate-binding protein | Environmental Information Processing | |
| K10553 | 2 | fructose transport system permease protein | Environmental Information Processing | |
| K10554 | 2 | fructose transport system ATP-binding protein | Environmental Information Processing | |
| K10555 | 9 | AI-2 transport system substrate-binding protein | Environmental Information Processing | |
| K10556 | 4 | AI-2 transport system permease protein | Environmental Information Processing | |
| K10557 | 2 | AI-2 transport system permease protein | Environmental Information Processing | |
| K10559 | 7 | rhamnose transport system substrate-binding protein | Environmental Information Processing | |
| K10560 | 3 | rhamnose transport system permease protein | Environmental Information Processing | |
| K10561 | 1 | rhamnose transport system permease protein | Environmental Information Processing | |
| K10697 | 6 | two-component system, OmpR family, response regulator RpaA | Environmental Information Processing | |
| K10715 | 7 | two-component system, sensor histidine kinase RpfC [EC:2.7.13.3] | Environmental Information Processing | |
| K10912 | 2 | two-component system, repressor protein LuxO | Environmental Information Processing | |
| K10914 | 3 | CRP/FNR family transcriptional regulator, cyclic AMP receptor | Environmental Information Processing | |
| K10941 | 18 | sigma-54 specific transcriptional regulator, flagellar regulatory | Environmental Information Processing | |
| K10942 | 29 | two-component system, sensor histidine kinase FlrB [EC:2.7.13.3] | Environmental Information Processing | |
| K10943 | 57 | two component system, response regulator FlrC | Environmental Information Processing | |
| K11003 | 4 | hemolysin D | Environmental Information Processing | |
| K11004 | 5 | ATP-binding cassette, subfamily B, bacterial HlyB/CyaB | Environmental Information Processing | |
| K11016 | 4 | hemolysin | Environmental Information Processing | |
| K11017 | 8 | hemolysin activation/secretion protein?? | Environmental Information Processing | |
| K11069 | 17 | spermidine/putrescine transport system substrate-binding protein | Environmental Information Processing | |
| K11070 | 19 | spermidine/putrescine transport system permease protein | Environmental Information Processing | |
| K11071 | 27 | spermidine/putrescine transport system permease protein | Environmental Information Processing | |
| K11072 | 19 | spermidine/putrescine transport system ATP-binding protein | Environmental Information Processing | |
| K11073 | 4 | putrescine transport system substrate-binding protein | Environmental Information Processing | |
| K11074 | 10 | putrescine transport system permease protein | Environmental Information Processing | |
| K11075 | 10 | putrescine transport system permease protein | Environmental Information Processing | |
| K11076 | 10 | putrescine transport system ATP-binding protein | Environmental Information Processing | |
| K11079 | 3 | mannopine transport system permease protein | Environmental Information Processing | |
| K11082 | 1 | 2-aminoethylphosphonate transport system permease protein | Environmental Information Processing | |
| K11085 | 118 | ATP-binding cassette, subfamily B, bacterial MsbA [EC:3.6.3.-] | Environmental Information Processing | |
| K11103 | 9 | aerobic C4-dicarboxylate transport protein | Environmental Information Processing | |
| K11183 | 16 | phosphocarrier protein FPr | Environmental Information Processing | |
| K11192 | 1 | PTS system, N-acetylmuramic acid-specific IIC component | Environmental Information Processing | |
| K11326 | 7 | cation efflux system protein involved in nickel and cobalt tolerance | Environmental Information Processing | |
| K11329 | 27 | two-component system, OmpR family, response regulator RpaB | Environmental Information Processing | |
| K11354 | 3 | two-component system, chemotaxis family, sensor kinase Cph1 | Environmental Information Processing | |
| K11355 | 3 | two-component system, chemotaxis family, response regulator Rcp1 | Environmental Information Processing | |
| K11356 | 2 | two-component system, sensor histidine kinase and response regulator | Environmental Information Processing | |
| K11357 | 7 | two-component system, cell cycle sensor histidine kinase DivJ | Environmental Information Processing | |
| K11383 | 3 | two-component system, NtrC family, sensor histidine kinase KinB | Environmental Information Processing | |
| K11384 | 7 | two-component system, NtrC family, response regulator AlgB | Environmental Information Processing | |
| K11443 | 7 | two-component system, cell cycle response regulator DivK | Environmental Information Processing | |
| K11444 | 8 | two-component system, chemotaxis family, response regulator WspR | Environmental Information Processing | |
| K11523 | 4 | two-component system, chemotaxis family, response regulator PixH | Environmental Information Processing | |
| K11525 | 1 | methyl-accepting chemotaxis protein PixJ | Environmental Information Processing | |
| K11526 | 1 | two-component system, chemotaxis family, sensor histidine kinase and | Environmental Information Processing | |
| K11603 | 1 | manganese transport system ATP-binding protein | Environmental Information Processing | |
| K11604 | 1 | manganese/iron transport system substrate-binding protein | Environmental Information Processing | |
| K11606 | 3 | manganese/iron transport system permease protein | Environmental Information Processing | |
| K11615 | 3 | two-component system, CitB family, response regulator MalR | Environmental Information Processing | |
| K11616 | 1 | malate:Na+ symporter | Environmental Information Processing | |
| K11617 | 3 | two-component system, NarL family, sensor histidine kinase LiaS | Environmental Information Processing | |
| K11618 | 39 | two-component system, NarL family, response regulator LiaR | Environmental Information Processing | |
| K11619 | 11 | lia operon protein LiaI | Environmental Information Processing | |
| K11621 | 1 | lia operon protein LiaG | Environmental Information Processing | |
| K11624 | 2 | two-component system, NarL family, response regulator YdfI | Environmental Information Processing | |

| | | | | |
|---|---|---|---|---|
| K11625 | 9 | membrane protein YdfJ | Environmental Information Processing | |
| K11634 | 1 | two-component system, OmpR family, response regulator YxdJ | Environmental Information Processing | |
| K11688 | 92 | C4-dicarboxylate-binding protein DctP | Environmental Information Processing | |
| K11689 | 12 | C4-dicarboxylate transporter, DctQ subunit | Environmental Information Processing | |
| K11690 | 144 | C4-dicarboxylate transporter, DctM subunit | Environmental Information Processing | |
| K11710 | 3 | manganese/zinc/iron transport system ATP- binding protein | Environmental Information Processing | |
| K11711 | 16 | two-component system, LuxR family, sensor histidine kinase DctS | Environmental Information Processing | |
| K11712 | 12 | two-component system, LuxR family, response regulator DctR | Environmental Information Processing | |
| K11720 | 14 | lipopolysaccharide export system permease protein | Environmental Information Processing | |
| K11891 | 8 | type VI secretion system protein ImpL | Environmental Information Processing | |
| K11892 | 8 | type VI secretion system protein ImpK | Environmental Information Processing | |
| K11903 | 1 | type VI secretion system secreted protein Hcp | Environmental Information Processing | |
| K11904 | 9 | type VI secretion system secreted protein VgrG | Environmental Information Processing | |
| K11906 | 7 | type VI secretion system protein VasD | Environmental Information Processing | |
| K11907 | 13 | type VI secretion system protein VasG | Environmental Information Processing | |
| K11912 | 28 | serine/threonine-protein kinase PpkA [EC:2.7.11.1] | Environmental Information Processing | |
| K11913 | 3 | type VI secretion system protein | Environmental Information Processing | |
| K11953 | 1 | bicarbonate transport system ATP-binding protein [EC:3.6.3.-] | Environmental Information Processing | |
| K11954 | 2 | neutral amino acid transport system substrate-binding protein | Environmental Information Processing | |
| K11955 | 1 | neutral amino acid transport system permease protein | Environmental Information Processing | |
| K11956 | 1 | neutral amino acid transport system permease protein | Environmental Information Processing | |
| K11957 | 2 | neutral amino acid transport system ATP-binding protein | Environmental Information Processing | |
| K11958 | 2 | neutral amino acid transport system ATP-binding protein | Environmental Information Processing | |
| K11959 | 4 | urea transport system substrate-binding protein | Environmental Information Processing | |
| K11960 | 1 | urea transport system permease protein | Environmental Information Processing | |
| K11961 | 2 | urea transport system permease protein | Environmental Information Processing | |
| K11963 | 1 | urea transport system ATP-binding protein | Environmental Information Processing | |
| K11987 | 1 | prostaglandin-endoperoxide synthase 2 [EC:1.14.99.1] | Environmental Information Processing | |
| K12257 | 30 | SecD/SecF fusion protein | Environmental Information Processing | |
| K12340 | 36 | outer membrane channel protein TolC | Environmental Information Processing | |
| K12368 | 17 | dipeptide transport system substrate-binding protein | Environmental Information Processing | |
| K12369 | 12 | dipeptide transport system permease protein | Environmental Information Processing | |
| K12370 | 10 | dipeptide transport system permease protein | Environmental Information Processing | |
| K12371 | 6 | dipeptide transport system ATP-binding protein | Environmental Information Processing | |
| K12372 | 1 | dipeptide transport system ATP-binding protein | Environmental Information Processing | |
| K12536 | 4 | ATP-binding cassette, subfamily C, bacterial HasD | Environmental Information Processing | |
| K12541 | 3 | ATP-binding cassette, subfamily C, bacterial LapB | Environmental Information Processing | |
| K13041 | 1 | two-component system, LuxR family, response regulator TtrR | Environmental Information Processing | |
| K13409 | 1 | ATP-binding cassette, subfamily B, bacterial RaxB | Environmental Information Processing | |
| K13486 | 2 | chemotaxis protein methyltransferase WspC | Environmental Information Processing | |
| K13490 | 5 | two-component system, chemotaxis family, sensor histidine kinase and | Environmental Information Processing | |
| K13491 | 1 | two-component system, chemotaxis family, response regulator WspF | Environmental Information Processing | |
| K13532 | 2 | two-component system, sporulation sensor kinase D [EC:2.7.13.3] | Environmental Information Processing | |
| K13533 | 7 | two-component system, sporulation sensor kinase E [EC:2.7.13.3] | Environmental Information Processing | |
| K13584 | 1 | two-component system, cell cycle response regulator CtrA | Environmental Information Processing | |
| K13587 | 41 | two-component system, cell cycle sensor histidine kinase and | Environmental Information Processing | |
| K13588 | 1 | histidine phosphotransferase ChpT | Environmental Information Processing | |
| K13589 | 2 | two-component system, cell cycle response regulator CpdR | Environmental Information Processing | |
| K13598 | 35 | two-component system, NtrC family, nitrogen regulation sensor | Environmental Information Processing | |
| K13599 | 57 | two-component system, NtrC family, nitrogen regulation response | Environmental Information Processing | |
| K13815 | 21 | two-component system, response regulator RpfG | Environmental Information Processing | |
| K13816 | 7 | DSF synthase | Environmental Information Processing | |
| K13889 | 13 | glutathione transport system substrate-binding protein | Environmental Information Processing | |
| K13890 | 6 | glutathione transport system permease protein | Environmental Information Processing | |
| K13891 | 2 | glutathione transport system permease protein | Environmental Information Processing | |
| K13892 | 4 | glutathione transport system ATP-binding protein | Environmental Information Processing | |
| K13893 | 9 | microcin C transport system substrate-binding protein | Environmental Information Processing | |
| K13894 | 2 | microcin C transport system permease protein | Environmental Information Processing | |
| K13895 | 4 | microcin C transport system permease protein | Environmental Information Processing | |
| K13896 | 3 | microcin C transport system ATP-binding protein | Environmental Information Processing | |
| K13924 | 124 | two-component system, chemotaxis family, CheB/CheR fusion protein | Environmental Information Processing | |

| | | | | |
|---|---|---|---|---|
| | | | | |
| | | | | |

| KEGG ID | Sequences | KEGG pathway | KEGG category |
|---|---|---|---|
| K00566 | 48 | tRNA (5-methylaminomethyl-2-thiouridylate)-methyltransferase | Genetic Information Processing |
| K00604 | 50 | methionyl-tRNA formyltransferase [EC:2.1.2.9] | Genetic Information Processing |
| K00784 | 75 | ribonuclease Z [EC:3.1.26.11] | Genetic Information Processing |
| K00850 | 56 | 6-phosphofructokinase [EC:2.7.1.11] | Genetic Information Processing |
| K00937 | 60 | polyphosphate kinase [EC:2.7.4.1] | Genetic Information Processing |
| K00962 | 39 | polyribonucleotide nucleotidyltransferase [EC:2.7.7.8] | Genetic Information Processing |
| K00970 | 57 | poly(A) polymerase [EC:2.7.7.19] | Genetic Information Processing |
| K00974 | 27 | tRNA nucleotidyltransferase (CCA-adding enzyme) [EC:2.7.7.25 | Genetic Information Processing |
| K01011 | 21 | 3-mercaptopyruvate sulfurtransferase [EC:2.8.1.2] | Genetic Information Processing |
| K01042 | 37 | L-seryl-tRNA(Ser) seleniumtransferase [EC:2.9.1.1] | Genetic Information Processing |
| K01142 | 29 | exodeoxyribonuclease III [EC:3.1.11.2] | Genetic Information Processing |
| K01151 | 37 | deoxyribonuclease IV [EC:3.1.21.2] | Genetic Information Processing |
| K01159 | 34 | crossover junction endodeoxyribonuclease RuvC [EC:3.1.22.4] | Genetic Information Processing |
| K01246 | 12 | DNA-3-methyladenine glycosylase I [EC:3.2.2.20] | Genetic Information Processing |
| K01247 | 18 | DNA-3-methyladenine glycosylase II [EC:3.2.2.21] | Genetic Information Processing |
| K01689 | 42 | enolase [EC:4.2.1.11] | Genetic Information Processing |
| K01866 | 51 | tyrosyl-tRNA synthetase [EC:6.1.1.1] | Genetic Information Processing |
| K01867 | 46 | tryptophanyl-tRNA synthetase [EC:6.1.1.2] | Genetic Information Processing |
| K01868 | 67 | threonyl-tRNA synthetase [EC:6.1.1.3] | Genetic Information Processing |
| K01869 | 73 | leucyl-tRNA synthetase [EC:6.1.1.4] | Genetic Information Processing |
| K01870 | 85 | isoleucyl-tRNA synthetase [EC:6.1.1.5] | Genetic Information Processing |
| K01872 | 71 | alanyl-tRNA synthetase [EC:6.1.1.7] | Genetic Information Processing |
| K01873 | 99 | valyl-tRNA synthetase [EC:6.1.1.9] | Genetic Information Processing |
| K01874 | 56 | methionyl-tRNA synthetase [EC:6.1.1.10] | Genetic Information Processing |
| K01875 | 59 | seryl-tRNA synthetase [EC:6.1.1.11] | Genetic Information Processing |
| K01876 | 57 | aspartyl-tRNA synthetase [EC:6.1.1.12] | Genetic Information Processing |
| K01878 | 12 | glycyl-tRNA synthetase alpha chain [EC:6.1.1.14] | Genetic Information Processing |
| K01879 | 36 | glycyl-tRNA synthetase beta chain [EC:6.1.1.14] | Genetic Information Processing |
| K01880 | 17 | glycyl-tRNA synthetase [EC:6.1.1.14] | Genetic Information Processing |
| K01881 | 50 | prolyl-tRNA synthetase [EC:6.1.1.15] | Genetic Information Processing |
| K01883 | 69 | cysteinyl-tRNA synthetase [EC:6.1.1.16] | Genetic Information Processing |
| K01885 | 74 | glutamyl-tRNA synthetase [EC:6.1.1.17] | Genetic Information Processing |
| K01886 | 28 | glutaminyl-tRNA synthetase [EC:6.1.1.18] | Genetic Information Processing |
| K01887 | 498 | arginyl-tRNA synthetase [EC:6.1.1.19] | Genetic Information Processing |
| K01889 | 40 | phenylalanyl-tRNA synthetase alpha chain [EC:6.1.1.20] | Genetic Information Processing |
| K01890 | 515 | phenylalanyl-tRNA synthetase beta chain [EC:6.1.1.20] | Genetic Information Processing |
| K01892 | 46 | histidyl-tRNA synthetase [EC:6.1.1.21] | Genetic Information Processing |
| K01893 | 25 | asparaginyl-tRNA synthetase [EC:6.1.1.22] | Genetic Information Processing |
| K01971 | 172 | DNA ligase (ATP) [EC:6.5.1.1] | Genetic Information Processing |
| K01972 | 63 | DNA ligase (NAD+) [EC:6.5.1.2] | Genetic Information Processing |
| K02314 | 78 | replicative DNA helicase [EC:3.6.1.-] | Genetic Information Processing |
| K02316 | 65 | DNA primase [EC:2.7.7.-] | Genetic Information Processing |
| K02319 | 11 | DNA polymerase I [EC:2.7.7.7] | Genetic Information Processing |
| K02330 | 6 | DNA polymerase beta subunit [EC:2.7.7.7 4.2.99.-] | Genetic Information Processing |
| K02335 | 90 | DNA polymerase I [EC:2.7.7.7] | Genetic Information Processing |
| K02337 | 211 | DNA polymerase III subunit alpha [EC:2.7.7.7] | Genetic Information Processing |
| K02338 | 59 | DNA polymerase III subunit beta [EC:2.7.7.7] | Genetic Information Processing |
| K02339 | 6 | DNA polymerase III subunit chi [EC:2.7.7.7] | Genetic Information Processing |
| K02340 | 11 | DNA polymerase III subunit delta [EC:2.7.7.7] | Genetic Information Processing |
| K02341 | 35 | DNA polymerase III subunit delta' [EC:2.7.7.7] | Genetic Information Processing |
| K02342 | 60 | DNA polymerase III subunit epsilon [EC:2.7.7.7] | Genetic Information Processing |
| K02343 | 65 | DNA polymerase III subunit gamma/tau [EC:2.7.7.7] | Genetic Information Processing |
| K02345 | 1 | DNA polymerase III subunit theta [EC:2.7.7.7] | Genetic Information Processing |
| K02433 | 141 | aspartyl-tRNA(Asn)/glutamyl-tRNA (Gln) amidotransferase subunit A | Genetic Information Processing |
| K02434 | 54 | aspartyl-tRNA(Asn)/glutamyl-tRNA (Gln) amidotransferase subunit B | Genetic Information Processing |
| K02435 | 18 | aspartyl-tRNA(Asn)/glutamyl-tRNA (Gln) amidotransferase subunit C | Genetic Information Processing |
| K02686 | 4 | primosomal replication protein N | Genetic Information Processing |
| K02863 | 21 | large subunit ribosomal protein L1 | Genetic Information Processing |
| K02864 | 19 | large subunit ribosomal protein L10 | Genetic Information Processing |
| K02867 | 17 | large subunit ribosomal protein L11 | Genetic Information Processing |
| K02871 | 16 | large subunit ribosomal protein L13 | Genetic Information Processing |

| | | | |
|---|---|---|---|
| K02874 | 22 | large subunit ribosomal protein L14 | Genetic Information Processing |
| K02876 | 12 | large subunit ribosomal protein L15 | Genetic Information Processing |
| K02878 | 19 | large subunit ribosomal protein L16 | Genetic Information Processing |
| K02879 | 21 | large subunit ribosomal protein L17 | Genetic Information Processing |
| K02881 | 11 | large subunit ribosomal protein L18 | Genetic Information Processing |
| K02884 | 16 | large subunit ribosomal protein L19 | Genetic Information Processing |
| K02886 | 47 | large subunit ribosomal protein L2 | Genetic Information Processing |
| K02887 | 6 | large subunit ribosomal protein L20 | Genetic Information Processing |
| K02888 | 23 | large subunit ribosomal protein L21 | Genetic Information Processing |
| K02890 | 18 | large subunit ribosomal protein L22 | Genetic Information Processing |
| K02892 | 34 | large subunit ribosomal protein L23 | Genetic Information Processing |
| K02895 | 14 | large subunit ribosomal protein L24 | Genetic Information Processing |
| K02897 | 28 | large subunit ribosomal protein L25 | Genetic Information Processing |
| K02899 | 20 | large subunit ribosomal protein L27 | Genetic Information Processing |
| K02902 | 9 | large subunit ribosomal protein L28 | Genetic Information Processing |
| K02904 | 6 | large subunit ribosomal protein L29 | Genetic Information Processing |
| K02906 | 36 | large subunit ribosomal protein L3 | Genetic Information Processing |
| K02907 | 9 | large subunit ribosomal protein L30 | Genetic Information Processing |
| K02909 | 22 | large subunit ribosomal protein L31 | Genetic Information Processing |
| K02911 | 73 | large subunit ribosomal protein L32 | Genetic Information Processing |
| K02913 | 9 | large subunit ribosomal protein L33 | Genetic Information Processing |
| K02914 | 15 | large subunit ribosomal protein L34 | Genetic Information Processing |
| K02916 | 7 | large subunit ribosomal protein L35 | Genetic Information Processing |
| K02919 | 2 | large subunit ribosomal protein L36 | Genetic Information Processing |
| K02926 | 30 | large subunit ribosomal protein L4 | Genetic Information Processing |
| K02931 | 15 | large subunit ribosomal protein L5 | Genetic Information Processing |
| K02933 | 28 | large subunit ribosomal protein L6 | Genetic Information Processing |
| K02935 | 6 | large subunit ribosomal protein L7/L12 | Genetic Information Processing |
| K02939 | 23 | large subunit ribosomal protein L9 | Genetic Information Processing |
| K02945 | 47 | small subunit ribosomal protein S1 | Genetic Information Processing |
| K02946 | 441 | small subunit ribosomal protein S10 | Genetic Information Processing |
| K02948 | 11 | small subunit ribosomal protein S11 | Genetic Information Processing |
| K02950 | 7 | small subunit ribosomal protein S12 | Genetic Information Processing |
| K02952 | 15 | small subunit ribosomal protein S13 | Genetic Information Processing |
| K02954 | 7 | small subunit ribosomal protein S14 | Genetic Information Processing |
| K02956 | 8 | small subunit ribosomal protein S15 | Genetic Information Processing |
| K02959 | 23 | small subunit ribosomal protein S16 | Genetic Information Processing |
| K02961 | 10 | small subunit ribosomal protein S17 | Genetic Information Processing |
| K02963 | 15 | small subunit ribosomal protein S18 | Genetic Information Processing |
| K02965 | 4 | small subunit ribosomal protein S19 | Genetic Information Processing |
| K02967 | 68 | small subunit ribosomal protein S2 | Genetic Information Processing |
| K02968 | 28 | small subunit ribosomal protein S20 | Genetic Information Processing |
| K02970 | 4 | small subunit ribosomal protein S21 | Genetic Information Processing |
| K02982 | 20 | small subunit ribosomal protein S3 | Genetic Information Processing |
| K02986 | 30 | small subunit ribosomal protein S4 | Genetic Information Processing |
| K02988 | 13 | small subunit ribosomal protein S5 | Genetic Information Processing |
| K02990 | 23 | small subunit ribosomal protein S6 | Genetic Information Processing |
| K02992 | 15 | small subunit ribosomal protein S7 | Genetic Information Processing |
| K02994 | 23 | small subunit ribosomal protein S8 | Genetic Information Processing |
| K02996 | 16 | small subunit ribosomal protein S9 | Genetic Information Processing |
| K03040 | 34 | DNA-directed RNA polymerase subunit alpha [EC:2.7.7.6] | Genetic Information Processing |
| K03043 | 77 | DNA-directed RNA polymerase subunit beta [EC:2.7.7.6] | Genetic Information Processing |
| K03046 | 516 | DNA-directed RNA polymerase subunit beta' [EC:2.7.7.6] | Genetic Information Processing |
| K03060 | 10 | DNA-directed RNA polymerase subunit omega [EC:2.7.7.6] | Genetic Information Processing |
| K03070 | 74 | preprotein translocase subunit SecA | Genetic Information Processing |
| K03071 | 7 | preprotein translocase subunit SecB | Genetic Information Processing |
| K03072 | 476 | preprotein translocase subunit SecD | Genetic Information Processing |
| K03073 | 2 | preprotein translocase subunit SecE | Genetic Information Processing |
| K03074 | 41 | preprotein translocase subunit SecF | Genetic Information Processing |
| K03075 | 3 | preprotein translocase subunit SecG | Genetic Information Processing |
| K03076 | 36 | preprotein translocase subunit SecY | Genetic Information Processing |
| K03100 | 92 | signal peptidase I [EC:3.4.21.89] | Genetic Information Processing |

| | | | |
|---|---|---|---|
| K03101 | 20 | signal peptidase II [EC:3.4.23.36] | Genetic Information Processing |
| K03106 | 47 | signal recognition particle subunit SRP54 | Genetic Information Processing |
| K03110 | 23 | fused signal recognition particle receptor | Genetic Information Processing |
| K03111 | 34 | single-strand DNA-binding protein | Genetic Information Processing |
| K03113 | 2 | translation initiation factor SUI1 | Genetic Information Processing |
| K03116 | 10 | sec-independent protein translocase protein TatA | Genetic Information Processing |
| K03117 | 4 | sec-independent protein translocase protein TatB | Genetic Information Processing |
| K03118 | 57 | sec-independent protein translocase protein TatC | Genetic Information Processing |
| K03148 | 16 | adenylyltransferase ThiF [EC:2.7.7.-] | Genetic Information Processing |
| K03151 | 4 | thiamine biosynthesis protein ThiI | Genetic Information Processing |
| K03154 | 5 | thiamine biosynthesis ThiS | Genetic Information Processing |
| K03210 | 16 | preprotein translocase subunit YajC | Genetic Information Processing |
| K03217 | 71 | preprotein translocase subunit YidC | Genetic Information Processing |
| K03265 | 1 | peptide chain release factor eRF subunit 1 | Genetic Information Processing |
| K03432 | 2 | proteasome alpha subunit [EC:3.4.25.1] | Genetic Information Processing |
| K03433 | 9 | proteasome beta subunit [EC:3.4.25.1] | Genetic Information Processing |
| K03469 | 41 | ribonuclease HI [EC:3.1.26.4] | Genetic Information Processing |
| K03470 | 44 | ribonuclease HII [EC:3.1.26.4] | Genetic Information Processing |
| K03471 | 3 | ribonuclease HIII [EC:3.1.26.4] | Genetic Information Processing |
| K03540 | 15 | ribonuclease P subunit RPR2 [EC:3.1.26.5] | Genetic Information Processing |
| K03550 | 21 | holliday junction DNA helicase RuvA | Genetic Information Processing |
| K03551 | 35 | holliday junction DNA helicase RuvB | Genetic Information Processing |
| K03553 | 41 | recombination protein RecA | Genetic Information Processing |
| K03555 | 79 | DNA mismatch repair protein MutS | Genetic Information Processing |
| K03572 | 44 | DNA mismatch repair protein MutL | Genetic Information Processing |
| K03575 | 41 | A/G-specific adenine glycosylase [EC:3.2.2.-] | Genetic Information Processing |
| K03581 | 29 | exodeoxyribonuclease V alpha subunit [EC:3.1.11.5] | Genetic Information Processing |
| K03582 | 8 | exodeoxyribonuclease V beta subunit [EC:3.1.11.5] | Genetic Information Processing |
| K03583 | 15 | exodeoxyribonuclease V gamma subunit [EC:3.1.11.5] | Genetic Information Processing |
| K03584 | 34 | DNA repair protein RecO (recombination protein O) | Genetic Information Processing |
| K03601 | 45 | exodeoxyribonuclease VII large subunit [EC:3.1.11.6] | Genetic Information Processing |
| K03602 | 5 | exodeoxyribonuclease VII small subunit [EC:3.1.11.6] | Genetic Information Processing |
| K03628 | 36 | transcription termination factor Rho | Genetic Information Processing |
| K03629 | 18 | DNA replication and repair protein RecF | Genetic Information Processing |
| K03635 | 22 | molybdenum cofactor biosynthesis protein E | Genetic Information Processing |
| K03636 | 10 | molybdenum cofactor biosynthesis protein D | Genetic Information Processing |
| K03637 | 16 | molybdenum cofactor biosynthesis protein C | Genetic Information Processing |
| K03639 | 47 | molybdenum cofactor biosynthesis protein A | Genetic Information Processing |
| K03648 | 29 | uracil-DNA glycosylase [EC:3.2.2.-] | Genetic Information Processing |
| K03649 | 18 | TDG/mug DNA glycosylase family protein [EC:3.2.2.-] | Genetic Information Processing |
| K03652 | 17 | DNA-3-methyladenine glycosylase [EC:3.2.2.21] | Genetic Information Processing |
| K03654 | 47 | ATP-dependent DNA helicase RecQ [EC:3.6.1.-] | Genetic Information Processing |
| K03655 | 58 | ATP-dependent DNA helicase RecG [EC:3.6.1.-] | Genetic Information Processing |
| K03657 | 133 | DNA helicase II / ATP-dependent DNA helicase PcrA [EC:3.6.1.-] | Genetic Information Processing |
| K03666 | 10 | host factor-I protein | Genetic Information Processing |
| K03685 | 34 | ribonuclease III [EC:3.1.26.3] | Genetic Information Processing |
| K03701 | 117 | excinuclease ABC subunit A | Genetic Information Processing |
| K03702 | 42 | excinuclease ABC subunit B | Genetic Information Processing |
| K03703 | 61 | excinuclease ABC subunit C | Genetic Information Processing |
| K03723 | 74 | transcription-repair coupling factor (superfamily II helicase) | Genetic Information Processing |
| K03732 | 1 | ATP-dependent RNA helicase RhlB [EC:3.6.4.13] | Genetic Information Processing |
| K03763 | 10 | DNA polymerase III subunit alpha, Gram-positive type [EC:2.7.7.7] | Genetic Information Processing |
| K03831 | 8 | molybdopterin biosynthesis protein Mog | Genetic Information Processing |
| K04043 | 64 | molecular chaperone DnaK | Genetic Information Processing |
| K04066 | 67 | primosomal protein N' (replication factor Y) (superfamily II | Genetic Information Processing |
| K04067 | 9 | primosomal replication protein N'' | Genetic Information Processing |
| K04077 | 43 | chaperonin GroEL | Genetic Information Processing |
| K04079 | 17 | molecular chaperone HtpG | Genetic Information Processing |
| K04085 | 8 | tRNA 2-thiouridine synthesizing protein A [EC:2.8.1.-] | Genetic Information Processing |
| K04487 | 57 | cysteine desulfurase [EC:2.8.1.7] | Genetic Information Processing |
| K04566 | 6 | lysyl-tRNA synthetase, class I [EC:6.1.1.6] | Genetic Information Processing |
| K04567 | 51 | lysyl-tRNA synthetase, class II [EC:6.1.1.6] | Genetic Information Processing |

| | | | |
|---|---|---|---|
| K05522 | 12 | endonuclease VIII [EC:3.2.2.- 4.2.99.18] | Genetic Information Processing |
| K05592 | 36 | ATP-dependent RNA helicase DeaD [EC:3.6.4.13] | Genetic Information Processing |
| K06187 | 21 | recombination protein RecR | Genetic Information Processing |
| K06223 | 4 | DNA adenine methylase [EC:2.1.1.72] | Genetic Information Processing |
| K07178 | 2 | RIO kinase 1 [EC:2.7.11.1] | Genetic Information Processing |
| K07236 | 1 | tRNA 2-thiouridine synthesizing protein C | Genetic Information Processing |
| K07237 | 3 | tRNA 2-thiouridine synthesizing protein B | Genetic Information Processing |
| K07456 | 34 | DNA mismatch repair protein MutS2 | Genetic Information Processing |
| K07462 | 59 | single-stranded-DNA-specific exonuclease [EC:3.1.-.-] | Genetic Information Processing |
| K08300 | 19 | ribonuclease E [EC:3.1.26.12] | Genetic Information Processing |
| K08311 | 4 | putative (di)nucleoside polyphosphate hydrolase [EC:3.6.1.-] | Genetic Information Processing |
| K09698 | 20 | nondiscriminating glutamyl-tRNA synthetase [EC:6.1.1.24] | Genetic Information Processing |
| K09759 | 2 | nondiscriminating aspartyl-tRNA synthetase [EC:6.1.1.23] | Genetic Information Processing |
| K10563 | 62 | formamidopyrimidine-DNA glycosylase [EC:3.2.2.23 4.2.99.18] | Genetic Information Processing |
| K10747 | 4 | DNA ligase 1 [EC:6.5.1.1] | Genetic Information Processing |
| K10754 | 4 | replication factor C subunit 1 | Genetic Information Processing |
| K10773 | 24 | endonuclease III [EC:4.2.99.18] | Genetic Information Processing |
| K10843 | 12 | DNA excision repair protein ERCC-3 [EC:3.6.1.-] | Genetic Information Processing |
| K10844 | 3 | DNA excision repair protein ERCC-2 [EC:3.6.1.-] | Genetic Information Processing |
| K10857 | 1 | exodeoxyribonuclease X [EC:3.1.11.-] | Genetic Information Processing |
| K10979 | 36 | DNA end-binding protein Ku | Genetic Information Processing |
| K11179 | 14 | tRNA 2-thiouridine synthesizing protein E [EC:2.8.1.-] | Genetic Information Processing |
| K11927 | 27 | ATP-dependent RNA helicase RhlE [EC:3.6.4.13] | Genetic Information Processing |
| K12257 | 30 | SecD/SecF fusion protein | Genetic Information Processing |
| K12573 | 53 | ribonuclease R [EC:3.1.-.-] | Genetic Information Processing |
| K12574 | 39 | ribonuclease J [EC:3.1.-.-] | Genetic Information Processing |
| K13280 | 2 | signal peptidase, endoplasmic reticulum-type [EC:3.4.-.-] | Genetic Information Processing |
| K13288 | 10 | oligoribonuclease [EC:3.1.-.-] | Genetic Information Processing |
| K13525 | 23 | transitional endoplasmic reticulum ATPase | Genetic Information Processing |
| K13527 | 10 | proteasome-associated ATPase | Genetic Information Processing |
| K13529 | 16 | AraC family transcriptional regulator, regulatory protein of | Genetic Information Processing |
| K13797 | 2 | DNA-directed RNA polymerase subunit beta-beta' [EC:2.7.7.6] | Genetic Information Processing |
| K13993 | 58 | HSP20 family protein | Genetic Information Processing |

| KEGG I | Sequences | KEGG pathway | KEGG category | |
|---|---|---|---|---|
| K00036 | 118 | glucose-6-phosphate 1-dehydrogenase [EC:1.1.1.49] | Human diseases | |
| K00079 | 12 | carbonyl reductase (NADPH) [EC:1.1.1.184] | Human diseases | |
| K00121 | 49 | S-(hydroxymethyl)glutathione dehydrogenase / alcohol dehydrogenase | Human diseases | |
| K00129 | 2 | aldehyde dehydrogenase (NAD(P)+) [EC:1.2.1.5] | Human diseases | |
| K00134 | 29 | glyceraldehyde 3-phosphate dehydrogenase [EC:1.2.1.12] | Human diseases | |
| K00161 | 65 | pyruvate dehydrogenase E1 component subunit alpha [EC:1.2.4.1] | Human diseases | |
| K00162 | 66 | pyruvate dehydrogenase E1 component subunit beta [EC:1.2.4.1] | Human diseases | |
| K00239 | 70 | succinate dehydrogenase flavoprotein subunit [EC:1.3.99.1] | Human diseases | |
| K00274 | 21 | monoamine oxidase [EC:1.4.3.4] | Human diseases | |
| K00411 | 10 | ubiquinol-cytochrome c reductase iron-sulfur subunit [EC:1.10.2.2] | Human diseases | |
| K00412 | 35 | ubiquinol-cytochrome c reductase cytochrome b subunit [EC:1.10.2.2] | Human diseases | |
| K00413 | 8 | ubiquinol-cytochrome c reductase cytochrome c1 subunit [EC:1.10.2.2] | Human diseases | |
| K00558 | 15 | DNA (cytosine-5-)-methyltransferase [EC:2.1.1.37] | Human diseases | |
| K00622 | 5 | arylamine N-acetyltransferase [EC:2.3.1.5] | Human diseases | |
| K00677 | 32 | UDP-N-acetylglucosamine acyltransferase [EC:2.3.1.129] | Human diseases | |
| K00688 | 90 | starch phosphorylase [EC:2.4.1.1] | Human diseases | |
| K00693 | 14 | glycogen(starch) synthase [EC:2.4.1.11] | Human diseases | |
| K00758 | 21 | thymidine phosphorylase [EC:2.4.2.4] | Human diseases | |
| K00799 | 108 | glutathione S-transferase [EC:2.5.1.18] | Human diseases | |
| K00820 | 67 | glucosamine--fructose-6-phosphate aminotransferase (isomerizing) | Human diseases | |
| K00850 | 56 | 6-phosphofructokinase [EC:2.7.1.11] | Human diseases | |
| K00873 | 44 | pyruvate kinase [EC:2.7.1.40] | Human diseases | |
| K00901 | 6 | diacylglycerol kinase [EC:2.7.1.107] | Human diseases | |
| K01000 | 52 | phospho-N-acetylmuramoyl-pentapeptide-transferase [EC:2.7.8.13] | Human diseases | |
| K01078 | 17 | acid phosphatase [EC:3.1.3.2] | Human diseases | |
| K01115 | 8 | phospholipase D [EC:3.1.4.4] | Human diseases | |
| K01207 | 53 | beta-N-acetylhexosaminidase [EC:3.2.1.52] | Human diseases | |
| K01253 | 35 | microsomal epoxide hydrolase [EC:3.3.2.9] | Human diseases | |
| K01283 | 4 | peptidyl-dipeptidase A [EC:3.4.15.1] | Human diseases | |
| K01312 | 9 | trypsin [EC:3.4.21.4] | Human diseases | |
| K01354 | 61 | oligopeptidase B [EC:3.4.21.83] | Human diseases | |
| K01389 | 2 | neprilysin [EC:3.4.24.11] | Human diseases | |
| K01392 | 9 | thimet oligopeptidase [EC:3.4.24.15] | Human diseases | |
| K01401 | 1 | aureolysin [EC:3.4.24.29] | Human diseases | |
| K01406 | 35 | serralysin [EC:3.4.24.40] | Human diseases | |
| K01425 | 10 | glutaminase [EC:3.5.1.2] | Human diseases | |
| K01428 | 11 | urease alpha subunit [EC:3.5.1.5] | Human diseases | |
| K01448 | 611 | N-acetylmuramoyl-L-alanine amidase [EC:3.5.1.28] | Human diseases | |
| K01467 | 153 | beta-lactamase [EC:3.5.2.6] | Human diseases | |
| K01476 | 13 | arginase [EC:3.5.3.1] | Human diseases | |
| K01488 | 35 | adenosine deaminase [EC:3.5.4.4] | Human diseases | |
| K01580 | 8 | glutamate decarboxylase [EC:4.1.1.15] | Human diseases | |
| K01593 | 61 | aromatic-L-amino-acid decarboxylase [EC:4.1.1.28] | Human diseases | |
| K01596 | 30 | phosphoenolpyruvate carboxykinase (GTP) [EC:4.1.1.32] | Human diseases | |
| K01679 | 37 | fumarate hydratase, class II [EC:4.2.1.2] | Human diseases | |
| K01775 | 45 | alanine racemase [EC:5.1.1.1] | Human diseases | |
| K01834 | 100 | phosphoglycerate mutase [EC:5.4.2.1] | Human diseases | |
| K01921 | 88 | D-alanine-D-alanine ligase [EC:6.3.2.4] | Human diseases | |
| K01929 | 58 | UDP-N-acetylmuramoylalanyl-D-glutamyl-2,6-diaminopimelate--D-alanyl- | Human diseases | |
| K02040 | 62 | phosphate transport system substrate-binding protein | Human diseases | |
| K02330 | 6 | DNA polymerase beta subunit [EC:2.7.7.7 4.2.99.-] | Human diseases | |
| K02405 | 21 | RNA polymerase sigma factor for flagellar operon FliA | Human diseases | |
| K02406 | 10 | flagellin | Human diseases | |
| K02545 | 2 | penicillin-binding protein 2 prime | Human diseases | |
| K02547 | 1 | methicillin resistance protein | Human diseases | |
| K02563 | 30 | UDP-N-acetylglucosamine--N-acetylmuramyl-(pentapeptide) | Human diseases | |

| | | | | |
|---|---|---|---|---|
| K03087 | 22 | RNA polymerase nonessential primary-like sigma factor | Human diseases | |
| K03092 | 47 | RNA polymerase sigma-54 factor | Human diseases | |
| K03196 | 1 | type IV secretion system protein VirB11 | Human diseases | |
| K03197 | 2 | type IV secretion system protein VirB2 | Human diseases | |
| K03367 | 32 | D-alanine-poly(phosphoribitol) ligase [EC:6.1.1.13] | Human diseases | |
| K03385 | 9 | formate-dependent nitrite reductase, periplasmic cytochrome c552 | Human diseases | |
| K03585 | 50 | membrane fusion protein | Human diseases | |
| K03587 | 74 | cell division protein FtsI (penicillin-binding protein 3) | Human diseases | |
| K03596 | 50 | GTP-binding protein LepA | Human diseases | |
| K03648 | 29 | uracil-DNA glycosylase [EC:3.2.2.-] | Human diseases | |
| K03673 | 13 | thiol:disulfide interchange protein DsbA | Human diseases | |
| K03685 | 34 | ribonuclease III [EC:3.1.26.3] | Human diseases | |
| K03693 | 1 | penicillin-binding protein | Human diseases | |
| K03740 | 1 | D-alanine transfer protein | Human diseases | |
| K03760 | 1 | phosphoethanolamine transferase | Human diseases | |
| K03767 | 15 | peptidyl-prolyl cis-trans isomerase A (cyclophilin A) [EC:5.2.1.8] | Human diseases | |
| K03781 | 12 | catalase [EC:1.11.1.6] | Human diseases | |
| K03943 | 6 | NADH dehydrogenase (ubiquinone) flavoprotein 2 [EC:1.6.5.3 1.6.99.3] | Human diseases | |
| K04043 | 64 | molecular chaperone DnaK | Human diseases | |
| K04077 | 43 | chaperonin GroEL | Human diseases | |
| K04079 | 17 | molecular chaperone HtpG | Human diseases | |
| K04097 | 7 | glutathione S-transferase [EC:2.5.1.18] | Human diseases | |
| K04496 | 9 | C-terminal binding protein | Human diseases | |
| K04564 | 27 | superoxide dismutase, Fe-Mn family [EC:1.15.1.1] | Human diseases | |
| K04565 | 6 | Cu/Zn superoxide dismutase [EC:1.15.1.1] | Human diseases | |
| K04771 | 6 | serine protease Do [EC:3.4.21.107] | Human diseases | |
| K05366 | 129 | penicillin-binding protein 1A [EC:2.4.1.- 3.4.-.-] | Human diseases | |
| K05515 | 85 | penicillin-binding protein 2 | Human diseases | |
| K05692 | 2 | actin beta/gamma 1 | Human diseases | |
| K05916 | 3 | nitric oxide dioxygenase [EC:1.14.12.17] | Human diseases | |
| K07173 | 15 | S-ribosylhomocysteine lyase [EC:4.4.1.21] | Human diseases | |
| K07260 | 7 | D-alanyl-D-alanine carboxypeptidase [EC:3.4.16.4] | Human diseases | |
| K07264 | 23 | 4-amino-4-deoxy-L-arabinose transferase [EC:2.-.-.-] | Human diseases | |
| K07326 | 7 | hemolysin activation/secretion protein | Human diseases | |
| K07345 | 1 | major type 1 subunit fimbrin (pilin) | Human diseases | |
| K07347 | 11 | outer membrane usher protein | Human diseases | |
| K07637 | 1 | two-component system, OmpR family, sensor histidine kinase PhoQ | Human diseases | |
| K07640 | 6 | two-component system, OmpR family, sensor histidine kinase CpxA | Human diseases | |
| K07643 | 3 | two-component system, OmpR family, sensor histidine kinase BasS | Human diseases | |
| K07660 | 1 | two-component system, OmpR family, response regulator PhoP | Human diseases | |
| K07662 | 5 | two-component system, OmpR family, response regulator CpxR | Human diseases | |
| K07679 | 4 | two-component system, NarL family, sensor histidine kinase EvgS | Human diseases | |
| K07690 | 5 | two-component system, NarL family, response regulator EvgA | Human diseases | |
| K07771 | 1 | two-component system, OmpR family, response regulator BasR | Human diseases | |
| K07806 | 20 | UDP-4-amino-4-deoxy-L-arabinose-oxoglutarate aminotransferase | Human diseases | |
| K08218 | 18 | MFS transporter, PAT family, beta-lactamase induction signal | Human diseases | |
| K08303 | 23 | putative protease [EC:3.4.-.-] | Human diseases | |
| K08477 | 2 | outer membrane protease E [EC:3.4.21.-] | Human diseases | |
| K08641 | 17 | D-alanyl-D-alanine dipeptidase [EC:3.4.13.-] | Human diseases | |
| K08720 | 3 | outer membrane protein OmpU | Human diseases | |
| K08738 | 12 | cytochrome c | Human diseases | |
| K10011 | 7 | UDP-GlcUA decarboxylase/UDP-L-Ara4N formyltransferase | Human diseases | |
| K10012 | 8 | undecaprenyl-phosphate 4-deoxy-4-formamido-L-arabinose transferase | Human diseases | |
| K10912 | 2 | two-component system, repressor protein LuxO | Human diseases | |
| K10914 | 3 | CRP/FNR family transcriptional regulator, cyclic AMP receptor | Human diseases | |
| K10915 | 1 | CAI-1 autoinducer synthase [EC:2.3.-.-] | Human diseases | |
| K10917 | 8 | PadR family transcriptional regulator, regulatory protein AphA | Human diseases | |

| | | | | |
|---|---|---|---|---|
| K10918 | 14 | LysR family transcriptional regulator, transcriptional activator | Human diseases | |
| K10941 | 18 | sigma-54 specific transcriptional regulator, flagellar regulatory | Human diseases | |
| K10942 | 29 | two-component system, sensor histidine kinase FlrB [EC:2.7.13.3] | Human diseases | |
| K10943 | 57 | two component system, response regulator FlrC | Human diseases | |
| K11003 | 4 | hemolysin D | Human diseases | |
| K11004 | 5 | ATP-binding cassette, subfamily B, bacterial HlyB/CyaB | Human diseases | |
| K11029 | 4 | anthrax edema toxin adenylate cyclase [EC:4.6.1.1] | Human diseases | |
| K11041 | 2 | exfoliative toxin A/B | Human diseases | |
| K11418 | 13 | histone deacetylase 11 [EC:3.5.1.98] | Human diseases | |
| K11533 | 1 | fatty acid synthase, bacteria type [EC:2.3.1.-] | Human diseases | |
| K11987 | 1 | prostaglandin-endoperoxide synthase 2 [EC:1.14.99.1] | Human diseases | |
| K12264 | 2 | anaerobic nitric oxide reductase flavorubredoxin | Human diseases | |
| K12265 | 1 | nitric oxide reductase FlRd-NAD(+) reductase [EC:1.18.1.-] | Human diseases | |
| K12266 | 6 | anaerobic nitric oxide reductase transcription regulator | Human diseases | |
| K12340 | 36 | outer membrane channel protein TolC | Human diseases | |
| K12344 | 4 | 3-oxo-5-alpha-steroid 4-dehydrogenase 2 [EC:1.3.99.5] | Human diseases | |
| K12555 | 1 | penicillin-binding protein 2A [EC:2.4.1.129 2.3.2.-] | Human diseases | |
| K12688 | 2 | autotransporter serine protease [EC:3.4.21.-] | Human diseases | |
| K13014 | 2 | undecaprenyl phosphate-alpha-L-ara4FN deformylase [EC:3.5.-.-] | Human diseases | |
| K13285 | 1 | invasin B | Human diseases | |
| K13525 | 23 | transitional endoplasmic reticulum ATPase | Human diseases | |
| K13730 | 107 | internalin A | Human diseases | |
| K13735 | 100 | adhesin/invasin | Human diseases | |
| K13741 | 1 | guanine nucleotide exchange factor SopE | Human diseases | |
| K13742 | 1 | protein IpgB1 | Human diseases | |
| K13771 | 2 | Rrf2 family transcriptional regulator, nitric oxide-sensitive | Human diseases | |
| K13963 | 11 | serpin B | Human diseases | |
| | | | | |
| | | | | |
| | | | | |
| | | | | |
| | | | | |
| | | | | |
| | | | | |
| | | | | |
| | | | | |
| | | | | |

| KEGG ID | Sequences | KEGG pathway | KEGG category |
|---|---|---|---|
| K00031 | 37 | isocitrate dehydrogenase [EC:1.1.1.42] | Cellular Processes |
| K00232 | 1 | acyl-CoA oxidase [EC:1.3.3.6] | Cellular Processes |
| K00273 | 15 | D-amino-acid oxidase [EC:1.4.3.3] | Cellular Processes |
| K00477 | 2 | phytanoyl-CoA hydroxylase [EC:1.14.11.18] | Cellular Processes |
| K00494 | 70 | alkanal monooxygenase (FMN-linked) [EC:1.14.14.3] | Cellular Processes |
| K00575 | 79 | chemotaxis protein methyltransferase CheR [EC:2.1.1.80] | Cellular Processes |
| K00803 | 23 | alkyldihydroxyacetonephosphate synthase [EC:2.5.1.26] | Cellular Processes |
| K00869 | 1 | mevalonate kinase [EC:2.7.1.36] | Cellular Processes |
| K01114 | 33 | phospholipase C [EC:3.1.4.3] | Cellular Processes |
| K01115 | 8 | phospholipase D [EC:3.1.4.4] | Cellular Processes |
| K01132 | 17 | N-acetylgalactosamine-6-sulfatase [EC:3.1.6.4] | Cellular Processes |
| K01134 | 23 | arylsulfatase A [EC:3.1.6.8] | Cellular Processes |
| K01135 | 17 | arylsulfatase B [EC:3.1.6.12] | Cellular Processes |
| K01136 | 8 | iduronate 2-sulfatase [EC:3.1.6.13] | Cellular Processes |
| K01137 | 10 | N-acetylglucosamine-6-sulfatase [EC:3.1.6.14] | Cellular Processes |
| K01173 | 1 | endonuclease [EC:3.1.30.-] | Cellular Processes |
| K01186 | 2 | sialidase-1 [EC:3.2.1.18] | Cellular Processes |
| K01192 | 9 | beta-mannosidase [EC:3.2.1.25] | Cellular Processes |
| K01195 | 8 | beta-glucuronidase [EC:3.2.1.31] | Cellular Processes |
| K01201 | 13 | glucosylceramidase [EC:3.2.1.45] | Cellular Processes |
| K01218 | 6 | mannan endo-1,4-beta-mannosidase [EC:3.2.1.78] | Cellular Processes |
| K01318 | 1 | glutamyl endopeptidase [EC:3.4.21.19] | Cellular Processes |
| K01338 | 158 | ATP-dependent Lon protease [EC:3.4.21.53] | Cellular Processes |
| K01342 | 54 | subtilisin [EC:3.4.21.62] | Cellular Processes |
| K01358 | 30 | ATP-dependent Clp protease, protease subunit [EC:3.4.21.92] | Cellular Processes |
| K01444 | 9 | N4-(beta-N-acetylglucosaminyl)-L-asparaginase [EC:3.5.1.26] | Cellular Processes |
| K01497 | 24 | GTP cyclohydrolase II [EC:3.5.4.25] | Cellular Processes |
| K01578 | 19 | malonyl-CoA decarboxylase [EC:4.1.1.9] | Cellular Processes |
| K01580 | 8 | glutamate decarboxylase [EC:4.1.1.15] | Cellular Processes |
| K01626 | 26 | 3-deoxy-7-phosphoheptulonate synthase [EC:2.5.1.54] | Cellular Processes |
| K01635 | 10 | tagatose 1,6-diphosphate aldolase [EC:4.1.2.40] | Cellular Processes |
| K01640 | 23 | hydroxymethylglutaryl-CoA lyase [EC:4.1.3.4] | Cellular Processes |
| K01657 | 59 | anthranilate synthase component I [EC:4.1.3.27] | Cellular Processes |
| K01658 | 28 | anthranilate synthase component II [EC:4.1.3.27] | Cellular Processes |
| K01728 | 1 | pectate lyase [EC:4.2.2.2] | Cellular Processes |
| K01768 | 403 | adenylate cyclase [EC:4.6.1.1] | Cellular Processes |
| K01796 | 35 | alpha-methylacyl-CoA racemase [EC:5.1.99.4] | Cellular Processes |
| K01897 | 294 | long-chain acyl-CoA synthetase [EC:6.2.1.3] | Cellular Processes |
| K01995 | 126 | branched-chain amino acid transport system ATP-binding protein | Cellular Processes |
| K01996 | 125 | branched-chain amino acid transport system ATP-binding protein | Cellular Processes |
| K01997 | 153 | branched-chain amino acid transport system permease protein | Cellular Processes |
| K01998 | 326 | branched-chain amino acid transport system permease protein | Cellular Processes |
| K01999 | 254 | branched-chain amino acid transport system substrate-binding protein | Cellular Processes |
| K02031 | 66 | peptide/nickel transport system ATP-binding protein | Cellular Processes |
| K02032 | 185 | peptide/nickel transport system ATP-binding protein | Cellular Processes |
| K02033 | 207 | peptide/nickel transport system permease protein | Cellular Processes |
| K02034 | 165 | peptide/nickel transport system permease protein | Cellular Processes |
| K02035 | 407 | peptide/nickel transport system substrate-binding protein | Cellular Processes |
| K02052 | 32 | putative spermidine/putrescine transport system ATP-binding protein | Cellular Processes |
| K02053 | 39 | putative spermidine/putrescine transport system permease protein | Cellular Processes |
| K02054 | 31 | putative spermidine/putrescine transport system permease protein | Cellular Processes |
| K02055 | 46 | putative spermidine/putrescine transport system substrate-binding | Cellular Processes |
| K02313 | 51 | chromosomal replication initiator protein | Cellular Processes |
| K02314 | 78 | replicative DNA helicase [EC:3.6.1.-] | Cellular Processes |
| K02386 | 1 | flagella basal body P-ring formation protein FlgA | Cellular Processes |
| K02387 | 12 | flagellar basal-body rod protein FlgB | Cellular Processes |
| K02388 | 133 | flagellar basal-body rod protein FlgC | Cellular Processes |
| K02389 | 14 | flagellar basal-body rod modification protein FlgD | Cellular Processes |
| K02390 | 14 | flagellar hook protein FlgE | Cellular Processes |

| | | | |
|---|---|---|---|
| K02391 | 4 | flagellar basal-body rod protein FlgF | Cellular Processes |
| K02392 | 15 | flagellar basal-body rod protein FlgG | Cellular Processes |
| K02393 | 4 | flagellar L-ring protein precursor FlgH | Cellular Processes |
| K02394 | 6 | flagellar P-ring protein precursor FlgI | Cellular Processes |
| K02396 | 12 | flagellar hook-associated protein 1 FlgK | Cellular Processes |
| K02397 | 8 | flagellar hook-associated protein 3 FlgL | Cellular Processes |
| K02400 | 16 | flagellar biosynthesis protein FlhA | Cellular Processes |
| K02401 | 8 | flagellar biosynthetic protein FlhB | Cellular Processes |
| K02402 | 1 | flagellar transcriptional activator FlhC | Cellular Processes |
| K02406 | 10 | flagellin | Cellular Processes |
| K02407 | 17 | flagellar hook-associated protein 2 | Cellular Processes |
| K02408 | 2 | flagellar hook-basal body complex protein FliE | Cellular Processes |
| K02409 | 14 | flagellar M-ring protein FliF | Cellular Processes |
| K02410 | 16 | flagellar motor switch protein FliG | Cellular Processes |
| K02411 | 3 | flagellar assembly protein FliH | Cellular Processes |
| K02412 | 12 | flagellum-specific ATP synthase [EC:3.6.3.14] | Cellular Processes |
| K02413 | 1 | flagellar FliJ protein | Cellular Processes |
| K02414 | 1 | flagellar hook-length control protein FliK | Cellular Processes |
| K02416 | 15 | flagellar motor switch protein FliM | Cellular Processes |
| K02417 | 6 | flagellar motor switch protein FliN/FliY | Cellular Processes |
| K02418 | 2 | flagellar protein FliO/FliZ | Cellular Processes |
| K02419 | 7 | flagellar biosynthetic protein FliP | Cellular Processes |
| K02420 | 2 | flagellar biosynthetic protein FliQ | Cellular Processes |
| K02421 | 2 | flagellar biosynthetic protein FliR | Cellular Processes |
| K02422 | 3 | flagellar protein FliS | Cellular Processes |
| K02488 | 217 | two-component system, cell cycle response regulator | Cellular Processes |
| K02490 | 30 | two-component system, response regulator, stage 0 sporulation | Cellular Processes |
| K02556 | 5 | chemotaxis protein MotA | Cellular Processes |
| K02557 | 23 | chemotaxis protein MotB | Cellular Processes |
| K02563 | 30 | UDP-N-acetylglucosamine--N-acetylmuramyl-(pentapeptide) | Cellular Processes |
| K02651 | 5 | pilus assembly protein Flp/PilA | Cellular Processes |
| K03070 | 74 | preprotein translocase subunit SecA | Cellular Processes |
| K03071 | 7 | preprotein translocase subunit SecB | Cellular Processes |
| K03073 | 2 | preprotein translocase subunit SecE | Cellular Processes |
| K03075 | 3 | preprotein translocase subunit SecG | Cellular Processes |
| K03076 | 36 | preprotein translocase subunit SecY | Cellular Processes |
| K03100 | 92 | signal peptidase I [EC:3.4.21.89] | Cellular Processes |
| K03106 | 47 | signal recognition particle subunit SRP54 | Cellular Processes |
| K03110 | 23 | fused signal recognition particle receptor | Cellular Processes |
| K03210 | 16 | preprotein translocase subunit YajC | Cellular Processes |
| K03217 | 71 | preprotein translocase subunit YidC | Cellular Processes |
| K03386 | 31 | peroxiredoxin (alkyl hydroperoxide reductase subunit C) | Cellular Processes |
| K03406 | 186 | methyl-accepting chemotaxis protein | Cellular Processes |
| K03407 | 82 | two-component system, chemotaxis family, sensor kinase CheA | Cellular Processes |
| K03408 | 21 | purine-binding chemotaxis protein CheW | Cellular Processes |
| K03410 | 4 | chemotaxis protein CheC | Cellular Processes |
| K03411 | 16 | chemotaxis protein CheD [EC:3.5.1.44] | Cellular Processes |
| K03412 | 64 | two-component system, chemotaxis family, response regulator CheB | Cellular Processes |
| K03413 | 117 | two-component system, chemotaxis family, response regulator CheY | Cellular Processes |
| K03414 | 3 | chemotaxis protein CheZ | Cellular Processes |
| K03426 | 21 | NAD+ diphosphatase [EC:3.6.1.22] | Cellular Processes |
| K03531 | 44 | cell division protein FtsZ | Cellular Processes |
| K03544 | 21 | ATP-dependent Clp protease ATP-binding subunit ClpX | Cellular Processes |
| K03588 | 46 | cell division protein FtsW | Cellular Processes |
| K03589 | 17 | cell division protein FtsQ | Cellular Processes |
| K03590 | 36 | cell division protein FtsA | Cellular Processes |
| K03666 | 10 | host factor-I protein | Cellular Processes |
| K03776 | 16 | aerotaxis receptor | Cellular Processes |
| K03781 | 12 | catalase [EC:1.11.1.6] | Cellular Processes |
| K04562 | 441 | flagellar biosynthesis protein FlhG | Cellular Processes |

| | | | |
|---|---|---|---|
| K04564 | 27 | superoxide dismutase, Fe-Mn family [EC:1.15.1.1] | Cellular Processes |
| K04565 | 6 | Cu/Zn superoxide dismutase [EC:1.15.1.1] | Cellular Processes |
| K05692 | 2 | actin beta/gamma 1 | Cellular Processes |
| K05874 | 21 | methyl-accepting chemotaxis protein I, serine sensor receptor | Cellular Processes |
| K05875 | 8 | methyl-accepting chemotaxis protein II, aspartate sensor receptor | Cellular Processes |
| K05876 | 2 | methyl-accepting chemotaxis protein III, ribose and galactose sensor | Cellular Processes |
| K06985 | 5 | aspartyl protease family protein | Cellular Processes |
| K06998 | 53 | Unclassified; K06998 | Cellular Processes |
| K07173 | 15 | S-ribosylhomocysteine lyase [EC:4.4.1.21] | Cellular Processes |
| K07177 | 6 | PDZ domain-containing protein | Cellular Processes |
| K07645 | 33 | two-component system, OmpR family, sensor histidine kinase QseC | Cellular Processes |
| K07666 | 3 | two-component system, OmpR family, response regulator QseB | Cellular Processes |
| K07667 | 53 | two-component system, OmpR family, KDP operon response regulator | Cellular Processes |
| K07680 | 15 | two-component system, NarL family, sensor histidine kinase ComP | Cellular Processes |
| K07691 | 24 | two-component system, NarL family, competent response regulator Com | Cellular Processes |
| K07692 | 37 | two-component system, NarL family, response regulator DegU | Cellular Processes |
| K07699 | 1 | two-component system, response regulator, stage 0 sporulation | Cellular Processes |
| K07707 | 4 | two-component system, AgrA family, response regulator AgrA | Cellular Processes |
| K07711 | 10 | two-component system, NtrC family, sensor histidine kinase YfhK | Cellular Processes |
| K07715 | 78 | two-component system, NtrC family, response regulator YfhA | Cellular Processes |
| K07716 | 16 | two-component system, cell cycle sensor histidine kinase PleC | Cellular Processes |
| K07782 | 1 | LuxR family transcriptional regulator | Cellular Processes |
| K08139 | 13 | MFS transporter, SP family, sugar:H+ symporter | Cellular Processes |
| K08605 | 1 | coccolysin [EC:3.4.24.30] | Cellular Processes |
| K08738 | 12 | cytochrome c | Cellular Processes |
| K08777 | 1 | neutral peptidase B [EC:3.4.24.-] | Cellular Processes |
| K09936 | 3 | hypothetical protein | Cellular Processes |
| K10108 | 11 | maltose/maltodextrin transport system substrate-binding protein | Cellular Processes |
| K10439 | 21 | ribose transport system substrate-binding protein | Cellular Processes |
| K10715 | 7 | two-component system, sensor histidine kinase RpfC [EC:2.7.13.3] | Cellular Processes |
| K10912 | 2 | two-component system, repressor protein LuxO | Cellular Processes |
| K10914 | 3 | CRP/FNR family transcriptional regulator, cyclic AMP receptor | Cellular Processes |
| K10915 | 1 | CAI-1 autoinducer synthase [EC:2.3.-.-] | Cellular Processes |
| K10917 | 8 | PadR family transcriptional regulator, regulatory protein AphA | Cellular Processes |
| K11031 | 2 | thiol-activated cytolysin | Cellular Processes |
| K11357 | 7 | two-component system, cell cycle sensor histidine kinase DivJ | Cellular Processes |
| K11443 | 7 | two-component system, cell cycle response regulator DivK | Cellular Processes |
| K11749 | 54 | regulator of sigma E protease [EC:3.4.24.-] | Cellular Processes |
| K11752 | 32 | diaminohydroxyphosphoribosylaminopyrimidine deaminase / | Cellular Processes |
| K12257 | 30 | SecD/SecF fusion protein | Cellular Processes |
| K12368 | 17 | dipeptide transport system substrate-binding protein | Cellular Processes |
| K12373 | 31 | beta-hexosaminidase [EC:3.2.1.52] | Cellular Processes |
| K13063 | 1 | phenazine biosynthesis protein phzE [EC:2.6.1.86] | Cellular Processes |
| K13075 | 16 | N-acyl homoserine lactone hydrolase [EC:3.1.1.81] | Cellular Processes |
| K13237 | 3 | peroxisomal 2,4-dienoyl-CoA reductase [EC:1.3.1.34] | Cellular Processes |
| K13245 | 7 | c-di-GMP-specific phosphodiesterase [EC:3.1.4.52] | Cellular Processes |
| K13277 | 3 | minor extracellular protease Epr [EC:3.4.21.-] | Cellular Processes |
| K13581 | 4 | modification methylase [EC:2.1.1.72] | Cellular Processes |
| K13582 | 2 | localization factor PodJL | Cellular Processes |
| K13583 | 3 | GcrA cell cycle regulator | Cellular Processes |
| K13584 | 1 | two-component system, cell cycle response regulator CtrA | Cellular Processes |
| K13587 | 41 | two-component system, cell cycle sensor histidine kinase and | Cellular Processes |
| K13588 | 1 | histidine phosphotransferase ChpT | Cellular Processes |
| K13589 | 2 | two-component system, cell cycle response regulator CpdR | Cellular Processes |
| K13590 | 30 | diguanylate cyclase | Cellular Processes |
| K13592 | 1 | regulator of CtrA degradation | Cellular Processes |
| K13593 | 1 | cyclic-di-GMP phosphodiesterase, flagellum assembly factor TipF | Cellular Processes |
| K13815 | 21 | two-component system, response regulator RpfG | Cellular Processes |
| K13816 | 7 | DSF synthase | Cellular Processes |
| K13820 | 1 | flagellar biosynthetic protein FliR/FlhB | Cellular Processes |

| | | | | |
|---|---|---|---|---|
| K13924 | 124 | two-component system, chemotaxis family, CheB/CheR fusion protein | Cellular Processes | |
| | | | | |
| | | | | |
| | | | | |
| | | | | |
| | | | | |
| | | | | |
| | | | | |
| | | | | |
| | | | | |
| | | | | |

| KEGG ID | Sequences | KEGG pathway | KEGG category |
|---|---|---|---|
| K00016 | 26 | L-lactate dehydrogenase [EC:1.1.1.27] | Organismal system |
| K00021 | 14 | 3-hydroxy-3-methylglutaryl-CoA reductase [EC:1.1.1.34] | Organismal system |
| K00044 | 10 | estradiol 17beta-dehydrogenase [EC:1.1.1.62] | Organismal system |
| K00071 | 3 | 11beta-hydroxysteroid dehydrogenase [EC:1.1.1.146] | Organismal system |
| K00161 | 65 | pyruvate dehydrogenase E1 component subunit alpha [EC:1.2.4.1] | Organismal system |
| K00162 | 66 | pyruvate dehydrogenase E1 component subunit beta [EC:1.2.4.1] | Organismal system |
| K00232 | 1 | acyl-CoA oxidase [EC:1.3.3.6] | Organismal system |
| K00249 | 223 | acyl-CoA dehydrogenase [EC:1.3.99.3] | Organismal system |
| K00255 | 14 | long-chain-acyl-CoA dehydrogenase [EC:1.3.99.13] | Organismal system |
| K00261 | 38 | glutamate dehydrogenase (NAD(P)+) [EC:1.4.1.3] | Organismal system |
| K00274 | 21 | monoamine oxidase [EC:1.4.3.4] | Organismal system |
| K00383 | 19 | glutathione reductase (NADPH) [EC:1.8.1.7] | Organismal system |
| K00411 | 10 | ubiquinol-cytochrome c reductase iron-sulfur subunit [EC:1.10.2.2] | Organismal system |
| K00412 | 35 | ubiquinol-cytochrome c reductase cytochrome b subunit [EC:1.10.2.2] | Organismal system |
| K00413 | 8 | ubiquinol-cytochrome c reductase cytochrome c1 subunit [EC:1.10.2.2] | Organismal system |
| K00432 | 7 | glutathione peroxidase [EC:1.11.1.9] | Organismal system |
| K00507 | 40 | stearoyl-CoA desaturase (delta-9 desaturase) [EC:1.14.19.1] | Organismal system |
| K00660 | 12 | chalcone synthase [EC:2.3.1.74] | Organismal system |
| K00688 | 90 | starch phosphorylase [EC:2.4.1.1] | Organismal system |
| K00693 | 14 | glycogen(starch) synthase [EC:2.4.1.11] | Organismal system |
| K00863 | 8 | dihydroxyacetone kinase [EC:2.7.1.29] | Organismal system |
| K00864 | 41 | glycerol kinase [EC:2.7.1.30] | Organismal system |
| K00873 | 44 | pyruvate kinase [EC:2.7.1.40] | Organismal system |
| K00965 | 24 | UDPglucose--hexose-1-phosphate uridylyltransferase [EC:2.7.7.12] | Organismal system |
| K01054 | 3 | acylglycerol lipase [EC:3.1.1.23] | Organismal system |
| K01114 | 33 | phospholipase C [EC:3.1.4.3] | Organismal system |
| K01115 | 8 | phospholipase D [EC:3.1.4.4] | Organismal system |
| K01176 | 36 | alpha-amylase [EC:3.2.1.1] | Organismal system |
| K01253 | 35 | microsomal epoxide hydrolase [EC:3.3.2.9] | Organismal system |
| K01278 | 26 | dipeptidyl-peptidase 4 [EC:3.4.14.5] | Organismal system |
| K01283 | 4 | peptidyl-dipeptidase A [EC:3.4.15.1] | Organismal system |
| K01312 | 9 | trypsin [EC:3.4.21.4] | Organismal system |
| K01322 | 103 | prolyl oligopeptidase [EC:3.4.21.26] | Organismal system |
| K01325 | 2 | tissue kallikrein [EC:3.4.21.35] | Organismal system |
| K01344 | 1 | protein C (activated) [EC:3.4.21.69] | Organismal system |
| K01358 | 30 | ATP-dependent Clp protease, protease subunit [EC:3.4.21.92] | Organismal system |
| K01389 | 2 | neprilysin [EC:3.4.24.11] | Organismal system |
| K01392 | 9 | thimet oligopeptidase [EC:3.4.24.15] | Organismal system |
| K01425 | 10 | glutaminase [EC:3.5.1.2] | Organismal system |
| K01539 | 6 | sodium/potassium-transporting ATPase subunit alpha [EC:3.6.3.9] | Organismal system |
| K01580 | 8 | glutamate decarboxylase [EC:4.1.1.15] | Organismal system |
| K01593 | 61 | aromatic-L-amino-acid decarboxylase [EC:4.1.1.28] | Organismal system |
| K01596 | 30 | phosphoenolpyruvate carboxykinase (GTP) [EC:4.1.1.32] | Organismal system |
| K01768 | 403 | adenylate cyclase [EC:4.6.1.1] | Organismal system |
| K01834 | 100 | phosphoglycerate mutase [EC:5.4.2.1] | Organismal system |
| K01897 | 294 | long-chain acyl-CoA synthetase [EC:6.2.1.3] | Organismal system |
| K01915 | 141 | glutamine synthetase [EC:6.3.1.2] | Organismal system |
| K02406 | 10 | flagellin | Organismal system |
| K03695 | 75 | ATP-dependent Clp protease ATP-binding subunit ClpB | Organismal system |
| K03781 | 12 | catalase [EC:1.11.1.6] | Organismal system |
| K03841 | 11 | fructose-1,6-bisphosphatase I [EC:3.1.3.11] | Organismal system |
| K04043 | 64 | molecular chaperone DnaK | Organismal system |
| K04077 | 43 | chaperonin GroEL | Organismal system |
| K04079 | 17 | molecular chaperone HtpG | Organismal system |
| K04564 | 27 | superoxide dismutase, Fe-Mn family [EC:1.15.1.1] | Organismal system |
| K04565 | 6 | Cu/Zn superoxide dismutase [EC:1.15.1.1] | Organismal system |
| K05692 | 2 | actin beta/gamma 1 | Organismal system |
| K06027 | 29 | vesicle-fusing ATPase [EC:3.6.4.6] | Organismal system |
| K07192 | 11 | flotillin | Organismal system |

| | | | | |
|---|---|---|---|---|
| K07213 | 6 | Unclassified; K07213 | Organismal system | |
| K11031 | 2 | thiol-activated cytolysin | Organismal system | |
| K11987 | 1 | prostaglandin-endoperoxide synthase 2 [EC:1.14.99.1] | Organismal system | |
| K12340 | 36 | outer membrane channel protein TolC | Organismal system | |
| K13408 | 2 | membrane fusion protein RaxA | Organismal system | |
| K13409 | 1 | ATP-binding cassette, subfamily B, bacterial RaxB | Organismal system | |
| | | | | |
| | | | | |
| | | | | |
| | | | | |
| | | | | |
| | | | | |
| | | | | |
| | | | | |
| | | | | |
| | | | | |

| KEGG category | Counts |
|---|---|
| **Metabolism** | 41140 |
| **Environmetal Information Processing** | 11379 |
| **Genetic Information Processing** | 9314 |
| **Human Diseases** | 4080 |
| **Cellular Processes** | 7038 |
| **Organismal Systems** | 2542 |

| GO ID | Sequence count | GO terms |
|---|---|---|
| GO:00000 | 31 | phosphopyruvate hydratase complex |
| GO:00000 | 29 | mannosyltransferase activity |
| GO:00000 | 7 | transition metal ion transport |
| GO:00000 | 82 | tRNA binding |
| GO:00000 | 2 | fatty-acyl-CoA binding |
| GO:00001 | 15 | sulfate assimilation |
| GO:00001 | 133 | histidine biosynthetic process |
| GO:00001 | 28 | recombinase activity |
| GO:00001 | 173 | phosphorelay sensor kinase activity |
| GO:00001 | 63 | phosphorelay response regulator activity |
| GO:00001 | 1186 | phosphorelay signal transduction system |
| GO:00001 | 1170 | nucleotide binding |
| GO:00002 | 9 | allantoin catabolic process |
| GO:00002 | 47 | polysaccharide biosynthetic process |
| GO:00002 | 2 | polysaccharide catabolic process |
| GO:00002 | 137 | magnesium ion binding |
| GO:00003 | 3 | 3-hydroxyanthranilate 3,4-dioxygenase activity |
| GO:00004 | 34 | protein peptidyl-prolyl isomerization |
| GO:00007 | 2 | telomere maintenance |
| GO:00007 | 21 | adenyl-nucleotide exchange factor activity |
| GO:00009 | 36 | cell morphogenesis |
| GO:00009 | 6 | barrier septum assembly |
| GO:00010 | 2 | bacterial-type RNA polymerase core enzyme binding |
| GO:00011 | 1 | RNA polymerase II transcription cofactor activity |
| GO:00015 | 125 | pseudouridine synthesis |
| GO:00018 | 20 | nucleoside binding |
| GO:00021 | 2 | tRNA wobble position uridine thiolation |
| GO:00021 | 47 | aminoacyl-tRNA editing activity |
| GO:00033 | 240 | amino acid transmembrane transport |
| GO:00036 | 429 | nucleic acid binding |
| GO:00036 | 2604 | DNA binding |
| GO:00036 | 61 | DNA helicase activity |
| GO:00036 | 127 | damaged DNA binding |
| GO:00036 | 59 | single-stranded DNA binding |
| GO:00037 | 821 | transcription factor activity, sequence-specific DNA binding |
| GO:00037 | 4 | transcription coactivator activity |
| GO:00037 | 541 | RNA binding |
| GO:00037 | 77 | double-stranded RNA binding |
| GO:00037 | 2 | mRNA binding |
| GO:00037 | 487 | structural constituent of ribosome |
| GO:00037 | 22 | translation initiation factor activity |
| GO:00037 | 34 | translation elongation factor activity |
| GO:00037 | 41 | translation release factor activity |
| GO:00037 | 34 | peptidyl-prolyl cis-trans isomerase activity |
| GO:00037 | 15 | motor activity |
| GO:00037 | 5 | lysozyme activity |
| GO:00038 | 4368 | catalytic activity |
| GO:00038 | 136 | gamma-glutamyltransferase activity |
| GO:00038 | 1 | 1-alkyl-2-acetylglycerophosphocholine esterase activity |
| GO:00038 | 17 | 2-amino-4-hydroxy-6-hydroxymethyldihydropteridine diphosphokinase activity |
| GO:00038 | 13 | 3-deoxy-7-phosphoheptulonate synthase activity |
| GO:00038 | 9 | 3-beta-hydroxy-delta5-steroid dehydrogenase activity |
| GO:00038 | 20 | 3-dehydroquinate dehydratase activity |
| GO:00038 | 2 | 3-dehydroquinate synthase activity |
| GO:00038 | 98 | 3-hydroxyacyl-CoA dehydrogenase activity |
| GO:00038 | 26 | 3-methyl-2-oxobutanoate hydroxymethyltransferase activity |
| GO:00038 | 74 | 5-methyltetrahydropteroyltriglutamate-homocysteine S-methyltransferase activity |
| GO:00038 | 54 | 6-phosphofructokinase activity |
| GO:00038 | 26 | ATP phosphoribosyltransferase activity |
| GO:00038 | 30 | CTP synthase activity |
| GO:00038 | 11 | D-arabinono-1,4-lactone oxidase activity |
| GO:00038 | 181 | DNA-directed DNA polymerase activity |
| GO:00038 | 69 | DNA primase activity |
| GO:00038 | 190 | DNA-directed RNA polymerase activity |
| GO:00039 | 14 | alkylbase DNA N-glycosylase activity |
| GO:00039 | 64 | DNA-(apurinic or apyrimidinic site) lyase activity |
| GO:00039 | 59 | DNA ligase (ATP) activity |
| GO:00039 | 63 | DNA ligase (NAD+) activity |
| GO:00039 | 28 | DNA photolyase activity |
| GO:00039 | 77 | DNA topoisomerase activity |
| GO:00039 | 158 | DNA topoisomerase type II (ATP-hydrolyzing) activity |
| GO:00039 | 27 | FMN adenylyltransferase activity |
| GO:00039 | 23 | GMP synthase (glutamine-hydrolyzing) activity |
| GO:00039 | 62 | GTPase activity |
| GO:00039 | 8 | GTP cyclohydrolase activity |
| GO:00039 | 23 | GTP cyclohydrolase II activity |
| GO:00039 | 28 | IMP cyclohydrolase activity |
| GO:00039 | 42 | N-acetyl-gamma-glutamyl-phosphate reductase activity |
| GO:00039 | 35 | NAD+ kinase activity |
| GO:00039 | 19 | acetyl-CoA carboxylase activity |
| GO:00039 | 2 | acid phosphatase activity |
| GO:00039 | 7 | aconitate hydratase activity |
| GO:00039 | 16 | acyl-CoA dehydrogenase activity |
| GO:00039 | 1 | acyl-CoA oxidase activity |
| GO:00040 | 28 | adenosylhomocysteinase activity |
| GO:00040 | 3 | adenosylmethionine decarboxylase activity |
| GO:00040 | 43 | adenylosuccinate synthase activity |
| GO:00040 | 15 | adenylylsulfate kinase activity |
| GO:00040 | 8 | allantoicase activity |
| GO:00040 | 2 | amidase activity |
| GO:00040 | 27 | aminoacyl-tRNA hydrolase activity |
| GO:00040 | 30 | argininosuccinate synthase activity |
| GO:00040 | 7 | arginyltransferase activity |
| GO:00040 | 24 | arylformamidase activity |
| GO:00040 | 152 | asparagine synthase (glutamine-hydrolyzing) activity |
| GO:00040 | 6 | aspartate 1-decarboxylase activity |
| GO:00040 | 6 | aspartate-ammonia ligase activity |
| GO:00040 | 24 | carbonate dehydratase activity |
| GO:00040 | 11 | catalase activity |
| GO:00041 | 34 | chorismate synthase activity |
| GO:00041 | 7 | coproporphyrinogen oxidase activity |
| GO:00041 | 2 | 3',5'-cyclic-AMP phosphodiesterase activity |
| GO:00041 | 2 | cytidine deaminase activity |

| | | | | |
|---|---|---|---|---|
| GO:00041 | 18 | cytidylate kinase activity | | |
| GO:00041 | 185 | cytochrome-c oxidase activity | | |
| GO:00041 | 68 | 4-alpha-glucanotransferase activity | | |
| GO:00041 | 17 | amylo-alpha-1,6-glucosidase activity | | |
| GO:00041 | 19 | dephospho-CoA kinase activity | | |
| GO:00041 | 5 | diacylglycerol kinase activity | | |
| GO:00041 | 59 | diacylglycerol O-acyltransferase activity | | |
| GO:00041 | 12 | dihydrofolate reductase activity | | |
| GO:00041 | 13 | dihydroneopterin aldolase activity | | |
| GO:00041 | 37 | electron-transferring-flavoprotein dehydrogenase activity | | |
| GO:00041 | 107 | ATP-dependent peptidase activity | | |
| GO:00041 | 93 | aminopeptidase activity | | |
| GO:00041 | 78 | metallocarboxypeptidase activity | | |
| GO:00041 | 44 | serine-type carboxypeptidase activity | | |
| GO:00041 | 19 | aspartic-type endopeptidase activity | | |
| GO:00041 | 22 | cysteine-type endopeptidase activity | | |
| GO:00042 | 393 | metalloendopeptidase activity | | |
| GO:00042 | 422 | serine-type endopeptidase activity | | |
| GO:00042 | 25 | threonine-type endopeptidase activity | | |
| GO:00043 | 3 | 3-oxoacyl-[acyl-carrier-protein] synthase activity | | |
| GO:00043 | 32 | ferrochelatase activity | | |
| GO:00043 | 22 | formate-tetrahydrofolate ligase activity | | |
| GO:00043 | 16 | fructose-bisphosphate aldolase activity | | |
| GO:00043 | 9 | glucokinase activity | | |
| GO:00043 | 39 | glucose-6-phosphate dehydrogenase activity | | |
| GO:00043 | 52 | glucose-6-phosphate isomerase activity | | |
| GO:00043 | 4 | glucosylceramidase activity | | |
| GO:00043 | 37 | glutamate dehydrogenase (NAD+) activity | | |
| GO:00043 | 115 | glutamate-ammonia ligase activity | | |
| GO:00043 | 48 | glutamate-cysteine ligase activity | | |
| GO:00043 | 31 | glutamate N-acetyltransferase activity | | |
| GO:00043 | 10 | glutaminase activity | | |
| GO:00043 | 21 | glutathione synthase activity | | |
| GO:00043 | 17 | glycerol-3-phosphate dehydrogenase [NAD+] activity | | |
| GO:00043 | 14 | glycerone kinase activity | | |
| GO:00043 | 63 | glycine dehydrogenase (decarboxylating) activity | | |
| GO:00043 | 5 | guanylate cyclase activity | | |
| GO:00043 | 43 | histidinol dehydrogenase activity | | |
| GO:00044 | 27 | homogentisate 1,2-dioxygenase activity | | |
| GO:00044 | 2 | hydroxyethylthiazole kinase activity | | |
| GO:00044 | 28 | hydroxymethylbilane synthase activity | | |
| GO:00044 | 17 | hydroxymethylglutaryl-CoA reductase (NADPH) activity | | |
| GO:00044 | 14 | imidazoleglycerol-phosphate dehydratase activity | | |
| GO:00044 | 19 | indole-3-glycerol-phosphate synthase activity | | |
| GO:00044 | 48 | inorganic diphosphatase activity | | |
| GO:00044 | 10 | isocitrate dehydrogenase (NADP+) activity | | |
| GO:00044 | 12 | isocitrate lyase activity | | |
| GO:00044 | 16 | ketol-acid reductoisomerase activity | | |
| GO:00044 | 47 | malate dehydrogenase (decarboxylating) (NAD+) activity | | |
| GO:00044 | 22 | malate synthase activity | | |
| GO:00044 | 19 | mannose-6-phosphate isomerase activity | | |
| GO:00044 | 19 | methionine adenosyltransferase activity | | |
| GO:00044 | 33 | methylenetetrahydrofolate dehydrogenase (NADP+) activity | | |
| GO:00044 | 53 | methylenetetrahydrofolate reductase (NAD(P)H) activity | | |
| GO:00044 | 1 | monooxygenase activity | | |
| GO:00044 | 6 | N,N-dimethylaniline monooxygenase activity | | |
| GO:00045 | 1 | squalene monooxygenase activity | | |
| GO:00045 | 12 | inositol-3-phosphate synthase activity | | |
| GO:00045 | 35 | nicotinate-nucleotide diphosphorylase (carboxylating) activity | | |
| GO:00045 | 2 | nitric-oxide synthase activity | | |
| GO:00045 | 23 | nuclease activity | | |
| GO:00045 | 101 | endonuclease activity | | |
| GO:00045 | 19 | endodeoxyribonuclease activity | | |
| GO:00045 | 9 | endoribonuclease activity | | |
| GO:00045 | 10 | RNA-DNA hybrid ribonuclease activity | | |
| GO:00045 | 26 | ribonuclease III activity | | |
| GO:00045 | 18 | ribonuclease P activity | | |
| GO:00045 | 15 | nucleoside diphosphate kinase activity | | |
| GO:00045 | 438 | hydrolase activity, hydrolyzing O-glycosyl compounds | | |
| GO:00045 | 5 | alpha,alpha-trehalase activity | | |
| GO:00045 | 22 | alpha-galactosidase activity | | |
| GO:00045 | 7 | alpha-mannosidase activity | | |
| GO:00045 | 18 | alpha-L-fucosidase activity | | |
| GO:00045 | 5 | beta-fructofuranosidase activity | | |
| GO:00045 | 13 | beta-galactosidase activity | | |
| GO:00045 | 1 | chitinase activity | | |
| GO:00045 | 2 | mannosyl-oligosaccharide 1,2-alpha-mannosidase activity | | |
| GO:00045 | 5 | sucrose alpha-glucosidase activity | | |
| GO:00045 | 28 | orotidine-5'-phosphate decarboxylase activity | | |
| GO:00045 | 21 | pantoate-beta-alanine ligase activity | | |
| GO:00045 | 30 | pantothenate kinase activity | | |
| GO:00046 | 34 | peroxidase activity | | |
| GO:00046 | 7 | glutathione peroxidase activity | | |
| GO:00046 | 14 | phosphatidylserine decarboxylase activity | | |
| GO:00046 | 30 | phosphoenolpyruvate carboxykinase activity | | |
| GO:00046 | 20 | phosphoenolpyruvate carboxykinase (ATP) activity | | |
| GO:00046 | 138 | phosphogluconate dehydrogenase (decarboxylating) activity | | |
| GO:00046 | 35 | phosphoglycerate kinase activity | | |
| GO:00046 | 13 | phosphoglycerate mutase activity | | |
| GO:00046 | 8 | phospholipase activity | | |
| GO:00046 | 31 | phosphopyruvate hydratase activity | | |
| GO:00046 | 8 | phosphoribosyl-AMP cyclohydrolase activity | | |
| GO:00046 | 21 | phosphoribosylanthranilate isomerase activity | | |
| GO:00046 | 27 | phosphoribosylaminoimidazolecarboxamide formyltransferase activity | | |
| GO:00046 | 2 | polygalacturonase activity | | |
| GO:00046 | 18 | porphobilinogen synthase activity | | |
| GO:00046 | 92 | prenyltransferase activity | | |
| GO:00046 | 25 | prephenate dehydratase activity | | |
| GO:00046 | 21 | prephenate dehydrogenase (NADP+) activity | | |
| GO:00046 | 12 | protein-arginine deiminase activity | | |
| GO:00046 | 11 | protein C-terminal S-isoprenylcysteine carboxyl O-methyltransferase activity | | |
| GO:00046 | 375 | protein kinase activity | | |
| GO:00046 | 88 | protein histidine kinase activity | | |
| GO:00046 | 1 | protein serine/threonine kinase activity | | |

| | | | | |
|---|---|---|---|---|
| GO:00047 | 39 | protein-L-isoaspartate (D-aspartate) O-methyltransferase activity | | |
| GO:00047 | 1 | protein tyrosine phosphatase activity | | |
| GO:00047 | 55 | pyridoxamine-phosphate oxidase activity | | |
| GO:00047 | 42 | pyruvate kinase activity | | |
| GO:00047 | 1 | ribonucleoside-diphosphate reductase activity, thioredoxin disulfide as acceptor | | |
| GO:00047 | 7 | ribose-5-phosphate isomerase activity | | |
| GO:00047 | 37 | shikimate 3-dehydrogenase (NADP+) activity | | |
| GO:00047 | 16 | sulfate adenylyltransferase (ATP) activity | | |
| GO:00047 | 21 | superoxide dismutase activity | | |
| GO:00047 | 21 | thiamine-phosphate diphosphorylase activity | | |
| GO:00047 | 7 | thymidine kinase activity | | |
| GO:00047 | 9 | thymidylate synthase activity | | |
| GO:00048 | 409 | transposase activity | | |
| GO:00048 | 5 | triglyceride lipase activity | | |
| GO:00048 | 17 | triose-phosphate isomerase activity | | |
| GO:00048 | 8 | tRNA adenylyltransferase activity | | |
| GO:00048 | 696 | aminoacyl-tRNA ligase activity | | |
| GO:00048 | 71 | alanine-tRNA ligase activity | | |
| GO:00048 | 65 | arginine-tRNA ligase activity | | |
| GO:00048 | 46 | glycine-tRNA ligase activity | | |
| GO:00048 | 71 | phenylalanine-tRNA ligase activity | | |
| GO:00048 | 12 | proline-tRNA ligase activity | | |
| GO:00048 | 3 | threonine-tRNA ligase activity | | |
| GO:00048 | 11 | tryptophan 2,3-dioxygenase activity | | |
| GO:00048 | 29 | tryptophan synthase activity | | |
| GO:00048 | 2 | ubiquitin-protein transferase activity | | |
| GO:00048 | 22 | uroporphyrinogen-III synthase activity | | |
| GO:00048 | 40 | uroporphyrinogen decarboxylase activity | | |
| GO:00048 | 10 | endopeptidase inhibitor activity | | |
| GO:00048 | 213 | signal transducer activity | | |
| GO:00048 | 213 | receptor activity | | |
| GO:00050 | 1 | GTPase activator activity | | |
| GO:00051 | 45 | structural molecule activity | | |
| GO:00052 | 1 | extracellular matrix structural constituent | | |
| GO:00052 | 1692 | transporter activity | | |
| GO:00052 | 18 | ion channel activity | | |
| GO:00052 | 4 | inward rectifier potassium channel activity | | |
| GO:00052 | 26 | voltage-gated chloride channel activity | | |
| GO:00053 | 31 | inorganic phosphate transmembrane transporter activity | | |
| GO:00053 | 5 | neurotransmitter:sodium symporter activity | | |
| GO:00053 | 13 | iron ion transmembrane transporter activity | | |
| GO:00054 | 1 | monovalent cation:proton antiporter activity | | |
| GO:00054 | 15 | ATP:ADP antiporter activity | | |
| GO:00054 | 37 | binding | | |
| GO:00055 | 381 | iron ion binding | | |
| GO:00055 | 205 | copper ion binding | | |
| GO:00055 | 83 | calcium ion binding | | |
| GO:00055 | 882 | protein binding | | |
| GO:00055 | 6227 | ATP binding | | |
| GO:00055 | 390 | GTP binding | | |
| GO:00055 | 20 | folic acid binding | | |
| GO:00055 | 48 | extracellular region | | |
| GO:00056 | 11 | extracellular space | | |
| GO:00056 | 7 | cell wall | | |
| GO:00056 | 403 | intracellular | | |
| GO:00056 | 2 | nucleus | | |
| GO:00056 | 1 | anaphase-promoting complex | | |
| GO:00056 | 22 | chromosome | | |
| GO:00057 | 44 | extrachromosomal circular DNA | | |
| GO:00057 | 1 | nucleolus | | |
| GO:00057 | 521 | cytoplasm | | |
| GO:00057 | 1 | peroxisome | | |
| GO:00058 | 25 | proteasome core complex | | |
| GO:00058 | 495 | ribosome | | |
| GO:00058 | 175 | plasma membrane | | |
| GO:00058 | 13 | integral component of plasma membrane | | |
| GO:00059 | 1466 | carbohydrate metabolic process | | |
| GO:00059 | 5 | polysaccharide metabolic process | | |
| GO:00059 | 17 | glycogen catabolic process | | |
| GO:00059 | 5 | trehalose metabolic process | | |
| GO:00059 | 57 | trehalose biosynthetic process | | |
| GO:00059 | 2 | monosaccharide metabolic process | | |
| GO:00060 | 14 | fucose metabolic process | | |
| GO:00060 | 50 | glucose metabolic process | | |
| GO:00060 | 13 | glucose catabolic process | | |
| GO:00060 | 1 | UDP-glucose metabolic process | | |
| GO:00060 | 25 | galactose metabolic process | | |
| GO:00060 | 12 | mannose metabolic process | | |
| GO:00060 | 12 | inositol biosynthetic process | | |
| GO:00060 | 1 | chitin catabolic process | | |
| GO:00060 | 23 | amino sugar metabolic process | | |
| GO:00060 | 42 | UDP-N-acetylglucosamine metabolic process | | |
| GO:00060 | 13 | glucuronate catabolic process | | |
| GO:00060 | 34 | glycerol metabolic process | | |
| GO:00060 | 16 | acetyl-CoA metabolic process | | |
| GO:00060 | 114 | gluconeogenesis | | |
| GO:00060 | 231 | glycolytic process | | |
| GO:00060 | 22 | glyoxylate cycle | | |
| GO:00060 | 12 | pentose-phosphate shunt | | |
| GO:00060 | 56 | tricarboxylic acid cycle | | |
| GO:00061 | 5 | fumarate metabolic process | | |
| GO:00061 | 23 | regulation of carbohydrate metabolic process | | |
| GO:00061 | 92 | nucleobase-containing compound metabolic process | | |
| GO:00061 | 94 | purine nucleotide biosynthetic process | | |
| GO:00061 | 15 | nucleoside diphosphate phosphorylation | | |
| GO:00061 | 23 | GMP biosynthetic process | | |
| GO:00061 | 5 | cGMP biosynthetic process | | |
| GO:00061 | 15 | GTP biosynthetic process | | |
| GO:00061 | 1 | IMP biosynthetic process | | |
| GO:00061 | 34 | 'de novo' IMP biosynthetic process | | |
| GO:00061 | 2 | cAMP catabolic process | | |
| GO:00062 | 28 | 'de novo' pyrimidine nucleobase biosynthetic process | | |
| GO:00062 | 8 | pyrimidine nucleoside metabolic process | | |
| GO:00062 | 30 | pyrimidine nucleotide biosynthetic process | | |

| | | | | |
|---|---|---|---|---|
| GO:00062 | 15 | UTP biosynthetic process | | |
| GO:00062 | 30 | dTMP biosynthetic process | | |
| GO:00062 | 15 | CTP biosynthetic process | | |
| GO:00062 | 7 | DNA metabolic process | | |
| GO:00062 | 486 | DNA replication | | |
| GO:00062 | 234 | DNA topological change | | |
| GO:00062 | 58 | DNA replication, synthesis of RNA primer | | |
| GO:00062 | 1 | DNA replication initiation | | |
| GO:00062 | 1 | plasmid maintenance | | |
| GO:00062 | 371 | DNA repair | | |
| GO:00062 | 9 | regulation of DNA repair | | |
| GO:00062 | 121 | base-excision repair | | |
| GO:00062 | 23 | nucleotide-excision repair | | |
| GO:00062 | 155 | mismatch repair | | |
| GO:00063 | 36 | double-strand break repair via nonhomologous end joining | | |
| GO:00063 | 41 | DNA modification | | |
| GO:00063 | 100 | DNA methylation | | |
| GO:00063 | 11 | DNA catabolic process | | |
| GO:00063 | 360 | DNA recombination | | |
| GO:00063 | 409 | transposition, DNA-mediated | | |
| GO:00063 | 2 | DNA packaging | | |
| GO:00063 | 190 | transcription, DNA-templated | | |
| GO:00063 | 511 | DNA-templated transcription, initiation | | |
| GO:00063 | 6 | DNA-templated transcription, termination | | |
| GO:00063 | 1551 | regulation of transcription, DNA-templated | | |
| GO:00063 | 1 | regulation of transcription from RNA polymerase II promoter | | |
| GO:00063 | 96 | rRNA processing | | |
| GO:00063 | 1 | tRNA splicing, via endonucleolytic cleavage and ligation | | |
| GO:00063 | 284 | RNA processing | | |
| GO:00063 | 1 | mRNA processing | | |
| GO:00064 | 32 | tRNA modification | | |
| GO:00064 | 1 | RNA catabolic process | | |
| GO:00064 | 6 | mRNA catabolic process | | |
| GO:00064 | 488 | translation | | |
| GO:00064 | 40 | translational initiation | | |
| GO:00064 | 34 | translational elongation | | |
| GO:00064 | 48 | translational termination | | |
| GO:00064 | 678 | tRNA aminoacylation for protein translation | | |
| GO:00064 | 71 | alanyl-tRNA aminoacylation | | |
| GO:00064 | 65 | arginyl-tRNA aminoacylation | | |
| GO:00064 | 46 | glycyl-tRNA aminoacylation | | |
| GO:00064 | 1 | phenylalanyl-tRNA aminoacylation | | |
| GO:00064 | 12 | prolyl-tRNA aminoacylation | | |
| GO:00064 | 7 | regulation of translational fidelity | | |
| GO:00064 | 152 | protein folding | | |
| GO:00064 | 2 | protein complex assembly | | |
| GO:00064 | 103 | cellular protein modification process | | |
| GO:00064 | 358 | protein phosphorylation | | |
| GO:00064 | 3 | protein dephosphorylation | | |
| GO:00064 | 11 | C-terminal protein methylation | | |
| GO:00064 | 2 | protein glycosylation | | |
| GO:00064 | 29 | protein O-linked glycosylation | | |
| GO:00065 | 1 | GPI anchor metabolic process | | |
| GO:00065 | 1 | GPI anchor biosynthetic process | | |
| GO:00065 | 1512 | proteolysis | | |
| GO:00065 | 1 | ubiquitin-dependent protein catabolic process | | |
| GO:00065 | 245 | cellular amino acid metabolic process | | |
| GO:00065 | 6 | alanine biosynthetic process | | |
| GO:00065 | 8 | arginine metabolic process | | |
| GO:00065 | 61 | arginine biosynthetic process | | |
| GO:00065 | 8 | arginine catabolic process | | |
| GO:00065 | 158 | asparagine biosynthetic process | | |
| GO:00065 | 3 | cysteine biosynthetic process from serine | | |
| GO:00065 | 56 | glutamate biosynthetic process | | |
| GO:00065 | 25 | glutamine metabolic process | | |
| GO:00065 | 12 | glycine biosynthetic process | | |
| GO:00065 | 63 | glycine catabolic process | | |
| GO:00065 | 53 | methionine metabolic process | | |
| GO:00065 | 19 | S-adenosylmethionine biosynthetic process | | |
| GO:00065 | 27 | L-phenylalanine catabolic process | | |
| GO:00065 | 50 | tryptophan metabolic process | | |
| GO:00065 | 27 | tyrosine metabolic process | | |
| GO:00065 | 21 | tyrosine biosynthetic process | | |
| GO:00066 | 4 | protein targeting | | |
| GO:00066 | 64 | SRP-dependent cotranslational protein targeting to membrane | | |
| GO:00066 | 205 | lipid metabolic process | | |
| GO:00066 | 103 | fatty acid metabolic process | | |
| GO:00066 | 97 | fatty acid biosynthetic process | | |
| GO:00066 | 1 | fatty acid beta-oxidation | | |
| GO:00066 | 4 | sphingolipid metabolic process | | |
| GO:00066 | 9 | steroid biosynthetic process | | |
| GO:00067 | 72 | cellular aromatic compound metabolic process | | |
| GO:00067 | 19 | tetrahydrobiopterin biosynthetic process | | |
| GO:00067 | 40 | one-carbon metabolic process | | |
| GO:00067 | 35 | NADP biosynthetic process | | |
| GO:00067 | 7 | NADP catabolic process | | |
| GO:00067 | 92 | ubiquinone biosynthetic process | | |
| GO:00067 | 136 | glutathione metabolic process | | |
| GO:00067 | 27 | glutathione biosynthetic process | | |
| GO:00067 | 13 | folic acid-containing compound metabolic process | | |
| GO:00067 | 61 | Mo-molybdopterin cofactor biosynthetic process | | |
| GO:00067 | 56 | porphyrin-containing compound biosynthetic process | | |
| GO:00067 | 32 | heme biosynthetic process | | |
| GO:00067 | 27 | heme a biosynthetic process | | |
| GO:00067 | 20 | phosphate-containing compound metabolic process | | |
| GO:00068 | 24 | superoxide metabolic process | | |
| GO:00068 | 328 | nitrogen compound metabolic process | | |
| GO:00068 | 14 | regulation of nitrogen utilization | | |
| GO:00068 | 2 | nitric oxide biosynthetic process | | |
| GO:00068 | 3644 | transport | | |
| GO:00068 | 3 | ion transport | | |
| GO:00068 | 248 | cation transport | | |
| GO:00068 | 112 | potassium ion transport | | |
| GO:00068 | 42 | sodium ion transport | | |

| | | | | |
|---|---|---|---|---|
| GO:00068 | 31 | phosphate ion transport | | |
| GO:00068 | 26 | chloride transport | | |
| GO:00068 | 13 | high-affinity iron ion transmembrane transport | | |
| GO:00068 | 31 | dicarboxylic acid transport | | |
| GO:00068 | 5 | neurotransmitter transport | | |
| GO:00068 | 48 | drug transmembrane transport | | |
| GO:00068 | 22 | amino acid transport | | |
| GO:00068 | 5 | lipid transport | | |
| GO:00068 | 1 | cellular copper ion homeostasis | | |
| GO:00068 | 36 | cellular iron ion homeostasis | | |
| GO:00068 | 15 | regulation of pH | | |
| GO:00068 | 4 | intracellular protein transport | | |
| GO:00069 | 18 | movement of cell or subcellular component | | |
| GO:00069 | 141 | chemotaxis | | |
| GO:00069 | 154 | response to stress | | |
| GO:00069 | 78 | response to oxidative stress | | |
| GO:00070 | 1 | mitochondrion organization | | |
| GO:00070 | 64 | cell cycle | | |
| GO:00070 | 60 | chromosome segregation | | |
| GO:00071 | 28 | cell communication | | |
| GO:00071 | 19 | cell adhesion | | |
| GO:00071 | 3 | homophilic cell adhesion via plasma membrane adhesion molecules | | |
| GO:00071 | 199 | signal transduction | | |
| GO:00071 | 1 | G-protein coupled receptor signaling pathway | | |
| GO:00072 | 3 | Notch signaling pathway | | |
| GO:00072 | 1 | small GTPase mediated signal transduction | | |
| GO:00076 | 1 | circadian rhythm | | |
| GO:00080 | 1 | beta-catenin binding | | |
| GO:00080 | 56 | ATP-dependent helicase activity | | |
| GO:00080 | 193 | tRNA processing | | |
| GO:00080 | 14 | enzyme activator activity | | |
| GO:00080 | 285 | N-acetyltransferase activity | | |
| GO:00080 | 36 | phosphoric diester hydrolase activity | | |
| GO:00080 | 10 | 5S rRNA binding | | |
| GO:00081 | 1 | galactoside 2-alpha-L-fucosyltransferase activity | | |
| GO:00081 | 25 | UDP-glucose:hexose-1-phosphate uridylyltransferase activity | | |
| GO:00081 | 33 | peptide-methionine (S)-S-oxide reductase activity | | |
| GO:00081 | 2 | sarcosine oxidase activity | | |
| GO:00081 | 19 | 4-alpha-hydroxytetrahydrobiopterin dehydratase activity | | |
| GO:00081 | 10 | primary amine oxidase activity | | |
| GO:00081 | 400 | transcription factor binding | | |
| GO:00081 | 62 | NADH dehydrogenase (ubiquinone) activity | | |
| GO:00081 | 2 | protein tyrosine/serine/threonine phosphatase activity | | |
| GO:00081 | 7 | sulfotransferase activity | | |
| GO:00081 | 4852 | metabolic process | | |
| GO:00081 | 660 | methyltransferase activity | | |
| GO:00081 | 100 | N-methyltransferase activity | | |
| GO:00081 | 39 | O-methyltransferase activity | | |
| GO:00081 | 136 | RNA methyltransferase activity | | |
| GO:00081 | 7 | tRNA (guanine-N7-)-methyltransferase activity | | |
| GO:00081 | 42 | glycogen phosphorylase activity | | |
| GO:00081 | 19 | ferrous iron binding | | |
| GO:00081 | 65 | ferric iron binding | | |
| GO:00082 | 117 | peptidase activity | | |
| GO:00082 | 10 | cysteine-type peptidase activity | | |
| GO:00082 | 309 | serine-type peptidase activity | | |
| GO:00082 | 45 | metallopeptidase activity | | |
| GO:00082 | 7 | dipeptidyl-peptidase activity | | |
| GO:00082 | 3 | 5'-nucleotidase activity | | |
| GO:00082 | 716 | zinc ion binding | | |
| GO:00082 | 53 | sulfate transport | | |
| GO:00082 | 5 | lipid binding | | |
| GO:00082 | 3 | spermidine biosynthetic process | | |
| GO:00082 | 67 | isoprenoid biosynthetic process | | |
| GO:00083 | 147 | cation transmembrane transporter activity | | |
| GO:00083 | 26 | regulation of cell shape | | |
| GO:00084 | 260 | 3'-5' exonuclease activity | | |
| GO:00084 | 66 | CoA-transferase activity | | |
| GO:00084 | 2 | fucosyltransferase activity | | |
| GO:00084 | 11 | selenium binding | | |
| GO:00084 | 48 | RNA ligase activity | | |
| GO:00084 | 25 | queuine tRNA-ribosyltransferase activity | | |
| GO:00084 | 265 | transaminase activity | | |
| GO:00084 | 135 | sulfuric ester hydrolase activity | | |
| GO:00084 | 3 | gamma-glutamyl carboxylase activity | | |
| GO:00085 | 59 | ammonium transmembrane transporter activity | | |
| GO:00085 | 5 | acetyl-CoA transporter activity | | |
| GO:00085 | 1 | riboflavin kinase activity | | |
| GO:00085 | 28 | potassium-transporting ATPase activity | | |
| GO:00085 | 53 | protein transporter activity | | |
| GO:00086 | 121 | lipid biosynthetic process | | |
| GO:00086 | 18 | peptidyl-lysine modification to peptidyl-hypusine | | |
| GO:00086 | 50 | pyridoxine biosynthetic process | | |
| GO:00086 | 70 | queuosine biosynthetic process | | |
| GO:00086 | 42 | small protein activating enzyme activity | | |
| GO:00086 | 3 | hexose transport | | |
| GO:00086 | 25 | rRNA methyltransferase activity | | |
| GO:00086 | 56 | cellular amino acid biosynthetic process | | |
| GO:00086 | 125 | phospholipid biosynthetic process | | |
| GO:00086 | 1 | DNA topoisomerase (ATP-hydrolyzing) inhibitor activity | | |
| GO:00086 | 174 | penicillin binding | | |
| GO:00086 | 40 | 1-deoxy-D-xylulose-5-phosphate synthase activity | | |
| GO:00086 | 1 | 2-dehydro-3-deoxygalactonokinase activity | | |
| GO:00086 | 30 | 2-dehydropantoate 2-reductase activity | | |
| GO:00086 | 18 | 2-C-methyl-D-erythritol 2,4-cyclodiphosphate synthase activity | | |
| GO:00086 | 17 | 3,4-dihydroxy-2-butanone-4-phosphate synthase activity | | |
| GO:00087 | 94 | 5-amino-6-(5-phosphoribosylamino)uracil reductase activity | | |
| GO:00087 | 25 | methionine synthase activity | | |
| GO:00087 | 64 | D-alanine-D-alanine ligase activity | | |
| GO:00087 | 10 | DNA-3-methyladenine glycosylase activity | | |
| GO:00087 | 3 | L-arabinose isomerase activity | | |
| GO:00087 | 14 | L-fucose isomerase activity | | |
| GO:00087 | 9 | L-rhamnose isomerase activity | | |
| GO:00087 | 56 | N-acetylmuramoyl-L-alanine amidase activity | | |

| | | | | |
|---|---|---|---|---|
| GO:00087 | 18 | NAD(P)+ transhydrogenase (AB-specific) activity | | |
| GO:00087 | 6 | S-adenosylmethionine-dependent methyltransferase activity | | |
| GO:00087 | 25 | UDP-3-O-[3-hydroxymyristoyl] N-acetylglucosamine deacetylase activity | | |
| GO:00087 | 143 | UDP-N-acetylmuramate dehydrogenase activity | | |
| GO:00087 | 11 | UDP-galactopyranose mutase activity | | |
| GO:00087 | 1 | [acyl-carrier-protein] phosphodiesterase activity | | |
| GO:00087 | 11 | [isocitrate dehydrogenase (NADP+)] kinase activity | | |
| GO:00087 | 19 | [protein-PII] uridylyltransferase activity | | |
| GO:00087 | 3 | acetaldehyde dehydrogenase (acetylating) activity | | |
| GO:00087 | 3 | arginine N-succinyltransferase activity | | |
| GO:00087 | 5 | arginine decarboxylase activity | | |
| GO:00088 | 4 | cellulase activity | | |
| GO:00088 | 3 | chloramphenicol O-acetyltransferase activity | | |
| GO:00088 | 1 | citrate CoA-transferase activity | | |
| GO:00088 | 8 | cob(I)yrinic acid a,c-diamide adenosyltransferase activity | | |
| GO:00088 | 3 | cytochrome o ubiquinol oxidase activity | | |
| GO:00088 | 13 | dCTP deaminase activity | | |
| GO:00088 | 33 | dTDP-4-dehydrorhamnose 3,5-epimerase activity | | |
| GO:00088 | 26 | diaminopimelate epimerase activity | | |
| GO:00088 | 22 | 4-hydroxy-tetrahydrodipicolinate reductase | | |
| GO:00088 | 5 | ethanolamine ammonia-lyase activity | | |
| GO:00088 | 49 | exodeoxyribonuclease VII activity | | |
| GO:00088 | 6 | glucuronate isomerase activity | | |
| GO:00088 | 55 | [glutamate-ammonia-ligase] adenylyltransferase activity | | |
| GO:00088 | 42 | glutamyl-tRNA reductase activity | | |
| GO:00088 | 12 | glycerate kinase activity | | |
| GO:00088 | 19 | holo-[acyl-carrier-protein] synthase activity | | |
| GO:00088 | 53 | S-adenosylmethionine-homocysteine S-methyltransferase activity | | |
| GO:00088 | 1 | homoserine O-succinyltransferase activity | | |
| GO:00089 | 12 | leucyltransferase activity | | |
| GO:00089 | 28 | lipid-A-disaccharide synthase activity | | |
| GO:00089 | 7 | mannonate dehydratase activity | | |
| GO:00089 | 5 | nicotinate-nucleotide-dimethylbenzimidazole phosphoribosyltransferase activity | | |
| GO:00089 | 8 | phosphatidylglycerophosphatase activity | | |
| GO:00089 | 56 | phospho-N-acetylmuramoyl-pentapeptide-transferase activity | | |
| GO:00089 | 39 | phosphoenolpyruvate carboxylase activity | | |
| GO:00089 | 21 | prephenate dehydrogenase activity | | |
| GO:00089 | 10 | protein-N(PI)-phosphohistidine-sugar phosphotransferase activity | | |
| GO:00089 | 63 | protein-glutamate methylesterase activity | | |
| GO:00089 | 23 | quinolinate synthetase A activity | | |
| GO:00090 | 3 | serine O-acetyltransferase activity | | |
| GO:00090 | 36 | serine-type D-Ala-D-Ala carboxypeptidase activity | | |
| GO:00090 | 5 | site-specific DNA-methyltransferase (adenine-specific) activity | | |
| GO:00090 | 1 | N-succinylarginine dihydrolase activity | | |
| GO:00090 | 11 | tetraacyldisaccharide 4'-kinase activity | | |
| GO:00090 | 3 | Type II site-specific deoxyribonuclease activity | | |
| GO:00090 | 1 | xylan 1,4-beta-xylosidase activity | | |
| GO:00090 | 7 | pentose-phosphate shunt, non-oxidative branch | | |
| GO:00090 | 538 | electron carrier activity | | |
| GO:00090 | 10 | catabolic process | | |
| GO:00090 | 1671 | biosynthetic process | | |
| GO:00090 | 119 | aerobic respiration | | |
| GO:00090 | 49 | aromatic amino acid family biosynthetic process | | |
| GO:00090 | 2 | branched-chain amino acid biosynthetic process | | |
| GO:00090 | 99 | methionine biosynthetic process | | |
| GO:00090 | 48 | lysine biosynthetic process via diaminopimelate | | |
| GO:00090 | 25 | L-phenylalanine biosynthetic process | | |
| GO:00091 | 22 | lipopolysaccharide biosynthetic process | | |
| GO:00091 | 188 | nucleoside metabolic process | | |
| GO:00091 | 3 | nucleotide metabolic process | | |
| GO:00091 | 12 | nucleotide biosynthetic process | | |
| GO:00091 | 41 | nucleotide catabolic process | | |
| GO:00091 | 14 | deoxyribonucleoside diphosphate metabolic process | | |
| GO:00091 | 179 | cyclic nucleotide biosynthetic process | | |
| GO:00092 | 33 | thiamine biosynthetic process | | |
| GO:00092 | 176 | riboflavin biosynthetic process | | |
| GO:00092 | 75 | cobalamin biosynthetic process | | |
| GO:00092 | 64 | lipid A biosynthetic process | | |
| GO:00092 | 9 | glycolipid biosynthetic process | | |
| GO:00092 | 26 | protein lipoylation | | |
| GO:00092 | 5 | peptidoglycan biosynthetic process | | |
| GO:00092 | 61 | peptidoglycan catabolic process | | |
| GO:00092 | 24 | peptidoglycan turnover | | |
| GO:00092 | 25 | cellular response to starvation | | |
| GO:00092 | 7 | Gram-negative-bacterium-type cell wall | | |
| GO:00092 | 19 | cell outer membrane | | |
| GO:00092 | 15 | bacterial-type flagellum | | |
| GO:00092 | 7 | pilus | | |
| GO:00092 | 4 | unidirectional conjugation | | |
| GO:00092 | 37 | DNA mediated transformation | | |
| GO:00092 | 2 | nucleoid | | |
| GO:00093 | 161 | protein secretion | | |
| GO:00093 | 5 | DNA restriction-modification system | | |
| GO:00093 | 10 | amine metabolic process | | |
| GO:00093 | 19 | acetyl-CoA carboxylase complex | | |
| GO:00093 | 5 | exodeoxyribonuclease VII complex | | |
| GO:00093 | 13 | beta-galactosidase complex | | |
| GO:00093 | 1 | citrate lyase complex | | |
| GO:00093 | 15 | riboflavin synthase complex | | |
| GO:00093 | 59 | DNA polymerase III complex | | |
| GO:00093 | 52 | four-way junction helicase activity | | |
| GO:00093 | 13 | 2'-deoxyribonucleotide metabolic process | | |
| GO:00093 | 72 | folic acid-containing compound biosynthetic process | | |
| GO:00094 | 36 | phosphoenolpyruvate-dependent sugar phosphotransferase system | | |
| GO:00094 | 2 | toxin biosynthetic process | | |
| GO:00094 | 2 | pathogenesis | | |
| GO:00094 | 1 | bacterial-type flagellum hook | | |
| GO:00094 | 11 | bacterial-type flagellum basal body | | |
| GO:00094 | 2 | bacterial-type flagellum basal body, distal rod, L ring | | |
| GO:00094 | 4 | bacterial-type flagellum basal body, distal rod, P ring | | |
| GO:00094 | 38 | SOS response | | |
| GO:00094 | 58 | NAD biosynthetic process | | |
| GO:00094 | 1 | cyanate metabolic process | | |
| GO:00094 | 12 | putrescine biosynthetic process | | |

| | | | | |
|---|---|---|---|---|
| GO:00094 | 140 | RNA modification | | |
| GO:00094 | 6 | plastoquinol--plastocyanin reductase activity | | |
| GO:00095 | 11 | detection of visible light | | |
| GO:00096 | 28 | hydrogen-translocating pyrophosphatase activity | | |
| GO:00097 | 2 | photosynthetic electron transport in photosystem II | | |
| GO:00099 | 2 | cytidine deamination | | |
| GO:00099 | 125 | pseudouridine synthase activity | | |
| GO:00100 | 27 | response to organic substance | | |
| GO:00100 | 10 | response to metal ion | | |
| GO:00101 | 178 | FMN binding | | |
| GO:00102 | 1 | chlorophyllide a oxygenase [overall] activity | | |
| GO:00103 | 1 | acireductone dioxygenase [iron(II)-requiring] activity | | |
| GO:00104 | 8 | regulation of gene expression | | |
| GO:00104 | 17 | proteasomal protein catabolic process | | |
| GO:00125 | 6 | vesicle membrane | | |
| GO:00150 | 37 | heme-copper terminal oxidase activity | | |
| GO:00150 | 2 | [heparan sulfate]-glucosamine N-sulfotransferase activity | | |
| GO:00150 | 172 | protein transport | | |
| GO:00150 | 74 | protein disulfide oxidoreductase activity | | |
| GO:00150 | 384 | DNA integration | | |
| GO:00150 | 1 | ion transmembrane transporter activity | | |
| GO:00150 | 70 | hydrogen ion transmembrane transporter activity | | |
| GO:00150 | 37 | potassium ion transmembrane transporter activity | | |
| GO:00150 | 26 | ferrous iron transmembrane transporter activity | | |
| GO:00150 | 15 | nickel cation transmembrane transporter activity | | |
| GO:00151 | 4 | arsenite transmembrane transporter activity | | |
| GO:00151 | 47 | chromate transmembrane transporter activity | | |
| GO:00151 | 2 | phosphate ion transmembrane transporter activity | | |
| GO:00151 | 53 | sulfate transmembrane transporter activity | | |
| GO:00151 | 5 | gluconate transmembrane transporter activity | | |
| GO:00151 | 13 | lactate transmembrane transporter activity | | |
| GO:00151 | 3 | rhamnose transmembrane transporter activity | | |
| GO:00151 | 37 | polysaccharide transmembrane transporter activity | | |
| GO:00151 | 240 | amino acid transmembrane transporter activity | | |
| GO:00152 | 5 | biotin transporter activity | | |
| GO:00152 | 15 | heme transporter activity | | |
| GO:00152 | 48 | drug transmembrane transporter activity | | |
| GO:00152 | 2 | porin activity | | |
| GO:00152 | 48 | antiporter activity | | |
| GO:00152 | 107 | solute:proton antiporter activity | | |
| GO:00153 | 10 | sodium-dependent phosphate transmembrane transporter activity | | |
| GO:00154 | 2 | P-P-bond-hydrolysis-driven protein transmembrane transporter activity | | |
| GO:00154 | 1 | cholesterol binding | | |
| GO:00155 | 4 | glutamate:sodium symporter activity | | |
| GO:00155 | 4 | C4-dicarboxylate transmembrane transporter activity | | |
| GO:00155 | 2 | p-aminobenzoyl-glutamate uptake transmembrane transporter activity | | |
| GO:00156 | 9 | type II protein secretion system complex | | |
| GO:00156 | 9 | protein secretion by the type II secretion system | | |
| GO:00156 | 1 | monovalent inorganic cation transport | | |
| GO:00156 | 26 | ferrous iron transport | | |
| GO:00156 | 59 | ammonium transport | | |
| GO:00157 | 4 | arsenite transport | | |
| GO:00157 | 47 | chromate transport | | |
| GO:00157 | 13 | lactate transport | | |
| GO:00157 | 4 | C4-dicarboxylate transport | | |
| GO:00157 | 45 | polysaccharide transport | | |
| GO:00158 | 4 | L-glutamate transport | | |
| GO:00158 | 5 | biotin transport | | |
| GO:00158 | 15 | heme transport | | |
| GO:00159 | 12 | mannosidase activity | | |
| GO:00159 | 3 | glucosidase activity | | |
| GO:00159 | 56 | glutamate synthase activity | | |
| GO:00159 | 2 | large ribosomal subunit | | |
| GO:00159 | 17 | coenzyme A metabolic process | | |
| GO:00159 | 19 | coenzyme A biosynthetic process | | |
| GO:00159 | 47 | pantothenate biosynthetic process | | |
| GO:00159 | 35 | methanogenesis | | |
| GO:00159 | 1 | guanosine tetraphosphate metabolic process | | |
| GO:00159 | 39 | carbon fixation | | |
| GO:00159 | 87 | photosynthesis | | |
| GO:00159 | 119 | ATP synthesis coupled proton transport | | |
| GO:00159 | 13 | ATP hydrolysis coupled proton transport | | |
| GO:00159 | 29 | proton transport | | |
| GO:00159 | 87 | chlorophyll biosynthetic process | | |
| GO:00160 | 5394 | membrane | | |
| GO:00160 | 2979 | integral component of membrane | | |
| GO:00160 | 6 | lipid catabolic process | | |
| GO:00160 | 18 | carbohydrate biosynthetic process | | |
| GO:00160 | 1 | Wnt signaling pathway | | |
| GO:00161 | 82 | terpenoid biosynthetic process | | |
| GO:00161 | 13 | carotenoid biosynthetic process | | |
| GO:00161 | 8 | translation release factor activity, codon specific | | |
| GO:00161 | 98 | nickel cation binding | | |
| GO:00161 | 17 | urocanate hydratase activity | | |
| GO:00162 | 113 | antioxidant activity | | |
| GO:00162 | 71 | iron-sulfur cluster assembly | | |
| GO:00163 | 183 | kinase activity | | |
| GO:00163 | 195 | phosphorylation | | |
| GO:00163 | 36 | dephosphorylation | | |
| GO:00164 | 16 | acetyltransferase activity | | |
| GO:00164 | 6 | tRNA (adenine-N1-)-methyltransferase activity | | |
| GO:00164 | 1 | tRNA cytidylyltransferase activity | | |
| GO:00164 | 1 | myosin complex | | |
| GO:00164 | 3 | pyrophosphatase activity | | |
| GO:00164 | 5017 | oxidoreductase activity | | |
| GO:00165 | 1 | mediator complex | | |
| GO:00165 | 49 | amino acid binding | | |
| GO:00165 | 7 | protein arginylation | | |
| GO:00166 | 3 | nuclear speck | | |
| GO:00166 | 176 | oxidoreductase activity, acting on CH-OH group of donors | | |
| GO:00166 | 213 | oxidoreductase activity, acting on the CH-OH group of donors, NAD or NADP as acceptor | | |
| GO:00166 | 108 | oxidoreductase activity, acting on the aldehyde or oxo group of donors, NAD or NADP as acceptor | | |
| GO:00166 | 105 | oxidoreductase activity, acting on the aldehyde or oxo group of donors, disulfide as acceptor | | |
| GO:00166 | 69 | oxidoreductase activity, acting on the aldehyde or oxo group of donors, iron-sulfur protein as acceptor | | |

| | | | | |
|---|---|---|---|---|
| GO:00166 | 516 | oxidoreductase activity, acting on the CH-CH group of donors | | |
| GO:00166 | 56 | oxidoreductase activity, acting on the CH-NH2 group of donors | | |
| GO:00166 | 3 | oxidoreductase activity, acting on the CH-NH group of donors | | |
| GO:00166 | 94 | oxidoreductase activity, acting on NAD(P)H | | |
| GO:00166 | 2 | oxidoreductase activity, acting on NAD(P)H, quinone or similar compound as acceptor | | |
| GO:00167 | 407 | oxidoreductase activity, acting on paired donors, with incorporation or reduction of molecular oxygen | | |
| GO:00167 | 6 | oxidoreductase activity, acting on paired donors, with incorporation or reduction of molecular oxygen, 2-oxoglutarate as one donor, and incorporation of one atom each of oxygen into l | | |
| GO:00167 | 48 | oxidoreductase activity, acting on paired donors, with incorporation or reduction of molecular oxygen, NAD(P)H as one donor, and incorporation of two atoms of oxygen into one donc | | |
| GO:00167 | 17 | oxidoreductase activity, acting on paired donors, with incorporation or reduction of molecular oxygen, reduced pteridine as one donor, and incorporation of one atom of oxygen | | |
| GO:00167 | 2 | oxidoreductase activity, acting on iron-sulfur proteins as donors | | |
| GO:00167 | 328 | transferase activity | | |
| GO:00167 | 83 | hydroxymethyl-, formyl- and related transferase activity | | |
| GO:00167 | 78 | carboxyl- or carbamoyltransferase activity | | |
| GO:00167 | 315 | transferase activity, transferring acyl groups | | |
| GO:00167 | 347 | transferase activity, transferring acyl groups other than amino-acyl groups | | |
| GO:00167 | 5 | transferase activity, transferring amino-acyl groups | | |
| GO:00167 | 1 | glutathione gamma-glutamylcysteinyltransferase activity | | |
| GO:00167 | 139 | transferase activity, transferring glycosyl groups | | |
| GO:00167 | 68 | transferase activity, transferring hexosyl groups | | |
| GO:00167 | 16 | transferase activity, transferring pentosyl groups | | |
| GO:00167 | 133 | transferase activity, transferring alkyl or aryl (other than methyl) groups | | |
| GO:00167 | 104 | transferase activity, transferring phosphorus-containing groups | | |
| GO:00167 | 120 | phosphotransferase activity, alcohol group as acceptor | | |
| GO:00167 | 29 | phosphotransferase activity, carboxyl group as acceptor | | |
| GO:00167 | 302 | nucleotidyltransferase activity | | |
| GO:00167 | 73 | phosphotransferase activity, for other substituted phosphate groups | | |
| GO:00167 | 16 | sulfurtransferase activity | | |
| GO:00167 | 1690 | hydrolase activity | | |
| GO:00167 | 101 | hydrolase activity, acting on ester bonds | | |
| GO:00167 | 2 | thiolester hydrolase activity | | |
| GO:00167 | 22 | phosphatase activity | | |
| GO:00167 | 21 | hydrolase activity, acting on glycosyl bonds | | |
| GO:00167 | 64 | hydrolase activity, hydrolyzing N-glycosyl compounds | | |
| GO:00168 | 54 | dipeptidase activity | | |
| GO:00168 | 238 | hydrolase activity, acting on carbon-nitrogen (but not peptide) bonds | | |
| GO:00168 | 37 | hydrolase activity, acting on carbon-nitrogen (but not peptide) bonds, in linear amides | | |
| GO:00168 | 29 | hydrolase activity, acting on carbon-nitrogen (but not peptide) bonds, in linear amidines | | |
| GO:00168 | 3 | hydrolase activity, acting on acid anhydrides | | |
| GO:00168 | 146 | hydrolase activity, acting on acid anhydrides, in phosphorus-containing anhydrides | | |
| GO:00168 | 5 | hydrolase activity, acting on acid anhydrides, catalyzing transmembrane movement of substances | | |
| GO:00168 | 185 | lyase activity | | |
| GO:00168 | 172 | carboxy-lyase activity | | |
| GO:00168 | 8 | aldehyde-lyase activity | | |
| GO:00168 | 43 | hydro-lyase activity | | |
| GO:00168 | 1 | carbon-nitrogen lyase activity | | |
| GO:00168 | 32 | ammonia-lyase activity | | |
| GO:00168 | 10 | strictosidine synthase activity | | |
| GO:00168 | 35 | carbon-sulfur lyase activity | | |
| GO:00168 | 179 | phosphorus-oxygen lyase activity | | |
| GO:00168 | 75 | magnesium chelatase activity | | |
| GO:00168 | 6 | sirohydrochlorin cobaltochelatase activity | | |
| GO:00168 | 120 | isomerase activity | | |
| GO:00168 | 20 | racemase and epimerase activity, acting on carbohydrates and derivatives | | |
| GO:00168 | 2 | intramolecular oxidoreductase activity, interconverting aldoses and ketoses | | |
| GO:00168 | 73 | intramolecular transferase activity | | |
| GO:00168 | 121 | intramolecular transferase activity, phosphotransferases | | |
| GO:00168 | 7 | intramolecular lyase activity | | |
| GO:00168 | 53 | ligase activity | | |
| GO:00168 | 111 | ligase activity, forming aminoacyl-tRNA and related compounds | | |
| GO:00168 | 181 | carbon-nitrogen ligase activity, with glutamine as amido-N-donor | | |
| GO:00168 | 1400 | ATPase activity | | |
| GO:00168 | 32 | endodeoxyribonuclease activity, producing 5'-phosphomonoesters | | |
| GO:00169 | 67 | oxidoreductase activity, acting on the CH-OH group of donors, quinone or similar compound as acceptor | | |
| GO:00169 | 77 | oxidoreductase activity, acting on the aldehyde or oxo group of donors | | |
| GO:00169 | 489 | sigma factor activity | | |
| GO:00169 | 1 | sigma factor antagonist activity | | |
| GO:00169 | 4 | precorrin-8X methylmutase activity | | |
| GO:00169 | 2 | precorrin-6A reductase activity | | |
| GO:00169 | 6 | cell wall macromolecule catabolic process | | |
| GO:00170 | 78 | antibiotic biosynthetic process | | |
| GO:00170 | 10 | protein-heme linkage | | |
| GO:00170 | 141 | cytochrome complex assembly | | |
| GO:00170 | 3 | protein-phycocyanobilin linkage | | |
| GO:00170 | 32 | protein flavinylation | | |
| GO:00170 | 73 | protein import | | |
| GO:00171 | 37 | tRNA dihydrouridine synthase activity | | |
| GO:00171 | 31 | sodium:dicarboxylate symporter activity | | |
| GO:00171 | 9 | cysteine dioxygenase activity | | |
| GO:00171 | 3 | peptidyl-glutamic acid carboxylation | | |
| GO:00180 | 1 | histone-lysine N-methyltransferase activity | | |
| GO:00181 | 12 | proline racemase activity | | |
| GO:00181 | 4 | peptidyl-amino acid modification | | |
| GO:00182 | 11 | protein-chromophore linkage | | |
| GO:00184 | 7 | carbon-monoxide dehydrogenase (acceptor) activity | | |
| GO:00185 | 67 | nitronate monooxygenase activity | | |
| GO:00187 | 3 | methenyltetrahydromethanopterin cyclohydrolase activity | | |
| GO:00188 | 10 | nitrile hydratase activity | | |
| GO:00188 | 3 | alkylmercury lyase activity | | |
| GO:00190 | 1 | guanyl nucleotide binding | | |
| GO:00190 | 26 | molybdopterin synthase complex | | |
| GO:00190 | 2 | viral capsid | | |
| GO:00190 | 1 | viral envelope | | |
| GO:00190 | 3 | viral life cycle | | |
| GO:00190 | 6 | virion assembly | | |
| GO:00190 | 2 | transformation of host cell by virus | | |
| GO:00192 | 34 | deaminase activity | | |
| GO:00192 | 1 | L-methionine biosynthetic process from homoserine via O-succinyl-L-homoserine and cystathionine | | |
| GO:00192 | 29 | isopentenyl diphosphate biosynthetic process, methylerythritol 4-phosphate pathway | | |
| GO:00192 | 6 | coenzyme M biosynthetic process | | |
| GO:00192 | 10 | rhamnose metabolic process | | |
| GO:00193 | 9 | siroheme biosynthetic process | | |
| GO:00193 | 7 | pyridine nucleotide biosynthetic process | | |
| GO:00193 | 1 | methanogenesis, from acetate | | |
| GO:00194 | 3 | galactitol metabolic process | | |

| | | | | |
|---|---|---|---|---|
| GO:00194 | 51 | aromatic compound catabolic process | | |
| GO:00194 | 35 | tryptophan catabolic process to kynurenine | | |
| GO:00194 | 24 | L-lysine catabolic process to acetate | | |
| GO:00194 | 17 | D-amino acid catabolic process | | |
| GO:00194 | 1 | ectoine biosynthetic process | | |
| GO:00195 | 84 | propionate catabolic process | | |
| GO:00195 | 37 | glutamate catabolic process to 2-oxoglutarate | | |
| GO:00196 | 8 | butyrate metabolic process | | |
| GO:00196 | 2 | urea metabolic process | | |
| GO:00196 | 3 | organic phosphonate metabolic process | | |
| GO:00196 | 1 | anaerobic electron transport chain | | |
| GO:00196 | 4 | anaerobic glutamate catabolic process | | |
| GO:00196 | 2 | photosynthesis, light reaction | | |
| GO:00197 | 1 | organic phosphonate catabolic process | | |
| GO:00197 | 95 | carboxylic acid metabolic process | | |
| GO:00198 | 12 | oxygen binding | | |
| GO:00198 | 61 | rRNA binding | | |
| GO:00198 | 139 | outer membrane | | |
| GO:00199 | 17 | modification-dependent protein catabolic process | | |
| GO:00200 | 692 | heme binding | | |
| GO:00228 | 67 | transmembrane transporter activity | | |
| GO:00229 | 7 | electron transport chain | | |
| GO:00229 | 36 | respiratory electron transport chain | | |
| GO:00300 | 101 | metal ion transport | | |
| GO:00301 | 22 | manganese ion binding | | |
| GO:00301 | 21 | molybdenum ion binding | | |
| GO:00301 | 18 | protein catabolic process | | |
| GO:00301 | 883 | pyridoxal phosphate binding | | |
| GO:00302 | 14 | enzyme regulator activity | | |
| GO:00302 | 1 | cellulose biosynthetic process | | |
| GO:00302 | 4 | cellulose catabolic process | | |
| GO:00302 | 192 | carbohydrate binding | | |
| GO:00302 | 2 | protein secretion by the type IV secretion system | | |
| GO:00302 | 42 | lipid glycosylation | | |
| GO:00302 | 1 | chromosome condensation | | |
| GO:00302 | 1 | tetrahydromethanopterin S-methyltransferase activity | | |
| GO:00302 | 932 | outer membrane-bounded periplasmic space | | |
| GO:00304 | 4 | sporulation resulting in formation of a cellular spore | | |
| GO:00304 | 6 | tRNA methylation | | |
| GO:00305 | 2 | pectinesterase activity | | |
| GO:00306 | 2 | bacterial-type flagellum basal body, rod | | |
| GO:00309 | 1 | protein splicing | | |
| GO:00309 | 42 | potassium ion binding | | |
| GO:00309 | 417 | thiamine pyrophosphate binding | | |
| GO:00309 | 119 | mismatched DNA binding | | |
| GO:00310 | 6 | extracellular matrix | | |
| GO:00311 | 23 | rRNA methylation | | |
| GO:00313 | 12 | organic acid phosphorylation | | |
| GO:00314 | 4 | gas vesicle | | |
| GO:00314 | 6 | gas vesicle organization | | |
| GO:00314 | 145 | cobalamin binding | | |
| GO:00315 | 6 | tRNA (m1A) methyltransferase complex | | |
| GO:00315 | 23 | regulation of DNA-templated transcription, termination | | |
| GO:00315 | 18 | transcription antitermination | | |
| GO:00316 | 1 | G-protein beta/gamma-subunit complex binding | | |
| GO:00322 | 54 | methylation | | |
| GO:00323 | 60 | molybdopterin cofactor biosynthetic process | | |
| GO:00327 | 5 | DNA methylation on adenine | | |
| GO:00327 | 35 | regulation of DNA-templated transcription, elongation | | |
| GO:00329 | 2 | regulation of barrier septum assembly | | |
| GO:00330 | 110 | tetrapyrrole biosynthetic process | | |
| GO:00331 | 8 | proton-transporting two-sector ATPase complex, proton-transporting domain | | |
| GO:00331 | 1 | proton-transporting two-sector ATPase complex, catalytic domain | | |
| GO:00331 | 4 | proton-transporting V-type ATPase, V0 domain | | |
| GO:00335 | 16 | fatty acid beta-oxidation using acyl-CoA dehydrogenase | | |
| GO:00335 | 13 | high-affinity iron permease complex | | |
| GO:00337 | 11 | preQ1 synthase activity | | |
| GO:00337 | 15 | peptide-methionine (R)-S-oxide reductase activity | | |
| GO:00338 | 19 | pyridoxine 5'-phosphate synthase activity | | |
| GO:00338 | 1 | ribonuclease T2 activity | | |
| GO:00339 | 1 | ectoine synthase activity | | |
| GO:00341 | 1 | D-galactonate catabolic process | | |
| GO:00342 | 1 | ion transmembrane transport | | |
| GO:00342 | 2 | nicotinamide riboside transmembrane transporter activity | | |
| GO:00342 | 2 | nicotinamide riboside transport | | |
| GO:00344 | 2 | U6 snRNA 3'-end processing | | |
| GO:00349 | 1 | histone lysine methylation | | |
| GO:00354 | 5 | gluconate transmembrane transport | | |
| GO:00354 | 2 | phosphate ion transmembrane transport | | |
| GO:00354 | 4 | cyclic-di-GMP binding | | |
| GO:00354 | 15 | nickel cation transmembrane transport | | |
| GO:00355 | 179 | intracellular signal transduction | | |
| GO:00363 | 54 | racemase activity, acting on amino acids and derivatives | | |
| GO:00364 | 1 | ubiquitinyl hydrolase activity | | |
| GO:00421 | 73 | nitrate assimilation | | |
| GO:00421 | 31 | fructose 1,6-bisphosphate 1-phosphatase activity | | |
| GO:00421 | 5 | lipoprotein metabolic process | | |
| GO:00421 | 26 | lipoprotein biosynthetic process | | |
| GO:00422 | 1 | tissue regeneration | | |
| GO:00422 | 20 | ribosome biogenesis | | |
| GO:00422 | 9 | nitrite reductase (cytochrome, ammonia-forming) activity | | |
| GO:00423 | 42 | cellular modified amino acid biosynthetic process | | |
| GO:00425 | 2 | cell wall modification | | |
| GO:00425 | 74 | pteridine-containing compound metabolic process | | |
| GO:00425 | 11 | phosphoric ester hydrolase activity | | |
| GO:00425 | 89 | periplasmic space | | |
| GO:00426 | 40 | poly-hydroxybutyrate biosynthetic process | | |
| GO:00426 | 254 | ATPase activity, coupled to transmembrane movement of substances | | |
| GO:00426 | 6 | thylakoid membrane | | |
| GO:00427 | 3 | defense response to bacterium | | |
| GO:00427 | 13 | ATP synthesis coupled electron transport | | |
| GO:00428 | 31 | protein homodimerization activity | | |
| GO:00428 | 12 | vitamin B6 biosynthetic process | | |
| GO:00428 | 12 | pyridoxal phosphate biosynthetic process | | |

| | | | | |
|---|---|---|---|---|
| GO:00429 | 1 | alkylphosphonate transport | | |
| GO:00429 | 6 | benzoate transport | | |
| GO:00429 | 6 | benzoate transporter activity | | |
| GO:00430 | 151 | tRNA aminoacylation | | |
| GO:00430 | 17 | peptide biosynthetic process | | |
| GO:00431 | 9 | precorrin-2 dehydrogenase activity | | |
| GO:00431 | 25 | ATP-dependent 3'-5' DNA helicase activity | | |
| GO:00431 | 6 | Gram-negative-bacterium-type cell outer membrane assembly | | |
| GO:00431 | 154 | cation binding | | |
| GO:00431 | 55 | ATP-binding cassette (ABC) transporter complex | | |
| GO:00432 | 10 | intracellular membrane-bounded organelle | | |
| GO:00434 | 4 | urea catabolic process | | |
| GO:00435 | 59 | ADP binding | | |
| GO:00435 | 82 | sequence-specific DNA binding | | |
| GO:00437 | 7 | adenosylcobinamide kinase activity | | |
| GO:00437 | 21 | acyl-phosphate glycerol-3-phosphate acyltransferase activity | | |
| GO:00442 | 27 | cellular metabolic process | | |
| GO:00442 | 17 | primary metabolic process | | |
| GO:00442 | 54 | cellular carbohydrate metabolic process | | |
| GO:00443 | 10 | sodium-dependent phosphate transport | | |
| GO:00447 | 1 | bacterial-type flagellum assembly | | |
| GO:00447 | 3 | bacterial-type flagellum organization | | |
| GO:00450 | 55 | glycerolipid biosynthetic process | | |
| GO:00451 | 2 | electron transporter, transferring electrons within the cyclic electron transport pathway of photosynthesis activity | | |
| GO:00452 | 1 | capsule polysaccharide biosynthetic process | | |
| GO:00452 | 42 | proton-transporting ATP synthase complex, catalytic core F(1) | | |
| GO:00452 | 12 | proton-transporting ATP synthase complex, coupling factor F(o) | | |
| GO:00453 | 4 | acyl-[acyl-carrier-protein] desaturase activity | | |
| GO:00454 | 75 | cell redox homeostasis | | |
| GO:00454 | 3 | xylan catabolic process | | |
| GO:00455 | 10 | dynein binding | | |
| GO:00458 | 14 | negative regulation of transcription, DNA-templated | | |
| GO:00458 | 5 | positive regulation of transcription, DNA-templated | | |
| GO:00459 | 3 | positive regulation of transcription from RNA polymerase II promoter | | |
| GO:00460 | 20 | dUTP metabolic process | | |
| GO:00461 | 22 | glycerol-3-phosphate catabolic process | | |
| GO:00463 | 3 | aminoglycoside 3-N-acetyltransferase activity | | |
| GO:00463 | 20 | L-arabinose metabolic process | | |
| GO:00464 | 3 | organomercury catabolic process | | |
| GO:00464 | 3 | chorismate metabolic process | | |
| GO:00464 | 24 | 4-hydroxy-3-methylbut-2-en-1-yl diphosphate synthase activity | | |
| GO:00464 | 9 | L-cysteine metabolic process | | |
| GO:00465 | 2 | 2,3-bisphosphoglycerate-independent phosphoglycerate mutase activity | | |
| GO:00465 | 20 | alpha-L-arabinofuranosidase activity | | |
| GO:00465 | 3 | alpha-glucuronidase activity | | |
| GO:00466 | 2 | tetrahydrofolate metabolic process | | |
| GO:00466 | 14 | response to antibiotic | | |
| GO:00466 | 4 | response to copper ion | | |
| GO:00468 | 60 | phosphatidylinositol phosphorylation | | |
| GO:00468 | 726 | metal ion binding | | |
| GO:00468 | 59 | metal ion transmembrane transporter activity | | |
| GO:00469 | 2 | intracellular transport | | |
| GO:00469 | 54 | transferase activity, transferring acyl groups, acyl groups converted into alkyl on transfer | | |
| GO:00469 | 32 | transition metal ion binding | | |
| GO:00469 | 61 | proton-transporting ATP synthase activity, rotational mechanism | | |
| GO:00469 | 1 | phytochelatin biosynthetic process | | |
| GO:00469 | 43 | proton-transporting ATPase activity, rotational mechanism | | |
| GO:00469 | 123 | protein dimerization activity | | |
| GO:00472 | 1 | tetrahydrodipicolinate N-acetyltransferase activity | | |
| GO:00473 | 12 | CDP-glycerol glycerophosphotransferase activity | | |
| GO:00475 | 84 | 2-methylcitrate dehydratase activity | | |
| GO:00479 | 8 | hydroxybutyrate-dimer hydrolase activity | | |
| GO:00480 | 1 | mRNA 5'-UTR binding | | |
| GO:00480 | 2 | monosaccharide binding | | |
| GO:00480 | 57 | cofactor binding | | |
| GO:00480 | 157 | quinone binding | | |
| GO:00484 | 8 | threonine-phosphate decarboxylase activity | | |
| GO:00485 | 7 | rhythmic process | | |
| GO:00500 | 19 | malonyl-CoA decarboxylase activity | | |
| GO:00500 | 4 | methylaspartate mutase activity | | |
| GO:00501 | 1 | oleate hydratase activity | | |
| GO:00502 | 4 | propanediol dehydratase activity | | |
| GO:00503 | 36 | undecaprenyl-diphosphatase activity | | |
| GO:00503 | 1 | ureidoglycolate lyase activity | | |
| GO:00505 | 19 | 2-C-methyl-D-erythritol 4-phosphate cytidylyltransferase activity | | |
| GO:00505 | 11 | 2-phosphosulfolactate phosphatase activity | | |
| GO:00505 | 6 | protein-glutamine glutaminase activity | | |
| GO:00505 | 31 | 4-hydroxythreonine-4-phosphate dehydrogenase activity | | |
| GO:00506 | 483 | flavin adenine dinucleotide binding | | |
| GO:00506 | 129 | NADP binding | | |
| GO:00506 | 468 | coenzyme binding | | |
| GO:00507 | 21 | thymidylate synthase (FAD) activity | | |
| GO:00509 | 1 | regulation of chemotaxis | | |
| GO:00509 | 29 | dimethylallyl diphosphate biosynthetic process | | |
| GO:00510 | 27 | unfolded protein binding | | |
| GO:00510 | 21 | chaperone binding | | |
| GO:00511 | 9 | glucose 6-phosphate metabolic process | | |
| GO:00511 | 7 | cofactor biosynthetic process | | |
| GO:00512 | 36 | protein insertion into membrane | | |
| GO:00512 | 9 | protein oligomerization | | |
| GO:00512 | 7 | protein tetramerization | | |
| GO:00512 | 545 | NAD binding | | |
| GO:00513 | 147 | cell division | | |
| GO:00513 | 17 | chromosome separation | | |
| GO:00515 | 1045 | iron-sulfur cluster binding | | |
| GO:00515 | 152 | 2 iron, 2 sulfur cluster binding | | |
| GO:00515 | 64 | 4 iron, 4 sulfur cluster binding | | |
| GO:00516 | 25 | proteolysis involved in cellular protein catabolic process | | |
| GO:00517 | 29 | 4-hydroxy-3-methylbut-2-en-1-yl diphosphate reductase activity | | |
| GO:00517 | 7 | negative regulation of cell division | | |
| GO:00519 | 65 | peroxiredoxin activity | | |
| GO:00526 | 4 | arabinosyltransferase activity | | |
| GO:00526 | 2 | carboxylic ester hydrolase activity | | |
| GO:00528 | 24 | ADP-dependent NAD(P)H-hydrate dehydratase activity | | |

| | | | | |
|---|---|---|---|---|
| GO:00550 | 6 | copper ion homeostasis | | |
| GO:00550 | 1905 | transmembrane transport | | |
| GO:00551 | 7777 | oxidation-reduction process | | |
| GO:00610 | 27 | membrane organization | | |
| GO:00700 | 71 | serine-type exopeptidase activity | | |
| GO:00704 | 11 | NADPH binding | | |
| GO:00704 | 28 | NAD+ binding | | |
| GO:00704 | 2 | rRNA base methylation | | |
| GO:00705 | 5 | uridylyltransferase activity | | |
| GO:00715 | 13 | cell wall organization | | |
| GO:00717 | 4 | Actinobacterium-type cell wall biogenesis | | |
| GO:00717 | 1 | aldehyde decarbonylase activity | | |
| GO:00718 | 37 | potassium ion transmembrane transport | | |
| GO:00719 | 1 | FAD binding | | |
| GO:00719 | 41 | bacterial-type flagellum-dependent cell motility | | |
| GO:19022 | 2 | regulation of bacterial-type flagellum assembly | | |
| GO:19022 | 1 | negative regulation of bacterial-type flagellum assembly | | |
| GO:19026 | 2 | p-aminobenzoyl-glutamate transmembrane transport | | |
